# Assessing state partner use of the Model Aquatic Health Code (MAHC): A cross comparison of five states with varying degrees of self-reported adoption status

**DOI:** 10.1371/journal.pwat.0000276

**Published:** 2024-08-08

**Authors:** Patrick Vander Kelen, Joseph P. Laco, Shannon McClenahan, Christopher Fletcher, Brian Hubbard

**Affiliations:** 1 Division of Environmental Health Science and Practice, National Center for Environmental Health, Centers for Disease Control and Prevention, Atlanta, Georgia, United States of America; 2 Oak Ridge Institute for Science and Education, Oak Ridge, Tennessee, United States of America

## Abstract

Despite the development of the Model Aquatic Health Code (MAHC), U.S. public health departments and aquatics agencies face obstacles in incorporating this guidance into their pool codes. A cross comparison of five state pool codes with the MAHC was conducted to quantify MAHC incorporation into these state codes. The proportion of MAHC code agreement with state codes in this study had a range of 14%–86%. Only 2% of all the MAHC codes available were present in all five state codes, conversely, 12% of the MAHC codes were not found in any state. These differences in code agreement highlight the challenge of measuring MAHC effectiveness at the national level. To improve aquatic safety at a national level, a potential solution is development and use of common core elements in state and local pool codes. Once there is a basis for code comparisons across states, public health programs can investigate whether core MAHC codes result in reduced waterborne illness outbreaks, drowning incidents, injuries from pool chemicals, health outcomes from exposure to disinfection by-products, and swimming-related emergency department visits.

## Introduction

Prior to the development of the Model Aquatic Health Code (MAHC), U.S. public health departments and aquatics agencies had little federal guidance to ensure the health and safety of swimmers and staff. State and local health department staff used their own existing pool codes of varying length and specificity and used limited and fragmented research to update and adopt new codes. This changed in 2014 when the Centers for Disease Control and Prevention (CDC) released the first edition of the MAHC [1]. Public health agencies then had access to science-based recommendations and best practices to guide updates to their existing code or to adopt sections of the MAHC to address new and emerging aquatic venues and practices.

The MAHC is a set of comprehensive guidelines to promote health and safety in public aquatic facilities, and provides up-to-date science and practices to address the adverse public health outcomes associated with drowning and near-drowning [2, 3], pool chemical exposures [4–9], and recreational water illnesses [5].

Since its initial publication, the MAHC has been revised several times and is currently on its 4^th^ edition. The revision process occurs on a 3-year cycle and is overseen by the CDC and the Council for the Model Aquatic Health Code, a nonprofit organization promoting health and safety at public aquatic facilities. The purpose behind the development and continual revision of the MAHC is to provide the public with uniform national guidance that state and local public health agencies can use to write or update their pool codes in part or in full as fits their jurisdiction’s needs [10].

At the time of this study in 2019, we tracked five majority MAHC adoptions and seven partial MAHC adoptions by states. Twenty-two states or counties were considering adopting the MAHC. Regardless of the adoption status reported by states or counties, there has been uncertainty as to what defines or differentiates majority adoption, partial adoption and considering adoption. This work serves to quantify the differences in order to improve measuring MAHC adoption.

The MAHC has been publicly available for 9 years, but little is known about how many states or jurisdictions have incorporated it into their pool codes. Some publications documented how states have incorporated the MAHC into their pool codes and the time required to complete comparisons, but they have been on an individual state level describing their process [11, 12]. There is a need to determine which states incorporated the MAHC into their pool codes and how much of the MAHC was used. Understanding the extent to which jurisdictions incorporate the MAHC may allow for a better comparison of how MAHC use can impact health between those jurisdictions. This study aims to determine and describe which parts of the MAHC are used by states and which are not. This will be the first publication to quantify MAHC agreement with state codes.

## Materials and methods

### MAHC code book development

We constructed a code book to determine which parts of the MAHC were present or not present in state codes. We used the MAHC as our basis for comparison so there was a stable denominator from which to compare across states with varying code lengths. We built the code book by converting the 2018 (3^rd^ Edition) MAHC code (S1 Text) into an Excel (Microsoft, Redmond WA) spreadsheet (S1 Data). To do this, we followed the MAHC numbering system. Each section (e.g., 6.3.3.3 Pre-service Plan), paragraph (e.g., 6.3.3.3.5 CPR/AED and First Aid Certificate) and subparagraph (e.g., 6.3.3.3.5.1 Copies Maintained) with a numbered item in the MAHC became a row (a “code”) in the Excel spreadsheet. We then eliminated any numbered items without associated text (i.e., it functioned as a title only). Subparts (e.g., 6.3.3 Safety Plan) were used to create code groups to categorize groups of individual codes. We did not split up lists or tables that included multiple items under one numbered MAHC code. Once code book formatting was complete, it was then uploaded into ATLAS.ti version 8.0 (Cleverbridge, Inc., Chicago IL), a qualitative analysis software that aided the coding process, documentation, and analysis of the large amount of MAHC textual data.

### State pool code selection

Historically, CDC categorized MAHC adoption status based on self-reporting of how much of the MAHC was used within state codes. These adoption statuses were defined as majority adopted (≥ 75%), partially adopted (1–74%), or considering adoption. Five states were chosen for this comparison based on their self-reported adoption and state-rule status, and MAHC adoption status at the time of this study: Arizona (considering adoption) (S2 Text), Delaware (partially adopted) (S3 Text), Florida (partially adopted) (S4 Text), Georgia (partially adopted) (S5 Text), and New Mexico (majority adopted) (S6 Text). We found state pool codes on state pool regulation websites and downloaded them to assess any cross references to other state regulations that pertained to pool codes (e.g., building codes). If we found cross references pertinent to state pool codes, we appended those documents to the existing state pool codes for inclusion in the cross comparison. All state pool codes were then uploaded into ATLAS.ti to assist comparison with the MAHC.

### Code comparison

Each state’s code was manually examined by a team of two to three MAHC subject matter experts. All five state code documents were analyzed line by line by each member of the team. Individual team members reviewed the MAHC code and determined if there was an agreement between the state and MAHC codes. Exact code language was not required to be considered a code match. For example, if the state code was more stringent than the MAHC, it was considered a code match because it offered the same or greater public health protection. However, if the meaning of the state code was less restrictive than the MAHC, it was not considered a match. The team discussed each potential match and came to consensus as to whether the state regulation and the MAHC code were in agreement. This coding system allowed for many-to-one and one-to-many relationships, meaning multiple MAHC codes could be cited for a single state code item and each MAHC code item could be used in multiple state codes. This system focused on documenting whether the state pool codes corresponded to, and aligned with, the MAHC codes; therefore, any coding duplicates were removed during data analysis. In this study, we did not use unique sections found in state codes that were not present in the MAHC because the focus was on use of MAHC code language, not the individual language of the state codes.

## Results and discussion

### MAHC code book

#### State codes.

The code book, derived from the MAHC code language, contained 2,119 individual codes and 121 different code groups. The length of state codes ranged from 20 to 325 pages (mean = 112 pages, median = 56 pages) (Table 1). The number of personnel hours attributed to each state’s code did not correspond to the length of the state code itself. Florida had the largest number of hours (130) despite their state code only being 56 pages, and Arizona had the second largest number of hours (88) and the shortest state code (22 pages). Delaware and Georgia were the only states that had a single source for all their pool codes; pool codes in the other states were cross referenced in other documents that required appending (e.g., building codes).

### Code comparison

The number of individual MAHC codes found matching state codes varied, with a mean of 693, range of 297 through 1,814, and a median of 436. The proportion of MAHC code agreement with state codes in this study had a range of 14%–86% (Table 2). New Mexico, the majority adopter, had the largest MAHC code agreement in their state code (86%). Arizona, considering adoption, had a MAHC code agreement of 14%. The three remaining states (Delaware, Florida, and Georgia), all partial adopters, together had an average of 21% agreement.

Only 51 individual MAHC codes were found within all five state codes (Table 3). This means only 2% of all the MAHC codes available were present in all states. Conversely, 12% or 252 of the MAHC codes were not found in any state. The proportion of MAHC codes found in three or more states was 22%.

**Fig 1.**
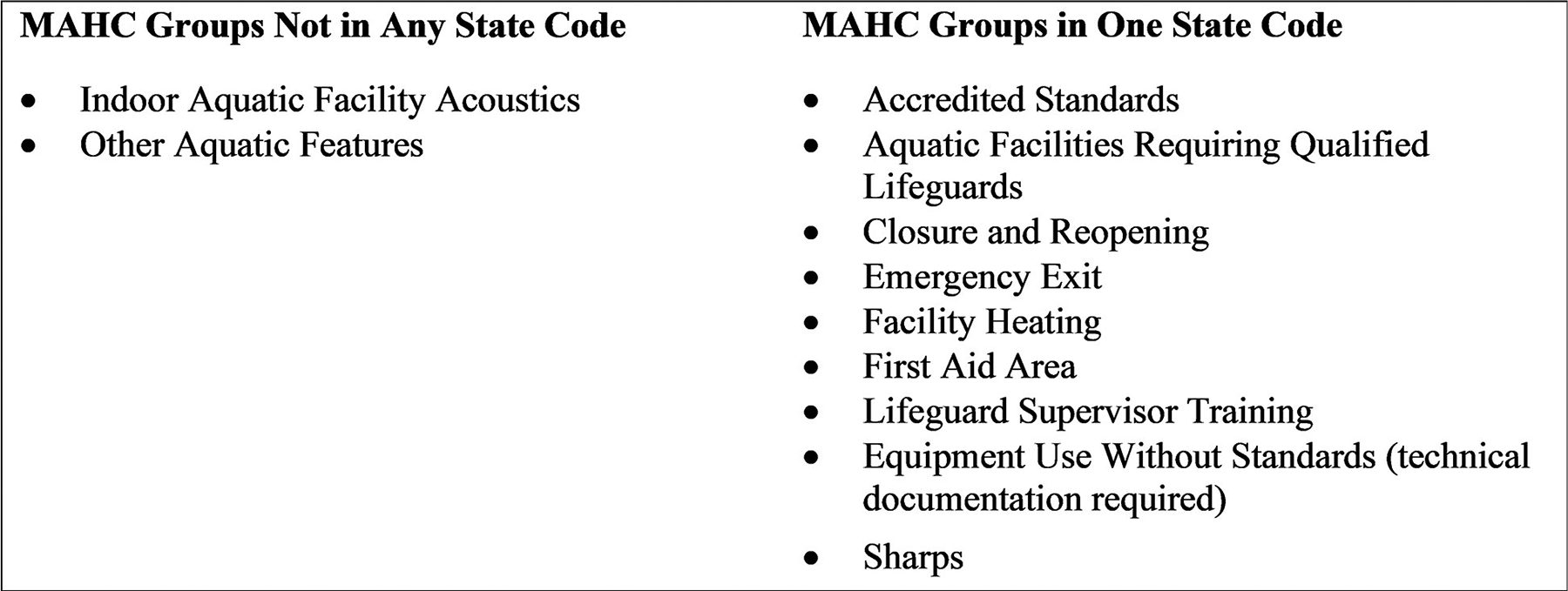
MAHC groups underrepresented in state pool codes.

Forty-three MAHC groups were found in all five state codes, representing 36% of the total groups. Only two groups, Facility Acoustics and Other Aquatic Features (Fig 1), were not present in any of the state codes. The proportion of MAHC groups found in three or more states was 77%.

At the time of this study, the MAHC had been publicly available for 8 years and during that time, 12 states self-reported they had adopted portions of the MAHC into their pool codes. MAHC adoption was being categorized through self-reporting. However, as this study shows, there is not a large difference in the amount of agreement to MAHC codes between states considering adoption (n = 1; 14%) and those that are partial adopters (14%, 21%, and 29%). This lack of differentiation makes self-reporting adoption unreliable and therefore questions the validity of the adoption categories, self-reports, and the core concept behind adoption. There are several challenges in trying to quantify how the MAHC is used by states.

The effort of adoption is best illustrated by New Mexico, the only state to be a majority adopter of MAHC codes. It took New Mexico nearly 3 years to adopt the first version of the MAHC, even though this is not uncharacteristic of a local government legislative process to incorporate new code, and by the time they incorporated it, a new version was being released [11]. As the MAHC evolves and grows on its 3-year cycle, states that have adopted or are in the process of adoption may be a version or two behind the current edition of the MAHC.

One of the largest burdens to adoption is the time it takes to analyze and compare existing codes to the MAHC to identify needs and gaps. This study found that it took an average of 82 hours to compare a state pool code to the MAHC. These hours are reflective of efforts made by people who are extensively knowledgeable of the MAHC and do not necessarily reflect the amount of time it would take for people with varying degrees of familiarity with it. As an example, Arizona’s pool code is 20 pages, and the 2018 MAHC (3^rd^ edition) is 195 pages [10]. Unless the state of Arizona expands their pool code to roughly ten times its current size, it will not be considered as a majority adopter. However, if the state continues with its current or similar pool code, they are considered a “partial” adopter, as they already met that requirement at 14% agreement, with other states self-reporting in the same category. States should consider what would be gained from a code comparison as compared to the cost of the process.

The MAHC was developed to help programs that regulate public aquatic facilities reduce the risk of disease, injury, and drowning in their communities [1]. These programs include local health agencies and pool inspectors. As the MAHC continues to grow and expand with the aquatic sector changes, it becomes more challenging to integrate into the code enforcement process. The current MAHC update cycle keeps up with the latest science, data, trends, emerging issues, and best practices. Updates to regulations often fall on legislators and health department directors. Inspectors have the job of enforcing the rules, not creating them. Anytime there are changes to laws, adjustments must be made requiring education and training for inspectors and operators. Jurisdictions will have to make their own determination of when it is best to update their pool regulations, based on their individual needs, gaps, and priorities.

This study found that only 2% of MAHC codes were present in the five states subjected to analysis. It is important to note that the absence of a code match does not mean that there was not a state code for a particular topic. The low percentage of code matches may be the result of the MAHC standard being more protective than a state’s code. However, 12% of MAHC guidance was not found in any of the state codes we reviewed. This indicates there may be issues with how the MAHC can be generalized across state codes. In looking at the code groups, we saw that 36% of the groups were present in all five states and 77% of the code groups were present in three or more states. This shows that states have codes pertaining to the larger groups present in the MAHC but lack the detailed differences found within the individual MAHC codes. The difference seen between the individual codes and the code groups could indicate the difficulty of effectively implementing all aspects of an exhaustive code. Moreover, it is unlikely that state pool codes will grow to the size of the MAHC because some state legislative processes limit the number of new regulations than can be added to existing pool codes. For example, a state agency might not adopt a new regulatory restriction unless it simultaneously removes two or more existing regulatory restrictions.

The fact that 77% of the code groups are present in three or more states indicates there is a common aspect covered in all pool codes. Greater effort needs to be exerted to understand these common elements and their potential for integration into state and local codes. One suggestion would be to extract a subset of essential core codes from the larger MAHC and have this subset serve as the foundation of an aquatic safety program. The entire MAHC addresses safety, but not all codes address an imminent health hazard that would require immediate intervention or pool closure. Instead of having to assess 195 pages of MAHC language, a smaller subset of essential codes could reduce the cost or burden on states that want to incorporate the MAHC into their pool codes. Furthermore, a core set of codes that are stable over time would eliminate the effect new versions of the MAHC have on state adoption status (e.g., New Mexico changing from 100% when they first adopted the MAHC to 86% two versions later). The common core codes would provide a stable denominator allowing for comparisons of aquatic safety outcomes between states.

## Conclusion

The MAHC was developed as a set of guidelines to help provide science-based recommendations for aquatic safety. Incorporating the MAHC into state pools codes may be a time consuming and complicated legislative process, for example, the state of Indiana took 18 months to review individual sections of MAHC code [12]. In the case of New Mexico, even though they adopted the MAHC in its entirety, they reported the adoption process took a period of 35 months [11]. Our code comparison provides information on resources needed and the most commonly adopted code elements, which may be helpful for state and local pool programs that are deciding to update their codes with existing MAHC guidance. Our comparison of five state pool codes with the MAHC demonstrated a large difference in the amount of agreement between state pool codes and the MAHC. This large difference in code agreement highlights the challenge of measuring MAHC effectiveness at the national level. A proposed solution to improving measurability could be to develop and use a set of common core MAHC elements in state and local pool codes. Once there is a basis for code comparisons across states, public health programs can investigate whether a set of common core MAHC codes result in reduced waterborne illness outbreaks, drowning incidents, injuries from pool chemicals, health outcomes from exposure to disinfection by-products, and swimming-related emergency department visits.

## Supplementary Material

S1_Data **S1 Data. MAHC code book.** CDC constructed code book developed by converting the 2018 (3rd Edition) MAHC code language into an Excel spreadsheet.
(XLSX)

S1_Text **S1 Text. 2018 MAHC code.** 2018, third edition, Model Aquatic Health Code. (PDF)

S2_Text **S2 Text. Arizona pool codes.** Title 9, Chapter 8, 2018 Arizona administrative code for Article 8: Public and semi-public swimming pools and bathing places.
(PDF)

S3_Text **S3 Text. Delaware pool codes.** Title 18 health and safety administrative code from the Delaware Department of Health and Social Services, Division of Public Health for public swimming pools established on October 11, 2015.
(PDF)

S4_Text **S4 Text. Florida pool codes.** Florida public swimming pool and bathing places code established September 2015.
(PDF)

S5_Text **S5 Text. Georgia pool codes.** The Georgia Department of Public Health rules and regulations for public swimming pools, spas, and recreational water parks, Chapter 511–3-5 established in 2017.
(PDF)

S6_Text **S6 Text. New Mexico pool codes.** New Mexico Title 7 health, Chapter 18 aquatic venues established in August 2018.
(PDF)

## Figures and Tables

**Table 1.** State pool codes.

State Code	Appended Code Sources	Total Pages	Total Person Hours	Total Clock Hours*
New Mexico	2	325	55	31
Delaware Administrative Code	1	45	53	26.5
Florida	3	56	130	56
Georgia	1	114	84	42
Arizona Administrative Code	2	20	88	32.5

* Total clock hours are the total time the review took regardless of how many reviewers participated during the process.

**Table 2.** Percent of individual 2018 MAHC codes within each state code.

State	Adoption Status	Number of Individual MAHC Codes in Each State Code	Percent of MAHC in State Codes
New Mexico	Majority Adoption	1,814	86%
Delaware	Partially Adopted	436	21%
Florida	Partially Adopted	305	14%
Georgia	Partially Adopted	613	29%
Arizona	Considering Adoption	297	14%

Total number of MAHC codes (N) = 2,119

**Table 3.** Number of states in the study with number of MAHC codes and code groups.

Number of States	Number of MAHC Codes	Percent of Total MAHC Codes	Number of MAHC Groups	Percent of Total MAHC Groups
0	252	12%	2	2%
1	974	46%	9	7%
2	437	21%	17	14%
3	258	12%	24	20%
4	147	7%	26	21%
5	51	2%	43	36%
**Totals**	**2,119**	100%	**121**	100%

## Data Availability

All relevant data are within the paper and its Supporting Information files.
