# Supplementary material for: Assessing state partner use of the Model Aquatic Health Code (MAHC): A cross comparison of five states with varying degrees of self-reported adoption status
Source: PLOS Water. Author manuscript; Available in PMC 2025 Jul 11. (PMC12247143; doi:10.1371/journal.pwat.0000276)
Supplement: S2_Text — S2 Text. Arizona pool codes. Title 9, Chapter 8, 2018 Arizona administrative code for Article 8: Public and semi-public swimming pools and bathing places. (PDF) [file NIHMS2046419-supplement-S2_Text.pdf]

c. Is not in the same room as food preparation areas, as specified in 9 A.A.C. 8, Article 1;
2. May be removed from the animal's habitat at the direction of a teacher;
3. When out of the animal's habitat, is under the control of a teacher or a student of the school, if the animal is:
   a. A bird, reptile, amphibian, or invertebrate;
   b. A large mammal, such as a horse, sheep, pig, goat, or cow;
   c. A rabbit or hare; or
   d. A rodent, such as a mouse, rat, hamster, guinea pig, or gerbil;
4. Has a current immunization against rabies, if the animal is a dog, cat or ferret, as documented by:
   a. A dog license issued by a state or county agency;
   b. A rabies immunization certificate from a veterinarian licensed under 3 A.A.C. 11;
   c. A receipt for veterinary services, showing the administration of a rabies vaccine; or
   d. A written statement attesting to the current immunization of the animal against rabies; and
5. Is not:
   a. A non-human primate;
   b. A deer mouse, or other wild mouse of the genus *Peromyscus*; and
   c. A bat, skunk, raccoon, fox, wolf-hybrid or coyote, except when brought into a classroom for an educational display, as defined in R12-4-401, by a person who has complied with provisions in 12 A.A.C. 4, Article 4, obtained a permit or license issued by the Arizona Game and Fish Department, and is experienced in handling the animal.

B. A responsible person shall ensure that a room, in which an animal in a school is kept:
1. Is free of animal waste, except in the habitat; and
2. Has:
   a. A lavatory with soap and single-use paper towels or air hand dryers; or
   b. A product to sanitize the hands of an individual who touches an animal or its habitat.

Historical Note

New Section made by final rulemaking at 12 A.A.R. 282, effective March 11, 2006 (Supp. 06-1).

R9-8-710. Pest Control

A responsible person shall ensure that indoor classroom and non-classroom areas are kept free of insects and rodents, except when the insects or rodents are being kept as specified in R9-8-709 or are food for animals being kept as specified in R9-8-709.

Historical Note

New Section made by final rulemaking at 12 A.A.R. 282, effective March 11, 2006 (Supp. 06-1).

R9-8-711. Inspections

The Department shall inspect:
1. A school for compliance with this Article at least once each calendar year, and
2. Areas of a school pertinent to the details of a complaint upon receipt of the complaint.

Historical Note

Section repealed; new Section made by final rulemaking at 12 A.A.R. 282, effective March 11, 2006 (Supp. 06-1).

R9-8-712. Repealed

Historical Note

Section repealed by final rulemaking at 12 A.A.R. 282, effective March 11, 2006 (Supp. 06-1).

R9-8-713. Repealed

Historical Note

Section repealed by final rulemaking at 12 A.A.R. 282, effective March 11, 2006 (Supp. 06-1).

R9-8-714. Repealed

Historical Note

Section repealed by final rulemaking at 12 A.A.R. 282, effective March 11, 2006 (Supp. 06-1).

R9-8-715. Repealed

Historical Note

Section repealed by final rulemaking at 12 A.A.R. 282, effective March 11, 2006 (Supp. 06-1).

R9-8-716. Repealed

Historical Note

Section repealed by final rulemaking at 12 A.A.R. 282, effective March 11, 2006 (Supp. 06-1).

R9-8-717. Repealed

Historical Note

Section repealed by final rulemaking at 12 A.A.R. 282, effective March 11, 2006 (Supp. 06-1).

ARTICLE 8. PUBLIC AND SEMIPUBLIC SWIMMING POOLS AND BATHING PLACES

R9-8-801. Definitions

In this Article, unless otherwise specified:
1. "Artificial lake" has the same meaning as in A.A.C. R18-5-201.
2. "Backwash" has the same meaning as in A.A.C. R18-5-201.
3. "Bathing place" means a volume of water that is used for water contact recreation.
4. "Clean" means free from slime, scum, dirt, or other debris.
5. "Deck" has the same meaning as in A.A.C. R18-5-201.
6. "Department" means the Arizona Department of Health Services.
7. "Incontinent" means unable to restrain a bowel movement.
8. "Local health department" has the same meaning as in R9-18-101.
9. "Maximum bathing load" has the same meaning as in A.A.C. R18-5-201.
10. "Natural bathing place" has the same meaning as in A.A.C. R18-5-201.
11. "Operate" has the same meaning as in A.A.C. R18-5-201.
12. "Operator" means an individual who owns, runs, maintains, or otherwise controls or directs the functioning of a bathing place.
13. "Oxidation-reduction potential" means the measurement in millivolts of the potential for transfer of electrons from one atom or molecule to another in water.
14. "Potable water" has the same meaning as in A.A.C. R18-5-201.
15. "Ppm" means parts per million.
16. "Private residential spa" has the same meaning as in A.A.C. R18-5-201.
17. "Private residential swimming pool" has the same meaning as in A.A.C. R18-5-201.
18. "Public health services district" has the same meaning as "district" in A.R.S. § 48-5801.

19. "Public spa" has the same meaning as in A.A.C. R18-5-201.
20. "Public swimming pool" has the same meaning as in A.A.C. R18-5-201.
21. "Regulatory authority" means the Department or a local health department or public health services district operating under a delegation of authority from the Department.
22. "Sanitary facility" means a designated area that includes a toilet, urinal, sink, or shower.
23. "Scum" means a film that forms on the surface of water.
24. "Semi-artificial bathing place" means a lake, pond, river, stream, swimming hole, or hot spring that is modified to be used for water contact recreation.
25. "Semipublic spa" has the same meaning as in A.A.C. R18-5-201.
26. "Semipublic swimming pool" has the same meaning as in A.A.C. R18-5-201.
27. "Shallow area" has the same meaning as in A.A.C. R18-5-201.
28. "Shock treatment" means adding chlorine to water to elevate the free chlorine residual to 20 ppm and destroy ammonia and nitrogenous and organic contaminants in the water.
29. "Slime" means a glutinous or viscous liquid matter.
30. "Spa" has the same meaning as in A.A.C. R18-5-201.
31. "Surface water" has the same meaning as in A.A.C. R18-11-101.
32. "Swimming pool" has the same meaning as in A.A.C. R18-5-201.
33. "Turnover rate" has the same meaning as in A.A.C. R18-5-201.
34. "Wading pool" has the same meaning as in A.A.C. R18-5-201.
35. "Water circulation system" has the same meaning as in A.A.C. R18-5-201.
36. "Water circulation system components" has the same meaning as in A.A.C. R18-5-201.
37. "Water fountain" means a bathing place that functions by using mechanical means to propel a stream of water out of an opening or structure.
38. "Water contact recreation" means an activity for enjoyment in which an individual wets all or part of the individual's body with water.

Historical Note

New Section made by final rulemaking at 8 A.A.R. 3645, effective August 9, 2002 (Supp. 02-3).

R9-8-802. Applicability

This Article does not apply to:

1. A private residential swimming pool,
2. A private residential spa,
3. A bathing place used for medical treatment or physical therapy supervised by licensed medical personnel, or
4. A body of water that is not used as a bathing place.

Historical Note

New Section made by final rulemaking at 8 A.A.R. 3645, effective August 9, 2002 (Supp. 02-3).

R9-8-803. Public and Semipublic Swimming Pool and Spa Water Quality and Disinfection Standards

A. An operator of a public or semipublic swimming pool or spa shall ensure that:
1. The swimming pool or spa is filled only with potable water;
2. The water in the swimming pool or spa:
   a. Complies with the water quality standards in this Section when the swimming pool or spa is open for water contact recreation;
   b. Maintains a pH of between 7.2 and 7.8;
   c. Maintains a total alkalinity of between 60 and 100 ppm; and
   d. Is sufficiently clear so that the main drain in the swimming pool or spa is visible from the deck of the swimming pool or spa;
3. The surface of the water in the swimming pool or spa is free from scum and floating debris;
4. The bottom and sides of the swimming pool or spa are free from sediment, dirt, slime, and algae;
5. The chemical disinfection level, pH, total alkalinity, and temperature of the water is tested at least once daily; and
6. A daily operating log that includes the results of the tests in subsection (A)(5) is maintained for 12 months from the date of the test and is available to a regulatory authority or a member of the public upon request.

B. An operator of a public or semipublic swimming pool or spa:
1. Shall not use chloramine as a primary disinfectant in the swimming pool or spa;
2. Shall not add gaseous disinfectant directly into the swimming pool;
3. Shall not add dry or liquid disinfectant directly into the swimming pool or spa for routine disinfection; and
4. May add dry or liquid disinfectant directly into the swimming pool or spa for shock treatment.

C. An operator of a public or semipublic swimming pool or spa using chlorinated isocyanurates or cyanuric acid stabilizer for disinfection and stabilization in the swimming pool or spa shall ensure that the water in the swimming pool or spa maintains an oxidation-reduction potential equal to or greater than 650 millivolts and that cyanuric acid levels, whether from chlorinated isocyanurates or from the separate addition of cyanuric acid stabilizer, do not exceed 150 ppm.

D. An operator of a public or semipublic swimming pool shall ensure that the water in the swimming pool meets one of the following chemical disinfection standards:
1. A free chlorine residual between 1.0 and 3.0 ppm as measured by the N, N-Diethyl-p-phenylenediamine test,
2. A free bromine residual between 2.0 and 4.0 ppm as measured by the N, N-Diethyl-p-phenylenediamine test, or
3. An oxidation-reduction potential equal to or greater than 650 millivolts.

E. An operator of a public or semipublic spa shall ensure that:
1. A chlorine gas disinfection system is not used in the spa;
2. The water temperature in the spa does not exceed 40EC; and
3. The water in the spa meets one of the following chemical disinfection standards:
   a. A free chlorine residual between 3.0 and 5.0 ppm as measured by the N, N-Diethyl-p-phenylenediamine test,
   b. A free bromine residual between 3.0 and 5.0 ppm as measured by the N, N-Diethyl-p-phenylenediamine test, or
   c. An oxidation-reduction potential equal to or greater than 650 millivolts.

Historical Note

New Section made by final rulemaking at 8 A.A.R. 3645, effective August 9, 2002 (Supp. 02-3).

R9-8-804. Public and Semipublic Swimming Pool and Spa Water Circulation Requirements

A. An operator of a public or semipublic swimming pool or spa shall ensure that:
1. The swimming pool or spa water circulation system complies with the water circulation requirements in 18 A.A.C. 5, Article 2; and
2. The swimming pool or spa is equipped with:
    a. A flow meter as specified in 18 A.A.C. 5, Article 2; and
    b. A vacuum cleaning system as specified in 18 A.A.C. 5, Article 2.

B. An operator may draw water from a swimming pool for a water slide or a water fountain without filtering or disinfecting the water.

Historical Note

New Section made by final rulemaking at 8 A.A.R. 3645, effective August 9, 2002 (Supp. 02-3).

R9-8-805. Public and Semipublic Swimming Pool and Spa Maximum Bathing Loads

An operator of a public or semipublic swimming pool or spa shall ensure that the maximum bathing load, as specified in 18 A.A.C. 5, Article 2, is not exceeded.

Historical Note

New Section made by final rulemaking at 8 A.A.R. 3645, effective August 9, 2002 (Supp. 02-3).

R9-8-806. Posting Requirements

An operator of a public or semipublic swimming pool or spa shall ensure that a sign is posted within 50 feet of the swimming pool or spa, that includes the following instructions:
1. Use the toilet before entering the pool or spa;
2. Take a shower before entering the pool or spa;
3. Do not enter the pool with a cold, skin or other body infection, open wound, diarrhea, or any other contagious condition;
4. If incontinent, wear tight fitting rubber or plastic pants or a swim diaper; and
5. Observe all safety regulations.

Historical Note

New Section made by final rulemaking at 8 A.A.R. 3645, effective August 9, 2002 (Supp. 02-3).

R9-8-807. Public and Semipublic Swimming Pool and Spa and Bathing Place Facility Sanitation

A. An operator of a public or semipublic swimming pool or spa shall ensure that a sanitary facility at the public or semipublic swimming pool is maintained in a clean condition.

B. An operator of a public or semipublic swimming pool or bathing place shall provide a soap dispenser with liquid or powdered soap at each sink in a sanitary facility.

Historical Note

New Section made by final rulemaking at 8 A.A.R. 3645, effective August 9, 2002 (Supp. 02-3).

R9-8-808. Bathing Place Towels

If a towel is provided by a bathing place to an individual using the bathing place, an operator of the bathing place shall ensure that the towel is washed with soap or detergent and hot water and thoroughly dried after each individual use.

Historical Note

New Section made by final rulemaking at 8 A.A.R. 3645, effective August 9, 2002 (Supp. 02-3).

R9-8-809. Disposal of Sewage, Filter Backwash, and Wasted Swimming Pool or Spa Water

An operator of a public or semipublic swimming pool or spa shall ensure that sewage, filter backwash, and swimming pool or spa water are disposed of according to A.A.C. R18-5-236.

Historical Note

New Section made by final rulemaking at 8 A.A.R. 3645, effective August 9, 2002 (Supp. 02-3).

R9-8-810. Fecal Contamination in Public and Semipublic Swimming Pools and Spas

A. If solid feces are found in a public or semipublic swimming pool or spa, an operator of the swimming pool or spa shall ensure that:
1. Each individual in the swimming pool or spa exits the swimming pool or spa and the swimming pool or spa is closed,
2. The feces in the swimming pool or spa are removed and disposed of in a toilet,
3. The chemical disinfection level of the water in the swimming pool or spa is tested to determine whether the water complies with the water quality and disinfection standards in R9-8-803, and
4. The swimming pool or spa is not reopened until a test conducted under subsection (A)(3) indicates that the water complies with the water quality and disinfection standards in R9-8-803.

B. If liquid feces are found in a public or semipublic swimming pool or spa, an operator of the swimming pool or spa shall ensure that:
1. Each individual in the swimming pool or spa exits the swimming pool or spa and the swimming pool or spa is closed;
2. The swimming pool or spa is closed for at least 24 hours;
3. As much of the liquid feces as possible in the swimming pool or spa is removed and disposed of in a toilet;
4. The swimming pool or spa is chemically treated with a shock treatment;
5. The water in the swimming pool or spa is tested 24 hours after applying the shock treatment to determine whether the water complies with the water quality and disinfection standards in R9-8-803; and
6. The swimming pool or spa is not reopened until a test conducted under subsection (B)(5) indicates that the water complies with the water quality and disinfection standards in R9-8-803.

Historical Note

New Section made by final rulemaking at 8 A.A.R. 3645, effective August 9, 2002 (Supp. 02-3).

R9-8-811. Natural and Semi-artificial Bathing Place and Artificial Lake Water Quality Standards

An operator of a public or semipublic natural bathing place, a semi-artificial bathing place, or an artificial lake shall ensure that the public or semipublic natural bathing place, semi-artificial bathing place, or artificial lake meets the narrative and numeric water quality standards in 18 A.A.C. 11, Article 1 when the public or semipublic natural bathing place, semi-artificial bathing place, or artificial lake is open for water contact recreation.

Historical Note

Section repealed; new Section made by final rulemaking at 8 A.A.R. 3645, effective August 9, 2002 (Supp. 02-3).

R9-8-812. Inspections

A. A regulatory authority shall inspect a bathing place to determine whether the bathing place complies with this Article.

B. A regulatory authority shall inspect a public swimming pool at least once each month that the swimming pool is open for water contact recreation.

Historical Note

Section repealed; new Section made by final rulemaking at 8 A.A.R. 3645, effective August 9, 2002 (Supp. 02-3).

R9-8-813. Cease and Desist and Abatement

A. Engaging in any practice in violation of this Article is a public nuisance.

B. If a regulatory authority has reasonable cause to believe that an operator of a public or semipublic swimming pool or bathing place is creating or maintaining a public nuisance at the public or semipublic swimming pool or bathing place, the regulatory authority shall order the operator to discontinue the activity and to abate the public nuisance as follows:

1. The regulatory authority shall serve on the operator a written cease and desist and abatement order requiring the operator to discontinue the activity and to remove the public nuisance at the operator's expense within 24 hours after service of the order. The order shall contain:
   a. A reference to the statute or rule that is alleged to have been violated or on which the order is based,
   b. A description of the operator's right to request a hearing, and
   c. A description of the operator's right to request an informal settlement conference.
2. The regulatory authority shall serve the order and any subsequent notices by personal delivery or certified mail, return receipt requested, to the operator or other party's last address of record with the regulatory authority or by any other method reasonably calculated to effect actual notice to the operator or other party.
3. The operator or another party whose rights are determined by the order may obtain a hearing to appeal the order by filing a written notice of appeal with the regulatory authority within 30 days after service of the order. The operator or other party appealing the order shall serve the notice of appeal upon the regulatory authority by personal delivery or certified mail, return receipt requested, to the office of the regulatory authority or by any other method reasonably calculated to effect actual notice on the regulatory authority. Appealing an order does not release the operator from the obligation to comply with the order.
4. If a notice of appeal is timely filed, the regulatory authority shall do one of the following:
   a. If the regulatory authority is the Department or a local health department or public health services district to which the duty to comply with A.R.S. Title 41, Chapter 6, Article 10 is delegated, the notification and hearing shall comply with A.R.S. Title 41, Chapter 6, Article 10 and any rules promulgated by the Office of Administrative Hearings.
   b. For all other regulatory authorities, the notification and hearing shall comply with the procedures adopted by a county board of supervisors as required by A.R.S. § 36-183.04(E).
5. If a written notice of appeal is not timely filed, the order becomes final.
6. A regulatory authority shall inspect the public or semipublic swimming pool or bathing place 24 hours after service of the order to determine whether the operator has complied with the order. If the regulatory authority determines upon inspection that the operator has not ceased the activity and abated the public nuisance, the regulatory authority shall cause the public nuisance to be removed.

Historical Note

Section repealed; new Section made by final rulemaking at 8 A.A.R. 3645, effective August 9, 2002 (Supp. 02-3).

R9-8-814. Repealed

Historical Note

Section repealed by final rulemaking at 8 A.A.R. 3645, effective August 9, 2002 (Supp. 02-3).

R9-8-815. Repealed

Historical Note

Section repealed by final rulemaking at 8 A.A.R. 3645, effective August 9, 2002 (Supp. 02-3).

R9-8-816. Repealed

Historical Note

Section repealed by final rulemaking at 8 A.A.R. 3645, effective August 9, 2002 (Supp. 02-3).

R9-8-817. Repealed

Historical Note

Section repealed by final rulemaking at 8 A.A.R. 3645, effective August 9, 2002 (Supp. 02-3).

R9-8-818. Reserved

R9-8-819. Reserved

R9-8-820. Reserved

R9-8-821. Repealed

Historical note

R9-8-821 repealed by summary action with an interim effective date of July 6, 1998; filed in the Office of the Secretary of State June 8, 1998 (Supp. 98-2). Adopted summary rules filed October 9, 1998; interim effective date of July 6, 1998, now the permanent effective date (Supp. 98-4).

R9-8-822. Repealed

Historical note

R9-8-822 repealed by summary action with an interim effective date of July 6, 1998; filed in the Office of the Secretary of State June 8, 1998 (Supp. 98-2). Adopted summary rules filed October 9, 1998; interim effective date of July 6, 1998, now the permanent effective date (Supp. 98-4).

R9-8-823. Repealed

Historical Note

Section repealed by final rulemaking at 8 A.A.R. 3645, effective August 9, 2002 (Supp. 02-3).

R9-8-824. Repealed

Historical Note

Section repealed by final rulemaking at 8 A.A.R. 3645, effective August 9, 2002 (Supp. 02-3).

R9-8-825. Reserved

R9-8-826. Reserved

R9-8-827. Reserved

R9-8-828. Reserved

R9-8-829. Reserved

Chlorine Dioxide	9

*The presence of one or both of these devices earns five points for the facility.

2. No points are added for Grade 1 small systems that:
   a. Only distribute groundwater;
   b. Serve fewer than 501 persons;
   c. Have no disinfection or disinfect by chlorine gas or hypochlorite only; and
   d. Do not store water or store water only in storage tanks.
3. The Department shall assign a grade by the total number of points assigned to the facility, as follows:

Grade	Point Range
Grade 1	0
Grade 2	1 to 20
Grade 3	21 to 35
Grade 4	More than 35

Historical Note

Former Section R9-20-520 repealed, new Section R9-20-520 adopted effective November 1, 1979 (Supp. 79-6). Former Section R9-20-520 amended, renumbered as Section R9-20-515, then renumbered as Section R18-4-115 effective October 23, 1987 (Supp. 87-4). R18-5-115 recodified from R18-4-115 (Supp. 95-2). Amended by final rulemaking at 7 A.A.R. 1171, effective February 16, 2001 (Supp. 01-1). Amended by final rulemaking at 7 A.A.R. 5079, effective October 16, 2001 (Supp. 01-4). Amended by final rulemaking at 11 A.A.R. 998, effective April 2, 2005 (Supp. 05-1).

R18-5-116. Initial Grading and Regrading of Facilities

A. The Department shall act under A.R.S. Title 41, Chapter 6, Article 10 and 18 A.A.C. 1, Article 2 when initially grading or when regrading a facility.

B. If it is determining the initial grade of a facility or whether to regrade a facility, the Department shall consider the facility characteristics in R18-5-114 and R18-5-115, and whether:
1. The facility has special design features or characteristics that make it unusually difficult to operate;
2. The water or wastewater is unusually difficult to treat;
3. The facility uses effluent; or
4. The facility poses a potential risk to public health, safety or welfare.

C. The owner of a facility that is regraded under this Article shall ensure that the facility is operated by an operator, in compliance with this Article, no later than one year from the effective date of the facility regrading.

Historical Note

New Section adopted by final rulemaking at 7 A.A.R. 1171, effective February 16, 2001 (Supp. 01-1).

ARTICLE 2. PUBLIC AND SEMIPUBLIC SWIMMING POOLS AND SPAS

R18-5-201. Definitions

"Air induction system" means a system whereby a volume of air is induced into a hollow ducting in a spa floor, bench, or wall. An air induction system is activated by an air power blower and is separate from the water circulation system.

"Artificial lake" means a man-made lake, lagoon, or basin, lined or unlined, with a surface area equal to or greater than two acres (87,120 square feet), that is used or intended to be used for water contact recreation.

"Backwash" means the process of thoroughly cleaning a filter by the reverse flow of water through the filter.

"Barrier" means a fence, wall, building, or landscaping that obstructs access to a public or semipublic swimming pool or spa.

"Cartridge filter" means a depth, pleated, or surface-type filter component with fixed dimensions that is designed to remove suspended particles from water flowing through the filter.

"Construct" means to build or install a new public or semipublic swimming pool or spa or to enlarge, deepen, or make a major modification to an existing public or semipublic swimming pool or spa.

"Coping" means the cap on a swimming pool or spa wall that provides a finished edge around the swimming pool or spa.

"Cross-connection" means any physical connection or structural arrangement between a potable water system and the piping system for a public or semipublic swimming pool or spa through which it is possible to introduce used water, gas, or any other substance into the potable water system. A bypass arrangement, jumper connection, removable section, swivel or change-over device, or any other temporary or permanent device that may cause backflow is a cross-connection.

"Deck" means a hard surface area immediately adjacent or attached to a swimming pool or spa that is designed for sitting, standing, or walking.

"Deep area" means the portion of a public or semipublic swimming pool that is more than 5 feet in depth.

"Discharge piping" means the portion of the circulation system that carries water from the filter back to the swimming pool or spa.

"Diving area" means the area of a public or semipublic swimming pool that is designated for diving from a diving board, diving platform, or starting block.

"Fill-and-draw swimming pool or spa" means a swimming pool or spa where the principal means of cleaning is the complete removal of the used water and its replacement with potable water.

"Filtration rate" means the rate of water flowing through a filter during the filter cycle expressed in gallons per minute per square foot of effective filter area.

"Flow-through swimming pool or spa" means a swimming pool or spa where new water enters the swimming pool or spa to replace an equal quantity of water that constantly flows out.

"Freeboard" means the vertical wall section of a swimming pool or spa wall between the waterline and the deck.

"Hose bibb" means a faucet with a threaded nozzle to which a hose may be attached.

"Hydrotherapy jet" means a fitting that blends air and water and creates a high-velocity, turbulent stream of air-enriched water for injection into a spa.

"Make-up water" means fresh water used to fill or refill a swimming pool or spa.

"Maximum bathing load" means the design capacity or the maximum number of users that a public or semipublic swimming pool or spa is designed to hold.

"Natural bathing place" means a lake, pond, river, stream, swimming hole, or hot springs which has not been modified by man.

"Operate" means to run, maintain, or otherwise control or direct the functioning of a public or semipublic swimming pool or spa.

"Overflow collection system" means equipment designed to remove water from a swimming pool or spa, including gutters, overflows, surface skimmers, and other surface water collection systems of various designs and manufacture.

"Potable water" means drinking water.

"Private residential spa" means a spa at a private residence used only by the owner, members of the owner's family, and invited guests, or a spa that serves a housing group consisting of no more than three living units [for example, duplexes or triplexes].

"Private residential swimming pool" means a swimming pool at a private residence used only by the owner, members of the owner's family, and invited guests, or a swimming pool that serves a housing group consisting of no more than three living units [for example, duplexes or triplexes].

"Public spa" means a spa that is open to the public with or without a fee, including a spa that is operated by a county, municipality, political subdivision, school district, university, college, or a commercial establishment whose primary business is the operation of a spa.

"Public swimming pool" means a swimming pool that is open to the public with or without a fee, including a swimming pool that is operated by a county, municipality, political subdivision, school district, university, college, or a commercial establishment whose primary business is the operation of a swimming pool.

"Recessed treads" means a series of vertically spaced, preformed stepholes in a swimming pool wall.

"Return inlet" means an aperture or fitting through which filtered water returns to a swimming pool or spa.

"Rope and float line" means a continuous line not less than 3/4 inch in diameter that is supported by buoys and attached to opposite sides of a swimming pool to separate areas of the swimming pool.

"Semi-artificial bathing place" means a natural bathing place that has been modified by man.

"Semipublic spa" means a spa operated for the residents of lodgings such as hotels, motels, resorts, apartments, condominiums, townhouse complexes, trailer courts, mobile home parks, or similar establishments. A semipublic spa includes a spa that is operated by a neighborhood or community association for the residents of the community and their guests and any spa at a country club, health club, camp, or similar establishment where the primary business of the establishment is not the operation of a spa and where the use of the spa is included in the fee for the primary use of the establishment.

"Semipublic swimming pool" means a swimming pool operated for the residents of lodgings such as hotels, motels, resorts, apartments, condominiums, townhouse complexes, trailer courts, mobile home parks, or similar establishments. A semipublic swimming pool includes a swimming pool that is operated by a neighborhood or community association for the residents of the community and their guests and a swimming pool at a country club, health club, camp, or similar establishment where the primary business of the establishment is not the operation of a swimming pool and where the use of the swimming pool is included in the fee for the primary use of the establishment.

"Shallow area" means the portion of a public or semipublic swimming pool that is 5 feet or less in depth.

"Slip-resistant" means a surface that has a static coefficient of friction [wet or dry] of at least 0.50.

"Spa" means an artificial basin, chamber, or tank of irregular or geometric shell design that is intended only for bathing or soaking and that is not drained, cleaned, or refilled for each user. A spa may include features such as hydrotherapy jet circulation, hot water, cold water mineral baths, or an air induction system. Industry terminology for a spa includes "hydrotherapy pool," "whirlpool," "hot tub," and "therapy pool."

"Special use pool" means a swimming pool intended for competitive aquatic events, aquatic exercise, or lap swimming. A special use pool includes a wave action pool, exit pool for a water slide, swimming pool that is part of an attraction at a water recreation park, water volleyball pool, or a swimming pool with special features used for training and instruction.

"Suction outlet" means the aperture or fitting through which water is withdrawn from a swimming pool or spa.

"Suction piping" means the water circulation system piping that carries water from a swimming pool or spa to the filter.

"Swimming pool" means an artificial basin, chamber, or tank that is designed for swimming or diving.

"Turnover rate" means the number of hours required to circulate a volume of water equal to the capacity of the swimming pool or spa.

"User" means a person who uses a swimming pool, spa, or adjoining deck area.

"Wading pool" means a shallow swimming pool used for bathing and wading by small children.

"Water circulation system" means an arrangement of mechanical equipment connected to a swimming pool or spa by piping in a closed loop that directs water from the swimming pool or spa to the filtration and disinfection equipment and returns the water to the swimming pool or spa.

"Water circulation system components" means the mechanical components that are part of a water circulation system of a swimming pool or spa, including pumps, filters, valves, surface skimmers, ion generators, electrolytic chlorine generators, ozone process equipment, and chemical feeding equipment.

"Water level" means either:

a. On swimming pools and spas with skimmer systems, the midpoint of the operating range of the skimmers, or
b. On swimming pools and spas with overflow gutters, the height of the overflow rim of the gutter.

Historical Note

Adopted effective February 19, 1998 (Supp. 98-1).

R18-5-202. Applicability

A. This Article applies to public and semipublic swimming pools and spas.

B. This Article does not apply to the following:
1. A private residential swimming pool or spa,
2. A swimming pool or spa used for medical treatment or physical therapy and supervised by licensed medical personnel,
3. A semi-artificial bathing place,
4. A natural bathing place, or
5. An artificial lake.

Historical Note

Adopted effective February 19, 1998 (Supp. 98-1).

R18-5-203. Design Approval

A. A person shall obtain design approval from the Department before starting construction of:
1. A new public or semipublic swimming pool or spa;
2. A major modification to an existing public or semipublic swimming pool or spa. For purposes of this subsection, a major modification means a change to the shape, depth, water circulation system, or disinfection system of a public or semipublic swimming pool or spa or the installation of diving equipment at a public or semipublic swimming pool;
3. A change in use from a semipublic swimming pool to a public swimming pool; and
4. A change in use from a private residential swimming pool to a public or semipublic swimming pool.

B. An applicant for a design approval shall submit an ADEQ application form to the Department in quadruplicate with four complete sets of plans and specifications for the swimming pool or spa and the information in subsection (C).

C. The application for design approval shall include four copies of the following:
1. A general plot plan;
2. Plans and specifications showing the size, shape, cross-section, slope, and dimensions of each swimming pool or spa, deck areas, and barriers;
3. Plans and specifications showing the water circulation and disinfection systems, including all piping, fittings, drains, suction outlets, filters, pumps, surface skimmers, return inlets, chemical feeders, disinfection equipment, gauges, flow meters, and strainers;
4. Plans and specifications showing the source of water supply and the method of disposal of filter backwash water; used swimming pool or spa water, and wastewater from toilets, urinals, sinks, and showers;
5. Detailed plans of bathhouses, dressing rooms, equipment rooms, and other appurtenances; and
6. Additional data required by the Department for a complete understanding of the project.

D. A professional engineer, architect, or a swimming pool or spa contractor with a current A-9, A-19, KA-5, KA-6 license shall prepare or supervise the preparation of all plans and specifications submitted to the Department for review.

E. An applicant shall submit an application for design approval to the Department at least 60 days prior to the date that the applicant wishes to begin construction of a swimming pool or spa.

F. The Department shall determine whether the application for design approval is complete within 30 days of the date of receipt of the application by the Department.

G. The Department shall issue or deny the application for design approval within 30 days of the date that the Department determines that the application for design approval is complete.

H. Unless an extension of time is granted in writing by the Department, a design approval is void if construction is not started within one year after the date of its issuance or there is a halt in construction of more than one year.

I. The Department may issue a design approval with conditions. The Department shall not issue an Approval of Construction if the design approval is conditioned and the construction of the swimming pool or spa does not comply with the stated conditions.

J. The Department may issue design approvals in phases to allow a political subdivision to start construction of a public swimming pool or spa without issuing a design approval for the entire construction project. A design approval may be issued in phases provided all of the following conditions are met:
1. A phased design approval is needed to accommodate a design/build contract, phased construction contract, multiple construction contracts, turnkey contract, or special contract that requires construction to begin prior to the completion of design plans and specifications for the entire public swimming pool or spa construction project.
2. The applicant submits a detailed project description for the entire public swimming pool or spa construction project to the Department.
3. There is a written agreement between the applicant and the Department which includes the following:
   a. A construction project schedule,
   b. A schedule to submit applications and supporting documentation for the phased design approval including any anticipated variance requests,
   c. Negotiated time-frames for administrative completeness and substantive review of each application for phased design approval, and
   d. A schedule of construction inspections by the Department or third-party certifications by the applicant.
4. The applicant certifies in writing that the applicant understands that the public swimming pool or spa cannot be operated without an Approval of Construction for each phase of the construction project pursuant to R18-5-204.
5. If the applicant and the Department cannot reach agreement regarding a phased design approval or Approval of Construction, then the requirements of R18-5-203(A) through (I) and R18-5-204 apply.

Historical Note

Adopted effective February 19, 1998 (Supp. 98-1).

R18-5-204. Approval of Construction

A. A public or semipublic swimming pool or spa shall not operate without receiving an Approval of Construction issued by the Department.

B. The construction of a public or semipublic swimming pool or spa shall conform to plans and specifications that have been approved by the Department. If the applicant wishes to make a change to the approved plans and specifications, the applicant shall submit revised plans and specifications with a written statement of the reasons for the change to the Department. The applicant shall obtain Department approval of the revised plans and specifications before starting any work affected by the change.

C. Prior to any construction that will cover the piping arrangement of the swimming pool or spa and at least 30 days prior to the expected date of completion of construction of a public swimming pool or spa, the applicant shall notify the Department to permit a construction inspection. The Department shall inspect the construction of a swimming pool or spa to determine if the swimming pool or spa has been constructed in

accordance with Department-approved plans, specifications, and conditions unless a professional engineer, architect, or registered sanitarian certifies that the swimming pool or spa has been constructed in accordance with Department-approved plans, specifications, and conditions.

D. If the swimming pool or spa has been constructed in accordance with Department-approved plans, specifications, and conditions, the Department shall issue the Approval of Construction within 30 days of the date of the construction inspection by the Department or the date the Department receives third-party certification.

Historical Note

Adopted effective February 19, 1998 (Supp. 98-1).

R18-5-205. Prohibitions

A. A fill-and-draw swimming pool or spa shall not be used as a public or semipublic swimming pool or spa.

B. A private residential spa shall not be used as a public or semipublic spa.

Historical Note

Adopted effective February 19, 1998 (Supp. 98-1).

R18-5-206. Water Source

Only water from a source that is approved by the Department shall be used in a public or semipublic swimming pool or spa. Reclaimed wastewater shall not be used as make-up water for a public or semipublic swimming pool or spa.

Historical Note

Adopted effective February 19, 1998 (Supp. 98-1).

R18-5-207. Construction Materials

A. A public or semipublic swimming pool or spa shall be constructed of concrete or other structurally rigid material that is equivalent in strength or durability to concrete, except that a public or semipublic spa may be constructed of fiberglass or acrylic.

B. A public or semipublic swimming pool or spa shall be constructed of materials that are nontoxic.

C. A public or semipublic swimming pool or spa shall be constructed of waterproof materials that provide a watertight structure.

D. A public or semipublic swimming pool or spa shall have a smooth and easily cleaned surface, without cracks or joints, excluding structural joints, or to which a smooth, easily cleaned surface finish is applied or attached.

E. All corners in a public or semipublic swimming pool or spa shall be rounded, including the corners formed by the intersection of a wall and floor.

F. A surface within a public or semipublic swimming pool or spa intended to provide footing for users shall have a slip-resistant surface. The roughness or irregularity of the surface shall not cause injury or discomfort to users' feet during normal use.

G. The color, pattern, or finish of the interior of a public or semipublic swimming pool or spa shall not obscure objects, surfaces within the swimming pool or spa, debris, sediment, or algae. Surface finishes shall be white, pastel, or other light color. The interior finish shall completely line the swimming pool or spa to the coping, tile, or gutter system.

Historical Note

Adopted effective February 19, 1998 (Supp. 98-1).

R18-5-208. Maximum Bathing Load

A. The maximum bathing load for a public or semipublic swimming pool or spa shall not be exceeded.

B. The maximum bathing load for a public or semipublic swimming pool shall be calculated as the sum of the following:

1. The shallow area of the swimming pool in square feet divided by 10 square feet, plus
2. The deep area of the swimming pool in square feet minus 300 square feet for each diving board divided by 24 square feet.

C. The maximum bathing load for a public swimming pool shall be limited by the number of users for the toilets, showers, or lavatories that are provided in the bathhouses or dressing rooms prescribed in R18-5-242.

D. The maximum bathing load for a public or semipublic spa shall not exceed the area of the spa in square feet divided by 9 square feet.

E. The maximum bathing load for a public or semipublic swimming pool or spa shall be posted.

Historical Note

Adopted effective February 19, 1998 (Supp. 98-1).

R18-5-209. Shape

A. A public or semipublic swimming pool or spa may be any shape except that the designer shall shape a public or semipublic swimming pool or spa to minimize hazards to users and provide adequate circulation of swimming pool or spa water.

B. There shall be no protrusions, extensions, means of entanglement, or other obstructions in a public or semipublic swimming pool or spa that may cause entrapment of or injury to the user. This subsection does not prohibit water features such as water fountains, slides, water play equipment, or water volleyball and basketball nets.

Historical Note

Adopted effective February 19, 1998 (Supp. 98-1).

R18-5-210. Walls

A. Where a racing lane terminates in a swimming pool, the wall shall be plumb to a minimum depth of 5 feet below the waterline. Below the 5-foot depth, the wall shall be radiused to join the floor.

B. There shall be no projections from a swimming pool or spa wall except for coping, cantilevered deck, ladders, and steps.

C. An underwater seat shall comply with the following:

1. The edges of an underwater seat shall be outlined with a sharply contrasting colored tile or other material that is clearly visible from the deck adjacent to the underwater seat;
2. An underwater seat shall have a slip-resistant surface;
3. An underwater seat shall be located outside of the deep area of a swimming pool that is equipped for diving. An underwater seat may be located in the deep area of a swimming pool that is not equipped for diving provided the underwater seat is either completely recessed into the swimming pool wall, shaped to be compatible with the shape of the swimming pool wall, or in a corner of the swimming pool;
4. The maximum depth of an underwater seat is 24 inches below the waterline. The minimum depth of an underwater seat is 12 inches below the waterline; and
5. The maximum width of an underwater seat is 20 inches.

D. If a spa is located immediately adjacent to a swimming pool, the separating wall between the spa and the swimming pool shall be no more than 8 inches wide. The top of the separating wall shall be no lower than the level of the coping of the swimming pool. If a separating wall is more than 8 inches wide, then the deck width shall comply with R18-5-217(D). A spa

shall not be located immediately adjacent to the deep area of a swimming pool.

E. Coping or cantilevered deck may project from a swimming pool or spa wall to provide a handhold for users. The coping or deck shall be rounded, have a slip-resistant surface finish, and shall not exceed 3 1/2 inches in thickness. The overhang of the coping or deck shall not exceed 2 inches or be less than 1 inch. All corners created by coping or cantilevered deck shall be rounded in both the vertical and horizontal dimensions to eliminate sharp corners.

Historical Note

Adopted effective February 19, 1998 (Supp. 98-1).

R18-5-211. Freeboard

A. The freeboard in a public or semipublic swimming pool or spa shall not exceed 8 inches, except as provided in subsection (B).

B. The freeboard in a semipublic swimming pool may exceed 8 inches to provide for walls, terraces, or other design features. The Department shall review each request to allow an increase in freeboard on a case-by-case basis. In reviewing the request, the Department shall consider safety, exit distances, alternative exits, and location. The length and height of the section where the freeboard area may be increased is limited. All of the following requirements shall be met:
1. Guard rails or similar devices are provided to prevent any raised area from being used as a diving platform.
2. The vertical surfaces of the freeboard area are constructed of inorganic materials. All vertical surfaces shall be rigid, smooth, and easily cleanable.
3. The horizontal surface areas comply with the provisions of this Article for decks.
4. The vertical surface area is included as surface area of the swimming pool to determine the type, size, location, and numbers of equipment and piping.

Historical Note

Adopted effective February 19, 1998 (Supp. 98-1).

R18-5-212. Floors

A. The slope of the floor of a public or semipublic swimming pool, from the end wall in the shallow area towards the deep area to the point of the first slope change shall be uniform and shall not exceed 1 foot of fall in 10 feet. The floor slope in a public or semipublic spa shall not exceed 1 foot of fall in 10 feet.

B. The floor slope of a public or semipublic swimming pool, from the point of the first slope change to the deepest part of the swimming pool, shall not exceed 1 foot of fall in 3 feet.

C. For a public or semipublic swimming pool that is equipped for diving, the depth of the swimming pool at the point of the first slope change shall be a minimum of 5 feet. For a public or semipublic swimming pool that is not equipped for diving, the depth of the swimming pool at the point of the first slope change shall be a minimum of 4 feet.

D. All portions of a swimming pool or spa floor shall slope towards a main drain.

E. The transitional radius where the floor of a public or semipublic swimming pool joins a wall shall comply with the following:
1. The center of the radius shall be no less than 3 feet below the waterline in the deep area or 2 feet below the waterline in the shallow area.
2. The radius shall be tangent at the point where the radius meets the wall or floor.
3. The radius shall be equal to or greater than the depth of the swimming pool minus the vertical wall depth measured from the waterline minus 3 inches.

Historical Note

Adopted effective February 19, 1998 (Supp. 98-1).

R18-5-213. Entries and Exits

A. Each public or semipublic swimming pool shall have at least two means of entry or exit consisting of ladders, steps, or recessed treads.

B. There shall be at least one ladder, set of steps, or set of recessed treads for each 75 feet of perimeter of a public or semipublic swimming pool or spa.

C. At least one means of entry and exit shall be provided in the deep area and at least one means of entry and exit shall be provided in the shallow area of a public or semipublic swimming pool. Where the water depth is 2 feet at the swimming pool wall in the shallow area or where there is a zero depth entry pool [for example, an artificial beach], the area shall be considered a means of entry or exit.

D. A set of steps shall be provided in a public or semipublic spa.

E. The location of stairs, ladders, and recessed treads shall not interfere with racing lanes.

Historical Note

Adopted effective February 19, 1998 (Supp. 98-1).

R18-5-214. Steps

A. Each set of steps shall be provided with at least one handrail to serve all treads and risers. Handrails shall be provided at one side or in the center of all steps. Handrails shall be installed in such a way that they can be removed only with tools.

B. Steps shall be permanently marked to be clearly visible from above and below the water level in a swimming pool or spa. The edges of steps shall be outlined with a sharply contrasting colored tile or other material that is clearly visible from the deck adjacent to the steps.

C. Steps may be constructed only in the shallow area of a public or semipublic swimming pool.

D. Steps shall not project into a public or semipublic swimming pool or spa in a manner that creates a hazard to users.

E. All tread surfaces on steps shall have slip-resistant surfaces.

F. Step treads shall have a minimum unobstructed horizontal depth of 10 inches. Risers shall have a maximum uniform height of 12 inches, with the bottom riser height allowed to vary ± 2 inches from the uniform riser height.

Historical Note

Adopted effective February 19, 1998 (Supp. 98-1).

R18-5-215. Ladders

A. At least one ladder shall be provided in the deep area of a public or semipublic swimming pool. If the width of the deep area of a swimming pool is greater than 20 feet, then one ladders shall be located on opposite sides of the deep area.

B. A swimming pool or spa ladder shall be equipped with two handrails.

C. All treads on ladders shall have slip-resistant surfaces.

D. Ladder treads shall have a minimum horizontal depth of 1 1/2 inches. The distance between ladder treads shall range from a minimum of 7 inches to a maximum of 12 inches.

E. Below the waterline, there shall be a clearance of not more than 6 inches and not less than 3 inches between any ladder tread edge and the wall as measured from the side of the tread closest to the wall.

Historical Note

Adopted effective February 19, 1998 (Supp. 98-1).

R18-5-216. Recessed Treads

A. Recessed treads with handrails may be substituted for ladders.
B. Recessed treads shall be pre-formed, readily cleanable, and designed to drain into the swimming pool or spa to prevent the accumulation of dirt in the recessed treads.
C. Each set of recessed treads shall be equipped with two handrails.
D. All recessed treads shall have slip-resistant surfaces.
E. The vertical distance between the swimming pool or spa coping edge or deck and the uppermost recessed tread shall be a maximum of 12 inches. Recessed treads at the centerline shall have a uniform vertical spacing of 12 inches maximum and 7 inches minimum.
F. Recessed treads shall be at least 5 inches deep and 12 inches wide.

Historical Note

Adopted effective February 19, 1998 (Supp. 98-1).

R18-5-217. Decks and Deck Equipment

A. Decks, ramps, coping, and similar step surfaces shall be constructed of concrete or other inorganic material, have a slip-resistant finish, and be easily cleanable.
B. The minimum continuous unobstructed deck width, including the coping, shall be 10 feet for a public swimming pool and 4 feet for a semipublic swimming pool. The dimensional design of decks at public and semipublic swimming pools shall comply with the dimensions shown in Illustration B.
C. A minimum 5 feet of deck width shall be provided on the sides and rear of any diving equipment at a public swimming pool. A minimum 4 feet of deck width shall be provided on the sides and rear of any diving equipment at a semipublic swimming pool. If diving equipment is installed at a public swimming pool, there shall be a minimum 15 feet of deck width from the swimming pool wall to the edge of the deck behind the diving equipment [See Illustration B].
D. A continuous unobstructed deck width of at least 4 feet, which may include the coping, shall be provided on at least two contiguous sides and around at least 50% of the perimeter of a public or semipublic spa.
E. Decks shall be sloped to effectively drain either to perimeter areas or to deck drains. Drainage shall remove splash water, deck cleaning water, and rain water without leaving standing water. The minimum slope of the deck shall be 1/4 inch per 1 foot. The maximum slope of the deck shall be 1 inch per 1 foot, except for ramps.
F. Decks shall be edged to eliminate sharp corners.
G. Site drainage shall be provided to direct all perimeter deck drainage and general site and roof drainage away from a public or semipublic swimming pool or spa. Yard drains may be required to prevent the accumulation or puddling of water in the general area of the deck and related improvements.
H. Hose bibbs shall be provided along the perimeter of the deck so that all parts of the deck may be washed down. At a minimum, each hose bibb shall be protected against back siphonage with an atmospheric vacuum breaker. The Department may approve quick disconnect style hose bibbs.
I. Any valve that is installed in or under any deck shall provide a minimum 10-inch diameter access cover and a valve pit to facilitate the repair and maintenance of the valve.
J. Joints in decks shall be provided to minimize the potential for cracks due to changes in elevations or movement of the slab. The maximum voids between adjoining concrete slabs or between concrete slabs and expansion joint material shall be 3/16 inch of horizontal clearance with a maximum difference in vertical elevation of 1/4 inch. Areas where the deck joins concrete shall be protected by expansion joints to protect the swimming pool or spa from the pressures of relative movements. Construction joints where pool or spa coping meets the deck shall be watertight and shall not allow water to pass through to the underlying ground.

Historical Note

Adopted effective February 19, 1998 (Supp. 98-1).

R18-5-218. Lighting

A. A public or semipublic swimming pool or spa and adjacent deck areas shall be lighted by natural or artificial means when they are in use.
B. A public or semipublic swimming pool or spa that is intended to be used at night shall be equipped with artificial lighting that is designed and spaced so that all parts of the swimming pool or spa, including the bottom, may be seen without glare.

Historical Note

Adopted effective February 19, 1998 (Supp. 98-1).

R18-5-219. Water Depths

A. Except as provided in subsection (B), the minimum water depth in the shallowest area of a public or semipublic swimming pool shall be 2 feet. The maximum water depth in the shallowest area of a public or semipublic swimming pool shall be 3 feet. In public swimming pools, where racing lanes terminate, the minimum depth shall be 5 feet from the water level to the point where the vertical wall is radiused to join the floor.
B. The Department may approve a depth of less than 2 feet in a wading pool or to allow a zero depth entry swimming pool.
C. The maximum water depth in a public or semipublic spa shall be 42 inches, measured from the water level.

Historical Note

Adopted effective February 19, 1998 (Supp. 98-1).

R18-5-220. Depth Markers

A. Water depths shall be conspicuously and permanently marked at or above the water level on the vertical wall and on the top of the coping or the edge of the deck next to a swimming pool.
  1. Depth markers on a vertical wall shall be positioned to be read from the water side.
  2. Depth markers on a deck shall be located within 18 inches of the side of the swimming pool and positioned to be read while standing on the deck facing the water. Depth markers that are located on a deck shall be made of slip-resistant materials.

B. Depth markers for a public or semipublic swimming pool shall be installed at points of maximum and minimum water depth and at all points of slope change. Depth markers are required in the shallow area at 1-foot depth intervals to a depth of 5 feet. Thereafter, depth markers shall be installed at 2-foot depth intervals. Depth markers shall not be spaced at distances greater than 25 feet.
C. Depth markers shall be located on both sides and at both ends of a public or semipublic swimming pool.
D. Depth markers shall be in Arabic numerals with a 4-inch minimum height. Arabic numerals shall be of contrasting color to the background.
E. In public swimming pools with racing lanes, approach warning markers shall be placed below the water level on the opposite walls at the ends of each racing lane. Warning markers shall be of contrasting color to the background. Warning markers shall be clearly visible in or out of the water from a minimum distance of 10 feet.

F. The shallow area of a public swimming pool shall be visually set apart from the deep area of the pool by a rope and float line.

G. Depth markers for a public or semipublic spa shall comply with all of the following:
1. A public or semipublic spa shall have permanent depth markers with numbers that are a minimum of 4 inches high. Depth markers shall be plainly and conspicuously visible from all points of entry.
2. The maximum depth of a public or semipublic spa shall be clearly indicated by depth markers.
3. There shall be a minimum of 2 depth markers at each public or semipublic spa.
4. Depth markers shall be spaced at no more than 25-foot intervals and shall be uniformly located around the perimeter of the spa.
5. Depth markers shall be positioned on the deck within 18 inches of the side of the spa. A depth marker shall be positioned so that it can be read by a person standing on the deck facing the water.
6. Depth markers that are on deck surfaces shall be made of slip-resistant material.

Historical Note

Adopted effective February 19, 1998 (Supp. 98-1).

R18-5-221. Diving Areas and Equipment

A. The dimensions of a diving area in a public or semipublic swimming pool shall comply with minimum requirements for length, width, depth, area, and other dimensions specified in Illustration A. The diving well profile in Illustration A does not apply to a special use pool that is intended for competitive diving and has been approved by Department pursuant to R18-5-248(A).

B. Diving equipment shall be permanently anchored to the swimming pool deck. Equipment shall be rigidly constructed with sufficient bracing to ensure stability. Supports, platforms, steps, and ladders for diving equipment shall be designed to carry anticipated loads.

C. All diving stands higher than 21 inches, measured from the deck to the top of the board, shall be provided with stairs or a ladder.

D. Diving equipment shall have a durable finish. The surface finish shall be free of tears, splinters, or cracks that may be a hazard to users.

E. Steps and ladders leading to diving boards and diving platforms shall be of corrosion-resisting materials and shall have slip-resistant tread surfaces. Step treads shall be self-draining.

F. Diving boards, diving platforms, and starting blocks shall have slip-resistant tread surfaces.

G. Handrails shall be provided at all steps and ladders leading to diving boards that are 1 meter or more above the water.

H. Diving boards and diving platforms that are 1 meter or higher shall be protected with guard rails. Guard rails shall be at least 30 inches above the diving board or diving platform and shall extend to the edge of the swimming pool wall.

I. A label shall be permanently affixed to a diving board and shall include the following:
1. Manufacturer's name and address,
2. Board length, and
3. Fulcrum setting instructions.

J. The maximum diving board height over the water is 3 meters. The maximum height of a diving platform over the water is 10 meters.

K. Starting blocks shall be located in the deep end of a public swimming pool or where the depth of the water is at least 5 feet.

L. There shall be a completely unobstructed clear vertical distance of 13 feet above any diving board measured from the center of the front end of the board. This clear, unobstructed vertical space shall extend horizontally at least 8 feet behind, 8 feet to each side, and 16 feet ahead of the front end of the board.

Historical Note

Adopted effective February 19, 1998 (Supp. 98-1).

R18-5-222. Prohibition Against Diving; Warning Signs

A. Diving equipment is prohibited in a public or semipublic swimming pool that does not meet the minimum diving well dimensions specified in Illustration A. If a public or semipublic swimming pool does not meet the dimensional requirements prescribed in Illustration A for diving, then the owner shall prominently display at least one sign that cautions users that the swimming pool is not suitable for diving. The warning sign shall state "NO DIVING" in letters that are 4 inches or larger or display the international symbol for no diving.

B. Diving from the deck of a public or semipublic swimming pool into water that is less than 5 feet deep shall be prohibited. Warning markers indicating in words or symbols that diving is prohibited shall be placed on the deck within 18 inches of the side of the shallow area of the swimming pool. A warning marker shall be positioned so that it can be read by a person standing on the deck facing the water.

Historical Note

Adopted effective February 19, 1998 (Supp. 98-1).

R18-5-223. Water Circulation System

A. A public or semipublic swimming pool or spa shall have a water circulation system that provides complete circulation of water through all parts of the swimming pool or spa and can maintain water chemistry and water clarity requirements.

B. The water circulation system for a public or semipublic swimming pool shall have a turnover rate of at least once every 8 hours. The water circulation system of a public or semipublic spa shall have a turnover rate of at least once every 30 minutes. The water circulation system for a wading pool shall have a turnover rate of at least once every hour. The water circulation system shall be designed to give the proper turnover rate without exceeding the maximum filtration rate for the filter in R18-5-227(E).

C. Water circulation system components shall comply with American National Standard/NSF International Standard Number 50, "Circulation System Components and Related Materials for Swimming Pools, Spas/Hot Tubs," NSF International, 3475 Plymouth Road, P.O. Box 130140, Ann Arbor, Michigan [revised July, 1996, and no future editions] which is incorporated by reference and on file with the Office of the Secretary of State and the Department.

D. Water circulation system components shall be accessible for inspection, repair, or replacement.

E. Except as provided by this subsection, water withdrawn from a public or semipublic swimming pool or spa shall not be returned unless it has been filtered and adequately disinfected. Water may be withdrawn from a swimming pool for a water slide or a water fountain without being filtered or disinfected.

F. In a swimming pool complex with more than one swimming pool or where there is a combination of swimming pools and spas, each swimming pool and spa shall have a separate water circulation system.

G. Hydrotherapy jets or other devices which create roiling water or similar effects in a spa shall not be connected to the water

circulation system, but shall be operated through a separate system.

Historical Note

Adopted effective February 19, 1998 (Supp. 98-1). Manifest typographical error corrected in subsection (B) (Supp. 01-1).

R18-5-224. Piping and Fittings

A. The water velocity in discharge piping for public and semipublic swimming pools and spas shall not exceed 10 feet per second, except for copper discharge piping where the velocity shall not exceed 8 feet per second. The water velocity in suction piping shall not exceed 6 feet per second. Piping shall be sized to permit the rated flows for filtering and cleaning without exceeding the maximum head of the pump.

B. Water circulation system piping and fittings shall be constructed of materials that are able to withstand 150% of normal operating pressures. Suction piping shall be of sufficient strength so that it does not collapse when there is a complete shutoff of flow on the suction side of the pump. A licensed Arizona contractor shall conduct an induced static hydraulic pressure test of the water circulation system piping at 25 pounds per square inch for at least 30 minutes. The pressure test shall be performed before the deck is poured. Pressure in the water circulation system piping shall be maintained during the deck pour.

C. Water circulation piping and fittings shall be made of nontoxic, corrosion-resistant materials.

D. Water circulation piping and fittings shall be installed so that piping or fittings do not project into a public or semipublic swimming pool or spa in a manner that is hazardous to users.

E. Piping that is subject to damage by freezing shall have a uniform slope in one direction and shall be equipped with valves that will permit the complete drainage of the water in the swimming pool or spa.

F. Piping shall be designed to drain the swimming pool or spa water by removing drain plugs, manipulating valves, or other means.

G. Piping systems shall be identified by color or by stencils or labels located at conspicuous points.

H. Plastic water circulation piping shall comply with American National Standard/NSF International Standard Number 14, "Plastics Piping System Components and Related Materials," NSF International, 3475 Plymouth Road, P.O. Box 130140, Ann Arbor, Michigan [revised September, 1996, and no future editions] which is incorporated by reference and on file with the Office of the Secretary of State and the Department.

Historical Note

Adopted effective February 19, 1998 (Supp. 98-1).

R18-5-225. Pumps and Motors

A. A pump and motor shall be provided for each water circulation system. The pump shall be sized to meet but not to exceed the flow rate required for filtering against the total head developed by the complete water circulation system. The pump shall be sized to comply with the turnover rate prescribed in R18-5-223(B).

B. Pumps and motors shall be readily and easily accessible for inspection, maintenance, and repair. When the pump is below the waterline, valves shall be installed on permanently connected suction and discharge lines. The valves shall be readily and easily accessible for maintenance and removal of the pump.

C. Each motor shall have an open, drip-proof enclosure. Each motor shall be constructed electrically and mechanically to perform satisfactorily and safely under the conditions of load in the environment normally encountered in swimming pool or spa installations. Each motor shall be capable of operating the pump under full load with a voltage variation of ± 10% from the nameplate rating. Each motor shall have thermal or current overload protection to provide locked rotor and running protection. Thermal or current overload protection may be built into the motor or in the line starter.

D. The pump shall be equipped with an emergency shut-off switch that is located within the swimming pool or spa enclosure to cut off power to the water circulation system if someone is entrapped on a main drain or suction outlet.

Historical Note

Adopted effective February 19, 1998 (Supp. 98-1).

R18-5-226. Drains and Suction Outlets

A. A public and semipublic swimming pool shall be equipped with at least two main drains located in the deepest part of the swimming pool or a single gravity drain that discharges to a surge tank.

B. Each main drain shall be covered by a grate that is not be readily removable by users. The openings in the grate shall have a total area that is at least four times the area of the drain pipe.

C. The spacing of the main drains shall not be greater than 20 feet on centers and not more than 15 feet from each side wall.

D. A minimum of two suction outlets shall be provided for each pump in a suction outlet system for a public or semipublic spa. The suction outlets shall be separated by a minimum of 3 feet or located on two different planes [that is, one suction outlet on the bottom and one on a vertical wall or one suction outlet each on two separate vertical walls]. The suction outlets shall be plumbed to draw water through them simultaneously through a common line to the pump. Suction outlets shall be plumbed to eliminate the possibility of entrapping suction.

E. If the suction outlet system for a public or semipublic swimming pool or spa has multiple suction outlets that can be isolated by valves, then each suction outlet shall protect against user entrapment by either an antivortex cover, a grate, or other means approved by the Department.

F. A public or semipublic spa may be equipped with a single gravity drain which discharges to a surge tank instead of suction outlets. The total velocity of water through grate openings of the drain shall not exceed 2 feet per second.

Historical Note

Adopted effective February 19, 1998 (Supp. 98-1).

R18-5-227. Filters

A. Filters shall be designed, located, and constructed to permit removal of filter manhole covers or heads for inspection, replacement, or repair of filter elements or filter media. No filtration system shall be installed beneath the surface of the ground or within an enclosure without providing adequate access for inspection and maintenance.

B. Pressure-type filters shall be equipped with a means to release internal pressure. Each pressure filter shall be equipped with an air relief piping system connected at an accessible point near the crown. Automatic air relief systems may be used instead of manual systems. The design of a filter with an automatic air relief system as its principal means of air release shall include lids that provide a slow and safe release of pressure. The design of a separation tank used in conjunction with any filter tank shall include a manual means of air release or a lid which provides a slow and safe release of pressure as it is opened.

C. Pressure filter systems shall be equipped with a sight glass installed on the waste discharge pipe.

D. Swimming pool and spa filters shall comply with American National Standard/NSF International Standard Number 50, "Circulation System Components and Related Materials for Swimming Pools, Spas/Hot Tubs," NSF International, 3475 Plymouth Road, P.O. Box 130140, Ann Arbor, Michigan [revised July, 1996, and no future editions] which is incorporated by reference and on file with the Office of the Secretary of State and the Department.

E. The maximum filtration rate shall not exceed the design flow rate prescribed by the National Sanitation Foundation Standard 50 for commercial filters. In no case shall the maximum filtration rate exceed the following:

1. The rate of filtration in a high-rate sand filter shall not exceed 25 gallons/minute/square foot.
2. The rate of filtration of a diatomaceous earth filter shall not exceed 2 gallons/minute/square foot.
3. The rate of filtration of a cartridge filter shall not exceed 0.375 gallons/minute/square foot.

Historical Note

Adopted effective February 19, 1998 (Supp. 98-1).

R18-5-228. Return Inlets

A. Adjustable return inlets shall be provided for each public and semipublic swimming pool or spa. Return inlets shall be designed, sized, and installed to produce a uniform circulation of water throughout the swimming pool or spa. Where surface skimmers are used, return inlets on vertical walls shall be located to help bring floating particles within range of the surface skimmers.

B. A public or semipublic swimming pool shall have a minimum of two return inlets, regardless of the size of the swimming pool. The number of return inlets shall be based on two return inlets per 600 square feet of surface area, or fraction thereof.

C. Return inlets in a public or semipublic swimming pool shall be on a closed loop piping system. Public or semipublic spas with three or more return inlets shall be on a closed loop piping system.

D. Where the width of a public or semipublic swimming pool exceeds 30 feet, bottom returns shall be required. Bottom returns shall be flush with the pool bottom or designed to prevent injury to users.

Historical Note

Adopted effective February 19, 1998 (Supp. 98-1).

R18-5-229. Gauges

A. Pressure gauges shall be installed on the water circulation system for each public and semipublic swimming pool and spa. Pressure gauges shall be installed in accessible locations where they can be read easily.

B. Pressure gauges shall be installed on the inlet and outlet manifold of the filter. Pressure gauges shall read at intervals of 1 pound per square inch [psi].

Historical Note

Adopted effective February 19, 1998 (Supp. 98-1).

R18-5-230. Flow meter

A public swimming pool shall be equipped with, a flow meter which indicates the rate of backwash through the filter. The flow meter shall be installed between the pump and the filter on a straight section of pipe in accordance with the manufacturer's specifications in a location where it can be read easily. The flow meter shall measure the rate of flow through the filter in gallons per minute and shall be accurate to within 5% under all conditions of flow. The flow meter shall have an indicator with a range of at least 150% of the normal flow rate.

Historical Note

Adopted effective February 19, 1998 (Supp. 98-1).

R18-5-231. Strainers

The water circulation system shall include a removable strainer located upstream of the pump to prevent solids, debris, hair, or lint from reaching the pump and filters. The strainer shall be made of corrosion-resistant material. A strainer shall have openings that have a total area which is equal to at least four times the area of the suction piping.

Historical Note

Adopted effective February 19, 1998 (Supp. 98-1).

R18-5-232. Overflow Collection Systems

A. An overflow collection system shall be installed in each public or semipublic swimming pool or spa.

B. The overflow collection system shall be designed and constructed so that the water level of the swimming pool is maintained at the mid-point of the operating range of the system's rim or weir device.

C. Rim type overflow collection systems shall be installed on at least two opposite sides and have a total length of at least 50% of the perimeter of a public or semipublic swimming pool. The overflow collection system shall be capable of carrying 50% of the design capacity of the water circulation system.

D. If overflow gutters are used, they shall be installed continuously around the swimming pool with the lip of the gutter level throughout its perimeter. Overflow gutters shall be provided with sufficient opening at the top and width at the bottom to permit easy cleaning. The overflow gutter bottom shall be pitched 1/4 inch per foot to drainage outlets located not more than 10 feet apart. Outlet piping shall be sized to circulate at least 50% of the capacity of the water circulation system and be properly covered by a drain grate. The surge tank for the overflow gutters shall be equipped with float controls which regulate the main drain, fill line, and overflow. The system surge capacity shall not be less than one gallon for each square foot of swimming pool surface area. Stainless steel gutters and other specialty gutter systems may be used if they are hydraulically equivalent to overflow gutters.

E. Surface skimmers shall be recessed into the swimming pool or spa wall and shall be installed to achieve effective skimming action throughout the swimming pool or spa.

1. A surface skimmer shall be provided for each 400 square feet of surface area, or fraction thereof, of a public or semipublic swimming pool. A minimum of two surface skimmers are required in a public or semipublic swimming pool. A surface skimmer shall be provided for each 200 square feet of surface area, or fraction thereof, of a public or semipublic spa.
2. The overflow slot shall be set level and shall not be less than 8 inches in width at the narrowest section.
3. The rate of flow through the skimmers shall be a minimum of 75% of the water circulation system capacity. Surface skimmers shall be designed to carry at least 30 gallons per minute per lineal foot of weir throat.
4. Where three or more surface skimmers are used, they must be on a closed loop piping system.
5. At least one surface skimmer shall be located on the side or near the corner of the swimming pool that is downwind of the area's prevailing winds.
6. Main drain piping shall be designed to carry at least 50% of the design flow.

F. Mixed inlet types [for example, surface skimmers and gutters] are prohibited in a public or semipublic swimming pool.

Historical Note

Adopted effective February 19, 1998 (Supp. 98-1).

R18-5-233. Vacuum Cleaning Systems

A vacuum cleaning system shall be provided for each public and semipublic swimming pool. A vacuum cleaning system shall not create a hazard or interfere with the operation or use of the swimming pool. In integral systems, a sufficient number of vacuum cleaner fittings shall be located in accessible positions at least 10 inches below the water line. Alternatively, vacuum cleaner fittings may be installed as an attachment to the surface skimmers. A pressure cleaning system may be installed in addition to the required vacuum cleaning system.

Historical Note

Adopted effective February 19, 1998 (Supp. 98-1).

R18-5-234. Disinfection

A. An adjustable automatic chemical feeder shall be provided to ensure the continuous disinfection of the water in a public or semipublic swimming pool or spa. Timers on disinfection equipment are prohibited. Disinfection shall be accomplished by chlorination or by another method that is approved by the Department. The method of disinfection shall effectively maintain an adequate disinfectant residual in the water which is subject to field testing by methods that are easy to use and accurate.

1. Chlorine disinfection equipment for a public or semipublic swimming pool shall be designed to maintain a free chlorine residual of 1.0 to 3.0 ppm. Chlorine disinfection equipment for a public or semipublic spa shall be designed to maintain a free chlorine residual of 3.0 to 5.0 ppm.
2. Bromine disinfection equipment for a public or semipublic swimming pool shall be designed to maintain a bromine residual of 2.0 to 4.0 ppm. Bromine disinfection equipment for a public or semipublic spa shall be designed to maintain a bromine residual of 3.0 to 5.0 ppm.

B. The use of chlorinated isocyanurates or cyanuric acid stabilizer for disinfection and stabilization is permitted. If used, chlorinated isocyanurates shall be fed so as to maintain required disinfectant residual levels. Cyanuric acid levels, whether from chlorinated isocyanurates or from the separate addition of cyanuric acid stabilizer, shall not exceed 150 ppm.

C. The use of chloramines as a primary disinfectant of swimming pool or spa water is prohibited.

D. The addition of gaseous disinfectant directly into a public or semipublic swimming pool is prohibited. The addition of dry or liquid disinfectant directly into a public or semipublic swimming pool or spa for routine disinfection is prohibited. This prohibition does not prohibit the use of liquid or dry disinfectants for shock treatment of a swimming pool or spa. A chlorine gas disinfection system shall not be used for the disinfection of water in a public or semipublic spa.

E. A common chlorine gas disinfection system may be utilized in separate swimming pools if separate metering and feeding devices are provided for each swimming pool.

F. If gaseous chlorine is used for disinfection, the following shall be provided:

1. The chlorinator, chlorine cylinders, and associated chlorination equipment shall be located in a separate well ventilated enclosure at or above ground level. The enclosure shall be reasonably gas-tight, noncombustible, and corrosion-resistant. The door of the enclosure shall open to the outside and shall not open directly toward the swimming pool.
2. If chlorination equipment is placed in a room, then an exhaust fan or gravity ventilation system shall be provided. Mechanical exhausters shall take suction 6 inches or less above the floor and discharge through corrosion-resistant louvers to a safe outside location. A gravity ventilation system shall be designed and constructed to discharge to the outside from floor level. Fresh air intakes shall be located no closer than 3 feet above the ventilation discharge. Chlorine room exhausts shall be directed away from the swimming pool to an area which is normally unoccupied. Chlorine room fans shall be capable of completely changing the air in the room at least once a minute.
3. Electrical switches to control lighting and ventilation in the chlorine room shall be located on the outside of the enclosure and adjacent to the door.
4. Chlorine cylinders shall be kept in an upright position and securely anchored to prevent them from falling. Chlorine cylinders may be stored indoors or out. If stored outside, chlorine cylinders shall not be stored in direct sunlight. Chlorine cylinders shall not be stored near an elevator, ventilation system, or heat source.
5. A warning sign shall be placed on the outside of the door to the chlorine room which cautions persons of the danger of chlorine gas within the enclosure. The warning shall be in letters 3 inches high or larger. The door to the chlorine room shall be provided with a shatter resistant inspection window.
6. Chlorinators shall be a solution-feed type, capable of delivering chlorine at its maximum rate without releasing chlorine gas to the atmosphere. Chlorinators shall be designed to prevent the backflow of water into the chlorine solution container.

G. Granular, tablet, stick, and other forms of dry disinfectant shall be fed by an adjustable automatic feeding device.

H. Disinfection equipment and chemical feeders shall comply with the requirements set forth in American National Standard/NSF International Standard 50, "Circulation System Components and Related Materials for Swimming Pools, Spas/Hot Tubs," NSF International, 3475 Plymouth Road, P.O. Box 130140, Ann Arbor, Michigan [revised July, 1996, and no future editions] which is incorporated by reference and on file with the Office of the Secretary of State and the Department.

I. If a chemical feeder is used, it shall be installed to inject solution downstream from the filter and the heater. An erosion-type feeder may be installed to feed solution to the suction side of the pump. A chemical feeder shall be wired so it cannot operate unless the filter pump is running.

Historical Note

Adopted effective February 19, 1998 (Supp. 98-1).

R18-5-235. Cross-Connection Control

A. Cross-connections between the distribution system of a public water system and the water circulation system of a public or semipublic swimming pool or spa are prohibited.

B. Potable water for make-up water purposes may be introduced into a public or semipublic swimming pool or spa in any of the following ways:

1. Through an over-the-rim spout with an air-gap of at least twice the diameter of the pipe and not less than 6 inches above the overflow level. If an over-the-rim spout is used, it shall be located so that it does present a tripping hazard. The open end of an over-the-rim spout shall have no

sharp edges and shall not protrude more than 2 inches beyond the edge of the swimming pool or spa wall;
2. Through a float controlled make-up water feed tank with an air gap of at least 3 inches above the overflow level; or
3. Through a submerged inlet that is protected against back-siphonage by at least a pressure vacuum breaker that is installed so that the bottom of the backflow prevention assembly is a minimum of 12 inches above the level of the coping.

Historical Note

Adopted effective February 19, 1998 (Supp. 98-1).

R18-5-236. Disposal of Filter Backwash, Wasted Swimming Pool or Spa Water, and Wastewater

All sewage from plumbing fixtures, including urinals, toilets, lavatories, showers, drinking fountains, floor drains, and other sanitary facilities shall be disposed of in a sanitary manner. Filter backwash and wasted swimming pool or spa water shall be discharged into a sanitary sewer through an approved air gap, an approved subsurface disposal system, or by other means that are approved by the Department. The method of disposal shall comply with applicable disposal requirements established by a county, municipal, or other local authority. There shall be no direct physical connection between the sewer system and the water circulation system of a public or semipublic swimming pool or spa.

Historical Note

Adopted effective February 19, 1998 (Supp. 98-1).

R18-5-237. Lifeguard Chairs

Each public swimming pool shall have at least one elevated lifeguard chair for each 3,000 square feet of pool surface area or fraction thereof. At least one lifeguard chair shall be located close to the deep area of the swimming pool and shall provide a clear, unobstructed view of the swimming pool bottom. If a public swimming pool is provided with more than one lifeguard chair or the width of the public swimming pool is 45 feet or more, then lifeguard chairs shall be located on each side of the public swimming pool.

Historical Note

Adopted effective February 19, 1998 (Supp. 98-1).

R18-5-238. Lifesaving and Safety Equipment

A. Public and semipublic swimming pools shall have lifesaving and safety equipment that is conspicuously and conveniently located and maintained ready for immediate use at all times.
B. Each public or semipublic swimming pool shall have one ring buoy or a similar flotation device. Each ring buoy or flotation device shall be attached to 50 feet of 1/4 inch rope.
C. Each semipublic and public swimming pool shall have at least one shepherd crook that is mounted on a rigid 16-foot pole.

Historical Note

Adopted effective February 19, 1998 (Supp. 98-1).

R18-5-239. Rope and Float Lines

A rope and float line shall be installed across each public swimming pool on the shallow side of the break in grade between the shallow and deep portions of the pool [that is, within 1 to 2 feet of the point where the floor slope begins to exceed 1 foot in 10 feet]. The rope shall be a minimum of 3/4 inch in diameter and supported by floats spaced at intervals not greater than 7 feet. The rope and float line shall be securely fastened to wall anchors that are made of corrosion-resistant materials. The wall anchors shall be recessed or have no projection that constitutes a hazard when the float line is removed.

Historical Note

Adopted effective February 19, 1998 (Supp. 98-1).

R18-5-240. Barriers

A. A public swimming pool or spa and deck shall be entirely enclosed by a fence, wall, or barrier that is at least 6 feet high. A semipublic swimming pool or spa and deck shall be entirely enclosed by a fence, wall, or barrier that is at least 5 feet high. The height of the fence, wall, or barrier shall be measured on the side of the barrier which faces away from the swimming pool or spa.
B. Fences or walls shall:
1. Be constructed to afford no external handholds or footholds;
2. Be of materials that are impenetrable to small children;
3. Have no openings or spacings of a size that a spherical object 4 inches in diameter can pass through; and
4. Be equipped with a gate that opens outward from the swimming pool or spa. The gate shall be equipped with a self-closing and self-latching closure mechanism or a locking closure located at or near the top of the gate, on the pool side of the gate, and at least 54 inches above the floor.

C. The distance between the horizontal components of a fence shall not be less than 45 inches apart. The horizontal members shall be located on the interior side of the fence. Spacing or openings between vertical members shall be of a size that a spherical object 4 inches in diameter cannot pass through.
D. The maximum mesh size for a wire mesh or chain link fence shall be a 1 3/4 inches square.
E. Masonry or stone walls shall not contain indentations or protrusions except for normal construction tolerances and tooled masonry joints.
F. If a wall of a building serves as part of the barrier around a public or semipublic swimming pool or spa, there shall be no direct access to the swimming pool or spa through the wall except as follows:
1. Windows leading to the swimming pool or spa area shall be equipped with a screwed-in place wire mesh screen or a keyed lock that prevents opening the window more than 4 inches.
2. A hinged door leading to the swimming pool or spa area shall be self-closing and shall have a self-latching device. The release mechanism of the self-latching device shall be located at least 54 inches above the floor.
3. If an additional set of doors is required by the fire code allowing access to the swimming pool or spa, they shall be self-closing and self-latching, equipped with panic bars no less than 54 inches from the floor to the bottom of the bar and designated "For Emergency Use Only."
4. Sliding doors leading to the swimming pool or spa area are prohibited except for sliding doors that are self-closing and self-latching.

G. If a barrier is composed of a combination concrete masonry unit and wrought-iron, the wrought iron portion shall be installed flush with the outside vertical surface of the concrete masonry unit. The space between the wrought iron and the concrete masonry unit shall be 1/2 inch or less. The vertical members of the wrought iron shall be spaced 4 inches on center.
H. Filtration, disinfection, and water circulation equipment shall be enclosed by a wall or fence.

Historical Note

Adopted effective February 19, 1998 (Supp. 98-1).

R18-5-241. Public Swimming Pools; Bathhouses and Dressing Rooms

A. Separate dressing rooms shall be provided for each sex. Dressing rooms shall be equipped with baskets or other checking facilities.

B. All entrances to and exits from the dressing rooms shall be effectively screened to interrupt the line of sight of persons outside the dressing rooms.

C. Walls and partitions of dressing rooms, locker rooms, toilets, and showers shall be light colored, smooth, nonabsorbent, and easily cleanable. Concrete or pumice blocks used for interior wall construction in these locations shall be finished and sealed to provide a smooth and easily cleanable surface. Partitions shall be designed so that a waterway is provided between partitions and the floor to permit thorough cleaning of the walls and floor areas with hoses and brooms.

D. Floors shall be of nonslip construction, free of cracks or openings, and sloped to adequate drains so the surface will be free of standing water and puddles. Floors shall be sloped not less than 1/4 inch per foot toward the drains to ensure positive drainage. Carpeting is prohibited.

E. All furniture shall be of simple character and easily cleanable. Locker compartments, partitions, booths, furniture, and other appurtenances in dressing rooms shall be so installed or raised above the floor to permit washing down the dressing rooms and bathhouse interiors.

F. An adequate number of hose bibs shall be provided for washing down the dressing room or bathhouse interior.

G. Dressing rooms, toilets, and showers shall be provided with adequate lighting and ventilation.

H. Toilet facilities shall be provided for each sex. For male users, there shall be one toilet and one urinal for each 100 bathers or fraction thereof. For female users, there shall be one toilet for each 50 bathers, or fraction thereof. In no case shall less than two toilets be provided for female users. Sanitary napkin dispensers shall be installed in toilet or shower areas designated for female users.

I. Shower and handwashing facilities with hot and cold water and soap shall be provided for each dressing room. Hot and cold water shall be provided at all shower heads. The water heater and thermostatic mixing valve shall be inaccessible to users and shall be capable of providing two gallons per minute of 90°F water to each shower head. A minimum of two shower heads shall be provided in each dressing room. Each dressing room shall have one shower head for each 50 bathers or fraction thereof.

J. One lavatory with an unbreakable mirror shall be provided in each dressing room for the first 100 users. An additional lavatory and unbreakable mirror shall be provided for each additional 100 users or fraction thereof. Soap dispensers for providing either liquid or powdered soap shall be provided at each lavatory. Soap dispensers shall be made of metal or plastic with no glass permitted.

Historical Note

Adopted effective February 19, 1998 (Supp. 98-1).

R18-5-242. Semipublic Swimming Pools; Toilets and Lavatories

A. A bathroom with a minimum of one toilet shall be provided for each sex.

B. Each bathroom shall have at least one lavatory. Soap dispensers for providing either liquid or powdered soap shall be provided at each lavatory. Soap dispensers shall be made of metal or plastic with no glass permitted.

C. An establishment that operates a semipublic swimming pool or spa and provides a private room with a toilet and lavatory for bathers shall be deemed to have complied with the requirements of this Section.

Historical Note

Adopted effective February 19, 1998 (Supp. 98-1).

R18-5-243. Drinking Water Fountains

Drinking water from an approved source and dispensed through one or more drinking fountains shall be located on the deck of each public swimming pool or spa.

Historical Note

Adopted effective February 19, 1998 (Supp. 98-1).

R18-5-244. Wading Pools

A. A wading pool is a type of public or semipublic swimming pool. The design criteria prescribed in this Article for public or semipublic swimming pools apply, except as provided in this Section.

B. A wading pool shall be physically set apart from public and semipublic swimming pools.
1. A wading pool shall be separated from a public swimming pool by a minimum 4-foot high fence or partition with a self-closing, self-latching gate.
2. A wading pool shall be separated from a semipublic swimming pool by at least 4 feet of deck.
3. A wading pool shall not be located adjacent to the deep area of a public or semipublic swimming pool.

C. A wading pool shall have a maximum depth of 24 inches. Water depths may be reduced from the stated maximums and brought to zero at the most shallow point of the wading pool.

D. The floor of a wading pool shall be uniform with a maximum slope of 1 foot of fall in 10 feet. The floor of a wading pool shall have a slip-resistant surface.

E. All wading pools shall have separate equipment for water circulation and disinfection. There shall be no cross-connection between the water circulation system of a wading pool and a public or semipublic swimming pool. The water in a wading pool shall have a maximum turnover cycle of 1 hour.

F. At least two main drains shall be provided at the deepest point in a wading pool. Each main drain shall be covered by a grate which cannot be removed by users. The openings in the grate shall have a total area that is at least four times the area of the drain pipe. In the alternative, a wading pool may be equipped with a single gravity drain which discharges to a surge tank.

G. Surface skimmers shall be provided on the basis of at least one skimmer for each 200 square feet of wading pool surface area. Surface skimmer flow rates shall be the same as required for public and semipublic swimming pools. Where only one skimmer is provided, the main drain may be connected through the skimmer.

H. Return inlets shall be provided and arranged to produce a uniform circulation of water and maintain a uniform disinfectant residual throughout the wading pool. Where three or more return inlets are required, they shall be on a closed loop piping system.

I. Suction outlets in a wading pool shall have plumbing provisions so as to relieve any possibility of entrapping suction.

J. Gaseous chlorine shall not be used for the disinfection of wading pool water.

K. A drinking fountain at a height convenient to small children or a drinking fountain with a raised step shall be provided in the area of the wading pool.

Historical Note

Adopted effective February 19, 1998 (Supp. 98-1).

R18-5-245. Timers for Public and Semipublic Spas

The timer for a public or semipublic spa which controls the hydrotherapy jets shall be located at least 5 feet from the spa and shall have a maximum time limit of 15 minutes.

Historical Note

Adopted effective February 19, 1998 (Supp. 98-1).

R18-5-246. Air blower and Air Induction Systems for Public and Semipublic Spas

An air blower system or air induction system for a public or semipublic spa shall comply with the following requirements:

1. The system shall prevent water backflow which could cause an electrical shock hazard.
2. Air intake sources shall not introduce water, dirt, or contaminants into the spa.
3. The system shall be properly sized for a commercial spa application.
4. If the air blower is installed within an enclosure or indoors, then adequate ventilation shall be provided.
5. Integral air passages shall be pressure tested and shall provide structural integrity to a value of 1 1/2 times the intended working pressure.

Historical Note

Adopted effective February 19, 1998 (Supp. 98-1).

R18-5-247. Water Temperature in Public and Semipublic Spas

The temperature of heated water coming into a public or semipublic spa shall not exceed 104°.

Historical Note

Adopted effective February 19, 1998 (Supp. 98-1).

R18-5-248. Special Use Pools

A. A person who intends to construct a special use pool shall notify the Department and provide plans, specifications, and a description of the intended use of the special use pool. The Department shall use best professional judgment in approving a special use pool, taking into consideration the intended use of the pool, the conditions under which it will operate, and the safety of users. The Department may consider the design requirements prescribed by an official sanctioning athletic body such as the National Collegiate Athletic Association [NCAA], National Federation of State High School Associations [NFSHSA], U.S. Swimming, U.S. Diving, or the Internationale de Natation Amateur [FINA] in using best professional judgement to approve a special use pool that is intended for competitive swimming and diving.

B. A special use pool that is designed with exercise or training bars in the pool shall be restricted to the special use when the bars are located in the pool. The bars shall:

1. Be constructed of durable and corrosion-resistant material;
2. Be sealed, welded shut, or capped at both ends to prevent retention of water within the bars;
3. Bars may be removable. Removable bars shall be wedge anchored in place and the anchors shall be covered. Water-tight anchor plugs [95% efficiency] shall be provided when the bars are removed; and
4. Extend not more than 4 inches from the side of the pool into the water. The minimum clear opening from the inside of the bar to the side of the swimming pool shall not be less than 2 inches.

D. A special use pool that is designed with a ramp shall comply with the following:

1. The ramp shall be constructed of slip-resistant material;
2. The slope of the ramp shall not exceed 1 foot in 12 feet;
3. The width of the ramp shall be at least 3 feet;
4. The ramp shall have a level platform at the top and the bottom of the ramp;
5. The ramp shall be equipped with at least a 3 1/2 foot high guardrail installed on the deck and extending the length of the ramp;
6. The ramp shall be constructed with return inlets located on the pool and ramp walls along the length of the ramp.

Historical Note

Adopted effective February 19, 1998 (Supp. 98-1).

R18-5-249. Variances

A. The Department may grant a variance from a requirement prescribed in this Article upon a demonstration by the applicant that an alternative design, material, appurtenance, or technology is equivalent to a requirement prescribed in this Article. If a variance is granted, it shall be conditioned upon the applicant's use of the approved alternative.

B. The Department shall not grant a variance that results in an unreasonable risk to the health of swimming pool or spa users.

C. The applicant shall request a variance in writing. A variance request shall contain the following information:

1. Identification of the requirement prescribed in this Article for which a variance is requested;
2. Explanation of the reasons why the applicant cannot comply with the requirement;
3. A complete description of the alternative design, material, or technology to be installed and used in the swimming pool or spa, including design plans, specifications, and a description of the cost;
4. A demonstration that the alternative design, material, or technology to be installed and used in the swimming pool or spa is equivalent to the requirement in this Article and will not result in an unreasonable risk to users; and
5. A statement that the applicant will perform reasonable requirements prescribed by the Department that are conditions of a variance.

D. The applicant shall submit a request for a variance with an application for design approval. The Department shall determine whether the application for design approval and the variance request are complete. Within 30 days after the date of the submittal of the application for design approval and the variance request, the Department shall issue a written notice to the applicant that states that the request for a variance and the application for design approval are complete or which states that the request for a variance or the application for design approval is incomplete and identifies specific information deficiencies in the application for design approval or the variance request.

E. The Department may convene an advisory committee consisting of representatives of public and semipublic swimming pool and spa owners, public and semipublic swimming pool and spa building contractors, professional engineers, and county environmental and health departments to make a recommendation on a variance request.

F. If the Department grants the request for a variance, the Department shall identify the requirement for which the variance is granted, specify any conditions to the grant of a variance, and issue a design approval. If the Department denies the request for a variance, the Department shall issue a notice of intent to deny the request for a variance to the applicant. The notice shall state the reasons for the denial of the request for a variance and shall include a description of the applicant's right to request a hearing on the denial of the variance request pursuant to A.R.S. § 41-1092.03 and to request an informal settlement

conference pursuant to A.R.S. § 41-1092.06. If the Department denies a request for a variance, the Department may either deny the application for design approval or issue a design approval that requires compliance with the requirement for which the variance is requested.

G. In considering a request for a variance from a requirement prescribed in this Article, the Director shall consider the following factors:
1. The intended use of the public or semipublic swimming pool or spa;
2. The safety of the alternative design, material, or technology for which a variance is requested; and
3. The cost and other economic considerations associated with requiring compliance with the requirement prescribed in this Article as compared to the alternative for which a variance is requested.

Historical Note

Adopted effective February 19, 1998 (Supp. 98-1).

R18-5-250. Inspections

A. An inspector from the Department, upon presentation of credentials, may enter into any public or semipublic swimming pool or spa to determine compliance with this Article. The inspector may inspect records, equipment, and facilities; take photographs; and take other action reasonably necessary to determine compliance with this Article.

B. The owner or manager of a public or semipublic swimming pool or spa may accompany the inspector during an inspection.

C. An inspector from the Department may inspect a public or semipublic swimming pool or spa without giving prior notice of the inspection to the owner or operator of the swimming pool or spa.

Historical Note

Adopted effective February 19, 1998 (Supp. 98-1).

R18-5-251. Enforcement

A. If an inspector finds a violation of this Article, the Department may issue a notice of violation to the owner of a public or semipublic swimming pool or spa. A notice of violation shall state specifically the nature of the violation and shall allow a reasonable time for the owner to correct the violation.

B. If the Director has reasonable cause to believe that a person has constructed a public or semipublic swimming pool or spa in violation of this Article, the Director may order the closure of the swimming pool or spa by issuing a cease and desist order by following the procedures for abatement of environmental nuisances in A.R.S. § 49-142.

Historical Note

Adopted effective February 19, 1998 (Supp. 98-1).

Illustration A. Diving Well Dimensions for Swimming Pools

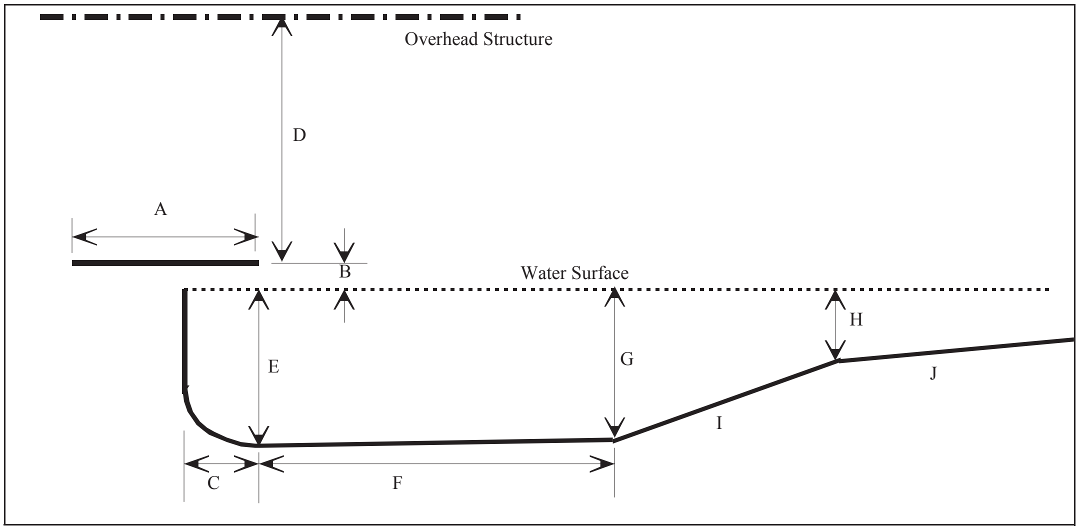

Note: This profile does not apply to a special use pool that is designed for competitive diving.

A. Maximum length of diving board	10 feet
B. Maximum height of board above the water	20 inches
C. Overhang of the board from wall	Minimum: 2 feet<br>Maximum: 3 feet
D. Minimum distance to an overhead structure	15 feet
E. Minimum depth of water at the plummet	9 feet
F. Distance from plummet to start of upslope	18 feet
G. Minimum depth of water at start of the upslope	Depth of water at plummet minus 6 inches

H. Depth of water at the breakpoint	Public swimming pool: 5 feet<br>Semipublic swimming pool: 4 feet
I. Maximum slope: breakpoint towards deep end	1 foot of fall in 3 feet
J. Slope of bottom in shallow area	1 foot of fall in 10 feet
Minimum width of pool in diving area	20 feet
From plummet to pool wall at the side	10 feet

Historical Note
Adopted effective February 19, 1998 (Supp. 98-1).

Illustration B. Minimum Distance Requirements for Decks

Dimension	Public (in Feet)	Semipublic (in feet)
A	10	4
B	5	4
C	15	11

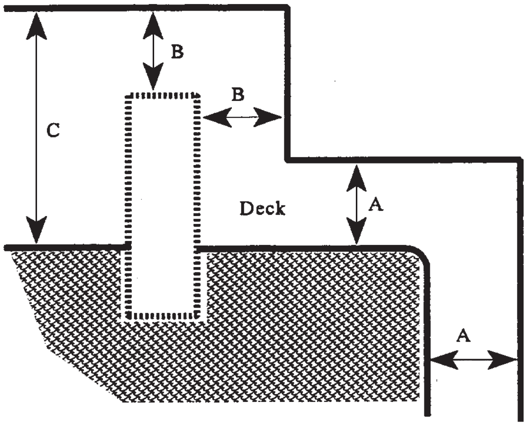

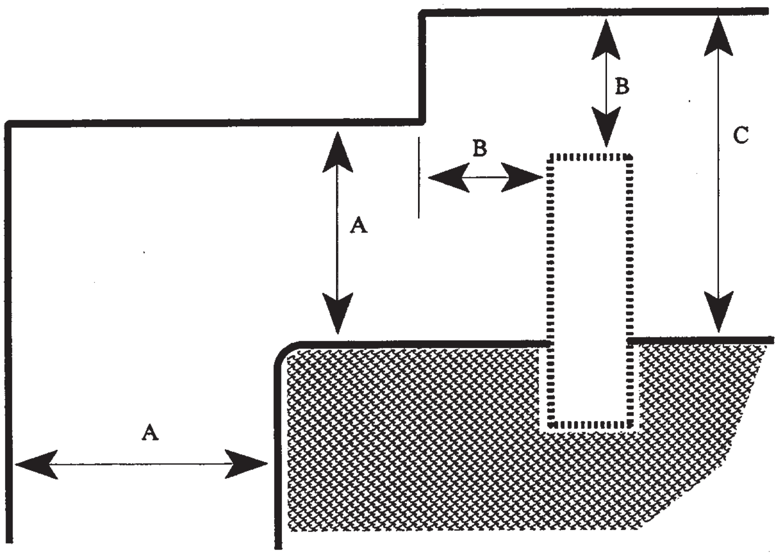

Historical Note
Adopted effective February 19, 1998 (Supp. 98-1).
