# Supplementary material for: Assessing state partner use of the Model Aquatic Health Code (MAHC): A cross comparison of five states with varying degrees of self-reported adoption status
Source: PLOS Water. Author manuscript; Available in PMC 2025 Jul 11. (PMC12247143; doi:10.1371/journal.pwat.0000276)
Supplement: S3_Text — S3 Text. Delaware pool codes. Title 18 health and safety administrative code from the Delaware Department of Health and Social Services, Division of Public Health for public swimming pools established on October 11, 2015. (PDF) [file NIHMS2046419-supplement-S3_Text.pdf]

DEPARTMENT OF HEALTH AND SOCIAL SERVICES

Division of Public Health

4400 Health Systems Protection

4464 Public Swimming Pools

1.0 General Provisions

1.1 Preamble. The Secretary of Delaware Health and Social Services adopts these Regulations pursuant to the authority vested in the Secretary by 16 **Del.C.** §122. These Regulations establish standards for the sanitary operation of public pools. For the purpose of these Regulations, the term “public pool” as defined in 16 **Del.C.** §122(1) and (3) (d) and (j). These Regulations provide a system of permitting and inspection of public pools and procedures for enforcement.

1.1.1 These Regulations are adopted on October 1, 2015 and have an effective date of October 11, 2015.

1.2 Purpose. To provide minimum standards for design, construction, maintenance and operation of public pools in the State of Delaware, and to assure a clean, healthful, and safe environment for all bathers using these pools. These Regulations in no way preclude a facility from establishing additional rules and operating procedures as long as they do not contradict those established herein.

1.3 Severability. In the event any particular clause or section of these Regulations should be declared invalid or unconstitutional by any court of competent jurisdiction, the remaining portions shall remain in full force and effect.

1.4 Exemptions.

1.4.1 There is no requirement of a lifeguard to be on duty at any pool of any motel, hotel or private campground facility, however an attendant is required.

1.4.2 These Regulations shall not apply to pools that are used by one (1) patron at a time and whose water is completely changed after each patron.

1.4.3 These regulations shall not apply to pools that have been granted private pool status.

1.5 Variance

1.5.1 A licensee may seek a variance from these Regulations by making a request for variance to the Division. The Division may grant a variance by modifying or waiving the requirements of these Regulations if in the opinion of the Division a health hazard or nuisance will not result from the variance.

1.5.2 A variance shall not be transferable from person to person, or from location to location.

1.5.3 If a variance is granted, the Division shall retain the information specified below in its records for the public pool.

1.5.3.1 A statement of the proposed variance of the requirement of these Regulations, citing the relevant section of these Regulations;

1.5.3.2 An analysis of the rationale for how the potential public health hazards or nuisances will be alternatively addressed by the proposal; and

1.5.3.3 Any other information requested by the Division that may be deemed necessary to render judgment.

1.5.3.4 A variance is rendered void upon occurrence of one or more of the following:

1.5.3.4.1 the physical facility is demolished or

1.5.3.4.2 a remodeling project in the facility includes the area(s) addressed in the variance

2.0 Definitions

For the purposes of these Regulations the following definitions apply:

“**Approved**” means acceptable to the Division, unless stated otherwise, based on its determination as to conformance with appropriate standards and good public health practices.

“**Aquatic Facility**” means a physical place that contains one or more aquatic venues and support infrastructure.

“**Aquatic Facility or Aquatic Venue Enclosure**” means an uninterrupted barrier surrounding and securing an aquatic facility or aquatic venue.

"**Aquatic Feature**" means an individual component within an aquatic venue. Examples include slides, structures designed to be climbed or walked across, and structures that create falling or shooting water.

"**Aquatic Venue**" means an artificially constructed structure or modified natural structure where the general public is exposed to water intended for recreational or therapeutic purpose. Such structures do not necessarily contain standing water, so water exposure may occur via contact, ingestion, or aerosolization. Examples include swimming pools, wave pools, lazy rivers, surf pools, spas (including spa pools and hot tubs), therapy pools, waterslide landing pools, spray pads, and other interactive water venues.

- "**Increased Risk Aquatic Venue**" means an aquatic venue which due to its intrinsic characteristics and intended users has a greater likelihood of affecting the of the bathers of that venue by being at increased risk for microbial contamination (e.g., by children less than 5 years old) or being used by people that may be more susceptible to infection (e.g., therapy patients with open wounds). Examples of increased-risk aquatic venues include spray pads, wading pools and other aquatic venues designed for children less than five years old as well as therapy pools.
- "**Lazy River**" means a channeled flow of water of near−constant depth in which the water is moved by pumps or other means of propulsion to provide a river−like flow that transports bathers over a defined path. A lazy river may include play features and devices. A lazy river may also be referred to as a tubing pool, leisure river, leisure pool or a current channel.
- "**Spa**" means a structure intended for either warm or cold water where prolonged exposure is not intended. Spa structures are intended to be used for bathing or other recreational uses and are not usually drained and refilled after each use. It may include, but is not limited to, hydrotherapy, air induction bubbles, and recirculation.
- "**Special Use Aquatic Venue**" means aquatic venues that do not meet the intended use and design

"**Attendant**" means a person who meets the training requirements of the Division specified in Section 9.7.

"**Attendant on Duty**" means an attendant who is either at poolside or in the pool (special situations), visually guarding the life of the bathers. An attendant on duty shall not be assigned other duties that will distract his/her attention from proper observation of the bathers, or prevent the rendering of immediate assistance to someone in distress. In facilities with a spa pool(s) only it means an attendant who is on the premises and can be easily located and summoned to render assistance to someone in distress. If the attendant is not in direct view of the spa pool when it is open, he/she shall be located such that the poolside alarm required by Section 9.19. can be easily heard.

"**Automated Controller**" means a system of at least one chemical probe, a controller, and auxiliary or integrated component that senses the level of one or more water parameters and provides a signal to other equipment to maintain the parameters within a user-established range.

"**Backflow**" means a hydraulic condition caused by a difference in water pressure that causes an undesirable reversal of the flow as the result of a higher pressure in the system than in its supply.

"**Bather**" means a person at an aquatic venue who has contact with water either through spray or partial or total immersion. The term bather as defined, also includes staff members, and refers to those users who can be exposed to contaminated water as well as potentially contaminate the water.

"**Bather Count**" means the number of bathers in an aquatic venue at any given time.

"**Beneficial Owner**" means an ownership interest in the entity owning the pool through direct ownership of the real property where the pool is located, direct ownership of stock in a stock corporation owning the real property where the stock represents an equity interest in the corporation, or direct ownership through being a member in a limited liability company (L.L.C.) or a partner in a partnership owning the real property upon which the pool is placed.

"**Breakpoint Chlorination**" means the conversion of inorganic chloramine compounds to nitrogen gas by reaction with Free Available Chlorine. When chlorine is added to water containing ammonia (from urine, sweat, or the environment, for example), it initially reacts with the ammonia to form monochloramine. If more chlorine is added, monochloramine is converted into dichloramine, which decomposes into nitrogen gas, hydrochloric acid and chlorine. The apparent residual chlorine decreases since it is partially reduced to hydrochloric acid. The point at which the drop occurs is referred to as the "breakpoint". The amount of free chlorine that must be added to the water to achieve breakpoint chlorination is approximately ten times the amount of combined chlorine in the water. As additional chlorine is added, all inorganic combined chlorine compounds disappear, resulting in a decrease in eye irritation potential and "chlorine odors."

“**Chemical Storage Space**” means a space in an aquatic facility used for the storage of pool chemicals such as acids, salt, or corrosive or oxidizing chemicals. “Chemical Storage Space” means a space in an aquatic facility used for the storage of pool chemicals such as acids, salt, or corrosive or oxidizing chemicals.

“**Chlorine**” means an element that at room temperature and pressure is a heavy greenish yellow gas with a characteristic penetrating and irritating smell; it is extremely toxic. It can be compressed in liquid form and stored in heavy steel tanks. When mixed with water, chlorine gas forms hypochlorous acid, the primary chlorine-based disinfecting agent, hypochlorite ion, and hydrochloric acid. Hypochlorous acid dissociation to hypochlorite ion is highly pH dependent. Chlorine is a general term used in these regulations which refers to hypochlorous acid and hypochlorite ion in aqueous solution derived from chlorine gas or a variety of chlorine-based disinfecting agents.

- “**Available Chlorine**” means the amount of chlorine in the +1 oxidation state, which is the reactive, oxidized form. In contrast, chloride ion (Cl–) is in the -1 oxidation state, which is the inert, reduced state. Available Chlorine is subdivided into Free Available Chlorine and Combined Available Chlorine. Pool chemicals containing Available Chlorine are both oxidizers and disinfectants. Elemental chlorine (Cl2) is defined as containing 100% available chlorine. The concentration of Available Chlorine in water is normally reported as mg/L (PPM) “as Cl2”, that is, the concentration is measured on a Cl2 basis, regardless of the source of the Available Chlorine.
- “**Free Chlorine Residual**” OR “**Free Available Chlorine**” means the portion of the total available chlorine that is not “combined chlorine” and is present as hypochlorous acid (HOCl) or hypochlorite ion (OCl-).The pH of the water determines the relative amounts of hypochlorous acid and hypochlorite ion. HOCl is a very effective bactericide and is the active bactericide in pool water. OCl- is also a bactericide, but acts more slowly than HOCl. Thus, chlorine is a more effective bactericide at low pH than at high pH. A free chlorine residual must be maintained for adequate disinfection.

“**Competitive Diving**” means either the training of divers or an actual diving competition among trained divers, which is sanctioned by the Federation International de Natation Amateur (FINA), the National Collegiate Athletic Association (NCAA), the National Federation of State High School Associations (NFSHSA) or United States Diving Inc. (USD). The institution which is sponsoring the diving training or diving competition shall be responsible for the hiring of a qualified person(s) who is competent and knowledgeable in the areas of diving mechanics and safety. This person(s) shall be present at every practice session and competition in order to ensure proper training, supervision and safety.

“**Contaminant**” means a substance that soils, stains, corrupts, or infects another substance by contact or association.

“**Contamination Response Plan**” means a plan for handling contamination from formed-stool, diarrheal-stool, vomit, and blood.

“**Corrosive Materials**” means pool chemicals, fertilizers, cleaning chemicals, oxidizing cleaning materials, salt, de-icing chemicals, other corrosive or oxidizing materials, pesticides, and such other materials which may cause injury to people or damage to the building, air-handling equipment, electrical equipment, safety equipment, or fire-suppression equipment, whether by direct contact or by contact via fumes or vapors, whether in original form or in a foreseeably likely decomposition, pyrolysis, or polymerization form. Refer to labels and SDS forms.

“**Crack**” means any and all breaks in the structural shell of a pool vessel or deck.

“**Cross-Connection**” means a connection or arrangement, physical or otherwise, between a potable water supply system and a plumbing fixture, tank, receptor, equipment, or device, through which it may be possible for non-potable, used, unclean, polluted and contaminated water, or other substances to enter into a part of such potable water system under any condition.

“**CT Value**” means a representation of the concentration of the disinfectant (C) multiplied by time in minutes (T) needed for inactivation of a particular contaminant. The concentration and time are inversely proportional; therefore, the higher the concentration of the disinfectant, the shorter the contact time required for inactivation. The CT value can vary with pH or temperature change so these values must also be supplied to allow comparison between values.

“**Cyanuric Acid**” means a chemical added to pool water intended to counteract degradation of the chlorine residual by ultraviolet light.

“**Deck**” means surface areas serving the aquatic venue, including the perimeter/wet deck, pool deck, and dry deck.

- “**Dry Deck**” means all pedestrian surface areas within the aquatic venue enclosure not subject to frequent splashing or constant wet foot traffic. The dry deck is not perimeter deck or pool deck, which connect the pool to adjacent amenities, entrances, and exits. Landscape areas are not included in this definition.
- “**Perimeter/Wet Deck**” means the hardscape surface area immediately adjacent to and within four feet (1.2 m) of the edge of the swimming pool also known as the “wet deck” area.
- “**Pool Deck**” means surface areas serving the aquatic venue, beyond perimeter deck, which is expected to be regularly trafficked and made wet by bathers.

“**Diaper-Changing Station**” means a hygiene station that includes a diaper-changing unit, hand-washing sink, soap and dispenser, a means for drying hands, trash receptacle, and disinfectant products to clean after use.

“**Diaper-Changing Unit**” means a diaper-changing surface that is part of a diaper-changing station.

“**Dichloramine**” means a disinfection by-product formed when chlorine binds to nitrogenous waste in pool water to form an amine- containing compound with two chlorine atoms (NHCl2). It is a known acute respiratory and ocular irritant.

“**Director**” means the Director of the Division of Public Health or an authorized agent.

“**Disinfection**” means a treatment that kills or irreversibly inactivates microorganisms (e.g., bacteria, viruses, and parasites); in water treatment, a chemical (commonly chlorine, chloramine, or ozone) or physical process (e.g., ultraviolet radiation) can be used.

“**Disinfection By-Product**” means a chemical compound formed by the reaction of a disinfectant (e.g. chlorine) with a precursor (e.g. natural organic matter, nitrogenous waste from bathers) in a water system (pool, water supply).

“**Diving**” means a head first entry into a body of water.

"**Division**" means the Division of Public Health of the Department of Health and Social Services, or anyone authorized by the Division as its designated representative, in conformance with 29 **Del.C.** §7904.

“**Emergency Action Plan**” means a plan that identifies the objectives that need to be met for a specific type of emergency, who will respond, what each person’s role will be during the response and what equipment is required as part of the response.

“**Enclosure**” means an uninterrupted constructed feature or obstacle used to surround and secure an area that is intended to deter or effectively prevent unpermitted, uncontrolled, and unfettered access. It is designed to resist climbing and to prevent passage through it and under it. Enclosure can apply to aquatic facilities or aquatic venues.

“**Environmental Health Survey**” means a comprehensive on-site review of the facilities, operation and management of a pool for the purpose of determining whether a safe environment and bathing water of acceptable quality are being provided.

“**EPA Registered**” means all products regulated and registered under the Federal Insecticide, Fungicide, and Rodenticide Act (FIFRA) by the U.S. Environmental Protection Agency (EPA; http://www.epa.gov/agriculture/ lfra.html). EPA registered products will have a registration number on the label (usually it will state “EPA Reg No.” followed by a series of numbers). This registration number can be verified by using the EPA National Pesticide Information Retrieval System (http://ppis.ceris.purdue.edu/#).

“**Equipment Room**” means a space intended for the operation of pool pumps, filters, heaters, and controllers. This space is not intended for the storage of hazardous pool chemicals.

“**Exit Gate**” means an emergency exit, which is a gate or door allowing free exit at all times.

“**Expansion Joint**” means a watertight joint provided in a pool vessel used to relieve flexural stresses due to movement caused by thermal expansion/contraction.

“**Fence**” means a continuous vertical barrier, either solid, or with openings, holes or gaps not exceeding four (4) inches in diameter or width, completely enclosing the pool area which will prevent the entry of small children and minimize the entry of unauthorized or unwary persons.

“**Flume**” means the riding channels of a waterslide which accommodate riders using or not using mats, tubes, rafts, and other transport vehicles as they slide along a path lubricated by a water flow.

“**Foot Baths**” means standing water in which bathers or aquatics staff rinse their feet.

“**Ground-Fault Circuit Interrupter**” means a device for protection of personnel that de-energizes an electrical circuit or portion thereof in the event of excessive ground current.

“**Guardian**” is an adult designated by a parent who is responsible for the children and their behavior.

“**Halogen**” means one of the chemical elements chlorine, bromine, or iodine.

“**Hand Wash Station**” means a location which has a hand wash sink, adjacent soap with dispenser, hand drying device or paper towels and dispenser, and trash receptacle.

“**Hot Water**” means an aquatic venue with water temperature over 90 degrees Fahrenheit (30 degrees Celsius).

“**Hygiene Facility**” means a structure or part of a structure that contains toilet, shower, diaper-changing unit, hand wash station, and dressing capabilities serving bathers and patrons at an aquatic facility.

“**Hygiene Fixtures**” means all components necessary for hygiene facilities including plumbing fixtures, diaper-changing stations, hand wash stations, trashcans, soap dispensers, paper towel dispensers or hand dryers, and toilet paper dispensers.

“**Hyperchlorination**” means the intentional and specific raising of chlorine levels for a prolonged period of time to inactivate pathogens following a fecal or vomit release in an aquatic venue as outlined in these regulations Section 9.28.

“**Imminent Health Hazard**” means a significant threat or danger to health that is considered to exist when there is evidence sufficient to show that a product, practice, circumstance, or event creates a situation that requires immediate correction or cessation of operation to prevent injury based on the number of potential injuries and the nature, severity, and duration of the anticipated injury or illness.

“**Inactive Pool**” means a pool which has been closed for twelve (12) or more continuous months.

“**Indoor Aquatic Facility**” means a physical place that contains one or more aquatic venues and the surrounding bather and spectator/stadium seating areas within a structure that meets the definition of “Building” per the 2012 International Building Code. It does not include equipment, chemical storage, or bather hygiene rooms or any other rooms with a direct opening to the aquatic facility. Otherwise known as a natatorium.

“**Infinity Edge**” means a pool wall structure and adjacent perimeter deck that is designed in such a way where the top of the pool wall and adjacent deck are not visible from certain vantage points in the pool or from the opposite side of the pool. Water from the pool flows over the edge and is captured and treated for reuse through the normal pool filtration system. They are often also referred to as “vanishing edges,” “negative edges,” or “zero edges.”

“**Inlet**” means wall or floor fittings where treated water is returned to the pool.

“**Interactive Water Play Aquatic Venue**” means any indoor or outdoor installation that includes sprayed, jetted or other water sources contacting bathers and not incorporating standing or captured water as part of the bather activity area. These aquatic venues are also known as splash pads, spray pads, wet decks. For the purposes of these regulations, only those designed to recirculate water and intended for public use and recreation shall be regulated.

“Island” means a structure inside a pool where the perimeter is completely surrounded by the pool water and the top is above the surface of the pool.

“Lifeguard on Duty” means a lifeguard who is either poolside or in the pool (special situations), visually guarding the life of the bathers. A lifeguard on duty shall not be assigned other duties that will distract his/her attention from proper observation of the bathers, or prevent the rendering of immediate assistance to someone in distress.

“**mg/L**” means milligrams per liter and is the equivalent metric measure to parts per million *(PPM)*.

“**Monitoring**” is the regular and purposeful observation and checking of systems or facilities and recording of data, including system alerts, excursions from acceptable ranges, and other facility issues. Monitoring includes human or electronic means.

“**No Diving Marker**” means a sign with the words “No Diving” and the universal international symbol for “No Diving” pictured as an image of a diver with a red circle with a slash through it.

“**NTU**” means Nephelometric Turbidity Unit which is a means of measuring the water turbidity.

“**Oocyst**” means the thick-walled, environmentally resistant structure released in the feces of infected animals that serves to transfer the infectious stages of sporozoan parasites (e.g., Cryptosporidium) to new hosts.

“**ORP**” means oxidation reduction potential which is an electrical measurement in millivolts (mV) of the strength of the disinfectant (oxidizer). A higher ORP means a higher disinfecting potential.

“**Oxidation**” means the process of changing the chemical structure of water contaminants by either increasing the number of oxygen atoms or reducing the number of electrons of the contaminant or other chemical

reaction, which allows the contaminant to be more readily removed from the water or made more soluble in the water. It is the “chemical cleaning” of pool water. Oxidation can be achieved by common disinfectants (e.g., chlorine, bromine), secondary disinfection/sanitation systems (e.g. ozone) and oxidizers (e.g. potassium monopersulfate).

“**Oxidation Reduction Potentia**l” means a measure of the tendency for a solution to either gain or lose electrons; higher (more positive) oxidation reduction potential indicates a more oxidative solution.

“**Patron**” means a bather or other person or occupant at an aquatic facility who may or may not have contact with aquatic venue water either through partial or total immersion. Patrons may not have contact with aquatic venue water, but could still be exposed to potential contamination from the aquatic facility air, surfaces, or aerosols.

“**Peninsula / Wing Wall**” means a structural projection into a pool intended to provide separation within the body of water.

“**Perimeter Gutter System**” means the alternative to skimmers as a method to remove water from the pool’s surface for treatment. The gutter provides a level structure along the pool perimeter versus intermittent skimmers.

“**Person**” means any corporation, company, association, firm, partnership, society, joint stock company or individual.

“**Person in Charge**” means the/an owner of the pool. This individual shall represent the pool at any hearing scheduled pursuant to 15.3. Every pool shall, upon request, provide the Division with the name of the person(s) in charge and how they can be contacted. An operator, hired by the/an owner, cannot be the person in charge.

“**pH**” means the negative log of the concentration of hydrogen ions. When water ionizes, it produces hydrogen ions (H+) and hydroxide ions (OH-). If there is an excess of hydrogen ions the water is acidic. If there is an excess of hydroxide ions the water is basic. pH ranges from 0 to 14. Pure water has a pH of 7.0. If pH is higher than 7.0, the water is said to be basic, or alkaline. If the water’s pH is lower than 7.0, the water is acidic. As pH is raised, more ionization occurs and chlorine disinfectants decrease in effectiveness.

“**Plumbing Fixture**” means a receptacle, fixture, or device that is connected to a water supply system or discharges to a drainage system or both and may be used for the distribution and use of water; for example: toilets, urinals, showers, and hose bibs. Such receptacles, fixtures, or devices require a supply of water; or discharge liquid waste or liquid-borne solid waste; or require a supply of water and discharge waste to a drainage system.

“**Pool**” means a subset of aquatic venues designed to have standing water for total or partial bather immersion. This does not include spas.

- “**Activity Pool**” means a water attraction designed primarily for play activity that uses constructed features and devices including pad walks, flotation devices, and similar attractions.
- “**Diving Pool**” means a pool used exclusively for diving.
- “**Landing Pool**” means an aquatic venue or designated section of an aquatic venue located at the exit of one or more waterslide flumes. The body of water is intended and designed to receive a bather emerging from the flume for the purpose of terminating the slide action and providing a means of exit to a deck or walkway area.
- “**Skimmer Pool**” means a pool using a skimmer system.
- “**Spa Pool**” means a pool containing water greater than ninety five (95) degrees Fahrenheit (oF), which is not emptied after each use, has a maximum depth of four (4) feet, is large enough for the immersion of one person and may have a high velocity air and/or water jet system.
- “**Spray Pads**” means any indoor or outdoor installation that includes sprayed, jetted or other water sources contacting bathers and not incorporating standing or captured water as part of the bather activity area. For the purposes of these Regulations only those designed to recirculate water and intended for public use and recreation shall be regulated.
- “**Surf Pool**” means any pool designed to generate waves dedicated to the activity of surfing on a surfboard or analogous surfing device commonly used in the ocean and intended for sport as opposed to general play intent for wave pools.
- “**Therapy Pool**” means a pool used exclusively for aquatic therapy, physical therapy, and/or rehabilitation to treat a diagnosed injury, illness, or medical condition, wherein the therapy is provided under the direct supervision of a licensed physical therapist, occupational therapist, or

athletic trainer. This could include wounded patients or immunocompromised patients whose health could be impacted if there is not additional water quality protection.

- "**Wading Pool**" means any pool used exclusively for wading and intended for use by young children where the depth does not exceed two feet (0.6 m).
- "**Wave Pools**" means any pool designed to simulate breaking or cyclic waves for purposes of general play. A wave pool is not the same as a surf pool, which generates waves dedicated to the activity of surfing on a surfboard or analogous surfing device commonly used in the ocean and intended for sport as opposed to general play intent for wave pools.

"Private Pool" means any indoor or outdoor artificial basin containing a body of water which is used for swimming, wading, diving, recreative bathing, or other aquatic purposes and is not open to the general public, or a limited section of the public, but is intended strictly for the use of the beneficial owner(s) and his/her/their family and/or their guests in either of the following situations:

- Individual beneficial owner or
- Multiple beneficial owners where all of the following can be demonstrated to the Division:
  - The pool is owned by a legal entity which is in turn owned by the beneficial owners.
  - Pool ownership is part of the ownership of real property by the beneficial owners.
  - The beneficial owners are able to assert ultimate dominion and control over access to and maintenance of the pool.
  - No pool memberships are available to non-beneficial owners.
- If it can be demonstrated that a pool meets all of the above criteria and if the owners want the pool to be approved as private, they shall contact the Division for the required procedure. Any multiple beneficial ownership pool that is approved private shall remain so until the owners notify the Division that the pool no longer meets all of the above criteria. These Regulations shall not apply to private pools.

"**Public Water Systems**" means water systems including community water systems, non-transient/non-community water systems, or transient non-community water systems with exceptions as noted by the Division and EPA.

"**Qualified Lifeguard**" means an individual who has successfully completed a Division recognized lifeguard training course offered by a Division recognized training agency, holds a current certificate for such training, has met the pre-service requirements, and is participating in continuing in-service training requirements of the aquatic facility.

"**Qualified Lifeguard Supervisor**" means an individual responsible for the oversight of lifeguard performance and emergency response at an aquatic facility. A qualified lifeguard supervisor is an individual who has successfully completed a lifeguard supervisor training course and holds an unexpired certificate for such training; and who has met the pre-service and continuing in-service requirements of the aquatic facility according to this code.

"**Qualified Operator**" means an individual, that is at least 18 years old, that is responsible for the operation and maintenance of the water and air quality systems and the associated infrastructure of the aquatic facility and who has successfully completed a Division recognized operator training course to operate an aquatic facility offered by a Division recognized training agency and holds a current certificate for such training.

"**Recessed Steps**" means a way of ingress/egress for a pool similar to a ladder but the individual treads are recessed into the pool wall.

"**Recirculation System**" means the combination of the main drain, gutter or skimmer, inlets, piping, pumps, controls, surge tank or balance tank to provide pool water recirculation to and from the pool and the treatment systems.

"**Reduction Equivalent Dose (RED) bias**" means a variable used in UV system validation to account for differences in UV sensitivity between the UV system challenge microbe and the actual microbe to be inactivated (e.g., Cryptosporidium).

"**Re-entrainment**" means a situation where the exhaust(s) from a ventilated source such as an indoor aquatic facility is located too close to the air handling system intake(s), which allows the exhausted air to be re-captured by the air handling system so it is transported directly back into the aquatic facility.

"**Responsible Supervisor**" means an individual on-site that is responsible for water treatment operations when a "qualified operator" is not on-site at an aquatic facility.

"**Robotic Cleaner**" means a modular vacuum system consisting of a motor-driven, in-pool suction device, either self-powered or powered through a low voltage cable, which is connected to a deck-side power supply.

"**Run-Out Slide**" means that part of a waterslide where riders are intended to decelerate and/or come to a stop. The runout is a continuation of the waterslide flume surface.

"**Safety**" (as it relates to construction items) means a design standard intended to prevent inadvertent or hazardous operation or use (i.e., a passive engineering strategy).

"**Safety Plan**" means a written document that has procedures, requirements and/or standards related to safety which the aquatic facility staff shall follow. These plans include training, emergency response, and operations procedures.

"**Safety Team**" means any employee of the aquatic facility with job responsibilities related to the aquatic facility's emergency action plan.

"**Sanitize**" means reducing the level of microbes to that considered safe by public health standards. This may be achieved through a variety of chemical or physical means including chemical treatment, physical cleaning, or drying.

"**Saturation Index**" means a mathematical representation or scale representing the ability of water to deposit calcium carbonate, or dissolve metal, concrete or grout.

"**Secondary Disinfection Systems**" means those disinfection processes or systems installed in addition to the standard systems required on all aquatic venues, which are required to be used for increased risk aquatic venues.

"**Secretary, Delaware Health and Social Services**" means the Administrator of the Department of Health and Social Services (DHSS) of the State of Delaware, who shall hereafter in this document be referred to as the Secretary, DHSS in conformance with 29 **Del.C.** §7904.

"**Shower**" means a device that sprays water on the body.

- "**Cleansing Shower**" means a shower located within a hygiene facility using warm water and soap. The purpose of these showers is to remove contaminants including perianal fecal material, sweat, skin cells, personal care products, and dirt before bathers enter the aquatic venue.
- "**Rinse Shower**" means a shower typically located in the pool deck area with ambient temperature water. The main purpose is to remove dirt, sand, or organic material prior to entering the aquatic venue to reduce the introduction of contaminants and the formation of disinfection by-products.

"**Skimmer**" means a device installed in the pool wall whose purpose is to remove floating debris and surface water to the filter. They shall include a weir to allow for the automatic adjustment to small changes in water level, maintaining skimming of the surface water.

"**Skimmer System**" means periodic locations along the top of the pool wall for removal of water from the pool's surface for treatment.

"**Slide**" means an aquatic feature where users slide down from an elevated height into water.

- "**Drop Slide**" means a slide that drops bathers into the water from a height above the water versus delivering the bather to the water entry point.
- "**Pool Slide**" means a slide having a configuration as defined in The Code of Federal Regulations (CFR) Ch. II, Title 16 Part 1207 by CSPC, or is similar in construction to a playground slide used to allow users to slide from an elevated height to a pool. They shall include children's (tot) slides and all other non- flume slides that are mounted on the pool deck or within the basin of a public swimming pool.
- "**Waterslide**" means a slide that runs into a landing pool or runout through a fabricated channel with flowing water.

"**Slip Resistant**" means a textured surface that is neither conducive to slipping when wet nor abrasive to bare feet and has a minimum static coefficient of friction of 0.6 (measured by an approved method).

"**Standard**" means something established by authority, custom, or general consent as a model for example.

"**Structural Crack**" means a break or split in the pool surface that weakens the structural integrity of the vessel.

"**Substantial Alteration**" means the alteration, modification, or renovation of an aquatic venue (for outdoor aquatic facilities) or indoor aquatic facility (for indoor aquatic facilities) where the total cost of the work exceeds 50% of the replacement cost of the aquatic venue (for outdoor aquatic facilities) or indoor aquatic facility (for indoor aquatic facilities).

"**Superchlorinate**" means the addition of large quantities of chlorine-based chemicals to kill algae, destroy odors, or improve the ability to maintain a disinfectant residual. This process is different from

Hyperchlorination, which is a prescribed amount to achieve a specific CT value whereas Superchlorination is the raising of free chlorine levels for water quality maintenance.

"**Supplemental Treatment Systems**" means those disinfection processes or systems which are not required on an aquatic venue for health and safety reasons. They may be used to enhance overall system performance and improve water quality.

"**Theoretical Peak Occupancy**" means the anticipated peak number of bathers in an aquatic venue or the anticipated peak number of occupants of the decks of an aquatic facility. This is the lower limit of peak occupancy to be used for design purposes for determining services that support occupants. Theoretical peak occupancy is used to determine the number of showers. For aquatic venues, the theoretical peak occupancy is calculated around the type of water use or space:

- "**Agitated Water**" means an aquatic venue with mechanical means (aquatic features) to discharge, spray, or move the water's surface above and/or below the static water line of the aquatic venue so people are standing or playing vertically. Where there is no static water line, movement shall be considered above the deck plane.
- "**Flat Water**" means an aquatic venue in which the water line is static except for movement made by users usually as a horizontal use as in swimming. Diving spargers do not void the flat water definition.
- "**Hot Water**" means an aquatic venue with a water temperature over 90oF (32oC).
- "**Stadium Seating**" means an area of high-occupancy seating provided above the pool level for observation.

"**Trichloramine**" means a disinfection by-product formed when chlorine binds to nitrogenous waste in pool water to form an amine-containing compound with three chlorine atoms (NCl3). It is a known acute respiratory and ocular irritant. It has low solubility in water and is rapidly released into the air above pools where it can accumulate, particularly in indoor settings.

"**Trihalomethanes**" means chemical compounds in which three of the four hydrogen atoms of methane (CH4) are replaced by halogen atoms.

"**Turbidity**" means a measure of the clarity or cloudiness of water

"**Turnover**" or "**Turnover Rate**" means the period of time, usually expressed in hours, required to circulate a volume of water equal to the capacity of the aquatic venue.

"**Underwater Bench**" means a submerged seat with or without hydrotherapy jets.

"**Underwater Ledge**" or "**Underwater Toe Ledge**" means a continuous step in the pool wall that allows swimmers to rest by standing without treading water.

"**UV Transmissivity**" means the percentage measurement of ultraviolet light able to pass through a solution.

"Water Replenishment System" means a way to remove water from the pool as needed and replace with make-up water in order to maintain water quality.

"**Water Quality Testing Device**" means a product designed to measure the level of a parameter in water. A WQTD includes a device or method to provide a visual indication of a parameter level, and may include one or more reagents and accessory items.

"**Zero Depth Entry**" means a sloped entry into a pool from deck level into the interior of the pool as a means of access and egress.

3.0 Preoperational Requirements

3.1 Permits

3.1.1 No person shall operate a public pool who does not have a valid permit issued by the Division. Only a person who complies with the requirements of these Regulations shall be entitled to receive or retain such a permit.

3.1.2 An establishment's valid permit shall be posted in a location easily observed by the customer.

3.1.3 Permits shall not be transferable from person to person or from location to location.

3.1.4 When a public pool changes ownership, management firm, or lessee, both the facility and its operation shall be brought into full compliance with these Regulations prior to the issuance of a permit. A variance may be issued, as provided by these Regulations.

3.1.5 Establishments in compliance with these Regulations shall be issued a permit in accordance with Section 3.3. of these Regulations. Establishments that continue to operate without proper permits from the Division

or operate in violation of these Regulations will be subject to legal remedial actions and sanctions as provided by law.

3.2 Operating Permit - No person shall operate a pool without a valid permit from the Division and operating permits shall expire annually. The operating permit is not transferable if either the pool name or ownership changes. The operating permit shall be available for viewing by any patron or representative of the Division upon request

3.3 Issuance of Permits

3.3.1 Any person desiring to operate a public pool shall make written application for a permit. Such application shall be made on forms provided by the Division and shall include the name and address of each applicant, the location and type of the proposed establishment and the signature of each applicant.

3.3.2 Upon issuance of an application an applicant shall permit the Division to enter the proposed public pool to conduct an inspection.

3.3.3 The Division shall issue a permit to the applicant if its inspection reveals that the proposed public pool complies with these Regulations.

3.3.4 A permit is no longer valid upon occurrence of one or more of the following events:

3.3.4.1 the establishment is closed for a period of sixty (60) days or more;

3.3.4.2 a new owner, firm, or lessee takes possession of the establishment; or

3.3.4.3 the permit is revoked by the Division for violation of these Regulations.

3.3.5 Inactive Pools

3.3.5.1 Any pool which closes for twelve (12) or more continuous months shall:

3.3.5.1.1 be classified as inactive;

3.3.5.1.2 contact the Division for an Environmental Health Survey prior to re-opening; and

3.3.5.1.3 make whatever changes are deemed necessary to achieve compliance with the current Regulations.

3.3.5.2 If the inactive pool does not have its own recirculation/filtration/disinfection system, this shall be provided prior to receiving approval to reopen.

3.3.5.3 If there is no intention of reopening an inactive pool, it shall be properly abandoned (filled with dirt to ground level).

3.3.5.4 Outdoor inactive pools shall be equipped with a tightly fitting cover during mosquito breeding season (normally May through September).

3.4 Submission of Plans

3.4.1 No person shall construct, install, alter or replace a pool, auxiliary pool structure, or pool equipment and no person shall convert a private pool to a public pool until three (3) copies of plans and specifications have been submitted to the Division, and an Approval to Construct has been issued. Whenever it is discovered that any of the above have occurred or are occurring without such approval, the Director shall order the owner, operator or contractor to immediately close the pool if it is open or stop the work or conversion, and to submit plans and specifications to the Division. Any part of the unapproved work or pool that is not in compliance with these Regulations shall be removed, replaced or reconstructed in order to achieve compliance. Plans and specifications shall be legible and on paper no larger than 30" X 42" and shall include, but are not limited to the following information:

3.4.1.1 The facility name and the name and telephone number of a contact person at or near the site.

3.4.1.2 The name, mailing address and telephone number of the owner.

3.4.1.3 The name, mailing address and telephone number of the builder/contractor/engineer.

3.4.1.4 A map of the area (city, town, rural area, etc.) showing the project location and a scaled drawing of the site showing the pool location.

3.4.1.5 A scaled drawing showing an overhead view of the pool which includes the location of but is not limited to: all recirculation system fittings and piping, depth markings, steps/ladders, diving board, lifeguard stand, fillspout, safety line/bottom marking, lights (underwater and overhead), deck/walkway, fence, pump/filter room*, bathhouse facilities, food/beverage service facilities and any other pools (e.g. wading pool, spa pool). This drawing shall also specify the materials of construction for the pool and deck, and the color of the pool walls and floor. If wood is planned for the wet deck/walkway area the manufacturer of the wood shall be specified, and if the wood has

been treated by the manufacturer or will be treated by the installer, any preservatives, coatings, paints, etc. shall be specified.

3.4.1.6 *Either on this drawing or a separate sheet, a blown up scaled drawing of the pump/filter room showing all piping, equipment, fittings, and the flow pattern shall be provided.

3.4.1.7 A scaled drawing showing a profile view(s) of the pool, the pool depths and the relative elevation of the pump and filter. If a diving board is planned, an end view showing the required dimensions shall also be provided.

3.4.1.8 The following pool information: volume, water surface area, perimeter, filter type, filter surface area, design flow rate, recirculation pump capacity, total dynamic head (TDH) in feet [if not provided a minimum of sixty (60) TDH will be used], pool turnover time, and the size and type of all piping (i.e. ductile iron, copper, plastic).

3.4.1.9 Specifications for and drawings or pictures of all recirculation system components, including but not limited to: skimmers* or gutters; drains; inlets; recirculation pump with pump curve; rate of flow indicator with manufacturer's installation instructions; the type, brand and surface area in square feet of the filter(s)*; multiport valve*; the type, brand and capacity of automatic disinfection equipment*; heater (including proof of AGA or UL approval); portable vacuum equipment; and the chemical name of the disinfectant to be used. All specifications shall include the manufacturer's name and model #.

3.4.1.10 *Specifications shall include proof of NSF International listing, or approved equivalent. In the remainder of these Regulations, wherever there is a referral to NSF, it means NSF International or approved equivalent.

3.4.1.11 The source of potable water (i.e. their own on-site well or the name of the water supplier if the water comes from off-site), and the location and methods of disposal for sanitary waste, filter backwash water and pool water pumped to waste.

3.4.1.12 For new pools, the following signed statement from the owner in all three (3) sets of plans and specifications: "I hereby acknowledge that all items either listed or shown in these plans and specifications as not in contract (NIC), by others, or equivalent, are my responsibility. I also realize that this entire project must be completed in accordance with the approved plans and specifications, and all conditions listed in the Approval to Construct, prior to the issuance of an operating permit by the Division." This statement with signature may appear directly on the plan drawings.

3.4.1.13 For changes to existing pools, the following signed statement from the owner: "I hereby acknowledge that all items either listed or shown in these plans and specifications as not in contract (NIC), by others, or equivalent, are my responsibility. I also realize that this entire project must be completed in accordance with the approved plans and specifications, and all conditions listed in the Approval to Construct, prior to this pool receiving permission from the Division to reopen." This statement with signature may appear directly on the plan drawings.

3.4.2 Upon receipt of plans and specifications, the Division shall determine if the project is approved or disapproved and notify the responsible person within thirty (30) days. If disapproved, the reasons shall be specified. If approved, an Approval to Construct shall be issued which shall be valid for one (1) year. All construction shall be in accordance with the approved plans and specifications, and all conditions listed in the Approval to Construct. Prior to opening, the owner or operator of a new pool shall contact the Division for a pre-operational inspection in order to receive an operating permit. The Division reserves the right to reject plans and specifications from a pool builder/pool service company based upon evidence of malfeasance or non-feasance related to a previous Approval to Construct.

4.0 Inspections

4.1 Inspections and Right of Access

4.1.1 Site Inspection - Prior to the issuance of an Approval to Construct for the construction of a new outdoor pool, a site inspection shall be made by a representative of the Division in order to confirm that the pool location meets the requirements of Section 3.4. After a specific site is approved, any change of location shall require another site inspection.

4.1.2 Pre-Operational Plan Review Inspection – A pre-operational plan review inspection will occur prior to an Environmental Health Inspection, and will be conducted by representatives of the Division in order to

confirm that the pool meets the requirements of Section 3.4 for operation. This inspection will be posted in a location conspicuous to the public until an Environmental Health Inspection has been conducted.

4.1.3 Right of Entry and Environmental Health Inspection - Representatives of the Division shall have the right of immediate entry any time a pool is open, and in special situations, at any reasonable time, in order to perform any inspection to determine compliance with these Regulations. An inspection shall be conducted at least once per year at every active pool. These inspections may include any room or area associated with the pool operation. In addition, the representatives shall be permitted to examine any pertinent records.

4.2 Posting Inspection Results

4.2.1 The results of the most recent inspection of the public pool shall be posted in a location conspicuous to the public.

4.3 Division Personnel Competency Requirement

4.3.1 Division personnel performing environmental health/sanitary evaluations or complaint investigations of public pool shall meet the same requirements as specified for a qualified pool operator in Section 4.4 of these Regulations prior to assuming responsibilities for this program.

4.4 Qualified Operator

4.4.1 The operation of every pool shall be monitored by a qualified operator. This person may be part of the pool staff or from a private company and shall monitor the pool weekly or more as necessary to maintain compliance with these Regulations. Weekly visits shall be documented and available for review by the Division.

4.4.2 The qualified operator’s certificate issued by the Division, or a copy, shall be available for viewing by any patron or representative of the Division upon request.

4.4.3 The Division reserves the right to revoke the approval of any operator based upon evidence of malfeasance or non-feasance.

5.0 Operational Requirements

5.1 Requirements for the Premises

5.1.1 Location

5.1.1.1 Pools shall not be located in areas which are subject to contamination from dust, soot, fly ash, smoke, improper drainage, a high water table, or other undesirable substances.

5.1.1.2 For outdoor pools, any overhead wiring not inside an electrical conduit shall not pass over any part of the deck or an area within twenty (20) feet of the nearest edge of the pool. If the edge of a pool, extended upward vertically, is located within fifteen (15) feet of a building, building roof or balcony, a protective barrier may be required (discretion of the Division) to prevent diving from the building, roof or balcony.

5.1.2 Design

5.1.2.1 No limits are specified for the shape of pools, however, consideration shall be given to shape from the standpoint of safety and proper water circulation.

5.1.2.2 Pools shall be designed such that: there is even and complete water circulation throughout; safe, sanitary conditions can be maintained at all times; all bathers can be effectively monitored.

5.1.2.3 There shall be no protrusions, extensions, means of entanglement, or other obstructions in the pool which can cause entrapment or injury.

5.1.2.4 Underwater or overhead projections or obstructions except for handholds, steps, ladders, facilities for persons with disabilities or recirculation system fittings shall be prohibited, unless such construction can be justified by engineering design.

5.1.2.5 Underwater seat benches may be permitted in areas where the water depth is five (5) feet or less provided that: the maximum water depth over the seat bench is twenty-four (24) inches;

5.1.2.5.1 the seat bench is completely recessed; the outer edge of the seat bench shall be outlined in contrasting color by a solid marking line at least one (1) inch wide; and

5.1.2.5.2 The seat bench surface is slip resistant.

5.1.3 Construction

5.1.3.1 Pool walls and bottoms shall be constructed of concrete, fiberglass, metal or other nontoxic, impervious and structurally rigid materials approved by the Division, which will provide a watertight basin, smooth, easily cleanable surfaces and a finish without cracks.

5.1.3.2 Sand, earth or wood construction shall be prohibited. The walls and bottom shall be white or light in color for the purpose of insuring contrast to identify objects.

5.1.3.3 Corners formed by the intersections of walls or walls and floors shall be curved (radiused).

5.1.3.4 Surfaces within the pool intended to provide footing for patrons shall be designed to be slip resistant.

5.1.3.5 Offset or safety ledges shall be prohibited.

5.1.4 Walls

5.1.4.1 All pool walls shall be vertical for a minimum depth of two (2) feet six (6) inches in areas less than five (5) feet deep.

5.1.4.2 Exceptions may be made for irregularly shaped pools (e.g. zero depth entry, T, L or Z shapes) in the recessed areas out of the main swimming area.

5.1.5 Floor/Slope

5.1.5.1 All pool floors shall be sloped toward the outlet/drain and all slopes shall be constant.

5.1.5.2 The slope shall not exceed one (1) foot in twelve (12) feet (1':12') where the water depth is five (5) feet or less, shall not exceed one (1) foot in three (3) feet (1':3') where the depth is greater than five (5) feet.

5.1.5.3 Any pool having a transition from the slope in the shallow end to the deep end shall do so through a slope break starting at a depth not less than three and one half (3 1/2) feet and not greater than five (5) feet.

5.1.5.4 At least one (1) foot and not more than two (2) feet on the shallow side of the slope break directly under the safety line, the floor shall be marked with a line of contrasting color that is either: solid and at least two (2) inches wide or intermittent with sections that are at least four (4) inches by four (4) inches and not more than one (1) foot apart on centers.

5.1.6 Shallow End Minimum/Maximum Depth

5.1.6.1 With the exception of diving pools, all pools shall have a minimum depth in the shallow area of three (3) feet and a maximum depth of four (4) feet.

5.1.6.2 Exceptions may be made for irregularly shaped pools (e.g. T, L or Z shapes) with recessed areas out of the main swimming area.

5.1.7 Ladders and Steps

5.1.7.1 All pools shall have at least two entry/exit points (ladders or steps), one (1) for each end.

5.1.7.1.1 Wading and treatment pools may have one entry / exit point (ladders or steps).

5.1.7.2 Any pool with water deeper than five (5) feet shall have at least two (2) ladders in the deep end.

5.1.7.3 Any pool with a perimeter greater than two hundred twenty-five (225) feet shall have one (1) additional ladder or set of steps for each additional seventy-five (75) feet of perimeter or fraction thereof.

5.1.7.4 Ladders shall be constructed of corrosion resistant materials and shall be securely anchored into the pool deck or built into the pool wall.

5.1.7.4.1 All ladders shall have two (2) handrails and at least two (2) slip resistant treads which are at least one and one half (1 1/2) inches deep, with a uniform length of at least twelve (12) inches and a uniform spacing of at least seven (7) inches and not more than twelve (12) inches. The clearance between any ladder and the pool wall shall be at least three (3) inches and not more than six (6) inches.

5.1.7.4.2 Recessed ladders (built into the wall) shall have two (2) handrails and shall have a tread at least five (5) inches in depth, at least twelve (12) inches in width and the uppermost tread shall be within twelve inches of the pool coping top edge or the deck surface.

5.1.7.4.3 All treads shall slope toward the pool.

5.1.7.5 Where steps are provided, they shall either be recessed or located in a corner.

5.1.7.5.1 All steps shall have a minimum tread length of twenty-four (24) inches, a tread depth of at least ten (10) inches and a uniform height of not more than twelve (12) inches, with the exception of either the top or bottom tread which may vary + two (2) inches.

5.1.7.5.2 The top surface edge of each step shall be outlined in contrasting color by a line (painted, tile, etc.) at least one (1) inch wide. Each set of steps shall have at least one (1) handrail per seven (7) feet of tread length.

5.1.7.5.3 If the steps are less than four (4) feet wide the handrail shall be located at the side and if the steps are four (4) feet wide or wider, the handrail shall be located in the center.

5.1.7.5.4 The tread surface shall be slip resistant.

5.1.8 Decks and Walkways

5.1.8.1 Every pool shall have a continuous unobstructed deck at rim level around the entire perimeter.

5.1.8.2 The deck shall be no less than four (4) feet wide at any point, measured from the pool edge.

5.1.8.3 For outdoor pools, the area of the deck shall be at least equal to the area of the pool water surface, and for indoor pools, the area of the deck shall be at least equal to two thirds (2/3) of the pool water surface.

5.1.8.4 A minimum width of four (4) feet shall be provided behind lifeguard stands and the ladders/steps of all diving boards.

5.1.8.5 Walkways shall be provided from the deck to all pool sanitary and bathhouse facilities.

5.1.8.6 In computing the minimum deck area for adjacent pools, no area of the deck shall be considered as serving both pools.

5.1.9 Standing water

5.1.9.1 To prevent standing water, decks and walkways shall have a uniform slope of not less than one quarter (1/4) inch per foot and not more than one half (1/2) inch per foot, away from the pool and toward deck drains or areas where the water will have a free unobstructed flow to points of disposal.

5.1.9.2 The edge of the pool deck at its junction with the pool wall shall be constructed of bullnose coping, or some other acceptable material, which will provide an adequate hand hold around the entire pool perimeter and which is not more than twelve (12) inches above the normal water level.

5.1.10 Wet deck/walkway areas

5.1.10.1 Shall be constructed of concrete or other approved material which has an impervious slip resistant surface, can be easily cleaned and is installed such that there are no hazards to patrons or their bare feet (e.g. tripping, abrasions, splinters, etc.).

5.1.10.2 If expansion joints are provided, the joint shall be filled with a non-rigid material such as mastic which shall not protrude above the deck. Exposed wooden expansion joints shall be prohibited.

5.1.10.3 Carpeting shall be prohibited in wet deck/walkway areas.

5.1.11 Electrical and Lighting Requirements

5.1.11.1 All electrical wiring and equipment associated with the pool shall be in compliance with all appropriate state and local codes and the current edition of the National Electric Code.

5.1.11.2 For indoor pools and all bathhouse facilities, all overhead wiring which is not behind the wall or ceiling shall be inside an electrical conduit.

5.1.12 Artificial lighting

5.1.12.1 Shall be provided at all pools which do not have adequate natural lighting or which are to be used for evening/night swimming. The artificial lighting shall meet all of the following conditions:

5.1.12.1.1 Lighting shall be sufficient such that all areas of the pool, and either of the following are clearly visible without glare from the deck: the main drain(s) and all bottom markings; or a black disk six (6) inches in diameter superimposed upon a white field and placed on the bottom at the deepest point.

5.1.12.1.2 Outdoor pools shall provide at least thirty (30) foot candles and indoor pools shall provide at least fifty (50) foot candles of illumination at the water surface from natural and/or artificial lighting.

5.1.12.1.3 All overhead lights shall be covered with adequate shatter resistant shields and equipped with shatter resistant bulbs.

5.1.13 Hose Bibbs

5.1.13.1 A sufficient number of hose bibs shall be provided and located such that all parts of the deck area, the pump/filter room and the bathhouse facilities are easily reachable with a fifty (50) foot hose, without the hose passing over or through the pool water.

5.1.13.2 Hose bibs shall be located at the edge of the deck such that they do not constitute a tripping hazard and shall be equipped with vacuum breakers or other approved backflow prevention devices.

5.1.14 Trees, Sand Areas and Planted Areas

5.1.14.1 Trees at the pool site prior to construction shall not be permitted inside the pool fence. Sand areas and other nongrass / unsodded areas designed for bather access shall not be permitted inside the pool fence or room.

5.1.14.2 Provisions shall be made so that bathers returning to the pool deck from these areas are routed past a foot rinse shower.

5.1.14.3 Flower beds, shrubs and other similar planted areas may be permitted inside the pool fence or room if all of the following conditions are satisfied:

5.1.14.3.1 There shall be a separation distance of at least ten (10) feet between the edge of the pool and the edge of the planted area

5.1.14.3.2 The planted area shall be designed such that regular maintenance can be easily accomplished

5.1.14.3.3 All planted area drainage shall be conducted away from the pool in a manner that will not create muddy, hazardous, or objectionable conditions.

5.1.14.3.4 The planted area shall be designed to discourage patron entry.

5.1.15 Sanitary/Bathhouse Facilities

5.1.15.1 All pools shall have at least one (1) permanent water closet with lavatory for the lifeguard\attendant, which is accessible when the pool is open and is located contiguous to the pool deck or pool room.

5.1.15.2 All pools, with the exception of those where all patrons' sanitary/bathhouse facilities are within one thousand (1000) feet, shall provide the following permanent sanitary/bathhouse facilities located contiguous to the pool deck or pool room:

5.1.15.2.1 One fixture set for each sex when the pool(s) water surface area is up to 2000 square feet

5.1.15.2.2 Two fixture sets for each sex when the pool(s) water surface is over 2000 to 4000 square feet.

5.1.15.2.3 Three fixture sets for each sex when the pool(s) water surface is over 4000 to 6000 square feet.

5.1.15.2.4 Four fixture sets for each sex when the pool(s) water surface is over 6000 to 8000 square feet.

5.1.15.2.5 For pools greater than eight thousand (8000) square feet, one additional fixture set for each sex shall be provided for each additional four thousand (4000) square feet, or fraction thereof.

5.1.16 Bathhouse facilities

5.1.16.1 All sanitary and bathhouse facilities shall be indoors, enclosed to provide privacy, finished in light colors, well ventilated, in good working order and designed such that good sanitation can be maintained throughout at all times.

5.1.16.2 Floors shall constructed of concrete or other approved material, shall have a smooth slip resistant finish and shall be sloped to floor drains or points of drainage.

5.1.16.3 Carpeting shall be prohibited in all wet deck/walkway areas.

5.1.16.4 Floor and wall junctions shall be curved radius for easy cleaning.

5.1.16.5 Lighting shall provide at least thirty (30) foot candles of illumination at floor level.

5.1.16.6 All lavatories and showers shall have hot and cold water and shall be equipped with tempering valves which provide water at a temperature not exceeding 120 degrees F.

5.1.16.7 All bathhouses shall have soap (liquid recommended), single service towels or hot air dryers, toilet tissue, and waste baskets.

5.1.16.8 All plumbing shall be in compliance with the "State of Delaware Regulations Governing a Detailed Plumbing Code" and new installations shall be inspected and approved by the appropriate authority prior to use.

5.1.16.9 All bathhouse facilities shall be at the same elevation as the pool deck or at an elevation that is accessible with a ramp having a slope that shall not exceed one (1) inch per foot and should not exceed one (1) inch per twenty (20) inches.

5.1.17 Diaper Changing Station

5.1.17.1 All public pools allowing use by diaper-aged bathers shall, upon adoption of this Regulation, have at least one diaper changing station in each male and female bathhouse.

5.1.17.2 An adjacent hand wash sink shall be installed and operational

5.1.17.3 Diaper changing units shall conform to either of the following standards:

5.1.17.3.1 ASTM standard F2285-04: *Consumer Performance Standards for Commercial Diaper-Changing Stations*, or

5.1.17.3.2 The standards for diaper-changing surfaces in the most current version of *Caring for Our Children: National Health and Safety Performance Standards: Guidelines for Out-of-Home Child Care Programs*.

5.1.17.4 A covered, hands-free, plastic-lined trash receptacle or diaper pail shall be located directly adjacent to the diaper changing unit.

5.1.17.5 An EPA-registered disinfectant shall be provided for maintaining a clean and disinfected diaper changing unit surface before and after each use.

5.1.18 Foot Rinse Shower

5.1.18.1 All outdoor pools, except those where bathers must go through the bathhouse in order to reach the deck, shall have a foot rinse shower at each patron entry point.

5.1.18.2 Any indoor pool with direct bather access to an outdoor sand beach or other unsodded area shall also have a foot rinse shower at the access point. The foot rinse shower shall be located such that bathers must walk past the deck discharge area.

5.1.18.3 In order to prevent standing water, the deck discharge area shall slope toward a drain or an area where the water will have a free unobstructed flow to points of disposal.

5.1.18.4 The foot rinse shower should: be a shower head; be approximately twelve (12) to twenty four (24) inches above the deck; have an automatic shut off valve when released; and point toward the deck at approximately a forty five (45) degree angle.

5.1.18.5 Foot baths (standing water in which patrons rinse their feet) shall be prohibited.

5.1.19 Drinking Fountain

5.1.19.1 At least one (1) sanitary type (guarded angle jet) drinking fountain in good working order shall be provided.

5.1.19.2 The fountain shall be easily accessible and located inside the pool fence.

5.1.19.3 Common drinking cups shall be prohibited.

5.1.20 Food and Beverage Facilities

5.1.20.1 Food and beverage service facilities that are inside the pool fence or room shall not be located within twenty (20) feet of the pool edge. If permitting is required, all such facilities shall be licensed by the Division or the appropriate authority.

5.1.21 Starting Blocks

5.1.21.1 If starting blocks for competitive swimming events are installed, the water depth under the blocks shall be greater than five (5) feet.

5.1.22 Escutcheon Plates

5.1.22.1 All anchor bolts shall be covered by escutcheon plates.

5.1.23 Access for Persons with Disabilities

5.1.23.1 Access for disabled persons shall conform to ADA Standards as approved by the Department of Justice.

5.1.23.2 Facilities designed to accommodate persons with disabilities will be approved within the limits of sound engineering practice and nationally recognized standards.

5.1.24 Swimming Pool Slides

5.1.24.1 The installation of swimming pool slides shall be prohibited.

5.1.25 Diving Area/Diving Pool and Diving Board

5.1.25.1 The dimensions of the diving area/diving pool and the diving board shall be approved by plan review as per Section 3.4.1.

5.1.25.2 Pools with diving boards higher than three (3) meters, and/or pools with diving platforms shall be constructed in accordance with the standards of the FINA, NCAA and USD and these devices shall be labeled "COMPETITIVE DIVING ONLY."

5.1.25.3 Diving boards shall have guardrails on both sides which are at least thirty (30) inches high and extend from the back end of the board to at least one (1) foot past the pool edge. Diving boards which are greater than one (1) meter high shall have handrails on both sides of the ladder/steps and the spacing between them exceed twelve (12) inches.

5.1.25.4 All diving boards shall be level and shall have slip resistant surfaces. All supports, steps, and railings shall be made of material which is of sufficient strength to handle the anticipated load. Trampoline type diving facilities and rope drops shall be prohibited. With the exception of competitive diving, any adjustable fulcrum diving board shall have the fulcrum locked in the maximum forward position.

5.1.25.5 Any diving board in existence on the effective date of these Regulations (April 15, 1990) that cannot comply with the requirements of this Section by being shortened and/or lowered, should be removed. Also, in order for the replacement of any diving board stand, or the replacement of any diving board stand and diving board to be approved, the pool shall be in compliance with the requirements of this Section.

6.0 Recirculation System – General Requirements

6.1 Recirculation system.

6.1.1 The recirculation system shall consist of, but is not limited to: a pump, a flow measuring device, a removable strainer with spare basket (not required on vacuum filters), a filter with at least one (1) pressure gauge, an automatic disinfectant feeder, piping, deep end floor outlet/drains, a perimeter overflow gutter or surface skimmers, return inlets, valves and other necessary equipment.

6.1.2 The recirculation system shall be designed to provide the maximum allowable turnover times:

6.1.2.1 Maximum Allowable Turnover Times

6.1.2.1.1 Swimming Pools maximum turnover 6 hours or less

6.1.2.1.2 Wading Pools maximum turnover 1 hour or less and shall have a secondary disinfection system

6.1.2.1.3 Spray Pads maximum turnover 1 hour or less and shall have a secondary disinfection system

6.1.2.1.4 Treatment pools maximum turnover 1 hour or less and shall have a secondary disinfection system

6.1.2.1.5 Spa Pool maximum turnover 15 minutes or less

6.1.3 In the pump/filter area, each suction and discharge line shall have a manual control valve capable of regulating flow or shutting off flow completely

6.1.3.1 If the required flow rate is provided collectively by multiple pumps, all of these pumps shall operate by a single control switch.

6.1.3.2 The difference between the minimum required flow rate in gallons per minute (gpm) (based on the type of pool) and the maximum allowable flow rate in gpm (based on the type of filtration) shall be at least ten (10) gpm.

6.2 Recirculation Pump

6.2.1 All recirculation pumps shall be of adequate capacity to provide the required turnover rate, a sufficient backwash rate and sufficient suction for any vacuum fittings.

6.2.2 If the pump or any suction side piping is located above the pool water level, the pump shall be self-priming.

6.2.3 All recirculation pumps shall be equipped with the necessary piping, valves etc. such that pool water can be pumped directly to waste.

6.2.3.1 This line shall terminate above any drain entry point by a distance of at least twice its diameter.

6.2.3.2 For the purpose of confirming or determining the flow rate, all recirculation pumps shall be equipped with pressure gauges on both the suction and pressure side the pump.

6.2.4 Rate of Flow Indicator

6.2.4.1 At least one (1) rate of flow indicator reading in gpm shall be installed after filtration on the pool return line.

6.2.4.2 The indicator shall be easily accessible for viewing, in proper working condition when the pool is open, sized such that the design flow rate is in the mid range of the indicator, and capable of measuring at least fifty (50) per cent more than the design flow rate.

6.2.4.3 The clearance upstream and downstream from the indicator shall comply with the manufacturer's specifications.

6.2.4.4 In lieu of the above, a pre-set flow control valve with a direct mounted meter kit and conversion chart may be used.

6.2.5 Floor Outlets/Drains

6.2.5.1 All pools shall be provided with at least two (2) floor outlets/drains at the deepest point which: are connected to the recirculation system through the same line; are at least six (6) feet apart at centers (for special designs, as far apart as possible or on different planes); are equipped with a flow control valve(s) near the recirculation pump; have an antivortex cover or a twelve inch by twelve inch (12" x 12") grate or larger; and can completely drain the pool.

6.2.5.2 All drain outlet pipes shall be recessed within the fitting or in a pit and equipped with a cover which can only be removed with a Phillips screwdriver or a special tool. The width and length of slot openings in the cover shall not exceed one half (1/2) inch and one (1) inch respectively.

6.2.6 Perimeter Overflow Gutters and Surface Skimmers

6.2.6.1 All pools shall have either perimeter overflow gutters or surface skimmers (not recommended for pools with a water surface area greater than 2500 square feet) which:

6.2.6.1.1 are capable of conducting one hundred (100) percent of the required flow rate;

6.2.6.1.2 are connected to the recirculation system; have flow control valves near the recirculation pump;

6.2.6.1.3 and effectively remove any floating material.

6.2.6.2 If perimeter overflow gutters are used, they shall be continuous around the pool with a uniform level rim which is not more than twelve (12) inches below the deck and which will provide a suitable handhold.

6.2.6.3 Gutters shall be designed so that the channel is easily accessible for cleaning and presents no entanglement hazard to bathers.

6.2.6.4 If the gutters are recessed, the access opening shall be at least four (4) inches.

6.2.6.5 Gutter drains shall be provided at uniform intervals not to exceed fifteen (15) feet and the gutter shall slope sufficiently to these drains.

6.2.6.6 The design shall ensure that the recirculation pump receive a continuous supply of water at all times either by adequate surge capacity within the gutters or a surge/balancing tank.

6.2.6.7 Any perimeter overflow gutter with a submerged protruding edge, shall have a solid marking line of contrasting color which is at least one (1) inch wide on the top surface edge of the gutter.

6.2.6.8 If surface skimmers are used, they shall be NSF listed and at least two (2) shall be provided.

6.2.6.8.1 For pools that are greater than one thousand (1000) square feet of water surface, one (1) additional skimmer shall be provided for each additional (500) square feet of pool water surface or fraction thereof.

6.2.6.8.2 Skimmers shall be optimally located around the pool perimeter and the recirculation system shall be designed such that the flow through each skimmer is at least twenty (20) gpm.

6.2.6.8.3 All skimmers shall have individual flow controls devices, an equalizer line (except WP pools), an easily removable, cleanable basket or screen and a cover.

6.2.7 Inlets

6.2.7.1 All pools shall have at least four (4) inlets, either on the side walls or on the floor, which are connected to the recirculation system.

6.2.7.1.1 Side wall inlets, with the exception of those built into a gutter, shall discharge at a depth of at least twelve (12) inches below the normal water level.

6.2.7.1.2 The distance between side wall or bottom inlets, measured along the wall or floor surface, shall not exceed twenty (20) feet and the spacing between any floor inlet and the side wall, measured along the surface, shall not exceed ten (10) feet.

6.2.7.1.3 When wall inlets are used, an inlet shall be provided within five (5) feet of each corner and one (1) in each recessed step area.

6.2.7.1.4 The inlets, either by adjustability or by design, shall provide the necessary flow to maintain the required disinfectant residual and chemical quality evenly throughout the pool.

6.2.8 Vacuuming

6.2.8.1 All pools shall have the capability of vacuuming the bottom either through a skimmer, a separate vacuum fitting or a portable vacuum system.

6.2.8.1.1 If a portable vacuum system must be used, it shall be stored on-site when the pool is open.

6.2.8.1.1.1 Vacuuming through a portable vacuum system that is connected to the potable water supply shall be prohibited.

6.2.8.1.1.2 In line and portable vacuum pumps must be equipped with a removable strainer with a spare basket.

6.2.9 Piping

6.2.9.1 Recirculation system piping shall be made of non-toxic material.

6.2.9.2 All plastic piping shall be: labeled with the manufacturer's name and the NSF logo for potable water; listed in the current NSF "Listing of Plastic Materials, Pipe, Fittings, and Appurtenances for Potable Water and Wastewater, or approved equivalent;" and used/installed in accordance with the manufacturer's specifications.

6.2.10 Multiport Valves

6.2.10.1 All multiport valves shall be NSF listed.

6.2.11 Pool Water Heaters

6.2.11.1 All gas heaters shall be design-certified by the American Gas Association (AGA) and shall display a rating data plate and the AGA seal.

6.2.11.2 All electric heaters shall be Underwriters Laboratories (UL) approved and shall display the UL seal.

6.2.11.3 All heaters shall have sufficient piping and valves to permit isolation and removal of the heater from the system.

6.2.12 Equipment Access, Freezing Protection and Drainage

6.2.12.1 Easy access shall be provided to all parts of the recirculation system that must be inspected or serviced.

6.2.12.2 All piping and equipment that is subject to freezing shall be provided with an adequate means of draining.

6.2.12.3 At least thirty (30) foot candles of illumination shall be provided around all equipment.

6.2.12.4 In order to prevent standing water, all equipment rooms shall be adequately graded toward floor drains or areas where the water will have a free unobstructed flow to points of disposal.

7.0 Filtration System

7.1 General Requirements - Each pool shall have its own filtration system.

7.1.1 All filters shall bear the manufacturer's name, the model #, the surface area and the filter shall be NSF listed. All filtration systems shall be sized such that the maximum allowable filtration rates are not exceeded and shall be operated such that the passage of unfiltered water will be prevented.

7.1.2 The filter effluent water shall have a turbidity of one (1) NTU or less.

7.1.3 All filters shall be capable of being totally drained through a manual valve or by disconnecting a union, and all filtration surfaces shall be accessible for inspection, maintenance or replacement.

7.1.4 Filters that require backwashing shall have a pressure gauge(s) for determining the time to backwash, and a site glass for observing the backwash water clarity.

7.1.5 The backwash discharge line shall terminate above the drain entry point by a distance of at least twice its diameter.

7.1.6 All pressure filters shall be equipped with manual or automatic air release valves, or shall be self-purging.

7.1.7 If a manual air release valve is present, specific instructions explaining its use shall be provided and shall be conspicuously posted on or near the filter.

7.1.8 In multiple filter installations filter piping shall be valved such that each filter can be isolated for repairs, while other filters remain in service.

7.2 Rapid Sand Filters

7.2.1 All media shall meet all of the specifications of the filter manufacturer.

7.2.2 In multiple filter installations filter piping shall be valved such that each filter can be individually backwashed.

7.2.3 The filtration rate for rapid sand filters shall not exceed three (3) gpm per square foot of filter area, or the NSF listed flow rate, whichever is less.

7.3 High Rate Sand Filters

7.3.1 All media shall meet all of the specifications of the filter manufacturer.

7.3.2 The filtration rate for high rate sand filters shall not exceed twenty (20) gpm per square foot of filter area, or the NSF listed flow rate, whichever is less.

7.4 Diatomaceous Earth Filters

7.4.1 If diatomaceous earth filters are equipped with a pressure type separation tank, there shall be no manual valves on the effluent line, and the following statement or equivalent shall be conspicuously posted on the top of the separation tank and at the pump control switch “Do not start the pump without opening the manual air release on the separation tank and checking to make sure that the top of the separation tank is securely attached.”

7.4.2 Any manufacturer’s instructions for the proper use of this separation tank shall display either on the top of the separation tank or on the wall nearby. The filtration rate for diatomaceous earth filters shall not exceed two (2) gpm per square foot of filter area, or the NSF listed flow rate, whichever is less.

7.4.3 Diatomaceous earth filters that have an NSF listing for slurry feed shall not exceed a filtration rate of 2.5 gpm per square foot of filter area.

7.5 Cartridge Filters

7.5.1 All pools with cartridge filtration systems shall have at least one (1) spare cartridge for each cartridge in use, which is clean and ready for installation, along with at least one (1) vat which is capable of submerging all of the cartridges from one filter vessel.

7.5.2 Pools with modular media cartridge filtration shall have at least one (1) spare cartridge for each type of cartridge in use, which is clean and ready for installation.

7.5.3 When cartridges become clogged to the extent that cleaning does not restore them, or they become damaged, they shall be discarded.

7.5.4 The filtration rate for cartridge filters shall not exceed three hundred seventy-five one thousandths (.375) gpm per square foot of filter area, or the NSF design listed flow rate, whichever is less.

8.0 Potable Water Supply, Pool Water Quality/testing, Chemicals and Wastewater

8.1 Potable Water Supply

8.1.1 All pools shall have a water supply which:

8.1.1.1 is approved by the Division;

8.1.1.2 is in compliance with the requirements of the “State of Delaware Regulations Governing Public Drinking Water Systems;”

8.1.1.3 and is of adequate capacity to meet peak demands while maintaining at least twenty five (25) pounds per square inch (psi) and not more than one hundred (100) psi at all points in the system.

8.1.2 All drinking fountains shall be in good working order.

8.1.3 Cross connections between the potable water supply and the pool water shall be prohibited.

8.1.4 Hose bibs shall be located such that they do not constitute a tripping hazard and shall be equipped with vacuum breakers or other backflow prevention devices approved by the Division.

8.2 Fillspout

8.2.1 All pools shall have a permanent fillspout which shall be air gapped at least two (2) pipe diameters above the pool rim, or the rim of any vessel or pipe that connects to the pool.

8.2.2 In order to minimize the possibility of creating a tripping hazard, all fillspouts that are on the deck shall be located immediately next to one of the pool ladders, or another approved location.

8.2.3 The portion of the water line passing through a concrete deck shall either be non-plastic piping or shall be inside a metal sleeve.

8.2.4 All pools that have a fillspout that is a potential tripping hazard, shall take whatever steps are appropriate to eliminate the hazard.

8.3 Clarity

8.3.1 The water in all pools shall be sufficiently clear and the lighting shall be sufficient such that all areas of the pool, and either of the following are clearly visible from the deck:

8.3.1.1 the main drain(s) and all bottom markings;

8.3.1.2 or a black disk six (6) inches in diameter superimposed upon a white field and placed on the bottom at the deepest point.

8.3.2 The turbidity of the pool water shall be five tenths (.5) NTU or less.

8.4 Bacteriological Quality

8.4.1 If samples are taken for the heterotrophic plate count test [standard plate count (SPC)], the result shall be less than (<) two hundred (200) colonies per milliliter.

8.4.2 If samples are taken for the total coliform test, when the membrane filtration (MF) technique is used, the result shall be less than (<) one (1) colony per one hundred (100) milliliters of sample,

8.4.3 when the multiple tube fermentation or most probable number (MPN) method is used, none of the confirmed portions shall show the presence of the coliform group [result reported as less than (<) 2.2] and when the presence/absence test is used, the result shall be absent.

8.4.4 Samples shall be taken by, or required by the Division whenever it is deemed necessary. For the purpose of determining compliance with this Section, samples may be considered only if they have been analyzed by the Division or by another approved laboratory.

8.5 Chemical Quality

8.5.1 The chemical quality of pool water shall not cause any irritation to the eyes or skin of bathers and shall be in compliance with the requirements of the “State of Delaware Regulations Governing Public Drinking Water Systems.”

8.5.1.1 The pH shall not be less than 7.2 or greater than 7.8.

8.5.1.2 Any chemical added directly or indirectly to a pool shall be approved by the Division, registered with the U. S. Environmental Protection Agency, used strictly in accordance with the manufacturer’s directions and properly labeled in accordance with the Hazardous Chemical Information Act 16 **Del.C.** Ch. 24.

8.5.2 Any pool with a documented history of pH level violations shall have an automatic pH adjustment chemical feeder that:

8.5.2.1 is the NSF listed; is approved by the Division;

8.5.2.2 has approved anti-siphon protection; and is wired such that it feeds only when the recirculation pump runs (shall be done on all pools which are in existence on the effective date of these Regulations when either the chemical feeder or recirculation pump are replaced).

8.5.3 The manual addition of approved non-disinfectant chemicals shall be permitted only in special situations (e.g. trying to achieve water balance), which require that the pool be closed.

8.5.3.1 After this manual addition has been completed, the pool shall remain closed for at least one (1) turnover and until such time as the chemical is thoroughly and evenly dispersed throughout the pool.

8.6 Disinfection

8.6.1 All pools shall be disinfected with an approved halogen that imparts an easily measured residual and is fed through an automatic feeder that:

8.6.1.1 is NSF listed; is approved by the Division; has approved anti-siphon protection;

8.6.1.2 is capable of providing a dosage of at least ten (10) ppm for outdoor pools and a dosage of five (5) ppm for indoor pools (shall be done on an existing pool when the disinfectant feeder is replaced);

8.6.1.3 and is wired such that it feeds only when the recirculation pump runs (shall be done on all pools which are in existence on the effective date of these Regulations when either the disinfectant feeder or recirculation pump are replaced).

8.6.2 A flow-through (erosion) feeder shall feed only the disinfectant(s) specified by the manufacturer. All feeders, with the exception of flow-through feeders, shall add the disinfectant downstream from the filter and heater.

8.6.3 All pool disinfectants shall be registered with the U.S. Environmental Protection Agency and approved by the Division.

8.6.4 Manual addition of an approved halogen disinfectant shall be permitted only in special situations (e.g. superchlorination), which require that the pool be closed.

8.6.4.1 After this manual addition has been completed, the pool shall remain closed: for at least one (1) turnover and until such time as the chemical is thoroughly and evenly dispersed throughout the pool;

8.6.4.1.1 and the automatic feeder is operating properly if it was not at the time of the manual addition.

8.6.5 Use of gas chlorine shall be prohibited.

8.6.6 Use of stabilized chlorine (cyanuric acid) in indoor pools shall be prohibited.

8.6.7 In order for any non-halogen to be approved as a stand alone disinfectant, satisfactory performance shall be demonstrated during an NSF evaluation.

8.6.8 All pools shall be disinfected in accordance with the following requirements:

8.6.8.1 Chlorine

8.6.8.1.1 Pools not using Cyanuric Acid minimum 1.0 PPM Free Available Chlorine required

8.6.8.1.2 Pools using Cyanuric Acid minimum 2.0 PPM Free Available Chlorine Required

8.6.8.1.3 Spas minimum 3.0 PPM Free Available Chlorine

8.6.8.1.4 Recommended maximum is 10.0 PPM Free Available Chlorine

8.6.8.2 Bromine

8.6.8.2.1 Pools minimum 3.0 PPM Bromine

8.6.8.2.2 Spas minimum 4.0 PPM Bromine

8.6.8.2.3 Cyanuric Acid should not exceed 100 PPM

8.6.9 Secondary Disinfection Systems

8.6.9.1 General Requirements

8.6.10 ANSI Listing and Labeling - Secondary Disinfection Systems shall be listed and labeled to ANSI/NSF 50 by an ANSI-accredited certification organization approved by the Division.

8.6.10.1 Required Facilities

8.6.10.1.1 The new construction or substantial alteration of the following public pools shall be required to use a secondary disinfection system after adoption of this regulation:

8.6.10.1.2 Pools designed primarily for children under 5 years old, such as

8.6.10.1.2.1 Wading pools

8.6.10.1.2.2 Splash pads with no standing water

8.6.10.1.2.3 Treatment pools

8.6.10.2 3-log Inactivation

8.6.10.2.1 Secondary disinfection system shall be designed to achieve a minimum 3-log (99.9%) reduction in the number of infective Cryptosporidium parvum oocysts per pass through the secondary disinfection system.

8.6.10.3 Installation

8.6.10.3.1 The secondary disinfection system shall be located in the treatment loop (post filtration) and treat a portion (up to 100%) of the filtration flow prior to return of the water to the pool or feature.

8.6.10.3.2 Minimum Flow Rate Calculation The flow rate (Q) through the secondary disinfection system shall be determined based upon the total volume of the pool or feature (V) and a prescribed dilution time (T) for theoretically reducing the number of assumed infective Cryptosporidium (108 oocysts from an initial total number of 100 million) oocysts to a concentration of one oocysts/100 mL.

8.6.10.3.3 Equation Accounting for a 3 log (99.9%) reduction of infective Cryptosporidium oocysts through the secondary disinfection system with each pass, the secondary disinfection system flow rate

8.6.10.3.3.1 (Q) shall be: Q = V x {[14.8 – ln (V)] / (60 x T)}, where:

8.6.10.3.3.1.1 Q = secondary disinfection system flow rate (gpm)

8.6.10.3.3.1.2 V = Total water volume of the pool or feature, including surge tanks, piping, equipment, etc. (gals)

8.6.10.3.3.1.3 T = Dilution time (hrs.)

8.6.10.3.4 Time for Dilution Reduction The dilution time shall be the lesser of nine hours or 75% of the uninterrupted time a pool is closed in a 24 hour period.

8.6.10.3.5 Flow Rate Measurements where a secondary disinfection system is installed, a means shall be installed to confirm the required flow rate to maintain a minimum 3 log (99.9%) reduction of infective Cryptosporidium oocysts at the minimum flow rate.

8.6.11 Water Testing Equipment and Sampling Frequency

8.6.11.1 All pools shall have approved testing equipment for pH, disinfectant residual, alkalinity, and calcium hardness.

8.6.11.2 For disinfectant residual, the DPD (Diethyl-P-Phenylene Diamine) method, or any other method in the current edition of Standard Methods for the Examination of Water and Wastewater, shall be used.

8.6.11.3 If the disinfectant is chlorine, the test kit shall be capable of measuring both free and total chlorine residual.

8.6.11.4 For pH, the phenol red colorimetric method, or any other method in the current edition of Standard Methods for the Examination of Water and Wastewater, shall be used and the test kit shall have a range of at least 6.8-8.2.

8.6.11.5 In addition to these parameters, pools that use a stabilized halogen shall have approved cyanuric acid testing equipment.

8.6.11.6 All chemical test kit reagents shall be dated when received, and shall be replaced just prior to the start of each outdoor pool season, and at least yearly for indoor pools, with the exception of phenol red which shall be replaced at least every six (6) months.

8.6.11.6.1 pH and disinfectant residual shall be measured daily, prior to the pool opening, and as often as necessary while the pool is open (recommended every one (1) to two (2) hours) in order to ensure the proper levels.

8.6.11.6.2 Alkalinity, calcium hardness and cyanuric acid (if applicable) shall be measured after each addition of makeup water and at least weekly.

8.6.12 All sample results shall be recorded along with the date, time and sample location (area of the pool).

8.6.13 Records of sample results shall be kept at the pool for at least one (1) year, shall be available for viewing by any representative of the Division during an inspection and shall be submitted to the Division upon request.

8.6.14 If any other substance is added to the pool on a regular basis (e.g. copper/silver from a supplemental disinfection system), the Division may require testing to ensure that the concentrations do not exceed acceptable levels.

8.7 Pool Water Level and Recirculation System

8.7.1 When the pool is open, the pool water level shall be maintained at an elevation suitable for continuous flow into the surface skimmers or intermittent flow into the gutter as bathers enter.

8.7.2 The recirculation system shall operate continuously (24 hours per day), with the exception of the automatic disinfectant feeder when the disinfectant level approaches or exceeds the upper recommended level.

8.7.3 All pools shall have the capability of pumping water to waste either directly or via filter backwash.

8.7.4 The Division may require a dye test to ensure that the recirculation system is providing even and complete recirculation throughout the pool.

8.8 Chemical Storage

8.8.1 All chemicals, including test kits, shall be stored in accordance with the storage recommendations on the manufacturer’s label and the MSDS, and they shall be stored in areas that are not easily accessible to bathers or other unauthorized personnel.

8.8.2 All pools shall keep adequate quantities of chemicals on hand such that shortages are not experienced.

8.8.3 “NO SMOKING” signs shall be conspicuously posted in all chemical storage areas.

8.9 Wastewater Disposal

8.9.1 Cross connections between the pool recirculation system and any wastewater system, including the filter backwash drain, shall be prohibited.

8.9.2 The backwash discharge line shall terminate above the drain entry point by a distance of at least twice its diameter.

9.0 Operation, Maintenance, General Sanitation, Personnel, Supervision and Safety

9.1 Operation and Maintenance

9.1.1 All pools, their premises, and appurtenances, shall be operated and maintained at all times with regard to the safety of bathers and employees.

9.1.2 All plumbing shall be properly installed and maintained.

9.1.3 During an electrical storm, use of any pool (indoor or outdoor) shall be prohibited.

9.1.4 Alcoholic beverages shall be prohibited in the pool and on the pool deck.

9.1.5 Food and beverages shall be prohibited in the pool and within four (4) feet of the pool edge.

9.1.6 Glass or other breakable containers, utensils, etc. shall be prohibited within the pool fence, pool room or bathhouse facilities.

9.1.7 Pool walls and floors shall be refinished when safe sanitary conditions cannot be maintained.

9.1.8 Any deck/walkway areas that are hazardous to patrons or their bare feet (e.g. tripping, abrasions, splinters, etc.) or do not properly drain, shall be repaired or replaced such that the hazard or drainage problem is eliminated.

9.1.9 Any electrical hazard shall be eliminated.

9.1.10 All pools which have a slope break, shall install a line of contrasting color one (1) to two (2) feet on the shallow side of the break directly under the safety line that is either: solid and at least two (2) inches wide or intermittent with sections that are at least four (4) inches by four (4) inches and not more than one (1) foot apart on centers.

9.1.11 All pools which have steps or benches, shall install a solid marking line of contrasting color which is at least one (1) inch wide on the top surface edge of each pool step or bench.

9.1.12 All pools which have any type of overflow gutter or a safety ledge with a submerged protruding edge, shall install a solid marking line of contrasting color which is at least one (1) inch wide on the top surface edge of the gutter.

9.2 General Sanitation and Sanitary Facilities

9.2.1 All pools and related facilities shall be maintained in a clean sanitary condition.

9.2.2 The pool floor, walls, deck, walkways and bathhouse facilities shall be cleaned and disinfected with an approved disinfectant as often as necessary in order to maintain an environment which is free of sediment, dirt, algae, grass/weeds and foreign objects.

9.2.3 All garbage shall be stored in containers which are sufficient in number and properly covered so as not to attract vermin.

9.2.4 Effective control measures shall be utilized to minimize or eliminate the presence of rodents, flies, roaches or other vermin.

9.2.5 All pools shall have at least one (1) permanent or portable water closet for the lifeguard/attendant, which is accessible when the pool is open and is located such that the path of travel from the nearest pool entrance/exit does not exceed five hundred (500) feet.

9.2.5.1 If a portable water closet is provided, it shall be properly vented, designed to exclude flies, equipped with a self-closing door, provided with toilet tissue and maintained in a clean sanitary condition and be located contiguous to the pool deck or pool room.

9.3 Animals

9.3.1 With the exception of guide / service animals for persons with disabilities, animals shall be prohibited within the pool fence, pool room or bathhouse facilities.

9.4 Bathing Suits

9.4.1 It is recommended that all bathers should wear bathing suits. A bathing suit is a garment designed for that purpose which covers the buttocks (male and female) and breasts (female).

9.4.2 If anyone who is not toilet trained is going to use a pool, the operator shall take whatever steps are necessary to ensure that fecal material does not enter the water (e.g. requiring the use of a swimsuit diaper).

9.4.3 Should fecal material enter any pool, the pool shall be closed immediately and the operator shall follow Division procedures for reopening.

9.5 Bather Health

9.5.1 Any person with evidence of an open sore, a bandaged wound or diarrhea shall be prohibited from entering the pool water. Any person suspected of being under the influence of alcohol or drugs shall be prohibited from entering the pool.

9.6 Lifeguard

9.6.1 Unless exempted by 16 **Del.C.**, each pool that is greater than four (4) feet deep (any portion) shall have a lifeguard on duty when the pool is open.

9.6.2 If a lifeguard who is working alone must leave poolside, for whatever reason, he/she shall take whatever steps are necessary to ensure that no one remains in or enters the water in his/her absence.

9.6.3 Copies of training/certification documentation for all lifeguards currently employed by the pool's management shall be present at the pool when it is open.

9.6.4 No person shall serve as a lifeguard unless he or she meets all of the following training requirements and can produce the appropriate documentation upon request of the Division:

9.6.4.1 Current certification in National YMCA Lifeguarding, or American Red Cross Basic Lifeguarding, Ellis and Associates International Lifeguard Training Program, Aquatic Resource Service Associates Aquatic Rescue Training or approved equivalent.

9.6.4.2 Current certification in American Red Cross, American Heart Association, or National Safety Council (NSC) Cardiopulmonary Resuscitation (CPR), or approved equivalent, that includes training in: one (1) person adult CPR, child CPR and infant CPR.

9.6.4.3 Current certification in American Red Cross Standard First Aid, or approved equivalent, that includes training in: obstructed airway, artificial breathing, control of bleeding, and treatment for shock.

9.7 Attendant

9.7.1 If none of the pools at a facility require a lifeguard based on depth, each pool, with the exception of those exempted by 16 **Del.C.**, shall have an attendant on duty when the pool is open.

9.7.2 If an attendant who is working alone must leave poolside, for whatever reason, he/she shall take whatever steps are necessary to ensure that no one remains in or enters the water in his/her absence.

9.7.3 In facilities with a spa pool(s) only, if the attendant is not in direct view of the pool, he/she shall be located such that the poolside alarm required by Section 9.19. can be easily heard.

9.7.4 Copies of training/certification documentation for all attendants currently employed by the pool's management shall be present at the pool when it is open.

9.7.5 No person shall serve as an attendant unless he or she meets all of the following training requirements and can produce the appropriate documentation upon request of the Division:

9.7.5.1 Current certification in American Red Cross, American Heart Association or NSC Cardiopulmonary Resuscitation (CPR), or approved equivalent that includes training in: one person adult CPR, child CPR and infant CPR.

9.7.5.2 Current certification in American Red Cross Standard First Aid, or approved equivalent, that includes training in: obstructed airway, artificial breathing, control of bleeding, and treatment for shock.

9.8 Lifeguard Warning Sign

9.8.1 Any exempted pool or any pool with a maximum depth of four (4) feet that does not have a lifeguard or attendant on duty shall conspicuously post the following sign at the pool entrance(s) and at least one (1) other location inside the pool room/fence:

9.8.1.1 "WARNING NO LIFEGUARD ON DUTY" in letters at least four (4) inches high along with "CHILDREN UNDER THE AGE OF SIXTEEN (16) SHOULD BE ACCOMPANIED BY A PARENT

OR GUARDIAN" in letters at least one (1) inch high. (NOTE -A guardian is an adult designated by a parent who is responsible for the children and their behavior)

9.9 Lifeguard Stand

9.9.1 All pools with a water surface area greater than two thousand (2000) square feet shall have at least one (1) lifeguard stand.

9.9.2 The lifeguard stand shall be at poolside, however, no part of the stand shall extend past the edge of the pool.

9.10 Unsupervised Solo Bathing - Unsupervised solo bathing shall be prohibited at all pools with the exception of those exempted by 16 **Del.C.**

9.11 Injury, Resuscitation or Death

9.11.1 The Division shall be notified within twenty-four (24) hours of any incident at a pool which:

9.11.1.1 requires referral to a hospital, doctor or other facility for medical attention;

9.11.1.2 requires resuscitation;

9.11.1.3 or results in death.

9.11.2 The notification shall be followed up by a written report within thirty (30) days which contains all pertinent details of the incident.

9.12 Pool Security

9.12.1 All entrances to indoor pools shall be equipped with locks and each entrance shall be locked when the pool is closed.

9.12.2 All outdoor pools shall be enclosed by a fence which provides a barrier that is at least four (4) feet high everywhere [six (6) feet recommended], measured from ground level outside the fence.

9.12.3 All pool fences shall be equipped with a locking gate which shall be locked when the pool is closed.

9.13 Depth Markings

9.13.1 With the exception of wading pools and spa pools, the water depth of all pools, in feet to the nearest one half (1/2) foot, shall be plainly marked at or above the water surface on the vertical pool wall, and on the coping or deck within eighteen (18) inches of the pool edge.

9.13.1.1 For pools with open gutters, the depth marking required on the vertical pool wall may be located on the wall of the room for indoor pools, and on the fence for outdoor pools.

9.13.2 These pairs of depth markings shall be located at the points of minimum depth, maximum depth, intermediate depths at no more than two (2) foot depth increments, breaks in slope and on each end of the pool.

9.13.3 All depth markings shall be at least four (4) inches high, of a contrasting color and spaced no more than twenty-five (25) feet apart on the pool perimeter.

9.13.4 Markings on the pool walls shall be positioned to be read from the water. Markings on the deck shall be positioned to be read while standing on the deck and facing the pool and shall be slip resistant.

9.14 Safety Line

9.14.1 Whenever non-swimmers are in the water, all pools that have a slope break shall have a safety line located at least one (1) foot and not more than two (2) feet on the shallow side of the slope break, directly above the line on the bottom required by Section 5.1.

9.14.2 The safety line shall have clearly visible colored floats spaced not more than five (5) feet apart. The safety line shall be of sufficient size and strength to provide a handhold which will support any bather who is in need of help and the connections to the pool wall shall be recessed.

9.15 Safety Equipment - All pools shall have the following safety equipment which shall be easily accessible:

9.15.1 A telephone,

9.15.1.1 Each facility shall have a functional telephone or other communication device that is hard wired and capable of directly dialing 911 or function as the emergency notification system.

9.15.1.2 The telephone or communication system or device shall be conspicuously provided and accessible to aquatic venue users such that it can be reached immediately.

9.15.1.3 Alternate functional systems, devices, or communication processes are allowed with AHJ approval in situations when a hardwired telephone is not logistically sound, and an alternate means of communication is available.

9.15.1.4 A permanent sign providing emergency dialing directions and the aquatic facility address shall be posted and maintained at the emergency telephone, system or device.

9.15.2 A first aid kit

9.15.2.1 An adequate supply of first aid supplies shall be continuously stocked and include, at a minimum, as follows:

9.15.2.1.1 A First Aid Guide,

9.15.2.1.2 Absorbent compress,

9.15.2.1.3 Adhesive bandages,

9.15.2.1.4 Adhesive tape,

9.15.2.1.5 Sterile pads,

9.15.2.1.6 Disposable gloves,

9.15.2.1.7 Scissors,

9.15.2.1.8 Elastic wrap,

9.15.2.1.9 Two Emergency blankets,

9.15.2.1.10 Resuscitation mask with one-way valve, and

9.15.2.1.11 Blood borne pathogen spill kit.

9.15.2.2 The public pool shall have designated locations for emergency and first aid equipment.

9.15.3 Signage – shall be provided at the aquatic facility or each aquatic venue, as necessary, which clearly identifies the following:

9.15.3.1 Sign Indicating First Aid Location

9.15.3.1.1 First aid location(s), and

9.15.3.1.2 Emergency telephone(s) or approved communication system or device.

9.15.3.2 Emergency Dialing Instructions

9.15.3.2.1 A permanent sign providing emergency dialing directions and the aquatic facility address shall be posted and maintained at the emergency telephone, system or device.

9.15.3.3 Management Contact Info

9.15.3.3.1 A permanent sign shall be conspicuously posted and maintained displaying contact information for emergency personnel and aquatic facility management.

9.15.3.4 Hours of Operation

9.15.3.4.1 A sign shall be posted stating the following:

9.15.3.4.1.1 The operating hours of the aquatic facility, and

9.15.3.4.1.2 Unauthorized use of the aquatic facility outside of these hours is prohibited.

9.15.4 In addition to the above, all pools without a lifeguard on duty (those exempted by 16 **Del.C.**) shall also be equipped with the following safety equipment which shall be and easily accessible:

9.15.4.1 One (1) or more lightweight poles at least twelve (12) and not more than fifteen (15) feet long, and equipped with a shepherd's hook.

9.15.4.2 One ring buoy at least eighteen (18) to twenty-four (24) inches in diameter attached to at least fifty (50) feet of rope.

9.15.4.3 In addition to the above, all pools with a lifeguard on duty shall also be equipped with the following safety equipment that shall be made easily accessible:

9.15.4.4 One rigid backboard (long board) with at least three (3) attached ties/straps which is compatible for transport in the Delaware State Police MED-E-VAC helicopter, meets the design requirements of Emergency Medical Services, and is approved by the Division. Six (6) attached ties/straps are recommended.

9.15.4.4.1 If a pool, because of size, will not accommodate a long board being placed under a bather, a reduced backboard (short board) with at least one (1) tie/strap may be substituted.

9.15.4.4.2 Current specification requirements for backboards are available upon request from the Division. (NOTE - This is not required for exempted pools that operate without a lifeguard).

9.15.4.5 In place of a ring buoy, at least one (1) rescue tube for each lifeguard on duty.

9.16 Diving

9.16.1 With the exception of official competition or when there is qualified instruction, diving shall be prohibited into water that is five (5) feet deep or less.

9.16.2 At Swimming and water slide flume pools, all areas where the water is five (5) feet deep or less shall be clearly labeled “No Diving” on the coping (edge of the deck).

9.16.3 The “No Diving” markings shall be: of a contrasting color; easily readable;, slip resistant and at least four (4) inches high consisting of the words “No Diving,” the words “No Diving” in combination with the picture (international) sign (diver inside a red circle with a red line across), or other approved markings.

9.16.4 The number of markings shall be at least equal to the number of depth markings within the “No Diving Area”.

9.17 Shallow End Starting Blocks

9.17.1 Shallow end starting blocks (water depth that is five (5) feet or less) shall be removed when there is no official competition, instruction or practice.

9.17.2 Shallow end starting blocks that are not removable shall be labeled “starting blocks shall be used only during official competition or when there is qualified instruction.”

9.18 Wading Pool Supervision

9.18.1 It is recommended that all children using a wading pool be supervised by a responsible person who is at least sixteen (16) years of age.

9.19 Attendant Alarm

9.19.1 Any spa pool, with the exception of those at facilities exempted by 16 **Del.C.**, which is located such that it is not in direct view of the attendant shall have, in the immediate vicinity of the pool, a clearly labeled alarm device that can be activated when a bather is in trouble and is easily heard throughout the area or building.

9.19.2 This alarm shall produce a distinctly different sound than that of the high temperature alarm.

9.20 Spa Pool Warning Sign

9.20.1 A clearly visible sign shall be mounted at the entrance of each Spa pool facility or adjacent to each spa pool and shall include, but is not limited to, the following:

9.20.1.1 All bathers should take a shower prior to entering.

9.20.1.2 Enter and exit slowly and cautiously.

9.20.1.3 Unsupervised solo bathing is prohibited.

9.20.1.4 Use by anyone under five (5) years of age is prohibited unless there is documented written permission from their physician.

9.20.1.5 Elderly persons and anyone with heart disease, diabetes, high or low blood pressure, or anyone under a physician’s care should not enter without consulting with their physician.

9.20.1.6 Pregnant women should not enter unless the temperature is less than 102°F.

9.20.1.7 Hot water immersion while under the influence of, anticoagulants, antihistamines, vasoconstrictors, vasodilators, stimulants, hypnotics, narcotics or tranquilizers may be hazardous to your health and is prohibited.

9.20.1.8 Observe a fifteen (15) minute time limit, then shower, cool down and return if you wish. Longer exposures to this water may result in nausea, dizziness or fainting.

9.20.1.9 Do not submerge to the bottom of the pool, hair may become entangled in bottom outlet. Long hair should be tied in a knot or a bun in order to keep it out of the water.

9.20.1.10 No body lotions or oils are permitted.

9.21 Spa Pool Timer Switch

9.21.1 All Spa pools shall have a clearly labeled reversible aeration/jetting system timer switch with a fifteen (15) minute time limit located in the pool area but such that it cannot be reset from within the pool.

9.22 Spa Pool Temperature/High Temperature Prevention System

9.22.1 The water temperature in spa pools shall not exceed 104°F.

9.22.2 All spa pools shall have a thermometer that is measuring the current water temperature.

9.22.3 If the thermometer is in the pool, it shall be non-glass and tethered.

9.22.4 All spa pools shall be equipped with an approved system that will ensure that the water does not exceed 104°F (e.g. a high temperature alarm, an automatic heater shut off device, etc.) The alarm shall produce a distinctly different sound than that of the attendant alarm.

9.22.5 If there is a documented history of high temperature violations, the high temperature prevention system shall be replaced/upgraded.

9.22.6 The Division may test the high temperature prevention system to ensure proper operation.

9.23 Spa Pool Clock

9.23.1 A clock which is easily readable from the spa pool shall be provided.

9.24 Water Slide Flume-Head of the Flume Personnel

9.24.1 At least one person who meets the training requirements of Section 9.7. shall be stationed at the head of the flume to supervise and control the start of each slider, the spacing of sliders and the monitoring of the downward progress of each slider.

9.25 Water Slide Flume-Splash Pool Attendant

9.25.1 At least one attendant shall be stationed near the end of the flume/run out to supervise and control the sliders as they arrive.

9.25.2 Additional attendants shall be required at the discretion of the Division.

9.26 Ventilation

9.26.1 All bathhouse rooms, equipment rooms, chemical storage rooms and indoor pool rooms shall be provided with adequate natural or mechanical ventilation such that satisfactory indoor air quality is maintained.

9.27 Cartridge Filtration

9.27.1 All pools with cartridge filtration systems shall have at least one (1) spare cartridge for each cartridge in use, which is clean and ready for installation, along with at least one (1) vat which is capable of submerging all of the cartridges from one filter vessel.

9.27.2 Pools with modular media cartridge filtration shall have at least one (1) spare cartridge for each type of cartridge in use, which is clean and ready for installation.

9.27.3 When cartridges become clogged to the extent that cleaning does not restore them, or they become damaged, they shall be discarded.

9.28 Fecal/Vomit/Blood Contamination Response

9.28.1 Contamination Response Plan

9.28.1.1 All public pools shall have a Contamination Response Plan for responding to formed-stool contamination, diarrheal-stool contamination, vomit contamination, and contamination involving blood.

9.28.2 Contamination Training

9.28.2.1 The Contamination Response Plan shall include procedures for response and cleanup, provisions for training staff in these procedures, and a list of equipment and supplies for clean-up.

9.28.2.2 A minimum of one person on-site while the public pool is open for use shall be:

9.28.2.2.1 Trained in the procedures for response to formed-stool contamination, diarrheal contamination, vomit contamination, and blood contamination; and

9.28.2.2.2 Trained in Personal Protective Equipment and other OSHA measures including the Bloodborne Pathogens Standard 29 CFR 1910.1030 to minimize exposure to bodily fluids that may be encountered as employees in an aquatic environment.

9.28.2.3 Informed - Staff shall be informed of any updates to the response plan.

9.28.2.4 Equipment and Supply Verification - The availability of equipment and supplies for remediation procedures shall be verified by the qualified operator at least weekly.

9.28.2.5 Plan Review - The response plan shall be reviewed at least annually and updated as necessary.

9.28.2.6 Plan Availability - The response plan shall be kept on site and available for viewing by the Division.

9.28.3 Aquatic Venue Water Contamination Response

9.28.3.1 Closure - In the event of a fecal or vomit contamination in a public pool, the trained staff shall immediately close the public pool to swimmers until remediation procedures are complete.

9.28.3.2 Closure Includes - This closure shall include the affected Public pool and other public pool venues that share the same recirculation system.

9.28.3.3 Physical Removal - Contaminating material shall be removed (e.g., using a net, scoop, or bucket) and disposed of in a sanitary manner.

9.28.3.4 Clean / Disinfect Net or Scoop

9.28.3.4.1 Fecal or vomit contamination of the item used to remove the contamination (e.g., the net or bucket) shall be removed by thorough cleaning followed by disinfection (e.g., after cleaning, leave the net, scoop, or bucket immersed in the pool during the disinfection procedure prescribed for formed-stool, diarrheal-stool, or vomit contamination, as appropriate).

9.28.3.5 No Vacuum Cleaners

9.28.3.5.1 Aquatic vacuum cleaners shall not be used for removal of contamination from the water or adjacent surfaces unless vacuum waste is discharged to a sanitary sewer and the vacuum equipment can be adequately disinfected.

9.28.3.6 Treated - Public Pool water that has been contaminated by feces or vomit shall be treated as follows:

9.28.3.6.1 Check to ensure that the water's pH is 7.5 or lower and adjust if necessary;

9.28.3.6.2 Verify and maintain water temperature at 77°F (25°C) or higher;

9.28.3.6.3 Operate the filtration/recirculation system while the POOL reaches and maintains the proper free CHLORINE concentration during the remediation process;

9.28.3.6.3.1 Test the chlorine residual at multiple sampling points to ensure the proper free chlorine concentration is achieved throughout the pool for the entire disinfection time; and

9.28.3.6.3.2 Use only non-stabilized chlorine products to raise the free chlorine levels during the remediation.

9.28.4 Aquatic Venue Water Contamination Disinfection

9.28.4.1 Formed-Stool Contamination

9.28.4.1.1 Formed-stool contaminated water shall have the free chlorine residual checked and the free chlorine residual raised to 2.0 mg/L (if less than 2.0 mg/L) and maintained for at least 25 minutes (or an equivalent time and concentration to reach the CT value) before reopening the public pool.

9.28.4.1.2 Pools Containing Chlorine Stabilizers - In public pool water that contains cyanuric acid or a stabilized chlorine product, water shall be treated by doubling the inactivation time required.

9.28.4.1.3 Measurement of Inactivation Time - Measurement of the inactivation time required shall start when the public pool reaches the intended free Chlorine level.

9.28.4.2 Diarrheal-Stool Contamination

9.28.4.2.1 Diarrheal-stool contaminated water shall:

9.28.4.2.1.1 Check the free chlorine residual and then raise the free chlorine residual to 20.0 mg/L and maintain for at least 12.75 hours (or an equivalent time and concentration to reach the CT value before reopening the public pool, or

9.28.4.2.1.2 Circulate the water through a secondary disinfection system to theoretically reduce the number of Cryptosporidium oocysts in the public pool below one oocyst/100 mL.

9.28.4.3 Pools Containing Chlorine Stabilizers

9.28.4.3.1 In AQUATIC VENUE water that contains cyanuric acid or a stabilized chlorine product, water shall be treated by:

9.28.4.3.1.1 Lowering the pH to 6.5, raising the free chlorine residual to 40 mg/L using a non-stabilized chlorine product, and maintaining at 40 mg/L for at least 30 hours or an equivalent time and concentration needed to reach the CT value. (Measurement of the inactivation time required shall start when the aquatic venue reaches the intended free chlorine level.) or;

9.28.4.3.1.2 Circulating the water through a secondary disinfection system to theoretically reduce the number of Cryptosporidium oocyst in the public pool below one oocyst/100 mL or;

9.28.4.3.1.3 Draining the AQUATIC VENUE completely.

9.28.5 Vomit-Contamination

9.28.5.1 Vomit-contaminated water shall have the free chlorine residual checked and the free chlorine residual raised to 2.0 mg/L (if less than 2.0 mg/L) and maintained for at least 25 minutes (or an equivalent time and concentration to reach the CT VALUE) before reopening the public pool.

9.28.5.2 Pools Containing Chlorine Stabilizers

9.28.5.2.1 In public pool water that contains cyanuric acid or a stabilized chlorine product, water shall be treated by doubling the inactivation time

9.28.5.3 Measurement of the Inactivation Time

9.28.5.3.1 Measurement of the inactivation time required shall start when the public pool reaches the intended free chlorine level.

9.28.6 Blood-Contamination

9.28.6.1 Blood contamination of a properly maintained public pool's water does not pose a public health risk to swimmers.

9.28.6.2 Operators Choose Treatment Method

9.28.6.2.1 Operators may choose whether or not to close the public pool and treat as a formed stool contamination to satisfy patron concerns.

9.28.7 Procedures for Brominated Pools

9.28.7.1 Formed-stool, diarrheal-stool, or vomit-contaminated water in a brominated public pool shall have chlorine added to the public pool in an amount that will increase the free chlorine residual to the level specified for the specific type of contamination for the specified time.

9.28.7.2 Bromine Residual

9.28.7.2.1 The bromine residual shall be adjusted if necessary before reopening the public pool.

9.28.8 Surface Contamination Cleaning and Disinfection

9.28.8.1 Limit Access - If a bodily fluid, such as feces, vomit, or blood, has contaminated a surface in an public pool, facility staff shall limit access to the affected area until remediation procedures have been completed.

9.28.8.2 Clean Surfaces - Before disinfection, all visible contaminant shall be cleaned and removed with disposable cleaning products effective with regard to type of contaminant present, type of surface to be cleaned, and the location within the facility.

9.28.8.3 Contaminant Removal and Disposal - contaminant removed by cleaning shall be disposed of in a sanitary manner or as required by law.

9.28.8.4 Disinfect Surface - Contaminated surfaces shall be disinfected with one of the following disinfection solution:

9.28.8.4.1 1:10 dilution of fresh household bleach with water; or

9.28.8.4.2 An equivalent EPA Registered disinfectant that has been approved for body fluids disinfection.

9.28.8.5 Soak - The disinfectant shall be left to soak on the affected area for a minimum of 20 minutes or as otherwise indicated on the disinfectant label directions.

9.28.8.6 Remove - Disinfectant shall be removed by cleaning and shall be disposed of in a sanitary manner or as required by the Division.

10.0 Wading Pools

10.1 General Requirements

10.1.1 Except as otherwise noted previously and in this Section, wading pools shall comply with all other requirements of these Regulations.

10.2 Location

10.2.1 Wading pools which are installed in conjunction with a pool shall be located adjacent to the shallowest area of the pool and shall be at least ten (10) feet away.

10.3 Floor/Slope

10.3.1 The slope of a wading pool floor shall not exceed six (6) inches in ten (10) feet (6"/10') and the floor shall be slip resistant.

10.4 Fencing

10.4.1 All wading pools which are indoors or within the confines of another pool fence shall be surrounded by a fence which is at least three (3) feet high and is equipped with a gate.

10.5 Recirculation System

10.5.1 The wading pool recirculation system shall be capable of providing a minimum of twenty-four (24) turnovers of the pool volume per day [one (1) every hour] against the maximum head.

10.6 Perimeter Overflow Gutters and Surface Skimmers

10.6.1 All wading pools shall be equipped with either a perimeter overflow gutter or at least one skimmer which is connected to the recirculation system and effectively remove any floating material.

10.6.2 For wading pools that are greater than five hundred (500) square feet of water surface, one (1) additional skimmer shall be provided for each additional (500) square feet of pool water surface or fraction thereof.

10.7 Inlets

10.7.1 All wading pools shall have at least two (2) inlets and in wading pools where only one (1) skimmer is required, the inlets shall be located such that they direct water toward the skimmer.

10.8 Water Replacement

10.8.1 The entire wading pool water volume should be replaced on at least a weekly basis either by normal backwashing or by pumping directly to waste.

11.0 Spa Pools

11.1 General Requirements

11.1.1 Except as otherwise noted previously and in this Section, spa pools shall comply with all other requirements of these Regulations.

11.2 Location

11.2.1 A spa pool shall be at least eight (8) feet away from any other pool.

11.3 Decks and Walkways

11.3.1 A deck at least four (4) feet wide shall be provided around at least fifty (50) per cent of the perimeter of a spa pool.

11.3.2 If the spa pool is free standing and not higher than thirty six (36) inches, the deck may be at floor level.

11.4 Steps and Benches

11.4.1 Spa pools which are greater than twenty-four (24) inches deep shall have at least one (1) step with at least one (1) handrail and all steps shall be located at a point where the deck is at least four (4) feet wide.

11.4.2 The step tread shall be at least ten (10) inches deep and at least twelve (12) inches wide, and the step riser shall be at least seven (7) inches and not be more than twelve (12) inches high.

11.4.3 When the bottom tread also serves as the bench, the height above the pool floor shall not exceed fourteen (14) inches.

11.4.4 The first and last risers need not be uniform. Intermediate risers, however, shall be uniform in height.

11.4.5 Benches that are installed in spa pools shall be permanent and the depth of water over the bench shall not exceed twenty-four (24) inches.

11.4.5.1 The top surface edge of all benches and steps shall be outlined in contrasting color by a marking line at least one (1) inch wide.

11.5 Recirculation System

11.5.1 The spa pool recirculation system shall be completely separate from the air/water jet system and shall be capable of providing a minimum of ninety-six (96) turnovers of the pool volume per day [one (1) every fifteen (15) minutes] against the maximum head.

11.6 Perimeter Overflow Gutters and Surface Skimmers

11.6.1 All spa pools shall be equipped with either a perimeter overflow gutter or at least one skimmer which is connected to the recirculation system and effectively removes any floating material.

11.7 Inlets

11.7.1 All spa pools shall have at least two (2) inlets which are connected to the recirculation system and in spa pools where only one (1) skimmer is required, the inlets shall be located such that they direct water toward the skimmer

11.8 Air Jetting System

11.8.1 All air jetting (blower) systems shall have a raised loop or some other approved method to prevent water backup that could cause electrical shock hazards.

12.0 Water Slide Flume

12.1 General Requirements

12.1.1 Except as otherwise noted previously and in this Section, water slide flumes shall comply with all other requirements of these Regulations.

12.2 Splash Pool Design and Depth

12.2.1 In order to facilitate prompt, safe exiting by bathers, all splash pools shall have either a set of steps along the entire length of the exit side (side opposite the flume terminus), or a floor that slopes upward to meet the water surface.

12.2.2 The steps or upward sloping floor shall have one (1) handrail per flume or one (1) handrail every seven (7) feet, whichever is greater.

12.2.3 Ladders shall be prohibited.

12.2.4 The depth of the splash pool at the flume end shall be at least three (3) feet and not more than four (4) feet, and this depth shall be maintained for at least twenty (20) feet in front of the flume end.

12.2.5 The floor slope shall not exceed one (1) foot in seven (7) feet and all slopes shall be constant.

12.3 Flume End

12.3.1 Flumes shall terminate either at a depth of at least six (6) inches below the splash pool operating water level, or not more than two (2) inches above the splash pool operating water level, provided the flume is level for at least the last ten (10) feet.

12.3.2 The distance between the side of any flume end and the side of the splash pool shall be at least five (5) feet.

12.3.3 The distance between the sides of adjacent flumes shall be at least six (6) feet and adjacent flumes utilizing the same splash pool shall be parallel at the ends.

12.4 Decks and Walkways

12.4.1 A deck at least ten (10) feet in width shall be provided along the entire exit side of the splash pool.

12.4.2 All walks and steps from the exit side to the top of the flume shall be: at least four (4) feet wide;

12.4.3 constructed of concrete or other slip resistant material approved by the Division;

12.4.4 smooth, easily cleanable and properly maintained;

12.4.5 and separated from any flume by a physical barrier such that people on the walks or steps cannot reach the flumes.

12.5 Recirculation System

12.5.1 The water slide flume recirculation system shall be capable of providing a minimum of twenty-four (24) turnovers of the splash pool and pump reservoir volume per day [one (1) every hour] against the maximum head.

12.6 Pump Reservoirs

12.6.1 Pump reservoirs shall be separate from the splash pool, shall be made of concrete or other impervious material with a smooth slip-resistant finish, and shall be connected to the splash pool by a weir.

12.6.2 The minimum pump reservoir volume shall be equal to five (5) minutes of the combined flow rate in gpm of all water slide flume pumps. Pump reservoirs shall be accessible only to authorized individuals.

12.6.3 Access decks shall be provided.

12.6.4 The pump intake(s) shall be located in the pump reservoir and shall be designed to allow cleaning without danger of operator entrapment.

12.6.5 The pump reservoir shall have a minimum of one (1) main drain with separate piping and valve to the filtration system.

12.6.6 Pumps shall have check valves on all discharge lines.

12.7 Perimeter Overflow Gutters and Surface Skimmers

12.7.1 All splash pools and pump reservoirs shall have either perimeter overflow gutters or surface skimmers which effectively remove any floating material and are connected to the recirculation system.

12.7.2 Where perimeter overflow gutters are used, they are not required along the weirs or under the flume end.

12.7.3 Where surface skimmers are used, one (1) shall be in the splash pool and one (1) shall be in the pump reservoir.

12.7.4 Where an odd number of surface skimmers are required they shall be positioned appropriately based on the size of the splash pool and pump reservoir.

12.8 Disinfection

12.8.1 Water slide flume pools may be permitted to manually add a halogen disinfectant, in addition to the automatic feeder, if it can be done somewhere other than the flume or splash pool.

12.9 Special Purpose Pools

12.9.1 General Requirements

12.9.1.1 The Division shall require compliance with any Sections of these Regulations which are deemed necessary, to assure the health and safety of special purpose pool bathers.

12.9.1.2 The approval of special purpose pools shall be approved by plan review as per Section 3.4.

13.0 Spray Pads

13.1 General Requirements

13.1.1 Except as otherwise noted previously and in this Section, spray pads shall comply with all other requirements of these Regulations

13.2 Surface

13.2.1 Shall have a slip-resistant and easily cleanable surface.

13.2.2 Any manufactured surfacing shall be deemed suitable by the manufacturer for aquatic and chlorinated environments.

13.3 Slope – Spray Pads shall be properly sloped so that only water from the pad flows back to the collection tank.

13.3.1 Adjacent areas adjacent to the spray pad shall be sloped away from the collection drains.

13.3.2 The slope of the Spray Pad shall be sufficient to prevent standing water from collecting on the pad.

13.4 Drains

13.4.1 The size, number and locations of the spray pad drains shall be determined and specified so as to assure water does not accumulate on the pad.

14.0 Administrative Action

14.1 Operating Without a Permit

14.1.1 If a pool is found operating without a valid permit, the Division shall order immediate closure.

14.1.2 The closure shall be effective upon receipt of a written notice by the person in charge of the pool.

14.1.3 The pool shall remain closed until submission, review and approval of plans, followed by a sanitary survey confirming compliance with these Regulations and issuance of an operating permit.

14.1.4 A conspicuous, colored placard shall be prominently displayed at all entrances of the pool which has failed to obtain a valid permit.

14.2 Suspension of a Permit

14.2.1 If conditions exist at a pool which presents an imminent health hazard to the public, the Director may suspend the operating permit and order immediate closure without a hearing upon written notice.

14.2.2 The suspension shall be effective upon receipt of written notice by the person in charge and a suspension statement on the Environmental Health Report constitutes a written notice.

14.2.3 The person in charge shall yield the permit to the Division.

14.2.4 A suspension notice shall be issued for any of the following conditions:

14.2.4.1 The clarity of the water is not in compliance with the provisions of Section 8.3.

14.2.4.2 The bacteriological quality of the swimming pool water is not in compliance with the provisions of Section 8.6.

14.2.4.3 The pH of the swimming pool water is less than 7.2 or greater than 7.8.

14.2.4.4 The automatic disinfection system is not functioning properly, or, there is no automatic disinfection system or disinfectant present.

14.2.4.5 The free chlorine residual is less than the level specified in Section 8.6.

14.2.4.6 The bromine residual is less than the level specified in Section 8.6.

14.2.4.7 The cyanuric acid level is greater than one hundred (100) ppm.

14.2.4.8 The recirculation pump is not operating or not present.

14.2.4.9 The filter is not operating or not present.

14.2.4.10 There is no qualified lifeguard or attendant on duty (unless exempted by 16 **Del.C.**)

14.2.4.11 The water temperature exceeds 104°F in a spa pool.

14.2.4.12 There is a bare electrical wire or other obvious electrical hazard present.

14.2.4.13 The lighting is not in compliance with the provisions of Section 5.1.11. and because of this, the following are not clearly visible without glare from the deck:

14.2.4.13.1 the main drain(s) and all bottom markings;

14.2.4.13.2 or a black disk six (6) inches in diameter superimposed upon a white field and placed on the bottom at the deepest point.

14.2.4.13.3 The bottom drain cover/grate is missing, broken, or not properly secured.

14.2.4.14 An authorized representative of the Division is denied immediate access to the pool pursuant to Section 4.1.3.

14.2.4.15 Fecal material is discharged into the pool water.

14.2.4.16 The facility fails to provide a qualified operator pursuant to Section 4.4.

14.2.4.17 There is any other condition, or combination of conditions which may endanger the health, safety, or welfare of the bathers.

14.2.4.18 If the person in charge, the pool operator or the lifeguard/attendant determines that any of the above conditions exist, they shall immediately close the pool.

14.2.5 The Division shall be notified of the closure and the pool shall remain closed until the condition is satisfactorily corrected.

14.2.6 The person in charge of the pool may request in writing, to the Division at any time during the suspension, an Environmental Health Report for the purpose of showing that the imminent health hazard no longer exists.

14.2.7 The person in charge of the pool may also request, in writing, to the Division at any time during the suspension, an administrative hearing to challenge the findings of the Environmental Health Report that resulted in the pool closure.

14.2.8 When the Division determines that the imminent health hazard no longer exists, the suspension shall be terminated and the permit returned.

14.2.9 If the Division determines that the imminent health hazard has not been corrected and that the hazard still exists, the suspension remains in force pending a hearing and the Division may recommend that the permit be revoked.

15.0 Hearings

15.1 If the Division receives a request for a hearing, the Division shall schedule an administrative hearing within ten (10) days of the request. The purpose of the hearing is to determine if the suspension should be lifted.

15.1.1 Serious Violations, Repeat Violations and General Unsanitary Conditions

15.1.1.1 If serious violations, repeat violations, or general unsanitary conditions exist, the Division may issue and properly serve due notice, by certified mail or by hand delivery, of the intention of the Division to suspend or revoke the permit of a pool.

15.1.1.2 The Division shall, not suspend or revoke a permit of a pool for serious or repeated violations that do not present an imminent health hazard, without having first issued and properly served such notice of intent to suspend or revoke.

15.1.2 Within thirty (30) days of the date of such notice of intent to suspend or revoke, the permit holder may submit to the Division a written request for an administrative hearing.

15.1.3 The suspension or revocation shall commence upon expiration of the notice of intent, unless within thirty (30) days of the date of such notice, the Division receives from the permit holder a written request for an administrative hearing.

15.1.4 If the permit holder makes a timely request for an administrative hearing, the suspension or revocation shall be stayed pending the results of the hearing.

15.1.5 A conspicuous, colored placard shall be prominently displayed at all entrances of a pool whose permit stands suspended or revoked.

15.2 Pool Permit Holder Right to an Administrative Hearing

15.2.1 Upon due notice that the Division intends to suspend or revoke the permit of a pool as indicated in 15.2, or for other reasons to protect public health, the permit holder may submit to the Division, within thirty (30) days of the date of such notice of intent, a written request for an administrative hearing.

15.2.2 When an administrative hearing is scheduled, the permit holder of the pool shall be informed at least five (5) days prior to the hearing of the place, time and date of the hearing and the specific charges against the pool.

15.2.3 Notification of the hearing shall be by certified mail or by hand delivery. Failure of the permit holder to be present for an administrative hearing shall result in automatic suspension of the permit and recommendation for revocation.

15.3 Records of Administrative Hearings

15.3.1 A written report of the hearing decision shall be furnished by the Division to the permit holder of the pool.

16.0 Enforcement and Interpretation

16.1 No provisions of Sections of these Regulations shall be applied retroactively, or interpreted to require reconstruction, alteration or replacement of a pool, or any part of a pool, which has been approved by the Division and which has been installed or is under construction.

16.2 If, however, a pool or any part of a pool is reconstructed, altered or replaced, or if a private pool is converted to a public pool after the effective date of these Regulations, this conversion, reconstruction, alteration or replacement shall meet all of the provisions of these Regulations.

17.0 Penalty

Any person who neglects or fails to comply with the requirements of these Regulations shall be subject to the provisions of 16 **Del.C.** §107, and shall be fined not less than $100 and not more than $1000, together with the costs, unless otherwise provided by law.

18.0 Repeal, Date of Effect and Amendments

18.1 All Regulations or parts of Regulations in conflict with these Regulations are hereby repealed, and these Regulations shall be in full force and effect on the date of adoption by the Secretary, DHSS. The Secretary, DHSS may propose changes to the Regulations herein established and shall hold at least one public hearing on the proposed changes.

18.2 At least thirty (30) days in advance of the public hearing, notice of proposed changes shall be published in at least two newspapers of general circulation in the State. Notice shall include a brief synopsis of the changes to be made, information on when and where the proposed changes may be reviewed by the public, the procedure for submitting comments, and the time, date and location of the public hearing.

18.3 A hearing officer shall be appointed by the Secretary, DHSS, and a record shall be kept of the hearing.

Appendix A

All chemicals in pool water affect the tendency of the water to be corrosive or to deposit a calcium carbonate scale. It is possible to control these two (2) conditions through the use of the Langelier Index (L.I.). The index was developed in 1936 by Professor W.F. Langelier at the University of California (Berkeley). It expresses the relationship between pH, alkalinity, calcium hardness and temperature. The resulting number (formula method - below) or position on the nomograph (nomograph method - Appendix B) indicates whether the pool water is chemically “balanced”. In theory, pool water that is “balanced” will neither corrode nor deposit a scale.

LANGELIER INDEX/WATER BALANCE FORMULA METHOD

“Balanced” pool water has an L.I. between -0.3 and +0.3. An L.I. that is greater negatively than -0.3 indicates a corrosive water and an L.I. greater than +0.3 indicates a water that will deposit scale. After determining the pool water pH, alkalinity, calcium hardness and temperature, find the alkalinity factor (AF), calcium hardness factor (CHF) and temperature factor (TF) using the table below. Use the pH and these three (3) factors in the following equation:

L.I. = pH + AF + CHF + TF - 12.1

FACTORS NECESSARY TO CALCULATE THE L.I.

Alkalinity Factor		Hardness Factor		Temp. (F) Factor	Calcium
5 ppm	0.7	5 ppm	0.3	32	0.1
25 ppm	1.4	25 ppm	1.0	37	0.1
50 ppm	1.7	50 ppm	1.3	46	0.2
75 ppm	1.9	75 ppm	1.5	53	0.3
100 ppm	2.0	100 ppm	1.6	60	0.4
150 ppm	2.2	150 ppm	1.8	66	0.5
200 ppm	2.3	200 ppm	1.9	76	0.6
300 ppm	2.5	300 ppm	2.1	84	0.7
400 ppm	2.6	400 ppm	2.2	94	0.8
800 ppm	2.9	800 ppm	2.5	104	0.9

Sample Problem

A pool water has the following characteristics: pH-7.4; alkalinity-50; calcium hardness-50 ppm; temperature-78 F. Using the tables above, the factors are 1.7 for alkalinity, 1.3 for calcium hardness; and 0.6 for temperature. Using the formula:

L.I. = 7.4 + 1.7 + 1.3 + 0.6 - 12.1 = -1.1

This pool water is not balanced and is corrosive. The pH is within the required range and almost ideal, however, the alkalinity and calcium hardness should be much higher (see Appendix B). Alkalinity can be raised by adding sodium bicarbonate (baking soda) and lowered by adding muriatic acid or sodium bisulfate (dry acid). Calcium hardness can be raised by adding calcium chloride but can only be lowered by replacing part or all of the pool water with lower calcium hardness water.

Appendix B

Langelier Index/water Balance Nomograph Method

Measure the pool water pH, alkalinity and calcium hardness. The pH must be 7.2 - 7.8. pH can be raised by adding sodium carbonate (soda ash) and can be lowered by adding carbon dioxide (CO_2), muriatic acid or sodium bisulfate (dry acid). Using the 104 F pH scale for spa pools and the 76 F pH scale for other pools, use a straight edge to line up the pH and alkalinity readings. The point where this extended line intersects the calcium hardness line is the level of calcium hardness needed for the water to be balanced. Do the same for pH and calcium hardness to get the alkalinity level needed for balance. The alkalinity and calcium hardness levels needed should be within the recommended ranges. Alkalinity can be raised by adding sodium bicarbonate (baking soda) and lowered by adding muriatic acid or sodium bisulfate (dry acid). Calcium hardness can be raised by adding calcium chloride but can only be lowered by replacing part or all of the pool water with lower calcium hardness water.

Appendix C

Division Training Requirements for the Qualified Operator

Qualifications: A qualified operator shall have completed an operator training course that is recognized by the Division.

Training Documentation: A qualified operator shall have a current certificate or written documentation acceptable to the Division showing completion of an approved operator training course.

Certificate Available: Originals or copies of such certificate or documentation shall be available on site for inspection by the Division for each qualified operator employed at or contracted by the site, as specified in this Regulation.

Essential Topics in Qualified Operator Training Courses

Course Content

All operator training courses recognized by the Division shall include, at a minimum, the following teaching elements:

1. Water disinfection,
2. Water chemistry,
3. Mechanical systems,
4. Health and safety, and
5. Operations.

1. ***Water Disinfection***

1.1 **Disinfectants types including**

1.1.1 Descriptions of different types of disinfectants,

1.1.2 Their unique physical *(e.g., shape or state [solid, liquid, or gas])* and chemical properties *(e.g., how it reacts with acids or bases)*

1.1.3 How they disinfect and impact water chemistry and monitoring systems,

1.1.4 How to calculate dosing,

1.1.5 How they are used safely, and

1.1.6 The advantages or disadvantages of using each disinfectant.

1.2 **CT Values** or Concentration x Time values including:

1.2.1 How to calculate the amount of time needed to inactivate pathogens at a given concentration of a disinfectant, and

1.2.2 The importance and reasons for maintaining appropriate water pH and temperature.

1.3 **Bromine including:**

1.3.1 Definition of bromine as an element,

1.3.2 Its use as a residual disinfectant and oxidizer in water,

1.3.3 Bromine chemistry,

1.3.4 The disinfection role of hypobromous acid,

1.3.5 On site generation,

1.3.6 pH meter requirements to prevent false readings, and

1.3.7 Bromine reuse.

1.4 **Chlorine** including:

1.4.1 Definition of chlorine as an element,

1.4.2 Its use as a residual disinfectant and oxidizer in water,

1.4.3 Chlorine chemistry and the role of PH,

1.4.4 The Disinfection role of hypochlorous acid,

1.4.5 Unstabilized products *(sodium hypochlorite, calcium hypochlorite, lithium* hypochlorite, and chlorine gas)

1.4.6 Stabilized products (sodium dichloro-s-triazinetrione and trichloro-s-triazinetrione),

1.4.7 Safe chemical handling, and

1.4.8 On-site Chlorine generation.

1.5 **Cyanuric Acid** *(CYA)* and stabilized chlorine product use including:

1.5.1 Description of CYA and how chlorine is bound to it;

1.5.2 Description of CYA use via addition of stabilized chlorine compounds or addition of cyanuric acid alone;

1.5.3 Response curves showing the impact of CYA on stabilization of chlorine residuals in the presence of UV;

1.5.4 Dose response curves showing the impact of CYA on chlorine kill rates including the impact of CYA concentrations on diarrheal fecal incident remediation procedures;

1.5.5 Strategies for controlling the concentration of CYA; and

1.5.6 Strategies for reducing the concentration of CYA when it exceeds the maximum allowable level.

1.6 **Breakpoint/ Super-Chlorination** including how to achieve it through calculation of chemical dosing to reach the desired free chlorine level and its relationship to reducing and controlling formation of combined chlorine including guidance for how to perform breakpoint chlorination in indoor aquatic settings.

1.7 **Hyperchlorination** including procedures for implementation of fecal/vomit/blood contamination response.

1.8 **Combined Chlorine** including:

1.8.1 How different combined chlorine and disinfection by-products are formed in the water and air;

1.8.2 The maximum acceptable level of combined chlorine;

1.8.3 How methods such as water replacement, breakpoint chlorination, ultraviolet light, ozone, ventilation, and use of other oxidizers can reduce combined chlorine level;

1.8.4 The advantages and disadvantages of each; and 5) Possible health effects of combined chlorine products in the air, particularly in indoor aquatic facilities.

1.9 **Secondary Disinfection Systems** including:

1.9.1 How ozone and ultraviolet disinfectants are used in conjunction with residual disinfectants to inactivate pathogens, and

1.9.2 Sizing guidelines/dosing calculations, safe use, and advantages and disadvantages of each method.

1.10 **Supplemental Disinfection** including other disinfection chemicals or systems on the market and their effectiveness in water treatment.

2. ***Water Chemistry*** Course work for water chemistry shall include:

2.1 **Source Water** including requirements for supply and pre-treatment.

2.2 **Water Balance** including:

2.2.1 Effect of unbalanced water on disinfection, pool feature surfaces, mechanical equipment, and fixtures; and

2.2.2 Details of water balance including pH, total alkalinity, calcium hardness, temperature, and TDS.

2.3 **Saturation Index** including calculations, ideal values, and effects of values which are too low or too high.

2.4 **Water Clarity** including:

2.4.1 Reasons why water quality is so important;

2.4.2 Causes of poor water clarity;

2.4.3 Maintenance of good water clarity; and

2.4.4 Closure requirements when water clarity is poor.

2.5 **pH** including:

2.5.1 How pH is a measure of the concentration of hydrogen ions in water;

2.5.2 Effects of high and low pH on bathers and equipment;

2.5.3 Ideal pH range for bather and equipment;

2.5.4 Factors that affect pH;

2.5.5 How pH affects disinfectant efficacy; and

2.5.6 How to decrease and increase pH.

2.6 **Total Alkalinity** including:

2.6.1 How total alkalinity relates to pH;

2.6.2 Effects of low and high total alkalinity;

2.6.3 Factors that affect total alkalinity;

2.6.4 Ideal total alkalinity range, and

2.6.5 How to increase or decrease total alkalinity.

2.7 **Calcium Hardness** including:

2.7.1 Why water naturally contains calcium;

2.7.2 How calcium hardness relates to total hardness and temperature;

2.7.3 Effects of low and high calcium hardness;

2.7.4 Factors that affect calcium hardness;

2.7.5 Ideal calcium hardness range; and

2.7.6 How to increase or decrease calcium hardness.

2.8 Temperature including:

2.8.1 How low and high water temperatures increase the likelihood of corrosion and scaling, respectively;
2.8.2 Effect on disinfection, its health effects, and other operational considerations;
2.8.3 Health effects; and
2.8.4 Other operational considerations.

2.9 **Total Dissolved Solids** *(TDS)* including:
2.9.1 Why the concentration of TDS increases over time;
2.9.2 Association with conductivity and organic contaminants; and
2.9.3 Key TDS levels as they relate to starting up a pool and galvanic corrosion.

2.10 **Water Treatment Systems** including:
2.10.1 Descriptions of system use, monitoring, calibration, and maintenance of automatic controllers;
2.10.2 Descriptions of common types of liquid, dry chemical, and gas mechanical feeders;
2.10.3 CHLORINE, bromine, and ozone generators;
2.10.4 Ultraviolet light systems;
2.10.5 Unique features of feeders, generators, and systems;
2.10.6 How to generally operate and maintain them;
2.10.7 Advantages and disadvantages of different feeders, ultraviolet light systems, and ozonator types; and
2.10.8 Alternate treatment methods.

2.11 **Water Testing** including:
2.11.1 How different methods *(including but not limited to colorimetric, titrimetric, turbidimetric, and electronic)* test water to determine the following levels:
2.11.1.1 Free available and total chlorine,
2.11.1.2 Total bromine,
2.11.1.3 pH,
2.11.1.4 Total alkalinity,
2.11.1.5 Calcium hardness,
2.11.1.6 Temperature,
2.11.1.7 TDS,
2.11.1.8 CYA,
2.11.1.9 Metals, and
2.11.1.10 Any other tests *(including but not limited to salt concentrations, phosphates, nitrates, potassium monopersulfate, copper, iron, and bacterial testing)*;
2.11.2 The advantages and disadvantages of each method;
2.11.3 How to maintain testing equipment;
2.11.4 How to collect water samples;
2.11.5 How to perform and interpret tests;
2.11.6 How frequently to test;
2.11.7 The steps of the dilution method; and
2.11.8 How to calculate combined chlorine levels.

3. ***Mechanical Systems*** Course work for mechanical systems shall include:
3.1 **Maintenance Calculations** including:
3.1.1 Explanations of why particular calculations are important;
3.1.2 How to convert units of measurement within and between the English and metric systems;
3.1.3 How to determine the surface area of regularly and irregularly shape aquatic venues
3.1.4 How to determine the water volume of regularly and irregularly shaped aquatic venues; and
3.1.5 Why proper sizing of filters, pumps, pipes, and feeders is important
3.2 Circulation including:
3.2.1 Why circulation is needed;
3.2.2 Factors that affect water flow;

3.2.3 How direct suction and overflow systems work;
3.2.4 How to calculate turnover and flow rates;
3.2.5 How the following components of the circulation system relate to each other:
3.2.5.1 Main drains,
3.2.5.2 Gutters and surface skimmers,
3.2.5.3 Circulation pump and motor,
3.2.5.4 Surge tanks,
3.2.5.5 Vacuum ports,
3.2.5.6 Valves, and
3.2.5.7 Return inlets;
3.2.6 How to read flow meters;
3.2.7 How to safely operate pressurized systems after the pump;
3.2.8 Information on dye testing; 9) An understanding of total dynamic head *(TDH)*;
3.2.9 How it TDH calculated;
3.2.10 How TDH is field-determined using vacuum and pressure gauges;
3.2.11 TDH effect on pump flow; and
3.2.12 Cross connections
3.3 **Main Drains** including:
3.3.1 A description of the role of main drains;
3.3.2 Why they should not be resized without engineering and public health consultation;
3.3.3 The importance of daily inspection of structural integrity; and
3.3.4 Discussion on balancing the need to maximize surface water flow while minimizing the likelihood of entrapment.
3.4 **Gutters & Surface Skimmers** including:
3.4.1 Why it is important to collect surface water;
3.4.2 A description of different gutter types *(at a minimum: scum, surge, and rim-flow)*;
3.4.3 How each type generally works;
3.4.4 The advantages and disadvantages of each; and
3.4.5 Description of the components of SKIMMERS *(e.g., weir, basket, and equalizer assembly)* and their respective roles.
3.5 **Mechanical System Balance** including:
3.5.1 An understanding of mechanical system balancing;
3.5.2 Methodology for setting proper operational water levels;
3.5.3 Basic hydraulics which affect proper functioning of the balance tank and aquatic venues;
3.5.4 Methods of setting and adjusting modulation valves;
3.5.5 Balance lines;
3.5.6 Skimmers;
3.5.7 Main drains;
3.5.8 The operation of the water make-up system;
3.5.9 Collector tanks/gravity drainage systems; and
3.5.10 Automatic controllers.
3.6 **Circulation Pump & Motor** including:
3.6.1 Descriptions of the role of the pump and motor;
3.6.2 Self-priming and flooded suction pumps;
3.6.3 Key components of a pump and how they work together;
3.6.4 Cavitation;
3.6.5 Possible causes of cavitation; and
3.6.6 Troubleshooting problems with the pump and motor.
3.7 **Valves including** *modulating/ automatic, and check)* and their safe operation.

3.8 **Return Inlets** including a description of the role of return inlets and the importance of replacing fittings with those that meet original specifications.

3.9 **Filtration including**

3.9.1 Why filtration is needed;

3.9.2 A description of pressure and vacuum filters and different types of filter media;

3.9.3 How to calculate filter surface area;

3.9.4 How to read pressure gauges;

3.9.5 A general description of sand, cartridge, and diatomaceous earth filters and alternative filter media types to include, at a minimum, perlite, zeolite, and crushed glass;

3.9.6 The characteristic flow rates and particle size entrapment of each filter type;

3.9.7 How to generally operate and maintain each filter type;

3.9.8 Troubleshooting problems with the filter; and

3.9.9 The advantages and disadvantages of different filters and filter media.

3.10 **Filter Backwashing/Cleaning** including:

3.10.1 Determining and setting proper backwash flow rates;

3.10.2 When backwashing/cleaning should be done and the steps needed for clearing a filter of fine particles and other contaminants;

3.10.3 Proper disposal of waste water from backwash; and

3.10.4 What additional fixtures/equipment may be needed *(i.e., sump, separation tank)*.

4. ***Health and Safety*** Course work for health and safety shall include:

4.1 **Recreational Water Illness** *(RWI)* including:

4.1.1 How water can contain or become contaminated with parasites, bacteria, viruses, fungi, disinfection by-products, or unsafe levels of chemicals; and

4.1.2 The role of the operator in reducing risk.

4.2 **Causes of RWIs**

4.2.1 Common infectious and chemical causes of RWIs, including but not limited to:

4.2.1.1 Diarrheal illness *(Cryptosporidium, Giardia, Shigella, and norovirus)*;

4.2.1.2 Skin rashes *(Pseudomonas aeruginosa, molluscum contagiosum)*;

4.2.1.3 Respiratory illness *(Legionella)*;

4.2.1.4 Neurologic infections *(echovirus, Naegleria)*;

4.2.1.5 Eye/ear illness *(Pseudomonas aeruginosa, adenovirus, Acanthamoeba);*

4.2.1.6 Hypersensitivity reactions *(Mycobacterium avium complex, Pontiac fever, endotoxins)*; and

4.2.1.7 Health effects of chloramines and disinfection by-products.

4.3 **RWI Prevention** including:

4.3.1 Methods of prevention of RWIs, including but not limited to chemical level control;

4.3.2 Why public health, operators, and patrons need to be educated about RWIs and collaborate on RWI prevention;

4.3.3 The role of showering;

4.3.4 The efficacy of swim diapers;

4.3.5 Formed-stool and diarrheal fecal incident response; and

4.3.6 Developing a plan to minimize pathogens and other biological *(e.g., blood, vomit, sweat, urine, and skin and hair care products)* contamination of the water.

4.4 **Risk Management** including techniques that identify hazards and risks and that prevent illness and injuries associated with aquatic facilities open to the public.

4.5 **Record Keeping** including the need to keep accurate and timely records of the following areas:

4.5.1 Operational conditions *(e.g., water chemistry, water temperature, filter pressure differential, flow meter reading, and water clarity)*;

4.5.2 Maintenance performed *(e.g., backwashing, change of equipment)*;

4.5.3 Incidents and response *(e.g., fecal incidents in the water and injuries)*; and

4.5.4 Staff training and attendance.

4.6 **Chemical Safety** including steps to safely store and handle chemicals including:

4.6.1 How to read labels and material safety data sheets;

4.6.2 How to prevent individual chemicals and inorganic and organic chlorine products from mixing together or with other substances *(including water)* or in chemical feeders; and

4.6.3 Use of PPE.

4.7 **Entrapment Prevention** including:

4.7.1 Different types of entrapment *(e.g., hair, limb, body, evisceration/disembowelment, and mechanical)*;

4.7.2 How to prevent and/or decrease likelihood of entrapment; and

4.7.3 Requirements of the Virginia Graeme Baker Pool and Spa Safety Act.

4.8 **Electrical Safety** including possible causes of electrical shock and steps that can be taken to prevent electrical shock *(e.g., bonding, grounding, ground fault interrupters, and prevention of accidental immersion of electrical devices)*.

4.9 **Rescue Equipment** including a description and rationale for the most commonly found rescue equipment including:

4.9.1 Rescue tubes,

4.9.2 Reaching poles,

4.9.3 Ring buoys and throwing lines,

4.9.4 Backboards,

4.9.5 First aid kits,

4.9.6 Emergency alert systems,

4.9.7 Emergency phones with current numbers posted, and

4.9.8 Resuscitation equipment.

4.10 **Injury Prevention** including basic steps known to decrease the likelihood of injury, at a minimum:

4.10.1 Banning glass containers at aquatic facilities,

4.10.2 Patron education, and

4.10.3 Daily visual inspection for hazards.

4.11 **Drowning Prevention** including causes and prevention of drowning.

4.12 **Barriers** including descriptions of how fences, gates, doors, and safety covers can be used to prevent access to water; and basics of design that effectively prevent access to water.

4.13 **Signage & Depth Markers** including the importance of maintaining signage and depth markers.

4.14 **Facility Sanitation** including:

4.14.1 Steps to clean and disinfect all surfaces that patrons would commonly come in contact with *(e.g., deck, restrooms, and diaper-changing areas)*, and

4.14.2 Procedures for implementation of Section 9.28 *Fecal-Vomit-Blood Contamination Response*, in relation to responding to a body fluid spill on these surfaces.

4.15 **Emergency Response Plan** including:

4.15.1 Steps to respond to emergencies *(at a minimum, severe weather events, drowning or injury, contamination of the water, chemical incidents)*; and

4.15.2 Communication and coordination with emergency responders and local health department notification as part of an Emergency Action Plan.

5. ***Operations*** Course work for operations shall include:

5.1 **Regulations** including the application of local, regional, state, and federal regulations and standards relating to the operation of aquatic facilities.

5.2 **Immediate Closure** - Course work shall also highlight reasons why an inspector or operator would immediately close an aquatic facilities.

5.3 **Local & State Health Departments** including stressing the importance of a good working relationship with the local and state health department.

5.4 **Aquatic Facility Types** including common aquatic venue types and settings and a discussion of features and play equipment that require specific operation and maintenance steps.

5.5 **Daily/Routine Operations** including listing and describing the daily inspection and maintenance requirements of an aquatic facility including, but not limited items listed:

5.5.1 Walkways/deck and exits are clear, clean, free of debris;

5.5.2 Drain covers, vacuum fitting covers, skimmer equalizer covers, and any other suction outlet covers are in place, secure, and unbroken;

5.5.3 Skimmer baskets, weirs, lids, flow adjusters, and suction outlets are free of any blockage;

5.5.4 Inlet and return covers and any other fittings are in place, secure, and unbroken;

5.5.5 Safety warning signs and other signage are in place and in good repair;

5.5.6 Entrapment prevention systems are operational;

5.5.7 Recirculation, disinfection systems, controller*(s)*, and probes are operating as required;

5.5.8 Secondary and/or supplemental disinfection systems are operating as required;

5.5.9 Underwater lights and other lighting are intact with no exposed wires or water in lights;

5.5.10 Slime and biofilm has been removed from accessible surfaces of aquatic venue, slides, and other aquatic features;

5.5.11 Doors to nonpublic areas *(chemical storage spaces, offices, etc.)* are locked;

5.5.12 First aid supplies are stocked;

5.5.13 Emergency communication equipment and systems are operational;

5.5.14 Fecal/vomit/blood incident contamination response protocols, materials, and equipment are available;

5.5.15 Aquatic features and amenities are functioning in accordance with the manufacturer's recommendations;

5.5.16 Fencing/barriers, gates, and self-latching or other locks are tested and are intact and functioning properly, and barriers do not have nearby furniture to encourage climbing;

5.5.17 Drinking fountains are clean and in functional condition;

5.5.18 Electrical devices are in good working condition and meet the requirements specified by Code;

5.5.19 Alarms, if required, are tested and functioning properly;

5.5.20 Assessing glare conditions throughout operating hours to assess whether the bottom and objects in the pool are clearly visible;

5.5.21 Play structures and diving boards are in good condition;

5.5.22 Safety equipment is in good condition, properly secured, accessible for intended use, and shall include at a minimum:

5.5.22.1 Rescue tubes,

5.5.22.2 Resuscitation masks with one-way valve,

5.5.22.3 First aid kits,

5.5.22.4 AED's,

5.5.22.5 Emergency oxygen,

5.5.22.6 Backboard, head immobilizer, straps, and

5.5.22.7 Lifeguard stands;

5.5.23 Emergency shut-off systems *(slides, water features, pumps, etc.)* function properly;

5.5.24 Depth markings are clearly visible;

5.5.25 Lifelines and buoys are in place and in good working order;

5.5.26 Ladders are non-slip and rungs secured tightly;

5.5.27 Waterslides are in functional, safe condition;

5.5.28 Moveable fulcrum is adjusted properly to control spring in the board as necessary;

5.5.29 Moveable starting blocks are properly stored;

5.5.30 Access to permanent starting blocks is restricted or controlled when not in use by swim teams and prohibited when not in use by competitive swimming or swimming practice that is under direct supervision of an instructor or coach;

5.5.31 Railings are secure;

5.5.32 SVRS is functioning according to manufacturer's guidelines;

5.5.33 Skimmers baskets and covers are clean and in place;

5.5.34 Water quality and clarity is Regulation compliant;

5.5.35 Water level is at an appropriate level;

5.5.36 Pumps retain the appropriate pressure;

5.5.37 Play structures are secure *(consider water velocity and reference manufacturers recommended levels)*; and

5.5.38 Verify required documentation and records are in place and signed by the appropriate personnel.

5.6 **Preventive Maintenance** including how to develop:

5.6.1 A preventive maintenance plan,

5.6.2 Routine maintenance procedures, and

5.6.3 Record keeping system needed to track maintenance performed.

5.7 **Weatherizing** including the importance of weatherizing and the steps to prevent damage to aquatic facilities and their mechanical systems due to very low temperatures or extreme weather conditions *(e.g., flooding)*.

5.8 **Facility Renovation & Design** – Aquatic facility renovation and design including:

5.8.1 Definitions of aquatic facility renovation, remodeling, and substantial alternation;

5.8.2 When it is necessary to renovate;

5.8.3 When it is necessary to notify the Division of planned renovations and remodeling; and

5.8.4 Current trends in facility renovation and design.

5.9 **Heating** issues including:

5.9.1 Recommended water temperatures and limits,

5.9.2 Factors that contribute to the water's heat loss and gain,

5.9.3 Heating equipment options,

5.9.4 Sizing gas heaters, and

5.9.5 How to troubleshoot problems with heaters.

5.10 **Air Circulation** including:

5.10.1 Air handling system considerations for an indoor aquatic facility,

5.10.2 The importance of regulating humidity,

5.10.3 The need to maintain negative pressure,

5.10.4 How poor indoor air quality can affect patrons and staff, and

5.10.5 How to balance air change and energy efficiency.

5.11 **Spa & Therapy Pool Issues** including:

5.11.1 Operational implications of smaller volumes of water and hot water,

5.11.2 How to maintain water chemistry,

5.11.3 Typical water temperature ranges highlighting maximum temperatures,

5.11.4 Risks of hyperthermia and hypothermia,

5.11.5 Need for emergency shut-off switches, and

5.11.6 Frequency of cleaning, draining, and disinfection.

19 DE Reg. 309 (10/01/15)
