# Supplementary material for: Assessing state partner use of the Model Aquatic Health Code (MAHC): A cross comparison of five states with varying degrees of self-reported adoption status
Source: PLOS Water. Author manuscript; Available in PMC 2025 Jul 11. (PMC12247143; doi:10.1371/journal.pwat.0000276)
Supplement: S4_Text — S4 Text. Florida pool codes. Florida public swimming pool and bathing places code established September 2015. (PDF) [file NIHMS2046419-supplement-S4_Text.pdf]

CHAPTER 64E-9
PUBLIC SWIMMING POOLS AND BATHING PLACES

64E-9.001 General
64E-9.002 Definitions (Repealed)
64E-9.003 Forms (Repealed)
64E-9.0035 Exemptions
64E-9.004 Operational Requirements
64E-9.005 Construction Plan or Modification Plan Approval (Repealed)
64E-9.006 Construction Plan Approval Standards (Repealed)
64E-9.007 Recirculation and Treatment System Requirements (Repealed)
64E-9.008 Supervision and Safety
64E-9.009 Wading Pools (Repealed)
64E-9.010 Spa Pools (Repealed)
64E-9.011 Water Recreation Attractions and Specialized Pools (Repealed)
64E-9.013 Bathing Places
64E-9.015 Fee Schedule
64E-9.016 Variances
64E-9.017 Enforcement
64E-9.018 Public Pool Service Technician Certification

64E-9.001 General.

(1) Regulation of public swimming pools and bathing places is considered by the department as significant in the prevention of disease, sanitary nuisances, and accidents by which the health or safety of an individual(s) may be threatened or impaired. Any change resulting in the operation of the pool in a manner unsanitary or dangerous to public health or safety shall subject the state operating permit to suspension or revocation. Failure to comply with any of the requirements of these rules shall constitute a public nuisance dangerous to health.

(2) All pools which do not meet the definition of private pools are public pools.

(3) A public pool owner or their agent shall first make application to the department for an initial operating permit on form DH 4159, Application for a Swimming Pool Operating Permit, 9/2015, herein incorporated by reference and available at http://www.flrules.org/Gateway/reference.asp?No=Ref-06893, along with the accompanying information listed in Section 514.031(1)(a), F.S., the required fee and a copy of the construction plans and specifications. The application shall be deemed incomplete pursuant to Section 120.60, F.S., until a copy of the final building department inspection is received by the department.

(a) After submitting an application for an initial operating permit, the owner or agent shall have the plans reviewed and a permit issued for the construction of a public pool by the jurisdictional building department in accordance with the Florida Building Code, Building, Chapter 4, Section 454.1.

(b) Upon completion of initial construction, the pool shall not be opened by the owner/operator for public use until an operational permit is issued by the department. At construction completion, the owner/operator or their agent shall notify the department in writing to request the department’s initial operating permit inspection. A copy of the final building department inspection shall be submitted with this request.

(c) For modifications, the owner/operator or agent shall submit form DH 4159 to the Department and follow the same sequence in paragraphs (a) and (b), however, the department does not charge a state fee.

(4) Annually, the pool owner/operator shall apply for an operating permit renewal from the department on form DH 4159. Approval of the permit shall be based upon the pool’s compliance with this chapter, with the previous operating permit, and the maintenance of the pool in the same functional, safety, and sanitation conditions as approved by the jurisdictional building department or the department. For the purposes of this determination, department staff shall refer to and use the Florida Building Code (FBC), Building Chapter 4, public swimming pool Section 454.1, or its predecessor, that was in effect at the time of the pool’s construction permitting. Annual operating permits expire on June 30.

(5) The 2014 FBC section 454.1 is hereby incorporated by reference, has been deemed copyright protected, and is available for inspection at the Department of Health, Bureau of Environmental Health, 4025 Esplanade Way, Tallahassee, Florida 32399-1710 or

at the Department of State, R.A. Gray Building, 500 South Bronough Street, Tallahassee, Florida 32399-0250.

Rulemaking Authority 381.006, 514.021 FS. Law Implemented 381.006, 514.0115(5), 514.021, 514.03, 514.031, 514.05, 514.06 FS. History–New 10-5-93, Formerly 10D-5.130, Amended 12-27-98, 5-27-04, 5-24-09, 7-20-16.

64E-9.002 Definitions.

Rulemaking Authority 381.006, 514.021 FS. Law Implemented 514.021, 514.03, 514.031, 514.05, 514.06, 514.071 FS. History–New 10-5-93, Formerly 10D-5.131, Amended 12-27-98, 5-27-04, 5-24-09, Repealed 7-20-16.

64E-9.003 Forms.

Rulemaking Authority 381.006, 514.021 FS. Law Implemented 381.006, 514.0115, 514.021, 514.025, 514.03, 514.031, 514.033 FS. History–New 10-5-93, Formerly 10D-5.132, Amended 12-27-98, 3-30-00, 5-27-04, 5-24-09, Repealed 7-20-16.

64E-9.0035 Exemptions.

(1) A person seeking an initial exemption, or an existing facility claiming an exemption from department regulation pursuant to the provisions of Section 514.0115, F.S., shall submit an application and supportive documentation to the department, as described below.

(a) Applicants for an exemption pursuant to Section 514.0115(2)(a) or (2)(b), F.S., shall submit either a completed form DH 4065, Application for a Swimming Pool Exemption Status – 32 Units or Less, 03/98, or a completed form DH 1704, Application for a Swimming Pool Exemption Status More Than 32 Units, 03/98, both of which are hereby incorporated by reference. Copies of these forms are available at http://www.flrules.org/Gateway/reference.asp?No=Ref-06894, and http://www.flrules.org/Gateway/reference.asp?No=Ref-06895.

(b) For purposes of determining exemption status, the term condominium shall be as defined in Chapter 718, F.S., and the term cooperative shall be as defined in Chapter 719, F.S. Applicants shall provide either the recorded declaration of condominium or the recorded cooperative documents and any additional documents which establish the criteria set forth in Section 514.0115(2)(a), or (2)(b), F.S.

(2) Beginning July 1, 2010, exemptions shall be renewed by July 1, every five years. Applicants seeking renewal, who have no changes to the pool or ownership, shall only submit the application form. If swimming pool related or ownership changes have been made, this information shall be submitted along with the application form.

(3) A person who received an exemption shall contact the department if the conditions upon which the exemption was granted change so as to eliminate the exemption status. Under such circumstances, the pool shall comply with the provisions of this chapter and Chapter 514, F.S.

(4) An exemption from department rules does not exempt the pool from other federal, state, and local requirements.

Rulemaking Authority 381.006, 514.021 FS. Law Implemented 514.0115, 514.031 FS. History–New 5-24-09, Amended 7-20-16.

64E-9.004 Operational Requirements.

(1) Water Quality – The water supply for all pools shall be an approved potable water system or shall meet the requirements for potable water systems by the submission from the operator of annual bacteriological and chemical laboratory reports to the county health department. Salt water sources are exempt from the potable water chemical standards except for iron and color requirements.

(a) Cross-connection prevention – To safeguard water quality, devices or systems shall be operational and maintained in their original functional condition.

(b) Bacteriological quality – The pool water shall be free of coliform bacteria contamination.

(c) Clarity – The pool water shall be 0.5 or less NTU and the main drain grate must be readily visible from the pool deck.

(d) Chemical quality – Chemicals used in controlling the quality of the pool water shall be tested and approved using the NSF/ANSI Standard 60-2011, Drinking Water Treatment Chemicals-Health Effects dated May 2011, which is incorporated by reference in these rules and shall be compatible with other accepted chemicals used in pools. The following parameters shall be adhered to for pool water treatment:

1. pH – 7.2 to 7.8.

2. Disinfection – Free chlorine residual shall be 1 milligram per liter (mg/L) to 10 mg/L, inclusive, in conventional swimming

pools and 2 mg/L to 10 mg/L, inclusive, in all other type pools such as spa-type pools and interactive water fountains; bromine residual shall be 1.5 mg/L to 10 mg/L, inclusive, in conventional swimming pools and 3 mg/L to 10 mg/L, inclusive, in all other type pools. Except that, the following maximum disinfectant levels shall apply to indoor conventional swimming pools: 5 mg/L free chlorine or 6 mg/L bromine.

3. When oxidation-reduction potential (ORP) controllers are required, the water potential shall be kept between 700 and 850 millivolts. Use of these units does not negate the manual daily testing requirement of subsection 64E-9.004(10), F.A.C.

4. Cyanuric acid – 100 mg/L maximum in pools, with 40 mg/L as the recommended maximum, and 40 mg/L maximum in spa pools.

5. Quaternary ammonium – 5 mg/L maximum.

6. Copper – 1 mg/L maximum.

7. Silver – 0.1 mg/L maximum.

(e) Landscape irrigation water that wets the wet deck area of the pool, the pool itself, enters the collector tank, or wets an interactive water feature must be potable water from a public water system or shall meet the bacteriological quality of potable water as evidenced by annual laboratory analysis submitted to the department. Reclaimed water may not be used in these areas. If reclaimed water is used in the vicinity of the pool (inside of the pool fence or within 100 feet of the pool water's edge) it must employ drip irrigation or soaker hoses. Signs shall be posted notifying pool patrons that reclaimed water is in use, and is not to be consumed.

(2) Manual addition of chemicals will be allowed under special conditions and requires that the pool be closed prior to addition and for at least 1 hour period after addition or a longer period as necessary for sufficient and safe distribution of the chemical. After treatment for breakpoint chlorination and algae prevention, use of the pool can be resumed when the free chlorine levels drop to 10 mg/L.

(3) Cleanliness – The pool and pool deck shall be kept free from sediment, floating debris, visible dirt and algae. Pools shall be refinished when the pool surfaces cannot be maintained in a safe and sanitary condition.

(4) Food, beverages, glass containers, and animals are prohibited in the pool. Individuals with a disability and service animal trainers may be accompanied by a service animal, as defined in Chapter 413.08, F.S., but the service animal is not allowed to enter the pool water or onto the drained area of an interactive water feature (IWF) in order to prevent a direct threat to the health of pool patrons.

(5) The pool recirculation system must be operated at all times when the pool is open for use. The recirculation system may be shut off three hours after the pool closes but must resume operation three hours before opening the pool. Shut down time must be controlled by a time clock. When a variable speed pump is used, the recirculation system shall be operated such that it achieves the equivalent of 6 hours of treatment at 100% design flowrate during the daily closed period, or at least one complete water volume turnover, whichever is greater. Exception: vacuum DE systems are excluded from this allowance.

(6) The pool water level must be maintained at an elevation suitable for continuous skimming without flooding during periods of non-use.

(7) When use of a public swimming pool requires an admission or a membership fee, the most recent pool inspection report shall be posted in plain view of existing and potential members and patrons.

(8) Footbaths are prohibited.

(9) Test kits are required to be on the premises of all pools to determine free active chlorine and total chlorine using N, N-Diethyl-p-Phenylenediamine (DPD), or bromine level, total alkalinity, calcium hardness, and pH. NSF/ANSI Standard 50-2012 certified water quality test devices/kits or specific laboratory analysis methods identified by the chemical product manufacturer must be available to determine the concentration in pool water of all NSF/ANSI Standard 60-2011 approved chemicals that are fed or added to a public pool, or the chemical cannot be used. NSF/ANSI Standard 50-2012, Equipment for Pools, Spas, Hot Tubs and other Recreational Water Facilities, September 16, 2012, is hereby incorporated by reference, has been deemed copyright protected, and is available for review at the Department of Health, Bureau of Environmental Health, 4025 Esplanade Way, Tallahassee, Florida 32399-1710 or at the Department of State, R.A. Gray Building, 500 South Bronough Street, Tallahassee, Florida 32399-0250.

(a) If the following chemicals are fed or added to the pool water, then test kits for the specific chemical must be used: cyanuric acid, sodium chloride, quaternary ammonium, ozone and copper.

(b) When silver is added as a supplemental disinfectant, a water analysis must be done every six months and be submitted to the department upon request.

(c) A test kit may be used for multiple pools, provided the pools have common ownership and they are located on contiguous property.

(d) The test kit shall be capable of measuring the level of disinfectant in the normal operating range.

(10) The keeping of a daily record of information regarding pool operation, using form DH 921, Monthly Swimming Pool Report, 3/98, hereby incorporated by reference and available at http://www.flrules.org/Gateway/reference.asp?No=Ref-06896, shall be the responsibility of the pool owner or operator. Customized report forms may be substituted provided they contain the appropriate information and are made available to the department. The completed report shall reflect manually conducted pool water tests for pH and disinfectant levels at least once every 24 hours, and weekly testing for cyanuric acid when chlorinated isocyanurates are used at spas and pools, and shall be retained at the pool and made available to the department upon request. Any able person can test the pool water and record it in the report.

(11) Should a human fecal accident occur, the pool operator or owner shall comply with all recommendations found in the Centers for Disease Control and Prevention's (CDC) "Fecal accident response recommendations for Aquatics Staff" dated February 15, 2008, hereby incorporated by reference and available at http://www.flrules.org/Gateway/reference.asp?No=Ref-06897. Alternative emergency disinfection methods developed by industry, or by the application of new disinfection technology, or by the use of chemical disinfectants that are effective, safe and appropriate for public bathing facilities, and are approved by the CDC, may also be used.

Rulemaking Authority 381.006, 514.021 FS. Law Implemented 381.006, 514.021, 514.031 FS. History–New 10-5-93, Formerly 10D-5.133, Amended 12-27-98, 5-27-04, 5-24-09, 7-20-16.

64E-9.005 Construction Plan or Modification Plan Approval.

Rulemaking Authority 381.006, 514.021 FS. Law Implemented 381.006, 514.021, 514.025, 514.03, 514.031, 514.05, 514.06 FS. History–New 10-5-93, Formerly 10D-5.134, Amended 12-27-98, 5-27-04, 5-24-09, Repealed 7-20-16.

64E-9.006 Construction Plan Approval Standards.

Rulemaking Authority 381.006, 514.021 FS. Law Implemented 381.006, 514.021, 514.03, 514.031, 514.05, 514.06 FS. History–New 10-5-93, Formerly 10D-5.135, Amended 12-27-98, 5-27-04, 5-24-09, Repealed 7-20-16.

64E-9.007 Recirculation and Treatment System Requirements.

Rulemaking Authority 381.006, 514.021 FS. Law Implemented 381.006, 514.021, 514.03, 514.031, 514.05, 514.06 FS. History–New 10-5-93, Formerly 10D-5.136, Amended 12-27-98, 5-27-04, 5-24-09, Repealed 7-20-16.

64E-9.008 Supervision and Safety.

(1) All owners, managers, lifeguards or swimming instructors in charge of, or working at, public swimming pools shall be responsible for the supervision and safety of the pool.

(2) Lifeguard and Swimming Instructor Requirements.

(a) Definitions:

1. "Lifeguard" – Person responsible for the safety of the users of a public swimming pool.

2. "Nationally Recognized Aquatic Training Program" – A training and certification program for swimming instructors and lifeguards equivalent to the programs offered by the American Red Cross or the Y.M.C.A.

3. "Swimming Instructor" – Person who offers progressive swimming instruction.

(b) Lifeguards or swimming instructors, if provided, shall be in full charge of persons using the pool and shall have authority to enforce all rules. Lifeguards and swimming instructors shall be certified in lifeguarding or swimming instruction, respectively, by the American Red Cross, the YMCA or other equivalent national aquatic training agencies which meet the established standards, objectives and standards of care provided in the American Red Cross or YMCA programs. For the purpose of this rule, the standards found in the 2007 edition of the American Red Cross Lifeguarding Instructors Manual, the 2009 edition of the American Red Cross Water Safety Instructors Manual, the On the Guard, The YMCA Lifeguard Manual, (2011) Fifth Edition, (YMCA), The Youth and Adult Aquatic Program Manual (1999), and (YMCA) The Parent/Child and Preschool Aquatic Program Manual (1999), are hereby adopted by reference, have been deemed copyright protected, and are available for review at the Department of Health, Bureau of

Environmental Health, 4025 Esplanade Way, Tallahassee, Florida 32399-1710 or at the Department of State, R.A. Gray Building, 500 South Bronough Street, Tallahassee, Florida 32399-0250. Swimming instructors of developmentally disabled students shall also be certified in accordance with Section 514.072, F.S.

(c) Lifeguards and swimming instructors shall also be currently certified in first aid and in adult, child and infant cardiopulmonary resuscitation through the American Red Cross, the American Heart Association, the National Safety Council, the American Academy of Orthopaedic Surgeons, by Medic First Aid International, Inc., or by American Safety and Health Institute.

(d) Swim coaches are exempted from the swimming instructor certification requirement when training advanced level swimmers for competition.

(e) Verification of equivalence, as required above, shall be the responsibility of the department. The department shall form an ad hoc advisory group composed of professionals in the field of aquatics. This group shall consist of five members and shall make recommendations to the department regarding the equivalence of lifeguard or swimming instructor certification programs submitted to the department under paragraph (b), above. Members shall be appointed for a period of 3 years with such appointments being staggered so that the terms of no more than two members expire in any one year.

(f) Lifeguard, swimming instructor, cardiopulmonary resuscitation and first aid certificates or photocopies thereof shall be maintained at the pool location and be available for inspection by department personnel at any reasonable hour.

(3) Safety Equipment – All pools shall be equipped with the following:

(a) Safety drain outlet cover(s)/grate(s) and allowable secondary anti-entrapment devices as required by section 514.0315, F.S.

(b) A shepherd’s hook securely attached to a one piece pole not less than 16 feet in length. Pools over 50 feet in length shall have a shepherd’s hook on each of the longer sides of the pool.

(c) At least one 18 inch diameter lifesaving ring with sufficient rope attached to reach all parts of the pool from the pool deck. Pools over 50 feet in length shall have a lifesaving ring on each of the longer sides of the pool.

(d) Safety equipment shall be mounted in a conspicuous place and be readily available for use.

(e) Spa pools under 200 square feet of surface area, and interactive water features or wading pools with two feet or less of water depth are exempt from this shepherd’s hook and lifesaving ring requirement.

(4) Safety Lines – All pools with a slope transition shall maintain safety line anchors and a safety line in place at all times. A safety line may be temporarily removed from the pool for patrons to swim laps only when there is a safety attendant or lifeguard present, and it must be reinstalled to its proper location upon completion of the exercise.

(5) Pool covers and solar blankets shall only be used during times when the pool is closed. If a pool cover or solar blanket is installed, it shall be secured around the entire perimeter and designed to support a live load of an adult person, or the pool area shall be inaccessible to unauthorized individuals during times of cover or blanket use.

(6) Pool Rules and regulations – Rules for bathers shall be posted as approved by the jurisdictional building department.

(7) Night swimming – Pools shall not be open for swimming at night unless lighting is provided as approved by the jurisdictional building department. Pools authorized for night swimming shall be noted on the operating permit issued by the department. Night swimming shall be considered one half hour before sunset to one half hour after sunrise.

(8) Pools with heaters shall have a maximum water temperature of 104º F and a functional in-line thermometer.

(9) General Pool Maintenance for Patron Safety.

(a) The bathing load shall be posted and the owner/operator shall not permit the bathing load to be exceeded at any time.

(b) The filtration system for swimming pools shall be maintained as capable of meeting operating performance standards as identified on the most current operating permit. Flowrate may not be reduced or adjusted after the initial operating permit is issued unless approved in advance by the department. All other types of projects shall be maintained as sized according to the anticipated bathing load and proposed uses.

(c) Access – All pools shall be maintained with a means of access as approved by the jurisdictional building department.

(10) General Equipment Maintenance for Safety –

(a) Recirculation and treatment equipment such as, but not limited to filters, recessed automatic surface skimmers, ionizers, ozone generators, UV systems, automatic controllers, disinfection feeders and chlorine generators must be tested and approved using the NSF/ANSI Standard 50-2012. The standard and a list of certified products is available from www.NSF.org, and product certifications are available from other American National Standards Institute (ANSI) 3rd party accredited product certifiers. If standards do not exist for a specific product, the manufacturer should consult NSF or other ANSI accredited product certifier to develop such standards.

(b) The recirculation system shall be operated to maintain a minimum of four turnovers of the pool volume per day (once per 6 hours). Pools that are less than 1000 square feet at health clubs are required to provide eight turnovers per day (once per 3 hours). Other pool types shall maintain the following minimum pool turnover rate: spa pool – 30 minutes; IWF – 30 minutes; wading pool – 1 hour; water activity pool – 1 hour in pools two foot deep or less, or 2 hours in pools over two foot deep; zero depth entry pool – 1 hour in area less than three feet deep; water slide plunge pool – 2 hours; river ride – 3 hours, and wave pool – 3 hours. Validation of the turnover rate shall be determined by the rate of the flow indicator.

(c) For compliance with Section 514.0315, F.S., and to ensure the safety of bathers:

1. All safety features shall be tested and replaced when necessary, in accordance with the manufacturer's specifications. The operations manual shall be onsite.

2. The owner/operator shall provide a completed form DH 4157, Pool Owner/Operator Verification of Entrapment Safety Features, 09/2015, herein incorporated by reference and available at http://www.flrules.org/Gateway/reference.asp?No=Ref-06898, to the department when a change in the safety feature occurs.

3. For an existing pool with a suction limiting vent (SLV) system, the system shall be tested annually by a Chapter 489, F.S., licensed pool contractor or a Florida licensed professional engineer to validate that the vacuum release timing is in compliance with the criteria for safety vacuum release systems in Section 514.0315(2)(a), F.S. A copy of the testing shall be submitted to the department with the annual operating permit renewal application.

(d) Filters – Filters sized to handle the required recirculation flow shall be maintained to perform as originally installed in accordance with the manufacturer's specifications and remain functional as designed.

1. The maximum filtration rate in gallons per minute per square foot of filter area shall be: fifteen (15), or twenty (20) if so approved utilizing the procedure in this chapter below for high rate sand filters, three (3) for rapid sand filters, three-hundred-seventy-five thousandths (375/1,000) for pleated cartridge filters, and two (2) for Diatomaceous Earth (D.E.) type filters.

2. Pressure filter systems shall be maintained to perform as originally equipped with a functional air relief valve, influent and effluent pressure gauges with minimum face size of two inches reading 0-60 pounds per square inch (psi), and a sight glass when a backwash line is required.

3. Vacuum filter systems shall be maintained to perform as originally equipped with a functional vacuum gauge which has a two inch face and reads from 0-30 inches of mercury.

4. D.E. filter elements shall be maintained as installed with a minimum one inch clear spacing between elements. All cartridges used in public pool filters shall be maintained as permanently marked with the manufacturer's name, pore size and area in square feet of filter material. All cartridges with end caps shall have the permanent markings on one end cap. The D.E. filter tank and elements shall be maintained as installed, such that the recirculation flow draw down does not expose the elements to the atmosphere whenever only the main drain valve is open.

(e) Disinfection and pH adjustment shall be maintained as added to the pool recirculation flow using automatic feeders meeting the requirement of NSF/ANSI Standard 50-2012. All chemicals shall be fed into the return line after the pump, heater and filters, unless the feeder was designed by the manufacturer and approved by the NSF to feed to the collector tank or to the suction side of the pump. Feeding chlorinated isocyanurates disinfectant is prohibited in spas, wading pools and interactive water features. Dual or multiuse feeders can be used if approved for and feeding an acceptable rate of alternate disinfectant. Where pH adjustment feeders are not present on these three types of pools that were required to replace chlorinated isocyanurates feeders, pH adjustment feeders shall be installed. Exception: spa pools of 100 square feet or less with original department approval to be built without a pH adjustment feeder.

1. Gas chlorination – When gas chlorination is utilized, the chlorinator shall be maintained as capable of continuously feeding a chlorine dosage of six (6) mg/L to the recirculated flow of the filtration system.

a. Gas chlorinators shall be maintained in above grade rooms and in areas which are inaccessible to unauthorized persons.

b. When booster pumps are used with the chlorinator, the pump shall use recirculated pool water supplied via the recirculation filtration system. The booster pump shall be maintained as electrically interlocked with the recirculation pump to prevent the feeding of chlorine when the recirculation pump is not operating.

2. Hypohalogenation and Electrolytic chlorine generators – The hypohalogenation type feeder and electrolytic chlorine generators shall continuously feed a dosage of six (6) mg/L to the minimum required turnover flow rate of the filtration systems. Required backup chlorine feeders and generators shall be operated at least once per month and this test shall be recorded in the monthly pool log.

3. Feeders for pH adjustment – Feeders for pH adjustment shall be provided on all pools. pH adjustment feeders shall be maintained as positive displacement type, shall be adjustable from zero to full range, and shall have an electrical interlock with the circulation pump to prevent discharge when the recirculation pump is not operating. When soda ash is used for pH adjustment, the maximum concentration of soda ash solution to be fed shall not exceed one-half pound soda ash per gallon of water. Feeders for soda ash shall be capable of feeding a minimum of three gallons of the above soda ash solution per pound of gas chlorination capacity. The minimum size of the solution reservoirs shall be maintained as not be less than 50 percent of the maximum daily capacity of the feeder. The solution reservoirs shall be marked to indicate the contents.

4. Ozone generating equipment –

a. The concentration of ozone in the return line to the pool shall not exceed 0.1 mg/L.

b. Ozone generating equipment shall be maintained as equipped with an air flow meter and a means to control the flow. The generator shall be maintained as electrically interlocked with the recirculation pump to prevent the feeding of ozone when the recirculation pump is not operating. A flow sensor controller can also be used to turn off the feeder when flow is not sensed.

5. UV equipment used for any purpose shall constantly produce a dosage of at least 40 mJ/cm2 (milliJoules per square centimeter).

6. Ozone generators shall produce no more than a pool water contact concentration of 0.1 milligrams per liter (mg/L). The contact concentration in mg/L shall be calculated as the amount of ozone in grams per hours divided by the recirculation flow rate in gallons per minute times 4.41.

(11) Maintenance for Safety of Wading Pools–

(a) Automated Oxidation Reduction Potential (ORP) and pH controllers with sensing probes shall be maintained to assist in maintaining proper disinfection and pH levels.

(b) All wading pools shall have drainage to waste without a cross-connection through a quick opening valve to facilitate emptying the pool should a fecal accident occur. Should a fecal accident occur, the requirements of this chapter shall be met or the pool may be drained and both the pool and the filter system and all plumbing shall be properly disinfected.

(12) Maintenance for Safety of Spa Pools–

(a) Oils, body lotions, and minerals – Oils, body lotions, and minerals or materials not associated with chemicals used for water chemistry balance, algae control, and disinfection of the water are prohibited in the spa pool.

(b) Automated Controllers – Automated Oxidation Reduction Potential (ORP) and pH controllers with sensing probes shall be provided and maintained on spa pools to assist in maintaining proper disinfection and pH levels.

(c) Spa pool signs shall be posted as approved by the jurisdictional building department.

(d) Should a fecal accident occur, the requirements of this chapter shall be met or the spa pool may be drained and both the spa pool and the filter system and all plumbing shall be properly disinfected.

(13) Maintenance for Safety of Water Recreation Attractions and Special Purpose Pools – A lifeguard and/or safety plan shall be submitted to the department with the application for the initial operation permit of water slide plunge pools and water activity pools when climbable structures are installed.

(a) Water slide plunge pools.

1. Pump reservoir volume minimum shall be equal to three minutes of the combined flow rate in gpm of all filter and slide pumps.

2. Pump reservoirs shall be accessible only to authorized individuals.

3. Filter areas minimum requirements shall be maintained as twice the filter areas specified for the recirculation rates stipulated for other pools in this chapter and FBC Section 454.1. The filtration system shall be capable of returning the pool water turbidity to five-tenths NTU within eight hours or less after peak bather load.

4. Disinfection equipment shall be maintained as capable of feeding 12 mg/L of halogen to the continuous recirculation flow of the filtration system.

(b) Water activity pools.

1. The recirculation-filtration system of water activity pools shall achieve a minimum of one turnover every two hours for water activity pools over two feet deep, and in one hour for these pools that are two feet deep or less.

2. All water activity pool signs shall be posted as approved by the jurisdictional building department.

(c) The recirculation-filtration system for zero depth entry pools shall be of a minimum of one turnover every two hours in the area of the pool that is three feet deep or less. In the remainder of the pool where the depth is greater than three feet, the system shall

have a maximum six hour turnover rate.

(d) Special purpose pool projects may deviate from the requirements of other sections of this chapter. Only those deviations necessary to accommodate the special usage shall be allowed and all other aspects of the pool shall comply with the requirements of this chapter and the FBC section 454.1. The operating permit shall state the purpose for which the pool is to be used.

(e) Interactive Water Features (IWFs).

1. An automatic skimmer system shall be maintained if provided in the collector tank. A variable height skimmer may be used or a custom surface skimmer device may be substituted.

2. Chemical feeders shall be maintained as in accordance with this chapter, except that the disinfection feeder shall be capable of feeding 12 mg/L of free chlorine to the pressure side of the recirculation system or the collector tank (based upon a hypothetical 30 minute turnover of the contained volume within the system). Automated Oxidation Reduction Potential (ORP) and pH controllers with sensing probes shall be provided to assist in maintaining proper disinfection and pH levels.

3. Hydraulics.

a. The filter system shall filter and chemically treat all water that is returned to the spray features. The filter system shall draft from the collector tank and return filtered water directly to the spray features. Excess water not required by the spray features shall be returned to the collector tank.

b. Alternatively, the contained volume of the system may be filtered and chemically treated based upon a 30 minute turnover of the contained volume with 100% returned to the collector tank by manifold piping. If this alternative is chosen, all water returned to the spray feature(s) must also be treated with an Ultraviolet (UV) light disinfection equipment to accomplish protozoan destruction in accordance with sound engineering. This alternative must have the ability to feed 6 mg/L free chlorine to the feature water as it is returned to the spray feature. The UV disinfection equipment shall be electrically interconnected such that whenever it fails to produce the required UV dosage, the water spray features pump(s) and flow will be immediately stopped.

c. An automatic water level controller shall be provided.

d. Where the filter system described in sub-subparagraph 3.a., above, is utilized, a second filter system and disinfection system shall be provided to treat the water in the collector tank when the feature/filter pump is not in operation. Said system shall be capable of filtering the total volume of water in the collector tank in 30 minutes and the disinfection system shall be capable of providing 12 mg/L of disinfectant to this flow rate.

4. All IWF pool rule signs shall be posted as approved by the jurisdictional building department.

(f) Rules and regulations for water theme parks shall be posted as approved by the jurisdictional building department.

Rulemaking Authority 381.006, 514.021, 514.071 FS. Law Implemented 381.0015, 381.006, 514.021, 514.025, 514.03, 514.031, 514.0315, 514.05, 514.06, 514.071 FS. History–New 10-5-93, Formerly 10D-5..134, 10D-5.137, Amended 12-27-98, 5-27-04, 5-24-09, 7-20-16.

64E-9.009 Wading Pools.

Rulemaking Authority 381.006, 514.021 FS. Law Implemented 381.006, 514.021 FS. History–New 10-5-93, Formerly 10D-5.138, Amended 12-27-98, 5-27-04, 5-24-09, Repealed 7-20-16.

64E-9.010 Spa Pools.

Rulemaking Authority 381.006, 514.021 FS. Law Implemented 381.006, 386.01, 386.02, 386.03, 386.041, 386.051, 514.011, 514.021, 514.03, 514.031, 514.05, 514.06 FS. History–New 10-5-93, Formerly 10D-5.139, Amended 12-27-98, 5-27-04, 5-24-09, 7-20-16.

64E-9.011 Water Recreation Attractions and Specialized Pools.

Rulemaking Authority 381.006, 514.021 FS. Law Implemented 381.006, 514.021, 514.03, 514.031, 514.05, 514.06 FS. History–New 10-5-93, Formerly 10D-5.140, Amended 12-27-98, 5-27-04, 5-24-09, Repealed 7-20-16.

64E-9.013 Bathing Places.

(1) General – All public bathing places are required to conduct monitoring for water quality, reporting these results to the department, notice to the department and public notification upon exceedance of water quality violations. As of April 29, 2012, bathing place operation permits are no longer required from the department by law.

(2) Operational water quality – The water shall be free of chemical and physical substances known or suspected of being

capable of creating toxic reactions or skin or membrane irritations. Algae and aquatic vegetation shall be controlled so that no hazard to bathers results.

(a) Bacteriological samples shall be collected by the owner/manager and tested monthly. A set of two samples shall be collected for every 500 feet of shoreline, the samples shall be taken a foot below the surface in three feet of water and at least 25 feet apart. The samples shall be analyzed by a DOH certified laboratory using EPA approved methods for ambient water and the results submitted to the department within 10 days after the end of the month.

1. Should the test results of these samples exceed the standards in subsection 64E-9.013(3), F.A.C., below, the county health department shall be notified within 24 hours of receipt of results by the owner/manager from the lab, and re-sampling by the owner/manager shall be required within 24 hours. All sampling results shall be submitted to the county health department.

2. If 24 hour re-sampling is not possible for any reason, then the bathing place owner/manager shall immediately post a No swimming advisory based upon these initial results during the time period waiting for re-sampling results. If the 24 hour confirmation samples reveal an exceedance of the standards, the bathing place owner/manager shall immediately post a No swimming advisory, form DH 4158, Bathing Place Public Health Advisory Sign – Poor Water Quality, 02/13, incorporated by reference and available at http://www.flrules.org/Gateway/reference.asp?No=Ref-06899, or sustain the already-posted advisory until additional testing reveals the water meets single sample standards again. The department shall post the advisory if the owner/manager does not. Only samples collected after the initial advisory shall be used to compare and calculate when the advisory may be rescinded. If a pollution source is identified, that source shall be eliminated before rescinding the bathing place advisory.

(b) County health departments shall perform an inspection upon receipt of test results exceeding standards, or upon receipt of a complaint from the public concerning safety, sanitation, illness, or water quality, and this inspection may include:

1. A site inspection in light of the original sanitary survey, changed natural conditions, changed use conditions, and originally permitted facilities.

2. A bacteriological test consisting of the normal monthly sampling requirement. The fecal coliform, *E. coli* or enterococci density must not exceed the single sample standards of subsection 64E-9.013(3), F.A.C.

3. A water clarity test wherein an 8" black and white secchi disk shall be visible to a minimum depth of four feet.

4. The bathing place shall be posted with an advisory or swimming prohibited, as appropriate, by the owner/manager or the department if inspection reveals water clarity violations, unsafe bacterial test results, or immediate hazards to health or safety such as, but not limited to sewage in water, broken glass, dangerous wildlife, hazardous structural or electrical conditions, toxic algal blooms, or other serious disease agents present.

5. Muck or silt shall not be present from the shoreline to a depth of five feet and aquatic vegetation shall be controlled.

6. Should an incident or finding of the county health department warrant it, site specific signage shall be provided. The bathing load shall be posted and due consideration shall be given to safety guidelines such as steep slope, diving areas, deep water, underwater obstruction, dangerous wildlife, or lifeguard not on duty. Additional signage shall be provided if the bathing area is longer than 300 feet.

7. Platforms, diving boards, docks, beaches and walkways shall be kept clean and in good repair. Diving areas shall be readily identified, and shall have and maintain adequate water depth for safe diving based on the depth requirements of public swimming pool construction requirements.

8. Glass items and domestic animals are prohibited in the bathing area and on the adjacent beach area.

9. Sanitary facilities shall be provided and maintained in good working order with all necessary supplies.

(3) Bacteriological Standards – Either fecal coliform, *E. coli, or enterococci* bacteria shall be tested for, at the option of the permit holder. All samples tested will be considered to determine compliance, unless found to be invalid by the certified lab or county health department. The *enterococci* density shall not exceed 61 colony forming units (CFU) per 100 mL of water in any single sample; or the *E. coli* density shall not exceed 235 CFU per 100 mL of water in any single sample; or the fecal coliform shall not exceed an average of 200 CFU per 100 mL of water, nor 400 per 100 mL of water in 10 percent of the samples, nor 800 CFU per 100 mL of water in a single sample. This average shall be expressed as geometric means using at least ten samples per 30 day period. Multiple samples collected on any one day during routine monthly sampling shall be arithmetically averaged to determine compliance for the bathing place.

Rulemaking Authority 381.006, 514.021 FS. Law Implemented 381.006, 514.021, 514.03, 514.031, 514.04, 514.05, 514.06 FS. History–New 10-5-93, Formerly 10D-5.142, Amended 12-27-98, 5-27-04, 5-24-09, 7-20-16.

64E-9.015 Fee Schedule.

(1) Operating Permit Issuance for Swimming Pools:

(a) Initial Operating Permit – $150.00.

(b) Annual Operating Permit or Annual Renewal – Full annual fee if the authorization was issued from July 1st to December 31st; one half the annual fee if the authorization was issued from January 1st to June 30th. Payment is not required for a replacement copy of an operating permit or reissuances of an operating permit due to change of ownership or name.

1. Pools greater than 25,000 gallons and bathing places – $250.00.

2. Pools of 25,000 gallons or less – $125.00.

3. Exempted condominiums/cooperatives with over 32 units – $50.00.

(2) Variances – Review of application for variance – $300.00.

Rulemaking Authority 381.006, 514.021, 514.033 FS. Law Implemented 514.021, 514.03, 514.031, 514.033 FS. History–New 10-5-93, Formerly 10D-5.144, Amended 12-27-98, 5-27-04, 5-24-09, 7-20-16.

64E-9.016 Variances.

A variance from requirements of these rules may be requested by the pool owner or their representative to relieve or prevent hardship only in cases involving deviations from the rule, when it is shown that the hardship was not caused intentionally by the action of the applicant, where no reasonable alternative exists and the health and safety of the pool patrons is not at risk. Application for variance shall be submitted through the county health department utilizing DOH Form 4080, Application for Variance from Chapter 64E-9, F.A.C., 07/08, which is incorporated by reference and available at http://www.flrules.org/Gateway/reference.asp?No=Ref-06981. Each application can be accompanied by supportive materials such as drawings, pictures or manufacturer's specifications and a fee must be paid in accordance with subsection 64E-9.015(2), F.A.C. Applications must be received by the county health department at least 30 days prior to the scheduled meeting of the Governor's Swimming Pool Advisory Board. Walk-in applications at the Board meeting may be reviewed only if there is time available as determined by the Chair. Each walkin applicant must provide evidence they: have paid the variance fee to the county health department, have received a department review of the application, and applicant provides eight full sets of the application package to the Board.

Rulemaking Authority 381.006, 514.0115, 514.021 FS. Law Implemented 514.0115, 514.021, 514.028, 514.051, 514.06 FS. History–New 10-5-93, Formerly 10D-5.145, Amended 12-27-98, 5-24-09, 7-20-16.

64E-9.017 Enforcement.

Any public pool can be immediately posted closed by the department as not being in compliance with this chapter whenever any of the following conditions occur:

(1)(a) The disinfectant level is below the minimum or above the maximum that is prescribed in this chapter.

(b) The pH of the pool water is below 7.2 or above 7.8.

(c) The clarity of the pool water is such that the main drain grate is not readily visible from the pool deck.

(d) The recirculation system or disinfection feeding equipment is missing or not functioning.

(e) Any portion of the anti-entrapment system is missing or not functional, or a main drain cover/grate is missing, unsecured, improperly secured, damaged, or does not meet the requirements of this chapter by the dates specified.

(f) Operation without a valid permit.

(g) Direct suction exists on the main drain or other outlets, except vacuum fittings, automatic surface skimmer(s), and their equalizer grates provided the flow velocity through the equalizer grate does not exceed 1.5 feet per second, or the corrective actions specified in this chapter and Section 514.0315, F.S., are not completed by dates specified.

(h) Any other conditions which endangers the health, safety, or welfare of persons using the pool, which may include, but is not limited to: a drowning hazard, broken glass, sharp edged or broken tile or metal, fecal accident(s), electrical code violation, or severe biological growth. The department may attach a sign that states "Pool Closed. This pool is not in compliance with Chapter 64E-9, F.A.C., and may endanger the health, safety or welfare of persons using this facility." With the department's permission, the pool operator may remove signs from the pool area immediately following correction of the cited deficiencies provided the county health department is notified of this action.

(2) Correction of Unauthorized Modifications.

(a) When it is discovered that a pool has been modified from the original approved plans and application, corrective actions and replacement shall be allowed to occur to bring the pool back into compliance with the plans and applications as approved without the requirement for a modification permit, unless any of the following exist:

1. Critical conditions identified in paragraphs 64E-9.017(1)(d) and (g), F.A.C., above, are discovered.
2. The original approved plans and application are not available for verification.
3. The extent of the unauthorized modification cannot be readily determined by the department or the design engineer.
4. The corrective construction or replacement will place the pool in violation of current pool construction rules.
5. The construction repair is regulated under the FBC 454.1 by the jurisdictional Building official.
6. Other unsanitary or unsafe conditions apparent to the department or the design engineer.

(b) Whenever any of the conditions subparagraphs 1 through 6, above, exist, the owner shall make application to the jurisdictional Building department for a modification permit to authorize any construction required to restore the condition of the pool to an approved or original condition. A copy of this permit application shall be provided to the department.

Rulemaking Authority 381.006, 514.021, 514.05 FS. Law Implemented 381.006, 514.021, 514.04, 514.05, 514.06 FS. History–New 10-5-93, Formerly 10D-5.146, Amended 12-27-98, 5-27-04, 5-24-09, 7-20-16.

64E-9.018 Public Pool Service Technician Certification.

An individual who services a public pool by maintaining the cleanliness, water quality and chemical balance of public pools shall be certified. To be certified an individual must demonstrate knowledge of public pools. Examples of such knowledge include: pool cleaning, general pool maintenance, make-up water supply, bacteriological, chemical and physical quality of water and water purification, testing, treatment, and disinfection procedures. To ensure that the pool technicians are knowledgeable, said technician shall attend a training course of national recognition that is approved by the department of at least 16 hours in length and shall pass a test acceptable to the department. Certification is conferred upon an individual and is nontransferable. Certification does not imply any licensure and specifically not that of contractor as regulated by the Department of Business and Professional Regulation under Section 489.105(3)(j), (k), or (l), F.S. A certified pool technician may not affect the structural integrity of the pool or equipment, and shall not delegate work to others, including employees, that are not themselves certified under this section, or otherwise exempt from this provision per Chapter 514, F.S.

(1) Training shall include the following study topics for the hours indicated:

(a) Swimming pool calculations 1 hour;

(b) Filter type and filtration circulation 4 hours;

(c) Water chemistry – balancing & testing 5 hours;

(d) Spas and warm water pools 1 hour;

(e) Pool and spa maintenance 2 hours;

(f) Operational and safety requirements 2 hours; and,

(g) State health code Chapter 64E-9, F.A.C., 1 hour.

(2) Course materials must be provided that cover the required topics in detail. The course approval shall be contingent upon their meeting the items listed in subsection (1), above, and the subjects listed in Section 514.075, F.S. The test approval shall be contingent upon all of the questions being related to the subject areas listed in subsection (1), above, and the subjects listed in Section 514.075, F.S., with at least 10% of the questions from the subject areas in paragraphs (1)(a) through (f), above, and the remaining 40% covering any of the seven pool subject areas listed in this rule or Section 514.075, F.S. The minimum passing score for the test shall be no less than 70% correct for all questions. There shall be a minimum of 50 questions.

(3) Any individual or organization requesting the department to review their courses for compliance with the requirements of this rule, must submit copies of their training materials to the department prior to providing that training within the state. A copy of the test to be given, answers to the test questions, and a statement indicating the length of time a classroom topic will be conducted shall be included. The department shall review the materials and inform the applicant of its findings within 60 days from receipt of all training materials.

(4) The department shall deem certified any individual who has been proven certified by a course of national recognition.

(5) This requirement does not apply to a person or the direct employee of a person permitted as a public pool operator under Section 514.031, F.S. Further, persons licensed under Section 489.105(3)(j), (k), or (l), F.S., shall be deemed certified.

(6) Proof of certification shall be posted conspicuously in the equipment room of each pool serviced or must otherwise be

available for inspection by the department.

(7) Any reference to department approval shall state no more than: “This course is approved by the Florida Department of Health for student certification as a Public Pool Service Technician under Chapter 514, F.S., and Chapter 64E-9, F.A.C.”

Rulemaking Authority 381.006, 514.021, 514.075 FS. Law Implemented 514.025, 514.075 FS. History–New 9-25-97, Amended 5-27-04, 5-24-09.

Section 454 Swimming Pools and Bathing Places (Public and Private)

454.1 Public swimming pools and bathing places

Public swimming pools and bathing places shall comply with the design and construction standards of this section.

Exceptions:

1. A portable pool used exclusively for providing swimming lessons or related instruction in support of an established educational program sponsored or provided by a school district may not be regulated as a public pool. Such pool shall be regulated as a private swimming pool under Section 454.2.

2. A temporary pool may not be regulated as a public pool. Such pool shall be regulated as a private swimming pool under Section 454.2.

454.1.1 Flood hazard areas

Public swimming pools installed in flood hazard areas established in Section 1612.3 shall comply with Section 1612.

Note: Other administrative and programmatic provisions apply. See Department of Health (DOH) Rule 64E-9, *Florida Administrative Code* and Chapter 514, *Florida Statutes*. The regulation and enforcement of the initial and annual operationpermit for public pools are preempted to the DOH. The construction permit holder is responsible for obtaining an operation permit issued by DOH, as a public swimming pool shall not be put into operation without an inspection and operation permit issued from the DOH. DOH may grant variances from the provisions of the *Florida Building Code* specifically pertaining to public swimming pools and bathing places as authorized by Section 514.0115, *Florida Statutes*. Building officials shall recognize and enforce variance orders issued by the Department of Health pursuant to Section 514.0115(5), *Florida Statutes* including any conditions attached to the granting of the variance.

"Bathing load" means the maximum number of persons allowed in the pool or bathing place at one time.

"Collector tank" means a reservoir, with a minimum of 2.25 square feet (0.2 m^2) water surface area open to the atmosphere, from which the recirculation or feature pump takes suction, which receives the gravity flow from the main drain line and surface overflow system or feature water source line, and that is cleanable.

"Department" means the permitting/inspection authority.

"Effective barrier" is a barrier which consists of a building, or equivalent structure, plus a 48-inch (1219 mm) minimum height fence on the remaining sides or a continuous 48-inch (1219 mm) minimum height fence. All access through the barrier must have one or more of the following safety features: alarm, key lock or self-locking doors and gates. Safety covers that comply with the American Society for Test Materials standard F1346 may also be considered as an effective barrier.

"D.E." is the Diatomaceous Earth that is used as a filter aid in D.E.-type filters. For the purpose of this rule, it also includes alternative filter aids that have been approved under NSF/ANSI Standard 50, and accepted by the filter manufacturer.

"Interactive water features" means a structure designed to allow for recreational activities with recirculated, filtered, and treated water; but having minimal standing water. Water from the interactive fountain type features is collected by gravity below grade in a collector tank or sump. The water is filtered, disinfected and then pumped to the feature spray discharge heads. The collector tank and water filtration features required make this structure a type of public swimming pool.

"Marking" or "Markings" refers to the placement and installation of visual marking cues to help patrons identify step, bench and swimout outlines, slope break location, depth designations and NO ENTRY and NO DIVING warnings. When markings are specified by code to be dark, the term "dark" shall mean a Munsell color value from zero to four.

"Perimeter overflow gutter" means a level trough or ledge around the inside perimeter of the pool containing drains to clean the pool water surface.

"Plunge pool" means the receiving body of water located at the terminus of a recreational water slide.

"Pool floor" means the interior pool bottom surface which consists of that area from a horizontal plane up to a maximum of a 45-degree slope.

"Pool wall" means the interior pool side surfaces which consist of that area from a vertical plane to a 45-degree slope.

"Pool turnover" means the circulation of the entire pool volume through the filter system. Pool volume shall be determined from the design water level which is the normal operating water level; for gutter-type pools it is the horizontal plane of the upper lip of the gutter and for skimmer pools it is the centerline of the skimmer opening.

Exported from UpCodes

“Portable pool” means a pool or spa, and related equipment systems of any kind, which is designed or intended to be movable from location to location.

“Precoat pot” means a container with a valved connection to the suction side of the recirculation pump of a pressure diatomaceous earth (D.E.) type filter system used for coating the filter with D. E. powder or NSF/ANSI Standard 50-2007 and manufacturer approved substitute filter aid.

A **“public swimming pool” or “public pool”** means a watertight structure of concrete, masonry, or other approved materials which is located either indoors or outdoors, used for bathing or swimming by humans, and filled with a filtered and disinfected water supply, together with buildings, appurtenances, and equipment used in connection therewith. A public swimming pool or public pool shall mean a conventional pool, spa-type pool, wading pool, special purpose pool, interactive water feature or water recreation attraction, to which admission may be gained with or without payment of a fee and includes, but is not limited to, pools operated by or serving camps, churches, cities, counties, day care centers, group home facilities for eight or more clients, health spas, institutions, parks, state agencies, schools, subdivisions, or the cooperative living-type projects of five or more living units, such as apartments, boardinghouses, hotels, mobile home parks, motels, recreational vehicle parks, and townhouses. The term does not include a swimming pool located on the grounds of a private residence.

“Recirculation system” means the system of piping and mechanics designed to remove the water from the pool then filter, disinfect and return it to the pool.

“Slip resistant” means having a textured surface which is not conducive to slipping under contact of bare feet unlike glazed tile or masonry terrazzo and nontextured plastic materials. Manufactured surface products shall be designated by the manufacturer as suitable for walking surfaces in wet areas.

“Spa pool” means a pool used in conjunction with high-velocity air or water.

“Special purpose pool” means a public pool used exclusively for a specific, supervised purpose, including springboard or platform diving training, SCUBA diving instruction, and aquatic programs for persons with disabilities, preschool or kindergarten children.

“Swimming pool slide” is a slide designed by its manufacturer to discharge over the sidewall of a swimming pool.

“Swim spa” is a pool used in conjunction with a directional flow of water against which one swims.

“Sun shelf” means an area of a pool that adjoins the pool wall with a water depth less than 12 inches (305 mm), and is used for seating and play.

“Temporary pool” means a pool intended to be used in conjunction with a sanctioned national or international swimming or diving competition event that does not exceed 30 consecutive days of use.

“Wading pool” means a shallow pool designed to be used by children.

“Water recreation attraction” means a facility with design and operational features that provide patron recreational activity and purposefully involves immersion of the body partially or totally in the water. Water recreation attractions include water slides, river rides, water course rides, water activity pools, interactive water features, wave pools and any additional pool within the boundaries of the attraction.

“Water activity pool” means a water recreation attraction which has water-related activities such as rope ladders, rope swings, cargo nets and other similar activities.

“Water slides” means a water recreation attraction ride which is characterized by having trough-like or tubular flumes or chutes.

“Water theme park” means a complex with controlled access, a fenced and gated attraction where guests enter through a limited number of entrances upon purchase of a ticket. These facilities are permanent and consist of multiple water recreation attractions. Lifeguards are present during all operating hours.

“Water therapy facilities,” as that term is used in Section 514.0115, Item 1, *Florida Statutes*, are pools used exclusively for water therapy to treat a diagnosed injury, illness or medical condition, wherein the therapy is provided under the direct supervision of a Florida licensed physical therapist, occupational therapist or athletic trainer; pursuant to prescription by a physician or a physician’s assistant (PA) licensed pursuant to Chapters 458 or 459, *Florida Statutes*, a podiatrist licensed pursuant to Chapter 461, *Florida Statutes*, or an advanced registered nurse practitioner (ARNP) licensed pursuant to Chapter 464, Florida Statutes; and the prescribing physician, PA, podiatrist or ARNP authorizes a plan of treatment justifying use of the pool for health care purposes.

“Wave pool” means a water recreation attraction that is characterized by wave action.

“Wet deck area” means the 4-foot-wide (1219 mm) unobstructed pool deck area around the outside of the pool water perimeter, curb, ladders, handrails, diving boards, diving towers, or pool slides, waterfalls, water features, starting blocks, planters or lifeguard chairs.

Exported from UpCodes

"Zero depth entry pool" means a pool where the pool floor continues to slope upward to a point where it meets the surface of the water and the pool deck.

454.1.1.1

Sizing

The bathing load for conventional swimming pools, wading pools, interactive water features, water activity pools less than 24 inches (610 mm) deep and special purpose pools shall be computed on the basis of one person per 5 gpm (0.32 L/s) of recirculation flow. The bathing load for spa type pools shall be based on one person per each 10 square feet (0.9 m^2) of surface area. The filtration system for swimming pools shall be capable of meeting all other requirements of these rules while providing a flow rate of at least 1 gpm (0.06 L/s) for each living unit at transient facilities and $^3/_4$ gpm (0.04 L/s) at nontransient facilities. Recreational vehicle sites, campsites and boat slips designated for live-aboards shall be considered a transient living unit. For properties with multiple pools, this requirement includes the cumulative total gpm of allswimming pools, excluding spas, wading pools and interactive water features. All other types of projects shall be sized according to the anticipated bathing load and proposed uses. For the purpose of determining minimum pool size only, the pool turnover period used cannot be less than 3 hours.

454.1.2 Swimming pool construction

standards

454.1.2.1 Pool structure

Pools shall be constructed of concrete or other impervious and structurally rigid material. All pools shall be watertight, free from structural cracks and shall have a nontoxic smooth and slipresistant finish. All materials shall be installed in accordance with manufacturer's specifications unless such specifications violate Chapter 64E-9, *Florida Administrative Code*, rule requirements or the approval criteria of NSF/ANSI Standard 50 or NSF/ANSI Standard 60.

(a) Floors and walls shall be white or pastel in color and shall have the characteristics of reflecting rather than absorbing light. Tile used in less than 5 feet (1524 mm) of water must be slip resistant. A minimum 4-inch (102 mm) tile line, each tile a minimum size of 1 inch (25 mm) on all sides, shall be installed at the water line, but shall not exceed 12 inches (305 mm) in height if a dark color is used. Gutter-type pools may substitute 2-inch (51 mm) tile, each a minimum size of 1 inch (25 mm) on all sides, along the pool wall edge of the gutter lip.

(b) One-inch (25 mm) square tile may be used if the manufacturer has specified the adhesive for use underwater to adhere the type of tile used [vitreous (glass) or ceramic]. Tiles shall not have sharp edges exposed that could cause bather injury.

454.1.2.2 Dimensions

454.1.2.2.1 Dimensional standards

Dimensional standards for competition-type pools shall be those published by the National Collegiate Athletic Association, 1990; Federation Internationale de Natation Amateur (FINA), 1998–2000 Handbook; 1998– 1999 Official Rules of Diving & Code Regulation of United States Diving Inc.; 1998 United States Swimming Rules and Regulations, and National Federation of State High School Associations, 1997–1998, which are incorporated by reference in this code.

454.1.2.2.2 Walls and corners

All pool walls shall have a clearance of 15 feet (4572 mm) perpendicular to thewall (as measured at design water level from gutter lip to gutter lip, or on skimmer pools, from vertical wall to vertical wall). Offset steps spa coves, spa pools and wading pools are exempt from this clearance requirement. Where interior steps protrude into the pool resulting in less than 15 feet (4572 mm) of clearance from any wall, such protrusion shall not exceed 6 feet (1828 mm) on any perpendicular line from a tangent to any pool wall from which the steps emanate. The upper part of pool walls in areas 5 feet deep or less shall be within 5 degrees (4572 mm) vertical for a minimum depth of $2^1/_2$ feet (762 mm) from which point the wall may join the floor with a maximum radius equal to the difference between the pool depth and $2^1/_2$ feet. The upper part of pool walls in areas over 5 feet (1524 mm) deep shall be within 5 degrees vertical for a minimum depth equal to the pool water depth minus $2^1/_2$ feet (762 mm) from which point the wall may join the floor with a maximum radius of $2^1/_2$ feet (762 mm). Corners shall be a minimum 90-degree angle. The corner intersections of walls which protrude or angle into the pool water area shall be rounded with a minimum radius of 2 inches (51 mm). This radius shall be continued through the top of the gutter edge; chamfering is allowed, pool coping shall not overhang into the pool more than $1^1/_2$ inches (38 mm).

454.1.2.2.3 Pool floor slope and slope transition

The radius of curvature between the floor andwalls is excluded from these requirements. Multiple floor levels in pools are prohibited, however, an area meeting all of the requirements of a sun shelf shall not be considered a violation of this requirement.

454.1.2.2.3.1 Floor slope shall be uniform

The floor slope shall be a maximum 1 unit vertical in 10 units horizontal and a minimum of 1 unit vertical in 60 units horizontal in areas 5 feet (1524 mm) deep or less. The floor slope shall be a maximum 1 unit vertical in 3 units horizontal in areas more than 5 feet (1524 mm) deep.

Exported from UpCodes

454.1.2.2.3.2

Any transition in floor slope shall occur at a minimum of 5 feet (1524 mm) of water depth. A slope transition must have a 2 to 6 inch (51 to 152 mm) wide dark contrasting tile marking across the bottom and must extend up both sides of the pool at the transition point. The marking shall be continuous except for recessing grouting. A slope transition must have a safety line mounted by use of recessed cup anchors, 2 feet (610 mm) before the contrasting marking, towards the shallow end. The safety line shall have visible floats at maximum 7-foot (2134 mm) intervals.

454.1.2.2.4 Pool depths

The minimum water depth shall be 3 feet (914 mm) in shallow areas and 4 feet (1219 mm) in deep areas.

454.1.2.3 Markings

454.1.2.3.1 Depth and markings

Depth and markings shall meet the following criteria:

1. The minimum water depth shall be 3 feet (914 mm) in shallow areas and 4 feet (1219 mm) in deep areas.

2. Permanent depth markings followed by the appropriate full or abbreviated words "FEET," "FT," or "INCHES," "IN," shall be installed in minimum 4-inch-high (102 mm) numbers and letters on a contrasting background. Depth markers shall indicate the actual pool depth, within 3 inches (76 mm), at normal operating water level when measured 3 feet (914 m^3) from the pool wall. Symmetrical pool designs with the deep point at the center may be allowed provided a dual marking system is used which indicates the depth at the wall and at the deep point.

3. At a minimum, the markings shall be located on both sides of the pool at the shallow end, slope break, deep-endwall and deep point (if located more than 5 feet (1524 mm) from the deep-end wall). Depth markings shall be legible from inside the pool and also from the pool deck. The maximum perimeter distance between depth markings is 25 feet (7620 mm). Pool size and geometry may necessitate additional depth marking placements about all sides of the pool to meet this requirement.

4. When a curb is provided, the depth markings shall be installed on the inside and outside or top of the pool curb. When a pool curb is not provided, the depth markings shall be located on the inside vertical wall at or above the water level and on the edge of the deck within 2 feet (610 mm) of the pool water. When open type gutter designs are utilized, depth markers shall be located on the back of the gutter wall. When a coping stone with curved or angled underside is provided, the depth markings may be installed on the curved or angled coping underside, and outside or top of the pool curb.

5. When deck level perimeter overflow systems are utilized, additional depth marking signs shall be posted nearby or placed on adjacent fencing or walls and the size shall be increased so they are recognizable from inside theswimming pool. Alternatively, tile depth markers may be placed at the top of the pool wall just under the water level. Depth markers placed on the pool deck shall be within 3 feet (914 mm) of the water.

6. Those areas of the pool that are not part of an approved diving bowl shall have dark contrasting tile, 4-inch-high (102 mm) "NO DIVING" markings installed along the perimeter of the pool on the top of the pool curb or deck within 2 feet (610 mm) of the pool water with a maximum perimeter distance of 25 feet (7620 mm) between markings. A 6-inch (152 mm) tile with a 4-inch (102 mm) or larger red, international "NO DIVING" symbol may be substituted for the "NO DIVING" markings.

7. All markings shall be tile, except that pools constructed of fiberglass, thermoplastic or stainless steel may substitute other type markings when it can be shown that said markings are permanent and will not fade over time. This exemption does not extend to concrete pools that are coated with fiberglass. Tile alternative examples include stone or manufactured plaques with engraved or sandblasted numbers and characters with permanent paint. Permanent appliqués may be used for fiberglass, thermoplastics or stainless steel pools. All markings installed on horizontal surfaces shall have a slip-resistant finish. Markings shall be flush with the surrounding area where placed and recessed if necessary to provide a smooth finish that will avoid creation of an injury hazard to bathers. Pools that are not conducive to tile can employ other equivalent markings as stated above.

454.1.2.3.2 Designs or logos

Any design or logo on the pool floor or walls shall be such that it will not hinder the detection of a human in distress, algae, sediment, or other objects in the pool.

454.1.2.3.3 Lane markings

Pools that are not intended to be utilized for officially sanctioned competition may install lap lane markings provided they meet the following criteria: the markings must be 2 to 6 inches (51 to 152 mm) wide, they must terminate 5 feet (1524 mm) from the end wall in a "T" with the "T" bar at least 18 inches (457.2 mm) long, they must be placed at 7-foot (2134 mm) intervals on center and be no closer than 4 feet (1219 mm) from any side wall, steps or other obstructions. Floating rope lines associated with lap lanes must not obstruct the entrance orexit from the pool and are prohibited when the pool is open for general use.

454.1.2.3.4 Targets

Pools that are not intended for officially sanctioned competition may have 2 to 6 inch (51 to 152 mm) wide 18-inch by 18-inch (457 mm by 457

Exported from UpCodes

mm) targets (+) installed on the pool wall.

454.1.2.3.5 Rules and regulations

signage

Rules and regulations for bathers shall be installed in minimum 1-inch (25.4 mm) letters which must be legible from the pool deck, and shall contain the following:

1. No food or beverages in pool or on pool wet deck.
2. No glass or animals in the fenced pool area (or 50 feet (15 240 mm) from unfenced pool).
3. Bathing load: ___ persons.
4. Pool hours: __ a.m. to __ p.m.
5. Shower before entering.
6. Pools of 200 square feet (18.58 m^2) in area or greater without an approved diving well configuration shall have “NO DIVING”, in 4 inch (102 mm) letters included with the above listed pool rules.
7. Do not swallow the pool water. This statement shall be added to signs at pools that conductalterations as that term is defined.
8. If the pool includes a sun shelf, “WARNING: DROP OFF AT SUN SHELF EDGE IS _×_ FEET DEEP” in 4-inch (102 mm) letters.
9. If the pool includes a sun shelf, “DO NOT PLACE FURNITURE IN POOL.”

454.1.2.4 Color

Pool floors and walls shall be white or light pastel in color and shall have the characteristic of reflecting rather than absorbing light. Theinterior finish coating floors and walls shall be comprised of a nonpigmented white cementitious binder component together with a sand/aggregate component. The finish coating shall have a dry lightness level (CIE L value) of 80.0 or greater and a wet luminous reflectance value (CIE Y value) of 50.0 or greater, as determined by test results provided by the manufacturer, utilizing testing methodology from American Standard ASTM D4086, ASTM E1477, ASTM E1347. Pools constructed of fiberglass, thermoplastic, or stainless steel shall be subject to the same interior finish color requirements. A minimum 4-inch (102 mm) tile line, each tile a minimum size of 1 inch (25 mm) on all sides, shall be installed at the water line, but shall not exceed 12 inches (305 mm) in height if a dark color is used. Gutter-type pools may substitute a 2-inch (51 mm) tile line, each tile a minimum size of 1 inch (25 mm) on all sides, along the pool wall edge of the gutter lip.

454.1.2.5 Access

All pools shall have a means of access every 75 feet (22 860 mm) of pool perimeter with a minimum of two, located so as to serve both ends of the pool. In addition, an access point shall be provided at the deep portion, if the deep portion is not at one end of the pool. When the deep portion of the pool is over 30 feet (9144 mm) wide, both sides of this area shall have a means of access. Access shall consist of ladders, stairs, recessed treads or swimouts and may be used in combination. All treads shall have a slipresistant surface.

454.1.2.5.1 Ladders

Ladders shall be of the crossbraced type and shall be constructed of corrosion-resistant materials and be securely anchored into the pool deck. Clearance between the ladder and pool wall shall be between 3 to 6 inches (76 mm to 152 mm). Ladders shall extend at least 28 inches (711 mm) and no more than 40 inches (1016 mm) above the pool deck. Ladder bottom braces shall have intact end caps or bumpers that rest firmly against the pool wall. The top rung of the ladder shall be at or below the water level on open-gutter pools and not more than 12 inches (305 mm) below the deck or curb top on all other type pools.

454.1.2.5.2 Recessed treads

Recessed treads shall be installed flush with the wall and shall be a minimum 5 inches (127 mm) wide, 10 inches (254 mm) long, with a maximum vertical distance of 12 inches (305 mm) between treads.

454.1.2.5.3 Stairs

Stairs shall have a minimum tread width of 10 inches (254 mm) and a maximum width of 48 inches (1219 mm) for a minimum tread length of 24 inches (610 mm) and a maximum riser height of 10 inches (254 mm). Treads and risers between the top and bottom treads shall be uniform to within $^1/_2$ inch (12.7 mm) in width and height. The riser heights shall be measured at the marked step edges and the differences in elevation shall be considered the riser heights. The front $^3/_4$ to 2 inches (19.1 to 51 mm) of the tread and the top 2 inches (51 mm) of the riser shall be tile, dark in color, contrasting with the interior of the pool. Tile shall be slip resistant. Bullnose tile that is slip resistant may be used when the $^3/_4$-inch (19 mm) segment is placed on the tread or horizontal surface and the 2-inch (51 mm) segment is placed on the riser or vertical surface. Where the gutter is used as the top step, the tile on the gutter for the width of the steps shall be slip resistant. Vinyl liner and fiberglass pools may use other material for the step edge marking, provided the material is permanent, permanently secured, dark in color, nonfading and slip resistant.

Exported from UpCodes

Exception: Where a gutter is used as a top step, the gutter's 2-inch slope from lip to the drain shall be continuous for the full length of the stairs, and the riser from the gutter to the next tread need not be uniform with the remaining risers and treads.

454.1.2.5.4 Swimouts

Swimouts shall extend 18 (457 mm) to 24 inches (610 mm) back from the pool wall, shall be 4 to 5 feet (1219 mm to 1524 mm) wide, shall be a maximum of 12 inches (305 mm) below the deck, unless stairs are provided in the swimout, and shall be located only in areas of the pool greater than 5 feet (1524 mm) deep. Pools that do not utilize a continuous perimeter overflow system must provide a wall return inlet in the swimout for circulation. A permanent dark contrasting colored band of tile shall be installed at the intersection of the pool wall and the swimout and must extend 2 inches (51 mm) on the horizontal and vertical surfaces. Tile must be slip resistant. Bullnose tile may be substituted and installed in accordance with Section 454.1.2.5.3 above.

454.1.2.5.5 Handrails and grabrails

Handrails shall be provided for all stairs, shall be anchored in the bottom step and the deck. Where "figure 4" deck-mounted-typehandrails are used, they shall be anchored in the deck and extend laterally to any point vertically above the bottom step. Grabrails must be mounted in the pool deck at each side of recessed steps. Handrails and grabrails shall extend between 28 and 40 inches (711 mm and 1016 mm) above the step edge and deck. Where stairs are used as an access point between a sun shelf and pool area, ahandrail shall be provided. The hand rail shall be anchored into the bottom step and the sun shelf floor.

454.1.2.5.6 Disabled access

Permanent or portable steps, ramps, handrails, lifts or other devices designed to accommodate handicapped individuals inswimming pools may be provided. Lifts mounted into the pool deck shall have a minimum 4-footwide (1219 mm) deck behind the lift mount.

454.1.2.6 Obstructions

The pool water area shall be unobstructed by any type structure unless justified by engineering design as a part of the recirculation system. Engineering design and material specifications shall show that such structures will not endanger the pool patron, can be maintained in a sanitary condition and will not create a problem for sanitary maintenance of any part of the pool, pool water, or pool facilities. Structures in accord with the above shall not be located in a diving bowl area or within 15 feet (4572 mm) of any pool wall.

Exceptions:

1. Stairs, ladders and ramps, necessary for entrance/exit from the pool are not considered obstructions.
2. Underwater seat benches may be installed in areas less than 5 feet (1524 mm) deep. Bench seats must be 14 to 18 inches (356 to 457 mm) wide and must have a dark contrasting tile marking on the seat edge extending 2 inches (51 mm) on the horizontal and vertical surface. Tile shall be slip resistant. Bullnose tile may be substituted and installed in accordance with Section 454.1.2.5.3. Vinyl liner, stainless steel and fiberglass pools may use other material for the bench edge marking as detailed in Section 454.1.2.3.1, Item 7, provided the material is permanently secured, dark in color, nonfading and slip resistant. Benches shall not protrude into the 15-foot (4572 mm) clearance requirement of Section 454.1.2.6. The bench shall not protrude into the diving bowl.
3. A sun shelf may be installed in pool areas with no more than 4 feet (1219 mm) of water depth, or less. A sun shelf must have a dark contrasting slip-resistant tile marking at the edge of the shelf and the pool wall extending 4 inches (102 mm) from the horizontal shelf edge surface. Additionally, a 2-inch (51 mm) contrasting tile line is required on the vertical pool wall at the edge of the shelf. Vinyl liner, stainless steel and fiberglass pools may use other material for the sun shelf edge marking as detailed in Section 454.1.2.3.1, Item 7, provided the material is permanently secured, dark in color, nonfading and slip resistant. When the edge of a sun shelf uses stairs as a transition, the sun shelf edge tile markings shall comply with step edge requirements as provided in Section 454.1.2.5.3. A sun shelf shall not protrude into the 15-foot (4572 mm) clearance requirement of Section 454.1.2.6. A sun shelf shall not protrude into the diving bowl. A sun shelf must additionally comply with Section 454.1.2.8.

454.1.2.7 Diving areas

Diving facilities shall meet the minimum requirements of the FINAdimensions for diving facilities in accordance with the 2005–2009 FINA Handbook and include the following

1. Diving boards or platforms with heights of less than the established standard shall meet the dimensional requirements of the next greater height.
2. Diving boards, platforms and ladders shall have a nonabsorbent, slip-resistant finish and be of sufficient strength to safely carry the anticipated loads. Diving equipment 1 meter and greater shall have guard rails which extend to the edge of the poolwall. All diving boards over 21 inches (533 mm) from the deck shall be provided with a ladder. Diving boards or platforms shall not be installed on curved walls where the wall enters into the defined rectangular diving area specified in this section. Adjacent platform and diving boards shall be parallel.
3. The location of pool ladders shall be such that the distance from the ladder to any point on a diving board orplatform centerline is not less than the plummet to side wall dimension (b) indicated in the FINA standards. Trampoline-type divingfacilities are prohibited.
4. Diving targets may be installed in accordance with FINA standards.

Exported from UpCodes

454.1.2.8 Sun

shelves

454.1.2.8.1 Sun shelf dimensional requirements

Sun shelf areas must be a minimum of 20 inches (508 mm) wide and provide a minimum of 10 square feet (0.93 m^2) of horizontal surface adjoining on the edge of the pool over a distance of not less than 3 feet (914 mm). The sun shelf floor shall be horizontal or shall a have uniform slope from a zero depth entry, and its maximum depth shall be between 8 inches (203 mm) to 12 inches (254 mm) below the water surface.

454.1.2.8.2 Depth markers at sun

shelves

Where a sun shelf is installed, wet deck-located depth and nodiving markers shall be placed every 20 feet (6096 mm) or less. If the vertical distance between the coping or wet deck and the shelf floor adjacent to the wall is 12 inches (305 mm) or less, these markers shall indicate the water depth of the sun shelf. For open-type gutter pools, the vertical distance shall be measured from the gutter lip to the shelf floor. Where vertical distance between the coping or wet deck and the shelf floor adjacent to the wall is more than 12 inches (305 mm), “No-Entry” markers as described in Section 454.1.9.6.4 shall be provided in the deck. When the sun shelf does not usestairs as a transition, depth markers of the adjacent pool depth at the sun shelf edge and no-dive markers shall be placed on the sun shelf floor, every 10 feet (3048 mm) or less, along a line no more than 1 foot (305 mm) back from the edge of the sun shelf above the deeper pool. All markers shall comply with Items 2, 6 and 7 of Section 454.1.2.3.1, except the distance between them as described in this section shall be followed.

454.1.2.8.3 Access to sun shelf

For the purposes ofSection 454.1.2.5, a sun shelf area shall be considered an entrance to or exit from the pool. If the vertical distance between the coping and the shelf floor adjacent to the wall is more than 10 inches (254 mm), stairs up to the deck or coping shall be provided which shall comply with Sections 454.1.2.5.3 and 454.1.2.5.5; or a zero-depth entry area complying withSection 454.1.9.6 may be provided instead of stairs. For open gutter pools, where the gutter is used as a step, additional steps shall not be required where the distance from the gutter lip to the shelf floor is 10 inches or less. At least one handrail that is compliant with Section 454.1.2.5.5 must be provided at the sun shelf.

454.1.2.8.4 Sun shelf turnover rate

Additional inlets shall be provided in the sun shelf area. The numbers and location shall be such as to ensure the volume of water in the shelf is filtered and chemically treated once every 60 minutes (1 hour) or less.

454.1.3 Pool appurtenances

454.1.3.1 Decks and walkways

454.1.3.1.1

Pool wet decks shall be constructed of concrete or other nonabsorbent material having a smooth slip-resistant finish. Wet deck area finishes shall be designed for such use and shall be installed in accordance with the manufacturer’s specifications. Wooden decks and walkways are prohibited.

454.1.3.1.2

Pool wet decks shall be uniformly sloped at a minimum of 2 percent to a maximum of 4 percent away from the pool or to deck drains to prevent standing water. Textured deck finishes that provide pitting and crevices of more than $^{3}/_{16}$ inch (4.8 mm) deep that accumulate soil are prohibited. If settling or weathering occurs that would cause standing water, the original slopes shall be restored or corrective drains installed. When a curb is provided, the deck shall not be more than 10 inches (254 mm) below the top of the curb.

454.1.3.1.3

Pool wet decks shall have a minimum unobstructed width of 4 feet (1219 mm) around the perimeter of the pool, pool curb, ladders, handrails, diving boards, diving towers and slides.

454.1.3.1.4

Traffic barriers shall be provided as needed so that parked vehicles do not extend over the deck area.

454.1.3.1.5

Walkways shall be provided between the pool and the sanitary facilities, and shall be constructed of concrete or other nonabsorbent material having a smooth slip-resistant finish for the first 15 feet (4572 mm) of the walkway measured from the nearest pool water’s edge. A hose bibb with a vacuum breaker shall be provided to allow the deck to be washed down with potable water.

454.1.3.1.6

Twenty percent of the deck along the pool perimeter may be obstructed as long as any one obstruction does not exceed 10 percent of the pool

Exported from UpCodes

perimeter or 20 feet (6096 mm), whichever is less, in any one area where water depth is 5 feet (1524 mm) or less. Obstructions shall have a wet deck area behind or through them, with the near edge of the walk within 15 feet (4572 mm) of the water except approved slide obstructions shall have the near edge of the walk within 35 feet (10 668 mm) of the water. These obstructions must be protected by a barrier or must be designed to discourage patron access. Obstructions shall not include pool exit points. When an obstruction exists in multiple areas around the pool, the minimum distance between obstructions shall be 4 feet (1219 mm).

454.1.3.1.7

Food or drink service facilities shall not be located within 12 feet (3658 mm) of the water's edge.

454.1.3.1.8

The vertical clearance above the pool deck shall be at least 7 feet (2137 mm).

454.1.3.1.9

All public pools shall be surrounded by a minimum 48 inch (1219 mm) high fence or other approved substantial barrier. The fence shall be continuous around the perimeter of the pool area that is not otherwise blocked or obstructed by adjacent buildings or structures and shall adjoin with itself or abut to the adjacent members. Access through the barrier or fence from dwelling units, such as homes, apartments, motel rooms and hotel rooms, shall be through self-closing, self-latching lockable gates of 48 inch (1219 mm) minimal height from the floor or ground with the latch located a minimum of 54 inches (1372 mm) from the bottom of the gate or at least 3 inches (76 mm) below the top of the gate on the pool side. If the self-closing, self-latching gate is also self-locking and is operated by a key lock, electronic opener or integral combination lock, then the operable parts of such locks or openers shall be 34 inches minimum (864 mm) and 48 inches maximum (1219 mm) above the finished floor or ground. Doored access points from public rooms such as lobbies or club houses need not be through gates if the door(s) meet the same self-closing, self-latching requirements as a gate. Gates shall open outward away from the pool area. A latched, lockable gate shall be placed in the fence within 10 feet (3048 mm) of the closest point between the pool and the equipment area for service access.

Instead of a fence, permanent natural or manmade features such as bulkheads, canals, lakes, navigable waterways, etc., adjacent to a pool may be permitted as a barrier when approved. When evaluating such barrier features, the applicable governing body may perform onsite inspections, and review evidence, such as surveys, aerial photographs, water management agency standards and specifications, and any other similar documentation to verify at minimum, the following: the barrier feature is not subject to natural changes, deviations or alterations and is capable of providing an equivalent level of protection as that provided by a structure, and the barrier feature clearly impedes, prohibits or restricts access to the pool.

Screened pool enclosures must be hardened on the bottom 3 feet (914 mm). Fencing consideration shall be given to the U.S. Consumer Product Safety Commission (CPSC) Publication, No. 362, March 2005, guidelines available from CPSC.gov; or Sections454.2.17.1.1 through 454.2.17.1.8. *Safety covers that comply with ASTM F1346-91 (Reapproved 2003), titled Safety Covers and Labeling Requirements for All Covers for Swimming Pools, Spas and Hot Tubs*, and available from ASTM.org, do not satisfy this requirement.

454.1.3.2 Bridges and overhead obstructions or river rides

Bridges and overhead obstructions over the pool shall be designed so they will not introduce any contamination to the pool water. The minimum height of the bridge or obstruction shall be at least 4 feet (1219 mm) above the surface of the pool in all cases except when the pool is a river ride where it shall be at least 5 feet (1524 mm) above the surface of the pool. Minimum 42-inch-high (1067 mm) handrails shall be provided along each side of the bridge. The walking surfaces shall be constructed of concrete or other nonabsorbent material having a smooth slip-resistant finish. Pool coping shall not overhang into the pool more than $1^1/_2$ inches (38 mm).

454.1.3.3 Safety

454.1.3.3.1

All swimming pools shall be installed with a shepherd's hook securely attached to a one piece pole not less than 16 feet (4880 mm) in length, and at least one 18-inch (457 mm) diameter lifesaving ring with sufficient rope attached to reach all parts of the pool from the pool deck. Safety equipment shall be mounted in a conspicuous place and be readily available for use. Pools greater than 50 feet (15 250 mm) in length shall have multiple units with at least one shepherd's hook and one lifesaving ring located along each of the longer sides of the pools. Spa pools under 200 square feet (1.86 m^2) of surface area, and interactive water features or wading pools with 2 feet (610 mm) or less of water depth are exempt from this requirement.

454.1.3.3.2

All pools with a slope transition shall have safety line anchors as required by Section 454.1.2.2.3.2.

454.1.3.3.3

If a pool cover or solar blanket is installed, it shall be secured around the entire perimeter and designed to support a live load of an adult person; or, the pool area shall be inaccessible to unauthorized individuals during times of cover or blanket use.

454.1.3.3.4

A room or space shall be provided for chemicals to be stored in a cool, dry, and well-ventilated area under a roof and the area shall be inaccessible to the public.

Exported from UpCodes

454.1.3.3.5

Swimming pool slides shall be installed in accordance with manufacturer's specifications and sound engineering practice. Pools with slides designed for swimming pools are not required to satisfy those of slide plunge pools inSection 454.1.9.2.1.

454.1.3.3.6

Floating and climb-on devices, objects or toys that are not a part of the approved pool design shall not be tethered in the pool or installed without an engineering alterations application.

454.1.4 Electrical systems

454.1.4.1 Electrical equipment and wiring

Electrical equipment wiring and installation, including the bonding and grounding of pool components shall comply with Chapter 27 of the *Florida Building Code, Building*. Outlets supplying pool pump motors connected to single-phase 120-volt through 240-volt branch circuits, whether by receptacle or by direct connection, and outlets supplying other electrical equipment and underwater luminaires operating at voltages greater than the low voltage contact limit, connected to single-phase, 120 volt through 240 volt branch circuits, rated 15 or 20 amperes, whether by receptacle or by direct connection, shall be provided with ground-fault circuit interrupter protection for personnel.

454.1.4.2 Lighting

Artificial lighting shall be provided at all swimming pools which are to be used at night or which do not have adequate natural lighting so that all portions of the pool, including the bottom, may be readily seen without glare.

454.1.4.2.1 Outdoor pool

lighting

Lighting shall provide a minimum of 3 footcandles (30 lux) of illumination at the pool water surface and the pool wet deck surface. Underwater lighting shall be a minimum of $^1/_2$ watt per square foot of pool water surface area.

454.1.4.2.2 Indoor pool

lighting

Lighting shall provide a minimum of 10 foot candles (100 lux) of illumination at the pool water surface and the pool wet deck surface. Underwater lighting shall be a minimum of $^8/_{10}$ watt per square foot of pool surface area.

454.1.4.2.3 Underwater

lighting

Underwater luminaires shall comply with Chapter 27 of the *Florida Building Code, Building*. The location of the underwater luminaires shall be such that the underwater illumination is as uniform as possible. Underwater lighting requirements can be waived when the overhead lighting provides at least 15 footcandles (150 lux) of illumination at the pool water surface and pool wet deck surface.

454.1.4.2.4 Overhead wiring

Overhead service wiring shall not pass within an area extending a distance of 10 feet (3048 mm) horizontally away from the inside edge of the pool walls, diving structures, observation stands, towers or platforms. Allowances for overhead conductor clearances to pools that meet the safety standards in the *National Electrical Code* may be used instead. Electrical equipment wiring and installation including the grounding of pool components shall comply with Chapter 27 of the *Florida Building Code, Building*.

454.1.5 Equipment area or rooms

454.1.5.1 Equipment

Equipment designated by the manufacturer for outdoor use may be located in an equipment area, all other equipment must be located in an equipment room or enclosure. Plastic pipe subject to a period of prolonged sunlight exposure must be coated to protect it from ultraviolet light degradation. An equipment area shall be surrounded with a fence at least 4 feet (1219 mm) high on all sides not confined by a building or equivalent structure. A self-closing and self-latching gate with a permanent locking device shall be provided if necessary for access. An equipment room shall be protected on at least three sides and overhead. Any fence or gate installed shall use members spacing that shall not allow passage of a 4-inch (102 mm) diameter sphere. The fourth side may be a gate, fence, or open if otherwise protected from unauthorized entrance. An equipment enclosure shall be lockable or otherwise protected from unauthorized access.

454.1.5.2 Indoor equipment

Equipment not designated by the manufacturer for outdoor use shall be located in an equipment room. An equipment room shall be protected on at least three sides and overhead. The fourth side may be a gate, fence or open if otherwise protected from unauthorized entrance.

454.1.5.3 Materials

The equipment enclosure, area or room floor shall be of concrete or other nonabsorbent material having a smooth slip-resistant finish and shall

Exported from UpCodes

have positive drainage, including a sump pump if necessary. Ancillary equipment, such as a heater, not contained in an equipment enclosure or room shall necessitate an equipment area as described above.

454.1.5.4 Ventilation

Equipment rooms shall have either forced draft or cross ventilation. All below-grade equipment rooms shall have a stairway access with forced draft ventilation or a fully louvered door and powered intake within 6 inches (152 mm) of the floor. Where stairway access is not necessary to carry heavy items into the below grade room or vault, a "ship's ladder" may be used if specified by the design engineer who must consider anticipated workload including equipment removal; and the ladder slope, tread height and width; and construction material of the ladder.

454.1.5.5 Access

The opening to an equipment room or area shall be a minimum 3 feet by 6 feet (914 mm by 1829 mm) and shall provide easy access to the equipment.

454.1.5.6 Size

The size of the equipment enclosure, room or area shall provide working space to perform routine operations. Clearance shall be provided for all equipment as prescribed by the manufacturer to allow normal maintenance operation and removal without disturbing other piping or equipment. In rooms with fixed ceilings, the minimum height shall be 7 feet (2137 mm).

454.1.5.7 Lighting

Equipment rooms or areas shall be lighted to provide 30 footcandles (300 lux) of illumination at floor level.

454.1.5.8 Storage

Equipment enclosures, rooms or areas shall not be used for storage of chemicals emitting corrosive fumes or for storage of other items to the extent that entrance to the room for inspection or operation of the equipment is impaired.

454.1.5.9 Hose

bibbs

A hose bibb with vacuum breaker shall be located in the equipment room or area.

454.1.6 Plumbing systems

454.1.6.1 Sanitary facilities

Swimming pools with a bathing load of 20 persons or less may utilize a unisex restroom. Pools with bathing loads of 40 persons or less may utilize two unisex restrooms or meet the requirements of Table 454.1.6.1. Unisex restrooms shall meet all the requirements for materials, drainage and signage as indicated in Sections 454.1.6.1.1 through 454.1.6.1.4. Each shall include a water closet, a diaper change table, a urinal and a lavatory. Pools with a bathing load larger than 40 persons shall provide separate sanitary facilities labeled for each sex. The entry doors of all restrooms shall be located within a 200-foot (60 960 mm) walking distance of the nearest water's edge of each pool served by the facilities.

Exception: Where a swimming pool serves only a designated group of residential dwelling units and not the general public, poolside sanitary facilities are not required if all living units are within a 200-foot (60 960 mm) horizontal radius of the nearest water's edge, are not over three stories in height unless serviced by an elevator, and are each equipped with private sanitary facilities.

TABLE 454.1.6.1

PUBLIC SWIMMING POOL—REQUIRED FIXTURE COUNT

SIZE OF POOL (square feet)	MEN'S RESTROOM			WOMEN'S RESTROOM	
	Urinals	WC	Lavatory	WC	Lavatory
0 – 2,500	1	1	1	1	1
2,501 – 5,000	2	1	1	5	1
5,001 – 7,500	2	2	2	6	2
7,501 – 10,000	3	2	3	8	3

For SI: 1 square foot = 0.0929 m^2.

454.1.6.1.1 Required fixtures

Fixtures shall be provided as indicated on Table 454.1.6.1. The fixture count on this chart is deemed to be adequate for the pool and pool deck area that is up to three times the area of the pool surface provided. When multiple fixture sets are required and separate facilities are provided for each sex, the fixtures used in ancillary family-style restrooms can be used to meet the requirements of this section.

One diaper changing table shall be provided at each restroom. Diaper changing tables are not required at restrooms where all pools served are restricted to adult use only. Swim diapers are recommended for use by children that are not toilet trained. Persons that are ill with

Exported from UpCodes

diarrhea cannot enter the pool.

Exception: When a public swimming pool meets all of the following conditions the following shall apply:

1. The pool serves only a designated group of dwelling units,
2. The pool is not for the use of the general public, and
3. A building provides sanitary facilities;

The fixture requirement for the building shall be determined and if it exceeds the requirement inTable 454.1.6.1 then the building requirement shall regulate the fixture count, otherwise the fixture count shall be based on the requirement for the pool. Under no circumstances shall the fixture counts be cumulative.

An additional set of fixtures shall be provided in the men's restroom for every 7,500 square feet (697 m^2) or major fraction thereof for pools greater than 10,000 square feet (929 m^2).

Women's restrooms shall have a ratio of three to two water closets provided for women as the combined total of water closets and urinals provided for men.

Lavatory counts shall be equal.

454.1.6.1.2 Outside access

Outside access to facilities shall be provided for bathers at outdoor pools. Where the restrooms are located within an adjacent building and the restroom doors do not open to the outside, the restroom doors shall be within 50 feet (15 240 mm) of the building's exterior door. If the restrooms are not visible from any portion of the pool deck, signs shall be posted showing directions to the facilities. Directions shall be legible from any portion of the pool deck; letters shall be a minimum of 1 inch (25 mm) high.

454.1.6.1.3 Sanitary facility floors

Floors of sanitary facilities shall be constructed of concrete or other nonabsorbent materials, shall have a smooth, slip-resistant finish, and shall slope to floor drains. Carpets, duckboards and footbaths are prohibited. The intersection between the floor and walls shall be coved where either floor or wall is not made of waterproof materials such as tile or vinyl.

454.1.6.1.4 Hose

bibb

A hose bibb with vacuum breaker shall be provided in or within 25 feet (7620 mm) of each restroom to allow for ease of cleaning.

454.1.6.2 Rinse

shower

A minimum of one rinse shower shall be provided on the pool deck of all outdoor pools within the perimeter of the fence.

454.1.6.3 Cross-connection prevention

An atmospheric break or approved backflow prevention device shall be provided in each pool water supply line that is connected to a public water supply. Vacuum breakers shall be installed on all hose bibbs.

454.1.6.4 Plastic

pipes

Plastic pipe subject to a period of prolonged sunlight exposure shall be coated to protect it from ultraviolet light degradation.

454.1.6.5 Recirculation and treatment systems

454.1.6.5.1 Equipment testing

Recirculation and treatment equipment such as filters, recessed automatic surface skimmers, ionizers, ozone generators, disinfection feeders and chlorine generators shall be tested and approved using the NSF/ANSI Standard 50, Circulation System Components and Related Materials for Swimming Pool, Spas/Hot Tubs, dated April 2007, which is incorporated by reference.

454.1.6.5.2 Volume

The recirculation system shall be designed to provide a minimum of four turnovers of the pool volume per day. Pools that are less than 1,000 square feet (93 m^2) at health clubs shall be required to provide eight turnovers per day.

454.1.6.5.3 System

Exported from UpCodes

design

The design pattern of recirculation flow shall be 100 percent through the main drain piping and 100 percent through the perimeter overflow system or 60 percent through the skimmer system.

454.1.6.5.3.1 Perimeter overflow gutters

The lip of the gutter shall be uniformly level with a maximum tolerance of $^1/_4$ inch (6 mm) between the high and low areas. The bottom of the gutter shall be level or slope to the drains. The spacing between drains shall not exceed 10 feet (3048 mm) for 2-inch (51 mm) drains or 15 feet (4572 mm) for $2^1/_2$-inch (64 mm) drains, unless hydraulically justified by the design engineer. Gutters may be eliminated along pool edges for no more than 15 feet (4572 mm) and this shall not exceed 10 percent of the perimeter (at least 90 percent of the perimeter shall be guttered). In areas where gutters are eliminated, handholds shall be provided within 9 inches (229 mm) of the water surface. Handhold design shall be approved by the jurisdictional building department prior to construction.

454.1.6.5.3.1.1

Either recessed-type or opentype gutters shall be used. Special designs can be approved provided they are within limits of sound engineering practice. Recessed-type gutters shall be at least 4 inches (102 mm) deep and 4 inches (102 mm) wide. No part of the recessed gutter shall be visible from a position directly above the gutter sighting vertically down the edge of the deck or curb. Open-type gutters shall be at least 6 inches (150 mm) deep and 12 inches (305 mm) wide. The gutter shall slope 2 inches (51 mm), +/-$^1/_4$ inch (+/-6 mm), from the lip to the drains. The gutter drains shall be located at the deepest part of the gutter.

454.1.6.5.3.1.2

All gutter systems shall discharge into a collector tank.

454.1.6.5.3.1.3

The gutter lip shall be tiled with a minimum of 2-inch (51 mm) tile on the poolwall, each a minimum size of 1 inch (25 mm) on all sides. The back vertical wall of the gutter shall be tiled with glazed tile. All tile used on the flat, horizontal part, or the leading edge of an open-type gutter, must be slip resistant.

Exception: Stainless steel gutter systems when it can be shown that the surfaces at the waterline and back of the gutter are easily cleanable.

454.1.6.5.3.2 Recessed automatic surface skimmers

Recessed automatic surface skimmers may be utilized when the pool water surface area is 1,000 square feet (93 m^2) or less excluding offset stairs and swimouts and the width of the pool is not over 20 feet (6096 mm).

454.1.6.5.3.2.1 Volume

The recessed automatic surface skimmer piping system shall be designed to carry 60 percent of the pool total design flow rate with each skimmer carrying a minimum 30 gpm (2 L/s). One skimmer for every 400 square feet (37 m^2) or fraction thereof of pool water surface area shall be provided.

454.1.6.5.3.2.2 Location

Prevailing wind direction and the pool outline shall be considered by the designer in the selection of skimmer locations. The location of skimmers shall be such that the interference of adjacent inlets and skimmers is minimized. Recessed automatic surface skimmers shall be installed so that there is no protrusion into the pool water area. The deck or curb shall provide for a handhold around the entire pool perimeter and shall not be located more than 9 inches (229 mm) above the midpoint of the opening of the skimmer.

454.1.6.5.3.2.3 Equalizers

Recessed automatic surface skimmers shall be installed with an equalizer valve and an equalizer line when the skimmer piping system is connected directly to pump suction. If installed, the equalizer valve shall be a spring-loaded vertical check valve which will not allow direct suction on the equalizer line. Float valves are prohibited. The equalizer line inlet shall be installed at least 1 foot (305 mm) below the norma pool water level and the equalizer line inlet shall be protected by an ASME/ANSI A112.19.8 compliant cover/grate. The equalizer line shall be sized to handle the expected flow with a 2-inch (51 mm) minimum line size.

454.1.6.5.3.2.4 Wall-inlet fitting

A wall-inlet fitting shall be provided directly across from each skimmer.

454.1.6.5.3.2.5 Waterline tile

A minimum 6-inch (152 mm) water line tile shall be provided on all pools with automatic skimmer systems, each a minimum size of 1 inch (25 mm) on all sides. Glazed tile that is smooth and easily cleanable shall be utilized.

454.1.6.5.4 Pumps

If the pump or suction piping is located above the water level of the pool, the pump shall be self-priming. Pumps that take suction prior to filtration shall be equipped with a hair and lint strainer. The recirculation pump shall be selected to provide the required recirculation flow

Exported from UpCodes

against a minimum total dynamic head of 60 feet (18 288 mm) unless hydraulically justified by the design engineer. Vacuum D.E. filter system pumps shall provide at least 50 feet (15 240 mm) of total dynamic head. Should the total dynamic head required not be appropriate for a given project, the design engineer shall provide an alternative.

454.1.6.5.5 Filters

Filters sized to handle the required recirculation flow shall be provided.

454.1.6.5.5.1 Filter capacities

The maximum filtration rate in gallons per minute per square foot of filter area shall be: 15 [20 if so approved using the procedure stated in Section 454.1.6.5.1 for high rate sand filters, 3 for rapid sand filters, 0.375 for pleated cartridge filters and 2 for Diatomaceous Earth (D.E.) type filters].

454.1.6.5.5.2 Filter appurtenances

454.1.6.5.5.2.1 Pressure filter systems

Pressure filter systems shall be equipped with an air relief valve, influent and effluent pressure gauges with minimum face size of 2 inches (51 mm) reading 0–60 psi (0–414 kPa), and a sight glass when a backwash line is required.

454.1.6.5.5.2.2 Vacuum filter systems

Vacuum filter systems shall be equipped with a vacuum gauge which has a 2-inch (51 mm) face and reads from 0–30 inches of mercury.

454.1.6.5.5.2.3 D.E.

systems

A precoat pot or collector tank shall be provided for D.E.-type systems.

454.1.6.5.5.3 Filter tanks and elements

The filter area shall be determined on the basis of effective filtering surfaces with no allowance given for areas of impaired filtration, such as broad supports, folds, or portions which may bridge. D.E.- type filter elements shall have a minimum 1-inch (25 mm) clear spacing between elements up to a 4 square foot (0.4 m^2) effective area. The spacing between filter elements shall increase $^1/_8$ inch (3 mm) for each additional square foot of filter area or fraction thereof above an effective filter area of 4 square feet (0.4 m^2). All cartridges used in public pool filters shall be permanently marked with the manufacturer’s name, pore size and area in square feet of filter material. All cartridges with end caps shall have the permanent markings on one end cap. Vacuum filter tanks shall have coved intersections between the wall and the floor and the tank floor shall slope to the filter tank drain. The D.E.-type filter tank and elements shall be installed such that the recirculation flow draw down does not expose the elements to the atmosphere whenever only the main drain valve is open or only the surface overflow gutter system valve is open.

454.1.6.5.6 Piping

All plastic pipe used in the recirculation system shall be imprinted with the manufacturer’s name and the NSF-pw logo for potable water applications. Size, schedule and type of pipe shall be included on the drawings. Plastic pipe subject to a period of prolonged sunlight exposure shall be coated to protect it from ultraviolet light degradation.

454.1.6.5.7 Valves

Return lines, main drain lines, and surface overflow system lines, shall each have proportioning valves.

454.1.6.5.8 Flow velocity

Pressure piping shall not exceed 10-feet per second (2038 mm/s), except that precoat lines with higher velocities may be used when necessary for agitation purposes. The flow velocity in suction piping shall not exceed 6-feet per second (1829 mm/s) except that flow velocities up to 10-feet per second (3048 mm/s) in filter assembly headers will be acceptable. Main drain systems and surface overflow systems which discharge to collector tanks shall be sized with a maximum flow velocity of 3-feet per second (914 mm/s). The filter and vacuuming system shall have the necessary valves and piping to allow filtering to pool, vacuuming to waste, vacuuming to filter, complete drainage of the filter tank, backwashing for sand and pressure D.E.- type filters and precoat recirculation for D.E.-type filters.

454.1.6.5.9 Inlets

All inlets shall be adjustable with wall-type inlets being directionally adjustable and floor-type inlets having a means of flow adjustment. Floor inlets shall be designed and installed such that they do not protrude above the pool floor and all inlets shall be designed and installed so as not to constitute sharp edges or protrusions hazardous to pool bathers. Floor inlets for vinyl liner and fiberglass pools, shall be smooth with no sharp edges, and shall not extend more than $^3/_8$ inch (9.5 mm) above the pool floor. Wall inlets shall be installed a minimum of 12 inches (305 mm) below the normal operating water level unless precluded by the pool depth or intended for a specific acceptable purpose.

454.1.6.5.9.1

Pools 30 feet (9144 mm) in width or less, with wall inlets only shall have enough inlets such that the inlet spacing does not exceed 20 feet (6096 mm) based on the pool water perimeter.

Exported from UpCodes

454.1.6.5.9.2

Pools 30 feet (9144 mm) in width or less with floor inlets only shall have a number of inlets provided such that the spacing between adjacent inlets does not exceed 20 feet (6096 mm) and the spacing between inlets and adjacent walls does not exceed 10 feet (3048 mm).

454.1.6.5.9.3

A combination of wall and floor inlets may be used in pools 30 feet (9144 mm) in width or less only if the requirements ofSection 454.1.6.5.9.1 or Section 454.1.6.5.9.2 are fully met.

454.1.6.5.9.4

Pools greater than 30 feet (9144 mm) in width shall have either floor inlets only, or a combination of floor inlets andwall inlets. Pools with floor inlets only shall have a number of floor inlets provided such that the spacing between adjacent inlets does not exceed 20 feet (6096 mm) and the spacing between inlets and an adjacent wall does not exceed 10 feet (3048 mm).

454.1.6.5.9.5

Pools greater than 30 feet (9144 mm) in width with a combination of wall and floor inlets shall have the number of wall inlets such that the maximum spacing between the wall inlets is 20 feet (6096 mm) and floor inlets are provided for the pool water area beyond a 15 feet (4572 mm) perpendicular distance from all walls. The number of floor inlets shall be such that the spacing between adjacent inlets does not exceed 20 feet (6096 mm) and the distance from a floor inlet and an adjacent wall does not exceed 25 feet (7620 mm). Floor inlets shall be designed and installed such that they do not protrude more than $^5/_8$ inch (16 mm) above the pool floor and all inlets shall be designed and installed so as not to constitute sharp edges or protrusions hazardous to pool bathers.

454.1.6.5.9.6

The flow rate through each inlet shall not exceed 20 gpm (1 L/s).

454.1.6.5.10 Main drain

outlets

All pools shall be provided with an outlet at the deepest point.

454.1.6.5.10.1

The depth at the outlet shall not deviate more than 3 inches (76 mm) from the side wall.

454.1.6.5.10.2

Outlets shall be covered by a secured grating which requires the use of a tool to remove and whose open area is such that the maximum velocity of water passing through the openings does not exceed $1^1/_2$ feet per second (457 mm/s) at 100 percent of the design recirculation flow. Main drain covers/grates shall comply with the requirements of ANSI/APSP 16 and the water velocity of this section.

454.1.6.5.10.3

Multiple outlets, equally spaced from the pool side walls and from each other, shall be installed in pools where the deep portion of the pool is greater than 30 feet (9144 mm) in width.

454.1.6.5.10.4

If the area is subject to high ground water, the pool shall be designed to withstand hydraulic uplift or shall be provided with hydrostatic relief devices.

454.1.6.5.10.5

The main drain outlet shall be connected to a collector tank. The capacity of the collector tank shall be at least 1 minute of the recirculated flow unless justified by the design engineer. Vacuum filter tanks are considered collector tanks.

454.1.6.5.11 Water makeup control

An automatic and manual water makeup control shall be provided to maintain the water level at the lip of the overflow gutter or at the mouth of the recessed automatic surface skimmers and shall discharge through an air gap into a fill pipe or collector tank. Over the rim fill spouts are prohibited.

454.1.6.5.12 Cleaning system

A portable or plumbed in vacuum cleaning system shall be provided. All vacuum pumps shall be equipped with hair and lint strainers. When the system is plumbed in, the vacuum fittings shall be located to allow cleaning the pool with a 50-foot (15 240 mm) maximum length of hose. Vacuum fittings shall be mounted no more than 15 inches (381 mm) below the water level, flush with the pool walls, and shall be provided with a spring-loaded safety cover which shall be in place at all times when the pool is not being vacuumed. Bag-type cleaners, which operate as ejectors on potable water supply pressure, shall be protected by a vacuum breaker. Cleaning devices shall not be used while the pool is open to bathers.

454.1.6.5.13 Rate of flow

indicators

A rate of flow indicator, reading in gpm, shall be installed on the return line. The rate of flow indicator shall be properly sized for the design flow rate and shall be capable of measuring from one-half to at least one-andone-half times the design flow rate. The clearances upstream and downstream from the rate of flow indicator shall comply with manufacturer's installation specifications.

454.1.6.5.14 Heaters

Pool heaters shall comply with nationally recognized standards acceptable to the jurisdictional building department and to the design engineer. Pools equipped with heaters shall have a fixed thermometer mounted in the pool recirculation line downstream from the heater outlet. Thermometers mounted on heater outlets do not meet this requirement. A sketch of any proposed heater installation including valves, thermometer, pipe sizes, and material specifications shall be included in the application for permit prior to installation. Piping and influent, effluent and bypass valves which allow isolation or removal of the heater from the system shall be provided. Materials used in solar and other heaters shall be nontoxic and acceptable for use with potable water. Heaters shall not prevent the attainment of the required turnover rate.

454.1.6.5.15 Pool waste water disposal

Pool waste water shall be discharged through an air gap; disposal shall be to sanitary sewers, storm sewers, drainfields, or by other means, in accordance with local requirements including obtaining all necessary permits. Disposal of water from pools using D.E. powder shall be accomplished through separation tanks which are equipped with air bleed valves, bottom drain lines, and isolation valves, or through a settling tank with final disposal being acceptable to local authorities. D.E. separator tanks shall have a capacity as rated by the manufacturer, equal to the square footage of the filter system. All lines shall be sized to handle the expected flow. There shall not be a direct physical connection between any drain from a pool or recirculation system and a sewer line.

454.1.6.5.16 Addition of chemicals

Disinfection and ph adjustment shall be added to the pool recirculation flow using automatic feeders meeting the requirement of ANSI/NSF 50. All chemicals shall be fed into the return line after the pump, heater and filters unless the feeder was designed by the manufacturer and approved by the NSF to feed to the collector tank or to the suction side of the pump.

454.1.6.5.16.1 Gas

chlorination

When gas chlorination is utilized, the chlorinator shall be capable of continuously feeding a chlorine dosage of 6 mg/L to the recirculated flow of the filtration system. The application point for chlorine shall be located in the return line downstream of the filter, recirculation pump, heater, and flow meter, and as far as possible from the pool.

454.1.6.5.16.1.1

Gas chlorinators shall be located in above-grade rooms and in areas which are inaccessible to unauthorized persons.

454.1.6.5.16.1.1.1

Chlorine rooms shall have: continuous forced draft ventilation capable of a minimum of one air change per minute with an exhaust at floor level to the outside, a minimum of 30 footcandles (300 lux) of illumination with the switch located outside and the door shall open out and shall not be located adjacent to the filter room entrance or the pool deck. A shatterproof gas-tight inspection window shall be provided.

454.1.6.5.16.1.1.2

Chlorine areas shall have a roof and shall be enclosed by a chain-link-type fence at least 6 feet (1829 mm) high to allowventilation and prevent vandalism.

454.1.6.5.16.1.2

When booster pumps are used with the chlorinator, the pump shall use recirculated pool water supplied via the recirculation filtration system. The booster pump shall be electrically interlocked with the recirculation pump to prevent the feeding of chlorine when the recirculation pump is not operating.

454.1.6.5.16.1.3

A means of weighing chlorine containers shall be provided. When 150-pound (68 kg) cylinders are used, platformtype scales shall be provided and shall be capable of weighing a minimum of two full cylinders at one time. The elevation of the scale platform shall be within 2 inches (51 mm) of the adjacent floor level, and the facilities shall be constructed to allow easy placement of full cylinders on the scales.

454.1.6.5.16.2 Hypohalogenation and electrolytic chlorine

generators

The hypohalogenation-type feeder and electrolytic chlorine generators shall be adjustable from 0 to full range. A rate of flow indicator is required on erosion-type feeders. The feeders shall be capable of continuously feeding a dosage of 6 mg/L to the minimum required turnover flow rate of the filtration systems. Solution feeders shall be capable of feeding the above dosage using a 10-percent sodium hypochlorite solution, or 5-percent calcium hypochlorite solution, whichever disinfectant is to be utilized at this facility. To prevent the disinfectant from siphoning or feeding directly into the pool or pool piping under any type failure of the recirculation equipment, an electrical interlock with the recirculation pump shall be incorporated into the system for electrically operated feeders. The minimum size of the

Exported from UpCodes

solution reservoirs shall be at least 50 percent of the maximum daily capacity of the feeder. The solution reservoirs shall be marked to indicate contents.

454.1.6.5.16.3 Feeders for pH

adjustment

Feeders for pH adjustment shall be provided on all pools. pH adjustment feeders shall be positive displacement type, shall be adjustable from 0 to full range, and shall have an electrical interlock with the circulation pump to prevent discharge when the recirculation pump is not operating. When soda ash is used for pH adjustment, the maximum concentration of soda ash solution to be fed shall not exceed $^{1}/_{2}$-pound (0.2 kg) soda ash per gallon of water. Feeders for soda ash shall be capable of feeding a minimum of 3 gallons (11 L) of the above soda ash solution per pound of gas chlorination capacity. The minimum size of the solution reservoirs shall not be less than 50 percent of the maximum daily capacity of the feeder. The solution reservoirs shall be marked to indicate the type of contents.

454.1.6.5.16.4 Ozone generating equipment

Ozone generating equipment may be used for supplemental water treatment on public swimming pools subject to the conditions of this section.

454.1.6.5.16.4.1

Ozone generating equipment electrical components and wiring shall comply with the requirements ofChapter 27 of the *Florida Building Code, Building* and the manufacturer shall provide a certificate of conformance. The process equipment shall be provided with an effective means to alert the user when a component of this equipment is not operating.

454.1.6.5.16.4.2

Ozone generating equipment shall meet the NSF/ANSI Standard 50.

454.1.6.5.16.4.3

The concentration of ozone in the return line to the pool shall not exceed 0.1 mg/L.

454.1.6.5.16.4.4

The injection point for ozone generating equipment shall be located in the pool return line after the filtration and heating equipment, prior to the halogen injection point, and as far as possible from the nearest pool return inlet with a minimum distance of 4 feet (1219 mm). Injection methods shall include a mixer, contact chamber, or other means of efficiently mixing the ozone with the recirculated water. The injection and mixing equipment shall not prevent the attainment of the required turnover rate of the recirculation system. Ozone generating equipment shall be equipped with a check valve between the generator and the injection point. Ozone generating equipment shall be equipped with an air flow meter and a means to control the flow. The generator shall be electrically interlocked with the recirculation pump to prevent the feeding of ozone when the recirculation pump is not operating. A flow sensor controller can also be used to turn off the feeder when flow is sensed.

454.1.6.5.16.4.5 Ventilation requirements

Ozone generating equipment shall be installed in equipment rooms with either forced draft or cross draftventilation. Below-grade equipment rooms with ozone generators shall have forced draft ventilation and all equipment rooms with forced draft ventilation shall have the fan control switch located outside the equipment room door. The exhaust fan intake for forced draft ventilation and at least one vent grille for cross draft ventilation shall be located at floor level.

454.1.6.5.16.4.6

A self-contained breathing apparatus designed and rated by its manufacturer for use in ozone contaminated air shall be provided when ozone generator installations are capable of exceeding the maximum pool water ozone contact concentration of 0.1 mg/L. The self-contained breathing apparatus shall be available at all times and shall be used at times when the maintenance or service personnel have determined that the equipment room ozone concentration exceeds 10 mg/L. Ozone generator installations which require the self-contained breathing apparatus shall also be provided with Draeger-type detector tube equipment which is capable of detecting ozone levels of 10 mg/L and greater.

Exception: In lieu of the self-contained breathing apparatus an ozone detector capable of detecting 1 mg/L may be used. Said detector shall be capable of stopping the production of ozone, venting the room and sounding an alarm once ozone is detected.

454.1.6.5.16.5

Ionization units may be used as supplemental water treatment on public pools subject to the condition of this section.

454.1.6.5.16.5.1

Ionization equipment and electrical components and wiring shall comply with the requirements ofChapter 27 of the *Florida Building Code, Building* and the manufacturer shall provide a certification of conformance.

454.1.6.5.16.5.2

Ionization equipment shall meet the NSF/ANSI Standard 50, Circulation *System Components and Related Materials for Swimming Pools,*

Exported from UpCodes

Spas/Hot Tubs, or equivalent, shall meet UL standards and shall be electrically interlocked with a recirculation pump.

454.1.6.5.16.6

Ultraviolet (UV) light disinfectant equipment may be used as supplemental water treatment on public pools [and additional treatment on interactive water features (IWFs)] subject to the conditions of this paragraph and manufacturer's specifications. UV is encouraged to be used to eliminate or reduce chlorine-resistant pathogens, especially the protozoan cryptosporidium.

1. UV equipment and electrical components and wiring shall comply with the requirements of the *National Electrical Code* and the manufacturer shall provide a certification of conformance to the jurisdictional building department.

2. UV equipment shall meet UL standards and shall be electrically interlocked with recirculation pump(s) on all pools and with feature pumps(s) on an IWF such that when the UV equipment fails to produce the required dosage as measured by an automated sensor, the feature pump(s) are disabled so the water features do not operate.

3. UV equipment used in higher risk facilities such as interactive water features, wading pools, and activity pools shall be validated by a capable party that it delivers the required and predicted UV dose at the validated flow, lamp power and water UV transmittance conditions, and has complied with all professional practices summarized in the *USEPA Ultraviolet Disinfectant Guidance Manual* dated November 2006, which is publication number EPA 815-R-06-007 available from the department at http://www.floridashealth.org/Environment/water/swim/ index.html or at http://www.epa.gov/safewater/disinfection/lt2/pdfs/guideit2_uguidance.pdf.

   Exception: Not applicable when Section 454.1.9.8.6.1 alternative is used.

4. UV equipment shall constantly produce a validated dosage of at least 40 mJ/cm^2 (millijoules per square centimeter) at the end of lamp life.

5. The UV equipment shall not be located in a side stream flow and shall be located to treat all water returning to the pool or water features.

454.1.6.5.17

Water features such as waterfalls or fountains in pools may use up to 20 percent of the return water from the filter system, however all waters used in the feature shall not be counted toward attaining the designed turnover rate. Return piping system shall be designed and capable of handling the additional feature flow when the feature is turned off. Features that require more than 20 percent of the flow rate shall be supplied by an additional pump that drafts from a suitable collector tank. All water features that utilize water from the pool shall be designed to return the water to the pool. Spray features mounted in the pool deck shall be flush with the pool deck and shall be designed with the safety of the pool patron in mind.

454.1.6.5.18 Chemical quality

Only NSF-60 approved chemicals shall be provided.

454.1.7 Wading pools

454.1.7.1 General

Wading pools shall meet the requirements of Sections 454.1.1 through 454.1.6.5, unless otherwise indicated. Wading pools and associated piping shall not be physically connected to any other swimming pools and have no minimum width dimensions requirements.

454.1.7.2 Depths

Wading pools shall have a maximum depth of 2 feet (610 mm). The depth at the perimeter of the pool shall be uniform and shall not exceed 12 inches (305 mm). However, where jurisdictional building department-approved zero depth entry designs are used, this uniform depth requirement must be met only on the remainder of the pool outside the zero depth entry portion. The pool floor shall not be more than 12 inches (305 mm) below the deck unless steps and handrails are provided. Depth and "NO DIVING" markers are not required on wading pools.

454.1.7.3 Recirculation

Wading pools shall have a minimum of one turnover every hour. Lines from main drains shall discharge into a collector tank.

454.1.7.3.1

Skimmer equalizer lines when required shall be plumbed into the main drain installed in the pool floor with a grate covering.

454.1.7.3.2

The grate cover shall be sized so as not to allow the flow to exceed 1$^1/_2$ feet per second (457 mm/s) when the equalizer line is operating.

454.1.7.4 Inlets

Wading pools with 20 feet (6096 mm) or less of perimeter shall have a minimum of two equally spaced adjustable inlets.

Exported from UpCodes

454.1.7.5 Emergency drainage

All wading pools shall have drainage to waste without a cross connection through a quick opening valve to facilitate emptying the wading pool should accidental bowel or other discharge occur.

454.1.7.6 Vacuuming

Wading pools with 200 square feet (19 m^2) or more of pool water surface area shall have provisions for vacuuming through the skimmer, a portable vacuum system or an alternative approved method that does not involve a direct suction port in the pool.

454.1.7.7 Wading pool decks

When adjacent to swimming pools, wading pools shall be separated from the swimming pool by barrier or a fence of a minimum of 48 inches (1219 mm) in height with self-latching and self-closing gates. When adjacent to areas less than 1 foot (305 mm) deep of zero depth entry pools, the fence or effective barrier is required if the water edges are less than 40 feet (12 192 mm) apart. Wading pools shall have a minimum 10-feet (3048 mm) wide deck around at least 50 percent of their perimeter with the remainder of the perimeter deck being at least 4 feet (1219 mm) wide. There shall be at least 10 feet (3048 mm) between adjacent swimming pools and wading pools.

454.1.7.8 Lighting

Wading pools are exempt from underwater lighting requirements but shall have lighting installed for night use of 10 foot candles (100 lux) if indoors or 6 footcandles (60 lux) for outdoor night use. Such illumination shall be provided over the pool water surface and the pool deck surface

454.1.7.9

Automated oxidation reduction potential (ORP) and pH controllers with sensing probes shall be provided to assist in maintaining proper disinfection and pH levels.

454.1.8 Spa pools

454.1.8.1 General

Spa pools shall meet the requirements of Sections 454.1.1 through 454.1.6.5, unless specifically indicated otherwise.

454.1.8.2 Color, pattern, finish

The color, pattern or finish of the pool interior shall not obscure the existence or presence of objects or surfaces within the pool.

454.1.8.3 Water depths

Spa-type pools shall have a minimum water depth of $2^1/_2$ feet (762 mm) and a maximum water depth of 4 feet (1219 mm), except that swim spa pools may have a maximum water depth of 5 feet (1524 mm). Depth markers and “NO DIVING” markers are not required on spa-type pools with 200 square feet (19 m^2) or less of water surface area.

454.1.8.4 Steps and handrails

Steps or ladders shall be provided and shall be located to provide adequate entrance to and exit from the pool. The number of sets of steps or ladders required shall be on the basis of one for each 75 feet (22 860 mm), or major fraction thereof, of pool perimeter. Step sets for spa-type pools with more than 200-square-feet (19 m^2) of pool water surface area shall comply with Section 454.1.2.5. Step sets for spa-type pools with 200 square feet (19 m^2) or less of pool water surface area shall comply with the following: Step treads shall have a minimum width of 10 inches (254 mm) for a minimum continuous tread length of 12 inches (305 mm). Step riser heights shall not exceed 12 inches (305 mm). Intermediate treads and risers between the top and bottom treads and risers shall be uniform in width and height, respectively. Contrasting markings on the leading edges of the submerged benches and the intersections of the treads and risers are required to be installed in accordance with Section 454.1.2.5.

454.1.8.4.1

Handrails shall be provided for all sets of steps and shall be anchored in the bottom step and in the deckHandrails shall be located to provide maximum access to the steps and handrails shall extend 28 inches (711 mm) above the pool deck.

454.1.8.4.2

Where “figure 4” handrails are used, they shall be anchored in the deck and shall extend laterally to any point vertically above the bottom step. Handrails shall be located to provide maximum access to the steps and handrails shall extend 28 inches (711 mm) above the pool deck.

454.1.8.5 Decks

Decks shall have a minimum 4-footwide (1219 mm) unobstructed width around the entire pool perimeter except that pools of less than 120 square feet (11 m^2) of pool water surface area shall have a minimum 4-foot-wide (1219 mm) unobstructed continuous deck around a minimum of 50 percent of the pool perimeter. Decks less than 4 feet (1219 mm) wide shall have barriers to prevent their use. Decks shall not be more than 10 inches (254 mm) below the top of the pool. For pools of 120 square feet (11 m^2) or greater, 10 percent of the deck along the pool perimeter

Exported from UpCodes

may be obstructed.

454.1.8.6 Therapy or jet systems

454.1.8.6.1

The return lines of spa-type therapy or jet systems shall be independent of the recirculationfiltration and heating systems.

454.1.8.6.2

Therapy or jet pumps shall take suction from the collector tank. Collector tank sizing shall take this additional gallonage into consideration.

454.1.8.7 Filtration system

inlets

Spa-type pools with less than 20 feet (6096 mm) of perimeter shall have a minimum of two equally spaced adjustable inlets.

454.1.8.8 Filtration recirculation

Spa-type pools shall have a minimum of one turnover every 30 minutes. The piping, fittings, and hydraulic requirements shall be in accordance with Section 454.1.6.5. All recirculation lines to and from the pool shall be individually valved with proportional flow-type valves in order to control the recirculation flow.

454.1.8.9 Vacuuming

Spa-type pools of over 200 square feet (19 m^2) of pool water surface area shall have provisions for vacuuming.

454.1.8.10 Combination spas/pools

When spa pools are part of a conventional swimming pool, the spa pool area shall be offset from the main pool area with the same water depth as the main pool area. The spa pool shall meet all the spa pool requirements of this chapter, and the deck area at the spa shall be protected by connected 30-inch-high (762 mm) stanchions. The deck perimeter at the offset spa area shall not exceed 15 percent of the entire swimming pool perimeter. All benches shall have contrasting markings on the leading edges of the intersection of the bench seats. If tile is used, it shall be slip resistant.

454.1.8.11 Portable and wooden spa pools

Portable and wooden-type spa pools are prohibited.

454.1.8.12 Automated controllers

Automated oxidation reduction potential (ORP) and pH controllers with sensing probes shall be installed on spa pools to assist in maintaining proper disinfection and pH levels.

454.1.8.13

In addition to the requirements of Section 454.1.2.3.5 spa pool signs installed shall include the following:

1. Maximum water temperature: 104°F (40°C).
2. Children under twelve must have adult supervision.
3. Pregnant women, small children, people with health problems and people using alcohol, narcotics or other drugs that cause drowsiness should not use spa pools without first consulting a doctor.
4. Maximum use: 15 minutes.

454.1.8.14

A clock shall be visible from the spa pool to assist the patron in meeting Item 4 of Section 454.1.8.13.

454.1.8.15

If a spa is equipped with an emergency cutoff or kill switch, it shall include provisions for a minimum 80 decibel audible alarm near the spa to sound continuously until deactivated when such device is triggered. The following additional rule sign shall be installed to be visible by the spa which reads “ALARM INDICATES SPA PUMPS OFF. DO NOT USE SPA WHEN ALARM SOUNDS UNTIL ADVISED OTHERWISE.”

454.1.9 Water recreation attractions and specialized pools

454.1.9.1 General

Water recreation attraction projects shall be designed and constructed within the limits of sound engineering practice. In addition to the requirements of this section, compliance is required with Sections 454.1.1 through 454.1.6.5 of this chapter depending upon the pool design and

Exported from UpCodes

function. Additionally, all pools listed in this section shall have a 2-hour turnover rate unless otherwise noted.

454.1.9.2 Water slides

454.1.9.2.1 Water slide plunge pool

Plunge pools shall be constructed of concrete or other structurally rigid impervious materials with a nontoxic, smooth and slip-resistant finish. The plunge pool design shall meet the criteria of Sections 454.1.9.2.1.1 through 454.1.9.2.1.6.

454.1.9.2.1.1 Plunge pool water depth

The minimum plunge pool operating water depth at the slide flume terminus shall be 3 feet (914 mm). This depth shall be maintained for a minimum distance of 10 feet (3048 mm) in front of the slide terminus from which point the plunge pool floor may have a constant upward slope to allow a minimum water depth of 2 feet (51 mm) at the base of the steps. The floor slope shall not exceed 1 in 10. The plunge pool water depth shall be commensurate with safety and the ease of exit from the plunge pool.

454.1.9.2.1.2 Plunge pool dimension

The plunge pool dimension between any slide flume exit or terminus and the opposite side of the plunge pool shall be a minimum of 20 feet (6096 mm) excluding steps.

454.1.9.2.1.3 Slide flume terminus

454.1.9.2.1.3.1

The slide flume terminus shall be designed by the design engineer who can demonstrate to the jurisdictional building department's satisfaction that riders will be adequately slowed prior to discharge so as to prevent injury or harm to the rider upon impact with the plunge pool water. The slide terminus shall be flush with the pool wall and located at or below the pool water level.

454.1.9.2.1.3.2

The minimum distance between any plunge pool side wall and the outer edge of any slide terminus shall be 5 feet (1524 mm). The minimum distance between adjacent slide flumes shall be 6 feet (18 288 mm).

454.1.9.2.1.3.3

A minimum length of slide flume of 10 feet (3048 mm) shall be perpendicular to the plunge poolwall at the exit end of the flumes.

454.1.9.2.1.4 Plunge pool main drains

The plunge pool shall have a minimum of one main drain with separate piping and valve to the filtration systemcollector tank. The velocity through the openings of the main drain grate shall not exceed $1^1/_2$ feet per second (457 mm/s) at the design flow rate of the recirculation pump. The main drain piping shall be sized to handle 100 percent of the design flow rate of the filtration system with a maximum flow velocity of 3 feet (914 mm) per second.

454.1.9.2.1.5 Plunge pool floor slope

The plunge pool floor shall slope to the main drains and the slope shall not exceed 1 in 10.

454.1.9.2.1.6 Plunge pool decks

454.1.9.2.1.6.1 Width

The minimum width of plunge pool decks along the exit side shall be 10 feet (3048 mm).

454.1.9.2.1.6.2 Slopes

All plunge pool decks shall slope to the plunge pool or pump reservoir or to deck drains which discharge to waste, or other acceptable means. All slopes shall be between 2- and 4-percent grade.

454.1.9.2.2 Run out lanes

454.1.9.2.2.1

Run out lanes may be utilized in lieu of a plunge pool system, provided they are constructed to the slide manufacturers specifications and are approved by the design engineer of record.

454.1.9.2.2.2

Five-foot-wide (1524 mm) walkways shall be provided adjacent to run out lanes.

454.1.9.2.2.3

Minimum water level indicator markings shall be provided on both sides of the run out trough to ensure adequate water for the safe slowing

Exported from UpCodes

of pool patrons.

454.1.9.2.2.4

Water park personnel shall be provided at the top of the slides and at the run out.

454.1.9.2.3 Pump reservoirs

Pump reservoirs shall be made of concrete or other impervious material with a smooth slip-resistant finish. Pump reservoirs shall be for the slide pump intakes, but where properly sized may also be used as a collector tank for the filter system. Pump reservoir designs shall meet the criteria of Sections 454.1.9.2.3.1 through 454.1.9.2.3.5.

454.1.9.2.3.1 Pump reservoir volume

The minimum reservoir volume shall be equal to 3 minutes of the combined flow rate in gallons per minute of all filter and slide pumps.

454.1.9.2.3.2 Pump reservoir security

Pump reservoirs shall be accessible only to authorized individuals.

454.1.9.2.3.3 Pump reservoir maintenance accessibility

Access decks shall be provided for the reservoir such that all areas are accessible for vacuuming, skimming, and maintenance. The decks shall have a minimum width of 3 feet (914 mm) and shall have a minimum slope of 3:10 away from the reservoir.

454.1.9.2.3.4 Pump reservoir slide pump

intakes

The slide pump intakes shall be located in the pump reservoir and shall be designed to allow cleaning without danger of operator entrapment.

454.1.9.2.3.5 Pump reservoir main drains

The pump reservoir shall have a minimum of one main drain with separate piping and valve to the filtration systemcollector tank and the velocity through the openings of the main drain grates shall not exceed $1^1/_2$ feet per second (457 mm/s) at the design flow rate of the filtration system pump. The main drain piping shall be sized to handle 100 percent of design flow rate of the filtration system pump with a maximum flow velocity of 3 feet per second (914 mm/s).

454.1.9.2.3.6

The pump reservoir shall be fed by main drains within the plunge pool itself (either in the floor or sidewall). They shall have the maximum flow velocity of $1^1/_2$ feet per second (457 mm/s) through the main drain grating and 3 feet per second (3962 mm/s) through the reservoir piping.

454.1.9.2.4 Slide pump check valves

Slide pumps shall have check valves on all discharge lines.

454.1.9.2.5 Perimeter overflow gutters or skimmers

Plunge pools and pump reservoirs shall have perimeter overflow gutter system or skimmer which shall be an integral part of the filtration system.

454.1.9.2.5.1 Perimeter overflow gutter systems

Perimeter overflow gutter systems shall meet the requirements of Section 454.1.6.5.3.1 except that gutters are not required directly under slide flumes or along the weirs which separate plunge pools and pump reservoirs.

454.1.9.2.5.2 Surface skimmers

Surface skimmers may be used in lieu of perimeter overflow gutters and shall be appropriately spaced and located according to the structural design. Unless an overflow gutter system is used, surface skimmers shall be provided in the plunge pool and in the pump reservoir and the skimmer system shall be designed to carry 60 percent of the filtration system design flow rate with each skimmer carrying a minimum 30 gpm (2 L/s). All surface skimmers shall meet the requirements for NSF commercial approval as set forth in NSF/ANSI Standard 50, Circulation System Components and Related Materials for Swimming Pools, Spas/Hot Tubs, which is incorporated by reference in these rules, including an equalizer valve in the skimmer and an equalizer line to the pool wall on systems with direct connection to pump suction.

454.1.9.2.6 Water slide recirculation–filtration equipment

454.1.9.2.6.1 Recirculation rate

The recirculation-filtration system of water slides shall recirculate and filter a water volume equal to the total water volume of the facility in a period of 2 hours or less.

454.1.9.2.6.2 Filter areas

Exported from UpCodes

[illegible]

Minimum filter area requirements shall be twice the filter areas specified for the recirculation rates stipulated in Section 454.1.6.5.5.1. The filtration system shall be capable of returning the pool water turbidity to $^{5}/_{10}$ NTU within 8 hours or less after peak bather load.

454.1.9.2.6.3 Hair and lint strainer

Any filtration system pump which takes suction directly from the plunge pool and reservoir shall have a minimum 8-inch (208 mm) diameter hair and lint strainer on the suction side of the pump.

454.1.9.2.7 Disinfection

The disinfection equipment shall be capable of feeding 12 mg/L of halogen to the continuous recirculation flow of the filtration system.

454.1.9.2.8

Slide design and construction is the responsibility of a professional engineer licensed in Florida and the applicant.

454.1.9.2.9

A lockable gate shall be provided at the stair or ladder entrance to the slide.

454.1.9.2.10

Upon construction completion, a professional engineer licensed in Florida shall certify that the slide was constructed in accordance with the manufacturer’s specifications and is structurally sound.

454.1.9.3 Water activity pools

454.1.9.3.1

Water activity pools shall be designed and constructed within the limits of sound engineering practice.

454.1.9.3.2

Water activity pools shall be constructed of concrete or other structurally rigid impervious materials with a nontoxic, smooth and slip-resistant finish. These pools shall be of such shape and design as to be operated and maintained in a safe and sanitary manner.

454.1.9.3.3

The recirculation-filtration system of water activity pools shall achieve a minimum of one turnover every 2 hours for water activity pools over 2 feet (610 mm) deep, and 1 hour for these pools that are 2 feet (610 mm) deep or less.

454.1.9.3.4

Those portions of the activity pool where the water depth will not allow for the proper installation of underwater lighting shall be provided with 6 footcandles (60 lux) of lighting on the deck and water surface.

454.1.9.3.5

Fence requirements shall be in accordance with Section 454.1.7.7.

454.1.9.3.6

Play features with an overhead clearance of less than 4 feet (1219 mm) shall be blocked or barricaded to preclude children becoming entrapped.

454.1.9.3.7

In addition to the requirements of Section 454.1.2.3.5, all water activity pool signs installed shall have the following added in one inch letters:

“Do not swallow the pool water, it is recirculated.”

“Do not use pool if you are ill with diarrhea.”

454.1.9.4 Wave pools

454.1.9.4.1

Wave pools shall be designed and constructed within the limits of sound engineering practice.

454.1.9.4.2

Wave pools shall be constructed of concrete or other impervious materials with a smooth slip-resistant finish. These pools shall be of such shape and design as to be operated and maintained in a safe and sanitary manner.

Exported from UpCodes

454.1.9.4.3

The recirculation-filtration system of wave pools shall be capable of a minimum of one turnover every 3 hours.

454.1.9.4.4

Floors shall be sloped in accordance with the manufacturer's or designengineer's specifications; however, they shall not exceed the slope limits of Section 454.1.2.2.3.

454.1.9.5 River rides

454.1.9.5.1

River rides shall be constructed within the limits of sound engineering practice.

454.1.9.5.2

River rides shall be constructed on concrete or other impervious materials with a nontoxic, smooth and slip-resistant finish. These rides shall be of such shape and design as to be operated in a safe and sanitary manner.

454.1.9.5.3

The recirculation-filtration system of the river ride shall be capable of a minimum of one turnover every 3 hours.

454.1.9.5.4

The maximum water depth of the river ride shall not exceed 3 feet (914 mm) unless justified to the jurisdictional building department's satisfaction by the design engineer.

454.1.9.5.5

Decking shall be provided at the entrance and exit points as necessary to provide safe patron access but shall not be smaller than 10 feet (3048 mm) in width and length. Additional decking along the ride course is not required except that decking shall be required at lifeguard locations and emergency exit points.

454.1.9.5.6

Access and exit shall be provided at the start and end of the ride and additionalexit locations shall be located along the ride course as necessary to provide for the safety of the patrons.

Propulsion jets shall be installed in thewalls of the river ride. In the alternative, propulsion jets may be installed in the floor if they are covered by a grate that will inhibit entrapment or injury of the pool patrons' feet or limbs.

454.1.9.6 Zero depth entry

pools

454.1.9.6.1

Zero depth entry pools shall have a continuous floor slope from the water edge to the deep end.

454.1.9.6.2

The deck level perimeter overflow system with grate shall be provided at the water's edge across the entire zero depth portion of the pool.

454.1.9.6.3

The pool deck may slope toward the pool for no more than 7 feet (2133 mm), as measured from the overflow system grate outward. Beyond this area the deck shall slope away from the pool in accordance with Section 454.1.2.2.3.

454.1.9.6.4

"No Entry, Shallow Water" signs shall be provided along the poolwall edge where the water depth is less than 3 feet (914 mm) deep. No entry signs shall be slip-resistant, shall have 4-inchhigh (102 mm) letters, shall be located within 2 feet (610 mm) of the pool edge and shall be spaced no more than 15 feet (4572 mm) apart.

454.1.9.6.5

Additional inlets shall be provided in areas of less than 18 inches (457 mm) deep. The numbers and location shall be such as to double the flow rate into this area.

454.1.9.6.6

The recirculation-filtration system shall be of a minimum of one turnover every 2 hours in the area of the pool that is 3 feet (914 mm) deep or less. In the remainder of the pool where the depth is greater than 3 feet (914 mm), the system shall have a maximum 6 hour turnover rate. The design plans submitted by the applicant shall provide the volume of water in the pool area of 3 feet (914 mm) depth and less, the volume of

Exported from UpCodes

water in the pool area greater than 3 feet (914 mm) in depth and the total volume in the pool for determination of minimum circulation flow. The volume calculations shall provide verification that the correct volume of water is used to determine the minimum flow at the 2-hour and the 6-hour flow requirements.

454.1.9.6.7

Those portions of the zero depth entry pool, where the water depth will not allow for the proper installation of underwater lighting, shall be provided with 6 footcandles (60 lux) of lighting on the deck and the water.

454.1.9.6.8

Play structures in a zero depth entry area [in depth 0–3 feet (0 to 914 mm)] may be within 15 feet (4572 mm) of the poolwalls, but shall comply with sound engineering requirements for the safety of pool patrons.

454.1.9.7 Special purpose pools

454.1.9.7.1 General

Special purpose pool projects may deviate from the requirements of other sections of these rules provided the design and construction are within the limits of sound engineering practice. Only those deviations necessary to accommodate the special usage shall be allowed and all other aspects of the pool shall comply with the requirements of this section and with Section 454.1.2.

454.1.9.7.2

A special purpose pool may incorporate ledges which do not overhang into the pool.

454.1.9.8 Interactive water features (IWFs)

454.1.9.8.1

Waters discharged from all fountain or spray features shall not pond on the feature floor but shall flow by gravity through a main drain fitting to a below or collection system which discharges to a collector tank. The minimum size of the collector tank shall be equal to the volume of 3 minutes of the combined flow of all feature pumps and the filter pump. Smaller tanks may be utilized if hydraulically justified by the design engineer. Adequate access shall be provided to the sump or collector tank. Stairs or a ladder shall be provided as needed to ensure safe entry into the tank.

454.1.9.8.2

Reserved.

454.1.9.8.3

Chemical feeders shall be in accordance with Section 454.1.6.5; except that the disinfection feeder shall be capable of feeding 12 ppm of free chlorine to the pressure side of the recirculation system or the collector tank (based upon a hypothetical 30-minute turnover of the contained volume within the system). Automated oxidation reduction potential (ORP) and pH controllers with sensing probes shall be installed to assist in maintaining proper disinfection and pH levels.

454.1.9.8.4

If night operation is proposed, 6 footcandles (60 lux) of light shall be provided on the pool deck and the water feature area. Lighting that may be exposed to the feature pool water shall not exceed 15 volts, shall be installed in accordance with manufacturer's specifications and be approved for such use by UL or NSF.

454.1.9.8.5

All electrical work shall comply with Chapter 27 of the *Florida Building Code, Building*.

454.1.9.8.6 Hydraulics

454.1.9.8.6.1

All (100 percent) of the water from the collector tank must be first filtered, treated with disinfectant and pH adjustment chemicals, and then final treatment provided by an NSF Standard 50 certified UV disinfection unit with a minimum 40 mJ/cm^2 dose before any of this treated water is piped to the water features.

454.1.9.8.6.2

In the design above and the alternative below: excess water not required by the water features shall be returned to thecollector tank; the recirculation system shall be sized to treat the contained volume of water based upon a 30 minute turnover with a chlorine feeder/generator capable of producing a dosage of at least 12ppm; and the UV disinfection equipment shall be electrically interconnected such that whenever it fails to produce the required UV dosage, the water spray features pump(s) and flow will be immediately stopped.

454.1.9.8.6.3

Exported from UpCodes

In lieu of Section 454.1.9.8.6.1, the recirculation system must be designed to continuously return 100 percent of the water to thecollector tank after all (100 percent) of the water is first filtered, treated with disinfectant and pH adjustment chemicals, and the final treatment provided by a validated UV disinfectant unit described in Section 454.1.6.5.16.6 before any of this treated water is piped to the water features.

454.1.9.8.6.4

The flow rate through the feature nozzles of the water features shall be such as not to harm the patrons and shall not exceed 20 feet per second (6096 mm/s) unless justified by the design engineer and by the fountain system manufacturer.

454.1.9.8.6.5

An automatic water level controller shall be provided.

454.1.9.8.6.6

An overfill waste line with air gap shall be provided.

454.1.9.8.6.7

A means of vacuuming and completely draining the tank(s) shall be provided.

454.1.9.8.6.8

Reserved.

454.1.9.8.6.9

IWFs shall be fenced in the same fashion as wading pools as noted inSection 454.1.7.7. Where the IWF is at least 50 feet (15 240 mm) from all other pools and is not designed to have any standing water, fencing requirements should be carefully considered by the applicant to control usage, but are not required by rule.

454.1.9.8.6.10

A minimum 4-foot-wide (1219 mm) wet deck area shall be provided around all IWFs. The wet deck shall meet the requirements of Section 454.1.2.2.3; however, up to 50 percent of the perimeter may be obstructed.

454.1.9.8.6.11

IWFs shall be constructed of concrete or other impervious and structurally rigid material.

454.1.9.8.6.12

Floor slopes of an IWF shall be a maximum 1 foot (305 mm) vertical in 10 feet (3048 mm) horizontal and a minimum of 1 foot (305 mm) vertical in 50 feet (15 240 mm) horizontal.

454.1.9.8.6.13

In addition to the requirements of Section 454.1.2.3.5, all IWF pool rule signs installed shall have the following added in one inch letters:

“Do not swallow the fountain water, it is recirculated.”

“Do not use fountain if you are ill with diarrhea.”

454.1.9.8.7

Water theme parks shall meet all other aspects of these rules for the features provided.

454.1.9.8.7.1

Rules and regulations for water theme parks shall be posted in minimum 1-inch (305 mm) letters at each entrance to the park and shall contain the following:

1. No food, drink, glass or animals in or on the pool decks.
2. Park operating hours __A.M. to __P.M.
3. Shower before entering.
4. Do not swallow the pool water.

454.1.9.8.7.2

Showers shall be provided at or near the entrance (queue line) to a water recreation attraction.

Exported from UpCodes

454.1.9.8.7.3

Water theme parks are exempt from the fencing requirements ofSection 454.1.3.1.9, except that pools designed for small children shall be fenced when located within 50 feet (15 240 mm) of a pool with water depths of 3 feet (914 mm) or more.

454.1.9.8.7.4

Sanitary facilities within a water theme park shall be as near to the water recreation attractions as prudent to ensure patron use, but not over 200 feet (60 960 mm) walking distance from any exit of a water attraction.

454.1.10 Modifications

454.1.10.1 Repairs or alterations of pool structure and equipment

Replacement of the pool or spa shell is considered to be construction of a newfacility and shall be processed as such. Resurfacing the pool interior to original nontoxic, slip-resistant and smooth specifications is considered a repair or alteration. Equivalent replacement of equipment is not considered a repair or alteration. The following items shall be addressed during resurfacing projects:

454.1.10.1.1

The lip of the gutter must be leveled to within $^1/_4$ inch (6.4 mm) between the highest and lowest point and the downward slope from the lip to the drain must be maintained as originally designed or increased, but shall not exceed new construction standards.

454.1.10.1.2

Tile step markings must be installed meeting the requirements ofSection 454.1.2.5.3.

454.1.10.1.3

Where applicable, the slope break marking must be installed meeting the requirements ofSection 454.1.2.2.3.2 and safety line must be installed 2 feet (610 mm) before the marking.

454.1.10.1.4

Depth markers and NO DIVING markers must be installed in accordance withSection 454.1.2.3.

454.1.10.1.5

The pool ladder must have a 3 to 6 inch (76 to 152 mm) clearance from the poolwall. New cross-braced ladder(s) shall be installed in place of noncross-braced ladder(s) in conformance with Section 454.1.2.5.1 during a pool resurfacing.

454.1.10.1.6

Should resurfacing works affect the step riser heights, no riser shall exceed 10 inches (254 mm) for pools and 12 inches (305 mm) for spas, and the intermediate risers shall be made uniform.

454.1.10.1.7

Step treads that protrude from the pool wall shall be removed and replaced with a crossbraced ladder or reconstructed to meet the requirements of Section 454.1.2.5.1 or 454.1.2.5.2.

454.1.10.2

The painting of pools shall not be considered a *repair or alteration* provided the following conditions are met:

1. Only paints designated by the manufacturer as pool paints are used.
2. All step stripes, slope break markers and safety line, and depth and NO DIVING markings shall be provided to comply with the applicable provision(s) this section.

454.1.10.3

The installation of copper or copper/silver ionization units and ozone generators capable of producing less than a pool water ozone contact concentration of 0.1 milligrams per liter (mg/L) shall not be considered a pool repair or alteration provided compliance when the following is met:

1. The ionization or ozone generator unit complies with paragraph 64E-9.00*8(10)*(e), *Florida Administrative Code*.
2. The manufacturer provides one set of signed and sealed engineering drawings indicating the following:
    a. The unit does not interfere with the design flow rate.
    b. The unit and the typical installation meet the requirements of the *National Electrical Code*.
    c. A copper test kit and information regarding the maximum allowed copper and silver level and the minimum required chlorine level shall be available to the pool owner.

Exported from UpCodes

d. The unit shall meet the requirements of NSF/ANSI Standard 50.

3. At least 7 days before the time of installation, the installer will provide a photocopy of the above drawings and a letter of intent identifying the pool on which the unit is to be installed.

4. Upon completion of the installation, a professional engineer or electrician licensed in the state of Florida shall provide a letter to the county health department, indicating the unit was properly installed in accordance with the typical drawings, the *National Electrical Code* and local codes.

454.1.10.4 Electrical

454.1.10.4.1 Ground-fault circuit-interrupter protection for personnel

Outlets supplying repaired, replaced, altered, or relocated pool pump motors connected to single-phase, 120-volt through 240-volt branch circuits, whether by receptacle or by direct connection, and outlets supplying all other repaired, replaced, altered, or relocated electrical equipment and underwater luminaires operating at voltages greater than the low voltage contact limit, connected to single-phase, 120-volt through 240-volt branch circuits, rated 15- and 20-amperes, whether by receptacle or by direct connection, shall be provided with ground-fault circuit interrupter protection for personnel.

454.1.10.4.2 Equipotential bonding

Any of the parts specified in Sections 680.26(B)(1) through (B)(7) of the NFPA 70, *National Electrical Code* that are repaired, replaced, altered, or installed new at an existing swimming pool shall be connected to the existing bonding system using solid copper conductors, insulated, covered, or bare, not smaller than 8 AWG or with rigid metal conduit of brass or other identified corrosion-resistant metal. Connections to bonded parts shall be made in accordance with Section 250.8 of NFPA 70, *National Electrical Code*. An 8 AWG or larger solid copper bonding conductor provided to reduce voltage gradients in the pool area shall not be required to be extended or attached to remote panelboards, service equipment, or electrodes. All metallic float-in light rings shall be connected to the equipotential bonding grid. Float-in light rings with no provision for bonding, and other devices which do not provide an electrical connection between a metallic underwater luminaire and the forming shell of a wet niche fixture, including screws or bolts not supplied by the luminaire's manufacturer and listed for use with the specific luminaire, shall not be allowed for use with any underwater luminaire that is required to be grounded. Where none of the bonded parts is in direct connection with the pool water, the pool water shall be in direct contact with an approved corrosion-resistant conductive surface that exposes not less than 9 square inches (5800 mm^2) of surface area to the pool water at all times. The conductive surface shall be located where it is not exposed to physical damage or dislodgement during usual pool activities, and it shall be bonded in accordance with Section 680.26(B) of the NFPA 70, *National Electrical Code*. A bonded concrete pool shell shall be considered to be a conductive surface. The interior metallic surface or surfaces of any forming shell (wet niche) shall not be covered with any material, including plaster, except potting compound covering internal bonding connections in conformance with 680.23(B)(2)(b) of NFPA 70, *National Electrical Code*, shall be allowed.

454.2 Private swimming pools

454.2.1 Definitions–general

454.2.1.1 Tense, gender and number

For the purpose of this code, certain abbreviations, terms, phrases, words, and their derivatives shall be construed as set forth in this section. Words used in the present tense include the future. Words in the masculine gender include the feminine and neuter. Words in the feminine and neuter gender include the masculine. The singular number includes the plural and the plural number includes the singular.

454.2.1.2 Words not defined

Words not defined herein shall have the meanings stated in the *Florida Building Code, Building Florida Building Code, Mechanical*; *Florida Building Code, Plumbing*; *Florida Building Code, Fuel Gas*; or *Florida Fire Prevention Code*. Words not defined in the *Florida Building Code* shall have the meanings stated in the Webster's *Ninth New Collegiate Dictionary*, as revised.

454.2.2 Definitions

ABOVE-GROUND/ON-GROUND POOL. See "Swimming pool."

ADMINISTRATIVE AUTHORITY. The individual official, board, department or agency established and authorized by a state, county, city or other political subdivision created by law to administer and enforce the provisions of the swimming pool code as adopted or amended.

APPROVED. Accepted or acceptable under an applicable specification stated or cited in this code, or accepted as suitable for the proposed use under procedures and power of the administrative authority.

APPROVED SAFETY COVER. A manually or power-applied safety pool cover that meets all of the performance standards of ASTM International in compliance with ASTM F1346.

Exported from UpCodes

APPROVED TESTING AGENCY. An organization primarily established for the purpose of testing to approved standards and approved by the administrative authority.

BACKWASH PIPING. See “Filter waste discharge piping.”

BARRIER. A fence, dwelling wall or nondwelling wall or any combination thereof which completely surrounds theswimming pool and obstructs access to the swimming pool, especially access from the residence or from theyard outside the barrier.

BODY FEED. Filter aid fed into a diatomite-type filter throughout the filtering cycle.

CARTRIDGE FILTER. A filter using cartridge-type filter elements.

CHEMICAL PIPING. Piping which conveys concentrated chemical solutions from a feeding apparatus to the circulation piping.

CIRCULATION PIPING SYSTEM. Piping between the pool structure and the mechanical equipment. Usually includes suction piping, face piping and return piping.

COMBINATION VALVE. A multipart valve intended to perform more than one function.

DESIGN HEAD. Total head requirement of the circulation system at the design rate of flow.

DIATOIMTE (DIATOAMCEOUS EARTH). A type of filter aid.

DIATOIMTE-TYPE FILTER. A filter designed to be used with filter aid.

DIRECT ACCESS FROM THE HOME. Any opening which discharges into the “perimeter” of the pool or any opening in an exteriordwelling wall, or interior wall (for indoor pools) which faces the pool.

EXIT ALARM. A device that makes audible, continuous alarm sounds when any door or window which permits access from the residence to any pool that is without an intervening enclosure is opened or left ajar.

FACE PIPING. Piping, with all valves and fittings, which is used to connect the filter system together as a unit.

FILTER. Any apparatus by which water is clarified.

FILTER AID. A nonpermanent type of filter medium or aid such as diatomite, alum, etc.

FILTER CARTRIDGE. A disposable or renewable filter element which generally employs no filter aid.

FILTER ELEMENT. That part of a filter which retains the filter medium.

FILTER MEDIUM. Fine material which entraps the suspended particles and removes them from the water.

FILTER RATE. Average rate of flow per square foot of filter area.

FILTER ROCK. Specially graded rock and gravel used to support filter sand.

FILTER SAND. A specially graded type of permanent filter medium.

FILTER SEPTUM. That part of the filter element in a diatomite-type filter upon which a cake of diatomite or other nonpermanent filter aid may be deposited.

FILTER WASTE DISCHARGE PIPING. Piping that conducts waste water from a filter to a drainage system. Connection to drainage system is made through an air gap or other approved methods.

FRESH WATER. Those waters having a specific conductivity less than a solution containing 6,000 ppm of sodium chloride.

HIGH RATE SAND FILTER. A sand filter designed for flows in excess of 5 gpm (0.3 L/s) per square foot.

HOT TUB. See “Swimming pool.”

Exported from UpCodes

INGROUND POOL. See "Swimming pool."

INLET FITTING. Fitting or fixture through which circulated water enters the pool.

MAIN SUCTION OUTLET. Outlet at the deep portion of the pool through which the main flow of water leaves the pool when being drained or circulated.

MESH SAFETY BARRIER. A combination of materials, including fabric, posts, and other hardware to form a barrier around a swimming pool.

MEDICALLY FRAIL ELDERLY PERSON. Any person who is at least 65 years of age and has a medical problem that affects balance, vision, or judgment, including but not limited to a heart condition, diabetes, or Alzheimer's disease or any related disorder.

POOL. See "Swimming pool."

POOL DEPTHS. The distance between the floor of pool and the maximum operating water level.

POOL PERIMETER. A pool perimeter is defined by the limits of the pool deck, its surrounding area including yard area on same property, and any dwelling or nondwelling wall or any combination thereof which completely surrounds the pool.

POOL PLUMBING. All chemical, circulation, filter waste discharge piping, deck drainage and water filling systems.

PORTABLE POOL. A prefabricated pool which may be erected at the point of intended use and which may be subsequently disassembled and reerected at a new location. Generally installed on the surface of the ground and without excavation.

PRECOAT. In a diatomite-type filter, the initial coating or filter aid placed on the filter septum at the start of the filter cycle.

RAPID SAND FILTER. A filter designed to be used with sand as the filter medium and for flows not to exceed 5 gpm (0.3 L/s) per square foot.

RECEPTOR. An approved plumbing fixture or device of such material, shape and capacity as to adequately receive the discharge from indirect waste piping, so constructed and located as to be readily cleaned.

RESIDENTIAL. Situated on the premises of a detached one- or two-family dwelling or a one-family townhouse not more than three stories high.

RETURN PIPING. That portion of the circulation piping which extends from the outlet side of the filters to the pool.

SALINE WATER. Those waters having a specific conductivity in excess of a solution containing 6,000 ppm of sodium chloride.

SEPARATION TANK. A device used to clarify filter rinse or waste water; sometimes called a "reclamation tank."

SKIM FILTER. A surface skimmer combined with a vacuum diatomite filter.

SPA, NONPORTABLE. See "Swimming pool."

SPA, PORTABLE. Nonpermanent structure intended for recreational bathing, in which all controls and water heating and water circulating equipment are an integral part of the product and which is cord-connected and not permanently electrically wired.

SUCTION PIPING. That portion of the circulation piping located between the pool structure and the inlet side of the pump and usually includes mair outlet piping, skimmer piping, vacuum piping and surge tank piping.

SURFACE SKIMMER. A device generally located in the pool wall which skims the pool surface by drawing pool water over a self-adjusting weir.

SWIMMING POOL, PRIVATE. Any structure, located in a residential area, that is intended for swimming or recreational bathing and contains water over 24 inches (610 mm) deep including but not limited to inground, aboveground, and onground swimming pools, hot tubs, and nonportable spas.

SWIMMING POOL, INDOOR. A swimming pool which is totally contained within a structure and surrounded on all four sides by walls of said structure.

SWIMMING POOL, OUTDOOR. Any swimming pool which is not an indoor pool.

SWIMMING POOL, PUBLIC. A watertight structure of concrete, masonry, fiberglass, stainless steel or plastic which is located either indoors or

Exported from UpCodes

outdoors, used for bathing or swimming by humans, and filled with a filtered and disinfected water supply, together with buildings, appurtenances and equipment used in connection therewith. A public swimming pool or public pool shall mean a conventional pool, spa-type pool, wading pool, special purpose pool or water recreation attraction, to which admission may be gained with or without payment of a fee and includes, pools operated by or serving camps, churches, cities, counties, day care centers, group home facilities for eight or more clients, health spas, institutions, parks, state agencies, schools, subdivisions; or the cooperative living-type projects of five or more living units, such as apartments, boarding houses, hotels, mobile home parks, motels, recreational vehicle parks and townhouses.

SWIMMING POOL, RESIDENTIAL. See "Swimming pool, private."

TURNOVER TIME. The time in hours required for the circulation system to filter and recirculate a volume of water equal to the pool volume.

VACUUM FITTING. A fitting in the pool which is used as a convenient outlet for connecting the underwater suction cleaning equipment.

VACUUM PIPING. The piping from the suction side of a pump connected to a vacuum fitting located at the pool and below the water level.

WASTE PIPING. See "Filter waste discharge piping."

WIDTH AND/OR LENGTH. Actual water dimension taken from wall to wall at the maximum operating water level.

YOUNG CHILD. Any person under the age of 6 years.

454.2.3 Mechanical requirements

Unless otherwise specified in this code, all piping, equipment and materials used in the process piping system ofswimming pools that are built in place shall conform to the *Florida Building Code, Plumbing*.

454.2.4 Approvals

454.2.4.1 Compliance

All materials, piping, valves, equipment or appliances entering into the construction of swimming pools or portions thereof shall be of a type complying with this code or of a type recommended and approved by a nationally recognized testing agency or conforming to other recognized standards acceptable to the administrative authority.

454.2.4.2 Items not covered

For any items not specifically covered in these requirements, the administrative authority is hereby authorized to require that all equipment, materials, methods of construction and design features shall be proven to function adequately, effectively and without excessive maintenance and operational difficulties.

454.2.4.2.1 Flood hazard areas

Private swimming pools installed in flood hazard areas established in Section 1612.3 shall comply with Section 1612.

454.2.4.3 Applicant responsibility

It shall be the responsibility of the applicant to provide such data, tests or other adequate proof that the device, material or product will satisfactorily perform the function for which it is intended, before such item shall be approved or accepted for tests.

454.2.5 Alternate materials and methods of construction

454.2.5.1 Approval and authorization

The provisions of this code are not intended to prevent the use of any alternate material, method of construction, appliance or equipment, provided any such alternate has been first approved and its use authorized by the administrative authority.

454.2.5.2 Required

tests

When there is insufficient evidence to substantiate claims for alternates, the administrative authority may require tests, as proof of compliance, to be made by an approved agency at the expense of the applicant.

454.2.6 Private swimming pools

454.2.6.1 Conformance standard

Design, construction and workmanship shall be in conformity with the requirements of ANSI/APSP/ICC 3, ANSI/APSP/ICC 4, ANSI/APSP/ICC 5, ANSI/APSP/ICC 6, and ANSI/APSP/ICC 7.

454.2.6.2 Required equipment

454.2.6.2 Required equipment

Every swimming pool shall be equipped complete with approved mechanical equipment consisting of filter, pump, piping valves and component parts.

Exception: Pools with a supply of fresh water equivalent to the volume of the pool in the specified turnover time will be allowed.

454.2.6.3 Water velocity

Pool piping shall be designed so the water velocity will not exceed 10 feet per second (mm/s) for pressure piping and 8 feet per second (mm/s) for suction piping, except that the water velocity shall not exceed 8 feet per second (3048 mm/s) in copper tubing. Main suction outlet velocity must comply with ANSI/APSP/ICC 7.

Exception: Jet inlet fittings shall not be deemed subject to this requirement.

454.2.6.4 Piping to heater

Water flow through the heater, any bypass plumbing installed, any backsiphoning protection, and the use of heat sinks shall be done in accordance with the manufacturer's recommendations.

454.2.6.5 Piping installation

All piping materials shall be installed in strict accordance with the manufacturer's installation standards.

Exception: Primer and glue on exposed aboveground piping not required to be colored.

454.2.6.6

Entrapment protection for suction outlets shall be installed in accordance with requirements of ANSI/APSP 7/ICC 7.

454.2.7 Pumps

454.2.7.1 Strainer

Pool circulating pumps shall be equipped on the inlet side with an approved-type hair and lint strainer when used with a pressure filter.

454.2.7.2 Installation

Pumps shall be installed in accordance with manufacturer recommendations.

454.2.7.3 Capacity

Pumps shall have design capacity at the following heads.

1. Pressure diatomaceous earth–At least 60 feet (18 288 mm).
2. Vacuum D.E.–20-inch (508 mm) vacuum on the suction side and 40 feet (1219 mm) total head.
3. Rapid sand–At least 45 feet (13 716 mm).
4. High rate sand–At least 60 feet (18 288 mm).

454.2.7.4 Materials

Pump impellers, shafts, wear rings and other working parts shall be of corrosionresistant materials.

454.2.8 Valves

454.2.8.1 General

Valves shall be made of materials that are approved in the *Florida Building Code, Plumbing*. Valves located under concrete slabs shall be set in a pit having a least dimension of five pipe diameters with a minimum of at least 10 inches (254 mm) and fitted with a suitable cover. All valves shall be located where they will be readily accessible for maintenance and removal.

454.2.8.2 Full-way (gate)

valves

Full-way valves shall be installed to insure proper functioning of the filtration and piping system. When the pump is located below the overflow rim of the pool, a valve shall be installed on the discharge outlet and the suction line.

454.2.8.3 Check valves

Where check valves are installed they shall be of the swing, spring or vertical check patterns.

454.2.8.4 Combination valves

Combination valves shall be installed per the manufacturer's installation instructions.

454.2.9 Water

supply

Unless an approved type of filling system is installed, any water supply which in the judgment of the administrative authority may be used to fill the pool, shall be equipped with backflow protection. No over the rim fill spout shall be accepted unless located under a diving board, or properly guarded.

454.2.10 Waste water disposal

454.2.10.1 Connection limitations

Direct or indirect connections shall not be made between any storm drain, sewer, drainage system, seepage pit underground leaching pit, or subsoil drainage line, and any line connected to a swimming pool unless approved by the administrative authority.

454.2.10.2 Disposal through public

sewer

When the waste water from a swimming pool is to be disposed of through a public sewer, a 3-inch (76 mm) P-trap shall be installed on the lower terminus of the building drain and the tall piece from the trap shall extend a minimum of 3 inches (76 mm) above finished grade and below finished floor grade. This trap need not be vented. The connection between the filter waste discharge piping and the P-trap shall be made by means of an indirect connection.

454.2.10.3 Deviations

Plans and specifications for any deviation from the above manner of installation shall first be approved by the administrative authority before any portion of any such system is installed. When waste water disposal is to seepage pit installation, it shall be installed in accordance with the approval granted by the administrative authority.

454.2.11 Separation tank

A separation tank of an approved type may be used in lieu of the aforementioned means of waste water disposal when connected as a reclamation system.

454.2.12 Tests

454.2.12.1 Pressure

test

All pool piping shall be tested and proved tight to the satisfaction of the administrative authority, under a static water or air pressure test of not less than 35 psi (241 kPa) for 15 minutes.

Exception: Circulating pumps need not be tested as required in this section.

454.2.12.2 Drain and waste piping

All drain and waste piping shall be tested by filling with water to the point of overflow and alljoints shall be tight.

454.2.13 Drain piping

454.2.13.1 Slope to discharge

Drain piping serving gravity overflow gutter drains and deck drains shall be installed to provide continuous grade to point of discharge.

454.2.13.2 Joints and connections

Joints and connections shall be made as required by the *Florida Building Code, Plumbing*.

454.2.14 Water heating equipment

454.2.14.1 Labels

Swimming pool water heating equipment shall conform to the design, construction and installation requirements in accordance with accepted engineering practices and shall bear the label of a recognized testing agency, and shall include a consideration of combustion air, venting and gas supply requirements for water heaters.

454.2.14.2 Water retention

If a heater is not equipped or designed for an approved permanent bypass or antisiphon device, an approved permanent bypass or antisiphon

Exported from UpCodes

device shall be installed to provide a positive means of retaining water in the heater when the pump is not in operation.

454.2.14.3 Pit

drainage

When the heater is installed in a pit, the pit shall be provided with approved drainage facilities.

454.2.14.4 Connections

All water heating equipment shall be installed with flanges or union connection adjacent to the heater.

454.2.14.5 Relief valve

When water heating equipment which is installed in a closed system has a valve between the appliance and the pool, a pressure relief valve shall be installed on the discharge side of the water heating equipment. For units up to and including 200,000 Btu/hour input, the relief valve shall be rated by the American Gas Association.

454.2.15 Gas piping

Gas piping shall comply with the *Florida Building Code, Fuel Gas*.

454.2.16 Electrical

Electrical equipment wiring and installation, including the bonding and grounding of pool components, shall comply with Chapter 27 of the *Florida Building Code, Building*. Outlets supplying pool pump motors connected to single-phase 120-volt through 240-volt branch circuits, whether by receptacle or by direct connection, and outlets supplying other electrical equipment and underwater luminaires operating at voltages greater than the low voltage contact limit, connected to single-phase, 120 volt through 240 volt branch circuits, rated 15 or 20 amperes, whether by receptacle or by direct connection, shall be provided with ground-fault circuit interrupter protection for personnel.

454.2.17 Residential swimming barrier requirement

Residential swimming pools shall comply with Sections 454.2.17.1 through 454.2.17.3.

Exception: A swimming pool with an approved safety pool cover complying with ASTM F1346.

454.2.17.1 Outdoor swimming pools

Outdoor swimming pools shall be provided with a barrier complying with Sections 454.2.17.1.1 through 454.2.17.1.14.

454.2.17.1.1

The top of the barrier shall be at least 48 inches (1219 mm) above grade measured on the side of the barrier which faces away from the swimming pool. The maximum vertical clearance between grade and the bottom of the barrier shall be 2 inches (51 mm) measured on the side of the barrier which faces away from the swimming pool. Where the top of the pool structure is above grade the barrier may be at ground level or mounted on top of the pool structure. Where the barrier is mounted on top of the pool structure, the maximum vertical clearance between the top of the pool structure and the bottom of the barrier shall be 4 inches (102 mm).

454.2.17.1.2

The barrier may not have any gaps, openings, indentations, protrusions, or structural components that could allow a young child to crawl under, squeeze through, or climb over the barrier as herein described below. One end of a removable child barrier shall not be removable without the aid of tools. Openings in any barrier shall not allow passage of a 4-inch diameter (102 mm) sphere.

454.2.17.1.3

Solid barriers which do not have openings shall not contain indentations or protrusions except for normal construction tolerances and tooled masonry joints.

454.2.17.1.4

Where the barrier is composed of horizontal and vertical members and the distance between the tops of the horizontal members is less than 45 inches (1143 mm), the horizontal members shall be located on the swimming pool side of the fence. Spacing between vertical members shall not exceed $1^3/_4$ inches (44 mm) in width. Where there are decorative cutouts within vertical members, spacing within the cutouts shall not exceed $1^3/_4$ inches (44 mm) in width.

454.2.17.1.5

Where the barrier is composed of horizontal and vertical members and the distance between the tops of the horizontal members is 45 inches (1143 mm) or more, spacing between vertical members shall not exceed 4 inches (102 mm). Where there are decorative cutouts within vertical members, spacing within the cutouts shall not exceed $1^3/_4$ inches (44 mm) in width.

454.2.17.1.6

Maximum mesh size for chain link fences shall be a $2^1/_4$ inch (57 mm) square unless the fence is provided with slats fastened at the top or

bottom which reduce the openings to no more than $1^3/_4$ inches (44 mm).

454.2.17.1.7

Where the barrier is composed of diagonal members, the maximum opening formed by the diagonal members shall be no more than $1^3/_4$ inches (44 mm).

454.2.17.1.8

Access gates, when provided, shall be self-closing and shall comply with the requirements of Sections 454.2.17.1.1 through 454.2.17.1.7 and shall be equipped with a self-latching locking device located on the pool side of the gate. Where the device release is located no less than 54 inches (1372 mm) from the bottom of the gate, the device release mechanism may be located on either side of the gate and so placed that it cannot be reached by a young child over the top or through any opening or gap from the outside. Gates that provide access to the swimming pool must open outward away from the pool. The gates and barrier shall have no opening greater than $^1/_2$ inch (12.7 mm) within 18 inches (457 mm) of the release mechanism.

454.2.17.1.9

Where a wall of a dwelling serves as part of the barrier, one of the following shall apply:

1. All doors and windows providing direct access from the home to the pool shall be equipped with anexit alarm complying with UL 2017 that has a minimum sound pressure rating of 85 dBA at 10 feet (3048 mm). Any deactivation switch shall be located at least 54 inches (1372 mm) above the threshold of the access. Separate alarms are not required for each door or window if sensors wired to a central alarm sound when contact is broken at any opening.
   Exceptions:
   a. Screened or protected windows having a bottom sill height of 48 inches (1219 mm) or more measured from the interior finished floor at the pool access level.
   b. Windows facing the pool on the floor above the firststory.
   c. Screened or protected pass-through kitchen windows 42 inches (1067 mm) or higher with a counter beneath.
2. All doors providing direct access from the home to the pool must be equipped with a selfclosing, self-latching device with positive mechanical latching/locking installed a minimum of 54 inches (1372 mm) above the threshold, which is approved by the authority having jurisdiction.
3. A swimming pool alarm that, when placed in a pool, sounds an alarm upon detection of an accidental or unauthorized entrance into the water. Such pool alarm must meet and be independently certified to ASTM F2208, titled "Standard Safety Specification for Residential Pool Alarms," which includes surface motion, pressure, sonar, laser, and infrared alarms. For purposes of this paragraph, the term "swimming pool alarm" does not include any swimming protection alarm device designed for individual use, such as an alarm attached to a child that sounds when the child exceeds a certain distance or becomes submerged in water.

454.2.17.1.10

Where an above-ground pool structure is used as a barrier or where the barrier is mounted on top of the pool structure, and the means of access is a ladder or steps, the ladder or steps either shall be capable of being secured, locked or removed to prevent access, or the ladder or steps shall be surrounded by a barrier which meets the requirements of Sections 454.2.17.1.1 through 454.2.17.1.9 and Sections 454.2.17.1.12 through 454.2.17.1.14. When the ladder or steps are secured, locked or removed, any opening created shall not allow the passage of a 4-inch-diameter (102 mm) sphere.

454.2.17.1.11

Standard screen enclosures which meet the requirements of Section 454.2.17 may be utilized as part of or all of the "barrier" and shall be considered a "nondwelling" wall. Removable child barriers shall have one end of the barrier nonremovable without the aid of tools.

454.2.17.1.12

The barrier must be placed around the perimeter of the pool and must be separate from any fence,wall, or other enclosure surrounding the yard unless the fence, wall, or other enclosure or portion thereof is situated on the perimeter of the pool, is being used as part of the barrier, and meets the barrier requirements of this section.

454.2.17.1.13

Removable child barriers must be placed sufficiently away from the water's edge to prevent a young child or medically frail elderly person who may manage to penetrate the barrier from immediately falling into the water. Sufficiently away from the water's edge shall mean no less than 20 inches (508 mm) from the barrier to the water's edge. Dwelling or nondwelling walls including screen enclosures, when used as part or all of the barrier and meeting the other barrier requirements, may be as close to the water's edge as permitted by this code.

454.2.17.1.14

Exported from UpCodes

A wall of a dwelling may serve as part of the barrier if it does not contain any door or window that opens to providedirect access from the home to the swimming pool.

454.2.17.1.15

A mesh safety barrier meeting the requirements of Section 454.2.17 and the following minimum requirements shall be considered a barrier as defined in this section:

1. Individual component vertical support posts shall be capable of resisting a minimum of 52 pounds (24 kg) of horizontal force prior to breakage when measured at a 36 inch (914 mm) height above grade. Vertical posts of the child safety barrier shall extend a minimum of 3 inches (76 mm) below deck level and shall be spaced no greater than 36 inches (914 mm) apart.

2. The mesh utilized in the barrier shall have a minimum tensile strength according to ASTM D5034 of 100 pounds per foot, and a minimum ball burst strength according to ASTM D3787 of 150 pounds per foot. The mesh shall not be capable of deformation such that a $^{1}/_{4}$-inch (6.4 mm) round object could not pass through the mesh. The mesh shall receive a descriptive performance rating of no less than “trace discoloration” or “slight discoloration” when tested according to ASTM G53, Weatherability, 1,200 hours.

3. When using a molding strip to attach the mesh to the vertical posts, this strip shall contain, at a minimum, #8 by $^{1}/_{2}$ inch (12.7 mm) screws with a minimum of two screws at the top and two at the bottom with the remaining screws spaced a maximum of 6 inches (152 mm) apart on center.

4. Patio deck sleeves (vertical post receptacles) placed inside the patio surface shall be of a nonconductive material.

5. A latching device shall attach each barrier section at a height no lower than 45 inches (1143 mm) above grade. Common latching devices that include, but are not limited to, devices that provide the security equal to or greater than that of a hook-and-eye-type latch incorporating a spring actuated retaining lever (commonly referred to as a safety gate hook).

6. The bottom of the mesh safety barrier shall not be more than 1 inch (25 mm) above the deck or installed surface (grade).

454.2.17.1.16 Adjacent waterways

Permanent natural or permanent man-made features such as bulkheads, canals, lakes, navigable waterways, etc., adjacent to a public or private swimming pool or spa may be permitted as a barrier when approved by the authority having jurisdiction. When evaluating such barrier features, the authority may perform on-site inspections and review evidence such as surveys, aerial photographs, water management agency standards and specifications, and any other similar documentation to verify, at a minimum, the following:

1. The barrier feature is not subject to natural changes, deviations, or alterations and is capable of providing an equivalent level of protection as that provided by the code.

2. The barrier feature clearly impedes, prohibits or restricts access to the swimming pool or spa.

454.2.17.2 Indoor swimming pools

All walls surrounding indoor swimming pools shall comply with Section 454.2.17.1.9.

454.2.17.3 Prohibited locations

A barrier may not be located in a way that allows any permanent structure, equipment, or window that opens to provide access from the home to the swimming pool.

454.2.18 Ladders and steps

All pools whether public or private shall be provided with a ladder or steps in the shallow end where water depth exceeds 24 inches (610 mm). In private pools where water depth exceeds 5 feet (1524 mm) there shall be ladders, stairs or underwater benches/ swim-outs in the deep end. Where manufactured diving equipment is to be used, benches or swim-outs shall be recessed or located in a corner.

Exception: In private pools having more than one shallow end, only one set of steps are required. A bench, swimout or ladder may be used at all additional shallow ends in lieu of an additional set of steps.

454.2.19 Final inspection

Final electrical, and barrier code, inspection shall be completed prior to filling the pool with water.

Exception: Vinyl liner and fiberglass pools are required to be filled with water upon installation.

454.2.20 Filters

Exported from UpCodes

Components shall have sufficient capacity to provide a complete turnover of pool water in 12 hours or less.

454.2.20.1 Sand filters

454.2.20.1.1 Approved types

Rapid sand filters [flow up to 5 gpm per square foot (0.3L/s)] shall be constructed in accordance with approved standards. Where high rate sand filters [flow in excess of 5 gpm per square foot (0.3 L/s)] are used, they shall be of an approved type. The circulation system and backwash piping shall be adequate for proper backwashing of said filter and shall provide backwash flow rates of at least 12 gpm per square foot (0.8 L/s) or rapid sand filters or 15 gpm per square foot (0.9 L/s) for high rate sand filters.

454.2.20.1.2 Instructions

Every filter system shall be provided with written operating instructions.

454.2.20.1.3 Filter system equipment

On pressure-type filters, a means shall be provided to permit the release of internal pressure. A filter incorporating an automatic internal air release as its principal means of air release shall have lids which provide a slow and safe release of pressure as part of its design. A separation tank used in conjunction with a filter tank shall have as part of its design a manual means of air release or a lid which provides a slow and safe release of pressure as it is opened.

454.2.20.2 Diatomite-type filters

454.2.20.2.1 Design

Diatomite-type filters shall be designed for operation under either pressure or vacuum. The design capacity for both pressure and vacuum filters shall not exceed 2 gpm per square foot (0.13 L/s) of effective filter area.

454.2.20.2.2 Filter

aid

Provision shall be made to introduce filter aid into the filter in such a way as to evenly precoat the filter septum.

454.2.21 Pool fittings

454.2.21.1 Approved type

Pool fittings shall be of an approved type and design as to be appropriate for the specific application.

454.2.21.2 Skimmers

Approved surface skimmers are required and shall be installed in strict accordance with the manufacturer's installation instructions. Skimmers shall be installed on the basis of one per 800 square feet (74 m^2) of surface area or fraction thereof, and shall be designed for a flow rate of at least 25 gpm (94 L/m) per skimmer.

454.2.21.3 Main

outlet

An approved main outlet, when provided, shall be located on a wall or floor at or near the deepest point in the pool for emptying or circulation, or both, of the water in the pool.

454.2.21.4 Hydrostatic relief device

In areas of anticipated water table an approved hydrostatic relief device shall be installed.

Exception: Plastic liner pools (where there is no structural bottom to the pool).

454.2.21.5 Inlet fittings

Approved manufactured inlet fittings for the return of recirculated pool water shall be provided on the basis of at least one per 300 square feet (28 m^2) of surface area. Such inlet fittings shall be designed and constructed to insure an adequate seal to the pool structure and shall incorporate a convenient means of sealing for pressure testing of the pool circulation piping. Where more than one inlet is required, the shortest distance between any two required inlets shall be at least 10 feet (3048 mm).

454.2.22 Equipment foundations and

enclosures

All pool motors and equipment shall be installed in compliance with the manufacturer's recommendations. All heating and electrical equipment, unless approved for outdoor installation, shall be adequately protected against the weather or installed within a building.

[illegible]

Exported from UpCodes

454.2.23 Accessibility and clearances

Equipment shall be so installed as to provide ready accessibility for cleaning, operating, maintenance and servicing.

Exported from UpCodes

The Florida Senate

2019 Florida Statutes

Title XXXIII<br>REGULATION OF TRADE, COMMERCE, INVESTMENTS, AND SOLICITATIONS	**Chapter 514**<br>**PUBLIC SWIMMING AND BATHING FACILITIES**

CHAPTER 514
PUBLIC SWIMMING AND BATHING FACILITIES

514.011 Definitions.
514.0115 Exemptions from supervision or regulation; variances.
514.021 Department authorization.
514.023 Sampling of beach waters; and public bathing places; health advisories.
514.0231 Advisory committee to oversee sampling of beach waters.
514.025 Assignment of authority to county health departments.
514.028 Advisory review board.
514.03 Approval necessary to construct, develop, or modify public swimming pools or public bathing places.
514.031 Permit necessary to operate public swimming pool.
514.0315 Required safety features for public swimming pools and spas.
514.033 Creation of fee schedules authorized.
514.04 Right of entry.
514.05 Denial, suspension, or revocation of permit; administrative fines.
514.06 Injunction to restrain violations.
514.071 Certification of swimming instructors and lifeguards required.
514.072 Certification of swimming instructors for people who have developmental disabilities.
514.075 Public pool service technician; certification.

514.011 Definitions.— As used in this chapter:

(1) "Department" means the Department of Health.

(2) "Public swimming pool" or "public pool" means a watertight structure of concrete, masonry, or other approved materials which is located either indoors or outdoors, used for bathing or swimming by humans, and filled with a filtered and disinfected water supply, together with buildings, appurtenances, and equipment used in connection therewith. A public swimming pool or public pool shall mean a conventional pool, spa-type pool, wading pool, special purpose pool, or water recreation attraction, to which admission may be gained with or without payment of a fee and includes, but is not limited to, pools operated by or serving camps, churches, cities, counties, day care centers, group home facilities for eight or more clients, health spas, institutions, parks, state agencies, schools, subdivisions, or the cooperative living-type projects of five or more living units, such as apartments, boardinghouses, hotels, mobile home parks, motels, recreational vehicle parks, and townhouses.

(3) "Private pool" means a facility used only by an individual, family, or living unit members and their guests which does not serve any type of cooperative housing or joint tenancy of five or more living units.

(4) "Public bathing place" means a body of water, natural or modified by humans, for swimming, diving, and recreational bathing used by consent of the owner or owners and held out to the public by any person or public body, irrespective of whether a fee is charged for the use thereof. The bathing water areas of public bathing places include, but are not limited to, lakes, ponds, rivers, streams, artificial impoundments, and waters along the coastal and intracoastal beaches and shores of the state.

(5) "Portable pool" means a pool or spa, and related equipment systems of any kind, which is designed or intended to be movable from location to location.

(6) "Temporary pool" means a pool intended to be used in conjunction with a sanctioned national or international swimming or diving competition event that does not exceed 30 consecutive days of use.

History.—ss. 1, 14, ch. 85-173; s. 4, ch. 91-429; s. 676, ch. 97-103; s. 77, ch. 97-237; s. 45, ch. 98-151; s. 1, ch. 2000-309; s. 103, ch. 2012-184; s. 10, ch. 2016-129.

514.0115 Exemptions from supervision or regulation; variances.—

(1) Private pools and water therapy facilities connected with facilities connected with hospitals, medical doctors' offices, and licensed physical therapy establishments shall be exempt from supervision under this chapter.

(2)(a) Pools serving no more than 32 condominium or cooperative units which are not operated as a public lodging establishment shall be exempt from supervision under this chapter, except for water quality.

(b) Pools serving condominium or cooperative associations of more than 32 units and whose recorded documents prohibit the rental or sublease of the units for periods of less than 60 days are exempt from supervision under this chapter, except that the condominium or cooperative owner or association must file applications with the department and obtain construction plans approval and receive an initial operating permit. The department shall inspect the swimming pools at such places annually, at the fee set forth in s. 514.033(3), or upon request by a unit owner, to determine compliance with department rules relating to water quality and lifesaving equipment. The department may not require compliance with rules relating to swimming pool lifeguard standards.

(3) A private pool used for instructional purposes in swimming shall not be regulated as a public pool.

(4) Any pool serving a residential child care agency registered and exempt from licensure pursuant to s. 409.176 shall be exempt from supervision or regulation under this chapter related to construction standards if the pool is used exclusively by the facility's residents and if admission may not be gained by the public.

(5) A portable pool used exclusively for providing swimming lessons or related instruction in support of an established educational program sponsored or provided by a school district may not be regulated as a public pool.

(6) A temporary pool may not be regulated as a public pool.

(7) The department may grant variances from any rule adopted under this chapter pursuant to procedures adopted by department rule. The department may also grant, pursuant to procedures adopted by department rule, variances from the provisions of the Florida Building Code specifically pertaining to public swimming pools and bathing places when requested by the pool owner or the pool owner's representative to relieve hardship in cases involving deviations from the Florida Building Code provisions, when it is shown that the hardship was not caused intentionally by the action of the applicant, where no reasonable alternative exists, and the health and safety of the pool patrons is not at risk.

History.—ss. 1, 14, ch. 85-173; s. 2, ch. 87-117; s. 46, ch. 98-151; s. 1, ch. 99-182; s. 13, ch. 2014-154; s. 67, ch. 2015-2; s. 11, ch. 2016-129.

514.021 Department authorization.—

(1) The department may adopt and enforce rules to protect the health, safety, or welfare of persons by setting sanitation and safety standards for public swimming pools and public bathing places. The department shall review and revise such rules as necessary, but not less than biennially. Sanitation and safety standards shall be limited to matters relating to source of water supply; microbiological, chemical, and physical quality of water in the pool or bathing area; method of water purification, treatment, and disinfection; lifesaving apparatus; and measures to ensure safety of bathers.

(2) The department may not establish by rule any regulation governing the design, alteration, modification, or repair of public swimming pools and bathing places which has no impact on sanitation and safety of persons using public swimming pools and bathing places. Further, the department may not adopt by rule any regulation governing the construction, erection, or demolition of public swimming pools and bathing places. It is the intent of the Legislature to preempt those functions to the Florida Building Commission through adoption and maintenance of the Florida Building Code. The department shall provide technical assistance to the commission in updating the construction standards of the Florida Building Code which govern public swimming pools. This subsection does not

abrogate the authority of the department to adopt and enforce appropriate sanitary regulations and requirements as authorized in subsection (1).

History.—ss. 2, 14, ch. 85-173; s. 65, ch. 87-225; s. 4, ch. 91-429; s. 49, ch. 2000-141; s. 48, ch. 2000-242; s. 27, ch. 2000-367; s. 34, ch. 2001-186; s. 3, ch. 2001-372; s. 104, ch. 2012-184.

514.023 Sampling of beach waters; and public bathing places; health advisories.—

(1) As used in this section, the term "beach waters" means the waters along the coastal and intracoastal beaches and shores of the state, and includes salt water and brackish water.

(2) The department may adopt and enforce rules to protect the health, safety, and welfare of persons using the beach waters and public bathing places of the state. The rules must establish health standards and prescribe procedures and timeframes for bacteriological sampling of beach waters and public bathing places.

(3) The department may issue health advisories if the quality of beach waters or a public bathing place fails to meet standards established by the department. The issuance of health advisories related to the results of bacteriological sampling of beach waters is preempted to the state.

(4) When the department issues a health advisory against swimming in beach waters or a public bathing place on the basis of finding elevated levels of fecal coliform, *Escherichia coli*, or enterococci bacteria in a water sample, the department shall concurrently notify the municipality or county in which the affected beach waters are located, whichever has jurisdiction, and the local office of the Department of Environmental Protection, of the advisory. The local office of the Department of Environmental Protection shall promptly investigate wastewater treatment facilities within 1 mile of the affected beach waters or public bathing place to determine if a facility experienced an incident that may have contributed to the contamination and provide the results of the investigation in writing or by electronic means to the municipality or county, as applicable.

History.—s. 2, ch. 2000-309; s. 1, ch. 2009-231; s. 105, ch. 2012-184.

514.0231 Advisory committee to oversee sampling of beach waters.— The Department of Health shall form an interagency technical advisory committee to oversee the performance of the study required in s. 514.023 and to advise it in rulemaking pertaining to standards for public bathing places along the coastal and intracoastal beaches and shores of the state. Membership on the committee shall consist of equal numbers of staff of the Department of Health and the Department of Environmental Protection with expertise in the subject matter of the study. Members shall be appointed by the State Surgeon General and the Secretary of Environmental Protection. The committee shall be chaired by a representative from the Department of Health.

History.—s. 5, ch. 2000-309; s. 22, ch. 2001-63; s. 109, ch. 2008-6.

514.025 Assignment of authority to county health departments.—

(1) The department shall assign to county health departments that are staffed with qualified engineering personnel the functions of reviewing applications and plans for the construction, development, or modification of public swimming pools or bathing places; of conducting inspections; and of issuing all permits. If the county health department determines that qualified staff are not available, the department shall be responsible for such functions.

(2) County health departments are responsible for routine surveillance of water quality in all public swimming pools and bathing places, including routine inspections, complaint investigations, enforcement procedures, and operating permits.

(3) The department may assign the responsibilities and functions specified in this section to any multicounty independent special district created by the Legislature to perform multiple functions, to include municipal services and improvements, to the same extent and under the same conditions as provided in subsections (1) and (2), upon request of the special district.

History.—s. 7, ch. 78-356; s. 2, ch. 81-318; ss. 3, 13, 14, ch. 85-173; s. 66, ch. 87-225; s. 4, ch. 91-429; s. 151, ch. 97-101; s. 2, ch. 2009-231; s. 106, ch. 2012-184.

Note.— Former s. 514.032.

514.028 Advisory review board.—

(1) The Governor shall appoint an advisory review board which shall meet as necessary or at least quarterly, to recommend agency action on variance request, rule and policy development, and other technical review problems. The board shall be comprised of:

(a) A representative from the office of licensure and certification of the department.

(b) A representative from the county health departments.

(c) Three representatives from the swimming pool construction industry.

(d) A representative from the public lodging industry.

(e) A representative from a county or local building department.

(2) The purpose of the advisory review board is to promote better relations, understanding, and cooperation between such industries and the department; to review and make recommendations regarding department product approval standards; to suggest means of better protecting the health, welfare, or safety of persons using the services offered by such industries; and to give the department the benefit of the knowledge and experience of the board concerning the industries and individual businesses affected by the laws and rules administered by the department.

(3) Members shall be reimbursed for travel expenses incurred in connection with service on the advisory review board pursuant to s. 112.061.

History.—ss. 8, 14, 15, ch. 85-173; ss. 4, 5, ch. 91-429; s. 152, ch. 97-101; s. 78, ch. 97-237; s. 17, ch. 2011-222.

514.03 Approval necessary to construct, develop, or modify public swimming pools or public bathing places.—

(1) A person or public body desiring to construct, develop, or modify a public swimming pool must submit an application, containing the information required under s. 514.031(1)(a)1.-6. to the department for an operating permit before filing an application for a building permit under s. 553.79. A copy of the final inspection required under s. 514.031(1)(a)5. shall be submitted to the department upon receipt by the applicant. The application shall be deemed incomplete pursuant to s. 120.60 until such copy is submitted to the department.

(2) Local governments or local enforcement districts may determine compliance with the general construction standards of the Florida Building Code, pursuant to s. 553.80. Local governments or local enforcement districts may conduct plan reviews and inspections of public swimming pools and public bathing places for this purpose.

History.—s. 2, ch. 7825, 1919; CGL 3769; ss. 19, 35, ch. 69-106; s. 3, ch. 76-168; s. 447, ch. 77-147; s. 1, ch. 77-457; ss. 2, 9, ch. 78-356; s. 2, ch. 81-318; ss. 4, 13, 14, ch. 85-173; s. 4, ch. 91-429; s. 47, ch. 98-151; s. 50, ch. 2000-141; s. 3, ch. 2000-309; s. 107, ch. 2012-184; s. 14, ch. 2014-154.

514.031 Permit necessary to operate public swimming pool.—

(1) It is unlawful for any person or public body to operate or continue to operate any public swimming pool without a valid permit from the department, such permit to be obtained in the following manner:

(a) Any person or public body desiring to operate any public swimming pool shall file an application for an operating permit with the department, on application forms provided by the department, and shall accompany such application with:

1. A description of the structure, its appurtenances, and its operation.

2. A description of the source or sources of water supply, and the amount and quality of water available and intended to be used.

3. The method and manner of water purification, treatment, disinfection, and heating.

4. The safety equipment and standards to be used.

5. A copy of the final inspection from the local enforcement agency as defined in s. 553.71.

6. Any other pertinent information deemed necessary by the department.

(b) The applicant shall respond to a request for additional information due to an incomplete application for an operating permit pursuant to s. 120.60. Upon receipt of an application, whether complete or incomplete, as required in s. 514.03 and as set forth under this section, the department shall review and provide to the local enforcement agency and the applicant any comment or proposed modifications on the information received pursuant to subparagraphs (a) 1.-6.

(c) If the department determines that the public swimming pool is or may reasonably be expected to be operated in compliance with this chapter and the rules adopted hereunder, the department shall grant the application for permit.

(d) If the department determines that the public swimming pool does not meet the provisions outlined in this chapter or the rules adopted hereunder, the department shall deny the application for a permit pursuant to the provisions of chapter 120. Such denial shall be in writing and shall list the circumstances for the denial. Upon correction of such circumstances, an applicant previously denied permission to operate a public swimming pool or bathing place may reapply for a permit.

(2) Operating permits shall not be required for coastal or intracoastal beaches.

(3) Operating permits may be transferred from one name or owner to another. When the ownership or name of an existing public swimming pool is changed and such establishment is operating at the time of the change with a valid permit from the department, the new owner of the establishment shall apply to the department, upon forms provided by the department, within 30 days after such a change.

(4) Each such operating permit shall be renewed annually and the permit must be posted in a conspicuous place.

(5) An owner or operator of a public swimming pool, including, but not limited to, a spa, wading, or special purpose pool, to which admittance is obtained by membership for a fee shall post in a prominent location within the facility the most recent pool inspection report issued by the department pertaining to the health and safety conditions of such facility. The report shall be legible and readily accessible to members or potential members. The department shall adopt rules to enforce this subsection. A portable pool may not be used as a public pool unless it is exempt under s. 514.0115.

History.—s. 7, ch. 78-356; s. 2, ch. 81-318; ss. 5, 13, 14, ch. 85-173; s. 4, ch. 91-429; s. 48, ch. 98-151; s. 49, ch. 2000-154; s. 4, ch. 2000-309; s. 108, ch. 2012-184; s. 15, ch. 2014-154; s. 13, ch. 2016-129.

514.0315 Required safety features for public swimming pools and spas.—

(1) A public swimming pool or spa must be equipped with an anti-entrapment system or device that complies with American Society of Mechanical Engineers/American National Standards Institute standard A112.19.8, or any successor standard.

(2) A public swimming pool or spa built before January 1, 1993, with a single main drain other than an unblockable drain must be equipped with at least one of the following features that complies with any American Society of Mechanical Engineers, American National Standards Institute, American Society for Testing and Materials, or other applicable consumer product safety standard for such system or device and protects against evisceration and body-and-limb suction entrapment:

(a) A safety vacuum release system that ceases operation of the pump, reverses the circulation flow, or otherwise provides a vacuum release at a suction outlet when a blockage is detected and that has been tested by an independent third party and found to conform to American Society of Mechanical Engineers/American National Standards Institute standard A112.19.17, American Society for Testing and Materials standard F2387, or any successor standard.

(b) A suction-limiting vent system with a tamper-resistant atmospheric opening.

(c) A gravity drainage system that uses a collector tank.

(d) An automatic pump shut-off system.

(e) A device or system that disables the drain.

(3) The determination and selection of a feature under subsection (2) for a public swimming pool or spa constructed before January 1, 1993, is at the sole discretion of the owner or operator of the public swimming pool or spa. A licensed contractor described in s. 489.105(3)(j), (k), or (l) must install the feature.

History.—s. 18, ch. 2011-222; s. 68, ch. 2012-5.

514.033 Creation of fee schedules authorized.—

(1) The department is authorized to establish a schedule of fees to be charged by the department or by any authorized county health department as detailed in s. 514.025. Fees assessed under this chapter shall be in an amount sufficient to meet the cost of carrying out the provisions of this chapter.

(2) The fee schedule shall be: for original construction or development plan approval, not less than $275 and not more than $500; for modification of original construction, not less than $100 and not more than $150; for an initial operating permit, not less than $125 and not more than $250; and for review of variance applications, not less than $240 and not more than $400. The department shall assess the minimum fees provided in this subsection until a fee schedule is promulgated by rule of the department.

(3) Fees shall be based on pool aggregate gallonage, which shall be: up to and including 25,000 gallons, not less than $75 and not more than $125; and in excess of 25,000 gallons, not less than $160 and not more than $265, except for a pool inspected pursuant to s. 514.0115(2)(b) for which the annual fee shall be $50.

(4) Fees collected by the department in accordance with this chapter shall be deposited into the Grants and Donations Trust Fund or the County Health Department Trust Fund. Any fee collected under this chapter is nonrefundable.

(5) The department may not charge any fees for services provided under this chapter other than those fees authorized in this section. However, the department shall prorate the initial annual fee for an operating permit on a half-year basis.

History.—s. 7, ch. 78-356; s. 2, ch. 81-318; s. 9, ch. 83-230; ss. 6, 13, 14, ch. 85-173; s. 1, ch. 87-117; s. 4, ch. 91-429; s. 9, ch. 96-407; s. 225, ch. 97-101; s. 49, ch. 98-151; s. 109, ch. 2012-184.

514.04 Right of entry.— For the purpose of this chapter, department personnel at any reasonable time may enter upon any and all parts of the premises of such public swimming pools and bathing places to make an examination and investigation to determine the sanitary and safety conditions of such places.

History.—s. 3, ch. 7825, 1919; CGL 3770; ss. 19, 35, ch. 69-106; s. 3, ch. 76-168; s. 448, ch. 77-147; s. 1, ch. 77-457; ss. 3, 9, ch. 78-356; s. 2, ch. 81-318; ss. 7, 13, 14, ch. 85-173; s. 4, ch. 91-429.

514.05 Denial, suspension, or revocation of permit; administrative fines.—

(1) The department may deny an application for a permit, suspend or revoke a permit issued to any person or public body, or impose an administrative fine upon the failure of such person or public body to comply with the provisions of this chapter or the rules adopted hereunder.

(2) The department may impose an administrative fine, which shall not exceed $500 for each violation, for the violation of this chapter or the rules adopted hereunder and for the violation of any of the provisions of chapter 386. Notice of intent to impose such fine shall be given by the department to the alleged violator. Each day that a violation continues may constitute a separate violation.

(3) In determining the amount of fine to be imposed, if any, for a violation, the following factors shall be considered:

(a) The gravity of the violation and the extent to which the provisions of the applicable statutes or rules were violated.

(b) Actions taken by the operator to correct violations.

(c) Any previous violations.

(4) All amounts collected pursuant to this section shall be deposited into the Grants and Donations Trust Fund or into the County Health Department Trust Fund, whichever is applicable.

(5) Under conditions specified by rule, the department may close a public pool that is not in compliance with this chapter or the rules adopted under this chapter.

History.—s. 4, ch. 7825, 1919; CGL 3771; ss. 19, 35, ch. 69-106; s. 3, ch. 76-168; s. 449, ch. 77-147; s. 1, ch. 77-457; ss. 4, 9, ch. 78-356; s. 2, ch. 81-318; ss. 9, 13, 14, ch. 85-173; s. 4, ch. 91-429; s. 153, ch. 97-101; s. 50, ch. 98-151; s. 110, ch. 2012-184.

514.06 Injunction to restrain violations.— Any public swimming pool or public bathing place presenting a significant risk to public health by failing to meet sanitation and safety standards established pursuant to this chapter is declared to be a public nuisance, dangerous to health or safety. Such nuisances may be abated or enjoined in an action brought by the county health department or the department.

History.—s. 5, ch. 7825, 1919; CGL 3772; ss. 19, 35, ch. 69-106; s. 139, ch. 71-355; s. 3, ch. 76-168; s. 450, ch. 77-147; s. 1, ch. 77-457; ss. 5, 9, ch. 78-356; s. 2, ch. 81-318; ss. 10, 13, 14, ch. 85-173; s. 4, ch. 91-429; s. 154, ch. 97-101; s. 111, ch. 2012-184.

514.071 Certification of swimming instructors and lifeguards required.—

(1) Any person working as a swimming instructor or lifeguard at a public swimming pool must be certified by the American Red Cross, the Y.M.C.A., or other nationally recognized aquatic training programs. Swimming instructors must be currently certified in swimming instruction, first aid, and cardiopulmonary resuscitation. Lifeguards must be currently certified in lifeguarding, first aid, and cardiopulmonary resuscitation.

(2) In addition to any other remedies available to the department, the department may sue to enjoin the operation of any public swimming pool that uses any swimming instructor or lifeguard in violation of subsection (1).

(3) The department shall adopt rules necessary to implement this section which shall include, but not be limited to, defining the terms "swimming instructor," "lifeguard," and "nationally recognized aquatic training program."

History.—ss. 1, 3, ch. 90-47; s. 4, ch. 91-429.

514.072 Certification of swimming instructors for people who have developmental disabilities.— Any person working at a swimming pool who holds himself or herself out as a swimming instructor specializing in training people who have developmental disabilities, as defined in s. 393.063, may be certified by the Dan Marino Foundation, Inc., in addition to being certified under s. 514.071. The Dan Marino Foundation, Inc., must develop certification requirements and a training curriculum for swimming instructors for people who have developmental disabilities. A person certified under s. 514.071 must meet the additional certification requirements of this section within 6 months after receiving certification under s. 514.071.

History.—s. 1, ch. 2006-153; s. 69, ch. 2012-5; s. 18, ch. 2013-162.

514.075 Public pool service technician; certification.— The department may require that a public pool, as defined in s. 514.011, be serviced by a person certified as a pool service technician. To be certified, an individual must demonstrate knowledge of public pools which includes, but is not limited to: pool cleaning; general pool maintenance; source of the water supply; bacteriological, chemical, and physical quality of water; and water purification, testing, treatment, and disinfection procedures. The department may, by rule, establish the requirement for the certification course and course approval. The department shall deem certified any individual who is certified by a course of national recognition or any person licensed under s. 489.105(3)(j), (k), or (l). This requirement does not apply to a person, or the direct employee of a person, permitted as a public pool operator under s. 514.031.

History.—s. 19, ch. 96-298; s. 73, ch. 96-388.

Disclaimer: The information on this system is unverified. The journals or printed bills of the respective chambers should be consulted for official purposes.

Copyright © 2000- 2019 State of Florida.
