# Supplementary material for: Assessing state partner use of the Model Aquatic Health Code (MAHC): A cross comparison of five states with varying degrees of self-reported adoption status
Source: PLOS Water. Author manuscript; Available in PMC 2025 Jul 11. (PMC12247143; doi:10.1371/journal.pwat.0000276)
Supplement: S5_Text — S5 Text. Georgia pool codes. The Georgia Department of Public Health rules and regulations for public swimming pools, spas, and recreational water parks, Chapter 511–3-5 established in 2017. (PDF) [file NIHMS2046419-supplement-S5_Text.pdf]

Georgia Department of Public Health

Rules and Regulations
Public Swimming Pools, Spas, and Recreational Water Parks
Chapter 511-3-5

2017

We Protect Lives.

RULES OF
DEPARTMENT OF PUBLIC HEALTH

CHAPTER 511-3-5
PUBLIC SWIMMING POOLS, SPAS, AND RECREATIONAL WATER PARKS

TABLE OF CONTENTS

511-3-5-.01 Definitions
511-3-5-.02 Scope
511-3-5-.03 Permits
511-3-5-.04 Structural Design
511-3-5-.05 Dimensional Design
511-3-5-.06 Decks and Deck Equipment
511-3-5-.07 Circulation Systems
511-3-5-.08 Filters
511-3-5-.09 Pumps and Motors
511-3-5-.10 Return Inlets and Suction Outlets
511-3-5-.11 Surface Skimmer Systems
511-3-5-.12 Lighting and Electrical Requirements
511-3-5-.13 Heaters and Temperature Requirements
511-3-5-.14 Air Blower and Air Induction Systems
511-3-5-.15 Water Supply and Wastewater Disposal
511-3-5-.16 Disinfectant Equipment and Chemical Feeders
511-3-5-.17 Chemical Operational Parameters
511-3-5-.18 Specific Safety Features and Markers
511-3-5-.19 Dressing Facilities and Sanitary Facilities
511-3-5-.20 Recreational Water Parks and Special Purpose Pools
511-3-5-.21 Food Service
511-3-5-.22 Operation and Management
511-3-5-.23 Compliance Procedures
511-3-5-.24 Environmental Health Personnel

511-3-5-.01 Definitions.

The following definitions shall apply in the interpretation and enforcement of this chapter.

(1) “Abandoned Pool” means a public pool that has not been permitted or not in operation for at least four calendar years.

(2) "Abrasion Hazard" means a sharp or rough surface that would scrape the skin by chance during normal use.

(3) "Accessible" means easily exposed for inspection and for the replacement of materials or parts with the use of tools.

(4) "Active Area" means those water areas in pools which are three feet or less in water depth.

(5) "Air Induction System" means a system whereby a volume of air (only) is induced into a hollow duct in a spa floor, bench or other location. The air induction system is activated by a separate air power blower.

(6) "Air Pump Assist Backwash" means the compression of air in the filter effluent chamber (by means of an air compressor or by the water pressure from the recirculating pump) which, when released, rapidly decompresses and forces water in the filter chamber through the elements in reverse, dislodging the filter aid and accumulated dirt, carrying it to waste.

(7) "Alkalinity" means the amount of bicarbonate, carbonate or hydroxide compounds present in water solution. See also "Total Alkalinity."

(8) “Aquatic Facility” means a physical place that contains one or more public swimming pools and support infrastructure.

(9) “Aquatic Feature” means an individual component within a public pool. Examples include slides, structures designed to be climbed or walked across by bathers, and structures that create falling or shooting water.

(10) “Automated Controller” means a system of at least one chemical probe, a controller, and auxiliary or integrated component that senses the level of one or more water parameters and provides a signal to other equipment to maintain the parameters within a user-established range.

(11) "Backwash" means the process of thoroughly cleansing the filter medium, elements, and the contents of the filter vessel by the reverse flow of water through the filter.

(12) "Barrier" means a fence, safety cover, wall, building wall or a combination thereof, which completely surrounds or covers the swimming pool or spa and obstructs access to the swimming pool, spa or recreational water park.

(13) "Bather" means any person who uses a swimming pool, spa, or recreational water park, or adjoining deck areas for the purpose of water sports, recreation, therapy, or related activities.

(14) "Booster or Jet Pump System" means a system whereby one or more hydrotherapy jets are activated by the use of a pump which is completely independent of the filtration and heating system of a spa. It may also mean a device used to provide hydraulic support for certain types of equipment such as cleaning systems, gas chlorinators and solar systems.

(15) "Breakpoint Chlorination" means the conversion of inorganic chloramine compounds to nitrogen gas by reaction with free available chlorine. The point at which the drop occurs is referred to as the “breakpoint”. The amount of free chlorine that must be added to the water to achieve breakpoint chlorination is approximately ten times the amount of combined chlorine in the water.

(16) "Brominator" means a device to apply or to deliver a bromine disinfectant to water at a controlled rate.

(17) "Cartridge" means a pleated or surface-type filter component with fixed dimensions that is designed to remove suspended particles from water flowing through the filter.

(18) "Chemical Feeder" means a mechanical device for applying chemicals to pool or spa water.

(19) "Chloramine" means a compound formed when chlorine combines with nitrogen or ammonia that causes eye and skin irritation and has a strong, objectionable odor.

(20) "Chlorinator" means a device to apply or to deliver a chlorine disinfectant to water at a controlled rate.

(21) "Chlorine Generator" means equipment that generates chlorine, hypochlorous acid or hypochlorite on site for disinfection and oxidation of water contaminants.

(22) "Circulation Equipment" means the mechanical components that are part of a circulation system in a pool or spa. Circulation equipment includes, but is not limited to, pumps, hair and lint strainers, filters, valves, gauges, meters, heaters, surface skimmers, inlet/outlet fittings, and chemical feeding devices. These components may have separate functions, but when connected to each other by piping, perform as a coordinated system for purposes of maintaining pool and spa water in a clear, sanitary and desirable condition.

(23) "Circulation System" means an arrangement of mechanical equipment or components, connected by piping to a pool or spa in a closed circuit. The function of a circulation system is to direct water from the pool or spa, causing it to flow through the various system components for purposes of clarifying, heating, purifying and returning the water back to the original body of water.

(24) "Clarifier" means a chemical that coagulates and neutralizes suspended particles in water, such as inorganic salts of aluminum or iron and water-soluble organic polyelectrolyte polymers. Also called coagulant or flocculent.

(25) "Contact Concentration" means the concentration of a chemical in a flow of water. This concentration depends on the rate of addition, the flow rate of the water and the efficiency of the mixing. It is calculated using the equation (assumes complete mixing): Amount of Chemical (gpm)/Water Flow Rate (gpm) x 4.41 = Contact Concentration (mg/L).

(26) “Combined chlorine” means the reaction of free chlorine with ammonia and nitrogen compounds to form chloramines.

(27) “Contamination Response Plan” means a plan for handling contamination from formed-stool, diarrheal-stool, and vomit.

(28) "Coping" means the cap on the pool or spa wall that provides a finishing edge around the pool or spa, whether formed, cast in place, pre-cast concrete, or pre-fabricated from metal or plastic materials.

(29) "Country Club" means a location with facilities for outdoor sports and social activities for which members pay a membership fee other than a daily fee, periodically for the use of facilities and services by them and their guests. Fraternal organizations may be included in this definition.

(30) "Cove" means the radius between the pool or spa wall and the pool or spa floor.

(31) “CT Value” means a representation of the concentration of the disinfectant (C) multiplied by time in minutes (T) needed for inactivation of a particular contaminant. The concentration and time are inversely proportional; therefore, the higher the concentration of the disinfectant, the shorter the contact time required for inactivation. The CT value can vary with pH or temperature change so these values must also be supplied to allow comparison between values.

(32) "Cyanuric Acid" means a chemical that helps reduce the excess loss of chlorine in water due to the ultraviolet rays of the sun. It is also called stabilizer, isocyanuric acid, conditioner or triazinetrione.

(33) "Decks" means those areas immediately adjacent to or attached to a pool or spa that are intended for bathers to sit, stand, or walk upon. It connects the pool to adjacent amenities, entrances, and exits. This area is expected to be regularly trafficked and made wet by bathers.

(34) "Deep Areas" means water depths in excess of five feet.

(35) "Department" means the Georgia Department of Public Health.

(36) "Diatomite" means the filtering medium of a diatomaceous earth filter composed of microscopic fossil skeletons of the "diatom," a tiny freshwater marine plankton.

(37) "Disinfectant" means an agent used to kill undesirable or pathogenic (disease-causing) organisms that have a measurable residual at a level adequate to make the desired kill.

(38) "Diving Board" means a recreational mechanism for entering a swimming pool, consisting of a semi rigid board that derives its elasticity through the use of a fulcrum mounted below the board. This term includes, without limitation, a “jump board” with a coil spring, leaf spring or comparable device located beneath the board which is activated by the force exerted in jumping on the board, and a “stationary diving platform” used for diving and constructed or located on site, including natural or artificial rocks, pedestals or other items.

(39) "DPD (Diethyl-p-phenylene Diamine)" means a reagent and test method that specifically measure bromine or free available and total chlorine, producing a series of colors from pale pink to dark red.

(40) "Effective Filter Area" means total surface area through which the designed flow rate will be maintained during filtration.

(41) "Effluent" means the water that flows out of a filter, pump or other device.

(42) “EPA Registered” means all products regulated and registered under the Federal Insecticide, Fungicide, and Rodenticide Act (FIFRA) by the U.S. Environmental Protection Agency EPA registered products will have a registration number on the label which can be verified by using the EPA National Pesticide Information Retrieval System.

(43) “Equipment Room” means a space intended for the operation of pool pumps, filters, heaters and controllers.

(44) "Feet of Head" means a basis for indicating the resistance in a hydraulic system, equivalent to the height of a column of water that would cause the same resistance (100 feet of head equals 43 pounds per square inch).

(45) "Filter" means a device that removes undissolved particles from water by recirculating the water through a porous substance (a filter medium or element).

(46) "Filter Element" means a device within a filter tank designed to entrap solids and conduct water to a manifold, collection header, pipe, or similar conduit and return it to the pool or spa. A filter element usually consists of a septum and septum support or a cartridge.

(47) "Free Available Chlorine (FAC)" means the portion of the total available chlorine that is not “combined chlorine” and is present as hypochlorous acid (HOCl) or hypochlorite ion (OCl-) and will react chemically with undesirable or pathogenic organisms.

(48) “Flume” means the riding channels of a waterslide which accommodate riders using or not using mats, tubes, rafts, and other transport vehicles as they slide along a path lubricated by a water flow.

(49) "Handhold/Handrail" means a device that can be gripped by a user for the purpose of resting or steadying him/herself. It is not limited to but may be located inside or outside the pool or spa or as part of a set of steps or deck-installed equipment.

(50) "Hardness" means the amount of calcium and magnesium dissolved in water; measured by a test kit and expressed as parts per million (ppm) of equivalent calcium carbonate.

(51) "Health Authority" means both the Georgia Department of Public Health and the County Board of Health - Environmental Health Office.

(52) "Hydrotherapy Spa or Spa” means a unit that may have a therapeutic use but which is not drained, cleaned, or refilled for each individual. It may include, but not be limited to, hydrotherapy jet circulation, hot water/cold water mineral baths, air induction bubbles or any combination thereof. Industry terminology for a spa includes, but is not limited to, "therapeutic pool," "hydrotherapy pool," "whirlpool," and "hot spa."

(53) “Imminent Health Hazard” means a product, practice, circumstance, event or condition that requires immediate correction or cessation of operation in order to prevent a significant threat of danger or death, injury or illness.

(54) “Increased Risk Public Pool” means a public pool which, due to its intrinsic characteristics and intended users, has a greater likelihood of microbial contamination. An increased-risk public pool includes spray pads, wading pools, and others designed for children less than five years old.

(55) "Influent" means the water entering a filter or other device.

(56) "In ground Swimming Pool" means any pool where the sides rest in partial or full contact with the earth.

(57) “Lifeguard” means an individual who has successfully completed a recognized lifeguard training course, holds a current certificate for such training, has met the pre-service requirements, and is participating in continuing in-service training requirements of the facility.

(58) “Modification” means changes or repairs to equipment, interior finishes, or fixtures on a public swimming pool that will not disturb or remove any portion of the plumbing, decking, or watertight shell structure.

(59) “Monitoring” means the regular and purposeful observation and checking of systems or facilities and recording of data, including system alerts, excursions from acceptable ranges, and other facility issues. Monitoring includes human or electronic means.

(60) "Multi -port Filter-Control Valve" means a multiport valve having a number of control positions for various filter operations that combines in one unit the function of two or more single valves.

(61) "Non-swimming Area" means any portion of a pool where water depth, offset ledges, or similar irregularities prevents normal swimming activities.

(62) "Organic Matter" means perspiration, urine, saliva, suntan oil, cosmetics, lotions, dead skin, and similar debris introduced to water by users and the environment.

(63) "Orthotolidine (OTO)" means a colorless reagent that reacts with chlorine or bromine to produce a series of yellow-to-orange colors which indicate the amount of chlorine or bromine in water.

(64) "“Oxidation Reduction Potential” means a measure of the tendency for a solution to either gain or lose electrons; higher (more positive) oxidation reduction potential indicates a more oxidative solution.

(65) "Overflow System" means a system for the removal of pool/spa surface water through the use of overflows, surface skimmers and surface water collection systems of various design and manufacture.

(66) “Patron” means a bather or other person or occupant at a public pool who may or may not have contact with water either through partial or total immersion. Patrons may not have contact with the water, but who might still be exposed to potential contamination from the facility’s air, surfaces, or aerosols.

(67) “Peninsula / Wing Wall” means a structural projection into a pool intended to provide partial separation within the body of water.

(68) "pH" means the negative log of the concentration of hydrogen ions. As pH is raised, more ionization occurs and chlorine disinfectants decrease in effectiveness.

(69) "Pool" means any artificial water holding structure with a closed-loop circulation of water through a water treatment system with a return to the structure.

(70) "Private Pool" means any constructed pool, permanent or non-portable, that serves a single-family dwelling and is used only by the residents of that dwelling and their guests.

(71) “Public Swimming Pool" means any structure, chamber, or tank containing an artificial body of water shared and used by the public for swimming, diving, wading,

recreation or therapy, together with buildings, appurtenances and equipment used in connection with the body of water, regardless of whether a fee is charged for its use. The term includes municipal, school, hotel, or motel pools, and any pool to which access is granted in exchange for payment of a daily fee. The term shall also include pools and spas operated by or serving camps, churches, day care centers, group home facilities of twelve or more clients, institutions, parks, state agencies, condominiums, mobile home parks, recreational vehicle parks, associations, health clubs, special purpose pools, and recreational water parks. Public swimming pools are divided into the following classifications:

(a) Class “A” means a pool intended for use for accredited competitive swimming events such as Federation Internationale De Natation (FINA), USA Swimming, USA Diving, National Collegiate Athletic Association (NCAA) and National Federation of State High School Associations (NFHS) and other governing bodies. The use of such a pool is not limited to competitive events.

(b) Class “B” means a pool intended for general public recreational use.

(c) Class “C” means a pool operated solely for and in conjunction with lodging and housing such as hotels, motels, campgrounds and multi-residential housing.

(d) Class “D” means special purpose pools (see special purpose pool types).

(e) Class “E” means pools or spas used for instruction, play, or therapy, and having a water temperature above 90 degrees ^{0}F (32 degrees ^{0}C).

(72) "Special Purpose Pools” means any pool operated for recreational play and other special purposes, including, but not limited to, wave or surf action pools, activity pools, interactive water activity pools, wading pools, and activity pools. These include, but are not limited to the following types:

(a) Activity Pools. A pool designed for casual water play ranging from simple splashing activity to the use of attractions placed in the pool for recreation. This includes, but is not

limited to slides, flumes, lilypad walks, log rolls, cable, rope, boom drops, and any other falling entry features. These pools allow for the bather to drop into the pool area from a height of six inches to four feet above the water surface and in various positions of entry.

(b) Continuous Water Course. A manufactured stream of water of near-constant depth in which water is moved by pumps or other propulsion to provide a flow that transports bathers over a defined path.

(c) Diving Pool. A pool used exclusively for diving.

(d) Dual Use Pool. A pool that is normally used as a swimming pool, but which has no more than one water slide or one other feature other than diving boards which uses the main body of water as its landing or activity area.

(e) Exercise Spa. A variant of a spa in which the design and construction includes specific features and equipment to produce a water flow intended to allow recreational physical activity including but not limited to, biking and treadmills. Spas can include peripheral jetted seats intended for water therapy, heater, circulation and filtration system, which must be separate and distinct from the spa and must have separate controls.

(f) Interactive Water Play Pool. A pad which contains various fountains, interactive water sprays, or waterfall features. The pad slopes to one or more drains which empties into a reservoir which is recirculated and disinfected before its return to the water features. These pools are also known as splash pads, spray pads, wet decks. For the purposes of the Chapter, only those designed to recirculate water and intended for public use and recreation shall be regulated.

(g) Landing Pool. A pool located at the exit of one or more waterslide flumes. The body of water is intended and designed to receive a bather emerging from the flume for the purpose of terminating the slide action and providing a means of exit to a deck or walkway area.

(h) Leisure River. A riding water course where ingress and egress is effectively limited to designated points of entry and exit, also known as a lazy river.

(i) Sensory Deprivation Chamber (float tank). A chamber that provides a light and sound free environment, contains a saturated solution of sodium chloride or magnesium sulfate having a specific gravity of 1.27 to 1.3, and is maintained at a temperature of approximately 93.5°F (34.1°C).

(j) Wading Pool. A shallow pool with a depth of 18 inches or less, and which has no water activity features.

(k) Wading Interactive Pool. A pool with a depth of 18 inches or less and which has any number of water features within the pool area.

(l) Wave Pool. A large body of water that has a mechanism for generating an oscillating wave-form at one end and ending at the other end with a zero-depth-entry.

(73) Zero Depth Entry Pools. A pool in any classification that has a sloping edge or beach at one end in place of a wall.

(74) "Permanently Installed Swimming Pool" means a pool that is constructed in the ground or in a building in such a manner that it cannot be readily disassembled for storage.

(75). "Therapeutic Pool" means a pool used in physical programs operated by medical facilities licensed by the Department of Community Health or operated by a licensed physical therapist.

(76) “Pool Slide” means a slide having a configuration as defined in the Code of Federal Regulations (CFR) Ch. II, Title 16 Part 1207 of the Consumer Product Safety Commission, or which is similar in construction to a playground slide designed to allow users to slide from an elevated height to a pool. The term includes children’s (tot) slides and all other non-flume slides that are mounted on the pool deck or within the basin of a public swimming pool.

(77) "Potable Water" means any water, such as an approved domestic water supply, which is microbiologically safe and otherwise suitable for drinking.

(78) "PPM" means an abbreviation for parts per million, the unit of measurement used in chemical testing which indicates the parts by weight in relation to one million parts by weight of water, such as the term milligrams per liter (mg/L).

(79) "Precipitate" means a solid material which is forced out of a solution by some chemical reaction and which may settle out or remain as a haze in suspension (turbidity).

(80) "PSI" means an abbreviation for pounds per square inch.

(81) "Rate of Flow" means the quantity of water flowing past a designated point within a specified time, such as the number of gallons flowing in one minute (gpm).

(82) "Rated Pressure" means that pressure that is equal to or less than the designed pressure and appears on the data plate of the equipment.

(83) "Recreational Water Park" means a facility or area which incorporates one or more special purpose pools, together with associated buildings, appurtenances, and equipment designated for public bathing or swimming.

(84) "Removable" means something that can be disassembled with the use of simple tools such as a screwdriver, pliers or wrench.

(85) "Remodeling" means the activity of restoring all or part of the physical structure of a pool or spa into good condition. This includes the rebuilding or replacing of worn and broken parts or components that require disturbing segments of the piping system, decking, or watertight shell structure.

(86) “Responsible Person” means an individual that is responsible for daily water monitoring operations when a “trained operator” is not on-site or making visits to the public swimming pool daily.

(87) "Return Inlet" means the opening or fitting through which the water under positive pressure returns into a pool or spa.

(88) "Return Piping" means that piping through which water is returned to the pool.

(89) "Ring Buoy" means a ring-shaped floating buoy capable of supporting a bather in the water.

(90) “Rinse Shower” means a shower typically located on the deck area and supplied with ambient temperature water. The main purpose is to remove dirt, sand, or organic material prior to entering the water to reduce the introduction of contaminants and the formation of disinfection by-products.

(91) "Shallow Area" means portions of a pool or spa with water depths five feet or less.

(92) “Safety Plan” means a written document that has procedures, requirements, or standards related to safety which the pool staff shall follow. The safety plan shall include training and emergency response procedures.

(93) “Sanitize” means reducing the level of microbes to that level considered safe by public health standards. This may be achieved through a variety of chemical or physical means including chemical treatment, physical cleaning, or drying.

(94) “Saturation Index” means a mathematical representation or scale representing the ability of water to deposit calcium carbonate, or dissolve metal, concrete or grout.

(95) "Shock Treatment" means the practice of adding significant amounts of an oxidizing chemical to water to destroy ammonia and nitrogenous and organic contaminants in water.

(96) "Skimmer Weir" means the part of a skimmer which adjusts automatically to small changes in water level to assure a continuous flow of water to the skimmer.

(97) "Slip Resistant" means a surface that has been treated or constructed so as to significantly reduce the chance of a patron slipping. The surface shall not be an abrasion hazard. All surfaces required to be slip-resistant shall have a minimum dynamic

coefficient of friction at least equal to the requirements of ANSI A137.1-2012 or successor standard for that installation as measured by the DCOF AcuTest.

(98) "Sodium Hypochlorite (NaOCl)" means a clear liquid form of an inorganic chlorine compound obtainable in concentrations of 5% to 16% available chlorine.

(99) **“**Special Use Pool” means a pool that does not meet the operational and design characteristics of any public swimming pool class or type identified elsewhere in this chapter. A special use pool may be considered through an application for a variance.

(100) "Suction Outlet" means the opening or fitting through which the water under negative pressure is drawn from the pool or spa.

(101) "Suction Piping" means that piping through which water is removed from the pool.

(102) "Surface Skimming System" means perimeter-type overflows, surface skimmers and surface water collection systems of various design and manufacture which permit the continuous removal of floating debris and surface water to the filter.

(103) “Supplemental Disinfection Systems” means those disinfection processes or systems installed in addition to the primary system required on all increased risk public pools.

(104) "Test Kit" means a device used to monitor specific chemical or agent residual or demands in pool or spa water.

(105) “Theoretical Peak Occupancy Load” means the anticipated peak number of bathers in the water and on the deck. This occupancy is also used for design purposes and to determine services that support bathers. For public swimming pools, the theoretical peak occupancy load is calculated by using the water density factor and deck area:

(a) Flat Water. A public swimming pool in which the water line is static except for movement made by users usually as a horizontal use as in swimming. Diving spargers do not void the flat water definition.

(b) Agitated Water. A public swimming pool with mechanical means (aquatic features) to discharge, spray, or move the water's surface above or below the static water line of the pool so people are standing or playing vertically. Where there is no static water line, movement shall be considered above the deck plane.

(c) Hot Water. A pool or spa with a water temperature over 90ºF (32ºC).

(106) "Time Clock" means a mechanical device that automatically controls the periods that a pump, filter, chlorinator, heater, blower and other electrical devices are running.

(107) "Total Alkalinity" means the ability or capacity of water to resist change in pH; also known as the buffering capacity of water. Measured with a test kit and expressed as ppm.

(108) "Total Available Chlorine (TAC)" means the sum of both the free available and combined chlorines.

(109) “Trained Operator” means an individual responsible for the operation and maintenance of the public pool water and the associated infrastructure of the facility who has successfully completed a Department approved operator training course.

(110) "Turbidity" means the cloudy condition of water due to the presence of extremely fine particulate materials in suspension that interfere with the passage of light.

(111) "Turnover Rate" means the period of time (usually in hours) required to circulate a volume of water equal to the pool or spa capacity.

(112) “Underwater Seat Bench” means a submerged seat without hydrotherapy jets.

(113) "Vacuum" means the reduction of atmospheric pressure within a pipe, tank, pump or other vessel. Vacuum is measured in inches of mercury. One inch (1") of mercury is equivalent to one and thirteen hundredths feet (1.13') of head. The practical maximum vacuum is thirty inches (30") of mercury or thirty three and nine tenths feet (33.9') of head.

(114) "Waterline". The waterline shall be defined in one of the following ways:

(a) Skimmer System. The waterline shall be at the midpoint of the operating range of the skimmers when there are no bathers in the pool or spa.

(b) Overflow System. The waterline shall be at the top of the overflow rim.

(115) Water slide. A slide that runs into a landing pool or runout through a fabricated channel with flowing water. A water slide may be classified by their physical and intended use characteristics. The following are types of waterslides:

(a) Body Slide. A water slide used without a vehicle.

(b) Children's Slide. A water slide generally intended only for use by persons under the height of 48 inches. Water slide has a maximum fall distance of 3 inches from slide exit where the rider enters the water and water depth is no more than 24 inches.

(c) Mat Slides. Water slide used with a designated mat as a vehicle.

(d) Specialty Slides. A proprietary water slide design, such as an uphill, half-pipe, or bowl ride, which does not conform to the standard classification.

(e) Speed Slide. Water slide where the riders achieve a velocity of 25 feet per second or more during the course of the ride.

(f) Tub Slides. Water slide used with a single or multi-person water slide tube.

(116) "Water Quality Testing Device" means a product designed to measure the level of a parameter in water. A WQTD includes a device or method to provide a visual indication of a parameter level, and may include one or more reagents and accessory items.

[Authority: O.C.G.A. Secs. 31-2A-6, 31-12-8, 31-45-10.]

511-3-5-.02 Scope.

(1) These rules prescribe minimum design, construction, and operation requirements for the protection of public health and safety of the public in swimming pools, spas, and recreational water parks.

Rule .02

(2) These rules are intended to cover certain aspects of the design, equipment, operation, permanent installation, new construction and remodeling of swimming pools, spas and recreational water parks. Where adequate standards do not exist and these rules do not provide sufficient guidance for consideration of innovations in design, construction and operation of proposed pools, spas or recreational water parks, the Department will establish requirements necessary to protect the health and safety of the pool patrons.

(3) These rules shall not apply to private swimming pool and hot tubs or spas serving a single-family dwelling and used only by the residents of that dwelling and their guests, apartment complex pools, country club pools, subdivision pools which are open only to residents of the subdivision and their guests, therapeutic pools operated by a licensed medical facility or a licensed physical therapist, therapeutic chambers which are drained, cleaned, and refilled after each individual use, or religious ritual baths used solely for religious purposes.

(4) This Chapter shall apply to those counties where local rules and regulations governing public swimming pools were not in effect on 31 December 2000; provided, however, that the governing authority of a county or municipality may by ordinance or resolution elect to make apartment pools within their jurisdiction subject to this Chapter.

(5) All pools shall meet the requirements of this Chapter except as provided in subsection (6) below.

(6) Public swimming pools constructed prior to December 31, 2016 shall continue to be governed by the 2013 version of this Chapter with regard to design and construction requirements. Any such pool that is remodeled after 31 December 2016 shall be required to comply with the current version of this Chapter. Notwithstanding the

foregoing, all pools shall be required to meet the current requirements of this Chapter related to the abatement of suction hazards.

(7) All single, dual, or multiple drain covers and grates shall comply with ANSI/APSP-16 or any successor standard that may be prescribed by ANSI/APSP.

[Authority: O.C.G.A. Secs. 31-2A-6, 31-12-8, 31-45-10.]

511-3-5-.03 Permits.

(1) **General Provisions.**

(a) It shall be unlawful for any person to operate a public pool, spa, or recreational water park without having first obtained a valid operating permit from the health authority pursuant to this Chapter. Each pool must operate under a separate permit.

(b) It shall be unlawful for any person to build, alter, or remodel a public pool, spa, or recreational water park without first having obtained a valid construction permit from the health authority pursuant to this Chapter.

(c) Prior to the issuance of any permit under these rules, the applicant shall provide evidence of satisfactory compliance with the provisions of this Chapter and all other laws which apply to the location, construction, and maintenance of the pool, spa, or recreational water park, and the safety of persons therein.

(d) All permit applications shall be prepared in duplicate on forms provided by the Department. The original shall be filed with the health authority for the county in which the pool is located and a copy retained by the applicant.

(2) **Construction Permits.**

(a) Two complete sets of scaled construction plans must be submitted to the local health authority for approval when a public swimming pool, spa, or recreational water park attraction is to be constructed, altered, or remodeled, or when an existing or

abandoned structure is to be converted for use as a public pool, spa, or recreational water park attraction. The plans shall be submitted at least thirty days prior to beginning construction and shall indicate, at a minimum, the proposed layout, the mechanical plans, the construction materials, and the type and model of proposed equipment.

(b) The theoretical peak occupancy shall be stated on the plans.

(c) One approved set of the construction plans shall remain at the construction site at all times during construction, and all contractors must have access to the plans.

(d) The construction plans must bear the seal of a licensed architect or professional engineer, unless the health authority deems it to be unnecessary.

(e) Complete specifications for the project shall accompany the plans, including manufacturer's cut sheets and specifications on all equipment.

(f) A professional engineer licensed in the state must sign the department hydraulic analysis form and submit the completed worksheet with the construction plans, unless the health authority deems it to be unnecessary.

(g) Any additional data required by the health authority for purpose of clarification, anticipated use, or to support any changes in design or scope of the project, must be submitted before issuance of a construction permit.

(h) The swimming pool, spa, or recreational water park shall be built in compliance with the plans as approved unless written approval of changes has been given by the health authority.

(i) A construction permit is valid for twelve months from the date of issue. If the project has not been completed within that time, then the owner must apply to renew the permit.

(j) The owner or its agent shall notify the health authority at specific, predetermined stages of construction and at the time of completion of the pool to allow inspections.

Rule .03(2)

(k) The health authority may make or require third party inspections of any new or existing construction work to determine compliance with the provisions of this chapter and other ordinances or laws.

(3) **Operating Permit.**

(a) The permit shall be prominently displayed at all times, as close to the main entrance as practicable or as determined by the health authority.

(b) An operating permit shall not be valid for more than twelve months.

(c) An operational permit will not be issued to a facility if any violation of this Chapter is found during the permitting inspection, if applicable, written evidence of compliance with other state laws or local ordinances is not provided at the time of inspection, or if any outstanding fees are due.

(d) The owner must provide the health authority with a letter from a licensed architect, or professional engineer stating that the pool was built in compliance with the regulation and applicable codes. This letter need only be provided once.

(e) Copies of any testing reports for systems, such as air handling, are conducted, shall be furnished with the application.

[Authority: O.C.G.A. Secs. 31-2A-6, 31-12-8, 31-45-10.]

511-3-5-.04 Structural Design.

(1) Pools shall be constructed of reinforced concrete or impervious and structurally sound materials, which provide a smooth, easily cleaned, watertight structure capable of withstanding the anticipated loads for full and empty conditions, taking into account climatic and hydrostatic considerations, and the integration of the pool with other structures. The structural design and materials used shall be in accordance with generally accepted structural engineering practices.

Rule .04

(2) Pool shell construction material may also include fiberglass, stainless steel and modular panel systems meeting the requirements of this section and any applicable American Society of Testing and Materials standards or state building code.

(3) Sand or earth shall not be permitted as an interior finish in a swimming pool or spa.

(4) The pool or spa shell, appurtenances, piping, filter system, pump and motor, and other components shall be constructed to facilitate protection from damage due to freezing.

(5) Surfaces within the pool or spa intended to provide footing for users shall be designed to provide a slip-resistant surface that is rigid and resistant to puncture and tear.

(6) Polyvinyl chloride (PVC) membrane systems may be used as an interior finish of a public pool if the supporting watertight pool shell to which the system is attached meets the structure requirements of the chapter. If the structure complies with the chapter, the contractor may permanently attach an approved PVC membrane or panel system to all surfaces within the pool. A PVC membrane shall be a minimum of 55 mils in thickness.

(7) The roughness or irregularity of such surfaces shall not be such as to cause injury or discomfort to the feet during normal use.

(8) The color of the interior shall be white or light pastel and shall not obscure the presence of a bather on the bottom of the pool, or of objects, debris, algae, or surface cracks within the pool.

(9) Swimming pools and spas and their appurtenances shall be constructed of materials which are nontoxic to man and the environment, impervious and enduring, able to withstand design stresses, and able to provide a watertight structure with a smooth and easily cleaned surface without cracks or joints, excluding structural joints, or to which a smooth, easily cleaned surface finish is applied or attached. Materials of manufacture for

swimming pools and spas shall be capable of fulfilling the design, installation, and intended use requirements in these rules. The materials of manufacture, components and accessories used in public spas shall comply with the following:

(a) **Plumbing.** All plumbing shall be sized, installed, and maintained according to applicable State Regulations and local plumbing codes. Written evidence shall be provided from a licensed plumbing contractor or the plumbing inspector, as required by the local health authority, of compliance with the plumbing code.

(b) **Electrical Systems.** All electrical wiring, equipment, and installation, including the grounding of pool components, shall conform with national, state and local electrical codes. Written evidence shall be provided from a licensed electrical contractor or electrical inspector, as required by the local health authority, of compliance with all electrical codes.

(c) **Recirculation and Treatment Systems and other Components.** All recirculation and treatment system equipment and all other components such as filters, recessed automatic surface skimmers, ionizers, ozone generators, solar heaters, disinfection feeders, chlorine generators and sensory deprivation chambers or float pods must be tested and approved using the current NSF Standard 50, Circulation System Components and Related Materials for Swimming Pool, Spas/Hot Tubs." Written evidence shall be provided from the designing engineer that all recirculation and treatment systems and all components used in the installation meet these approved standards.

(d) **Material Surfaces.** All surfaces that come in contact with the user shall be finished so that they do not constitute a cutting, pinching, puncturing or abrasion hazard under casual contact and intended use. All materials shall be maintained in accordance with manufacturer's instructions.

Rule .04(9)

(e) **Compatibility**. Combinations of different materials shall be chemically and mechanically compatible for their intended use and environment. Any pool with a metal-based shell or utilizing dissimilar metals shall be provided with sacrificial anodes or other approved means to reduce galvanic action and electrolytic corrosion.

(f) **Ventilation.** Mechanical ventilation shall be provided for all indoor public swimming pools and pump rooms. All systems shall be sized, installed and maintained according to applicable state regulations and local codes. Written evidence shall be provided by a licensed heating, ventilation and air-conditioning contractor certifying compliance with all applicable codes and with the latest ANSI/ASHRAE standard 62.1, Ventilation for Acceptable Indoor Air Quality. A written statement of commissioning shall be provided to the owner stating that the amount of outdoor air meets the performance requirements of the applicable codes.

(10) Roofs or canopies over pools or spas shall be constructed so that water run-off does not drain into the pool or spa water.

[Authority: O.C.G.A. Secs. 31-2A-6, 31-12-8, 31-45-10.]

511-3-5-.05 Dimensional Design.

(1) Swimming pools, spas, and recreational water park attractions may be constructed in any shape that is safe and which allows for adequate circulation of the water.

(a) There shall be no protrusions, extensions, or other means of entanglement or obstructions in the swimming area which can cause the entrapment or injury of the bather.

(b) There shall be construction tolerances allowed on all dimensional designs. Overall length, width and depth in the deep end of a swimming pool may vary plus or minus three inches. All other overall dimensions in a swimming pool and in a spa may vary

plus or minus two inches, unless otherwise specified. The designed waterline shall have a maximum construction tolerance at the time of completion of the work of plus or minus one-fourth inch for pools and spas with adjustable weir surface skimming systems and of plus or minus one-eighth inch for pools and spas with nonadjustable surface skimming systems.

(c) The pool size shall be governed by the requirements of the activities for which the installation is intended.

(2) **Walls.** Walls shall not be more than eleven degrees from plumb for a minimum depth of two feet nine inches from the waterline in deep areas or two feet three inches in the shallow areas. Below these depths the wall may be radiused to join the floor. The finished construction tolerance for the wall slope shall be ±3 percent.

(3) **Floor Slopes.** Floor slopes shall comply with the following minimum standards:

(a) All slopes shall be uniform.

(b) The slope of the floor from the shallow end wall towards the deep end shall not exceed one foot in twelve feet to the point of the first slope change.

(c) The point of the first slope change shall be defined as the point at which the floor slope exceeds one foot in twelve feet and shall not occur at a depth of more than five feet.

(d) The slope of the floor from the point of the first slope change to the deep end shall not exceed one foot in three feet. Such slopes may not provide any less water depth than those specified if the pool is intended for diving.

(e) Transitional radius from wall to floor where floor slopes join the wall shall comply with the following:

1. The radius shall have its center no less than two feet nine inches below the waterline in deep areas or two feet six inches in the shallow area.

Rule .05(3)(e)

2. The radius shall be tangent at the point where the radius either meets the wall or the floor.

3. The radius (R) shall be at least equal to or greater than the depth of the pool minus the vertical wall depth measured from the waterline or tolerance allowed in DPH Rule 511-3-5-.05 (2) minus three inches to allow draining to the main drain. (R minimum = Pool depth - Vertical wall depth - 3")

4. Walls shall intersect with the floor at an angle or a transition profile. Where a transitional profile is provided at water depths of three feet or less, a transitional radius shall not exceed six inches and shall be tangent to the wall and is permitted to be tangent to or intersect the floor.

(4) **Water depths.** Water depths at the shallow end of the swimming area shall be a maximum of three feet six inches except for competitive racing pools.

(a) The active area of a pool shall be visually set apart from, but may be adjoined to, the shallow area and shall not adjoin the deep area.

(b) The transition point or point of slope change of the pool from the active area to the shallow area and from the shallow area to the deep area and at the points of separation of diving, slide and amusement areas shall be visually set apart with a rope and float line, depth markers and a four inch minimum width row of floor tile, or similar means of a color contrasting with the bottom. In diving pools with a constant slope, the shallow area shall be visually set apart from the deep area with a rope and float line, depth markers and a four inch minimum width row of floor tile, or similar means of a color contrasting with the bottom. The health authority may waive the need for a rope and float line in swim-out areas or similar construction where deemed necessary.

(c) Starting platforms built after the adoption of this chapter shall be installed according to manufacturer’s instructions and this section.

Rule .05(4)(c)

1. Starting platforms shall be installed in a minimum water depth of five feet.

2. The leading edge of starting platforms shall have a maximum height of 30 inches above the water surface.

3. Platforms shall have slip resistant tread surfaces.

(d) Starting platforms shall be used by swimmers certified for racing starts and under the direct supervision of a qualified coach or instructor.

(e) Starting platforms shall be removed, if possible, or prohibited from use during all recreational or non-competitive swimming activity by covering platforms with a manufacturer-supplied platform cover or with another means or device that is readily visible and clearly prohibits use.

(5) Diving areas in non-competitive pools shall conform to the minimum water depths, areas, slopes and other dimensions shown in DPH Rule 511-3-5-.05(7). If a wall exists, then it shall conform to the 5:1 slope in the Point D dimension and the $L_{1-2-3-4}$ dimensions and shall be installed in accordance with the manufacturer's instructions.

(6) Installation and use instructions for manufactured diving equipment shall be provided by the manufacturer and shall specify the minimum water dimensions required for each diving board and diving stand combination. The manufacturer's instructions shall refer to the water envelope type by dimensionally relating their products to Point A on the water envelopes referenced in subsection (b) below. The board manufacturer shall specify which boards fit on the design pool geometry types.

(a) When diving equipment is installed, it shall conform to the specifications set forth in DPH Rule 511-3-5-.06(7) and shall be located in the diving area of the pool so as to provide the minimum dimensions as shown in DPH Rule 511-3-5-.05(7).

(b) The tip of the diving equipment shall be located at Point A shown in the diagram in DPH Rule 511-3-5-.05(7)(a), which is the reference point of all other dimensions.

Rule .05(6)

(c) There shall be a completely unobstructed clear vertical distance of thirteen feet above any diving board measured from the center of the front end of the board. This area shall extend horizontally at least eight feet behind, eight feet to each side and sixteen feet ahead of Point A shown in the diagram in DPH Rule 511-3-5-.05(7)(a).

(d) Public non-competitive pools with diving facilities in excess of three meters in height, or pools designed for platform diving, shall comply with the dimensional design requirements of the Federation Internationale de Natation Amateur (FINA), U.S. Diving, National Federation of State High School Associations (NFSHSA), or similar authority.

(7) **Minimum Dimensions for Diving Portion of Pools.**

(a) Diagram showing points where dimensions are measured. Note that the shallow portion of the pool is not shown.

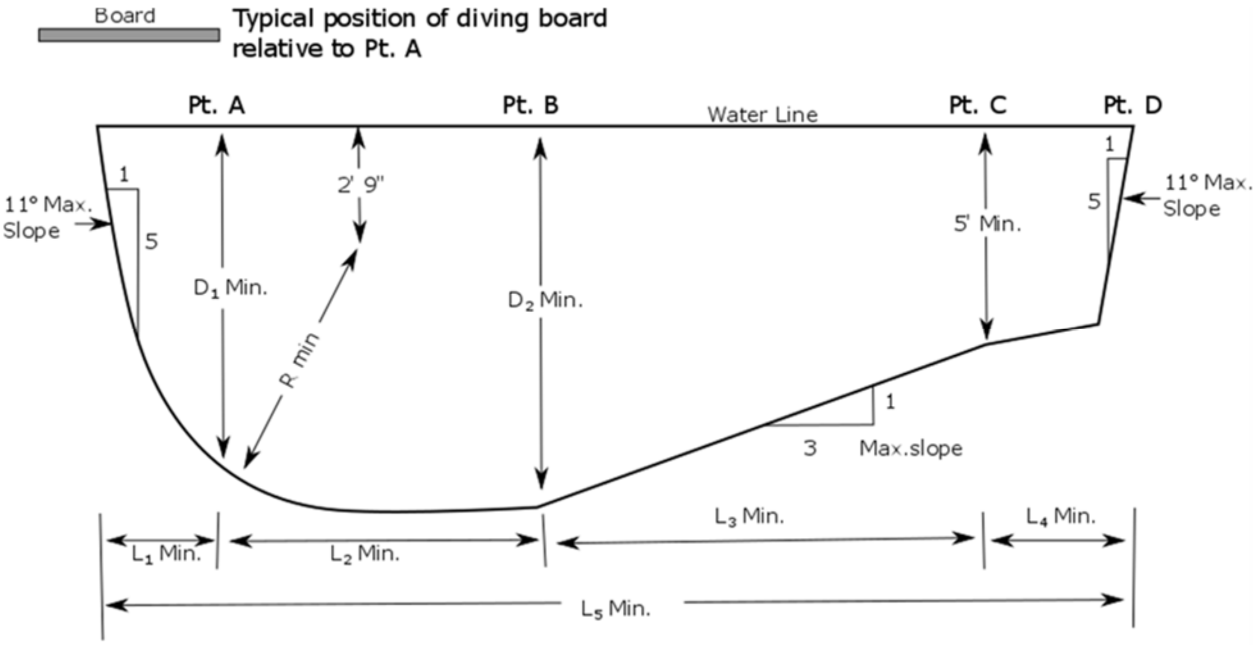

Note: L_4 is a minimum dimension to allow sufficient length opposite the board. This may of course be lengthened to form the shallow portion of the pool.

Rule .05(7)

(b) Minimum dimensions for points given in diagram (a).

RELATED DIVING EQUIPMENT		MINIMUM DIMENSIONS								MINIMUM WIDTH OF POOL AT:		
MAX DIVING BOARD LENGHT	MAX BOARD HEIGHT OVER WATER	D_1	D_2	R	L_1	L_2	L_3	L_4	L_5	PT.A	PT.B	PT.C
10’	26”(2/3meter)	7’-0”	8’-6”	5’-6”	2’-6”	8’-0”	10’-6”	7’-0”	28’-0”	16’-0”	18’-0”	18’-0”
12’	30”(3/4 meter)	7’-6”	9’-0”	6’-0”	3’-0”	9’-0”	12’-0”	4’-0”	28’-0”	18’-0”	20’-0”	20’-0”
16’	1 Meter	8’-6”	10’-0”	7’-0”	4’-0”	10’-0”	15’-0”	2’-0”	31’-0”	20’-0”	22’-0”	22’-0”
16’	3 Meter	11’-0”	12’-0”	8’-6”	6’-0”	10’-6”	21’-0”	0	37’-6”	22’-0”	24’-0”	24’-0”

1. L2, L3, and L4 combined, represent the minimum distance from the tip of the board to pool wall opposite diving equipment.

2. Placement of boards shall observe the following minimum dimensions. With multiple board installations minimum pool widths must be increased accordingly.

- Deck Level Board to Pool Side 8'
- 1 Meter Board to Pool Side 10'
- 3 Meter Board to Pool Side 11'
- 1 Meter or Deck Level Board to 3 Meter Board 10'
- 1 Meter or Deck Level Board to another 1 Meter or Deck Level Board 8'
- 3 Meter to another 3 Meter Board 10'

(8) **Offset Ledges.** When provided, offset ledges shall fall within eleven degrees from plumb starting at the junction of the pool wall and waterline and shall have a slip-resistant surface. The outer two inch edge shall be lined with slip resistant tile in a contrasting color. The maximum width shall be eight inches. The typical allowable dimensions are based on the depths shown below.

Rule .05(8)

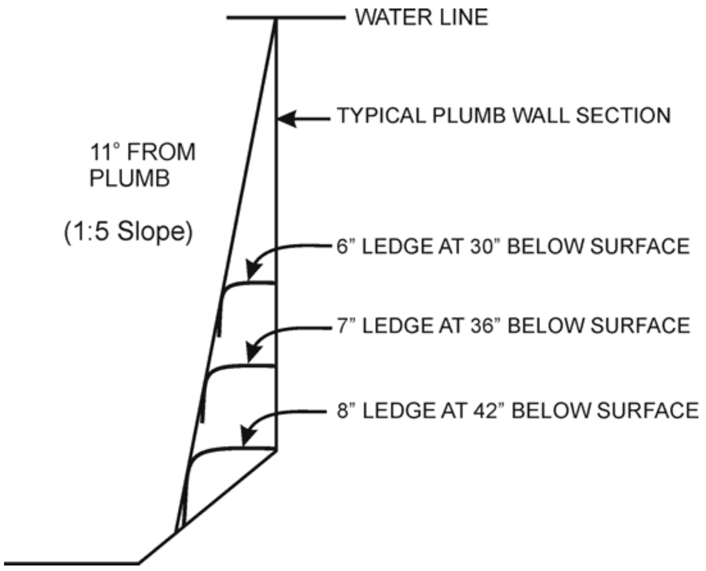

(9) **Underwater Seat Benches.** Underwater seat benches in pools, if provided, shall have a maximum horizontal seat bench depth of twenty inches below the waterline, be visually set apart by having the outer two inches of each seat lined with a slip-resistant tile in a contrasting color, and shall be located fully outside of the required minimum diving water envelope if the pool is intended for use with diving equipment. .

(10) **Swimouts.** Swimouts shall be located in the shallow area of a pool outside of the perimeter and comply with all the following:

(a) The horizontal surface shall be not be more than twenty inches below waterline.

(b) An unobstructed surface shall be provided that is equal to or greater than that required for the top tread of the pool stairs in DPH Rule 511-3-5-.06(3).

(c) Where used as an entry and exit access, swimouts shall be provided with steps that comply with the pool stair requirement in DPH Rule 511-3-5-.06(3).

(d) The leading two inches of the outer edge shall be visually set apart with slip resistant tiles in a contrasting color.

Rule .05

(11) **Underwater/Tanning Shelf.** An underwater shelf used as the required entry or exit access shall be located not more than twelve inches below the waterline.

The leading two inches of the outer edge shall be visually set apart with contrasting tiles.

The shelf surface area is excluded when determining the occupancy load.

(12) **Theoretical Peak Occupancy Load.** The theoretical peak occupancy for a public swimming pool shall be used for designing systems that serve bathers, and shall incorporate non-water related areas such as decking. This peak occupancy shall be the total number of bathers that are permissible on the deck and in the water at any given point in time.

(a) The theoretical peak occupancy shall be calculated by dividing the pool area in square feet by the density factor (D) representing the specific water types or devices and pool deck area.

(b) Use Table 1. Density Factors (D) in square feet to determine the theoretical peak occupancy.

Rule .05(12)(b)

Table 1. Density Factors (D) in square feet

	Agitated Water in Shallow area or Shallow water or Wading area	Flat Water in Deep area or Deep water (not including the Diving Area)	Diving Area (per each diving board)	Entry Area for all other devices including slides
Pools with minimal deck area	15 square feet per user	20 square feet per user	300 square feet per diving board	150 square feet per device
Pools with deck area at least equal to the water surface area	12 square feet per user	15 square feet per user	300 square feet per diving board	150 square feet per device
Pool with deck area at least twice the water surface area	10 square feet per user	12 square feet per user	300 square feet per diving board	150 square feet per device

(c) The theoretical peak occupancy calculations shall be calculated by adding the sums of the applicable figures from Table 1.

(d) A spa or hot water venue density factor shall not exceed one bather per ten square feet of surface area.

(e) A waterslide landing pool may use the manufacturer established capacity if given.

(13) **Wading Pool Water Depth.** Wading pools constructed after adoption of this chapter shall be separate and physically set apart from beginning or shallow water areas of swimming pools by at least fifteen feet of deck. Where a wading pool is adjacent to

any deep water area, a minimum four foot high barrier shall be installed to separate the two pools.

(a) Wading pools shall have a maximum water depth of eighteen inches. Water depths may be reduced from the above maximums and brought to zero at the most shallow point. The areas where the water depth at the edge of the pool exceeds nine inches shall be considered as non-entry areas.

(b) Walls in wading pools shall be vertical or within 11^0 of vertical except for the lower six inches which shall be radiused to the floor. Walls shall not extend more than six inches above the waterline at any point.

(c) Floors of wading pools shall be uniform and sloped to drain with a maximum slope of one foot in twelve feet vertical to horizontal.

(14) **Spa Water Depth.** The maximum water depth in a spa shall be four feet measured from the waterline. Exceptions may be made for spas designed for a special purpose.

(a) Multi-level seating in a spa may be provided, but the maximum water depth of any seat or sitting bench shall be twenty-eight inches measured from the waterline.

(b) The spa shall be provided with a suitable handhold around its perimeter in areas where water depths exceed three feet six inches. Handholds shall be provided no more than four feet apart and may consist of any one or a combination of the following options:

1. Coping, ledges, radiused flanges or decks along the immediate top edge of the spa shall provide a suitable slip-resistant handhold located not more than twelve inches above the waterline; or

2. Ladders, steps or seat ledges; or

3. A secured rope or railing at or not more than twelve inches above the waterline.

Rule .05(14)

(c) The slope of the floor in a spa shall not exceed one foot in twelve feet vertical to horizontal.

[Authority: O.C.G.A. Secs. 31-2A-6, 31-12-8, 31-45-10.]

511-3-5-.06 Decks and Deck Equipment.

(1) **Decks**. These requirements shall apply to all decks and deck equipment at the time of construction.

(a) Decks shall be designed and installed in accordance with the engineering practices required in the area of installation. Decks shall be constructed with a uniform and easily cleaned surface such as concrete, tile, manufactured or acrylic surfaces. This includes the design of sub base when required, concrete mix design, reinforcing, and joints, if a concrete deck is selected. In the absence of specific local engineering practices, the work shall be performed in accordance with the recommended practices of the latest edition of American Concrete Institute (ACI) Standard 302.1R-80, "Guide for Concrete Floor and Slab Construction or successor standard."

(b) Decking shall be flush with the lip of the pool, spa walls, and copings. Decks, ramps, coping and similar step surfaces shall be slip-resistant and easily cleanable.

(c) Special features in or on decks, such as markers or brand insignias, shall conform to this Chapter.

(d) Risers for steps for the deck shall be uniform and have a minimum height of three and three-fourths inches and a maximum height of seven and one-half inches. The minimum tread depth shall be twelve inches.

(e) Backfilled areas that support a deck shall be adequately compacted.

(f) The deck, including coping, shall have a minimum four feet width of continuous, unobstructed walking area maintained at all times unless otherwise allowed in the chapter.

(g) A minimum four foot deck width shall be provided on the sides and rear of any diving equipment or waterslide stairs. A deck clearance of forty-eight inches shall be provided around all deck equipment.

(h) The approved decking shall connect all site amenities, entrances, and exits.

(i) A four foot minimum continuous unobstructed deck, which may include the coping, shall be provided around at least 50 percent or more of a spa.

(j) The minimum slope of the decks shall be one-eighth inch per one foot vertical to horizontal.

(k) The maximum voids between adjoining concrete slabs, or between concrete slabs and expansion joint material, shall be three-sixteenths inch of horizontal clearance with a maximum difference in vertical elevation of one-fourth inch.

(l) Open joints or gaps larger than three-sixteenths inch wide or with vertical elevations exceeding 1/4 inches shall be rectified using appropriate fillers. Construction joints where pool coping meets the decks shall be watertight and shall not allow water to pass to the ground beneath.

(m) The areas where the decks join the pool and spa coping shall be designed and installed so as to protect the coping and its mortar bed from damage as a result of anticipated movement of adjoining decks.

(n) Joints in decks shall be provided to minimize the potential for cracks due to a change in elevations, separation of surfaces or movement of the slab.

(o) The areas where the decks join concrete work shall be protected by expansion joints to protect the pool adequately from the pressures of relative movements.

Rule .06(1)

(p) Decks shall be edged, have a radius, or be otherwise relieved to eliminate sharp corners.

(q) Decks shall be sloped to effectively drain either to perimeter areas or to deck drains. Drainage shall remove pool and spa splash water, deck cleaning water, and rain waterwithout leaving standing water of more than one-eighth inch depth twenty minutes after the cessation of the addition of water to the deck.

(r) Site drainage shall be provided to direct all perimeter deck drainage as well as general site and roof drainage away from the pool. When required, yard drains shall be installed to prevent the accumulation or puddling of site water in the general area of the decks and related improvements.

(s) There shall be no direct connection between the deck drains and the sanitary or storm sewer system, or the gutter or skimmer recirculation system.

(t) Wing walls or peninsulas less than eighteen inches in width shall not be considered a part of the deck.

(u) If a backwash sump is used, then an open pit or leaching design for backwash sump purposes shall be located so that it falls completely below adjacent decks and fully between a line projected 45^0 downward and away from such decks, or shall be designed to accommodate local soil conditions and the volume of backwash.

Rule .06(1)(u)

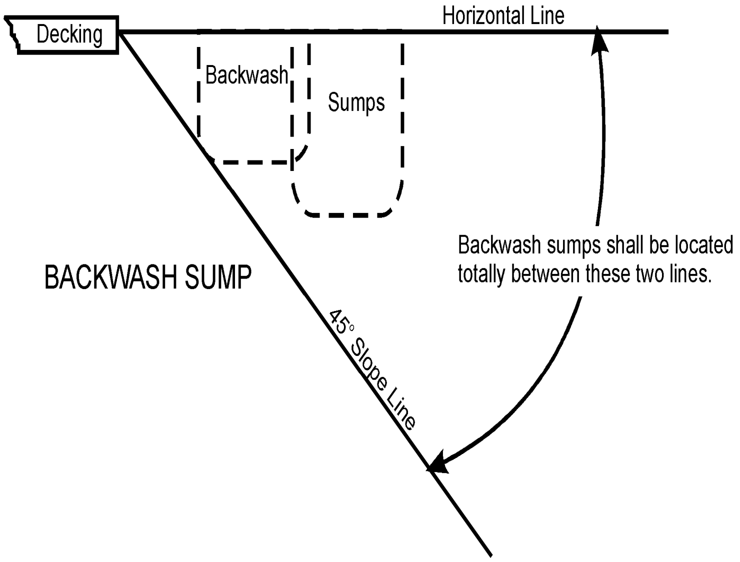

(v) Circulation system piping, other than that integrally included in the manufacture of the pool or spa, shall be subject to an induced static hydraulic pressure test (sealed system) at twenty-five pounds per square inch (psi) for at least fifteen minutes or longer if required by the local code official or health authority. This test shall be performed before the deck is poured and the pressure shall be maintained through the deck pour.

(w) Valves installed in or under any deck shall provide a minimum ten inch diameter access cover and valve pit to facilitate servicing.

(x) A hose bib and a vacuum breaker shall be provided for washing down the entire deck area and shall be located not more than one hundred-fifty feet apart. Water-powered lifts shall have a dedicated hose bib water source.

(y) The deck area will be kept clean of all trash and debris.

(z) Carpet, wood and artificial turf may not be used on the deck adjoining the pool. Additionally, loose plant material or bedding shall not be permitted on the deck area within four feet of the water surface area.

(2) **Entry/Exit.** All pools, except spas, shall have at least two means of entry/exit located so as to serve both ends of the pool and the deepest portion. These shall

consist of ladders, stairs or recessed treads, or a walking entry, and may be used in combination. All treads shall have slip-resistant surfaces. Handicapped accessible entry/exit into the pool shall be designed and provided in accordance with federal, state or local requirements.

(a) Where water depths are twenty-four inches or less at the pool wall, such areas shall be considered as providing their own natural mode for entry/exit.

(b) For pools or water areas over thirty feet in width, each side of the deepest portions of the pool shall have its own entries/exits.

(c) For pools with water depths of more than five feet, a means of entry/exit for the shallow end shall be located between the shallow end wall and the cross section at Point C, while a means of entry/exit for the deep end shall be between the deep end wall and the cross section at Point B as shown in DPH Rule 511-3-5-.05(7).

(d) A means of entry/exit shall be provided at a minimum of every seventy-five linear feet of pool wall or fraction thereof.

(e) Stairs, ladders and recessed treads shall be located to not interfere with racing lanes if applicable.

(3) **Pool Stairs.** The design and construction of protruding and recessed pool stairs shall conform to the following:

(a) Step treads shall have a minimum unobstructed horizontal depth of ten inches and a minimum unobstructed surface area of two hundred forty square inches.

(b) Risers at the centerline of the treads shall have a maximum uniform height of twelve inches, with the bottom riser height allowed to vary from the floor to not more than twelve inches.

(c) The vertical distance from the pool coping, deck, or step surface to the uppermost tread shall not be greater than twelve inches.

(d) Where stairs are located in water depths of more than forty-eight inches, the lowest tread shall be not less than forty-eight inches below the deck and the stairs shall not protrude into the pool. The stairs shall be set back into the pool wall.

(e) The outer two inches of each step shall be marked with slip resistant tiles in a contrasting color.

(f) Each set of stairs shall be provided with at least one handrail to serve all treads and risers. Handrails shall conform to the following standards:

1. Handrails, if removable, shall be installed in such a way that they cannot be removed without the use of tools.

2. The leading edge of handrails facilitating stairs and pool entry/exit shall be no more than eighteen inches plus or minus three inches, horizontally from the vertical plane of the bottom riser (where applicable).

3. The outside diameter of handrails shall be between one and one quarter inch and two inches.

(g) Underwater seats, benches or swimouts may be provided as part of the stairs or recessed treads.

(h) Stairs wider than five feet shall have at least one additional handrail for every twelve feet of stair width or fraction.

(4) **Pool Ladders.** The design and construction of pool ladder(s) shall conform to the following standards:

(a) Pool ladders shall be made entirely of corrosion-resisting materials.

(b) Ladders shall provide two handholds or two handrails.

(c) Below the water level, there shall be a clearance of not less than three inches and not more than six inches between any ladders tread edge, measured from the pool wall side of the tread and the pool wall.

Rule .06(4)

(d) The clear distance between ladder handrails shall be a minimum of seventeen inches and a maximum of twenty-four inches.

(e) There shall be a uniform height between ladder treads, with a seven inch minimum distance and a twelve inch maximum distance.

(f) Ladder treads shall have a minimum horizontal depth of two inches.

(5) **Recessed Treads.** The design and construction of recessed treads in the pool wall shall conform to the following standards:

(a) Recessed treads at the centerline shall have a uniform vertical spacing of twelve inches maximum and seven inches minimum.

(b) The vertical distance between the pool coping edge, deck or step surface and the uppermost recessed tread shall be a maximum of twelve inches.

(c) Recessed treads shall have a minimum depth of five inches and a minimum width of twelve inches.

(d) Recessed treads shall drain into the pool to prevent the accumulation of dirt.

(e) Each set of recessed treads shall be provided with a set of handrails, grabrails, or handholds to serve all treads and risers.

(f) The clear distance between handrails and grab rails shall be between seventeen and twenty-four inches.

(6) **Spa Entry/Exit.** Spas shall have a means of entry/exit at a minimum of every fifty feet or portion thereof, where water depths are more than twenty-four inches.

(a) DPH Rule 511-3-5-.06(4) and (5) shall apply to ladders and recessed treads in spas.

(b) Spas shall be equipped with at least one handrail (or ladder equivalent) for each fifty feet of perimeter or portion thereof, to designate the point of entry and exit.

1. Handrails shall be installed in such a way that they cannot be removed without the use of tools.

Rule .06(6)(b)

2. The leading edge of a handrail in the spa shall be no more than eighteen inches plus or minus three inches horizontally from the vertical plane of the bottom riser (where applicable).

3. The outside diameter of handrails shall be between one and one-quarter inch and two inches.

(c) The design and construction of spa steps and seat benches, where used, shall conform to the following standards:

1. Step treads shall have a minimum unobstructed horizontal depth of ten inches for a minimum continuous width of twelve inches. Step treads shall have slip-resistant surfaces.

2. Riser heights shall not be more than twelve inches. Where the bottom tread serves as a bench or seat, the bottom riser may be a maximum of fourteen inches above the spa floor.

3. The first and last risers need not be uniform but shall comply with riser height requirements as noted above. The (top riser is measured from the finished deck.

4. Intermediate risers, those between the first and last risers, shall be uniform in height.

5. Each set of steps shall be provided with at least one handrail to serve all treads and risers.

6. The outer two inch edge of each step shall be marked with slip resistant tiles in   a contrasting color.

(7) **Supports for Diving Equipment.** Supports, platforms, stairs and ladders for diving equipment shall be designed to carry the anticipated loads. Stairs and ladders shall be of corrosion-resisting material, easily cleanable and with slip-resistant tread.

Rule .06(7)

(a) All diving stands higher than twenty-one inches as measured from the deck to the top butt end of the board shall be provided with stairs or a ladder. Step treads shall be self-draining.

(b) Platforms and diving equipment of one meter or higher shall be protected with guard rails which shall be at least thirty inches above the diving board and extend to the edge of the pool wall. All platforms or diving equipment higher than one meter shall have guard rails which are at least thirty-six inches above the diving board and extend to the edge of the pool wall.

(8) **Diving Equipment.** Diving equipment shall be designed for swimming pool use and shall be installed in accordance with the manufacturer's recommendations.

Diving boards shall be permitted only when the diving envelope conforms to the standards of the certifying agency that regulates competitive diving at the facility or, if designed for noncompetitive diving, shall follow this section.

(a) Diving equipment manufacturers shall provide installation instructions and specifications with each unit.

(b) A label shall be permanently affixed to the diving equipment or jump board and shall include:

1. Manufacturer's name, identification and address,
2. board equipment length,
3. identification as to diving or jump board,
4. fulcrum setting specifications (if applicable),
5. minimum water envelope required for each diving board and diving stand combination,
6. date of manufacture, and
7. maximum weight of the user.

(c) Diving equipment shall have slip-resistant tread surfaces.

(d) Diving equipment shall be permanently anchored to the pool deck. The edge of the board at the tip end shall be level with the water surface. The tip end of the board over the pool water surface may be higher than the butt end of the board.

(e) Maximum board height over the water shall have plus three inches tolerance.

(f) The maximum construction tolerance of the tip of the board from Point A as shown in DPH Rule 511-3-5-.05(7) shall be plus or minus three inches. The diving equipment shall be in compliance with DPH Rule 511-3-5-.05(6).

(9) **Pool slides.** The requirements of the U.S. Consumer Product Safety Commission Standard for Swimming Pool Slides as published in the Code of Federal Regulations, 16 CFR Part 1207, shall be used for standards relating to swimming pool slides. Installation and use instructions shall be provided with each unit by the manufacturer.

(10) **Play structures and other equipment.** Play structures and other equipment shall meet all requirements set by appropriate authorities such as building codes, the U.S. Consumer Product Safety Commission, ASTM standards, and amusement ride regulations.

(11) **Bridges**. Bridges spanning a pool or any other structures not intended for interactive play shall have a minimum clearance of seven feet from the bottom of the structure to the bottom of the pool and a minimum height of four feet above the water surface. A bridge shall have a minimum forty-two inch high barrier on both sides and a slip resistant walking surface constructed of concrete or a non-absorbent material. A “no diving and no-jumping” sign shall be placed at both ends of the bridge.

[Authority: O.C.G.A. Secs. 31-2A-6, 31-12-8, 31-45-10.]

511-3-5-.07 Circulation Systems.

(1) A circulation system consisting of pumps, piping, return inlets and suction outlets, filters and other necessary equipment shall be provided for complete circulation of water through all parts of the pool.

(a) The equipment for a swimming pool shall be of adequate size to turn over the entire pool water capacity. The turnover rate for pools constructed after the adoption of this chapter shall not exceed the sum of one and one-half times the average water depth in feet; where the number of hours is equal to the number feet calculated, or exceed once every six hours whichever is less. Unless the rate is otherwise specified in subsection (b) below. This system shall be designed to give the proper turnover rate based on the manufacturer's recommended maximum pressure flow of the filter in dirty media condition of the filter, immediately prior to cleaning the filter.

(b) Turnover rates for pools by type listed below:

Type	Turnover Rate
1. Activity Pools	2 hours
2. Continuous Water Channels	1 hours
3. Dual Use Swimming Pools (swimming pools with a water slide and/ or one other feature with an average depth exceeding 24 inches)	4 hours
4. Diving Pools	8 hours
5. Interactive Water Play Pools/Spray Pads	30 minutes
6. Landing Pools, (Flumes, Slides and All Other Plunge Pools	60 minutes
7. Leisure Rivers	2 hours
8. Spas/Exercise Spas	30 minutes
9. Wading Interactive Pools (maximum depth, 18 inches)	60 minutes
10. Wading Pools (without any interactive equipment)	60 minutes
11. Water Attraction/Equipment Pump Reservoir Tanks	30 minutes
12. Wave Pools	2 hours

(c) Timing devices will be allowed for the purpose of turning down the circulation system during times when a pool is not being used. Timing devices must be set to provide at least one complete turnover immediately prior to the pool reopening.

1. The system flowrate shall not be reduced more than 25% lower than the minimum design flowrate requirement and only reduced when the pool is unoccupied.

2. The system flowrate shall ensure the minimum water clarity required under the chapter is met before opening to the public.

3. The system shall be required to maintain required disinfectant and pH levels at all times.

(d) For spas, a minute timer that does not exceed 15 minutes shall be connected to the agitation system. The timer shall be located out of reach of a bather in the spa.

(e) Water clarity shall be maintained. When standing at the pool's edge at the deep end, the main drain suction outlet covers or a four inch by four inch square marker tile in contrasting color shall be clearly visible. When standing at a spa's edge, the deepest portion of the spa floor shall be visible when the water is still.

1. For pools over ten feet deep an eight inch by eight inch square marker tile in a contrasting color to the pool floor shall be visible at the deepest part of the pool.

2. This reference point shall be visible at all times from the edge of the deck.

(f) Circulation system components which require replacement or servicing shall be accessible for inspection, repair or replacement and shall be installed in accordance with the manufacturer's instructions.

(g) Where equipment sizing falls within the scope of NSF testing, materials and equipment used in the circulation system shall comply with the appropriate requirements of NSF Standard 50.

Rule .07(1)

(h) Equipment used for a public pool shall be properly supported to prevent damage from misalignment or settlement. The equipment shall be mounted so as to minimize the potential for the accumulation of debris and moisture, following manufacturer's instructions.

(2) **Water Velocity.** The water velocity in the pool or spa piping for discharge piping shall not exceed eight feet per second and for suction piping, shall not exceed six feet per second.

(a) Pool and spa piping shall be sized to permit the rated flows for filtering and cleaning without exceeding the maximum head of the pump.

(b) The pump shall be sized to deliver the required flow rate against the total system head involved.

(3) **Piping and Fittings.** The circulation system piping and fittings shall be nontoxic, shall be considered to be process piping, and shall be of material able to withstand operating pressures and operating conditions.

(a) Pool and spa piping subject to damage by freezing shall have a uniform slope in one direction equipped with valves for adequate drainage. Pool and spa piping shall be supported at sufficient intervals to prevent entrapment of air, water, or dirt. Provisions shall be made for expansion or contraction of pipes. All piping shall comply with NSF Standard 14 or other applicable standards.

(b) Equipment shall be designed and fabricated to drain the pool or spa water from the equipment, together with exposed face piping, by removal of drain plugs and manipulating valves or by other methods. Refer to manufacturer's recommendations for specific information on draining the system.

(4) **System Condition.** Gauges shall be provided as follows:

(a) A pressure or vacuum gauge or other means of indicating system condition shall be provided in the circulation system in an easily readable location.

(b) A flow meter measuring the rate of flow through the filter system with an appropriate range readable in gallons per minute (GPM) and accurate within ten percent actual flow shall be provided. The flow indicator shall be capable of measuring from one-half to at least one and one-half times the design flow rate. The gauge shall be located after the filtering equipment and in such location on the return line, so as to measure the total amount of water returning to the pool according to the manufacturer's installation specifications.

(5) **Water Clarity and Chemistry.** The circulation system shall be capable of maintaining water clarity and water chemistry requirements and shall operate twenty-four hours per day, except as otherwise provided in this Chapter.

(6) **Instructions.** Written operation and maintenance instructions shall be provided for the circulation system.

[Authority: O.C.G.A. Secs. 31-2A-6, 31-12-8, 31-45-10.]

511-3-5-.08 Filters.

(1) **Design.** Filters shall be designed and maintained so as to provide the water clarity noted in DPH Rule 511-3-5-.07(1) (e).

(a) Filters shall be listed per NSF Standard 50 with the specific maximum flow rates per surface area based on media used.

(b) The following filtration rates for the specific media shall be used in determining the filter area required for the circulation system:

Rule .08(1)(b)

1. High-rate granular media filters shall be designed to operate at no more than fifteen gallons per minute per square foot when a minimum bed depth of fifteen inches is provided per manufacturer. When a bed depth is less than fifteen inches, filters shall be designed to operate at no more than twelve gallons per minute per square foot.

2. The design filtration rate for surface-type cartridge filters shall not exceed three-tenths gallons per minute per square foot. One complete set of spare cartridges shall be maintained on site in a clean and dry condition.

3. The design filtration rate for pre-coat filters shall be based on the following types:

(i) Vacuum pre-coat filters shall not be more than either two gallons per minute per square foot, or two and one half gallons per minute per square foot when used with a continuous pre-coat media feed.

(ii) Pressure pre-coat filters shall not be more than two gallons per minute per square foot of effective filter surface area.

(iii) The filtration surface area shall be based on the outside surface area of the media with the manufacturer's recommended thickness of pre-coat media and consistent with their NSF Standard 50 listing and labeling.

(c) Filters shall be designed so that filtration surfaces can be inspected and serviced.

(d) Alternate types of filter media shall be listed and labeled to NSF Standard 50.

(2) **Internal Pressure.** On pressure-type filters, a means shall be provided to permit the release of internal pressure.

(a) Any filter incorporating an automatic internal air release as its principal means of air release shall have lids which provide a slow and safe release of pressure as a part of its design.

(b) Any separation tank used in conjunction with any filter tank shall have a manual means of air release or a lid which provides a slow and safe release of pressure as it is opened as a part of its design.

(c) Influent and effluent pressure gauges (if both are present in the system) shall have the capability to measure up to twenty pounds per square inch increase in the differential pressure across the filter bed in increments of one pound per square inch or less.

(3) **Instructions.** Pressure filters and separation tanks shall have operation and maintenance instructions permanently installed on the filter or separation tank and shall include a precautionary warning statement not to start up the system after maintenance without first opening the air release and properly reassembling the filter and separation tank. The statement shall be visible and noticeable within the area of the air release.

(4) **Piping.** Piping furnished with the filter shall be of suitable material capable of withstanding one and one-half times the working pressure. The suction piping shall not collapse when there is a complete shutoff of flow on the suction side of the pump.

(5) A sight glass shall be installed on the waste discharge line of pressure filters so that the progress of filter washing can be observed.

(6) All piping shall be marked with directional arrows as necessary to determine flow direction. All piping in the equipment room shall be permanently identified by its use and the pool and or aquatic feature it serves.

[Authority: O.C.G.A. Secs. 31-2A-6, 31-12-8, 31-45-10.]

511-3-5-.09 Pumps and Motors.

(1) **Sizing.** A pump and motor shall be provided for circulation of the pool and spa water. Performance of all pumps shall meet or exceed the conditions of flow required for filtering and cleaning (if applicable) the filters against the total dynamic head developed

Rule .09(1)

by the complete system. Where applicable pumps shall comply with the NSF International Standard 50 or Underwriters Laboratories (UL) Standard 1081.

(2) **Strainer or Screen.** With all pressure filter systems, a cleanable strainer or screen shall be provided upstream of the circulation pumps to remove solids and debris such as hair and lint.

(3) Pumps and motors shall be accessible for inspection and service.

(4) **Safe Operation.** The design and construction of the pumps and component parts shall provide for safe operation.

(5) **Pump Seal.** Where a mechanical pump seal is provided, components of the seal shall be corrosion-resisting and capable of operating under conditions normally encountered in pool operation.

(6) **Capability.** Motors shall be capable of operating the pump under full load with a voltage variation of plus or minus ten percent from the nameplate rating. If the maximum service factor of the motor is exceeded (at full voltage), the manufacturer shall indicate this on the pump curve.

(7) **Overload Protection.** All motors shall have thermal or current overload protection, either built in or in the line starter, to provide locked rotor and running protection.

(8) If the pump is below the waterline, valves shall be installed on permanently connected suction and discharge lines, located in an accessible place outside the walls of the pool, where they shall be readily and easily accessible for maintenance and removal of the pump.

(9) Pressure or vacuum gauges shall be installed on all public pools and spas.

(a) The pump vacuum gauge shall be installed as close to the suction side of the pump as possible while still maintaining an accurate reading.

Rule .09(9)

(b) The pressure gauge shall be installed downstream from the pump, on the face piping ahead of the filter or on top of the filter in the area of greatest filter pressure.

[Authority: O.C.G.A. Secs. 31-2A-6, 31-12-8, 31-45-10.]

511-3-5-.10 Return Inlets and Suction Outlets.

(1) **Location.** Return inlets shall be installed and arranged to produce a uniform circulation of water and maintain a uniform disinfectant residual throughout the pool or spa. Where skimmers are used, the return inlets shall be located so as to help bring floating particles within range of the skimmers.

(a) Pools shall use wall or floor inlets that are adequate in design, number and location to provide adequate mixing.

(b) A swimming pool shall have a minimum of two return inlets regardless of pool size.

(c). The total number of return inlets shall be based on one inlet per three hundred square feet of pool surface area or one inlet for every twenty feet of pool perimeter or fraction thereof, whichever is greater. The return inlets placement shall be as follows:

1. Wall inlets shall be placed within five feet of each pool corner and at least five feet from a skimmer.

(i) Wall return inlets for the circulation system shall be designed to include directionally adjustable fitting to provide effective distribution of water.

(ii) The fitting shall not constitute a hazard to the user.

2. Floor return inlets must be used on pools more than fifty feet in width based on the following placement:

(i) Floor inlets shall be spaced to effectively distribute the treated water throughout the pool.

(ii) Distance between floor inlets shall be no more than twenty feet.

Rule .10(1)(c)2.

(iii) A row of floor inlets shall be located within fifteen feet of each side wall.

(iv) Floor inlets shall be flush with the bottom of the pool.

(v) Floor inlets used in combination with wall inlets shall be spaced no more than twenty-five feet from the nearest side walls.

3. For an aquatic facility with multiple pool types in combination using the same body of water, inlets shall meet the chapter's placement criteria and be hydraulically sized to provide the required turnover rate for each pool type.

4. Inlets shall be placed in each recessed or isolated area of the pool.

5. Wall inlets shall not be required to provide directional flow if part of a manufactured gutter system in which the filtered return water conduit is contained within the gutter structure.

(2) Location. All pools shall be provided with at least two main drain suction outlets with sumps in the lowest point of the pool floor or other approved methods.

(a) The main drain system shall be designed at a minimum to handle recirculation flow of 100% of total design recirculation flow rate. The branch pipe from each main drain outlet shall be designed to carry 100% of the recirculation flow rate

(b) The spacing of the main drains shall be at least three feet apart, but not more than twenty on centers nor more than fifteen feet from each side wall.

(c) Three or more suction outlets are subject to the three feet spacing requirement measured from the centerline between the outermost suction outlets.

(3) All spas shall have a minimum of two suction outlets provided for each pump in the suction outlet system, separated by a minimum of three feet or located on two different planes; e.g., one on the bottom and one on the vertical wall, or one each on two separate vertical walls. These suction outlets shall be plumbed such that water is drawn through them simultaneously through a common line to the pump.

(4) Suction outlets shall be provided with a cover that has been tested and approved by a nationally recognized testing laboratory and shall comply with the current ANSI/APSP-16 , Suction Fittings For Use in Swimming Pools, Wading Pools, Spas, and Hot Tubs or a successor standard and the following:

(a) Where three or more main drain suction outlets are connected by branch piping, the flow through each branch pipe from each main drain suction outlet shall be calculated as follows:

1. Quantity (Q) of flow (gpm) maximum for each drain = total design flowrate (DFR)) divided by number of drains (N) minus one drain, or

2. Q(gpm per drain) = DFR/(N-1).

(b) The suction outlets shall be connected to a single main suction pipe by branch lines piped to provide hydraulic balance between the drains.

(c) The branch lines shall not be valved so as to be capable of operating independently.

(d) All covers/grates shall be in the same body of water.

(e) Each suction outlet cover shall be attached to a properly manufactured or field fabricated sump that meets ANSI/APSP 16 or successor standard.

(f) The maximum flow on the pump's curve shall be used to select the cover.

(g) Field fabricated suction outlets must be designed and certified by a registered professional engineer to comply with ANSI/APSP 16 or successor standard.

1. Field fabricated suction outlet covers or grates must provide sufficient area so that the maximum velocity of the water passing the grate will not exceed one and one-half feet per second.

2. The field fabricated sumps shall be built so that the opening of the suction pipe will be no closer than one and one-half times the inside pipe diameter from the bottom of the

Rule .10(4)(g)2.

listed suction outlet cover/grate or in accordance with the standard or manufacturer instructions.

3. The width of openings in grating shall be not less than on eight inch and not more than one half inch. The pool or spa shall not be operated if the outlet grate is missing, broken or secured in such a way that it can be removed without the use of a tool.

(5) **Entrapment Avoidance.** If the suction outlet system, such as a filtration system, booster system, automatic cleaning system, or solar system, has a single suction outlet or multiple suction outlets which can be isolated, each suction outlet shall protect against user entrapment by installing a cover/grate that complies with ANSIAPSP- 7 Standard for Suction Entrapment Avoidance in Swimming Pools, Wading Pools, Spas, Hot Tubs, and Catch Basins or successor standard and as many of the following as necessary:

(a) A safety vacuum release system that has been tested by a nationally recognized independent third party and found to conform to ANSI/ASME standard A112.19.17 or ASTM standard F2387 and installed in accordance with manufacturers' instructions.

(b) A suction-limiting vent system designed by a professional engineer,

(c) A gravity drainage system designed by a professional engineer,

(d) Automatic pump shut off system that has been tested by a nationally recognized independent third party and found to conform to a recognized standard,

(e) Other means determined to be equally effective by the Department meeting the requirements of an applicable ASME/ANSI, ASTM or a Consumer Product Safety Commission standard.

(6) Where provided, the vacuum cleaner fittings shall be located in an accessible position between six and eighteen inches below the minimum operating water level or as an attachment to the skimmer(s).

Rule .10

(7) The vacuum line shall be protected with a self-closing, self-latching fitting that complies with the current IAMPO SPS 4- Special Use Suction Fitting for Swimming Pools, Spas and Hot Tubs.

[Authority: O.C.G.A. Secs. 31-2A-6, 31-12-8, 31-45-10.]

511-3-5-.11 Surface Skimmer Systems.

(1) A surface skimming system shall be provided on all swimming pools and spas and shall be designed and constructed to skim the pool or spa surface when the water level is maintained within the operational parameters of the system's rim or weir device. Surface skimming devices shall comply with NSF Standard 50.

(2) Skimming devices shall be designed and installed so as not to constitute a hazard to the user.

(3) **Automatic Surface Skimmers.** Where automatic surface skimmers are used as the sole overflow system in pools, at least two surface skimmers shall be provided for the first four hundred square feet or fraction thereof of the water surface area and one skimmer shall be provided for each additional four hundred square feet of surface area. In spas, one skimmer shall be provided for each one hundred square feet of surface area.

(a) Nominal recessed areas such as stairs and swimouts, shall not be considered in the calculation.

(b) When skimmers are used, they shall be located to maintain effective skimming action over the entire surface of the pool or spa.

(c) The skimmer flow rate shall not be less than twenty- five gallons per minutes or more than fifty-five gallons per minutes unless they are based on the manufacturer’s design

specifications. The flow rate for the skimmers shall comply with manufacturer data plates or NSF/ANSI 50 including Annex K.

(d) Each skimmer shall have a weir that adjusts automatically to variations in water level over a minimum range of four inches.

(e) Each skimmer shall be equipped with a trimmer valve capable of distributing the total flow between individual skimmers.

(f) The skimmer equalizer lines, when used, shall be located on the wall with the center no more than eighteen inches below the maximum operating level.

(g) The skimmer equalizer lines shall be protected by an approved cover/grated with a flow rating equal the maximum system flow divided by the number of skimmers in the system or the maximum flow rating of the skimmer, whichever is greater.

(h) Additional skimmers may be required to achieve effective skimming under site-specific conditions.

(i) The base of each skimmer shall be level with all other skimmers in the pool within a tolerance of plus or minus one-half inch.

(4) **Perimeter Surface Skimmer (Gutter).** Where a perimeter type surface skimming system is used as the sole surface skimming system, this system shall extend completely around the perimeter of the pool except at steps or recessed ladders.

(a) The lip of the gutter shall be level and shall be designed to serve as a handhold for bathers.

(b) The perimeter surface skimming system shall be connected to the circulation system with a system surge capacity of not less than one gallon for each square foot of pool surface or two and one half gallons for each square foot of spa surface.

(c) The hydraulic capacity of the overflow system shall be capable of handling one hundred percent of the circulation flow.

Rule .11(4)

(d) Gutters shall be level within a tolerance of plus or minus one-sixteenth inch around the perimeter of the pool.

(e) Automatic makeup water supply equipment shall be provided to maintain continuous skimming of pools with perimeter overflow systems.

(f) Makeup water shall be supplied through an air gap or other approved backflow prevention device.

[Authority: O.C.G.A. Secs. 31-2A-6, 31-12-8, 31-45-10.]

511-3-5-.12 Lighting and Electrical Requirements.

(1) **Artificial Lighting.** Artificial lighting shall be provided for all indoor and outdoor pools and spas. Lighting shall be adequate to illuminate the entire swimming pool enclosure without glare. All installations shall comply with local building code requirements. Ground-fault interrupters must be provided. Lighting in dressing rooms, sanitary facilities, equipment rooms and concessions shall comply with local code requirements.

(a) **Water Surface and Deck Area Illumination.** The water surface and deck area light levels shall meet the following minimum illumination levels:

1. An indoor pool water surface and deck: thirty horizontal foot-candles.

2. An outdoor pool deck: ten horizontal foot-candles.

3. An outdoor pool water surface with underwater lighting: ten horizontal foot-candles.

4. An outdoor pool water surface without underwater lighting: fifteen horizontal foot-candles.

(b) Underwater lighting is not required. However, if underwater lighting is used, then the following shall apply:

Rule .12(1)(b)

1. A minimum of eight lumens per square foot of water surface area must be provided in conjunction with overhead or equivalent deck lighting.

2. Underwater lights, in conjunction with overhead or equivalent deck lighting, shall be located to provide the required illumination so that all portions of the pool, including the pool bottom and main drain suction outlets, may be readily seen.

3. Dimmable or color lighting shall not be used for underwater lighting.

(c) Illumination shall render all portions of the pool, including the pool bottom and main drain suction outlets, readily visible.

(d) More light may be required as deemed necessary by the Health Authority or by other codes which apply.

(e) The lighting shall be evenly spaced around the pool to prevent glare.

(f) Higher underwater light levels shall be considered for deeper water to achieve the outcome. This must be approved by the professional engineer or architect.

(2) For outdoor pools, when not being used for night swimming or recreation, a minimum of three foot candles shall be maintained at the surface of pool and deck areas. Motion detector type lights are acceptable.

(3) No switches, starters, panel boards or similar electrical equipment shall be located in areas readily accessible to bathers while in the pool or on the designated deck area.

(4) No overhead wiring shall pass within twenty feet (horizontal distance) of the pool enclosure.

(5) No electrical outlets shall be located within ten feet of the pool edge.

(6) Public swimming pools that operate outside of daylight hours shall be provided with sufficient emergency lighting to permit evacuation of the pool and securing of the area in the event of power failure. The emergency lighting intensity shall be not less than one foot candle at the water surface and the walking surface of the deck.

(7) Color lighting is prohibited for use as pool deck and water surface illumination.

[Authority: O.C.G.A. Secs. 31-2A-6, 31-12-8, 31-45-10.]

511-3-5-.13 Heaters and Temperature Requirements.

(1) **Sizing.** Heaters, when used, shall be properly sized according to the volume of water, square footage of surface area, and manufacturer's recommendations.

(2) **Water Temperature.** The owner/operator shall routinely check the in-pool or in-spa water to ensure that the temperature does not exceed 104^{0}F.

(a) If adjustments are necessary, those adjustments shall be performed in accordance with manufacturer's instructions or by a qualified technician.

(b) An annual gas fired inspections shall be performed by a qualified professional.

(c) A thermometer shall be available to measure the temperature of the water. It shall be attached or available to the operator at all times.

(3) **Installation.** The heater shall be installed in accordance with state and local codes as well as the manufacturer's recommendations.

(a) **Support.** The heater shall be installed on a surface with sufficient structural strength to support the heater when it is full of water and operating. The heater shall be level and not able to move after plumbing, gas, and electrical connections are completed.

(b) **Ventilation.** Fossil fuel heaters shall have adequate ventilation in order to assure proper operation.

(c) **Make Up Air.** Make up air shall be sufficient for proper operation.

[Authority: O.C.G.A. Secs. 31-2A-6, 31-12-8, 31-45-10.]

511-3-5-.14 Air Blower and Air Induction Systems.

(1) **Entry Devices.** This rule pertains to all devices and systems which induce or allow air to enter the spa either by means of a power pump or passive design.

(2) **Air Intake Source.** Air intake sources shall not induce water external to the spa unit, dirt, or contaminants into the spa.

(3) **Make Up Air.** An air blower installed within an enclosure or indoors shall have adequate ventilation. The air induction system shall be installed in accordance with any applicable codes and the manufacturer's recommendation for air openings to the enclosure.

(4) **Accessibility.** The air blower shall be accessible for inspection and service.

(5) **Air Passages.** Integral air passages shall be pressure tested at the time of manufacture to provide structural integrity to a value of one and one-half times the intended working pressure.

[Authority: O.C.G.A. Secs. 31-2A-6, 31-12-8, 31-45-10.]

511-3-5-.15 Water Supply and Wastewater Disposal.

(1) The water supply for public pools and spas, showers, lavatories, drinking fountains and any other uses in conjunction with the public pool shall be from an approved and potable source and shall be approved by the local health authority before use. Water in the pool shall meet the requirements of DPH Rule 511-3-5-.17 before the pool may be used by bathers.

(2) No direct mechanical connection shall be made between the potable water supply and the swimming pool, chlorinating equipment, or the system of piping for the pool, unless it is protected against backflow and back-siphonage through an air gap meeting the latest ANSI/ASME standard A112.1.2 and the International Plumbing Code or other equivalent means approved by the health authority.

(3) An over-the-rim spout, if used, shall be located under a diving board, adjacent to a ladder or otherwise properly shielded so as not to create a hazard. Its open end shall have no sharp edges and shall not protrude more than two inches beyond the edge of the pool. The open end shall be separated from the water by an air gap of at least one and one-half pipe diameters measured from the pipe outlet to the rim.

(4) Backwash water may be discharged into a sanitary sewer through an approved air gap or into an approved subsurface disposal system or by other means approved by the health authority.

(5) Backwash water shall not be returned to the public swimming pool, equipment reservoir or surge tank. Use of backwash water for other purposes must meet state or local law or ordinances.

(6) Where necessary, filter backwash water shall be diverted to a settling tank to eliminate diatomaceous earth and contaminants in the water that exceed the limits set by the state or local water authority.

(7) If required by the water authority, pool water may require neutralizing before being completely drained into a sanitary sewer.

[Authority: O.C.G.A. Secs. 31-2A-6, 31-12-8, 31-45-10.]

511-3-5-.16 Disinfectant Equipment and Chemical Feeders.

(1) Disinfectant equipment and chemical feeders, such as flow-through chemical feeders, electrolytic chemical generators, mechanical chemical feeders, chemical feed pumps, and automated controllers shall comply with the requirements of NSF Standard 50.

(2) The disinfection equipment shall be capable of precisely delivering a sufficient quantity of a registered disinfecting agent in the appropriate amount as outlined in (3) in

Rule .16(2)

this section and maintain the residual concentrations in DPH Rule 511-3-5-.17 of this Chapter.

(a) Every pool and spa shall be required to have at least one unit of disinfectant agent equipment that introduces the agent through the circulation system in compliance with this rule

1. Additional units may be required to maintain chemical and physical parameters of the pool water for new construction or an existing facility, if deemed necessary by the health authority or as required in DPH Rule 511-3-5-.16(2)(3).

2. Increased risk public pools constructed or remodeled after the adoption of this chapter shall deliver, monitor and control disinfectant and pH chemical feeders through an automated chemical controller.

3. Increased risk public pools constructed after the adoption of this chapter shall be required to use an NSF Standard 50 approved supplemental disinfection treatment system such as ozone or ultraviolent light (UV).

(b) The pool or spa water shall be continuously disinfected by a disinfecting agent that imparts an easily measured residual. The disinfecting agent used shall be subject to field testing procedures that are simple and accurate.

(c) Gaseous chlorine, chlorine compounds, bromine compounds or other bactericidal agents shall be acceptable when meeting the disinfectant level parameters outlined in DPH Rule 511-3-5-.17 of this Chapter. Other disinfectant agents not outlined in DPH Rule 511-3-5-.17 may be used if,

1. The owner/operator provides test results to the health authority that show the agent to be an adequate disinfectant for swimming pool and spa use, and

2. A test kit for these other agents is supplied to the health authority by the manufacturer or the pool owner.

(d) All disinfectant agents shall be registered by the U.S. Environmental Protection Agency.

(e) Where water is drawn from the pool to supply water to aquatic features the water may be reused prior to filtration if;

1. The disinfectant and pH levels of the supply water are maintained at required levels and the ratio of interactive play feature, slide, or other apparatus unfiltered water to filtered water circulated in the reservoir or pool shall be no more than 3:1 in order to maintain the efficiency of the filtration system, or

2. The apparatus or device shall use only water that has been filtered and disinfected immediately prior to being discharged into the pool. This includes, but is not limited to, slides, fountains, water wheels, “mushrooms”, and squirt guns.

(f) Any water discharged into the pool water shall at least the same level of disinfection that is required for the type of pool that the device is in as listed in DPH Rule 511-3-5-.17.

(3) **Chemical Feeders.** The installation and use of chemical feeders shall conform to the following standards:

(a) Chemical feeders must be installed downstream from the filter and heater.

(b) If the chemical feeder is equipped with its own pump, it shall be installed so it introduces the gas or solution downstream from the heater and, if possible, at a position lower than the heater outlet fitting.

(c) Chemical feed pumps and controllers shall be wired so they cannot operate unless the filter pump is running. If the chlorinator has an independent timer, the filter and chemical feed pump timers shall be interlocked.

(d) All chlorine dosing and generating equipment including erosion feeders, or in line electrolytic and brine/batch generators, shall be designed with the capacity to provide an

Rule .16(3)(d)

adequate dose of disinfectant based on the class, use, load, and setting. The system shall be designed with a capacity to provide the following:

1. Outdoor pools design capacity shall be four pounds of free available chlorine/day/10,000 gallons of pool water;

2. Indoor pools design capacity shall be two and one-half pounds free available chlorine/day/10,000 gallons of pool water.

(e) The rates above are minimums and in all cases the professional engineer or manufacturer shall validate the feed and production equipment specified. Stabilized levels must be able the meet the chapter.

(f) A physical barrier shall be installed between chemical feed pumps supplying acid or liquid hypochlorite solution and other pool components to shield staff and equipment from chemical sprays which might result from leaking connections.

(g) Feeders shall be capable of supplying disinfectant and pH control chemicals, if applicable, to maintain the minimum required disinfection levels at all times in accordance with the chapter.

(h) The injection point of disinfection chemicals shall be located before any pH control chemical injection point with sufficient physical separation of the injection points to reduce the likelihood of mixing of these chemicals in the piping during periods of interruption of recirculation system flow.

(i) The professional engineer shall validate the feed and production equipment specified. Disinfectant levels must meet the requirements of the chapter.

(j) In-line generators shall be permitted on pools using the following requirements:

1. In-line generators shall use pool-grade salt dosed into the water to produce and introduce chlorine into the pool treatment loop through an electrolytic chamber.

Rule .16(3)(j)

2. Electrolytic generators shall have a total dissolved solid (TDS) or salt (NaCl) readout and a low salt indicator.

3. The feed rate shall be adjustable from zero to full range.

4. The generator unit shall be listed and labeled to NSF Standard 50 and UL 1081 for electrical/fire/shock safety by an ANSI-accredited certification organization.

5. The generator shall be interlocked and installed according to the manufacturer's instructions.

6. The saline content of the pool water shall be maintained in the required range.

7. Brine batch generators shall produce chlorine through an electrolytic cell and produces chlorine from brines composed of pool-grade salt.

8. In line generator equipment shall have an EPA facility registration number.

9. An in line generator maybe supplemented with other systems to meet the dosing requirements in subsection (3) (d) above.

(k) Feeders for pH adjustment shall comply with the following:

1. Chemicals for pH adjustment shall include but not be limited to muriatic (hydrochloric) acid, sodium bisulfate, carbon dioxide, sulfuric acid, sodium bicarbonate, and soda ash.

2. A pH adjustment feeders shall be adjustable from zero to full range.

3. Reservoirs shall be clearly marked and labeled with contents.

(l) Automated controllers shall be installed for monitoring and turning on or off chemical feeders used for pH and disinfectant control in facilities referenced in DPH Rule 511-3-5-.16(2)(a)2.

(m) Operation manuals or other instructions that give clear directions for cleaning and calibrating automated controller probes and sensors shall be provided for the automated controller.

Rule .16(3)

(n) Where used, ultraviolet light (UV) systems shall be installed in the recirculation system after the filters;

1. A bypass pipe that is valved on both ends shall be installed to allow maintenance on the UV unit while the pool is in operation.

2. UV system operation shall be interlocked with the recirculation pump so that power to the UV system is interrupted when there is no water flow to the UV unit.

(4) **Gas Feed Systems.** Carbon dioxide and ozone are the only gas feed systems permitted at a new public pool. Where CO_2 cylinders are located indoors, a monitor and alarm shall be provided to alert patrons/operator of high CO_2 or low O_2 levels**.**

(5) **Elemental (Gaseous) Chlorine**. Chlorine in the gaseous form may not be used as a disinfectant in pools constructed after 31 December 2016. Facilities that currently use gas chlorine systems may continue to use them if they follow subsections (a)-(p) below.

(a) Users of gas chlorine must be trained on the proper procedures for handling chlorine and the appropriate emergency procedures.

(b) Gas chlorination equipment shall be located so that equipment failure or malfunction will have minimal effect on evacuation of pool patrons in an emergency.

(c) Gas chlorine feeders (chlorinators) shall be activated by a booster pump using recirculated water supplies via the recirculation system. The booster pump shall be interlocked to the filter pump to prevent feeding of chlorine when the recirculation pump is not running.

(d) The chlorinator, cylinders of chlorine, and associated equipment shall be housed in a reasonably gas-tight and corrosion-resisting housing having a floor area adequate for the purpose. Cylinders shall always be stored in an upright position and properly secured so they cannot tip over if bumped.

Rule .16(5)

(e) All enclosures shall be located at or above ground level. The enclosure shall be provided with a motor-driven exhaust fan capable of producing at least one air change per minute. This fan must be located at the lower part of the enclosure and there must be louvers of good design near the top of the enclosure for admitting fresh air. A warning sign stating “Chlorine Gas” shall be posted on doors. Doors to the chlorine room shall open away from the pool and be equipped with a viewing window located so that the chlorinator and the inside of the enclosure can be clearly seen prior to entering.

(f) Electrical switches for the control of artificial lighting and ventilation systems shall be on the outside of the enclosure adjacent to the door.

(g) Facilities shall include a scale suitable for weighing the cylinders.

(h) Connections from the cylinders to the system depend on the type of chlorinator to be used and shall comply with the chlorinator manufacturer's recommendation.

(i) An automatic chlorine leak detector and alarm shall be installed in the chlorinator room.

(j) Respirators approved by the National Institute for Occupational Safety and Health (NIOSH) shall be provided for protection against chlorine. Occupational Safety and Health Administration (OSHA) regulations require training and maintenance programs for respirators.

(k) Containers may be stored indoors or outdoors. Full and empty cylinders shall be segregated and appropriately tagged. Storage conditions shall:

1. minimize external corrosion;
2. be clean and free of trash;
3. be located away from an elevator or ventilation system; and
4. be located away from elevated temperatures or heat sources.

Rule .16(5)

(l) A specific person shall be made responsible for chlorination operations and shall be trained in the performance of routine operations including emergency procedures and leak control procedures, and maintain current documentation of their training in proper respirator use.

(m) Chlorine cylinders must be handled with care. Valve protection caps and valve outlet caps shall be in place at all times except when the cylinder is connected for use. Cylinders must not be dropped and shall be protected from falling objects. Cylinders shall be used on a first-in, first-out basis. New, approved washers shall be used each time a cylinder is connected.

(n) A safety wall chart shall be posted in or near the chlorine enclosure and a second chart shall be posted in the pool office near the telephone. The telephone number of the chlorine supplier shall be shown on this chart.

(o) Pool personnel shall be informed about leak control procedures.

(p) As soon as a container is empty, the valve shall be closed and the lines disconnected. The outlet cap shall be applied promptly and the valve protection hood attached. The open end of the disconnected line shall be plugged or capped promptly to keep atmospheric moisture out of the system.

(6) **Training.** Personnel responsible for the operation of the disinfection agent equipment shall be properly trained in the operation of that equipment and the procedure for performing and interpreting the necessary chemical field tests and the appropriate emergency procedures.

(7) **Test Kits.** Every pool shall be supplied with an accurate and reliable water quality testing device capable of measuring any agent that is introduced into the water of the pool.

Rule .16(7)

(a) Digital water quality testing devices shall be listed and labeled to NSF 50 or approved by the health authority.

(b) All test kits should include methods for the determination of pH, free available chlorine (FAC), total available chlorine (TAC) if chlorine is used, bromine or other chemical disinfectant residuals, cyanuric acid (if used), total alkalinity, calcium hardness, and copper and silver (if a copper or copper/silver ionization unit has been installed).

(c) The local health authority shall be given, upon request, a field testing kit for any agents introduced into the water supply. If a field testing kit is not available, the agent cannot be introduced until standards for testing have been established by, and written approval has been obtained from, the health authority.

(d) The Orthotolidine test (OTO) is unacceptable since it cannot distinguish FAC and TAC.

(e) The test kit shall be stored in accordance with manufacturer's instructions. Chemical agents shall be maintained at proper manufacturer specified temperatures.

(f) A test kit that requires calibration shall be calibrated in accordance with the manufacturer's instructions.

[Authority: O.C.G.A. Secs. 31-2A-6, 31-12-8, 31-45-10.]

511-3-5-.17 Chemical Operational Parameters.

The chemical operational parameters in swimming pool or spa water shall not exceed the maximum level or be lower than the minimum level given in the following parameters. Where no minimum or maximum is given, additional information is within this Chapter to assist the pool operator.

Rule.17

	Minimum	Ideal	Maximum	Comments
(**1) Disinfectant Levels**<br><br>(a) Free chlorine, ppm in pools not using cyanuric acid or a stabilized chlorine compound use (b),1.-5.				
(b) All public pools except as listed below:	1.0	1.0-3.0	10.0	In a pool, hot weather/heavy use may require operation at or near maximum levels. Regular superchlorination is recommended<br><br>(see Remedial Practices below).
1**.** Spas	3.0	3.0-5.0	10.0	
2.Activity/interactive/ Wading Pools	2.0	2.0-5.0	10.0	
3. Interactive Water Play Pool(Spray Pad)	2.0	2.0-5.0	10.0	
4. Wading Pools	2.0	2.0-5.0	10.0	
5. Water Attraction Pump Reservoirs	2.0	2.0-5.0	10.0	
(c) Free Chlorine level in pools using cyanuric acid or a stabilized chlorine product	2.0	2.0-5.0	10.0	
Combined chlorine, ppm	None	None	0.4	High combined chlorine results in chloramine formation and reduced chemical efficacy. Take remedial action to establish break point chlorination (See Remedial Practices below). Other signs of combined chlorine: -Sharp chlorine odor -Eye irritation -Algae growth

	Minimum	Ideal	Maximum	Comments
Bromine, ppm	Pool 3.0<br><br>Spa 4.0	Pool 3.0-5.0<br>Spa 4.0-6.0	Pool 8.0<br>Spa 8.0	
**(2) Chemical Values**				
pH	7.2	7.4-7.6	7.8	**If pH is:**<br>Too High:<br>– Low chlorine efficiency<br>– Scale formation Cloudy Water<br>Too Low:<br>– Rapid dissipation of disinfectant<br>– Eye discomfort<br>– Plaster and concrete etching<br>– Corrosion of metals and vinyl liner damage
Alkalinity (buffering), ppm as $CaC0_3$	60 ppm	80-100 for halogen compounds with a high pH 100-120 for halogen compounds with a low pH	180	**If total alkalinity:**<br>Too Low:<br>– pH bounce<br>– corrosion tendency<br>Too High:<br>– Cloudy water<br>– Increased scaling potential<br>– pH tends to be too high

	Minimum	Ideal	Maximum	Comments
Total dissolved solids (TDS), ppm				These values are offered as guidelines rather than absolute values to indicate concern for accumulation of impurities in the course of operation. Excessive high TDS may lead to hazy water or corrosion of fixtures, and can be reduced by partial draining with addition of fresh water.<br><br>High initial TDS may indicate poor water quality due to corrosive mineral salts, humus or organic matter. Consult local water authority.<br><br>Increasing TDS indicates build-up of impurities to be controlled by partial drain/refill with fresh water.
Calcium hardness, ppm, as $CaCo_3$	150	200-400 to balance water	1000	Operations of pools, spas and hot tubs at maximum hardness will depend on alkalinity (buffering) requirements of the disinfectant used. Maximum alkalinity and lower pH must be used with maximum hardness (over 500 ppm)

**(2) Chemical Values (con't)**				
Heavy metals, ppm	None	None		If heavy metals, such as copper, iron, manganese, silver are present:<br>-Staining may occur<br>-Water may discolor<br>-Chlorine dissipates rapidly<br>-Filter may plug<br>-May indicate pH too low or corrosion.
**(3) Biological Values**				
Algae	None	None	None	If algae are observed:<br>-Shock treat pool (See Remedial Practices, Shock treatment)<br>-Supplement with brushing and vacuuming.<br>-Use approved algaecide according to label directions (See Remedial Practices below)

	Minimum	Ideal	Maximum	Comments
Bacteria	None	Recognized Water quality Standard		If bacteria count exceeds maximum allowed:<br>-Superchlorinate and follow proper maintenance procedures<br>-Maintain proper disinfectant residual.
**(4) Stabilizer (if used)**				
Cyanuric acid, ppm	-	30-50	90	**If stabilizer is:**<br>Too High:<br>– May reduce chlorine efficacy<br>Too Low:<br>– Chlorine Residual rapidly destroyed by sunlight<br><br>Note: Stabilizer is not needed in indoor or brominated pools and spas.

	Minimum	Ideal	Maximum	Comments
**(5) Remedial Practices**				
Break point chlorination dosage in ppm.<br><br>Superchlorination				When combined chlorine is over 0.4 ppm, superchlorinate by adding ten times the combined chlorine in ppm and subtract the current disinfectant level. (e.g. combined chlorine is 0.5 ppm, superchlorinate by adding 4 ppm. (5 ppm -1 ppm current chlorine level = 4 ppm)<br><br>Applied at the end of daily usage, hold this level for 1-4 hours to clarify the water, remove ammonia (combined chlorine), and to kill any algae present.<br><br>Can also be applied when no bathers are present and as required to maintain clear water and the required halogen residual.
Superchlorination frequency	Pool- monthly	Pool- Every other week<br><br>Spa- Daily	Pool- Weekly when the temperature is over 85 ^{0}F	Note: Some high use pools may need superchlorination three times a week or more as a preventative measure or when combined chlorine is over 0.4

	Minimum	Ideal	Maximum	Comments
Shock treatment, dosage in ppm	10.0	--------	---------	Nonchlorine oxidizers are not considered biocidal, but may reduce organic contaminants.
Clarifying/Flocculating frequency	------	When needed	---------	Use all clarifiers following manufacturer's directions.
Algaecides				Follow manufacturer's directions. Use E.P.A. registered products.
Water replacement				Water in spas that have high bather use may require partial or complete replacement of water periodically to dilute dissolved solids, to maintain water clarity and to do necessary routine maintenance.

	Minimum	Ideal	Maximum	Comments
Foam	None	None	None	Foam may harbor persistent microorganisms. If foaming is not adequately controlled, consider daily shock treatment, water replacement or an appropriate antifoam agent. Follow manufacturer's directions.
**(6) Temperature** ^{0}F	--	78 -82 ^{0}F or Bather preference	104 ^{0}F	**If temperature is:**<br>Too High:<br><br>– Health hazard<br>– Bather discomfort<br>– Excessive fuel requirement<br>– Increased evaporation<br>– Increased scaling potential<br>– Increased use of disinfectants<br>– Increase potential for corrosion<br><br>Too Low:<br><br>– Bather discomfort<br>– Increase chance of hyperthermia

	Minimum	Ideal	Maximum	Comments
**(7) Water Clarity**				
Water turbidity	Must be able to see main drain covers or marker tile on the bottom of the deepest portion of the pool.	--	--	**If water is turbid:**<br>– Disinfectant level may be low<br>– Filtration system may be inoperative<br>– Improper chemical balance<br>– Bottom should be clearly visible at the deepest part of the pool or spa.<br>– Consult remedial practices

	Minimum	Ideal	Maximum	Comments
**(8) Oxidizers**				
Ozone, low output generators<br><br>Contact concentration mg/L when ozone is injected and not removed prior to entry into pool.<br><br>Above pool and spa levels	--<br><br>0	--<br><br>0	0.1<br><br>0.05	Serves as oxidizer of water contaminants.<br><br>Indoor installations should have adequate ventilation.
**(9) Oxidizer Reduction Potential**				
ORP	750 MV	--	--	When chlorine or bromine is used as the primary disinfectant, ORP can be used as a supplemental measurement of proper disinfectant activity. The use of ORP testing does not eliminate or supersede the need for testing the disinfectant level with standard test kits and ORP reading may be affected by a number of factors including (1) pH, (2) probe film and (3) cyanuric acid. Follow manufacturer's recommendations.

[Authority: O.C.G.A. Secs. 31-2A-6, 31-12-8, 31-45-10.]

511-3-5-.18 Specific Safety Features and Markers.

(1) **Handholds.** A public pool shall have a suitable handhold around its perimeter in areas where the depth exceeds three feet six inches. Handholds shall be provided no more than four feet apart and shall consist of any one or a combination of the items listed below:

(a) Coping, ledge or deck along the immediate top edge of the pool which provides a slip-resisting surface of at least four inches minimum horizontal width and located at or not more than twelve inches above the waterline; or

(b) Ladders, stairs or seat ledges; or

(c) A railing placed at or not more than twelve inches above the waterline.

(2) **Rope and Float Line.** A rope and float line shall be provided within one foot of and on the shallow side of the break in grade between the shallow and deep portions of the swimming pool, with its position marked with visible floats at intervals of seven feet or less.

(a) The rope and float line shall be securely fastened to wall anchors of corrosion-resisting materials and of the type which shall be recessed or have no projection that will constitute a hazard when the line is removed.

(b) The line shall be of sufficient size and strength to offer a good handhold and support loads normally imposed by users.

(c) The operator may remove the float line when the pool is used for lap swimming or swim meets. The line must be reattached immediately after completion of the event.

(3) **Depth Markers for Swimming Pools**. Depth of water in feet and inches shall be plainly and conspicuously marked at or above the waterline on the vertical pool wall and on the top of the coping or edge of the deck or walk next to the pool. The word or abbreviation for “feet” and “inches” must be specified. Where displayed in meters in addition to feet and inches, the word meter shall be spelled out.

Rule .18(3)

(a) Depth markers on the vertical pool wall shall be positioned to be read from the water side. The marker shall be placed to allow as much of the number to be visible above the waterline as possible.

(b) Depth markers on the deck shall be within eighteen inches of the water edge and positioned to be read while standing on the deck facing the water.

(c) Depth markers shall be slip-resistant.

(d) Depth markers shall be installed at the maximum and minimum water depths and at all points of slope change.

(e) Depth markers shall be installed at intermediate increments of water depth of two feet or less, and shall be spaced at intervals of twenty-five feet or less.

(f) Depth markers shall be arranged uniformly on both sides and both ends of the pool.

(g) Depth markers on irregularly shaped pools shall designate depths at all major deviations in shape.

(h) Depth markers number and letters shall be tile and four inches minimum in height. Numbers shall be of contrasting color to the background on which they are applied.

(i) Depth markers shall indicate the actual pool depth within plus or minus three inches, at normal operating level when measured three feet from the pool wall or at the tangent point where the cove radius meets the floor, whichever is deeper.

(j) Interactive water play pools shall not be required to have depth markings or “No Diving” signage.

(4) **Depth Markers for Spas.** Public spas shall have permanent depth markers with numbers and letters a minimum of four inches high plainly and conspicuously visible from all obvious points of entry and in conformance with subsections (a) thru (f) below:

(a) There shall be a minimum of two depth markers per spa, regardless of spa size or shape.

Rule .18(4)

(b) Depth markers shall be spaced no more than twenty-five feet apart and shall be uniformly located around the perimeter of the spa.

(c) Depth markers shall be positioned on the deck within eighteen inches of the water line.

(d) Depth markers shall be positioned to be read while standing on the deck facing the water.

(e) Depth markers in or on the deck surfaces shall be slip-resisting.

(5) **Clock.** All public facilities shall have a functioning clock which is visible to spa users.

(6) **Water Temperature.** The maximum temperature in a spa shall not exceed 104^0F (40^0C).

(a) The spa operator shall be provided with an accurate thermometer (±1^0F tolerance) and shall periodically check to ensure that the maximum temperature does not exceed 104^0F.

(b) A means to determine the spa temperature with a ±1^0F tolerance shall be provided to the user.

(7) **Water Agitation**. The agitation system on spas constructed after 31 December 2016 shall be connected to a minute timer that does not exceed fifteen minutes and shall be located out of reach of a bather in the spa.

(8) **Emergency shutoff switch.** A clearly labeled emergency shutoff switch shall be provided for all pools and spas constructed or remodeled after the adoption of this chapter. The emergency shutoff or control switch shall stop the motors that provide power to the circulation system and hydrotherapy or agitation system pump. The emergency shutoff switch installation shall be installed in accordance with the applicable electrical code.

(9) **Lifeguards.** All owners, managers, or lifeguards, if provided, shall be responsible for the supervision and safety of the pool, spa, or recreational water park. If lifeguards and safety assistants are provided, then they must hold current, nationally recognized certifications in lifeguarding and a designated title commensurate to the assigned duties.Adult/child/infant CPR and First Aid certifications also must be current. The certificates, or photocopies thereof, shall be maintained at the facility and be available to the local health authority for inspection.

(10) **Lifesaving Equipment.** All public swimming pools shall have lifesaving equipment conspicuously and conveniently on hand at all times. Lifesaving equipment for special purpose pools may be exempted from this requirement or the requirements will be provided as deemed necessary by Health Authority. The following will be provided:

(a) A light, strong pole not less than twelve feet long including body hook.

(b) A minimum one-fourth inch diameter throwing rope one and one-half times the maximum width of the pool or fifty feet in length, whichever is less, to which has been firmly attached a ring buoy with an outside diameter of approximately fifteen inches or a similar flotation device which is U.S. Coast Guard approved.

(c) An operable, hard-wired, conventional telephone line or continuous powered source, weatherproof emergency phone shall be permanently installed in a conspicuous location within the pool enclosure and must be readily available to bathers at all times. The emergency phone shall be capable of connecting to 911 and electronically transferring an automatic number identification and automatic locator identification of the pool emergency phone to the public safety answering point, if available. A 911 sign or the names and phone numbers of the nearest available police, fire, ambulance service or rescue unit shall be posted nearby.

Rule .18

(11) **Barriers.** All outdoor swimming pools and spas shall be enclosed by a barrier to prevent entry to the pool area when the pool is closed.

(a) The top of the barrier shall be at least forty eight inches above grade measured on the side of the barrier which faces away from the swimming pool.

1. The maximum vertical clearance between a solid surface and the bottom of the barrier shall be four inches measured on the side of the barrier which faces away from the swimming pool.

2. For non-solid surfaces, the vertical clearance between the bottom of the barrier and the grade shall not exceed two inches for a barrier constructed after adoption of the chapter.

3. Where the top of the pool structure is above grade, the barrier may be at ground level, such as the pool structure.

4. Where the barrier is mounted on top of the pool structure, the maximum vertical clearance between the top of the pool structure and the bottom of the barrier shall be four inches.

(b) Openings in the barrier shall not allow passage of a four inch diameter sphere.

(c) Solid barriers which do not have openings such as a masonry or stone wall, shall not contain indentations or protrusions except for normal construction tolerances and tooled masonry joints.

(d) Where the barrier is composed of horizontal and vertical members and the distance between the tops of the horizontal members is less than forty-five inches, the horizontal members shall be located on the swimming pool side of the fence. Spacing between vertical members shall not exceed one and three-fourth inches in width. Where there are decorative cutouts within vertical members, spacing within the cutouts shall not exceed one and three-fourth inches in width.

(e) Where the barrier is composed of horizontal and vertical members and the distance between the tops of the horizontal members is forty-five inches or more, spacing between vertical members shall not exceed four inches. Where there are decorative cutouts within vertical members, spacing within the cutouts shall not exceed one and three-fourth inches in width.

(f) Maximum size for chain link fences shall be one and three-fourth inches unless the fence is provided with slats fastened at the top or the bottom which reduce the openings to no more than one and three-fourth inches.

(g) Where the barrier is composed of diagonal members, such as a lattice fence, the maximum opening formed by the diagonal members shall be no more than one and three-fourth inches.

(h) Access gates shall also comply with the requirements of this Rule and shall be equipped to accommodate a locking device. Barrier gates shall open away from the pool in facilities constructed after adoption of this chapter. Pedestrian access gates shall be self-closing and have a self-latching device. Gates other than pedestrian access gates shall have a self-latching device. Where the release mechanism of the self-latching device is located less than fifty-four inches from the bottom of the gate,

1. The release mechanism shall be located on the pool side of the gate at least three inches below the top of the gate and

2. Any openings in the gate or barrier located within eighteen inches of the release mechanism shall be less than one-half inch.

3. Mechanisms controlled by an access card reader, key entry device or keypad shall be located on the outside of the access gate.

(i) There shall not be direct access from any dwelling into the pool enclosure. For indoor pools, other means of protection, such as self-closing doors with self-latching devices,

Rule .18(11)(i)

keypads, card readers or key entry devices which are approved by the appropriate administrative authority, shall be accepted so long as the degree of protection afforded is not less than the protection of an outdoor facility.

(j) Barriers shall be located so as to prohibit permanent structures, equipment or similar objects from being used to climb the barriers. There shall be a clear zone of not less than thirty-six inches around the exterior of the barrier.

(k) A spa with a safety cover that complies with ASTM F1346, “Performance Specification for Safety Covers and Labeling Requirements for All Covers for Swimming Pools, Spas and Hot Tubs” shall be exempt from the provisions of this section. Swimming pools with safety covers shall not be exempt from the provisions of this rule.

(l) Windows on a building that form part of a barrier around a pool shall have a maximum opening width not to exceed four inches. If designed to be opened, windows shall also be provided with a non-removable screen.

(m) For a passage through a wall separating the indoor portion of a pool from an outdoor portion of the same pool, the overhead clearance of the passage to the pool floor shall be at least six feet eight inches to any solid structure overhead.

(12) **Warning Signs for Swimming Pools.** Signs shall be provided as follows:

(a) The words "No Diving" and the universal international symbol for “NO DIVING” shall be permanently visible at the edge of the deck for water five feet in depth or less, placed only on the deck beside the depth markers, and shall conform to that outlined for depth markers in subsections (3)(a) thru (h) above.

(b) Where no lifeguard is on duty, a sign or signs shall be placed in clear view at or near the entrance to the pool and shall state in clearly legible letters at least four inches high “WARNING - NO LIFE GUARD ON DUTY and RISK OF DROWNING - SUPERVISE CHILDREN CLOSELY”.

(c) The same sign in subsection (b) above or an additional sign will state under the heading “Pool Risks” the following items in clearly legible letters at least one inch high:

1. Shower before entering the water.
2. Children shall not use pool without an adult in attendance.
3. Adults should not swim alone.
4. All children three years old and younger and any child not potty-trained must wear snug fitting plastic pants or a water resistant swim diaper.
5. Do not swim if the suction outlets are missing, broken, or not clearly visible from the deck.
6. No glass articles allowed in or around pool.
7. Do not swallow the pool water.
8. Do not dive unless diving area is clear of other bathers.
9. Do not swim if you had diarrhea within the past two weeks.
10. No animals are allowed in the pool or pool enclosure, except service animals are allowed on the deck.

(13) **Warning Signs for Spas.** Signs shall be provided as follows:

(a) Signage which states safety, emergency and operational aspects of the spa, shall be prominently located near the spa.

(b) Warning signs for spas shall be in clear view of the spa and prominently displayed. Signs shall state the spa's address, the location of the nearest telephone with references that emergency telephone numbers are posted at the location. These emergency telephone numbers should include the name and phone number of the nearest available police, fire or ambulance service, and "911" if available. Signs shall include, but not be limited to the following messages:

Rule .18(13)(b)

1. Risk of Fetus Damage. Hot water exposure limitations vary from person to person. Pregnant women and small children should not use spa without medical approval.

2. Risk of Drowning. Other persons suffering from heart disease, diabetes, high or low blood pressure, and other health problems should not enter the spa without medical approval.

3. Risk of Drowning. Do not use the spa while under the influence of alcohol, narcotics, or drugs that cause sleepiness and drowsiness or raise/lower blood pressure.

4. Risk of Drowning. Use caution when bathing alone. Overexposure to hot water may cause nausea, dizziness, and fainting. Lower water temperatures are recommended for young children and for extended use (more than10-15 minutes).

5. Risk of Drowning. Do not use or operate spa if the suction fitting is missing, broken, or loose.

6. Risk of Child Drowning. Unsupervised use by children is prohibited. Children under five shall not use the spa.

7. Risk of Injury. Check spa temperature before entering. The spa temperature should not exceed 104^0F.

8. Risk of Injury. Enter and exit slowly.

9. Risk of Injury. Keep all glass and breakable objects out of the spa area.

10. Risk of Shock. Never place electrical appliances (telephone, radio, or televisions) within five feet of the spa.

(c) A sign shall be posted stating the hours of operation in clear view near the entrance and shall state the theoretical peak occupancy.

(14) In all swimming pools built prior to December 31, 2016 which have floor slopes greater than that allowed in this chapter or which have other construction variances to

this chapter, the health authority may require a warning sign stating the possible hazard to be posted in public view.

(15) **Obstructions and Entrapment Avoidance.** There shall be no obstructions that might injure or entrap a user. Types of entrapment include, but are not limited to, wedge or pinch-type openings and rigid cantilevered protrusions.

(16) At least one drinking fountain shall be provided and available to users at the pool site.

(17) A minimum of one rinse shower shall be provided on the pool deck of all public pools and spas. Water used for rinse showers may be at ambient temperature.

(18) Class C multi-family residential housing pools are exempt from the requirements of (17) and (18) of the rule, if the facility is only open to residents and their guests.

[Authority: O.C.G.A. Secs. 31-2A-6, 31-12-8, 31-45-10.]

511-3-5-.19 Dressing Facilities and Sanitary Facilities.

(1) **Dressing Facilities.** Adequate dressing facilities for pools, spas and recreational water parks shall be provided adjacent to the pool unless adequate dressing facilities are provided elsewhere on the general premises in close proximity to the pool.

(a) Handicapped accessible dressing and sanitary facilities shall meet all state and local requirements, and may be included as part of the required total number of water closets, shower heads, and lavatories. Dressing rooms may be combined with sanitary facilities, so long as all other requirements of this Rule are met.

(b) Dressing facilities, when provided, shall be have separations for each sex with no interconnection. The rooms shall be well-lighted, drained, ventilated, and of good construction with impervious materials. They shall be developed and planned so that good sanitation can be maintained throughout the building at all times.

Rule .19)(1)

(c) Partitions between portions of the dressing room area, screen partitions, shower, toilet and dressing room booths shall be of durable material not subject to damage by water and shall be designed so that a waterway is provided between partitions and floor to permit thorough cleaning of the walls and floor areas with hoses and brooms.

(d) There shall be at least one shower for each sex for facilities less than 4000 square feet of water surface area. One additional shower head for each sex shall be added for each additional 4000 square feet of water surface area or fraction thereof. These showers, when provided, may be used in place of the deck showers. However, the use of deck showers may not be substituted for dressing facility showers.

(e) Hot and cold water under pressure shall be provided in dressing facility showers.

(f) Floors of the dressing facility shall be free of joints or openings and shall be continuous throughout the areas. Floors shall have a slip-resistant surface that shall be relatively smooth to insure thorough cleaning. Floor drains shall be provided and floors shall be sloped not less than one-fourth inch per foot toward the drains to insure positive drainage.

(g) An adequate number of three-fourths inch hose bibs shall be provided for flushing down the dressing facility interior.

(2) **Sanitary Facilities.** Lavatories and toilets shall be provided for all public pools and spas and shall be located no more than 300 feet from the entrance; provided, however, that and increased risk pool shall have sanitary facilities no more than 200 feet from the entrance.

(a)The minimum criteria for lavatories and toilets for public pools shall be based upon the theoretical peak occupancy as established. The occupancy is divided evenly, fifty percent female and fifty percent male for these determinations.

Rule .19(2)

(b) All public pools shall provide one water closet, one lavatory and one urinal for the first fifty male users. One additional water closet, lavatory and urinal shall be provided for each additional one hundred fifty male users or fraction thereof.

(c) All public pools shall provide two water closets and two lavatories for the first fifty female users. One additional water closet and lavatory shall be provided for each additional one hundred female users or fraction thereof.

(d) All spas shall provide at least one water closet and lavatory for each sex.

(e) Soap dispensers for providing either liquid or powdered soap shall be provided at each lavatory. The dispenser shall be of all metal or plastic. No glass shall be permitted in these units.

(f) At least one paper towel dispenser or hand blow dryer shall be provided for every three lavatories.

(g) An unbreakable mirror may be provided over each lavatory.

(h) Toilet paper holders shall be provided at each water closet.

(i) Soap, paper towels, and toilet tissue shall be provided in all dispensers.

(j) Fixtures shall be installed in accordance with local plumbing codes and shall be properly protected against back-siphonage.

(k) Fixtures shall be designed so that they may be readily and frequently cleaned and disinfected without damage.

(l) At least one trash receptacle will be available in toilet areas.

(m) Facilities shall provide a minimum of one diaper changing station in the male and female bathroom or dressing area.

(n) Sanitary facility fixtures and dressing area fixtures and furniture shall be cleaned and sanitized with an EPA-approved product and as needed to provide a clean and sanitary environment.

Rule .19(2)

(o) If a bodily fluid such as feces, vomit, or blood has contaminated a surface, facility staff shall limit access to the affected area until the following remediation procedures or department approved process has been completed;

1. Before disinfection, all visible contaminant shall be cleaned and removed with disposable cleaning products effective with regard to type of contaminant present, type of surface to be cleaned, and the location with the facility.

2. Contaminated surfaces shall be disinfected with one of the following:

(i) A 5,000 mg/l bleach disinfection solution, such as a 1:10 dilution of fresh household bleach with water; or

(ii) An equivalent disinfectant that has been approved by the U.S. EPA for bodily fluids disinfection.

3. The disinfectant shall be left on the affected area for a minimum of twenty minutes or as otherwise indicated on the disinfectant label directions.

[Authority: O.C.G.A. Secs. 31-2A-6, 31-12-8, 31-45-10.]

511-3-5-.20 Recreational Water Parks and Special Purpose Pools. The rule provides specifications for the design and operation of special purpose pools, such as amusement rides and water slides, whether used in recreational water parks or aquatic facilities as a standalone attraction or in combination with other attractions or pools. The design of special purpose pools shall comply with the specifications in this Rule and other applicable rules in this Chapter. This Rule describes several types of special purpose pools, but it is not intended to be an exhaustive list of such pools.

(1) **Deviation from requirements.**

(a) A special purpose pool may deviate from the requirements of this Chapter if and to the extent:

Rule .20(1)(a)

1. A variance from this regulation is obtained from the Department to accommodate the design and use of the special purpose pool; or

2. The design and construction of the attraction meet sound engineering practice and present no health or safety hazard; and

3. The facility provides appropriate supervision onsite during hours of operation.

(b) If combined pool types are approved within a recreational water park or aquatic facility, each pool must comply with the applicable chapter provisions as if the pool functioned independently.

(c) The designing engineer and manufacturer, if applicable, must verify that the device or design meets the applicable American Society for Testing and Materials standard or Consumer Product Safety Commission regulation.

(2) **Interactive Water Play Pool.**

(a) The water supply for an interactive water play pool must, at all times, meet the requirements relating to water quality set forth in DPH Rule 511-3-5-.17.

(b) The interactive water play pool must be equipped, at its lowest point, with an unvalved drain of sufficient capacity and design to prevent the accumulation of water in the pool. Any direct suction outlets shall be prohibited.

(c) If an interactive water play pool is positioned near a deeper water swimming pool, then it must be located at the shallow end and must be separated from the deeper water by at least ten feet of deck, or by a barrier or fence meeting the requirements of this section. The design shall meet the following:

1. The minimum size of the tank shall be equal to the volume of two and one-half minutes of the combined flow of all feature pumps and the filter pump.

Rule .20(2)(c)

2. Adequate access shall be provided to the reservoir. Stairs or a ladder shall be provided as needed to ensure safe entry into the tank.

3. When an underground reservoir is utilized, an automatic skimmer system shall be provided. A variable height skimmer may be used or a custom surface skimmer device may be substituted if deemed appropriate by both the design engineer and the health authority.

4. The filter system shall be capable of filtering and treating the entire water volume of the reservoir tank within thirty minutes. The filter system shall draft from the tank and return filtered and treated water to the tank through equally spaced inlet fittings.

5. The water feature pump shall draft from the reservoir tank and an automatic water level controller shall be provided.

6. The flow rate through the feature nozzles of the water features shall be such as not to harm the patrons and shall not exceed twenty feet per second unless justified by the design engineer and by the fountain system manufacturer.

7. An overfill waste line with air gap shall be provided and a means of vacuuming and completely draining the tank shall be provided.

8. Depth markers are not required.

(d) Interactive water play pools floor slope shall be at least one foot in twelve feet vertical to horizontal or gentler slope.

(e) The density factor used to determine theoretical peak occupancy shall be eight square feet per bather and one person per fifteen square feet of deck area.

(f) A barrier shall be provided to separate an interactive water play, wading, and wading interactive pools from other bodies of water within the same facility. The barrier shall comply with this Chapter unless:

Rule .20(2)(f)

1. The pool is separated by a distance of at least ten feet from other bodies of water;

2. If the aquatic facility consists only of one or more increased risk pools, such as interactive water play pools, then the requirements for an enclosure between pools are not required; or

3. A variance has been approved by the Department.

(g) For zero-depth-entry into pools, the floor slope shall be at a one foot in twelve feet vertical to horizontal or gentler slope. Trench drains shall be used along zero depth entries at the waterline to facilitate surface skimming.

(3) **Water slides.**

(a) A water slide shall consist of one or more flumes, landing pools, or slide runouts, a pump reservoir, and facilities for the disinfection and chemical treatment of the water.

(b) The structural design of a water slide and the materials used in its construction must conform to generally-accepted structural engineering practices and must provide a sound, durable structure that will safely sustain all the dead loads, operational loads, water loads, rider loads, and environmental loads encountered.

(c) All components of a water slide that come into contact with bathers must be assembled, arranged, and finished so that their external surfaces and edges do not present an injury hazard to the skin of users under casual contact.

(d) The owner of a water slide and the state registered professional engineer who designs and certifies the slide construction are responsible for the safe design and construction of the entire facility.

(e) The design engineer shall comply with this chapter and must provide documentation certification that the water slide design conforms to the following standards or any successor standards;

Rule .20(3)(e)

1. ASTM F2376-13 Standard Practice for Classification, Design, Manufacture, Construction, and Operation of Water Slide Systems;

2. ASTM F2469-09 Standard Practice for Manufacturer, Construction, Operation, and Maintenance of Aquatic Play Equipment; and

3. Ga. Rules & Regs. Chapter 120-3-27 Amusement Rides.

(f) **Flumes.**

1. Each flume of a water slide must be water-tight. Its surfaces must be inert, nontoxic, smooth and easily cleaned.

2. Flume material shall be demonstrated as strong enough to support specified loads.

3. Flume components, maintained using the manufacturer's instructions, shall not deteriorate over time in such a way that a hazard will develop.

4. If a tube-type flume is used, it must be designed or ventilated to prevent a hazardous concentration of toxic disinfectant fumes under all circumstances of operation.

5. Open flumes shall be configured to contain the rider or vehicle under all reasonable operating conditions.

6. Open water slide flumes shall be kept clear of obstacles within the water slide clearance envelope. Flume risers sections may be added to block access to anything encroaching in the area.

7. Water slides shall have additional sidewall height provided by a flume riser section on the outside part of all horizontal curves to contain the rider.

8. Lateral centripetal forces shall be considered in curved sections of flumes. Predicted rider speeds should be used to calculate these forces.

9. The flume must be designed and constructed so as to prevent bathers from falling out of the flume in elevated sections where a bather might be able to stop contrary to intended use.

Rule .20(3)(f)

10. The construction, dimensions and methods of mechanical attachment of a flume must provide a smooth and continuous surface through the entire length of the flume. Seams and joints shall be properly designed to prevent misalignment.

11. The walls of any flume must be designed so that the continuous and combined action of hydrostatic, dynamic and static loads, as well as normal environmental deterioration do not damage the flume bed to the extent of creating a structural failure that presents a hazard of injury to users or that requires frequent patch repairs that may weaken the structural integrity of the flume.

(g) **Flume exit.** The exit of any flume must be designed to ensure that bathers enter the landing pool or slide runout at a safe speed and angle of entry. If a slide that has two or more flumes, and there is a point of intersection between the centerlines of any two flumes, then the distance between that point and the point of exit for each intersecting flume must not be less than twenty feet, or thirty feet if any users exit a flume at high speed, or as otherwise certified by the design engineer.

(h) **Exit into landing pool.** If users exit the flume of a water slide into a landing pool, then the following requirements apply:

1. Landing pools shall be designed to decelerate and stop riders and allow them to exit the water slide without encountering an obstruction.

2. The exit path for riders shall not cross with the landing zone of other slides. The designated pool exit shall be such as to force the riders to move forward and away from the paths of riders from other flumes.

3. The flume must be horizontal and perpendicular to the wall of the pool at the point of exit.

4. The flume must be designed with an exit system that provides for safe entry into the landing pool or slide runout. Present practices for safe entry shall follow the

Rule .20(3)(h)4.

manufacturer's recommendation and ASTM standard. Other methods are acceptable as long as safe exit velocities and proper body altitudes are assured under normal use by the designing engineer;

5. The flume at pool entry shall be straight for the last eight of the water slide entering the pool. The exit must be flush with the vertical wall of the pool at the point of exit and not more than two inches above, nor less than six inches below, the normal operating level of the pool, unless otherwise certified by the design engineer; and

6. The distance between:

(i) The side wall for a body slide landing pool and that portion of the flume exit nearest the wall must be not less than five feet and for a tube slide landing pool not less than four and one-half feet at the points of measurement in the pool;

(ii) The centerline of the flume and the centerline of any adjacent flume must arranged to minimize the opportunity for contact with other riders when exiting the flumes of adjacent slides simultaneously and not be less than six feet at the point of exit; unless otherwise certified by the design engineer.

(iii) The point of exit and the side of the landing pool opposite the bathers as they exit must be of sufficient length to decelerate and stop riders and minimize the potential for contact with the landing pool wall or stationary objects like ladders or steps, must not be less than twenty if the flume ends above or below the normal operating water level of the pool, unless otherwise certified by the design engineer.

(i) **Landing Pools.**

1. When a landing pool is used at a water slide flume, it must be located at the end of the slide.

2. Except as otherwise provided in subsection (3)(e) above, or as certified by the design engineer, the water depth in a landing pool at the end of the flume must be minimum of

three and one-half feet from the normal operating water level to the floor. This depth must be maintained for distance of not less than twenty feet from the point of exit from the flume or not less than thirty feet if the point of exit is even with the normal operating water level. The landing pool for a high speed slide will require additional length and water depth. The health authority may waive these requirements if a special exit system is used or if the manufacturer or design engineer designates a safe exit configuration from the flume and safe entry into the landing pool.

3. Beyond the area of level floor required above, in the area of the pool opposite the point of exit from the flume or other falling-entry feature, the floor of the landing pool may have a constant slope or a slope upward of not more than l foot in 7 feet

4. If steps are provided instead of exit ladders or recessed steps with handrails, a handrail meeting the requirements of this Chapter must be provided at the steps opposite the point of exit from each flume.

(j) **Decks**. A deck must be provided along the exit side of the landing pool and along one or more of the other sides of the pool. The pump and reservoir must be accessible from a deck not less than three feet wide.

(k) **Means of access**.

1. A concrete walkway, steps, stairway, or ramp must be provided for access between the landing pool and the top of the flume.

2. The walkway or other means of access must:

(i) not retain standing water;

(ii) conform to the structural requirements of the local building code;

(iii) be at least four feet wide;

(iv) be provided with handrails;

(v) have a slip-resistant surface;

Rule .20(3)(k)2.

(vi) be separated from the flume by a physical barrier that is located a safe distance from the flume so that it cannot be touched by users of the flume.

(l) **Slide runouts.**

1. Slide runouts, if used, must have an exit opening or step, unless one or both of the walls of the runout are not more than nineteen inches in height.

2. Slide runouts must be designed with adequate length and water depth and sloped so as to bring the user to a safe stop.

(m) **Pump reservoirs**.

1. Pump reservoirs used in water slides must have sufficient volume to contain not less than two minutes of combined flow from all water treatment and flume pumps, or must contain enough water to ensure that the landing pool will maintain a constant water depth.

2. The interior of pump reservoirs must be water-tight with a hard trowel or equivalent slip-resistant finish.

3. Pump reservoirs must be accessible only to authorized persons. Intakes to the slide pump must be designed to allow cleaning without danger of trapping the operator.

(n) **Control of water.**

1. A surge-free automatic water makeup system with a manual override must be installed to maintain the normal operating water level of the landing pool at all times. An approved backflow prevention device must be provided.

2. The velocity of water at the weir or inlet grate must not exceed one and one-half feet per second.

3. The suction outlet drain of the falling-entry pool must be clearly visible from the deck with the flume water turned off.

Rule .20(3)

(o) **Waterslide Rules.** The operator of a water slide or other falling-entry feature shall post one or more warning signs at the entrance to the facility. A sign with the heading “Risk of Illness and Injury”, must state that the following types of conduct are prohibited within the facility:

1. Running, standing, kneeling, rotating, tumbling, or stopping in any flume or tunnel.
2. Rough playing on the slide or feature.
3. Diving or flipping while exiting from a flume or feature.
4. Use of the slide while under the influence of alcohol or drugs.
5. Use of the flume or feature by more than one person at a time.
6. Failure to obey the instructions of the pool attendant or lifeguard.
7. Failure to keep hands inside the flume while using the slide.
8. Failure to leave the falling-entry pool promptly after exiting from the slide.
9. The possession of any glass, bottle or food in or near any pool.
10. Entry into an area of grass or other vegetation and returning to slide, feature or pool.
11. The possession of any loose objects.
12. The use of any clothing other than the swimwear on the slide or feature.
13. Wearing any bracelet, watch, or other jewelry.

(p) **Precautions for safety.**

1. An attendant must be on duty at all times while a water slide is open for use. The attendant shall serve as the safety director of the slide. In that capacity, the attendant shall control crowds, keep bathers moving through the pool or runout in an orderly fashion, and control any unsafe behavior in the lower flumes, in the pool or runout, or on the decks at the base of the slide.

Rule .20(3)(p)

2. An attendant must be on duty at all times while the water slide is open for use. The attendant shall control bathers near the entrance, regulate the departure of each bather down the slide, and control any unsafe behavior in the upper flumes.

3. Radio or other means of communication acceptable to the health authority must be provided between the flume entry attendant and the splash pool or slide runout lifeguard

4. Each water slide must have a means to allow the flume entry attendant to monitor the slide exit.

(q) **Pool Slides.** All pool slides shall be designed, constructed, and installed to provide a safe environment for all bathers utilizing the slide in accordance with the applicable ASTM and CPSC standard.

1. Water depth at the slide exit shall be determined by the slide manufacturer.

2. The landing area in the pool shall be protected through the use of a float line, peninsula, or other similar design to prevent collision with other bathers.

3. Clear space shall be maintained to the pool edge and between other features per manufacturer requirements.

4. A barrier or netting shall be provided to prevent bather access underneath the pool slide where sufficient clearance is not provided. Openings in any barrier or netting shall not allow for the passage of a four inch sphere and no opening can create a finger entrapment.

5. Pool slides must have an attendant during hours of operation to monitor activity and compliance with the posted manufacturer warnings.

(4) **Activity pools.** Amusement devices used in activity pools must be designed and maintained so that their surfaces are smooth, nontoxic and easily cleanable. The devices must not pose a safety or health hazard to users and must not interfere with circulation or disinfection of the water. The pool and equipment shall meet the following;

(a) Play and water activity equipment shall be installed in accordance with the manufacturer's instructions.

(b) A rope and float line shall be provided to identify a water depth of more than four and one half feet in a constant floor slope configuration.

(c) Floating devices not intended to be mobile shall be anchored in a manner to restrict movement to the range established by the manufacturer; and

(d) ASTM F2469-09 Standard Practice for Manufacturer, Construction, Operation, and Maintenance of Aquatic Play Equipment and Consumer Product Safety Commission standards shall be met.

(5) **Wave pools.**

(a) The generation of waves more than three feet in height in a wave pool, regardless of the depth of the pool, must not continue for more than fifteen minutes at a time.

(b) The main drain must be clearly visible from the deck with the wave generating equipment turned off.

(c) Bather access to the wave pool shall be allowed only at the shallow or beach end. The sides of the pool must be protected from unauthorized entry into the pool by the use of a fence or other comparable barrier.

(d) Wave pools must be provided with handholds at the static water level. These handholds must be self-draining and must be installed so that their outer edge is flush with the pool wall. The design of the handholds must ensure that body extremities will not become entangled during wave action.

(e) Life jackets must be provided free for use by bathers who request them.

(f) Each permanent station for pool attendants and lifeguards must be provided with a clearly labeled and readily accessible emergency shut-off switch for the control of the

wave action. A minimum of two emergency shut-off switches to disable the wave action shall be provided, one on each side of the wave pool.

(g) An audible warning system must be provided to alert bathers of the beginning of wave generation.

(h) Stepholes and handrails must be provided at one or more locations along the wall of the wave pool. The stepholes and handrails must extend down the wall so they will be accessible during wave generation at the lowest water level. The distance between the handrail and the wall must not exceed six inches.

(i) A rope and float line shall be installed to restrict bather access to the wave pool caisson wall. The location of the rope and float line shall be in accordance with the wave equipment manufacturer’s instructions. The wall anchors shall be recessed and be made of corrosion-resistant material. A float line is not required to separate the first point of transition from shallow to deep.

(6) **Wading Interactive/Child amusement lagoons.** Devices used in child amusement lagoons must be designed and maintained so that their surfaces are smooth, nontoxic and easily cleanable.

(a) The devices must not pose a safety or health hazard to bathers and must not interfere with circulation or disinfection of the water.

(b) The devices shall comply with ASTM F2469-09 Standard Practice for Manufacturer, Construction, Operation, and Maintenance of Aquatic Play Equipment and Consumer Product Safety Commission Standards.

(7) **Leisure River, Continuous Water Channel** - **Watercourse rides.**

(a) Handrails, steps, stairs, and booster inlets for watercourse rides must not protrude into the watercourse.

(b) The watercourse must not be narrower than twelve feet and not deeper than three and one half feet.

(c) An approved method of exit must be provided at least every two hundred feet along the watercourse.

(d) A deck must be provided along at least one side of the water course.

(e) The design velocity of the water in a watercourse ride must not exceed two miles per hour.

(f) The design engineer of a continuous water course may deviate from the requirements in subsections (a) - (e) above if sound engineering and safety practices are met.

(g) All bridges spanning a watercourse shall have a minimum clearance of both seven feet from the bottom of the watercourse and four feet above the water surface to any structure overhead.

(8) **Sanitary and Dressing Facilities for Waterparks.** The design of the facility and the number of fixtures for the first 7500 square feet or fraction thereof of water available for bather access shall meet DPH Rule 511-3-5-.19. For every additional 7500 square feet or fraction thereof of water available for bather access at the facility, there shall be not less than one water closet for males, one urinal for males, one lavatory for males, one shower for males, two water closets for females, one lavatory for females and one shower for females.

(a) A rinse shower shall be on the deck or at entrance of each pool or attraction.

(b) Water used for rinse showers may be at ambient temperature.

[Authority: O.C.G.A. Secs. 31-2A-6, 31-12-8, 31-45-10.]

511-3-5-.21 Food Service.

(1) Food Service facilities shall comply with provisions of Article 13 of O.C.G.A. Chapter 26-2 and DPH Rule 511-6-1.

(2) Bathers shall not be allowed to eat or drink while in or partially in the water.

(3) Food and beverages shall only be served on non-breakable containers. The pool must be drained and vacuumed if any broken glass enters the water.

(4) Covered trash containers shall be provided where food or beverages are available and allowed to be consumed.

[Authority: O.C.G.A. Secs. 31-2A-6, 31-12-8, 31-45-10.]

511-3-5-.22 Operation and Management.

(1) All swimming pools and spas covered by this Chapter shall be maintained under the supervision and direction of a properly trained operator who shall be responsible for the sanitation, safety, and proper maintenance of the pool and all related equipment, and for and daily recordkeeping.

(2) The trained operator shall have a current certificate showing completion of an approved operator training course. A copy or the original certificate or documentation shall be available onsite for inspection by the Health Authority.

(3) The trained operator may be an employee or a contract service provider.

(4) Training for the operator can be obtained by completion of a course approved by the Department.

(5) The trained operator must perform a minimum of two visits weekly and be able to provide assistance whenever needed.

(6) Written documentation of the operator's visits must be available at the pool facility. At a minimum, the written record must indicate the condition of the following items:

(a) The circulation, filtration, and disinfection systems,

Rule .22(6)

(b) safety equipment on-site,

(c) pool stairs and deck condition,

(d) water chemistry test results and,

(e) record what corrective actions, if necessary, were taken by the operator.

(7) Facilities without an on-site trained operator must appoint a responsible on-site person. This individual must be capable of testing the water chemistry as required by the chapter, and must be trained to perform the requirements in DPH Rule 511-3-5-.16.

(8) The responsible person must receive training on basic pool operations from the trained operator, or from a local health department course if available.

(9) **Water Testing Frequency.** The trained operator or responsible person shall collect water samples from the water in the pool for monitoring. An in-line sampling port may be used for water held in reservoirs. The water quality testing frequency shall be as follows:

(a) For pools, free available chlorine or total bromine and pH shall be tested a minimum of two times daily during the hours of operation.

(b) Total alkalinity shall be tested weekly and calcium hardness shall be tested monthly.

(c) If stabilized chlorine is used as the primary disinfectant, the operator shall test cyanuric acid every two weeks. Otherwise, cyanuric acid shall be tested monthly. Cyanuric acid shall be tested twenty-four hours after addition to the water.

(d) For spas and hot water venues, free available chlorine, total bromine, pH and water temperature shall be tested prior to opening and recorded every four hours.

(e) In-line oxidation reduction potential readings (if applicable) shall be recorded at the same time the free available chlorine or total bromine and pH tests are performed.

(f) If in-line electrolytic chlorinators are used, salt levels shall be tested at least weekly or per manufacturer’s instructions.

Rule .22

(10) **Water Testing Procedure.** The pool operator or responsible person shall acquire a water sample for testing the chemical parameters:

(a) The sample shall be obtained from at least eighteen inches below the surface of the water and from a location between the inlets.

(b) The sample shall be obtained from a section of the pool that has a water depth of between three to four feet when available.

(c) For each water test, sampling locations shall rotate around the shallower end of the pool. The pool operator shall include the deepest area of the pool in the water sampling rotation once per week.

(11) If the water test results are not in compliance with DPH Rule 511-3-5-.17, the operator shall close the pool, record findings, and make the necessary adjustments to the water chemistry to comply with the chapter. The chemicals used and amounts shall be recorded on the operator log.

(12) A safety self-inspection shall be conducted daily by the trained operator or responsible person and documented on a log sheet.

(13) **Fecal and Non-Fecal Contamination Response Plan.** All public swimming pools shall have a written contamination response plan for responding to incidents of formed-stool, diarrheal-stool, and vomitus contamination. Such incidents shall be recorded and managed by the trained operator or responsible person as follows:

(a) A log shall be maintained to record each occurrence of contamination in the water or on the adjacent deck area for formed or diarrheal fecal material, whole stomach discharge of vomitus, and blood.

(b) After an incident, the public swimming pool will be closed for the time required to achieve the correct contact concentration and time (CT) value (CT, mg-min/L) for the

hazard, in accordance with the most recent recommendations published by the Centers for Disease Control and Prevention.

(14) Upon completion of any swimming pool or spa, the manager and his operators shall be given complete written and oral instructions by the builder as well as operational guidance of the pool, all equipment and the maintenance of the swimming pool water.

(15) The theoretical peak occupancy limit shall be observed by the management. A sign stating the occupancy shall be posted in a visible location near the entrance in four inch letters and numbers. The maximum number of bathers to be allowed in a pool enclosure at one time shall be based on DPH Rule 511-3-5-.05(12).

(16) Management shall establish an inclement weather policy for the safety of the bathers.

[Authority: O.C.G.A. Secs. 31-2A-6, 31-12-8, 31-45-10.]

511-3-5-.23 Compliance Procedures.

(1) A swimming pool, spa, or recreational water park shall not operate until a valid operating permit has been issued by the health authority after inspection.

(2) An operating permit shall not be issued until appropriate inspections show compliance with the requirements of this Chapter, with no violations noted on the inspection report.

(3) The health authority shall inspect the swimming pool, spa, or recreational water park for compliance as follows:

(a) Swimming pools, spas, or recreational water parks which open on or after April 1 and which close on or before October 31 shall be inspected at least once during the period of operation.

Rule .23(3)

(b) All other swimming pools, spas, or recreational water parks shall be inspected at least twice each year. Additional inspections may be made as determined necessary by the health authority.

(c) The inspection, testing and monitoring frequency may be changed by the health authority based on the occurrence of injury and illness or inspection history.

(d) The operator shall receive a copy of the inspection and place it in a location protected from the weather and in public view as designated by the health authority.

(e) Representatives of the health authority, after proper identification, shall be permitted to enter any swimming pool or spa facility or the grounds of any recreational water park at any reasonable time for the purpose of making inspections to determine compliance with this Chapter.

(4) **Inspection Report Ratings and Imminent Hazards**. The inspection report used will be as adopted by the Georgia Department of Public Health.

(a) An unsatisfactory rating will be given when any of the following occurs:

1. When an imminent health hazard as described in subsection (5) below is found;

2. when any two or more violations are found; or

3. when any violation is repeated on a follow up inspection.

(b) A satisfactory rating will be given:

1. When no more than one non-imminent health hazard violation is found, and

2. when there are no repeat violations on a follow-up inspection.

(c). A follow-up inspection shall be performed within thirty days from the date of an unsatisfactory rating.

(d) Violations which are not imminent health hazards shall be corrected within thirty days, or upon a timeframe in the plan of correction approved by the local health authority.

(e) An unsatisfactory rating may result in suspension or revocation of the operating permit.

(5) **Imminent Health Hazards**. Items that are considered imminent health hazards include the following:

(a) During operation, disinfectant levels are less than the minimum level specified in DPH Rule 511-3-5-.17. If the level of the disinfectant used is not specified in DPH Rule 511-3-5-.17, the disinfectant must be approved and kept at levels determined necessary by the health authority.

(b) During operation, the pH is less than the minimum or more than the maximum levels allowed in DPH Rule 511-3-5-.l7.

(c) The pump, automatic disinfectant equipment, or other equipment necessary for continuous filtration and disinfection of the swimming pool, spa, or recreational water park attraction is not working or is unable to maintain adequate turnover rate.

(d) The water turbidity is such that the suction outlet/main drain cover or a standard white marker tile on the bottom of the deepest portion of the pool cannot be seen.

(e) Broken glass or sharp objects in the water or on the deck area;

(f) Broken, unsecured, or missing main drain, or any submerged suction outlet cover/grate;

(g) Failure to provide and maintain a barrier to inhibit unauthorized access to the outdoor facility when required;

(h) Absence of required lifesaving equipment on the deck or an emergency phone;

(i) Number of bathers exceeds the posted peak occupancy;

(j) Use of an unapproved or contaminated water supply source for potable water use;

(k) A fecal matter contamination in the water; and

(l) Other hazards as determined by the health authority.

Rule .23

(6) Fecal incidents shall be recorded and reported to the local health authority at the time of the incident.

(7) The health authority may require the preparation of a water sampling and a water safety plan by an appropriate professional when operational conditions and bather health and safety warrant such action.

(8) **Voluntary Closure.** In lieu of suspension or revocation of a permit, a swimming pool, spa, or recreational water park attraction may be allowed to voluntarily close until such time as the violations resulting in an unsatisfactory rating are corrected. The health authority shall inspect the premises within two working days of notification that the hazard has been corrected by the operator.

(9) **Suspension or Revocation.** The health authority may deny permit applications, and may suspend or revoke permits, for failure to comply with the provisions of this Chapter. When an application for a permit is denied or the permit previously granted is to be suspended or revoked, the applicant or holder thereof shall be afforded notice and an opportunity for a hearing.

(a) The action of the health authority is effective upon service of a written notice thereof, and operation must cease immediately in the case of a suspension or revocation.

(b) The notice must state the basis for the action and advise the permit holder or applicant of the right to a hearing on request within 72 hours.

(c) If requested, the hearing will be conducted by an experienced supervisory level employee of the health authority not directly involved in the suspension.

(d) The rules of evidence will not apply, but both the health authority and the permit holder or applicant may present witnesses, documents, and argument.

(e) The hearing official will be authorized to rescind, affirm, or modify the action, and may impose conditions on any decision allowing the pool to operate.

Rule .23(9)

(f) If a hearing is not requested, the owner may request an inspection to reinstate the permit after correcting all violations.

(g) **Notice of Hearing.** A notice of hearing is properly served when delivered in person, or by registered or certified mail, to the owner, operator, responsible person, or authorized agent of the swimming pool, spa, or recreational water park.

(h) If the permit holder or applicant is unsatisfied by the decision of the hearing officer, then it may pursue an appeal to the Department in accordance with Code Section 31-5-3.

[Authority: O.C.G.A. Secs. 31-2A-6, 31-12-8, 31-45-10.]

511-3-5-.24 Environmental Health Personnel

(1) All Environmental Health personnel who are assigned responsibilities in public swimming pool plan review, permitting, inspecting, or other means of enforcing this Chapter, must complete a state approved exam demonstrating knowledge of the public swimming pool chapter.

(2) All in-service training must be approved by the local Environmental Health supervisor or lead personnel. Employee attendance records of approved training shall be maintained in the county of employment and shall be subject to Department monitoring.
