# Supplementary material for: Assessing state partner use of the Model Aquatic Health Code (MAHC): A cross comparison of five states with varying degrees of self-reported adoption status
Source: PLOS Water. Author manuscript; Available in PMC 2025 Jul 11. (PMC12247143; doi:10.1371/journal.pwat.0000276)
Supplement: S6_Text — S6 Text. New Mexico pool codes. New Mexico Title 7 health, Chapter 18 aquatic venues established in August 2018. (PDF) [file NIHMS2046419-supplement-S6_Text.pdf]

TITLE 7 HEALTH
CHAPTER 18 AQUATIC VENUES
PART 1 PUBLIC AQUATIC VENUES: GENERAL PROVISIONS

7.18.1.1 ISSUING AGENCY: New Mexico Environmental Improvement Board.
[7.18.1.1 NMAC - N, 8/1/16]

7.18.1.2 SCOPE: Owners and operators of public aquatic venues or other public bathing attractions.
[7.18.1.2 NMAC - N, 8/1/16]

7.18.1.3 STATUTORY AUTHORITY: Sections 74-1-1 through 74-1-16 NMSA 1978.
[7.18.1.3 NMAC - N, 8/1/16]

7.18.1.4 DURATION: Permanent.
[7.18.1.4 NMAC - N, 8/1/16]

7.18.1.5 EFFECTIVE DATE: August 1, 2016, unless a later date is cited at the end of a section.
[7.18.1.5 NMAC - N, 8/1/16]

7.18.1.6 OBJECTIVE: To protect public health and safety by establishing standards and provisions for the regulation of public aquatic venues and other public bathing attractions.
[7.18.1.6 NMAC - N, 8/1/16]

7.18.1.7 DEFINITIONS: [RESERVED]

7.18.1.8 ENFORCEMENT AUTHORITY:
A. Private aquatic venues shall not be subject to the provisions of this regulation or 7.18.2 NMAC, Aquatic Venues: Fees.
B. Authorized department representatives shall be responsible for the enforcement of this rule.
[7.18.1.8 NMAC - Rp, 7.18.2.9 NMAC, 8/1/16]

7.18.1.9 ADOPTION OF MODEL AQUATIC HEALTH CODE: Except as otherwise provided, the United States department of health and human services center for disease control and prevention Model Aquatic Health Code, 1st Edition is hereby incorporated by reference.
[7.18.1.9 NMAC - N, 8/1/16]

7.18.1.10 ADOPTION BY REFERENCE: Outside standards, listings, and publications referenced in this rule are part of this rule.
[7.18.1.10 NMAC - Rp, 7.18.2.8 NMAC, 8/1/16]

7.18.1.11 MODIFICATIONS, ADDITIONS, AND OMISSIONS: Except as otherwise provided, the following modifications, exceptions and omissions are made to the Model Aquatic Health Code, 1st Edition.
A. Modifications to Aquatic Code definitions. The following terms defined in the Model Aquatic Health Code, 1st Edition have the meanings set forth herein, in lieu of the meanings set forth in the Model Aquatic Health Code, 1st Edition:
(1) "Aquatic venue" means any artificially constructed structure that is expressly designated or used with the knowledge or consent of the owner or operator for swimming, water recreation, or bathing for the use of any segment of the public. Such structures do not necessarily contain standing water, so water exposure may occur via contact, ingestion, or aerosolization. The term "aquatic venue" includes all class A, class B, class C, and class D aquatic venues and spas. This term does not include residential housing or lodging facilities having five or fewer living units. Plumbing fixtures associated with a specific living unit, hot springs, and fill-and-draw tubs are also excluded. The term "aquatic venue" includes, but is not limited to, public pools and spas owned or operated by:
(a) travelers' accommodations including hotels, motels, inns, lodging and bed and breakfast facilities, hostels and recreational vehicle parks;
(b) residential housing or lodging facilities having six or more living units;
(c) apartments or apartment complexes, condominiums and mobile home parks;

(d) recreation parks;
(e) colleges or universities;
(f) schools and group homes;
(g) organizational camps;
(h) clubs;
(i) associations;
(j) business establishments for their patrons or employees;
(k) private persons with pools that are open to the public;
(l) recreation districts; or
(m) cities, municipalities, counties, the state of New Mexico or other political subdivisions.

(2) "Interactive water play aquatic venue" means any indoor or outdoor installation that includes sprayed, jetted or other water sources contacting bathers and not incorporating standing or captured water as part of the bather activity area. Only those intended for public use and recreation shall be regulated. These aquatic venues are also known as splash pads, spray pads, wet decks.

B. Additions to the Aquatic Code definitions. The following terms not defined in the Model Aquatic Health Code 1st Edition have the meanings set forth herein:

(1) Abbreviations.
(a) "CC" means combined chlorine.
(b) "DPD" means diethyl-p-phenylene diamine.

(2) "Approved" means accepted in writing by the department.

(3) "Class A aquatic venue" means any public aquatic venue including, but not limited to, general admission pools, aquatic centers, recreation parks, schools, colleges and universities, organizational camps, daycare facilities, clubs, recreation districts, city, municipal, county and state pools and pools operated by other political subdivisions as defined by law. Class A aquatic venues shall not include pools located at boarding schools, colleges and universities exclusively associated with specific living units but would not be open to the entire boarding school, college or university population or the general public.

(4) "Class B aquatic venue" means any public aquatic venue, located at, and operated to serve a facility having six or more living or guest units at:
(a) travelers' accommodations, including hotels, motels, inns, lodging, campgrounds, bed and breakfast facilities;
(b) apartments, condominiums, retirement homes, assisted-living facilities, mobile home parks;
(c) class A exclusions for boarding schools, colleges, and universities, and group homes;
(d) businesses that employ 10 or more people and own a swimming pool or spa that is for the exclusive use of employees and their guests; or
(e) homeowners associations, if the pool is for the exclusive use of the association members and their guests only and no memberships are sold to outside persons.

(5) "Class C aquatic venue" means a public aquatic venue that is designed specifically as a bathing attraction or for sporting or recreational purposes and may include, but is not limited to, special features such as:
(a) wave pools;
(b) diving pools;
(c) splash pools;
(d) zero depth pools;
(e) waterslides;
(f) vortex pools;
(g) interactive play attractions;
(h) watercourse rides;
(i) activity pools;
(j) portable pools;
(k) spray pads;
(l) lazy rivers; or
(m) wading pools.

(6) "Class D aquatic venue" means any public aquatic venue used for physical therapy or rehabilitation including, but not limited to, post-operative strength training, assistance of buoyancy of water, and other one-on-one training.

(7) "Club" means a facility constructed to provide entertainment, athletic or physical conditioning for its members, guests, invitees, occasional users, patrons, or clientele. It includes, but is not limited to, racquetball clubs, country clubs, golf clubs, health spas, fitness, sports and wellness facilities or aerobics instruction facilities.

(8) "Combined chlorine (CC)" means that portion of the total residual chlorine that is combined with ammonia or nitrogen compounds and will not react chemically with undesirable or pathogenic organisms.

(9) "Department" means the New Mexico environment department.

(10) "Department representative" means the secretary of the environment department or his/her designees.

(11) "Engineer" means any individual currently registered and in good standing under the "New Mexico Engineering and Surveying Practice Act."

(12) "New construction" means the activity of building or installing a public aquatic venue, and its component parts, where no such structure has previously existed or where previously existing aquatic venue structures have been removed.

(13) "Non-substantial alteration" means the alteration, modification, or renovation of an aquatic venue (for outdoor or indoor aquatic facilities) where the total cost of the work does not exceed fifty percent of the replacement cost of the aquatic venue.

(14) "Person" means:

(a) any person, individual, any public or private firm, partnership, corporation, company, society, association, and every managing body, officer, agent or employee thereof; or

(b) the state, local government, or any agency, institution or political subdivision thereof, including any governing or managing body.

(15) "Private aquatic venue" means:

(a) any pool or spa owned by no more than four individuals, either jointly, individually or through association, incorporation or otherwise, for the exclusive use of the occupants thereof and their guests or invitees; or

(b) an aquatic venue owned by a business employing fewer than 10 persons if the aquatic venue is for the exclusive use of employees and their guests.

(16) "Service animal" means a guide dog, signal dog, or other animal trained to do work or perform tasks for the benefit of an individual with a disability including, but not limited to, guiding individuals with impaired vision, alerting individuals with impaired hearing to intruders or sounds, or providing minimal protection or rescue work, such as pulling a wheelchair or fetching dropped items. Dogs whose sole function is to provide comfort or emotional support do not qualify as service animals.

(17) "Variance" means written permission from the department to use an alternative measure of compliance with any provision in the aquatic venue rules, except those specifically prohibited in the rule. The alternative measure shall provide public health and safety protection that is equal to or greater than the protections provided in this rule.

C. **Omissions to the Aquatic Code.** The following provisions of the Model Aquatic Health Code, 1st Edition are omitted:

(1) Subpart 4.1.4: Compliance Certificate;
(2) Section 4.1.5.3: Permit Issuance;
(3) Section 4.7.3.2.7.1: Feeders for pH Adjustment – Provided;
(4) Section 4.7.3.2.8.1.1: Automated Controllers – Installed;
(5) Subpart 5.2.2: Exemptions;
(6) Section 5.6.7.4: Water Replenishment;
(7) Section 5.7.5.2: Manual Disinfectant Feed System; and
(8) Section 6.3.2.1(4): List of aquatic facilities requiring qualified lifeguards.

[7.18.1.11 NMAC - N, 8/1/16]

7.18.1.12 CONSTRUCTION PERMITS:

A. No person shall construct or substantially alter a public aquatic venue, or part thereof, or convert a private aquatic venue into a public aquatic venue without first:

(1) submitting a construction permit application that shall include plans, specifications, supporting material, and other information required by the department;

(2) receiving a construction permit; and

(3) paying all applicable fees.

B. No person shall deviate from the approved plans and specifications during the construction or alteration of public aquatic venues described in this rule without first receiving prior written approval from the department.

C. Construction permits will be issued only to the owner or authorized agent of the owner.

D. A construction permit may be issued only when the facility owner or agent has provided sufficient information for the department to determine that the aquatic venue will:

(1) operate continuously in a clean and sanitary manner;

(2) not constitute a hazard to public health and safety; and

(3) provide health and safety protection equal to or greater than that required by the aquatic venue rules.

E. Persons submitting plans and specifications for a proposed public aquatic venue that demonstrate a new technology or alternative mode of operation not contemplated in these rules shall apply for a variance.

F. The department shall issue a construction permit, issue a construction permit with conditions, or deny the construction permit application. The department may revoke a construction permit under 7.18.1.19 NMAC or suspend a construction permit if the department determines that the provisions of the aquatic venue rules are not met.

G. The department shall notify the applicant in writing that the application is complete or incomplete within 30 days of receipt of the application.

H. Once the department determines that the application is complete, for class B aquatic venues, the department shall have up to 30 working days to issue the permit, issue the permit with conditions, or deny the permit application. For class A, C, and D aquatic venues, the department shall have up to 90 working days to issue the permit, issue the permit with conditions, or deny the permit application. All construction permit denials shall be in writing stating the reason the permit was denied. The applicant for a permit that has been denied may request an administrative hearing. The request for a hearing shall be made in writing to the department within 15 calendar days after notice of the department's decision has been received by the applicant. Hearings on permit denials shall be held in accordance with 7.18.1.21 NMAC and 20.1.5 NMAC.

I. Private aquatic venues that are converted into public aquatic venues or public aquatic venues that were previously operating without a permit shall be subject to the aquatic venue rules including, but not limited to, the requirement to obtain a construction permit.

J. Non-substantial alterations do not require a construction permit; however an aquatic facility owner planning a non-substantial alteration shall contact the department to review proposed changes prior to starting the non-substantial alteration.

[7.18.1.12 NMAC - Rp, 7.18.2.10 NMAC, 8/1/16]

7.18.1.13 PLANS AND SPECIFICATIONS:

A. Nothing in the swimming pool rules shall prevent the department from requiring the correction of errors in plans and specifications after those plans have been approved or the specifications accepted. The department may also revoke any construction permits or approvals that are issued in error, or obtained based upon material misrepresentations or erroneous information provided by the applicant.

B. The department may also issue a stop work order whenever construction work deviates from approved plans and specifications without prior written approval from the department, violates any permit condition, or is in violation of this or any other law or regulation. The department shall provide written notice of the stop work order to the person performing the work or causing the work to be performed, and the person receiving such notice shall cease and desist from performing, or causing the performance of, the work until authorized in writing by the department to proceed.

[7.18.1.13 NMAC - Rp, 7.18.2.11 NMAC, 8/1/16]

7.18.1.14 OPERATING PERMITS:

A. No person shall operate an aquatic venue without an operating permit from the department. Each aquatic venue in an aquatic facility shall be permitted separately.

B. Operating permits shall be issued for a period of 12 consecutive months and are non-transferrable between facilities or persons.

C. No person shall operate an aquatic venue without:

(1) receiving a construction permit from the department, if applicable;

(2) obtaining an approved final construction inspection, if applicable;

(3) initially applying for a permit to operate such aquatic venue;

(4) successful completion of a department inspection;

(5) receiving a permit from the department; and

(6) paying all applicable fees.

D. The permit shall remain the property of the department and shall be removed by the department representative when a permit is suspended or revoked.

E. Operating permits for all public aquatic venues shall expire on the last day of the anniversary month of the date of original issue.

F. Any public aquatic venue that has been closed or not operated for a period of 30 days or more shall be granted permission to re-open only after completion of a department re-opening inspection that demonstrates compliance with applicable aquatic venue rules.

G. The designated qualified operator shall request a re-opening inspection for an aquatic venue that has been closed for a period of 30 days or more, at least two weeks prior to the desired re-opening date. The department may require the qualified operator to be present for this inspection.

H. Operating permits are non-transferable between facilities or persons.

[7.18.1.14 NMAC - Rp, 7.18.2.12 NMAC, 8/1/16]

7.18.1.15 INSPECTION FREQUENCY: The department shall inspect each public aquatic venue at least annually and shall make as many re-inspections as necessary for enforcement of the aquatic venue rules.

[7.18.1.15 NMAC - Rp, 7.18.2.14 NMAC, 8/1/16]

7.18.1.16 SERVICE OF NOTICE:

A. Notice shall be deemed to be properly served when the original or a true copy of the inspection report form or other written notice has been delivered personally to the permit holder, the permit holder's agent, or a qualified operator; or when such notice has been sent by registered or certified mail to the last known address of the permit holder or qualified operator on file with the department.

B. A copy of such notice shall be filed in the department's records.

[7.18.1.16 NMAC - Rp, 7.18.2.15 NMAC, 8/1/16]

7.18.1.17 TEMPORARY SUSPENSION OF PERMITS:

A. The department may suspend a permit at any time when it determines that there is a violation that may affect public health or safety.

B. Whenever a permit holder or operator has failed to comply with any of the requirements of this rule, the permit holder or operator shall be notified in writing.

C. The notice shall:

(1) identify and reference the conditions that violate the aquatic venue rules;

(2) specify the time period within which such condition shall be brought into compliance, if any;

(3) state that failure to comply with any notice issued pursuant to the aquatic venue rules may result in immediate permit suspension; and

(4) advise that the permit shall be suspended if the permit holder or operator is still out of compliance at the end of five working days following the deadline for compliance, unless a request for a hearing is delivered to the department by the permit holder within the five-day period.

D. Immediate suspension: notwithstanding other provisions of this regulation, whenever a department representative finds a condition in a public aquatic venue's operation that constitutes an immediate hazard to public health, welfare, or safety, the department representative may, without prior warning, notice, or hearing, issue a written notice to the permit holder or operator citing such condition. The department's notice shall state that the permit is immediately suspended and all swimming or bathing of any kind is to be immediately discontinued.

E. All persons receiving a permit suspension notice shall immediately comply with the notice's terms.

F. For immediate suspensions, suspensions upheld after a hearing, and where no request for a hearing has been received, the department shall post a sign stating that the aquatic venue is closed.

G. The department may also require a written compliance plan.

[7.18.1.17 NMAC - Rp, 7.18.2.16 NMAC, 8/1/16]

7.18.1.18 REINSTATEMENT OF SUSPENDED PERMITS:

A. Any person whose permit has been suspended may make application for reinstatement of the permit in writing to the department. Within five working days following receipt of such a request, a department representative shall inspect the aquatic venue or premises or both. If the applicant is in compliance with the requirements of the aquatic venue rules and other applicable laws, regulations, and ordinances, the permit shall be reinstated.

B. The department may require a qualified operator or aquatic venue employee to attend additional training courses in aquatic venue sanitation and safety.

[7.18.1.18 NMAC - Rp, 7.18.2.18 NMAC, 8/1/16]

7.18.1.19 REVOCATION OF PERMIT:

A. A permit may be revoked when:

(1) it appears that a violation may affect public health or safety;

(2) any conditions of a permit are violated;

(3) there are willful or repeated violations of any of the requirements in the aquatic venue rules;

(4) the permit has been obtained through nondisclosure, misrepresentation, or misstatement of a material fact; or

(5) the owner or the owner's agent interferes with the department's performance of its duties.

B. Prior to such revocation, the department shall provide an opportunity for a hearing. A department representative shall notify the permit holder in writing stating the reason the permit is subject to revocation and advising that the permit shall be revoked at the end of five working days following service of such notice, unless a request for a hearing is delivered to the department by the permit holder within the five-day period.

C. Owners and operators of public aquatic venues who fail to comply with the provisions of a suspension notice or open their facility to the public without an approved permit will be subject to the penalties described in 7.18.1.56 NMAC.

[7.18.1.19 NMAC - Rp, 7.18.2.19 NMAC, 8/1/16]

7.18.1.20 OTHER REMEDIES: [RESERVED]

7.18.1.21 HEARINGS:

A. Hearings provided for in the aquatic venue rules shall be held within 15 working days of a petitioner's delivery of a hearing request to the department.

B. Hearings provided for in this regulation shall be conducted in accordance with 20.1.5 NMAC.

[7.18.1.21 NMAC - Rp, 7.18.2.22 NMAC, 8/1/16]

7.18.1.22 VARIANCE:

A. The department may grant a variance from the design and construction or operation and maintenance provisions of the aquatic venue rules through written permission for the use of alternative measures that will provide public health and safety protection that is equal to or greater than the protections provided in the aquatic venue rules. No variances shall be granted for procedural requirements, such as submitting construction or operating permit applications, including paying fees, obtaining construction or operating permits, operator certifications, or requesting a hearing.

B. Specific variance requests shall be made by the owner or the owner's designated agent. Designated agents shall provide written documentation signed by the owner that they are representing the owner regarding the specific variance application. All variance applications shall be signed by the owner and upon change of ownership or transfer of property, the new owner or their designated agent must re-apply for the variance.

C. It is the applicant's responsibility to provide all necessary information to support the request for a variance.

D. Any person applying for a variance from any provision of the aquatic venue rules shall do so by filing a written application with the department. Applications shall:

(1) be made on forms obtained from the department;

(2) remit applicable fee by check or money order made payable to the "water recreation facilities fund;"

(3) state the applicant's name and mailing address;
(4) state the date of the application;
(5) state the provision or provisions of this regulation for which the variance is sought;
(6) state in detail the extent to which the applicant wishes to vary from the provision or provisions;
(7) state the period of time for which the variance is sought;
(8) state why the applicant believes the variance is justified;
(9) be accompanied by any relevant documents or material which the applicant believes would support the application for a variance; and
(10) contain other relevant information the department may request.

E. Within 20 working days following receipt of a completed variance application, the department shall grant the variance, grant the variance subject to conditions, or deny the variance. The action taken by the department shall be by written order, a copy of which shall be sent to the applicant. The order shall:
(1) state the applicant's name and address;
(2) state the date the order is made;
(3) describe the location of the public aquatic venue; and
(4) state the department's decision and its reasons.

F. If a variance is granted, the order will state the effective period of time and any conditions that apply.

G. All variances shall be reviewed at the time of the annual operating permit inspection to determine whether all variance conditions have been met. If conditions of the variance have not been met, an operating permit shall not be issued.

H. Petitioners who are dissatisfied with the department's decision may request a hearing from the department secretary.
(1) The request shall be made in writing to the department secretary within 15 calendar days after notice of the department's decision has been received by the petitioner.
(2) Unless a request has been received within the 15 calendar day period, the department's decision shall be final.
(3) If a request has been received within the 15 calendar day period, the department secretary or his/her designated representative shall hold a hearing within 15 working days after the receipt of the request.

I. The department shall notify the petitioner by certified mail of the date, time and place of the hearing.

J. In the hearing, the burden of proof shall be upon the petitioner.

[7.18.1.22 NMAC - Rp, 7.18.2.23 NMAC, 8/1/16]

7.18.1.23 VOIDING OF VARIANCES:

A. An approved variance shall be void one year after the date of approval if the permitted activities granted thereby have not been utilized. If the department voids a variance for any reason, the department will serve written notice on the permit holder.

B. The department may void a variance if conditions of the variance have not been met, or if subsequent events show that the variance has created or may create conditions hazardous to the public health, safety, or welfare.

C. An approved variance shall be void if it is utilized in a way that violates the terms and conditions of the variance. Voiding a variance is in addition to, and not instead of, other remedies available to the department at any time for violation of the aquatic venue rules.

D. All variances shall become void upon change of ownership. Upon change of ownership, the new owner shall re-apply for a variance.

E. Any person who has been granted a variance shall sign a department approved indemnification and release of liability statement form. Variances shall not be valid unless and until the department receives a completed and signed indemnification and release of liability statement form back from the applicant.

[7.18.1.23 NMAC - Rp, 7.18.2.24 NMAC, 8/1/16]

7.18.1.24 RIGHT OF ENTRY:

A. Upon presentation of credentials, department representatives may enter any premises where a public aquatic venue is located or where records required by the aquatic venue rules are located during the aquatic venue's operating hours.

B. When entry is denied by the property owner, the department may seek a district court order to:

(1) have a right of entry to, upon, or through any premises where an aquatic venue is located;

(2) have a right of entry on any premises where any records required by the aquatic venue rules or by permit condition are kept;

(3) have access to and copy any records that the aquatic venue rules or a permit requires the facility to maintain;

(4) inspect any premises or equipment to determine compliance with the aquatic venue rules or any permit or variance condition; and

(5) obtain any sample(s) required to determine compliance with the aquatic venue rules or any permit or variance condition.

[7.18.1.24 NMAC - Rp, 7.18.2.25 NMAC, 8/1/16]

7.18.1.25 LIFEGUARDS: When swim teams and swimming exercise classes are the only users of an aquatic facility, in lieu of a qualified lifeguard the owner or designated agent may allow substitution of a swim coach attendant, who is certified by the *American red cross* or an equivalent organization in first aid and cardiopulmonary resuscitation (CPR) and is trained to deal with safety hazards. Both the owner of the public aquatic venue and the sponsoring organization furnishing the swim coach shall be responsible for assuring proper credentials, training and bather controls are maintained in accordance with these requirements.

[7.18.1.25 NMAC - N, 8/1/16]

7.18.1.26 POOL WATER QUALITY:

A. Testing equipment:

(1) All public aquatic venues shall have fully functional water quality testing devices for measuring the pH, free and combined chlorine concentration, or bromine, (or concentration of other approved disinfectant), and cyanuric acid if stabilized chlorine is used.

(2) Water quality testing devices shall use environmental protection agency (EPA) approved methods.

(3) Water quality testing devices for measuring free and total chlorine or bromine shall use *diethyl-P-phenylene diamine* (DPD) as the reagent.

(4) Feeders for pH adjustments and automated controllers shall be required on all aquatic venues within two years of the effective date of these regulations.

(5) All aquatic venues using a manual disinfectant feed system that does not have an automated controller shall be tested before the venue opens for the day and every four hours while open to the public.

B. The total available bromine in aquatic venues shall not exceed 8ppm.

C. Cyanuric acid shall not be used in indoor aquatic facilities.

D. Swim-up bars are considered an increased risk aquatic venue and shall install a secondary disinfection system.

[7.18.1.26 NMAC - N, 8/1/16]

7.18.1.27 TEMPORARY SPECIAL USE AQUATIC VENUES: Owners of aquatic venues that are used for public events at sports fields, county fairs, portable pools and similar special uses shall be reviewed by the department on an individual case basis. The department may require special conditions as part of approval of such pools to assure health and safety.

[7.18.1.27 NMAC - N, 8/1/16]

7.18.1.28 - 7.18.1.50 [RESERVED]

7.18.1.51 CONSTRUCTION: This part shall be liberally construed to carry out its purpose.

[7.18.1.51 NMAC - Rp, 7.18.2.51 NMAC, 8/1/16]

7.18.1.52 SEVERABILITY: If any provision or application of this part is held invalid, the remainder of this part, or its application to other situations or persons, shall not be affected.

[7.18.1.52 NMAC - Rp, 7.18.2.52 NMAC, 8/1/16]

7.18.2.53 REFERENCES IN OTHER REGULATIONS: Any reference to the aquatic venue regulations or to any prior version of the aquatic venue regulations in any other rule shall be construed as a reference to this rule. References to the "aquatic venue rules" in this part refer to all provisions contained in 7.18.1 through 7.18.2 NMAC.
[7.18.1.53 NMAC - Rp, 7.18.2.53 NMAC, 8/1/16]

7.18.1.54 SAVINGS CLAUSE: Repeal or supersession of prior versions of this part or the public swimming pool rules shall not affect any administrative or judicial action initiated under those prior versions.
[7.18.1.54 NMAC - Rp, 7.18.2.54 NMAC, 8/1/16]

7.18.1.55 COMPLIANCE WITH OTHER REGULATIONS: Compliance with the aquatic venue rules or this part does not relieve a person from the responsibility to comply with any other applicable federal, state, or local regulations.
[7.18.1.55 NMAC - Rp, 7.18.2.55 NMAC, 8/1/16]

7.18.1.56 PENALTY: Any person who violates any provision of this rule shall be subject to the penalty provisions in Section 74-1-10 NMSA 1978 of the Environmental Improvement Act, in addition to any other penalties provided for in the aquatic venue rules.
[7.18.1.56 NMAC - Rp, 7.18.2.56 NMAC, 8/1/16]

7.18.1.57 LIMITATION OF DEFENSE: The existence of a valid permit for the installation, modification or operation of an aquatic venue shall not constitute a defense to a violation of any section of this rule, except the requirement for obtaining a permit.
[7.18.1.57 NMAC - Rp, 7.18.2.57 NMAC, 8/1/16]

HISTORY OF 7.18.1 NMAC:

History of Repealed Material: [RESERVED]

Other History:
7.18.2 NMAC, Public Swimming Pools, Spas and Baths: General Provisions (filed 6/12/2008), repealed and replaced by 7.18.1 NMAC, Public Aquatic Venues: General Provisions, effective 8/1/2016.

The Model Aquatic Health Code

The Code

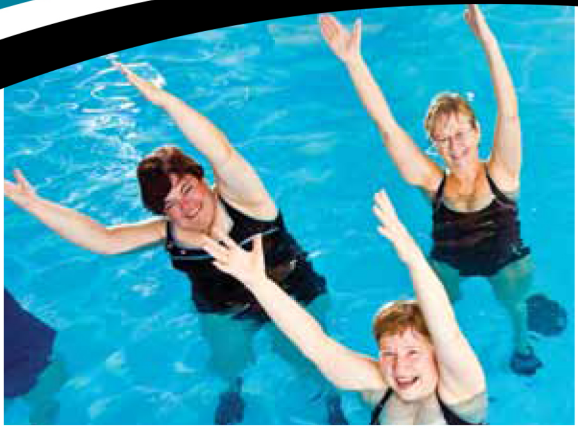

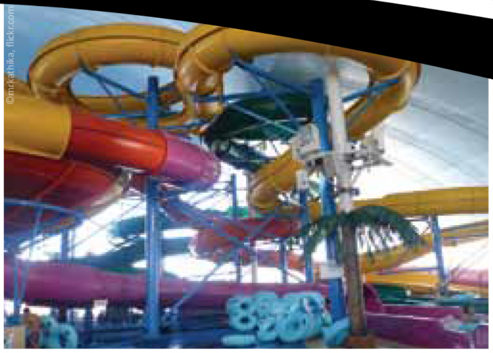

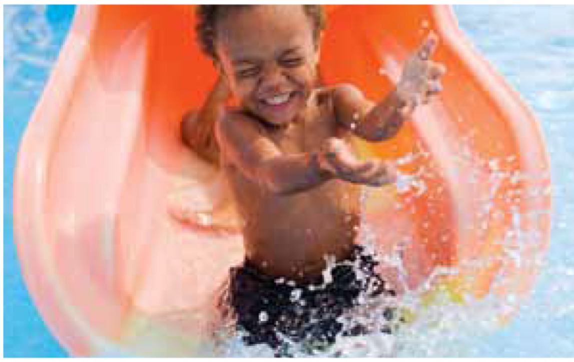

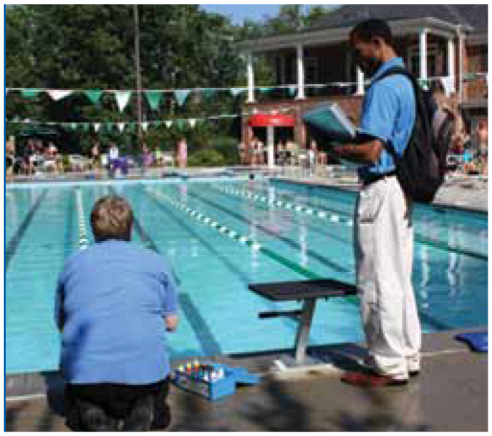

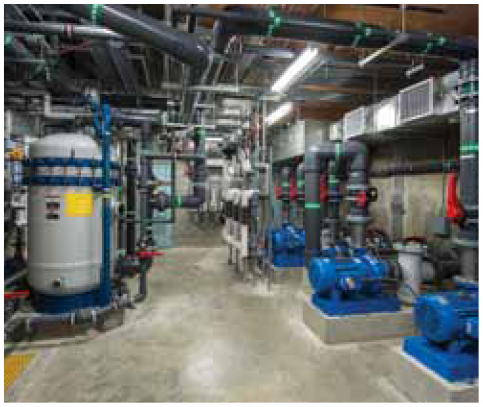

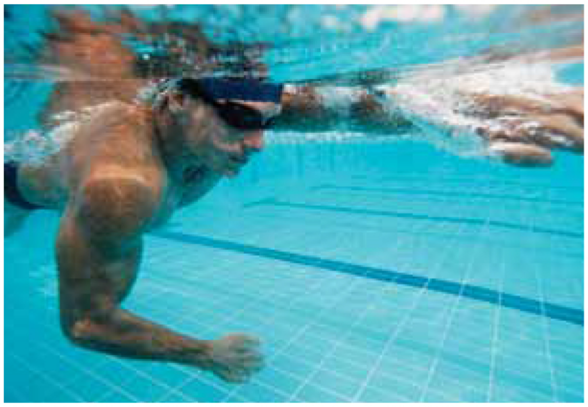

MAHC - The Code
1st Edition
August 2014

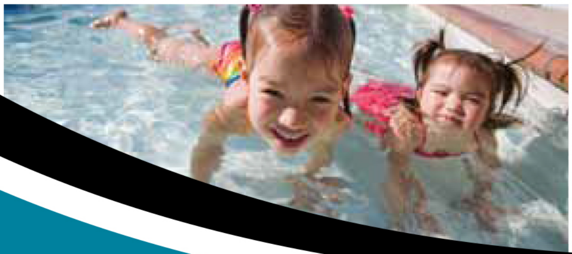

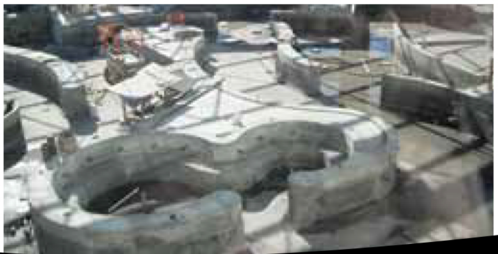

U.S. Department of Health and Human Services
Centers for Disease Control and Prevention

CS249779

Model Aquatic Health Code, 1st Edition

Posted on 08/29/2014

This information is distributed solely as guidance for the purpose of assisting state and local health departments, pool programs, building officials, the aquatics sector, and other interested parties in improving the health and safety at public aquatic facilities. This document does not address all health and safety concerns associated with its use. It is the responsibility of the user of this document to establish appropriate health and safety practices and determine the applicability of regulatory limitations prior to each use.

Foreword

Swimming, soaking, and playing in water have been global pastimes throughout written history. Twentieth-century advances in aquatics—combining disinfection, recirculation, and filtration systems—led to an explosion in recreational use of residential and public disinfected water. As backyard and community pool use has swept across the United States, leisure time with family and friends around the pool has increased. Advances in public aquatic facility design have pushed the horizons of treated aquatic facilities from the traditional rectangular community pool to the diverse multi-venue waterpark hosting tens of thousands of users a day. The expansion of indoor aquatic facilities has made the pool and waterpark into year-round attractions. At the same time, research has demonstrated the social, physical, and psychological benefits of aquatics for all age groups.

However, these aquatics sector changes—combined with changes in the general population, chlorine-tolerant pathogens, and imperfect bather hygiene—have resulted in significant increases in reports of waterborne outbreaks, with the greatest increase occurring in man-made disinfected aquatic venues. Drowning continues to claim the lives of far too many, especially children, and thousands of people visit hospitals every year for pool chemical-related injuries. The increase in outbreaks and continued injuries necessitates building stronger public health regulatory programs and supporting them with strong partnerships to implement health promotion efforts, conduct research, and develop prevention guidance. It also requires that public health officials continue to play a strong role in overseeing design and construction, advising on operation and maintenance, and helping inform policy and management.

The Model Aquatic Health Code *(MAHC)* is a set of voluntary guidelines based on science and best practices that were developed to help programs that regulate public aquatic facilities reduce the risk of disease, injury, and drowning in their communities. The MAHC is a leap forward from the Centers for Disease Control and Prevention's *(CDC)* operational and technical manuals published in 1959, 1976, and 1981 and a logical progression of CDC's Healthy Swimming Program started in 2001. The MAHC underscores CDC's long-term involvement and commitment to improving aquatic health and safety. The MAHC guidance document stemmed from concern about the increasing number of pool-associated outbreaks starting in the mid-1990s. Creation of the MAHC was the major recommendation of a 2005 national workshop held in Atlanta, Georgia charged with developing recommendations to reduce these outbreaks. Federal, state, and local public health officials and the aquatics sector formed an unprecedented collaboration to create the MAHC as an all-encompassing health and safety guidance document. The partnership hopes this truly will lead to achieving the MAHC vision of "Healthy and Safe Aquatic Experiences for Everyone" in the future.

Atlanta, Georgia

August 2014

Acknowledgments

Creating the MAHC has been a long process starting with the passage of a 2004 Council of State and Territorial Epidemiologists position statement, the subsequent 2005 national workshop, creation of the MAHC Steering Committee in 2007, recruitment for the 12 Technical Committees, two public comment periods, and a comprehensive cycle of editing and revising individual modules and incorporating them into the “knitted” version. As a result, many individuals have contributed greatly by devoting considerable time and effort to addressing concerns and developing recommendations that are now reflected in the MAHC 1st Edition. They brought this key national workshop recommendation to fruition. These individuals represent a diverse group of public health officials, educators, aquatics sector leaders, and consumer representatives acting through their agencies, companies, professional groups, or trade organizations.

It is only through the dedicated efforts and contributions of experienced professionals that a scientifically sound, well-focused, and up-to-date MAHC is possible. CDC acknowledges with immense gratitude the substantial assistance of those who contributed to public health and aquatic safety in the development of the MAHC. They deserve our heartfelt thanks and appreciation for volunteering their time, energy, and creativity to create the MAHC. In addition, we would like to also give our thanks to all the reviewers across the country who provided more than 4,400 public comments and spent a great deal of time combing through the detail of the MAHC code and annex and making suggestions for improvement. Their effort was worth the time investment; the MAHC was greatly improved after each of the two public comment periods.

MAHC Steering Committee

Doug Sackett, *Chair*
Michael Beach
Bart Bibler
Rob Blake
Chip Cleary
Tracynda Davis
Jim Dunn
Steven Goode
Joe Hunsaker
John Linn
David Ludwig
Colleen Maitoza
Chuck Neuman
Charles Otto
Bob Vincent

Steering Committee Assistants

Elaine Curtiss
CDR Jasen Kunz
Amanda Tarrier
Lee Tate

MAHC Technical Committees

Contamination Burden

Ellen Meyer, *Chair*
Lee Tate, *SC Liaison*
Anthony Carotenuto
Richard Carroll
Jose Gonzalez
Joe Hunsaker
Jeffrey Kosik
John Paccione
John Weber
Llew Withers

Disinfection & Water Quality

Jim Dingman, *Chair*
Tracynda Davis, *Vice Chair*
John Linn, *SC Liaison*
Terry Arko
Susan Campbell
Jerry Cormier
Beth Hamil
Tony Leigh
Leon Marquart
Bob Pryor
Jeffrey Sloan
Raj Solomon
Duane Stansbury
Terri Stroupe
Roy Vore

Facility Design & Construction

Carl Nylander, *Chair*
Colleen Maitoza, *SC Liaison*
Chris Baugh
Scott Bowron
Benny Burleson
Cory Cloward
Keith Cooper
Bob Foster
Michael Grimes
James Hogan
Robert Jelinek
Peter Jen
Alvaro Mendoza
Randy Mendiorez
Rob Morgan
Paul Sisson
Lee Tate

Facility Maintenance & Operation

Michael Beatty, *Chair*
Darby van Conover, *Vice Chair*
Lee Tate, *SC Liason*
Susan Campbell
James Cunningham
Carvin DiGiovanni
Deborah Fennel
Bob Foster
Frank Goldstein
James Hogan
Jeffrey Kosik
John McIlhargy
Garth Miner
David Roberts
Geoff Rogers
David Schwartz

Hygiene

Ralph Cordell, *Chair*
Tracynda Davis, *SC Liaison*
Scott Bowron
Lynita Docken
Bruce Flippens
Perry Fox
Brian Hodges
Dean Hyatt
Robert Jelinek
Jodi Jensen
Ted Kelly

Lifeguarding/Bather Supervision

Kathryn Scott, *Chair*
Roy Fielding, *Vice Chair*
Doug Sackett, *SC Liaison*
Richard Carroll
Dewey Case
James Cunningham
Patrick Daugherty
John Hunsucker
Daniel Jones
Stephen Keifer
Frank Pia
William Rich
Kay Smiley
Darby van Conover
Jill White

Monitoring & Testing

Vince Hill, *Chair*
Diane Miskowski, *Vice Chair*
Bob Vincent, *SC Liaison*
Neal Bloomenrader
Scott Bowron
Kevin Boyer
Jason Hammond
Tom Metzbower
John Pinckney
Joe Sweazy
Christopher Tate
Robert Tuttle
Tom Vyles

Operator Training

Dennis Berkshire, *Chair*
John Whitmore, *Vice Chair*
Doug Sackett, *SC Liaison*
Wayne Crokus
Tracynda Davis
Tom Donaldson
Lorraine Forston
Connie Harvey
Michele Hlavsa
Dean Hyatt
Steve Kraft
Colleen Maitoza
Mark Pabst
Carl Ralph
Kay Smiley
Raj Solomon
Joe Stefanyak
Greg Stockton
Mansoor Yasin

Recirculation Systems & Filtration

James Amburgey, *Chair*
Roy Fielding, *Vice Chair*
Chuck Neuman, *SC Liaison*
Michael Beatty
Susan Campbell
Joe Carpenter
Barry Gertz
Mike Fabiani
Garth Miner
David Schwartz
Kimberly Walsh
Mansoor Yasin

Regulatory Program Administration

Frank Guido, *Chair*
Tom Vyles, *Vice Chair*
Tracynda Davis, *SC Liaison*
Scott Bowron
Neal Bloomenrader
Scott Coughlin
Gary Fraser
Madeleine Gelsinon
Clyde Hegerfeld
Tressa Madden
Daryl Matzke

Risk Management & Safety

Amy Duck, *Chair*
Kelly Bernish, *V Chair*
Amanda Tarrier, *SC Liaison*
Maria Bella
James Cunningham
Carvin DiGiovanni
Claudia Duncan
Lee Engvall
Chris Gordon
Chevelle Glymph
Jason Hammond
Janina Jarvis
Karin Mack
Ellen Meyer
Robert Morgan
Mick Nelson
Barry Rice
Kay Smiley

Ventilation & Air Quality

Franceen Gonzales, *Chair*
Richard Cavestri, *Vice Chair*
Doug Sackett, *SC Liaison*
Chip Blatchley
Susan Campbell
Lilia Chen
Keith Coursin
Jim Dunn
Kevin Dunn
Mike Groves
Michele Hlavsa
TJ Liston
John McIlhargy
Mark Moore
Chuck Neuman

Jeff Radue
Steve Vehige

Other Technical Committee Members

Steve Ashworth
Brian Freber
Steve Hawksley
Ron Robol

CDC Employees & Contractors

Kate Awsumb
Michael Beach
Molly Berkery
Maggie Byrne
Robert Blake
Staci Close, *Contractor*
Elaine Curtiss, *Contractor*
Julie Gilchrist
Michele Hlavsa
Heather Horton
Jon Kennon
CDR Jasen Kunz
CAPT Charles Otto
Montrece Ransom
Gouthami Rao, *Contractor*
Teresa Sims, *Contractor*
Pam Wigington

Funding

CDC
CDC Foundation
National Swimming Pool Foundation
Arch Chemical/Lonza

Table of Contents

Foreword ..... *ii*

Acknowledgments ..... *iii*

Table of Contents ..... *vii*

1.0 Preface *44*

1.1 Introduction 44
1.1.1 Rationale 44
1.1.2 Need for Further Guidance 44
1.1.3 Responsibility of User 44
1.1.4 Original Manufacturer Intent 44
1.1.5 Local Jurisdiction 45

1.2 Recreational Water-Associated Illness Outbreaks and Injuries 45
1.2.1 RWI Outbreaks 45
1.2.2 Significance of *Cryptosporidium* 45
1.2.3 Drowning and Injuries 45
1.2.4 Pool Chemical-Related Injuries 45

1.3 Model Aquatic Health Code 45
1.3.1 Background 45
1.3.2 MAHC Vision and Mission 46
1.3.3 Science and Best Practice 46
1.3.4 Process 46
1.3.5 Open Access 47
1.3.6 Updating the MAHC 47
1.3.7 Authority 47
1.3.8 CDC Role 47
1.3.8.1 Public Health Role 47
1.3.8.2 Model Guidance 47

1.4 Public Health and Consumer Expectations 47
1.4.1 Aquatics Sector & Government Responsibility 47
1.4.2 Swimmer Responsibility 48

1.5 Advantages of Uniform Guidance 48
1.5.1 Sector Agreement 48
1.5.2 MAHC Provisions 48
1.5.3 Aquatic Facility Requirements 49

1.6 Modifications and Improvements in the MAHC 1st Edition 49

1.7 MAHC Adoption at State or Local Level 49
1.7.1 MAHC Adoption at State or Local Level 49
1.7.2 Conference for the Model Aquatic Health Code 49

1.8 The MAHC Revision Process 50
1.8.1 MAHC Revisions 50

1.8.2 Future Revisions ... 50

2.0 User Guide ... 51

2.1 Overview ... 51
2.1.1 New Users ... 51
2.1.2 Topic Presentations ... 51

2.2 MAHC Structure and Format ... 51
2.2.1 Numbering System ... 51
2.2.2 Title, Keyword, Phrase Text ... 52
2.2.3 MAHC Requirement ... 52
2.2.4 Illustrations ... 52
2.2.5 Consistency Between Chapters 4.0 and 5.0 ... 52
2.2.6 Conventions ... 52
2.2.7 Definitions ... 52

2.3 Annex ... 53
2.3.1 Rationale ... 53
2.3.2 Content ... 53
2.3.3 Bibliography ... 53
2.3.4 Appendices ... 53

3.0 Glossary of the MAHC Code and Annex ... 54

3.1 Acronyms and Initialisms Used in This Code and Annex ... 54

3.2 Glossary of Terms Used in This Code and Annex ... 56

4.0 Facility Design Standards and Construction ... 70

4.1 Plan Submittal ... 70
4.1.1 Plan Submittal ... 70
4.1.1.1 Purpose ... 70
4.1.1.2 Conform ... 70
4.1.1.3 Approved Plans ... 70
4.1.1.4 Plan Preparation ... 70
4.1.1.5 Required Statements ... 70
4.1.2 Content of Design Report ... 71
4.1.2.1 Basis of Design Report ... 71
4.1.2.1.1 Names / Addresses ... 71
4.1.2.1.2 Site Information ... 71
4.1.2.1.3 Plot Plan ... 71
4.1.2.2 Plans and Specifications ... 71
4.1.2.2.1 Drawings ... 71
4.1.2.2.2 Venue Attributes ... 71
4.1.2.2.3 Area Design ... 72
4.1.2.2.4 Venue Recirculation and Treatment Design ... 72
4.1.2.2.5 Equipment Room Design ... 72
4.1.2.2.6 Chemical Storage Space Design ... 73
4.1.2.2.7 Hygiene Facility Design ... 73
4.1.2.3 Technical Specifications ... 73

4.1.2.3.1 Accompanying Drawings 73
4.1.2.3.2 Technical Details 73
4.1.2.3.3 Water Sources 73
4.1.2.3.4 Area and Volume 73
4.1.2.3.5 Theoretical Peak Occupancy 73
4.1.2.3.6 Equipment Characteristics and Rating 74
4.1.2.3.7 Recirculation Rate and Turnover 74
4.1.2.3.8 Filter Media 75
4.1.2.3.9 Equipment Specifications 75
4.1.2.3.10 Safety Equipment Specifications 75
4.1.2.3.11 Other Specifications 75
4.1.3 Plan Approval 75
4.1.3.1 New Construction 75
4.1.3.1.1 Approval Limitations 75
4.1.3.1.2 Other Approvals 75
4.1.3.1.3 Plan Review Coordination 75
4.1.3.1.4 Plan Review Report 76
4.1.3.1.5 Plans Maintained 76
4.1.3.2 Non-Substantial Alterations 76
4.1.3.2.1 Alteration Review 76
4.1.3.2.2 Alteration Scope 76
4.1.3.3 Replacements 76
4.1.3.3.1 Replacement Equipment Equivalency 76
4.1.3.3.2 Replacement Approval 76
4.1.3.3.3 Emergency Replacement 76
4.1.3.3.4 Replacement Record Maintenance 77
4.1.3.3.5 Documentation 77
4.1.4 Compliance Certificate 77
4.1.4.1 Construction Compliance Certificate 77
4.1.4.2 Certificate Preparation 77
4.1.4.3 Certificate Statement 77
4.1.4.4 Systems Commissioning 77
4.1.4.5 Maintenance 77
4.1.5 Construction Permits 77
4.1.5.1 Building Permit for Construction 77
4.1.5.2 Remodeling Building Permit 78
4.1.5.3 Permit Issuance 78
4.1.5.4 Permit Denial 78
4.1.5.5 Documentation 78

4.2 Materials 78
4.2.1 Pools 78
4.2.1.1 Construction Material 78
4.2.1.2 Durability 78
4.2.1.3 Areas Subject to Freezing 78
4.2.1.4 Darker Colors 79
4.2.1.5 Competitive Pools 79
4.2.1.6 Design Parameters 79

4.2.1.6.1 Permission in Writing ... 79
4.2.1.7 Watertight ... 79
4.2.1.8 Smooth Finish ... 79
4.2.1.8.1 Daily Cleaning ... 79
4.2.1.8.2 Skimmer Pools ... 79
4.2.1.8.3 Gutter / Perimeter Overflow Systems ... 79
4.2.1.8.4 Dark Colors ... 79
4.2.1.9 Slip Resistant ... 79
4.2.1.10 Stainless Steel, Vinyl, PVC-P or PVC Pools ... 80
4.2.1.10.1 Damaged ... 80
4.2.1.11 Not Permitted ... 80
4.2.2 Indoor Aquatic Facility ... 80
4.2.2.1 Interior Finish ... 80
4.2.2.1.1 Relative Humidity ... 80
4.2.2.2 Condensation Prevention ... 80
4.2.2.2.1 Cold Weather ... 80
4.2.2.2.2 Paint or Coating ... 80
4.2.2.2.3 Perforated Interior-Finish Material ... 80
4.2.2.3 Mechanical Systems ... 81
4.2.2.3.1 Equipment Rooms ... 81
4.2.2.3.2 Chemical Storage Spaces ... 81
4.2.2.3.3 Indoor Aquatic Facility Air Pressure ... 81
4.2.2.3.4 Air Ducts ... 81
4.2.2.3.5 Filters ... 81
4.2.2.4 Indoor Aquatic Facility Doors ... 81
4.2.2.4.1 Corrosion-Resistant ... 81
4.2.2.4.2 Uncontrolled Condensation ... 81
4.2.2.4.3 Biological Contaminants ... 82
4.2.2.4.4 Air Leakage ... 82
4.2.2.4.5 Automatic Door Closer ... 82
4.2.2.5 Indoor Aquatic Facility Windows ... 82
4.2.2.5.1 Frames ... 82
4.2.2.5.2 Biological Contaminants ... 82
4.2.2.5.3 Thermal Breaks ... 82
4.2.2.6 Indoor Aquatic Facility Electrical Systems and Components ... 82

4.3 Equipment Standards ... 83
4.3.1 General ... 83
4.3.1.1 Accredited Standards ... 83
4.3.1.2 No Standards ... 83
4.3.1.2.1 Proof of Acceptability ... 83
4.3.2 Recirculation Systems and Equipment ... 83
4.3.2.1 ANSI-Accredited Testing ... 83
4.3.2.2 Suitable for Intent ... 83

4.4 Aquatic Venue Operation and Facility Maintenance [N/A] ... 83

4.5 Aquatic Venue Structure ... 83
4.5.1 Design for Risk Management ... 83

4.5.1.1 Basic Requirements ... 83
4.5.1.2 Water Clarity ... 84
4.5.1.2.1 Observing Water Clarity ... 84
4.5.1.2.2 Pools Over Ten Feet Deep ... 84
4.5.1.2.3 Visible ... 84
4.5.1.2.4 Spas ... 84
4.5.2 Bottom Slope ... 84
4.5.2.1 Parameters and Variance ... 84
4.5.2.2 Under Five Feet ... 84
4.5.2.3 Over Five Feet ... 84
4.5.2.4 Drain ... 84
4.5.3 Pool Access / Egress ... 85
4.5.3.1 Accessibility ... 85
4.5.3.2 Acceptable Means ... 85
4.5.3.3 Large Venues ... 85
4.5.4 Stairs ... 85
4.5.4.1 Slip Resistant ... 85
4.5.4.2 Outlined Edges ... 85
4.5.4.3 Deep Water ... 85
4.5.4.4 Rectangular Stairs ... 85
4.5.4.5 Dimensions ... 85
4.5.4.6 Stair Risers ... 87
4.5.4.7 Top Surface ... 87
4.5.4.8 Perimeter Gutter Systems ... 87
4.5.5 Handrails ... 87
4.5.5.1 Provided ... 87
4.5.5.2 Corrosion-resistant ... 87
4.5.5.3 Upper Railing ... 87
4.5.5.4 Wider Than Five feet ... 87
4.5.5.5 ADA Accessibility ... 88
4.5.5.6 Support ... 88
4.5.5.7 Dimensions ... 88
4.5.6 Grab Rails ... 88
4.5.6.1 Corrosion- Resistant ... 88
4.5.6.2 Anchored ... 89
4.5.6.3 Provided ... 89
4.5.6.4 Clear Space ... 89
4.5.6.5 Upper Railing ... 89
4.5.6.6 Support ... 89
4.5.7 Recessed Steps ... 89
4.5.7.1 Slip-Resistant ... 89
4.5.7.2 Easily Cleaned ... 89
4.5.7.3 Drain ... 89
4.5.7.4 Dimensions ... 89
4.5.7.5 Uniformly Spaced ... 91
4.5.7.6 Uppermost Step ... 91
4.5.7.7 Perimeter Gutter Systems ... 91
4.5.8 Ladders ... 91

4.5.8.1 General Guidelines for Ladders ... 91
4.5.8.1.1 Corrosion-Resistant ... 91
4.5.8.1.2 Anchored ... 91
4.5.8.2 Ladder Handrails ... 91
4.5.8.2.1 Two Handrails Provided ... 91
4.5.8.2.2 Clear Space ... 91
4.5.8.2.3 Upper Railing ... 91
4.5.8.2.4 Pool Wall ... 91
4.5.8.2.5 Support ... 91
4.5.8.3 Ladder Treads ... 92
4.5.8.3.1 Slip Resistant ... 92
4.5.8.3.2 Tread Depth ... 92
4.5.8.3.3 Uniformly Spaced ... 92
4.5.8.3.4 Upmost Ladder Tread ... 92
4.5.9 Zero Depth (Sloped) Entries ... 92
4.5.9.1 Slip Resistant ... 92
4.5.9.2 Maximum Floor Slope ... 92
4.5.9.2.1 Slope Changes ... 92
4.5.9.3 Trench Drains ... 92
4.5.9.3.1 Flat or Follow Slope ... 92
4.5.9.3.2 Handholds ... 93
4.5.10 Disabled Access ... 93
4.5.10.1 Conform to ADA Standards ... 93
4.5.11 Color and Finish ... 93
4.5.11.1 White or Light Pastel ... 93
4.5.11.1.1 Munsell Color Value ... 93
4.5.11.1.2 Exceptions ... 93
4.5.11.1.3 Darker Colors ... 93
4.5.12 Walls ... 93
4.5.12.1 Plumb ... 93
4.5.12.2 Support Ledges and Slopes ... 94
4.5.12.2.1 Contrasting Color ... 94
4.5.12.3 Rounded Corners ... 94
4.5.12.4 No Projections ... 94
4.5.13 Structural Stability ... 95
4.5.13.1 Withstand Loads ... 95
4.5.13.2 Hydrostatic Relief Valve ... 95
4.5.13.3 Freezing ... 95
4.5.14 Handholds ... 95
4.5.14.1 Handholds Provided ... 95
4.5.14.1.1 Installed ... 95
4.5.14.2 Horizontal Recesses ... 95
4.5.14.2.1 Drain ... 95
4.5.14.2.2 Consecutive Recesses ... 95
4.5.14.3 Decking ... 95
4.5.14.4 Coping Dimensions ... 96
4.5.14.5 Coping Thickness ... 96
4.5.15 Infinity Edges ... 96

4.5.15.1 Perimeter Restrictions 96
4.5.15.2 Length........ 96
4.5.15.2.1 Shallow Water........ 96
4.5.15.3 Handholds........ 96
4.5.15.4 Construction Guidelines........ 96
4.5.15.5 Overflow Basins........ 96
4.5.15.5.1 Finish........ 96
4.5.15.6 Maximum Height 96
4.5.16 Underwater Benches........ 97
4.5.16.1 Slip Resistant........ 97
4.5.16.2 Outlined Edges 97
4.5.16.3 Maximum Water Depth........ 97
4.5.16.4 Maximum Seat Depth........ 97
4.5.17 Underwater Ledges........ 97
4.5.17.1 Slip Resistant........ 97
4.5.17.2 Protrude 97
4.5.17.3 Five Feet or Greater........ 97
4.5.17.3.1 Underwater Toe Ledge 97
4.5.17.3.2 Below Water Level 97
4.5.17.4 Structural Support 97
4.5.17.5 Outlined........ 98
4.5.17.5.1 Visible 98
4.5.17.6 Tread Depths 98
4.5.18 Underwater Shelves 98
4.5.18.1 Immediately Adjacent 98
4.5.18.2 Nosing 98
4.5.18.3 Maximum Depth 98
4.5.19 Depth Markers and Markings 98
4.5.19.1 Location........ 98
4.5.19.1.1 Markings 98
4.5.19.1.2 Depth Measurements 98
4.5.19.1.3 Below Handhold 99
4.5.19.1.4 Coping or Deck........ 99
4.5.19.1.5 Read on Deck........ 99
4.5.19.1.6 Twenty-Five Foot Intervals........ 99
4.5.19.2 Construction / Size 99
4.5.19.2.1 Durable........ 99
4.5.19.2.2 Slip Resistant 99
4.5.19.2.3 Color and Height........ 99
4.5.19.2.4 Feet and Inches 99
4.5.19.3 Tolerance........ 100
4.5.19.4 No Diving Markers........ 100
4.5.19.4.1 Depths 100
4.5.19.4.2 Durable........ 100
4.5.19.4.3 Slip Resistant 100
4.5.19.4.4 At Least Four Inches........ 100
4.5.19.5 Depth Marking At Break in Floor Slope........ 100
4.5.19.5.1 Over Five Feet........ 100

4.5.19.5.2 Durable ... 100
4.5.19.5.3 Safety Rope ... 100
4.5.19.6 Dual Marking System ... 101
4.5.19.7 Non-Traditional Aquatic Venues ... 101
4.5.19.7.1 Clearly Visible ... 101
4.5.19.7.2 Lettering and Symbols ... 101
4.5.19.8 Wading Pool Depth Markers ... 101
4.5.19.9 Movable Floor Depth Markers ... 101
4.5.19.9.1 Vertical Measurement ... 101
4.5.19.9.2 Signage ... 101
4.5.19.10 Spas ... 101
4.5.20 Aquatic Venue Shell Maintenance [N/A] ... 101
4.5.21 Special Use Aquatic Venues ... 101
4.5.21.1 Adequately Support ... 101
4.5.21.2 Justification ... 102
4.6 Indoor / Outdoor Environment ... 102
4.6.1 Lighting ... 102
4.6.1.1 General Requirements ... 102
4.6.1.1.1 Outdoor Aquatic Venues ... 102
4.6.1.1.2 Accessible ... 102
4.6.1.2 Windows / Natural Light ... 102
4.6.1.3 Light Levels ... 102
4.6.1.4 Overhead Lighting ... 103
4.6.1.4.1 Artificial Lighting ... 103
4.6.1.4.2 Aquatic Venue Floor ... 103
4.6.1.4.3 Aquatic Venue Illumination ... 103
4.6.1.5 Underwater Lighting ... 103
4.6.1.5.1 Minimum Requirements ... 103
4.6.1.5.2 Dimmable Lighting ... 103
4.6.1.6 Night Swimming with No Underwater Lighting ... 103
4.6.1.6.1 Minimum Requirements ... 103
4.6.1.7 Emergency Lighting ... 104
4.6.1.7.1 Emergency Egress Lighting ... 104
4.6.1.7.2 Footcandles ... 104
4.6.1.8 Glare ... 104
4.6.2 Indoor Aquatic Facility Ventilation ... 104
4.6.2.1 Purpose ... 104
4.6.2.2 Exemptions ... 104
4.6.2.3 Indoor Aquatic Facility ... 104
4.6.2.4 Mechanical Code ... 104
4.6.2.5 ASHRAE 62.1 Compliance ... 104
4.6.2.6 Air Handling System Design ... 104
4.6.2.6.1 Mechanical Systems ... 104
4.6.2.6.2 Design Factors and Performance Requirements ... 105
4.6.2.6.3 Other Air Handling Systems ... 105
4.6.2.6.4 High Volume, Low Speed Fans ... 105
4.6.2.6.5 Occupied and Open All Seasons ... 105

4.6.2.6.6 Air Distribution Design 106
4.6.2.7 Performance Requirements for Air Handling Systems 106
4.6.2.7.1 Minimum Outdoor Air Requirements 106
4.6.2.7.2 System Alarm 106
4.6.2.7.3 Real-Time Occupancy 106
4.6.2.7.4 Air Delivery Rate 106
4.6.2.7.5 Consistent Air Flow 106
4.6.2.7.6 Relative Humidity 106
4.6.2.7.7 Negative Air Pressure 107
4.6.2.7.8 Disinfection By-Product Removal 107
4.6.2.7.9 Re-Entrainment of Exhaust 107
4.6.2.7.10 Access Control 107
4.6.2.7.11 Purge 107
4.6.2.7.12 Air Handling System Filters 108
4.6.2.8 Air Handling System Installation 108
4.6.2.8.1 Air Handling System Procedures 108
4.6.2.9 Air Handling System Commissioning 108
4.6.2.9.1 System Commissioning 108
4.6.2.9.2 Written Statement 108
4.6.3 Indoor Aquatic Facility Electrical Systems and Components 109
4.6.3.1 General Guidelines 109
4.6.3.1.1 NEC Requirements 109
4.6.3.1.2 Indoor Aquatic Facilities 109
4.6.3.2 Electrical Equipment in Interior Chemical Storage Spaces 109
4.6.3.2.1 Wet and Corrosive 109
4.6.3.2.2 Electrical Conduit 109
4.6.3.2.3 Electrical Devices 109
4.6.3.2.4 Protected Against Breakage 109
4.6.4 Pool Water Heating 109
4.6.4.1 High Temperature 109
4.6.4.2 Pressure Relief Device 110
4.6.4.3 Code Compliance 110
4.6.4.4 Equipment Room Requirements 110
4.6.4.5 Exception 110
4.6.5 First Aid Area 110
4.6.5.1 Station Design 110
4.6.6 Emergency Exit 110
4.6.6.1 Labeling 110
4.6.7 Drinking Fountains 110
4.6.7.1 Provided 110
4.6.7.1.1 Alternative 110
4.6.7.1.2 Common Use Area 110
4.6.7.2 Readily Accessible 111
4.6.7.2.1 Not Located 111
4.6.7.3 Single Fountain 111
4.6.7.4 Angle Jet Type 111
4.6.7.5 Potable Water Supply 111
4.6.7.6 Wastewater 111

4.6.8 Garbage Receptacles 111
4.6.8.1 Sufficient Number 111
4.6.8.2 Number and Location 111
4.6.8.3 Closable 111
4.6.9 Food and Drink Concessions 111
4.6.9.1 Meet AHJ Requirements 111
4.6.10 Spectator Areas 112
4.6.10.1 Within Aquatic Facility Enclosure 112
4.6.10.2 Deck 112
4.6.10.2.1 Additional Width 112
4.6.10.3 Balcony 112
4.6.10.4 Bleachers 112
4.7 Recirculation and Water Treatment 113
4.7.1 Recirculation Systems and Equipment 113
4.7.1.1 General 113
4.7.1.1.1 Equipped and Operated 113
4.7.1.1.2 Component Installation 113
4.7.1.1.3 Recirculation System 113
4.7.1.2 Combined Aquatic Venue Treatment 113
4.7.1.2.1 Maintain and Measure 113
4.7.1.2.2 Secondary Disinfection 113
4.7.1.2.3 Isolate 113
4.7.1.3 Inlets 113
4.7.1.3.1 General 113
4.7.1.3.2 Floor Inlets 114
4.7.1.3.3 Wall Inlets 114
4.7.1.4 Perimeter Overflow Systems/Gutters 115
4.7.1.4.1 General 115
4.7.1.4.2 Perimeter Overflow System Size and Shape 116
4.7.1.4.3 Gutter Outlets 116
4.7.1.4.4 Surge Tank Capacity 116
4.7.1.4.5 Tolerances 117
4.7.1.4.6 Makeup Water System 117
4.7.1.5 Skimmers and Alternative Gutter Technologies Using In-Pool Surge Capacity 117
4.7.1.5.1 General 117
4.7.1.5.2 Skimmer Location 118
4.7.1.5.3 Skimmer Flow Rate 118
4.7.1.5.4 Control 118
4.7.1.5.5 Tolerances 118
4.7.1.6 Submerged Suction Outlet 119
4.7.1.6.1 General 119
4.7.1.6.2 Number and Spacing 119
4.7.1.6.3 Tank Connection 119
4.7.1.6.4 Flow Distribution and Control 119
4.7.1.6.5 Flow Velocities 120
4.7.1.7 Piping 120

4.7.1.7.1 Design ... 120
4.7.1.7.2 Velocity in Pipes ... 120
4.7.1.7.3 Drainage and Installation ... 121
4.7.1.7.4 Piping and Component Identification ... 121
4.7.1.7.5 Testing ... 121
4.7.1.8 Strainers and Pumps ... 122
4.7.1.8.1 Strainers ... 122
4.7.1.8.2 Pumping Equipment ... 122
4.7.1.8.3 Operating Gauges ... 123
4.7.1.9 Flow Measurement and Control ... 123
4.7.1.9.1 Flow Meters ... 123
4.7.1.9.2 Valves ... 123
4.7.1.10 Flow Rates / Turnover Times ... 124
4.7.1.10.1 Maximum Allowable ... 124
4.7.1.10.2 Calculated ... 124
4.7.1.10.3 Turnover Variance ... 124
4.7.1.10.4 Turnover Times ... 124
4.7.1.10.5 Reuse Ratio ... 125
4.7.1.10.6 Flow Turndown System ... 125
4.7.2 Filtration ... 126
4.7.2.1 All Filters ... 126
4.7.2.1.1 Required ... 126
4.7.2.2 Granular Media Filters ... 126
4.7.2.2.1 General ... 126
4.7.2.2.2 Filter Location and Spacing ... 126
4.7.2.2.3 Filtration and Backwashing Rates ... 127
4.7.2.2.4 Minimum Filter Media Depth Requirements ... 127
4.7.2.2.5 Differential Pressure Measurement Gauges ... 127
4.7.2.2.6 Coagulant Injection Equipment Installation ... 127
4.7.2.3 Precoat Filters ... 127
4.7.2.3.1 General ... 127
4.7.2.3.2 Filtration Rates ... 128
4.7.2.3.3 Precoat Media Introduction System Process ... 128
4.7.2.3.4 Continuous Filter Media Feed Equipment ... 128
4.7.2.4 Cartridge Filters ... 129
4.7.2.4.1 Listed ... 129
4.7.2.4.2 Filtration Rates ... 129
4.7.2.4.3 Supplied and Sized ... 129
4.7.2.4.4 Spare Cartridge ... 129
4.7.3 Disinfection and pH Control ... 129
4.7.3.1 Chemical Addition Methods ... 129
4.7.3.1.1 Disinfection and pH ... 129
4.7.3.2 Feed Equipment ... 129
4.7.3.2.1 General ... 129
4.7.3.2.2 Sizing of Disinfection Equipment ... 130
4.7.3.2.3 Introduction of Chemicals ... 131
4.7.3.2.4 Compressed Chlorine Gas ... 131
4.7.3.2.5 Types of Feeders ... 131

4.7.3.2.6 Salt Electrolytic Chlorine Generators, Brine Electrolytic Chlorine, or Bromine Generators ... 132
4.7.3.2.7 Feeders for pH Adjustment ... 133
4.7.3.2.8 Automated Controllers ... 133
4.7.3.3 Secondary Disinfection Systems ... 134
4.7.3.3.1 General Requirements ... 134
4.7.3.3.2 3-log Inactivation and Oocyst Reduction ... 135
4.7.3.3.3 Ultraviolet Light Systems ... 136
4.7.3.3.4 Ozone Disinfection ... 138
4.7.3.4 Supplemental Treatment Systems ... 141
4.7.3.4.1 General Requirements ... 141
4.7.3.4.2 Ultraviolet Light ... 142
4.7.3.4.3 Ozone ... 142
4.7.3.4.4 Copper / Silver Ion Systems ... 142
4.7.3.4.5 Ultraviolet Light / Hydrogen Peroxide Systems ... 142
4.7.3.5 Water Quality Testing Devices and Kits ... 142
4.7.3.5.1 Compliance ... 142
4.7.3.5.2 Water Clarity Device ... 143
4.7.3.6 Microbiological Testing Equipment ... 143
4.7.3.6.1 EPA-Approved ... 143
4.7.4 Water Replenishment System ... 143
4.7.4.1 Discharge and Measure ... 143
4.7.4.1.1 Alternate System ... 143
4.7.4.1.2 Discharge ... 143
4.7.5 Spas ... 143
4.7.5.1 General ... 143
4.7.5.1.1 Requirements ... 143
4.7.5.2 Flow Rates/Turnover Times ... 144
4.7.5.2.1 Maximum Allowable ... 144
4.7.5.2.2 Turnover Time Variance ... 144
4.7.5.2.3 Turnover Times ... 144
4.7.5.3 Filtration System Inlets ... 144
4.7.5.4 Jet System Inlets ... 144
4.7.5.4.1 Air Flow ... 144
4.7.5.4.2 Skimmer ... 144

4.8 Decks and Equipment ... 144
4.8.1 Decks ... 144
4.8.1.1 General Standards for All Decks ... 144
4.8.1.1.1 Constructed ... 144
4.8.1.1.2 Lifeguard Placement and Safety Considerations ... 144
4.8.1.1.3 Joints or Gaps ... 145
4.8.1.1.4 Rounded Edges ... 145
4.8.1.1.5 Minimize Cracks ... 145
4.8.1.1.6 Concrete Decking ... 146
4.8.1.2 Standards for Perimeter Decks ... 146
4.8.1.2.1 Impervious ... 146
4.8.1.2.2 Watertight Expansion ... 146

4.8.1.2.3 Watertight Expansion ... 146
4.8.1.3 Drains ... 146
4.8.1.3.1 Slope ... 146
4.8.1.3.2 Placement ... 147
4.8.1.3.3 Cross Connection Control ... 147
4.8.1.3.4 Drain Bodies ... 148
4.8.1.3.5 Drain Covers ... 148
4.8.1.4 Materials / Slip Resistance ... 148
4.8.1.4.1 General ... 148
4.8.1.4.2 Slip Resistance ... 148
4.8.1.4.3 Carpet ... 148
4.8.1.4.4 Wood ... 148
4.8.1.4.5 Dry Deck ... 148
4.8.1.4.6 Landscaping ... 148
4.8.1.5 Deck Size / Width ... 148
4.8.1.5.1 Perimeter Deck ... 148
4.8.1.5.2 Fixed Equipment ... 149
4.8.1.5.3 Circulation Path ... 150
4.8.1.6 Wing Walls or Peninsulas ... 150
4.8.1.6.1 No Perimeter Deck ... 150
4.8.1.6.2 Perimeter Overflow System ... 150
4.8.1.6.3 Pool Perimeter ... 150
4.8.1.6.4 Normal Operating Water Level ... 151
4.8.1.6.5 Deck Drainage ... 151
4.8.1.6.6 Vertical Depth Markers ... 151
4.8.1.7 Islands ... 151
4.8.1.7.1 Minimum Width ... 151
4.8.1.7.2 Slip Resistant ... 151
4.8.1.7.3 Lifeguards ... 151
4.8.1.7.4 Vertical Depth Markers ... 151
4.8.1.7.5 Horizontal Depth Markers ... 151
4.8.1.7.6 Bridge or Stairway ... 151
4.8.1.7.7 Minimum Clearance ... 152
4.8.1.7.8 Guard Rails ... 152
4.8.1.8 Heated Decks ... 152
4.8.1.8.1 Freeze Protection ... 152
4.8.1.8.2 Clearly Delineated ... 152
4.8.1.9 Hose Bibbs ... 152
4.8.1.9.1 General ... 152
4.8.1.9.2 Backflow Prevention ... 152
4.8.2 Diving Boards and Platforms ... 152
4.8.2.1 Diving Envelope ... 152
4.8.2.1.1 Competitive Diving ... 152
4.8.2.1.2 Non-Competitive Diving ... 153
4.8.2.2 Steps and Guardrails ... 153
4.8.2.2.1 Higher than Twenty-One Inches ... 153
4.8.2.2.2 Self-Draining Treads ... 153
4.8.2.2.3 Short Platforms ... 153

4.8.2.2.4 Tall Platforms ... 153
4.8.3 Starting Platforms ... 155
4.8.3.1 Conform to Standard Codes ... 155
4.8.3.2 Minimum Water Depth ... 155
4.8.3.3 Leading Edge ... 155
4.8.3.4 Slip Resistant ... 155
4.8.3.5 Secure and Stable ... 156
4.8.4 Pool Slides [N/A] ... 156
4.8.5 Lifeguard- and Safety-Related Equipment ... 156
4.8.5.1 Equipment Inspection and Maintenance [N/A] ... 156
4.8.5.2 Safety Equipment Required at all Aquatic Facilities ... 156
4.8.5.2.1 Emergency Communication Equipment ... 156
4.8.5.3 Safety Equipment Required at Facilities with Lifeguards ... 156
4.8.5.3.1 Lifeguard Chair and Stand Placement ... 156
4.8.5.3.2 Lifeguard Chair and Stand Design ... 157
4.8.5.3.3 UV Protection for Chairs and Stands ... 157
4.8.6 Barriers and Enclosures ... 157
4.8.6.1 General Requirements ... 157
4.8.6.1.1 Enclosed ... 157
4.8.6.2 Construction Requirements ... 157
4.8.6.2.1 Discourage Climbing ... 157
4.8.6.2.2 Emergency Exit Paths ... 158
4.8.6.2.3 Windows ... 158
4.8.6.2.4 Height ... 158
4.8.6.3 Gates and Doors ... 158
4.8.6.3.1 Self-Closing and Latching ... 158
4.8.6.3.2 Gates ... 159
4.8.6.3.3 Turnstiles ... 159
4.8.6.3.4 Exit Gates ... 159
4.8.6.3.5 Swing Outward ... 159
4.8.6.3.6 Absence of Local Building Codes ... 159
4.8.6.3.7 Unguarded Pools ... 159
4.8.6.3.8 Other Aquatic Venues ... 159
4.8.6.4 Indoor Aquatic Venues ... 160
4.8.6.4.1 Enclosure ... 160
4.8.6.4.2 Securable ... 160
4.8.6.4.3 Indoor and Outdoor Aquatic Venues ... 160
4.8.6.4.4 Wall Separating ... 160
4.8.6.5 Multiple Aquatic Venues ... 160
4.8.6.5.1 One Enclosure ... 160
4.8.6.5.2 Wading Pools ... 160
4.8.7 Aquatic Venue Cleaning Systems ... 160
4.8.7.1 No Hazard ... 160
4.8.7.2 Common Cleaning Equipment ... 160
4.8.7.3 Integral Vacuum Systems ... 161
4.8.7.4 GFCI Power ... 161
4.8.7.5 Low Voltage ... 161
4.8.7.6 GFCI Connection ... 161

4.9 Filter/Equipment Room 161
4.9.1 Equipment Room 161
4.9.1.1 General Requirements 161
4.9.1.1.1 Nonabsorbent Material 161
4.9.1.1.2 Floor Slope 161
4.9.1.1.3 Opening 161
4.9.1.1.4 Hose Bibb 161
4.9.1.2 Construction 162
4.9.1.2.1 Size 162
4.9.1.2.2 Lighting 162
4.9.1.3 Electrical 162
4.9.1.3.1 Conform to NEC 162
4.9.1.3.2 Conform to NRTL 162
4.9.1.4 Ventilation 162
4.9.1.4.1 Code Conformance 162
4.9.1.5 Markings 162
4.9.1.5.1 Piping Identified 162
4.9.1.5.2 Piping Marked 163
4.9.1.5.3 Valves Identified 163
4.9.1.6 Equipment Rooms Containing Combustion Equipment [N/A] 163
4.9.1.7 Separation from Chemical Storage Spaces 163
4.9.1.7.1 Equipment 163
4.9.1.7.2 Doors and Openings 164
4.9.1.7.3 Indoor Aquatic Facility Access 165
4.9.1.8 Other Equipment Room Guidance 166
4.9.1.8.1 Access Space 166
4.9.1.8.2 Adequate Space 166
4.9.1.8.3 Minimize Hazards 166
4.9.1.8.4 Refrigeration Equipment 167
4.9.2 Chemical Storage Spaces 167
4.9.2.1 Outdoor / Indoor Storage 167
4.9.2.1.1 Stored Outdoors 167
4.9.2.1.2 Minimize Vapors 167
4.9.2.1.3 Dedicated Space 167
4.9.2.1.4 Eyewash 167
4.9.2.2 Construction 168
4.9.2.2.1 Foreseeable Hazards 168
4.9.2.2.2 Protected 168
4.9.2.2.3 Floor 168
4.9.2.2.4 Minimize Fumes 168
4.9.2.2.5 Surfaces 168
4.9.2.2.6 No Openings 168
4.9.2.3 Exterior Chemical Storage Spaces 168
4.9.2.3.1 Outdoor Equipment 168
4.9.2.3.2 Fencing 168
4.9.2.3.3 Gate 168
4.9.2.4 Chemical Storage Space Doors 169
4.9.2.4.1 Signage 169

4.9.2.4.2 Emergency Egress ... 169
4.9.2.4.3 Interior Door ... 169
4.9.2.4.4 Equipment Space ... 169
4.9.2.4.5 Interior Opening ... 169
4.9.2.5 Interior Chemical Storage Spaces ... 170
4.9.2.5.1 No Air Movement ... 170
4.9.2.5.2 Electrical Conduit System ... 171
4.9.2.6 Air Ducts in Interior Chemical Storage Spaces ... 172
4.9.2.6.1 No Air Movement ... 172
4.9.2.6.2 Chemical Storage ... 172
4.9.2.7 Pipes and Tubes in Interior Chemical Storage Spaces ... 172
4.9.2.7.1 Not Enter ... 172
4.9.2.7.2 Devices ... 173
4.9.2.7.3 Wall Penetrations ... 173
4.9.2.8 Combustion Equipment in Interior Chemical Storage Spaces ... 173
4.9.2.8.1 Installed ... 173
4.9.2.9 Electrical Equipment in Chemical Storage Spaces ... 173
4.9.2.10 Ozone Rooms ... 173
4.9.2.10.1 Only Ozone Equipment ... 173
4.9.2.10.2 Emergency Ventilation ... 173
4.9.2.10.3 Below Grade ... 174
4.9.2.10.4 Signage ... 174
4.9.2.10.5 Alarm System ... 174
4.9.2.11 Gaseous Chlorination Space ... 175
4.9.2.11.1 Existing Facilities ... 175
4.9.2.11.2 Adequate Size ... 175
4.9.2.11.3 Secure Tanks ... 175
4.9.2.11.4 Not Below Grade ... 175
4.9.2.11.5 Compressed-Chlorine Gas ... 175
4.9.2.11.6 Entry Door ... 175
4.9.2.11.7 Inspection Window ... 176
4.9.2.11.8 Ventilation ... 176
4.9.2.12 Windows in Chemical Storage Spaces ... 176
4.9.2.12.1 Not Required ... 176
4.9.2.12.2 Requirements ... 177
4.9.2.12.3 Exterior Window ... 177
4.9.2.13 Sealing and Blocking Materials ... 177
4.9.2.13.1 Minimize Leakage ... 177
4.9.2.13.2 Compatible ... 177
4.9.2.13.3 Fire Rating ... 177

4.10 Hygiene Facilities ... 177
4.10.1 General ... 177
4.10.1.1 Required at Adoption ... 177
4.10.1.2 Minimum to Provide ... 178
4.10.1.3 Construction ... 178
4.10.1.4 Minimum Toilets, Urinals, and Other Fixtures ... 178
4.10.1.5 Theoretical Peak Occupancy ... 178

4.10.2 Location ... 178
4.10.2.1 Distance ... 178
4.10.2.2 Children Less than Five Years of Age ... 178
4.10.3 Design and Construction ... 178
4.10.3.1 Floors ... 178
4.10.3.2 Floor Base ... 178
4.10.3.3 Floor Drains ... 179
4.10.3.3.1 Opening Grill Covers ... 179
4.10.3.3.2 Sloped to Drain ... 179
4.10.3.4 Partitions and Enclosures ... 179
4.10.3.5 Hose Bibb ... 179
4.10.4 Plumbing Fixture Requirements ... 179
4.10.4.1 General ... 179
4.10.4.1.1 Protected ... 179
4.10.4.1.2 Easily Cleaned ... 179
4.10.4.1.3 Toilet Counts ... 179
4.10.4.1.4 Hand Wash Sink ... 179
4.10.4.2 Cleansing Showers ... 180
4.10.4.2.1 Count ... 180
4.10.4.2.2 Distributed ... 180
4.10.4.2.3 Location ... 180
4.10.4.2.4 Enclosed ... 180
4.10.4.2.5 Soap Dispenser ... 180
4.10.4.2.6 Exemption ... 180
4.10.4.3 Rinse Showers ... 180
4.10.4.3.1 Minimum and Location ... 180
4.10.4.3.2 Temperature ... 180
4.10.4.3.3 Floor Sloped ... 181
4.10.4.3.4 Large Aquatic Facilities ... 181
4.10.4.3.5 Beach Entry ... 181
4.10.4.3.6 Lazy River ... 181
4.10.4.3.7 Waterslide ... 181
4.10.4.4 All Showers ... 181
4.10.4.5 Diaper-Changing Stations ... 181
4.10.4.5.1 Each Facility ... 181
4.10.4.5.2 Conform ... 182
4.10.4.5.3 Unisex ... 182
4.10.4.5.4 Trash Can ... 182
4.10.4.5.5 Disinfecting Surface ... 182
4.10.4.6 Non-Plumbing Fixture Requirements ... 182
4.10.4.6.1 Easy to Clean ... 182
4.10.4.6.2 Glass ... 182
4.10.4.6.3 Mirrors ... 182
4.10.4.6.4 Lockers ... 182
4.10.4.6.5 Soap Dispensers ... 182
4.10.4.6.6 Dryers / Paper Towels ... 183
4.10.4.6.7 Toilet Paper Dispensers ... 183
4.10.4.6.8 Female Facilities ... 183

4.10.4.6.9 Trash Can ... 183
4.10.5 Provision of Suits, Towels, and Shared Equipment ... 183
4.10.5.1 Adequate Space ... 183
4.10.6 Foot Baths ... 183
4.10.6.1 Prohibited ... 183
4.10.7 Sharps ... 183
4.10.7.1 Container ... 183

4.11 Water Supply/ Wastewater Disposal ... 184
4.11.1 Water Supply ... 184
4.11.1.1 Public Water System ... 184
4.11.1.1.1 Other Sources ... 184
4.11.1.1.2 Condensate / Reclaimed Water ... 184
4.11.1.2 Sufficient Capacity ... 184
4.11.1.2.1 Refill Pool ... 184
4.11.2 Fill Spout ... 184
4.11.2.1 Hazard ... 184
4.11.2.2 Shielded ... 184
4.11.2.3 Open End ... 184
4.11.2.4 Air Gap ... 185
4.11.3 Cross-Connection Control ... 185
4.11.3.1 Protected ... 185
4.11.4 Deck Drains and Rinse Showers ... 185
4.11.4.1 Sloped Walkway ... 185
4.11.4.2 Discharge ... 185
4.11.4.3 Area or Linear ... 185
4.11.4.4 Rinse Showers ... 185
4.11.5 Sanitary Wastes ... 185
4.11.5.1 Discharged ... 185
4.11.5.2 On-Site Sewer System ... 186
4.11.6 Pool Wastewater ... 186
4.11.6.1 Discharged ... 186
4.11.6.1.1 Storm Water Systems and Surface Waters ... 186
4.11.6.1.2 Recovery and Reuse ... 186
4.11.6.2 Ground Surface ... 186
4.11.6.3 Capacity ... 186
4.11.6.4 Separation Tank for Precoat Media Filters ... 186
4.11.6.4.1 Discharged ... 186

4.12 Specific Venues ... 186
4.12.1 Spas ... 186
4.12.1.1 Additional Provisions ... 186
4.12.1.2 Maximum Water Depth ... 187
4.12.1.2.1 Exercise Spas ... 187
4.12.1.2.2 Seating ... 187
4.12.1.3 Handholds ... 187
4.12.1.3.1 Options ... 187
4.12.1.4 Stairs ... 187
4.12.1.4.1 Handrail ... 187

4.12.1.4.2 Seating ... 187
4.12.1.4.3 Approach Steps ... 187
4.12.1.5 Perimeter Deck ... 187
4.12.1.5.1 Lower Ratio ... 187
4.12.1.5.2 Coping ... 188
4.12.1.5.3 Recessed ... 188
4.12.1.5.4 Elevated Spas ... 188
4.12.1.5.5 Minimum Distance ... 188
4.12.1.6 Depth Markers ... 188
4.12.1.7 Temperature ... 188
4.12.1.8 Drain ... 188
4.12.1.9 Air Induction System ... 188
4.12.1.9.1 Intake ... 188
4.12.1.10 Timers ... 188
4.12.1.11 Emergency Shutoff ... 188
4.12.2 Waterslides and Landing Pools ... 189
4.12.2.1 Design and Construction ... 189
4.12.2.1.1 Additional Provisions ... 189
4.12.2.1.2 Recognized Standards ... 189
4.12.2.2 Flumes ... 189
4.12.2.2.1 Surfaces ... 189
4.12.2.2.2 Dips ... 189
4.12.2.3 Flume Exits ... 189
4.12.2.3.1 Landing Pool ... 189
4.12.2.3.2 Intersection ... 190
4.12.2.4 Exit into Landing Pools ... 190
4.12.2.4.1 Water Level ... 190
4.12.2.4.2 Perpendicular ... 190
4.12.2.4.3 Exit System ... 190
4.12.2.4.4 Flume Exits ... 190
4.12.2.4.5 Point of Exit ... 190
4.12.2.5 Landing Pools ... 190
4.12.2.5.1 Steps ... 190
4.12.2.5.2 Landing Area ... 190
4.12.2.6 Decks ... 190
4.12.2.7 Means of Access ... 191
4.12.2.8 Slide Runouts ... 191
4.12.2.8.1 Egress ... 191
4.12.2.8.2 Designed ... 191
4.12.2.9 Drop Slides ... 191
4.12.2.9.1 Landing Area ... 191
4.12.2.9.2 Area Clearance ... 191
4.12.2.9.3 Steps ... 191
4.12.2.9.4 Water Depth ... 191
4.12.2.9.5 Manufacturer's Recommendation ... 191
4.12.2.10 Pool Slides ... 191
4.12.2.10.1 Designed for Safety ... 191
4.12.2.10.2 Non-Toxic ... 191

4.12.2.10.3 Water Depth ... 192
4.12.2.10.4 Pool Edge ... 192
4.12.2.11 Signage ... 192
4.12.3 Wave Pools ... 192
4.12.3.1 General ... 192
4.12.3.1.1 Additional Provisions ... 192
4.12.3.2 Access ... 192
4.12.3.2.1 Access Point ... 192
4.12.3.2.2 Perimeter Decks ... 193
4.12.3.2.3 Handholds ... 193
4.12.3.2.4 Steps and Handrails ... 193
4.12.3.2.5 Ladders ... 193
4.12.3.2.6 Float Line ... 193
4.12.3.3 Safety ... 194
4.12.3.3.1 Life Jackets ... 194
4.12.3.3.2 Shut-Off Switch ... 194
4.12.3.3.3 No Diving Sign ... 194
4.12.3.3.4 Caution Signs ... 194
4.12.4 Therapy Pools ... 194
4.12.4.1 Additional Provisions ... 194
4.12.4.2 Slope ... 194
4.12.4.2.1 Break Points ... 194
4.12.4.3 Hydrotherapy ... 194
4.12.4.4 Special Equipment ... 194
4.12.5 Lazy Rivers ... 195
4.12.5.1 General ... 195
4.12.5.1.1 Additional Provisions ... 195
4.12.5.1.2 Protrusions ... 195
4.12.5.2 Access and Egress ... 195
4.12.5.2.1 Means ... 195
4.12.5.2.2 Handhold ... 195
4.12.5.2.3 Deck ... 195
4.12.5.2.4 Bridges ... 195
4.12.6 Moveable Floors ... 195
4.12.6.1 General ... 195
4.12.6.1.1 Additional Provisions ... 195
4.12.6.1.2 Water Treatment ... 196
4.12.6.1.3 Underneath ... 196
4.12.6.2 Slip Resistance ... 196
4.12.6.3 Safety ... 196
4.12.6.3.1 Not Continuous ... 196
4.12.6.3.2 Underside ... 196
4.12.6.3.3 Entrapment ... 196
4.12.6.3.4 Hydraulic Fluid ... 196
4.12.6.4 Movement ... 196
4.12.6.4.1 Speed ... 196
4.12.6.4.2 Use ... 196
4.12.6.5 Water Depth and Markings ... 197

4.12.6.5.1 Displayed 197
4.12.6.5.2 Warning Markings 197
4.12.7 Bulkheads........ 197
4.12.7.1 Additional Provisions........ 197
4.12.7.2 Entrapment 197
4.12.7.3 Placement 197
4.12.7.4 Fixed........ 197
4.12.7.5 Gap 197
4.12.7.6 Handhold 197
4.12.7.7 Entrances and Exits 197
4.12.7.8 Guard Railings........ 197
4.12.7.9 Width........ 197
4.12.7.9.1 Starting Platforms 198
4.12.7.10 Bulkhead Travel 198
4.12.8 Interactive Water Play Venues 198
4.12.8.1 Additional Provisions........ 198
4.12.8.2 Surface........ 198
4.12.8.2.1 Manufactured Surfacing........ 198
4.12.8.3 Sloped........ 198
4.12.8.3.1 Adjacent Areas........ 198
4.12.8.3.2 Water Collection 198
4.12.8.4 Drains 198
4.12.8.4.1 Gravity 199
4.12.8.4.2 Direct Suction Outlets........ 199
4.12.8.5 Grate Openings........ 199
4.12.8.5.1 Tools 199
4.12.8.6 Treatment Tank 199
4.12.8.6.1 Completely Drain........ 199
4.12.8.6.2 Access Hatch........ 199
4.12.8.7 Deck Area........ 199
4.12.8.7.1 Deck Surface........ 199
4.12.8.7.2 Protected From Runoff 199
4.12.8.8 Barrier........ 199
4.12.8.9 Enclosures 200
4.12.8.10 Hazard 200
4.12.8.11 Maximum Velocity 200
4.12.8.12 Signage........ 200
4.12.8.13 NEC Requirements........ 200
4.12.9 Wading Pools........ 200
4.12.9.1 Additional Provisions........ 200
4.12.9.2 Barrier........ 200
4.12.9.2.1 Complete Enclosure........ 200
4.12.9.2.2 Shallow Water........ 200

5.0 Facility Operation and Maintenance........ 201

5.1 Operating Permits........ 201
5.1.1 Owner Responsibilities 201

5.1.1.1 Permit to Operate Required ... 201
5.1.1.2 Separate ... 201
5.1.1.3 Prior to Issuance ... 201
5.1.1.4 Permit Details ... 201
5.1.1.5 Permit Expiration ... 201
5.1.1.6 Permit Renewal ... 201
5.1.1.7 Permit Denial ... 201
5.1.1.8 Owner Responsibilities ... 202
5.1.2 Operating Permits ... 202
5.1.2.1 Permit Location ... 202
5.1.2.2 Operating Without a Permit ... 202
5.1.2.3 Required Closure ... 202

5.2 Inspections ... 202
5.2.1 Preoperational Inspections ... 202
5.2.1.1 Terms of Operation ... 202
5.2.2 Exemptions ... 202
5.2.2.1 Applying for Exemption ... 202
5.2.2.2 Change in Exemption Status ... 202
5.2.3 Variances ... 202
5.2.3.1 Variance Authority ... 202
5.2.3.2 Applying for a Variance ... 203
5.2.3.2.1 Application Components ... 203
5.2.3.3 Revoked ... 203
5.2.3.4 Not Transferable ... 203

5.3 Equipment Standards [N/A] ... 203

5.4 Aquatic Facility and Venue Operation and Maintenance ... 203
5.4.1 Closure and Reopening ... 203
5.4.1.1 Closure ... 203
5.4.1.1.1 Aquatic Venues With a Barrier ... 203
5.4.1.1.2 Aquatic Venues Without a Barrier but Open to the Public ... 204
5.4.1.1.3 Aquatic Venues Without a Barrier and Closed to the Public ... 204
5.4.1.2 Reopening ... 204
5.4.2 Preventive Maintenance Plan ... 204
5.4.2.1 Written Plan ... 204
5.4.2.1.1 Preventive Maintenance Plan Available ... 204
5.4.2.1.2 Contents ... 204
5.4.2.2 Facility Documentation ... 204
5.4.2.2.1 Original Plans and Specifications Available ... 204
5.4.2.2.2 Equipment Inventory ... 204
5.4.2.2.3 Inventory Details ... 205
5.4.2.2.4 Equipment Manuals ... 205
5.4.3 General Operations [N/A] ... 205

5.5 Aquatic Venue Structure ... 205
5.5.1 Shape [N/A] ... 205
5.5.2 Access Ladders [N/A] ... 205
5.5.3 Color and Finish [N/A] ... 205

5.5.4 Walls [N/A] ... 205
5.5.5 Depth Markings ... 205
5.5.5.1 Depth Markers ... 205
5.5.5.2 No Diving Markers ... 205
5.5.6 Pool Shell Maintenance ... 206
5.5.6.1 Cracking ... 206
5.5.6.1.1 Repaired ... 206
5.5.6.1.2 Document Cracks ... 206
5.5.6.1.3 Sharp Edges ... 206

5.6 Indoor / Outdoor Environment ... 206
5.6.1 Lighting ... 206
5.6.1.1 Lighting Maintained ... 206
5.6.1.1.1 Light Levels ... 206
5.6.1.1.2 Main Drain Visible ... 206
5.6.1.1.3 Underwater Lighting ... 206
5.6.1.1.4 Cracked Lenses ... 206
5.6.1.1.5 Intact Lenses ... 206
5.6.1.2 Glare ... 207
5.6.1.2.1 Assessments ... 207
5.6.1.2.2 Reduction ... 207
5.6.1.3 Night Swimming ... 207
5.6.1.3.1 Hours ... 207
5.6.1.4 Emergency Lighting ... 207
5.6.2 Indoor Aquatic Facility Ventilation ... 207
5.6.2.1 Purpose ... 207
5.6.2.2 Original Characteristics ... 207
5.6.2.3 Indoor Facility Areas ... 208
5.6.2.4 Ventilation Procedures ... 208
5.6.2.4.1 System Operation ... 208
5.6.2.5 Manuals/Commissioning Reports ... 208
5.6.2.6 Ventilation Monitoring ... 208
5.6.2.7 Air Filter Changing ... 208
5.6.2.8 Combined Chlorine Reduction ... 209
5.6.2.9 Building Purge Plan ... 209
5.6.2.10 Records ... 209
5.6.3 Electrical ... 209
5.6.3.1 Electrical Repairs ... 209
5.6.3.1.1 Local Codes ... 209
5.6.3.1.2 Immediately Repaired ... 209
5.6.3.1.3 Wiring ... 209
5.6.3.2 Electrical Receptacles ... 209
5.6.3.2.1 New Receptacles ... 209
5.6.3.2.2 Repairs ... 210
5.6.3.2.3 Replacement ... 210
5.6.3.2.4 Substitutions ... 210
5.6.3.3 Ground-Fault Circuit Interrupter ... 210
5.6.3.3.1 Manufacturer's Recommendations ... 210

5.6.3.3.2 Permanent Facilities ... 210
5.6.3.3.3 Testing ... 210
5.6.3.4 Grounding ... 210
5.6.3.4.1 Maintenance and Repair ... 210
5.6.3.4.2 Grounding Conductors ... 210
5.6.3.4.3 Damaged Conductors ... 210
5.6.3.4.4 Damaged Conductor Repair ... 211
5.6.3.4.5 Public Access ... 211
5.6.3.4.6 Venue Closure ... 211
5.6.3.5 Bonding ... 211
5.6.3.5.1 Local Codes ... 211
5.6.3.5.2 Bonding Conductors ... 211
5.6.3.5.3 Disconnected Conductors ... 211
5.6.3.5.4 Removable Covers ... 211
5.6.3.5.5 Scheduled Maintenance ... 211
5.6.3.5.6 Corrosion ... 211
5.6.3.5.7 Continuity ... 212
5.6.3.6 Extension Cords ... 212
5.6.3.6.1 Temporary Cords and Connectors ... 212
5.6.3.6.2 Minimum Distance from Water ... 212
5.6.3.6.3 Exception ... 212
5.6.3.6.4 GFCI Protection ... 212
5.6.3.6.5 Local Code ... 212
5.6.3.6.6 Compliance ... 212
5.6.3.7 Portable Electric Devices ... 212
5.6.3.8 Communication Devices and Dispatch Systems ... 212
5.6.4 Facility Heating ... 213
5.6.4.1 Facility Heating ... 213
5.6.4.1.1 Maintenance and Repair ... 213
5.6.4.1.2 Defects ... 213
5.6.4.1.3 Temperature ... 213
5.6.4.1.4 Combustion Device ... 213
5.6.4.2 Water Heating ... 213
5.6.5 First Aid Room [N/A] ... 213
5.6.6 Emergency Exit ... 213
5.6.6.1 Exit Routes ... 213
5.6.7 Plumbing ... 213
5.6.7.1 Water Supply ... 213
5.6.7.1.1 Water Pressure ... 213
5.6.7.1.2 Availability ... 213
5.6.7.1.3 Cross-Connection Control ... 214
5.6.7.2 Drinking Fountains ... 214
5.6.7.2.1 Good Repair ... 214
5.6.7.2.2 Clean ... 214
5.6.7.2.3 Catch Basin ... 214
5.6.7.2.4 Contamination ... 214
5.6.7.2.5 Water Pressure ... 214
5.6.7.3 Waste Water ... 214

5.6.7.3.1 Waste Water Disposal ... 214
5.6.7.3.2 Drainage ... 214
5.6.7.3.3 Drain Line ... 214
5.6.7.3.4 No Standing Water ... 214
5.6.7.4 Water Replenishment ... 215
5.6.7.4.1 Volume ... 215
5.6.7.4.2 Discharged ... 215
5.6.7.4.3 Multi-System Facilities ... 215
5.6.8 Solid Waste ... 215
5.6.8.1 Storage Receptacles ... 215
5.6.8.1.1 Good Repair and Clean ... 215
5.6.8.1.2 Areas ... 215
5.6.8.2 Disposal ... 215
5.6.8.2.1 Frequency ... 215
5.6.8.2.2 Local Code Compliance ... 215
5.6.9 Decks ... 216
5.6.9.1 Food Preparation and Consumption ... 216
5.6.9.1.1 Preparation ... 216
5.6.9.1.2 Eating and Drinking ... 216
5.6.9.2 Glass ... 216
5.6.9.2.1 Containers ... 216
5.6.9.2.2 Furniture ... 216
5.6.9.3 Deck Maintenance ... 216
5.6.9.3.1 Free From Obstructions ... 216
5.6.9.3.2 Diaper Changing ... 216
5.6.9.3.3 Vermin ... 216
5.6.9.3.4 Original Design ... 216
5.6.9.3.5 Standing Water ... 217
5.6.9.3.6 Drains ... 217
5.6.9.3.7 Wet Areas ... 217
5.6.9.3.8 Circulation Path ... 217
5.6.10 Aquatic Facility Maintenance ... 217
5.6.10.1 Diving Boards and Platforms ... 217
5.6.10.1.1 Slip-Resistant Finish ... 217
5.6.10.1.2 Loose Bolts and Cracked Boards ... 217
5.6.10.2 Steps and Guardrails ... 217
5.6.10.2.1 Immovable ... 217
5.6.10.2.2 Maintenance ... 217
5.6.10.3 Starting Platforms ... 218
5.6.10.4 Waterslides ... 218
5.6.10.4.1 Maintenance ... 218
5.6.10.4.2 Slime and Biofilm ... 218
5.6.10.4.3 Flow Rates ... 218
5.6.10.4.4 Disinfectant ... 218
5.6.10.5 Fencing and Barriers ... 218
5.6.10.5.1 Maintenance ... 218
5.6.10.5.2 Tested Daily ... 218
5.6.10.6 Aquatic Facility Cleaning ... 218

5.6.10.6.1 Cleaning 218
5.6.10.6.2 Vacuuming 218
5.6.10.6.3 Port Openings 219

5.7 Recirculation and Water Treatment 219
5.7.1 Recirculation Systems and Equipment 219
5.7.1.1 General 219
5.7.1.1.1 Continuous Operation 219
5.7.1.1.2 Flow 219
5.7.1.1.3 Gutter / Skimmer Pools 219
5.7.1.2 Combined Venue Treatment 220
5.7.1.3 Inlets 220
5.7.1.4 Surface Skimming Devices 220
5.7.1.4.1 Perimeter Overflow 220
5.7.1.4.2 Automatic Fill System 220
5.7.1.4.3 Skimmer Water Levels 220
5.7.1.4.4 Flow 220
5.7.1.4.5 Strainer Baskets 220
5.7.1.4.6 Weirs 220
5.7.1.4.7 Flotation Test 220
5.7.1.5 Submerged Drains/Suction Outlet Covers or Gratings 221
5.7.1.5.1 Replaced 221
5.7.1.5.2 Documentation 221
5.7.1.6 Piping [N/A] 221
5.7.1.7 Strainers & Pumps 221
5.7.1.8 Flow Meters 221
5.7.1.9 Flow Rates / Turnovers 221
5.7.1.9.1 New Construction or Substantially Altered Venues 221
5.7.1.9.2 Construction Before Adoption of this Code 221
5.7.2 Filtration 221
5.7.2.1 Granular Media Filters 222
5.7.2.1.1 Filtration Rates 222
5.7.2.1.2 Backwashing Rates 222
5.7.2.1.3 Clear Water 222
5.7.2.1.4 Backwashing Frequency 222
5.7.2.1.5 Filter Media Inspections 222
5.7.2.1.6 Vacuum Sand Filters 223
5.7.2.1.7 Filtration Enhancing Products 223
5.7.2.2 Precoat Filters 223
5.7.2.2.1 Appropriate 223
5.7.2.2.2 Return to the Pool 223
5.7.2.2.3 Operation 223
5.7.2.2.4 Cleaning 224
5.7.2.2.5 Continuous Feed Equipment 224
5.7.2.2.6 Bumping 224
5.7.2.2.7 Filter Media 224
5.7.2.3 Cartridge Filters 224
5.7.2.3.1 NSF Standards 224

5.7.2.3.2 Filtration Rates ... 224
5.7.2.3.3 Filter Elements ... 225
5.7.2.3.4 Spare Cartridge ... 226
5.7.3 Disinfection and pH Control ... 226
5.7.3.1 Primary Disinfectants ... 226
5.7.3.1.1 Chlorine (Hypochlorites) ... 226
5.7.3.1.2 Bromine ... 227
5.7.3.1.3 Stabilizers ... 227
5.7.3.1.4 Compressed Chlorine Gas ... 228
5.7.3.1.5 Salt Electrolytic Chlorine Generators, Brine Electrolytic Chlorine or Bromine Generators ... 228
5.7.3.2 Secondary or Supplemental Treatment Systems ... 229
5.7.3.2.1 Ultraviolet Light ... 229
5.7.3.2.2 Ozone ... 229
5.7.3.2.3 Copper / Silver Ions ... 230
5.7.3.3 Other Sanitizers, Disinfectants, or Chemicals ... 230
5.7.3.3.1 Chlorine Dioxide ... 230
5.7.3.3.2 Clarifiers, Flocculants, Defoamers ... 230
5.7.3.4 pH ... 231
5.7.3.4.1 pH levels ... 231
5.7.3.4.2 Approved Substances ... 231
5.7.3.5 Feed Equipment ... 231
5.7.3.5.1 Acceptable Chemical Delivery ... 231
5.7.3.5.2 Chemical Feeders ... 231
5.7.3.5.3 Dry Chemical Feeders ... 231
5.7.3.5.4 Venturi Inlet ... 232
5.7.3.5.5 Erosion Feeders ... 232
5.7.3.5.6 Liquid Solution Feeders ... 232
5.7.3.5.7 Checked Daily ... 232
5.7.3.5.8 Gas Feed Systems ... 232
5.7.3.5.9 Carbon Dioxide ... 232
5.7.3.6 Testing for Water Circulation and Quality ... 233
5.7.3.6.1 Available ... 233
5.7.3.6.2 Store ... 233
5.7.3.6.3 Temperature ... 233
5.7.3.6.4 Calibration ... 233
5.7.3.7 Automated Controllers and Equipment Monitoring ... 233
5.7.3.7.1 Use of Controller ... 233
5.7.3.7.2 Sampling ... 234
5.7.3.7.3 Monitor ... 234
5.7.3.7.4 Activities ... 234
5.7.3.7.5 Replacement Parts ... 234
5.7.3.7.6 Calibration ... 234
5.7.3.7.7 Ozone System ... 234
5.7.3.7.8 UV Systems ... 235
5.7.3.7.9 UV Alarm Testing and Maintenance ... 236
5.7.4 Water Sample Collection and Testing ... 236
5.7.4.1 Sample Collection ... 236

5.7.4.1.1 Same Volume ... 236
5.7.4.1.2 No Port ... 236
5.7.4.2 Routine Samples ... 236
5.7.4.2.1 Midday Collection ... 236
5.7.4.2.2 Compared ... 236
5.7.4.3 Bulk Water Sample ... 236
5.7.4.3.1 Obtain Sample ... 236
5.7.4.3.2 Rotate ... 237
5.7.4.3.3 Deepest Area ... 237
5.7.4.4 Aquatic Venue Water Chemical Balance ... 237
5.7.4.4.1 Total Alkalinity Levels ... 237
5.7.4.4.2 Combined Chlorine (Chloramines) ... 237
5.7.4.4.3 Calcium Hardness ... 237
5.7.4.4.4 Algaecides ... 237
5.7.4.5 Source (Fill) Water ... 237
5.7.4.6 Water Balance for Aquatic Venues ... 237
5.7.4.7 Water Temperature ... 238
5.7.4.7.1 Minimize Risk and Protect Safety ... 238
5.7.4.7.2 Maximum Temperature ... 238
5.7.5 Water Quality Chemical Testing Frequency ... 238
5.7.5.1 Chemical Levels ... 238
5.7.5.2 Manual Disinfectant Feed System ... 238
5.7.5.3 Automatic Disinfectant Feed System ... 238
5.7.5.4 In-Line ORP Readings ... 238
5.7.5.5 Total Alkalinity ... 238
5.7.5.6 Calcium Hardness ... 238
5.7.5.7 Cyanuric Acid ... 238
5.7.5.8 Saturation Index ... 238
5.7.5.8.1 Tested ... 238
5.7.5.8.2 Stabilized Chlorine ... 239
5.7.5.9 Total Dissolved Solids ... 239
5.7.5.10 Water Temperature ... 239
5.7.5.11 Salt ... 239
5.7.5.12 Copper/Silver Systems ... 239
5.7.6 Water Clarity ... 239
5.7.6.1 Water Clarity ... 239
5.7.6.1.1 Observation ... 239
5.7.6.1.2 Pools Over Ten Feet Deep ... 239
5.7.6.1.3 No Marker Tile ... 239
5.7.6.2 Visible ... 239
5.7.6.2.1 Spas ... 240

5.8 Decks and Equipment ... 240
5.8.1 Spectator Areas ... 240
5.8.1.1 Cross-Connection Control ... 240
5.8.1.1.1 Deck Drains ... 240
5.8.1.2 Materials / Slip Resistance ... 240
5.8.1.2.1 Clean and Good Repair ... 240

5.8.1.2.2 Risk Management ... 240
5.8.1.2.3 Tripping Hazards ... 240
5.8.1.3 Deck Size/Width ... 240
5.8.2 Diving Boards and Platforms [N/A] ... 240
5.8.3 Starting Blocks ... 240
5.8.3.1 Competitive Training and Competition ... 240
5.8.3.1.1 Supervision ... 240
5.8.3.1.2 Removed or Restricted ... 241
5.8.4 Pool Slides [N/A] ... 241
5.8.5 Lifeguard-and Safety Related Equipment ... 241
5.8.5.1 Equipment Inspection and Maintenance ... 241
5.8.5.2 Safety Equipment Required at All Aquatic Facilities ... 241
5.8.5.2.1 Emergency Communication Equipment ... 241
5.8.5.2.2 First Aid Equipment ... 241
5.8.5.2.3 Signage ... 242
5.8.5.3 Safety Equipment Required at Facilities with Lifeguards ... 242
5.8.5.3.1 UV Protection for Chairs and Stands ... 242
5.8.5.3.2 Spinal Injury Board ... 243
5.8.5.3.3 Rescue Tube Immediately Available ... 243
5.8.5.3.4 Rescue Tube on Person ... 243
5.8.5.3.5 Identifying Uniform ... 243
5.8.5.3.6 Signal Device ... 243
5.8.5.3.7 Sun Blocking Methods ... 243
5.8.5.3.8 Polarized Sunglasses ... 243
5.8.5.3.9 Personal Protective Equipment ... 243
5.8.5.3.10 Rescue Throwing Device ... 244
5.8.5.3.11 Reaching Pole ... 244
5.8.5.4 Safety Equipment and Signage Required at Facilities without Lifeguards ... 244
5.8.5.4.1 Throwing Device ... 244
5.8.5.4.2 Reaching Pole ... 244
5.8.5.4.3 CPR Posters ... 244
5.8.5.4.4 Imminent Hazard Sign ... 244
5.8.5.4.5 Additional Signage ... 245
5.8.6 Barriers and Enclosures ... 245
5.8.6.1 General Requirements ... 245
5.8.6.2 Construction Requirements (N/A) ... 245
5.8.6.3 Gates and Doors ... 245
5.8.6.3.1 Self-Closing and Latching ... 245

5.9 Filter/Equipment Room ... 245
5.9.1 Chemical Storage ... 245
5.9.1.1 Local Codes ... 245
5.9.1.2 OSHA and EPA ... 245
5.9.1.3 Safety Data Sheets ... 245
5.9.1.4 Access Prevention ... 246
5.9.1.5 Protected ... 246
5.9.1.6 No Mixing ... 246
5.9.1.6.1 SDS Consulted ... 246

5.9.1.7 Ignition Sources ... 246
5.9.1.8 Smoking ... 246
5.9.1.9 Lighting ... 246
5.9.1.10 PPE ... 246
5.9.1.11 Storage ... 246
5.9.1.12 Single Container ... 246
5.9.1.13 Separate ... 246
5.9.1.13.1 Waiver ... 247
5.9.1.14 Warning Signs ... 247
5.9.2 Chemical Handling ... 247
5.9.2.1 Identity ... 247
5.9.2.1.1 Labeling ... 247
5.9.2.2 NSF Standard ... 247
5.9.2.3 Measuring Devices ... 247
5.9.2.3.1 Clean and Dry ... 247
5.9.2.4 Chemical Addition Methods ... 247
5.9.2.4.1 Automatically Introduced ... 247
5.9.2.4.2 Safety Requirements ... 248
5.10 Hygiene Facilities ... 248
5.10.1 General [N/A] ... 248
5.10.2 Location [N/A] ... 248
5.10.3 Bathhouse Design [N/A] ... 248
5.10.4 Plumbing Fixture Requirements ... 248
5.10.4.1 General Requirements ... 248
5.10.4.1.1 Cleaned and Sanitized ... 248
5.10.4.1.2 Mold and Mildew ... 248
5.10.4.1.3 Hand Wash Station ... 249
5.10.4.2 Cleansing Showers ... 249
5.10.4.2.1 Cleaned and Sanitized ... 249
5.10.4.3 Rinse Showers ... 249
5.10.4.3.1 Cleaned ... 249
5.10.4.3.2 Easy Access ... 249
5.10.4.3.3 Not Blocked ... 249
5.10.4.3.4 No Soap ... 249
5.10.4.4 All Showers [N/A] ... 249
5.10.4.5 Diaper-Changing Stations ... 249
5.10.4.5.1 Hand Wash Sink Installed and Operational ... 249
5.10.4.5.2 Cleaned ... 249
5.10.4.5.3 Disinfectant ... 250
5.10.4.5.4 Portable Hand Wash Station ... 250
5.10.4.6 Non-Plumbing Fixture Requirements ... 250
5.10.4.6.1 Paper Towels ... 250
5.10.4.6.2 Soap ... 250
5.10.4.6.3 Trash ... 250
5.10.4.6.4 Floor Coverings ... 250
5.10.4.7 Sharps ... 251
5.10.4.7.1 Biohazard Action Plan ... 251

5.10.4.7.2 Disposed 251
5.10.5 Provision of Suits, Towels, and Shared Equipment 251
5.10.5.1 Towels 251
5.10.5.1.1 Suits 251
5.10.5.2 Receptacles 251
5.10.5.3 Shared Equipment Cleaned and Sanitized 251
5.10.5.4 Other Equipment 251
5.10.5.5 Good Repair 251
5.10.5.6 Used Equipment 252
5.10.5.6.1 Receptacles 252

5.11 Water Supply / Wastewater Disposal [N/A] 252

5.12 Special Requirements for Specific Venues 252
5.12.1 Waterslides 252
5.12.1.1 Signage 252
5.12.2 Wave Pools 252
5.12.2.1 Life Jackets 252
5.12.3 Moveable Floors 252
5.12.3.1 Starting Platforms 252
5.12.3.2 Diving Boards 252
5.12.4 Bulkheads 252
5.12.4.1 Open Area 252
5.12.4.2 Bulkhead Travel 252
5.12.5 Interactive Water Play Aquatic Venues 253
5.12.5.1 Cracks 253
5.12.5.2 Cleaning 253
5.12.5.2.1 No Sanitary Sewer Drain Available 253
5.12.6 Wading Pools 253
5.12.7 Spas 253
5.12.7.1 Required Operation Time 253
5.12.7.2 Drainage and Replacement 253
5.12.7.2.1 Calculated 253
5.12.7.3 Scrubbed 253

6.0 Policies and Management 254

6.0.1 Staff Training 254
6.0.1.1 Storage and Handling 254
6.0.1.2 PPE Procedures 254
6.0.1.3 Spill Procedures 254
6.0.1.4 OSHA Requirements 254
6.0.1.5 Chemical and SDS Lists 254
6.0.1.6 Training Plan 254
6.0.1.6.1 Training Topics 255
6.0.1.7 Training Records 255
6.0.1.8 Body Fluid Exposure 255
6.0.1.9 Exposure Control Program 255
6.0.1.10 PPE Provided and Disposed 255

6.1 Qualified Operator Training 255
6.1.1 Qualified Operator Qualifications and Certification 255
6.1.1.1 Qualifications 255
6.1.1.2 Training Documentation 255
6.1.1.2.1 Certificate Available 255
6.1.1.2.2 Originals 256
6.1.2 Essential Topics in Qualified Operator Training Courses 256
6.1.2.1 Course Content 256
6.1.2.1.1 Water Disinfection 256
6.1.2.1.2 Water Chemistry 259
6.1.2.1.3 Mechanical Systems 262
6.1.2.1.4 Health and Safety 265
6.1.2.1.5 Operations 268
6.1.3 General Requirements for Operator Training Courses 272
6.1.3.1 Course Providers 272
6.1.3.1.1 Recognized Courses 272
6.1.3.1.2 Providers 272
6.1.3.2 Course Content 272
6.1.3.3 Course Length 272
6.1.3.4 Instructor Requirements 272
6.1.3.5 Final Exam 273
6.1.3.5.1 Final Exam Administration 273
6.1.3.6 Course Certificates 273
6.1.3.7 Continuing Education [N/A] 273
6.1.3.8 Certificate Renewal 273
6.1.3.9 Certificate Suspension and Revocation 274
6.1.3.9.1 Evidence of Health Hazard 274
6.1.3.9.2 Evidence of Cheating 274
6.1.3.10 Additional Training or Testing 274
6.1.3.11 Certificate Recognition 274
6.1.3.12 Course Recognition 274
6.1.3.13 Length of Certificate Validity 274

6.2 Lifeguard Training 274
6.2.1 Lifeguard Qualifications 274
6.2.1.1 Course Content 275
6.2.1.1.1 Hazard Identification and Injury Prevention 275
6.2.1.1.2 Emergency Response Skill Set 275
6.2.1.1.3 Resuscitation Skills 275
6.2.1.1.4 First Aid 276
6.2.1.1.5 Legal Issues 276
6.2.1.2 Lifeguard Training Delivery 276
6.2.1.2.1 Standardized and Comprehensive 276
6.2.1.2.2 Skills Practice 276
6.2.1.2.3 Shallow Water Training 276
6.2.1.2.4 Deep Water Training 277
6.2.1.2.5 Sufficient Time 277
6.2.1.2.6 Certified Instructors 277

6.2.1.2.7 Training Equipment ... 277
6.2.1.3 Competency and Certification ... 278
6.2.1.3.1 Proficiency ... 278
6.2.1.3.2 Requirements ... 278
6.2.1.3.3 Instructor Physically Present ... 278
6.2.1.3.4 Certifications ... 278
6.2.1.3.5 Number of Years ... 278
6.2.1.3.6 Documentation ... 279
6.2.1.3.7 Expired Certificate ... 279
6.2.1.3.8 Certificate Suspension and Revocation ... 279
6.2.2 Aquatic Supervisor Training ... 280
6.2.2.1 Lifeguard Supervisor Candidate Prerequisites ... 280
6.2.2.2 Lifeguard Supervisor Training Elements ... 280
6.2.2.3 Lifeguard Supervisor Training Delivery ... 280
6.2.2.3.1 Standardized and Comprehensive ... 280
6.2.2.3.2 Sufficient Time ... 280
6.2.2.3.3 Course Setting ... 281
6.2.2.3.4 Lifeguard Supervisor Course Instructor Certification ... 281
6.2.2.4 Competency and Certification ... 282
6.2.2.4.1 Lifeguard Supervisor Proficiency ... 282
6.2.2.4.2 Lifeguard Supervisor Certifications ... 282
6.3 Facility Staffing ... 282
6.3.1 Qualified Operator Requirements and Availability ... 282
6.3.1.1 On-Site Qualified Operator Requirements ... 282
6.3.1.1.1 At Adoption ... 282
6.3.1.1.2 Size and Use ... 282
6.3.1.1.3 Bathers and Management ... 282
6.3.1.1.4 Compliance History ... 282
6.3.1.2 Contracted Off-site Qualified Operators ... 283
6.3.1.2.1 Visit Documentation ... 283
6.3.1.2.2 Documentation Details ... 283
6.3.1.2.3 Visit Corrective Actions ... 283
6.3.1.2.4 Onsite Responsible Supervisor ... 283
6.3.1.2.5 Onsite Responsible Supervisor Duties ... 283
6.3.2 Aquatic Facilities Requiring Qualified Lifeguards ... 284
6.3.2.1 List of Aquatic Facilities Requiring Qualified Lifeguards ... 284
6.3.3 Safety Plan ... 284
6.3.3.1 Code Compliance Staff Plan ... 284
6.3.3.1.1 Zone of Patron Surveillance ... 285
6.3.3.1.2 Rotation Procedures ... 285
6.3.3.1.3 Alternation of Tasks ... 285
6.3.3.1.4 Supervision Protocols ... 285
6.3.3.2 Emergency Action Plan ... 286
6.3.3.2.1 Coordination of Response ... 286
6.3.3.3 Pre-Service Requirements ... 286
6.3.3.3.1 Safety Team EAP Training ... 286
6.3.3.3.2 Safety Team Skills Proficiency ... 287

6.3.3.3.3 Qualified Lifeguard Emergency Action Plan Training ... 287
6.3.3.3.4 Qualified Lifeguard Skills Proficiency ... 287
6.3.3.3.5 CPR / AED and First Aid Certificate ... 287
6.3.3.3.6 Documentation of Pre-Service Training ... 288
6.3.3.4 In-Service Training ... 288
6.3.3.4.1 Documentation of In-Service Training ... 288
6.3.3.4.2 In-Service Documentation ... 288
6.3.3.4.3 In-Service Training Plan ... 288
6.3.3.4.4 Maintain Certificates ... 289
6.3.3.4.5 Competency Demonstration ... 289
6.3.3.5 AHJ Authority to Approve Safety Plan ... 289
6.3.3.5.1 Safety Plan on File ... 289
6.3.3.5.2 Safety Plan Implemented ... 289
6.3.4 Staff Management ... 289
6.3.4.1 Staff Provided Prior to Aquatic Venue Use ... 289
6.3.4.2 Safety Team Responsibilities ... 290
6.3.4.3 Lifeguard Staff ... 290
6.3.4.3.1 Minimum Number of Lifeguards ... 290
6.3.4.3.2 Lifeguard Responsibilities ... 290
6.3.4.3.3 Shallow Water Certified Lifeguards ... 290
6.3.4.3.4 Direct Surveillance ... 291
6.3.4.3.5 Distractions ... 291
6.3.4.4 Supervisor Staff ... 291
6.3.4.4.1 Lifeguard Supervisor Required ... 291
6.3.4.4.2 Designated Supervisor ... 291
6.3.4.4.3 Lifeguard Supervisor ... 291
6.3.4.5 Emergency Response and Communications Plans ... 291
6.3.4.5.1 Emergency Response and Communication Plan ... 291
6.3.4.5.2 Emergency Action Plan ... 292
6.3.4.5.3 Annual Review and Update ... 292
6.3.4.5.4 Available for Inspection ... 292
6.3.4.5.5 Training Documentation ... 292
6.3.4.5.6 Components ... 292
6.3.4.5.7 Facility Evacuation Plan ... 292
6.3.4.5.8 Communication Plan ... 293
6.3.4.5.9 Inclement Weather Plan ... 293
6.3.4.6 Remote Monitoring Systems ... 293
6.3.4.6.1 Lifeguard-Based ... 293
6.3.4.6.2 Operator-Based ... 294
6.3.4.6.3 Training ... 294
6.3.4.7 Employee Illness and Injury Policy ... 294
6.3.4.7.1 Illness Policy ... 294
6.3.4.7.2 Open Wounds ... 294

6.4 Facility Management ... 294
6.4.1 Operations ... 294
6.4.1.1 Operations Manual ... 294
6.4.1.1.1 Develop ... 294

6.4.1.1.2 Include 294
6.4.1.2 Operation Records 295
6.4.1.2.1 Record Maintenance 295
6.4.1.2.2 Additional Documentation 295
6.4.1.3 Safety and Maintenance Inspection and Recordkeeping 295
6.4.1.3.1 Daily Inspection Items 295
6.4.1.3.2 Other Inspection Items 296
6.4.1.4 Illness and Injury Incident Reports 296
6.4.1.4.1 Incidents to Record 296
6.4.1.4.2 Info to Include 296
6.4.1.4.3 Notify the AHJ 297
6.4.1.4.4 Lifeguard Rescues 297
6.4.1.5 Chemical Inventory Log 297
6.4.1.5.1 Expiration Dates 297
6.4.1.6 Daily Water Monitoring and Testing Records 297
6.4.1.7 Staff Certifications on File 298
6.4.1.7.1 Multiple Facilities 298
6.4.1.8 Bodily Fluids Remediation Log 298
6.4.1.8.1 Contamination Incidents 298
6.4.1.8.2 Standard Operating Procedures 298
6.4.1.8.3 Required Information 298
6.4.2 Patron-Related Management Aspects 299
6.4.2.1 Bather Count 299
6.4.2.1.1 User Guidelines 299
6.4.2.1.2 Maximum Occupancy 299
6.4.2.2 Signage 299
6.4.2.2.1 Facility Rules 299
6.4.2.2.2 Lettering 299
6.4.2.2.3 Sign Messages 299
6.4.2.2.4 Hygiene Facility Signage 301
6.4.2.2.5 Diaper-Changing Station Signage 301
6.4.2.3 Swimmer Empowerment Methods 301
6.4.2.3.1 Public Information and Health Messaging 301
6.4.2.3.2 Post Inspection Results 302

6.5 Fecal/Vomit/Blood Contamination Response 302
6.5.1 Contamination Response Plan 302
6.5.1.1 Contamination Response Plan 302
6.5.1.2 Contamination Training 302
6.5.1.2.1 Minimum 302
6.5.1.2.2 Informed 302
6.5.1.3 Equipment and Supply Verification 302
6.5.1.4 Plan Review 302
6.5.1.5 Plan Availability 302
6.5.2 Aquatic Venue Water Contamination Response 303
6.5.2.1 Closure 303
6.5.2.1.1 Closure Includes 303
6.5.2.2 Physical Removal 303

6.5.2.2.1 Clean / Disinfect Net or Scoop ... 303
6.5.2.2.2 No Vacuum Cleaners ... 303
6.5.2.3 Treated ... 303
6.5.3 Aquatic Venue Water Contamination Disinfection ... 304
6.5.3.1 Formed-Stool Contamination ... 304
6.5.3.1.1 Pools Containing Chlorine Stabilizers ... 304
6.5.3.1.2 Measurement of Inactivation Time ... 304
6.5.3.2 Diarrheal-Stool Contamination ... 304
6.5.3.2.1 Pools Containing Chlorine Stabilizers ... 304
6.5.3.3 Vomit-Contamination ... 305
6.5.3.3.1 Pools Containing Chlorine Stabilizers ... 305
6.5.3.3.2 Measurement of the Inactivation Time ... 305
6.5.3.4 Blood-Contamination ... 305
6.5.3.4.1 Operators Choose Treatment Method ... 305
6.5.3.5 Procedures for Brominated Pools ... 305
6.5.3.5.1 Bromine Residual ... 305
6.5.4 Surface Contamination Cleaning and Disinfection ... 305
6.5.4.1 Limit Access ... 305
6.5.4.2 Clean Surface ... 305
6.5.4.3 Contaminant Removal and Disposal ... 306
6.5.4.4 Disinfect Surface ... 306
6.5.4.5 Soak ... 306
6.5.4.6 Remove ... 306
6.6 AHJ Inspections ... 306
6.6.1 Inspection Process ... 306
6.6.1.1 Inspection Authority ... 306
6.6.1.2 Inspection Scope and Right ... 306
6.6.1.3 Based on Risk ... 306
6.6.1.4 Inspection Interference ... 307
6.6.2 Publication of Inspection Forms ... 307
6.6.2.1 Inspection Form Publication ... 307
6.6.3 Imminent Health Hazards ... 307
6.6.3.1 Violations Requiring Immediate Correction or Closure ... 307
6.6.3.1.1 Low pH Violations ... 308
6.6.3.1.2 High pH Violations ... 308
6.6.4 Enforcement ... 308
6.6.4.1 Placarding of Pool ... 308
6.6.4.2 Placard Location ... 308
6.6.4.2.1 State Authority ... 308
6.6.4.2.2 Tampering with Placard ... 308
6.6.4.3 Operator Follow-up ... 308
6.6.4.3.1 Correction of Violation ... 308
6.6.4.3.2 Hearing ... 308
6.6.4.4 Follow-up Inspection ... 308
6.6.4.4.1 Other Evidence of Correction ... 309
6.6.5 Enforcement Penalties ... 309
6.6.5.1 Liability and Jurisdiction ... 309

6.6.5.1.1 Failure to Comply ... 309
6.6.5.1.2 Civil Penalty ... 309
6.6.5.2 Continued Violation ... 309
6.6.5.3 Falsified Documents ... 309
6.6.5.4 Enforcement Process ... 309

The Model Aquatic Health Code

The Code

PREFACE

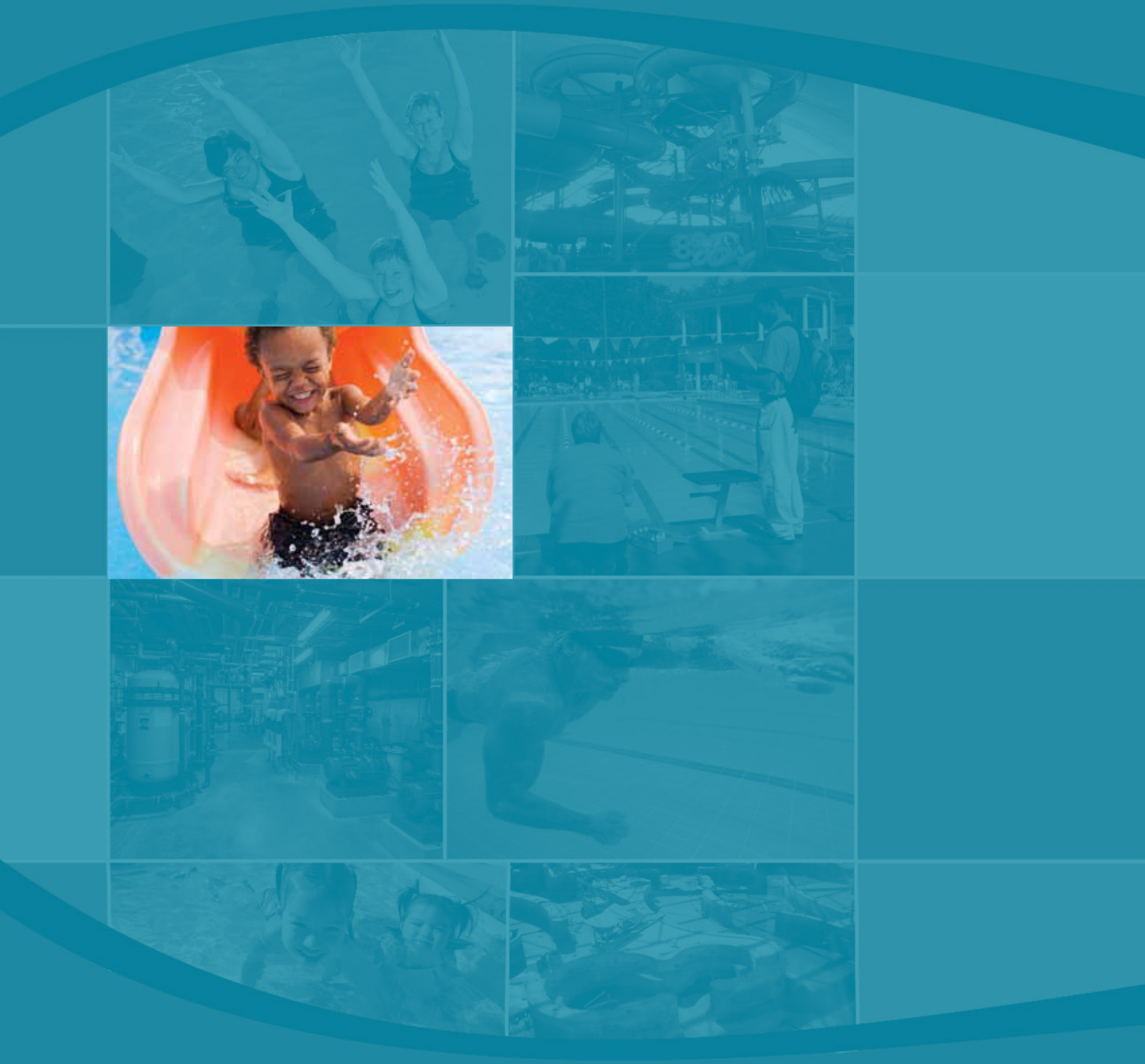

CS249779

1.0 Preface

1.1 Introduction

1.1.1 Rationale

In recent decades, public health practitioners have seen a dramatic increase in waterborne disease outbreaks associated with public disinfected AQUATIC FACILITIES *(e.g. swimming pools, water parks, etc.)*. As a result, public health investigations have revealed that many diseases can be prevented by proper maintenance and water treatment and by more modern disease prevention practices. Drowning and falling, diving, chemical use, and suction injuries continue to be major public health injuries associated with public AQUATIC FACILITIES, particularly for young children. In this context, the health and safety at public AQUATIC FACILITIES is regulated by state and local jurisdictions since, in the United States, there is no federal regulatory authority responsible for these public AQUATIC FACILITIES. All public pool codes are developed, reviewed, and approved by state and/or local public health officials or legislatures. Consequently, there is no uniform national guidance informing the design, construction, operation, and maintenance of public swimming pools and other public disinfected AQUATIC FACILITIES. As a result, the code requirements for preventing and responding to recreational water illnesses *(RWIs)* and injuries can vary significantly among local and state agencies. State and local jurisdictions spend a great deal of time, personnel, and resources creating and updating their individual codes on a periodic basic.

1.1.2 Need for Further Guidance

Based on illness tracking data, outbreak reporting, and stakeholder feedback, CDC believed further prevention-oriented planning and action were needed. CDC worked with the Council of State and Territorial Epidemiologists to get agreement on the need for a national workshop to develop guidance for preventing future RWI outbreaks. This CSTE position statement was passed in 2004 and CDC was tasked with organizing the national workshop, which was held in 2005.

1.1.3 Responsibility of User

This document does not address all safety or public health concerns associated with its use. It is the responsibility of the user of this document to establish appropriate health and safety practices and determine the applicability of regulatory limitations prior to each use.

1.1.4 Original Manufacturer Intent

In the absence of exceptions or further guidance, all fixtures and equipment shall be installed according to original manufacturer intent.

1.1.5 Local Jurisdiction

The MAHC refers to existing local codes in the jurisdiction for specific needs. In the absence of existing local codes, the authority having jurisdiction should specify an appropriate code reference.

1.2 Recreational Water-Associated Illness Outbreaks and Injuries

1.2.1 RWI Outbreaks

Large numbers of recreational water-related outbreaks are documented annually, which is a significant increase over the past several decades.

1.2.2 Significance of *Cryptosporidium*

Cryptosporidium causes a diarrheal disease spread from one person to another or, at aquatic venues, by ingestion of fecally-contaminated water. This pathogen is tolerant of CHLORINE and other halogen disinfectants. *Cryptosporidium* has emerged as the leading cause of pool-associated outbreaks in the United States.

1.2.3 Drowning and Injuries

Drowning and falling, diving, pool chemical use, and suction injuries continue to be major public health injuries associated with aquatic facilities. Drowning is a leading cause of injury death for young children and a leading cause of unintentional injury death for people of all ages.

1.2.4 Pool Chemical-Related Injuries

Pool chemical-related injuries occur regularly and can be prevented if pool chemicals are stored and used as recommended.

1.3 Model Aquatic Health Code

1.3.1 Background

All POOL CODES in the United States are reviewed and approved by state and/or local public health officials with no uniform national public health STANDARDS governing design, construction, operation, maintenance, policies, or management of public swimming pools and other public AQUATIC FACILITIES.

The effort to create the MAHC stems from a CDC-sponsored national workshop called "Recreational Water Illness Prevention at Disinfected Swimming Venues" that was convened on February 15-17, 2005, in Atlanta, Georgia. The workshop assembled persons from different disciplines working in state, local, and federal public health agencies, the aquatics sector, and academia to discuss ways to minimize the spread of recreational water illnesses at disinfected AQUATIC FACILITIES. The major recommendation from this workshop was that CDC lead a national partnership to create an open access model guidance document that helps local and state agencies incorporate science and best practices into their swimming POOL CODES and programs

without having to "recreate the wheel" each time they create or revise their POOL CODES. The attendees also recommended that this effort be all-encompassing so that it covered the spread of illness but also included drowning and injury prevention. Such an effort should increase the evidence base for AQUATIC FACILITY design, construction, operation, and maintenance while reducing the time, personnel, and resources needed to create and regularly update POOL CODES across the country.

Since 2007, CDC has been working with the public health sector, the aquatics sector, and academic representatives from across the United States to create this guidance document. Although, the initial workshop was responding to the significant increases in infectious disease outbreaks at AQUATIC FACILITIES, the MAHC is a complete AQUATIC FACILITY guidance document with the goal of reducing the spread of infectious disease and occurrence of drowning, injuries, and chemical exposures at public AQUATIC FACILITIES. Based on stakeholder feedback and recommendations, CDC agreed that public health improvements would be aided by development of an open access, comprehensive, systematic, collaboratively developed guidance document based on science and best practices covering AQUATIC FACILITY design and construction, operation and maintenance, and policies and management to address existing, emerging, and future public health threats.

1.3.2 MAHC Vision and Mission

The Model Aquatic Health Code's *(MAHC)* vision is "Healthy and Safe Aquatic Experiences for Everyone". The MAHC's mission is to incorporate science and best practices into guidance on how state and local officials can transform a typical health department pool program into a data-driven, knowledge-based, risk reduction effort to prevent disease and injuries and promote healthy recreational water experiences. The MAHC will provide local and state agencies with uniform guidelines and wording for the areas of design and construction, operation and maintenance, and policies and management of swimming POOLS, SPAS and other public disinfected AQUATIC FACILITIES.

1.3.3 Science and Best Practice

The availability of the MAHC should provide state and local agencies with the best available guidance for protecting public health using the latest science and best practices so they can use it to create or update their swimming POOL CODES.

1.3.4 Process

The MAHC development process created comprehensive consensus risk reduction guidance for AQUATIC FACILITIES based upon national interaction and discussion. The development plan encompassed design, construction, alteration, replacement, operation, and management of these facilities. The MAHC is driven by scientific data and best practices. It was developed by a process that included input from all sectors and levels of public health, the aquatics sector, academia, and the general public. It was open for two 60-day public comment periods during the process. It is national and comprehensive in scope and the guidance can be used to write or update POOL CODES across the U.S.

1.3.5 Open Access

The MAHC is an open access document that any interested individual, agency, or organization can freely copy, adapt, or fully incorporate MAHC wording into their aquatic facility oversight documents. As a federal agency, CDC does not copyright this material.

1.3.6 Updating the MAHC

The MAHC will be updated on a continuing basis through an inclusive, transparent, all-stakeholder process. This was a recommendation from the original national workshop and is essential to ensure that the MAHC stays current with the latest science, industry advances, and public health findings. To support this recommendation, CDC has supported creation of the Conference for the Model Aquatic Health Code (CMAHC; www.cmahc.org), a 501(c)(3) non-profit organization, to facilitate collecting, assessing, and relaying national input on needed MAHC revisions back to CDC for final consideration for acceptance.

1.3.7 Authority

Regulatory agencies like state and local governments have the authority to regulate AQUATIC FACILITIES in their jurisdiction.

1.3.8 CDC Role

The MAHC is hosted by the Centers for Disease Control and Prevention *(CDC)*, a Federal agency whose mission is “To promote health and quality of life by preventing and controlling disease, injury, and disability.” Furthermore, CDC has been involved in developing swimming pool-related guidance since the 1950s and officially tracking waterborne disease outbreaks associated with aquatic facility use since 1978.

1.3.8.1 Public Health Role

CDC is “the primary Federal agency for conducting and supporting public health activities in the United States”; however, CDC is not a regulatory agency.

1.3.8.2 Model Guidance

The MAHC is intended to be open access guidance that state and local public health agencies can use to write or update their POOL CODES in part or in full as fits their jurisdiction’s needs. The CDC adopted this project because no other U.S. federal agency has commission over public disinfected AQUATIC FACILITIES. Considering the CDC’s mission and historical interest in aquatics, this organization was the best qualified to lead a national consortia to create such a document.

1.4 Public Health and Consumer Expectations

1.4.1 Aquatics Sector & Government Responsibility

Both the aquatics sector and the government share the responsibility of offering AQUATIC FACILITIES that provide consumers and aquatics workers with safe and healthy recreational water experiences and job sites and that do not become sources for the

spread of infectious diseases, outbreaks, or the cause of injuries. This shared responsibility extends to working to meet consumer expectations that AQUATIC FACILITIES are properly designed, constructed, operated, and maintained.

1.4.2 Swimmer Responsibility

The PATRON or BATHER shares a responsibility in maintaining a healthy swimming environment by practicing the CDC-recommended healthy swimming behaviors to improve hygiene and reduce the spread of disease. Consumers and BATHERS also share responsibility for using AQUATIC FACILITIES in a healthy and safe manner to reduce the incidence of injuries.

1.5 Advantages of Uniform Guidance

1.5.1 Sector Agreement

The aquatics sector and public health officials recognize the value in uniform, consensus guidance created by multi-sector discussion and agreement – both for getting the best possible information and gaining sector acceptance. Since most public AQUATIC FACILITIES are already regulated, the MAHC is intended to be guidance to assist, strengthen, and streamline resource use by state and local code officials or legislatures that already regulate AQUATIC FACILITIES but need to regularly update and improve their AQUATIC FACILITY oversight and regulation. Uniform, consensus guidance using the latest science and best practices helps all public sectors, including businesses and consumers, resulting in the best product and experiences.

In addition, the MAHC's combination of performance-based and prescriptive recommendations gives AQUATIC FACILITIES freedom to use innovative approaches to achieve acceptable results. However, AQUATIC FACILITIES must ensure that these recommendations are still being met, whatever the approach may be, although innovation should be encouraged to achieve outlined performance-based requirements.

1.5.2 MAHC Provisions

The MAHC provides guidance on AQUATIC FACILITY design standards & construction, operation & maintenance, and policies & management that can be uniformly adopted for the aquatics sector.

The MAHC:

- Is the collective result of the efforts and recommendations of many individuals, public health agencies, and organizations within the aquatics sector, and
- Embraces the concept that safe and healthy recreational water experiences by the public are directly affected by how we collectively design, construct, operate, and maintain our AQUATIC FACILITIES.

1.5.3 Aquatic Facility Requirements

Model performance-based recommendations essentially define public aquatic health and safety expectations, usually in terms of how dangerous a pathogen or injury is to the public. By using a combination of performance-based recommendations and prescriptive measures, AQUATIC FACILITIES are free to use innovative approaches to provide healthy and safe AQUATIC FACILITIES whereas traditional evaluations mandate how AQUATIC FACILITIES achieve acceptable results. However, to show compliance with the model performance-based recommendation, the AQUATIC FACILITY must demonstrate that control measures are in place to ensure that the recommendations are being met. The underlying theme of the MAHC is that it should be based on the latest science where possible, best practices, and that change will be gradual so all parties can prepare for upcoming changes; "Evolution, not revolution".

1.6 Modifications and Improvements in the MAHC 1st Edition

The MAHC 1st Edition was assembled from 14 modules that were posted for one 60-day public comment period each, revised based upon public comment, and reposted individually with revisions. The individual modules were then assembled and cross-checked for discrepancies and duplications arising from the modular development approach. The complete MAHC "Knitted" version was posted for an additional 60-day public comment period to allow reviewers to check wording across sections and submit additional comments. The MAHC "Knitted" version was revised based on the second round of public comment and reposted as the MAHC 1st Edition.

1.7 MAHC Adoption at State or Local Level

1.7.1 MAHC Adoption at State or Local Level

The MAHC is provided as guidance for voluntary use by governing bodies at all levels to regulate public AQUATIC FACILITIES. At the state and local levels, the MAHC may be used in part or in whole to:

1) Enact into statute as an act of the state legislative body; or
2) Promulgate as a regulation, rule or code; or
3) Adopt as an ordinance.

CDC is committed to offering, at a minimum, assistance to states and localities in interpreting and implementing the MAHC. CDC welcomes suggestions for how it could best assist localities in using this guidance in the future. CDC also offers a MAHC toolkit *(including sample forms and checklists)* and is available to give operational guidance to public health pool programs when needed. CDC is committed to expanding its support of the MAHC and ensuring timely updates and improvements.

1.7.2 Conference for the Model Aquatic Health Code

Other assistance to localities will also be available. The Conference for the Model Aquatic Health Code *(CMAHC; www.cmahc.org)*, an independent, nonprofit 501(c)(3) organization, was created with CDC support in 2013 to support and improve public

health by promoting healthy and safe aquatic experiences for everyone. The CMAHC's role is to serve as a national clearinghouse for input and advice on needed improvements to CDC's Model Aquatic Health Code *(MAHC)*.The CMAHC will fulfill this role by:

1) Collecting, assessing, and relaying national input on needed MAHC improvements back to CDC for final consideration for acceptance,
2) Advocating for improved health and safety at swimming facilities,
3) Providing consultation and assistance to health departments, boards of health, legislatures, and other partners on MAHC uses, benefits, and implementation,
4) Providing consultation and assistance to the aquatics industry on uses, interpretation, and benefits of the MAHC, and
5) Soliciting, coordinating, and prioritizing MAHC research needs.

CDC and the CMAHC will work together closely to continue to incorporate national input into the MAHC and provide optimal guidance and assistance to public health officials and the aquatics sector.

1.8 The MAHC Revision Process

1.8.1 MAHC Revisions

Throughout the creation of the MAHC, the CDC accepted concerns and recommendations for modification of the MAHC from any individual or organization through two 60-day public comment periods via the email address MAHC@cdc.gov.

1.8.2 Future Revisions

CDC realizes that the MAHC should be an evolving document that is kept up to date with the latest science, industry advances, and public health findings. As the MAHC is used and recommendations are put into practice, MAHC revisions will need to be made. As the future brings new technologies and new aquatic health issues, the CMAHC, with CDC participation, will institute a process for collecting national input that welcomes all stakeholders to participate in making recommendations to improve the MAHC so it remains comprehensive, easy to understand, and as technically sound as possible. These final recommendations will then be weighed by CDC for final incorporation into a new edition of the MAHC.

The Model Aquatic Health Code

The Code

USER GUIDE

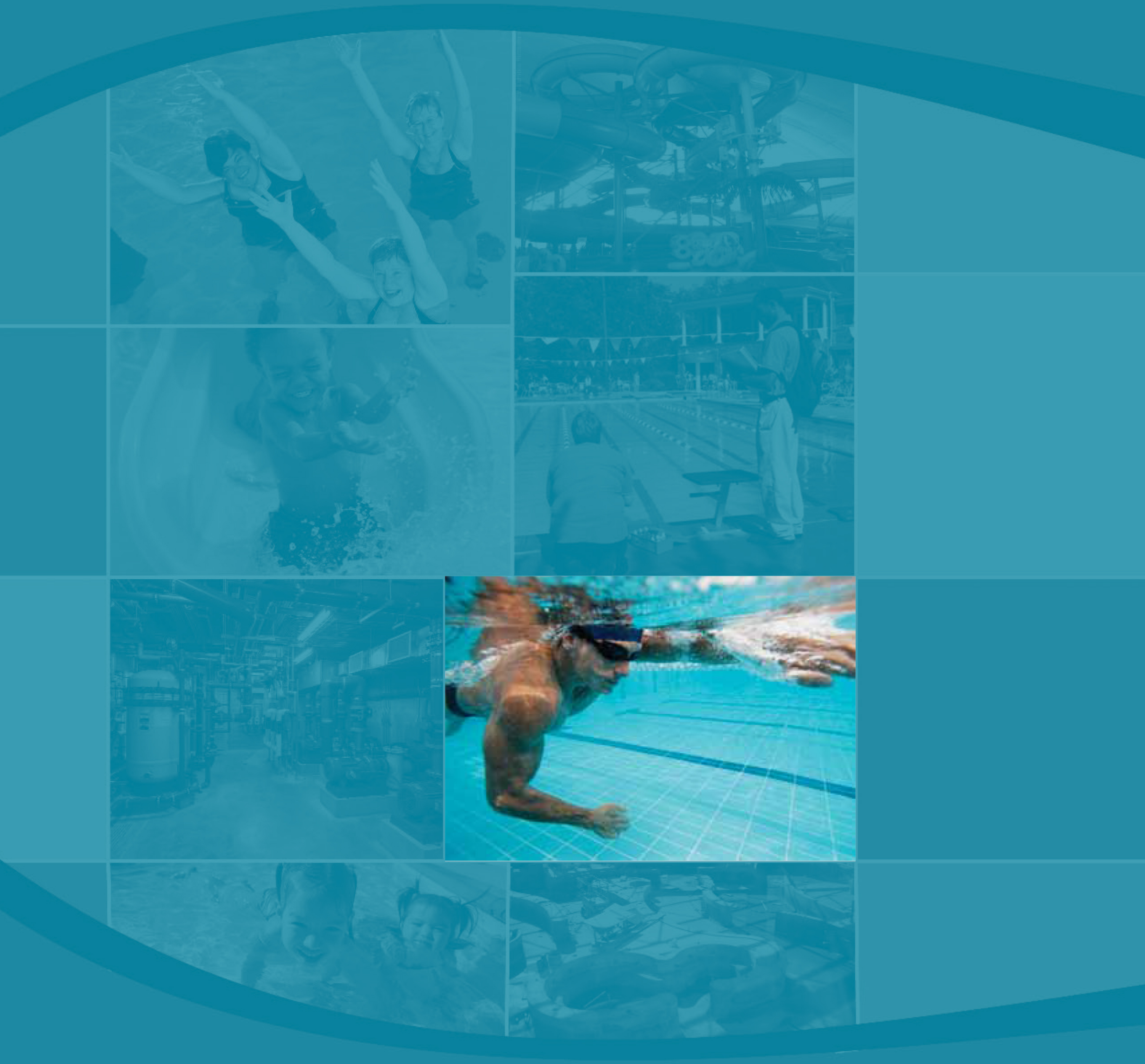

CS249779

2.0 User Guide

The provisions of Chapter 4 *(Design Standards and Construction)* apply to construction of a new AQUATIC FACILITY or AQUATIC VENUE or SUBSTANTIAL ALTERATION to an existing AQUATIC FACILITY or AQUATIC VENUE, unless otherwise noted.

The provisions of Chapter 5 and 6 apply to all AQUATIC FACILITIES covered by this code regardless of when constructed, unless otherwise noted.

2.1 Overview

2.1.1 New Users

A new user will find it helpful to review the Table of Contents in order to quickly gain an understanding of the scope and sequence of subjects included in the CODE.

2.1.2 Topic Presentations

MAHC provisions address essentially three areas:

- Design & Construction *(Chapter 4)*,
- Operation & Maintenance *(Chapter 5)*,
- Policies & Management *(Chapter 6)*.

In addition, an overarching, scientifically referenced explanation of the MAHC as a risk reduction plan is provided in the Annex using the same numbering format for easy cross reference.

2.2 MAHC Structure and Format

2.2.1 Numbering System

The CODE follows a numeric outline format. The structural numbering system having different indent, font and color size in the document is as follows:

1.0 Chapter

1.1 Part

1.1.1 Subpart

1.1.1.1 Section

1.1.1.1.1 Paragraph

1.1.1.1.1.1 Sub-Paragraph

2.2.2 Title, Keyword, Phrase Text

On the same line and next to the number is a title, keyword, or phrase summary showing the information contained in the corresponding MAHC wording below.

2.2.3 MAHC Requirement

Recommended MAHC requirement wording is shown below the number of title, keyword, or phrase. These requirements usually appear in sentence or paragraph format.

2.2.4 Illustrations

Appropriate charts, diagrams, and other illustrative material found in the Chapters will also appear in the Annex.

2.2.5 Consistency Between Chapters 4.0 and 5.0

Each Part or Sub-part is repeated throughout CODE Chapters 4.0 *(Design Standards & Construction)* and 5.0 *(Operation & Maintenance)*. For example, the section titled "Disinfection and pH Control," has two parts:

1) Design recommendations and construction aspects, addressed in Subpart 4.7.3 and
2) Operation and maintenance aspects, addressed in Subpart 5.7.3.

If a topic is not applicable then that section is marked with a N/A *(e.g., the size or width of the decking is not applicable for Operation & Maintenance versus Design Standards & Construction)*. This is designed to allow MAHC users to see how a topic of interest applies under both chapter headings.

2.2.6 Conventions

The following conventions are used in the Model Aquatic Health Code:

1) "Shall" means the act is imperative, i.e., "shall" constitutes a command.
2) "May not" means absolute prohibition.
3) "May" is permissive and means the act is allowed.
4) "Means" is followed by a declared fact.

2.2.7 Definitions

Defined glossary words and terms are in "SMALL CAPS" in the text of the CODE chapters to alert the reader that there is a specific meaning assigned to those terms and that the meaning of a provision is to be interpreted in the defined context. A concerted effort was also made to place in "SMALL CAPS" all forms and combinations of those defined words and terms that were intended to carry the weight of the definition.

2.3 Annex

2.3.1 Rationale

The annex is provided to:

1) Give further explanations of why certain recommendations are made;
2) Discuss rationale for making the MAHC content decisions;
3) Provide a discussion of the scientific basis for selecting certain criteria, as well as discuss why other scientific data may not have been selected, e.g. due to data inconsistencies;
4) State areas where additional research may be needed;
5) Discuss and explain terminology used; and
6) Provide additional material that may not have been appropriately placed in the main body of suggested MAHC recommendations. This would include summaries of scientific studies, charts, graphs, or other illustrative materials.

2.3.2 Content

The annex was developed to support the MAHC Code language and is meant to provide additional help, guidance, and rationale to those responsible for using the MAHC. Statements in the annex are intended to be supplements and additional explanations. They are not meant to be interpreted as MAHC code wording or used to create enforceable code language.

2.3.3 Bibliography

The Annex includes a list of CODES referenced, a bibliography of the reference materials, and scientific studies that form the basis for MAHC recommendations.

2.3.4 Appendices

The Appendices supply additional information or tools that may be useful to the reader of the MAHC Annex and Code.

The Model Aquatic Health Code

The Code

GLOSSARY OF ACRONYMS AND TERMS USED IN THIS CODE

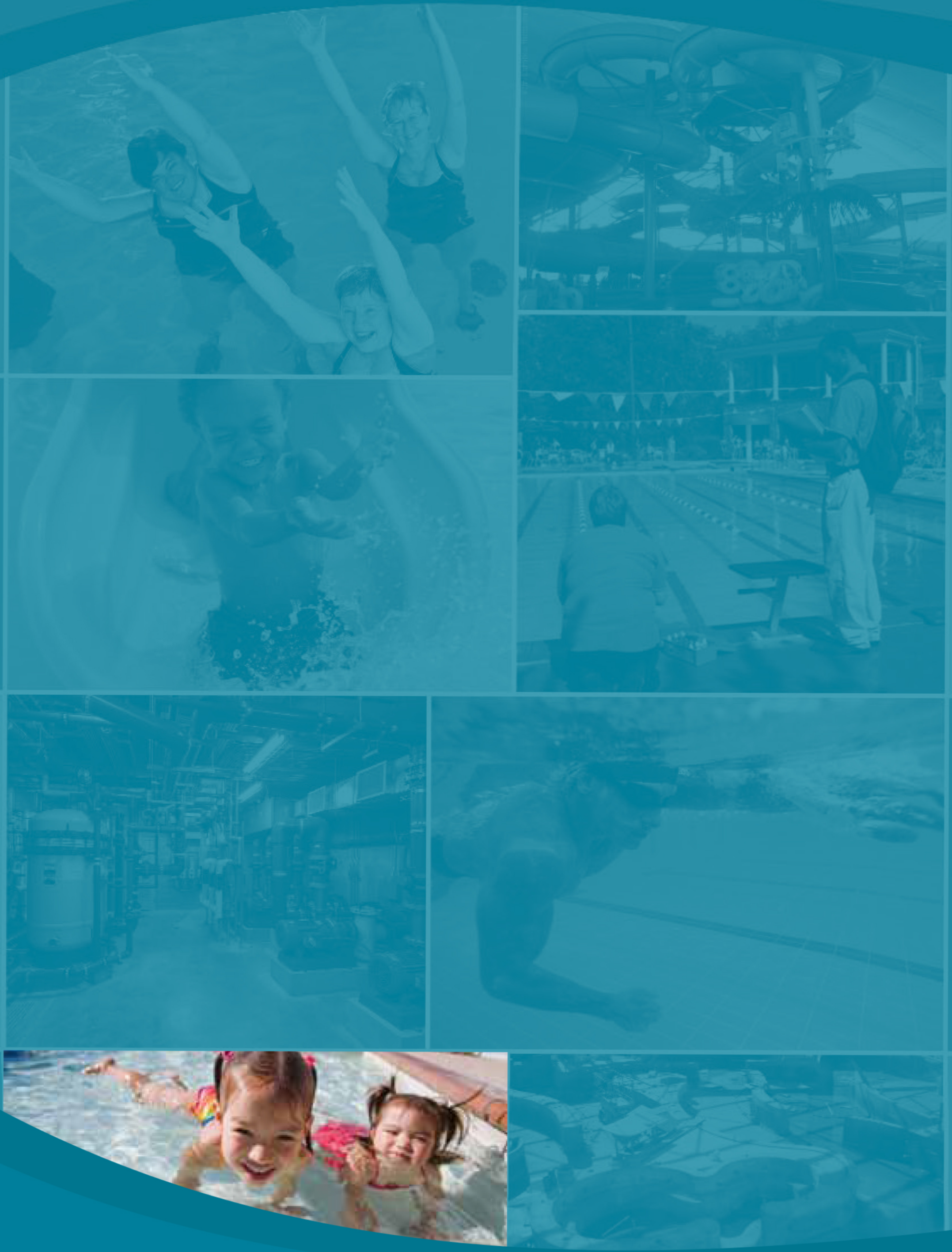

CS249779

3.0 Glossary of the MAHC Code and Annex

3.1 Acronyms and Initialisms Used in This Code and Annex

ACCA	Air Conditioning Contractors of America
ADA	Americans with Disabilities Act
ADASAD	2010 Americans with Disabilities Standards for Accessible Design
AED	Automated External Defibrillator
AHJ	Authority Having Jurisdiction
AMCA	Air Movement and Control Association
ANSI	American National Standards Institute
ASHRAE	American Society of Heating, Refrigerating and Air-Conditioning Engineers
ASME	American Society of Mechanical Engineers
ASTM	American Society for Testing and Materials
ATSDR	Agency for Toxic Substances and Disease Registry
BCDMH	1-bromo-3-chloro-5, 5-dimethylhydantoin
CCPRF	Citizen CPR Foundation
CDC	Centers for Disease Control and Prevention
CEU	Continuing Education Units
CFM	Cubic Feet Per Minute
CMAHC	The Conference for the Model Aquatic Health Code
CPR	Cardiopulmonary Resuscitation
CPSC	Consumer Product Safety Commission
CYA	Cyanuric Acid
DBDMH	Dibromodimethylhydantoin
DBP	Disinfection By-Product
DVGW	Deutscher Verein des Gas- und Wasserfaches e.V. – Technisch wissenschaftlicher Verein *(German Technical and Scientific Association for Gas and Water)*
EAP	Emergency Action Plan
ECCU	Emergency Cardiovascular Care Update
EPA	Environmental Protection Agency
FAC	Free Available Chlorine
FIFRA	Federal Insecticide, Fungicide, and Rodenticide Act
FINA	Federation Internationale de Natation Amateur
GFCI	Ground-Fault Circuit Interrupter
GPM	Gallons Per Minute
HMIS	Hazardous Material Identification System
HOCl	Hypochlorous Acid
HSEESS	Hazardous Substance Emergency Events Surveillance System
IARC	International Agency for Research on Cancer
IBC	International Building Code
ICC	International Code Commission
ICBO	International Council of Building Officials
IESNA	Illuminating Engineering Society of North America
ILCOR	International Liaison Committee on Resuscitation

IPC	International Plumbing Code
ISPSC	International Swimming Pool and Spa Code
MAHC	Model Aquatic Health Code
MERV	Minimum Efficiency Reporting Value
METS	Metabolic Equivalents
NCAA	National Collegiate Athletic Association
NEC	National Electrical Code
NEHA	National Environmental Health Association
NEISS	National Electrical Injury Surveillance System
NFPA	National Fire Protection Association
NPDS	National Poison Data System
NPSH	Net Positive Suction Head
NRTL	Nationally Recognized Testing Laboratory
NSF	National Sanitation Foundation
OEM	Original Equipment Manufacturer
ÖNORM	Österreichisches Normungsinstitut *(Austrian Standards Institute)*
ORP	Oxidation Reduction Potential
OSHA	Occupational Safety and Health Administration
PEL	Permissible Exposure Limit
PHMB	Polyhexamethylene Biguanide Hydrochloride
POS	Perimeter Overflow System
PPE	Personal Protective Equipment
PPM	Parts Per Million
PVC	Polyvinyl Chloride
PVC-P	Plasticized Polyvinyl Chloride
RED	Reduction Equivalent Dose
RLV	Relative Limit Value
RPZ	Reduced Pressure Zone
RWI	Recreational Water Illness
SDS	Safety Data Sheet
SCBA	Self-Contained Breathing Apparatus
SCI	Spinal Cord Injury
SMACNA	Sheet Metal and Air Conditioning Contractors' National Association
SVRS	Safety Vacuum Release System
TDH	Total Dynamic Head
TDS	Total Dissolved Solids
THM	Trihalomethane
TLV	Threshold Limit Value
UL	Underwriter Laboratories
UPC	Uniform Plumbing Code
USLSC	United States Lifeguarding Standards Coalition
USPSHTC	Uniform Swimming Pool, Spa, and Hot Tub Code
UV	Ultraviolet
VFD	Variable Frequency Drive
WC	Water Closet
WQTD	Water Quality Testing Device

3.2 Glossary of Terms Used in This Code and Annex

“Accessible Route” means access/egress standards as defined by 2010 ADA Standards for Accessible Design.

“Activity Pool” See “Pool.”

“Air Handling System” means equipment that brings in outdoor air into a building and removes air from a building for the purpose of introducing air with fewer contaminants and removing air with contaminants created while bathers are using aquatic venues. The system contains components that move and condition the air for temperature, humidity, and pressure control, and transport and distribute the air to prevent condensation, corrosion, and stratification, provide acceptable indoor air quality, and deliver outside air to the breathing zone.

“Agitated Water” means an aquatic venue with mechanical means *(aquatic features)* to discharge, spray, or move the water's surface above and/or below the static water line of the aquatic venue. Where there is no static water line, movement shall be considered above the deck plane.

“Aquatic Facility” means a physical place that contains one or more aquatic venues and support infrastructure.

“Aquatic Feature” means an individual component within an aquatic venue. Examples include slides, structures designed to be climbed or walked across, and structures that create falling or shooting water.

“Aquatic Facility or Aquatic Venue Enclosure” means an uninterrupted barrier surrounding and securing an aquatic facility or aquatic venue.

“Aquatic Venue” means an artificially constructed structure or modified natural structure where the general public is exposed to water intended for recreational or therapeutic purpose. Such structures do not necessarily contain standing water, so water exposure may occur via contact, ingestion, or aerosolization. Examples include swimming pools, wave pools, lazy rivers, surf pools, spas *(including spa pools and hot tubs)*, therapy pools, waterslide landing pools, spray pads, and other interactive water venues.

- **“Increased Risk Aquatic Venue”** means an aquatic venue which due to its intrinsic characteristics and intended users has a greater likelihood of affecting the of the bathers of that venue by being at increased risk for microbial contamination *(e.g., by children less than 5 years old)* or being used by people that may be more susceptible to infection *(e.g., therapy patients with open wounds)*. Examples of increased-risk aquatic venues include spray pads, wading pools and other aquatic venues designed for children less than five years old as well as therapy pools.

- **"Lazy River"** means a channeled flow of water of near−constant depth in which the water is moved by pumps or other means of propulsion to provide a river−like flow that transports bathers over a defined path. A lazy river may include play features and devices. A lazy river may also be referred to as a tubing pool, leisure river, leisure pool or a current channel.
- **"Spa"** means a structure intended for either warm or cold water where prolonged exposure is not intended. Spa structures are intended to be used for bathing or other recreational uses and are not usually drained and refilled after each use. It may include, but is not limited to, hydrotherapy, air induction bubbles, and recirculation.
- **"Special Use Aquatic Venue"** means aquatic venues that do not meet the intended use and design features of any other aquatic venue or pool listed/identified in this Code.

"Authority Having Jurisdiction" *(AHJ)* means an agency, organization, office, or individual responsible for enforcing the requirements of a code or standard, or for approving equipment, materials, installations, or procedures.

"Automated Controller" means a system of at least one chemical probe, a controller, and auxiliary or integrated component that senses the level of one or more water parameters and provides a signal to other equipment to maintain the parameters within a user-established range.

"Available Chlorine" *See "Chlorine"*

"Backflow" means a hydraulic condition caused by a difference in water pressure that causes an undesirable reversal of the flow as the result of a higher pressure in the system than in its supply.

"Barrier" means an obstacle intended to prevent direct access from one point to another.

"Bather" means a person at an aquatic venue who has contact with water either through spray or partial or total immersion. The term bather as defined, also includes staff members, and refers to those users who can be exposed to contaminated water as well as potentially contaminate the water.

"Bather Count" means the number of bathers in an aquatic venue at any given time.

"Best Practice" means a technique or methodology that, through experience and research, has been proven to reliably lead to a desired result.

"Body of Water" *(per NEC, q.v.)* means any aquatic venue holding standing water, whether permanent or storable.

"Breakpoint Chlorination" means the conversion of inorganic chloramine compounds to nitrogen gas by reaction with Free Available Chlorine. When chlorine is added to water containing ammonia *(from urine, sweat, or the environment, for example)*, it initially reacts with the ammonia to form monochloramine. If more chlorine is added, monochloramine is converted into dichloramine, which decomposes into nitrogen gas, hydrochloric acid and chlorine. The apparent residual chlorine decreases since it is partially reduced to hydrochloric acid. The point at which the drop occurs is referred to as the "breakpoint". The amount of free chlorine that must be added to the water to achieve breakpoint chlorination is approximately ten times the amount of combined chlorine in the water. As additional chlorine is added, all inorganic combined chlorine compounds disappear, resulting in a decrease in eye irritation potential and "chlorine odors."

"Bulkheads" means a movable partition that physically separates a pool into multiple sections.

"Chemical Storage Space" means a space in an aquatic facility used for the storage of pool chemicals such as acids, salt, or corrosive or oxidizing chemicals.

"Chlorine" means an element that at room temperature and pressure is a heavy greenish yellow gas with a characteristic penetrating and irritating smell; it is extremely toxic. It can be compressed in liquid form and stored in heavy steel tanks. When mixed with water, chlorine gas forms hypochlorous acid, the primary chlorine-based disinfecting agent, hypochlorite ion, and hydrochloric acid. Hypochlorous acid dissociation to hypochlorite ion is highly pH dependent. Chlorine is a general term used in the MAHC which refers to hypochlorous acid and hypochlorite ion in aqueous solution derived from chlorine gas or a variety of chlorine-based disinfecting agents.

- **"Available Chlorine"** means the amount of chlorine in the +1 oxidation state, which is the reactive, oxidized form. In contrast, chloride ion *(Cl^-)* is in the -1 oxidation state, which is the inert, reduced state. Available Chlorine is subdivided into Free Available Chlorine and Combined Available Chlorine. Pool chemicals containing Available Chlorine are both oxidizers and disinfectants. Elemental chlorine *(Cl_2)* is defined as containing 100% available chlorine. The concentration of Available Chlorine in water is normally reported as mg/L *(PPM)* "as Cl_2", that is, the concentration is measured on a Cl_2 basis, regardless of the source of the Available Chlorine.

- **"Free Chlorine Residual"** OR **"Free Available Chlorine"** means the portion of the total available chlorine that is not "combined chlorine" and is present as hypochlorous acid *(HOCl)* or hypochlorite ion *(OCl^-)*.The pH of the water determines the relative amounts of hypochlorous acid and hypochlorite ion. HOCl is a very effective bactericide and is the active bactericide in pool water. OCl^- is also a bactericide, but acts more slowly than HOCl. Thus, chlorine is a more effective bactericide at low pH than at high pH. A free chlorine residual must be maintained for adequate disinfection.

“Circulation Path” means an exterior or interior way of passage from one part of an aquatic facility to another for pedestrians, including, but not limited to walkways, pathways, decks, and stairways. This must be considered in relation to ADA.

“Cleansing Shower” *See “Shower.”*

“Code” means a systematic statement of a body of law, especially one given statutory force.

“Combustion Device” means any appliance or equipment using fire. These include, but may not be limited to, gas or oil furnaces, boilers, pool heaters, domestic water heaters, etc.

“Construction Joint” means a watertight joint provided to facilitate stopping places in the construction process. Construction joints also serve as contraction joints which control cracking.

“Contamination Response Plan” means a plan for handling contamination from formed-stool, diarrheal-stool, vomit, and blood.

“Contaminant” means a substance that soils, stains, corrupts, or infects another substance by contact or association.

“Corrosive Materials” means pool chemicals, fertilizers, cleaning chemicals, oxidizing cleaning materials, salt, de-icing chemicals, other corrosive or oxidizing materials, pesticides, and such other materials which may cause injury to people or damage to the building, air-handling equipment, electrical equipment, safety equipment, or fire-suppression equipment, whether by direct contact or by contact via fumes or vapors, whether in original form or in a foreseeably likely decomposition, pyrolysis, or polymerization form. Refer to labels and SDS forms.

“Crack” means any and all breaks in the structural shell of a pool vessel or deck.

“Cross-Connection” means a connection or arrangement, physical or otherwise, between a potable water supply system and a plumbing fixture, tank, receptor, equipment, or device, through which it may be possible for non-potable, used, unclean, polluted and contaminated water, or other substances to enter into a part of such potable water system under any condition.

“CT Value” means a representation of the concentration of the disinfectant *(C)* multiplied by time in minutes *(T)* needed for inactivation of a particular contaminant. The concentration and time are inversely proportional; therefore, the higher the concentration of the disinfectant, the shorter the contact time required for inactivation. The CT value can vary with pH or temperature change so these values must also be supplied to allow comparison between values.

"Deck" means surface areas serving the aquatic venue, including the perimeter/wet deck, pool deck, and dry deck.

- **"Dry Deck"** means all pedestrian surface areas within the aquatic venue enclosure not subject to frequent splashing or constant wet foot traffic. The dry deck is not perimeter deck or pool deck, which connect the pool to adjacent amenities, entrances, and exits. Landscape areas are not included in this definition.
- **"Perimeter/Wet Deck"** means the hardscape surface area immediately adjacent to and within four feet *(1.2 m)* of the edge of the swimming pool also known as the "wet deck" area.
- **"Pool Deck"** means surface areas serving the aquatic venue, beyond perimeter deck, which is expected to be regularly trafficked and made wet by bathers.

"Diaper-Changing Station" means a hygiene station that includes a diaper-changing unit, hand-washing sink, soap and dispenser, a means for drying hands, trash receptacle, and disinfectant products to clean after use.

"Diaper-Changing Unit" means a diaper-changing surface that is part of a diaper-changing station.

"Dichloramine" means a disinfection by-product formed when chlorine binds to nitrogenous waste in pool water to form an amine- containing compound with two chlorine atoms *($NHCl_2$)*. It is a known acute respiratory and ocular irritant.

"Disinfection" means a treatment that kills or irreversibly inactivates microorganisms *(e.g., bacteria, viruses, and parasites)*; in water treatment, a chemical *(commonly chlorine, chloramine, or ozone)* or physical process *(e.g., ultraviolet radiation)* can be used.

"Disinfection By-Product" means a chemical compound formed by the reaction of a disinfectant *(e.g. chlorine)* with a precursor *(e.g. natural organic matter, nitrogenous waste from bathers)* in a water system *(pool, water supply)*.

"Diving Pool" *See "Pool."*

"Drop Slide" *See "Slide."*

"Dry Deck" *See "Deck."*

"Emergency Action Plan" means a plan that identifies the objectives that need to be met for a specific type of emergency, who will respond, what each person's role will be during the response. and what equipment is required as part of the response.

"Enclosure" means an uninterrupted constructed feature or obstacle used to surround and secure an area that is intended to deter or effectively prevent unpermitted,

uncontrolled, and unfettered access . It is designed to resist climbing and to prevent passage through it and under it. Enclosure can apply to aquatic facilities or aquatic venues.

"EPA Registered" means all products regulated and registered under the Federal Insecticide, Fungicide, and Rodenticide Act *(FIFRA)* by the U.S. Environmental Protection Agency *(EPA; http://www.epa.gov/agriculture/lfra.html)*. EPA registered products will have a registration number on the label *(usually it will state "EPA Reg No." followed by a series of numbers)*. This registration number can be verified by using the EPA National Pesticide Information Retrieval System *(http://ppis.ceris.purdue.edu/#)*.

"Equipment Room" means a space intended for the operation of pool pumps, filters, heaters, and controllers. This space is not intended for the storage of hazardous pool chemicals.

"Exit Gate" means an emergency exit, which is a gate or door allowing free exit at all times.

"Expansion Joint" means a watertight joint provided in a pool vessel used to relieve flexural stresses due to movement caused by thermal expansion/contraction.

"Flat Water" means an aquatic venue in which the water line is static except for movement made by users. Diving spargers do not void the flat water definition.

"Flume" means the riding channels of a waterslide which accommodate riders using or not using mats, tubes, rafts, and other transport vehicles as they slide along a path lubricated by a water flow.

"Foot Baths" means standing water in which bathers or aquatics staff rinse their feet.

"Free Chlorine Residual" OR **"Free Available Chlorine"** *See "Chlorine."*

"Ground-Fault Circuit Interrupter" means a device for protection of personnel that de-energizes an electrical circuit or portion thereof in the event of excessive ground current.

"Hand Wash Station" means a location which has a hand wash sink, adjacent soap with dispenser, hand drying device or paper towels and dispenser, and trash receptacle.

"Hot Water" means an aquatic venue with water temperature over 90 degrees Fahrenheit *(30 degrees Celsius)*.

"Hygiene Facility" means a structure or part of a structure that contains toilet, shower, diaper-changing unit, hand wash station, and dressing capabilities serving bathers and patrons at an aquatic facility.

“Hygiene Fixtures” means all components necessary for hygiene facilities including plumbing fixtures, diaper-changing stations, hand wash stations, trashcans, soap dispensers, paper towel dispensers or hand dryers, and toilet paper dispensers.

“Hyperchlorination” means the intentional and specific raising of chlorine levels for a prolonged period of time to inactivate pathogens following a fecal or vomit release in an aquatic venue as outlined in MAHC Section 6.5.

“Imminent Health Hazard” means a significant threat or danger to health that is considered to exist when there is evidence sufficient to show that a product, practice, circumstance, or event creates a situation that requires immediate correction or cessation of operation to prevent injury based on the number of potential injuries and the nature, severity, and duration of the anticipated injury or illness.

“Increased Risk Aquatic Venue” *See “Aquatic Venue.”*

“Indoor Aquatic Facility” means a physical place that contains one or more aquatic venues and the surrounding bather and spectator/stadium seating areas within a structure that meets the definition of “Building” per the 2012 International Building Code. It does not include equipment, chemical storage, or bather hygiene rooms or any other rooms with a direct opening to the aquatic facility. Otherwise known as a natatorium.

“Infinity Edge” means a pool wall structure and adjacent perimeter deck that is designed in such a way where the top of the pool wall and adjacent deck are not visible from certain vantage points in the pool or from the opposite side of the pool. Water from the pool flows over the edge and is captured and treated for reuse through the normal pool filtration system. They are often also referred to as “vanishing edges,” “negative edges,” or “zero edges.”

“Inlet” means wall or floor fittings where treated water is returned to the pool.

“Interactive Water Play Aquatic Venue” means any indoor or outdoor installation that includes sprayed, jetted or other water sources contacting bathers and not incorporating standing or captured water as part of the bather activity area. These aquatic venues are also known as splash pads, spray pads, wet decks. For the purposes of the MAHC, only those designed to recirculate water and intended for public use and recreation shall be regulated.

“Interior Space” means any substantially enclosed space having a roof and having a wall or walls which might reduce the free flow of outdoor air. Ventilation openings, fans, blowers, windows, doors, etc., shall not be construed as allowing free flow of outdoor air.

“Island” means a structure inside a pool where the perimeter is completely surrounded by the pool water and the top is above the surface of the pool.

“Landing Pool” *See “Pool.”*

“Lazy River” *See “Aquatic Venue.”*

“mg/L” means milligrams per liter and is the equivalent metric measure to parts per million *(PPM)*.

“Monitoring” is the regular and purposeful observation and checking of systems or facilities and recording of data, including system alerts, excursions from acceptable ranges, and other facility issues. Monitoring includes human or electronic means.

“Moveable Floors” means a pool floor whose depth varies through the use of controls.

“No Diving Marker” means a sign with the words “No Diving” and the universal international symbol for “No Diving” pictured as an image of a diver with a red circle with a slash through it.

“Oocyst” means the thick-walled, environmentally resistant structure released in the feces of infected animals that serves to transfer the infectious stages of sporozoan parasites *(e.g., Cryptosporidium)* to new hosts.

“Oxidation” means the process of changing the chemical structure of water contaminants by either increasing the number of oxygen atoms or reducing the number of electrons of the contaminant or other chemical reaction, which allows the contaminant to be more readily removed from the water or made more soluble in the water. It is the “chemical cleaning” of pool water. Oxidation can be achieved by common disinfectants *(e.g., chlorine, bromine)*, secondary disinfection/sanitation systems *(e.g. ozone)* and oxidizers *(e.g. potassium monopersulfate)*.

“Oxidation Reduction Potential” means a measure of the tendency for a solution to either gain or lose electrons; higher *(more positive)* oxidation reduction potential indicates a more oxidative solution.

“Patron” means a bather or other person or occupant at an aquatic facility who may or may not have contact with aquatic venue water either through partial or total immersion. Patrons may not have contact with aquatic venue water, but could still be exposed to potential contamination from the aquatic facility air, surfaces, or aerosols.

“Peninsula / Wing Wall” means a structural projection into a pool intended to provide separation within the body of water.

“Perimeter Deck” *See “Deck.”*

“Perimeter Gutter System” means the alternative to skimmers as a method to remove water from the pool’s surface for treatment. The gutter provides a level structure along the pool perimeter versus intermittent skimmers.

“Plumbing Fixture” means a receptacle, fixture, or device that is connected to a water supply system or discharges to a drainage system or both and may be used for the

distribution and use of water; for example: toilets, urinals, showers, and hose bibs. Such receptacles, fixtures, or devices require a supply of water; or discharge liquid waste or liquid-borne solid waste; or require a supply of water and discharge waste to a drainage system.

"pH" means the negative log of the concentration of hydrogen ions. When water ionizes, it produces hydrogen ions *(H+)* and hydroxide ions *(OH-)*. If there is an excess of hydrogen ions the water is acidic. If there is an excess of hydroxide ions the water is basic. pH ranges from 0 to 14. Pure water has a pH of 7.0. If pH is higher than 7.0, the water is said to be basic, or alkaline. If the water's pH is lower than 7.0, the water is acidic. As pH is raised, more ionization occurs and chlorine disinfectants decrease in effectiveness.

"Pool" means a subset of aquatic venues designed to have standing water for total or partial bather immersion. This does not include spas.

- **"Activity Pool"** means a water attraction designed primarily for play activity that uses constructed features and devices including pad walks, flotation devices, and similar attractions.
- **"Diving Pool"** means a pool used exclusively for diving.
- **"Landing Pool"** means an aquatic venue or designated section of an aquatic venue located at the exit of one or more waterslide flumes. The body of water is intended and designed to receive a bather emerging from the flume for the purpose of terminating the slide action and providing a means of exit to a deck or walkway area.
- **"Skimmer Pool"** means a pool using a skimmer system.
- **"Surf Pool"** means any pool designed to generate waves dedicated to the activity of surfing on a surfboard or analogous surfing device commonly used in the ocean and intended for sport as opposed to general play intent for wave pools.
- **"Therapy Pool"** means a pool used exclusively for aquatic therapy, physical therapy, and/or rehabilitation to treat a diagnosed injury, illness, or medical condition, wherein the therapy is provided under the direct supervision of a licensed physical therapist, occupational therapist, or athletic trainer. This could include wound patients or immunocompromised patients whose health could be impacted if there is not additional water quality protection.
- **"Wading Pool"** means any pool used exclusively for wading and intended for use by young children where the depth does not exceed two feet *(0.6 m)*.
- **"Wave Pools"** means any pool designed to simulate breaking or cyclic waves for purposes of general play. A wave pool is not the same as a surf pool, which generates waves dedicated to the activity of surfing on a surfboard or analogous

surfing device commonly used in the ocean and intended for sport as opposed to general play intent for wave pools.

"Pool Deck" *See "Deck."*

"Pool Slide" *See "Slide."*

"Public Water Systems" means water systems including community water systems, non-transient/non-community water systems, or transient non-community water systems with exceptions as noted by AHJ and EPA.

"Purge" means to introduce a large volume of outdoor air to flush the interior space.

"Qualified Lifeguard" means an individual who has successfully completed an AHJ-recognized lifeguard training course offered by an AHJ-recognized training agency, holds a current certificate for such training, has met the pre-service requirements, and is participating in continuing in-service training requirements of the aquatic facility.

"Qualified Lifeguard Supervisor" means an individual responsible for the oversight of lifeguard performance and emergency response at an aquatic facility. A qualified lifeguard supervisor is an individual who has successfully completed a lifeguard supervisor training course and holds an unexpired certificate for such training; and who has met the pre-service and continuing in-service requirements of the aquatic facility according to this code.

"Qualified Operator" means an individual responsible for the operation and maintenance of the water and air quality systems and the associated infrastructure of the aquatic facility and who has successfully completed an AHJ-recognized operator training course to operate an aquatic facility offered by an AHJ-recognized training agency and holds a current certificate for such training.

"Recessed Steps" means a way of ingress/egress for a pool similar to a ladder but the individual treads are recessed into the pool wall.

"Recirculation System" means the combination of the main drain, gutter or skimmer, inlets, piping, pumps, controls, surge tank or balance tank to provide pool water recirculation to and from the pool and the treatment systems.

"Reduction Equivalent Dose *(RED)* bias" means a variable used in UV system validation to account for differences in UV sensitivity between the UV system challenge microbe *(e.g., MS2 virus)* and the actual microbe to be inactivated *(e.g., Cryptosporidium)*.

"Re-entrainment" means a situation where the exhaust*(s)* from a ventilated source such as an indoor aquatic facility is located too close to the air handling system intake*(s)*, which allows the exhausted air to be re-captured by the air handling system so it is transported directly back into the aquatic facility.

“Responsible Supervisor” means an individual on-site that is responsible for water treatment operations when a “qualified operator” is not on-site at an aquatic facility.

“Rinse Shower” *See “Shower.”*

“Robotic Cleaner” means a modular vacuum system consisting of a motor-driven, in-pool suction device, either self-powered or powered through a low voltage cable, which is connected to a deck-side power supply.

“Runout” means that part of a waterslide where riders are intended to decelerate and/or come to a stop. The runout is a continuation of the waterslide flume surface.

“Safety” *(as it relates to construction items)* means a design standard intended to prevent inadvertent or hazardous operation or use *(i.e., a passive engineering strategy)*.

“Safety Plan” means a written document that has procedures, requirements and/or standards related to safety which the aquatic facility staff shall follow. These plans include training, emergency response, and operations procedures.

“Safety Team” means any employee of the aquatic facility with job responsibilities related to the aquatic facility’s emergency action plan.

“Sanitize” means reducing the level of microbes to that considered safe by public health standards *(usually 99.999%)*. This may be achieved through a variety of chemical or physical means including chemical treatment, physical cleaning, or drying.

“Saturation Index” means a mathematical representation or scale representing the ability of water to deposit calcium carbonate, or dissolve metal, concrete or grout.

“Secondary Disinfection Systems” means those disinfection processes or systems installed in addition to the standard systems required on all aquatic venues, which are required to be used for increased risk aquatic venues.

“Shower” means a device that sprays water on the body.

- **“Cleansing Shower”** means a shower located within a hygiene facility using warm water and soap. The purpose of these showers is to remove contaminants including perianal fecal material, sweat, skin cells, personal care products, and dirt before bathers enter the aquatic venue.

- **“Rinse Shower”** means a shower typically located in the pool deck area with ambient temperature water. The main purpose is to remove dirt, sand, or organic material prior to entering the aquatic venue to reduce the introduction of contaminants and the formation of disinfection by-products.

“Skimmer” means a device installed in the pool wall whose purpose is to remove floating debris and surface water to the filter. They shall include a weir to allow for the

automatic adjustment to small changes in water level, maintaining skimming of the surface water.

"Skimmer Pool" *See "Pool."*

"Skimmer System" means periodic locations along the top of the pool wall for removal of water from the pool's surface for treatment.

"Slide" means an aquatic feature where users slide down from an elevated height into water.

- **"Drop Slide"** means a slide that drops bathers into the water from a height above the water versus delivering the bather to the water entry point.
- **"Pool Slide"** means a slide having a configuration as defined in The Code of Federal Regulations *(CFR)* Ch. II, Title 16 Part 1207 by CSPC, or is similar in construction to a playground slide used to allow users to slide from an elevated height to a pool. They shall include children's *(tot)* slides and all other non- flume slides that are mounted on the pool deck or within the basin of a public swimming pool.
- **"Waterslide"** means a slide that runs into a landing pool or runout through a fabricated channel with flowing water.

"Spa" *See "Aquatic Venue."*

"Special Use Aquatic Venue" *See "Aquatic Venue."*

"Standard" means something established by authority, custom, or general consent as a model or example.

"Storage" means the condition of remaining in one space for one hour or more. Materials in a closed pipe or tube awaiting transfer to another location shall not be considered to be stored.

"Structural Crack" means a break or split in the pool surface that weakens the structural integrity of the vessel.

"Substantial Alteration" means the alteration, modification, or renovation of an aquatic venue *(for outdoor aquatic facilities)* or indoor aquatic facility *(for indoor aquatic facilities)* where the total cost of the work exceeds 50% of the replacement cost of the aquatic venue *(for outdoor aquatic facilities)* or indoor aquatic facility *(for indoor aquatic facilities)*.

"Superchlorination" means the addition of large quantities of chlorine-based chemicals to kill algae, destroy odors, or improve the ability to maintain a disinfectant residual. This process is different from Hyperchlorination, which is a prescribed amount

to achieve a specific CT value whereas Superchlorination is the raising of free chlorine levels for water quality maintenance.

“Supplemental Treatment Systems” means those disinfection processes or systems which are not required on an aquatic venue for health and safety reasons. They may be used to enhance overall system performance and improve water quality.

“Surf Pool” *See “Pool.”*

“Theoretical Peak Occupancy” means the anticipated peak number of bathers in an aquatic venue or the anticipated peak number of occupants of the decks of an aquatic facility. This is the lower limit of peak occupancy to be used for design purposes for determining services that support occupants. Theoretical peak occupancy is used to determine the number of showers. For aquatic venues, the theoretical peak occupancy is calculated around the type of water use or space:

- **“Flat Water”** means an aquatic venue in which the water line is static except for movement made by users usually as a horizontal use as in swimming. Diving spargers do not void the flat water definition.
- **“Agitated Water”** means an aquatic venue with mechanical means *(aquatic features)* to discharge, spray, or move the water's surface above and/or below the static water line of the aquatic venue so people are standing or playing vertically. Where there is no static water line, movement shall be considered above the deck plane.
- **“Hot Water”** means an aquatic venue with a water temperature over 90°F *(32°C)*.
- **“Stadium Seating”** means an area of high-occupancy seating provided above the pool level for observation.

“Therapy Pool” *See “Pool.”*

“Toe Ledge” *See “Underwater Ledge.”*

“Trichloramine” means a disinfection by-product formed when chlorine binds to nitrogenous waste in pool water to form an amine-containing compound with three chlorine atoms *(NCl_3)*. It is a known acute respiratory and ocular irritant. It has low solubility in water and is rapidly released into the air above pools where it can accumulate, particularly in indoor settings.

“Trihalomethanes” means chemical compounds in which three of the four hydrogen atoms of methane *(CH_4)* are replaced by halogen atoms. Trihalomethanes are environmental pollutants and many are considered carcinogenic.

"Turnover" or "Turnover Rate" means the period of time, usually expressed in hours, required to circulate a volume of water equal to the capacity of the aquatic venue.

"Underwater Bench" means a submerged seat with or without hydrotherapy jets.

"Underwater Ledge" or "Underwater Toe Ledge" means a continuous step in the pool wall that allows swimmers to rest by standing without treading water.

"UV Transmissivity" means the percentage measurement of ultraviolet light able to pass through a solution.

"Wading Pool" *See "Pool."*

"Waterslide" *See "Slide."*

"Water Replenishment System" means a way to remove water from the pool as needed and replace with make-up water in order to maintain water quality.

"Water Quality Testing Device" means a product designed to measure the level of a parameter in water. A WQTD includes a device or method to provide a visual indication of a parameter level, and may include one or more reagents and accessory items.

"Wave Pools" *See "Pool."*

"Wing Wall / Peninsula" *See "Peninsula / Wing Wall."*

"Zero Depth Entry" means a sloped entry into a pool from deck level into the interior of the pool as a means of access and egress.

The Model Aquatic Health Code

The Code

DESIGN AND CONSTRUCTION

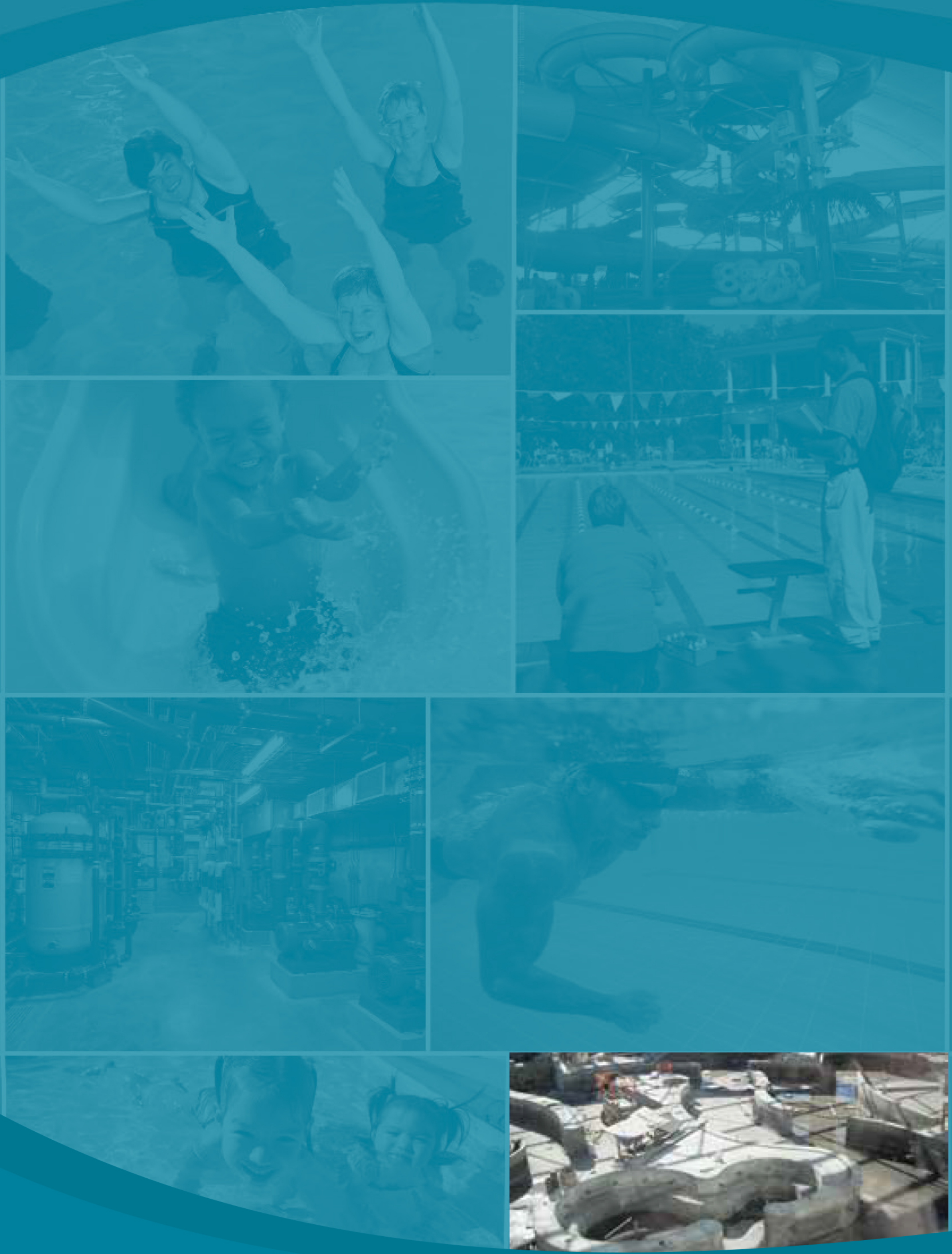

CS249779

4.0 Facility Design Standards and Construction

The provisions of MAHC Chapter 4 *(Facility Design Standards and Construction)* apply to construction of a new AQUATIC FACILITY or AQUATIC VENUE or SUBSTANTIAL ALTERATION to an existing AQUATIC FACILITY or AQUATIC VENUE, unless otherwise noted.

4.1 Plan Submittal

4.1.1 Plan Submittal

4.1.1.1 Purpose

AQUATIC FACILITY construction plans shall be designed to provide sufficient clarity to indicate the location, nature, and extent of the work proposed.

4.1.1.2 Conform

AQUATIC FACILITY construction plans shall show in detail that it will conform to the provisions of this CODE and relevant laws, ordinances, rules, and regulations, as determined by the AHJ and to protect the health and SAFETY of the facility's BATHERS and PATRONS.

4.1.1.3 Approved Plans

No person shall begin to construct a new AQUATIC FACILITY or shall SUBSTANTIALLY ALTER an existing AQUATIC FACILITY without first having the construction plans detailing the construction or SUBSTANTIAL ALTERATION submitted to and approved by the AHJ.

4.1.1.4 Plan Preparation

All plans shall be prepared by a design professional who is registered or licensed to practice their respective design profession as defined by the state or local laws governing professional practice within the jurisdiction in which the project is to be constructed.

4.1.1.5 Required Statements

All construction plans shall include the following statements:

1) "The proposed AQUATIC FACILITY and all equipment shall be constructed and installed in conformity with the approved plans and specifications or approved amendments," and

2) "No SUBSTANTIAL ALTERATION, changes, additions, or equipment not specified in the approved plans or allowed in the CODE can be made or added until the plans for such SUBSTANTIAL ALTERATION, changes, additions, or equipment are submitted to and approved by the AHJ."

4.1.2 Content of Design Report

4.1.2.1 Basis of Design Report

4.1.2.1.1 Names / Addresses

AQUATIC FACILITY plans shall include the name, address, and contact information for the owner, designer, and builder if available at the time of submission.

4.1.2.1.2 Site Information

AQUATIC FACILITY plans shall include site information indicating at a minimum the location of all utilities, wells, topography, natural water features, and potential sources of surface drainage and pollution which may affect the proposed AQUATIC FACILITY.

4.1.2.1.3 Plot Plan

AQUATIC FACILITY plans shall include a site plot plan including:

1) A general map and detailed scaled drawings of the AQUATIC FACILITY site plan or floor plan with detailed locations of the AQUATIC VENUES and AQUATIC FEATURES; and
2) The locations of all water supply facilities, sources of drinking water, public or private sewers, and relative elevations of paved or other walkways and the EQUIPMENT ROOM floor shall be shown on the plans with the elevations of storm and sanitary sewer inverts and street grade.

4.1.2.2 Plans and Specifications

4.1.2.2.1 Drawings

Detailed scaled and dimensional drawings for each individual AQUATIC VENUE shall include an AQUATIC VENUE area plan and layout plan along with dimensioned longitudinal and transverse cross sections of the AQUATIC VENUE.

4.1.2.2.2 Venue Attributes

Detailed scaled and dimensional drawings for each individual AQUATIC VENUE shall include location and type of:

1) INLETS,
2) Overflows,
3) Drains,
4) Suction outlets,
5) Overflow gutters or devices,
6) Piping,
7) Designed POOL water elevation,
8) AQUATIC FEATURES such as ladders, stairs, diving boards, slides, and play features,
9) Lighting,
10) Pool markings, and
11) Surface materials

4.1.2.2.3 Area Design

Detailed scaled and dimensional drawings of the AQUATIC FACILITY and for each individual AQUATIC VENUE, as appropriate, shall include location and type of:

1) Design of DECK, curb, or walls enclosing the AQUATIC VENUE,
2) DECK drains,
3) Paved walkways and other hardscape features,
4) Non-slip flooring,
5) AQUATIC VENUE area finishes,
6) Drinking fountains or other sources of drinking water,
7) Entries and exits,
8) Hose bibs,
9) Fences,
10) Telephones, and
11) Area lighting.

4.1.2.2.4 Venue Recirculation and Treatment Design

Detailed scaled and dimensional drawings for each individual AQUATIC VENUE shall contain a flow diagram showing the location, plan, elevation, and schematics of:

1) Filters,
2) Pumps,
3) Chemical feeders and interlocks
4) Chemical controllers and interlocks,
5) Secondary disinfection systems, if required,
6) Supplementary disinfection systems, if installed,
7) Ventilation devices or AIR HANDLING SYSTEMS,
8) Heaters,
9) Surge tanks, including operating levels,
10) BACKFLOW prevention assemblies and air gaps,
11) Valves,
12) Piping,
13) Flow meters,
14) Gauges,
15) Thermometers,
16) Test cocks,
17) Sight glasses, and
18) Drainage system for the disposal of AQUATIC VENUE water and filter wastewater.

4.1.2.2.5 Equipment Room Design

Detailed scaled and dimensional drawings for each individual AQUATIC VENUE shall contain a schematic layout of the AQUATIC VENUE EQUIPMENT ROOM *(or equipment area if permitted by the local AHJ)* showing accessibility for installation and maintenance.

4.1.2.2.6 Chemical Storage Space Design

Detailed scaled and dimensional drawings for each individual AQUATIC VENUE shall contain a schematic layout of the AQUATIC FACILITY CHEMICAL STORAGE SPACE*(s)*.

4.1.2.2.7 Hygiene Facility Design

Detailed scaled and dimensional drawings for each AQUATIC FACILITY shall show the location and number of all available HYGIENE FACILITIES provided including dressing rooms, lockers and basket STORAGE, showers, lavatory, toilet fixtures, and DIAPER-CHANGING STATIONS.

4.1.2.3 Technical Specifications

4.1.2.3.1 Accompanying Drawings

Technical specifications for the construction of each AQUATIC VENUE and all appurtenances shall accompany the drawings for the AQUATIC FACILITY plans.

4.1.2.3.2 Technical Details

The technical specifications for each AQUATIC FACILITY and each AQUATIC VENUE shall include all construction details not shown on the plans that relate to the AQUATIC FACILITY.

4.1.2.3.3 Water Sources

The technical specifications for each AQUATIC FACILITY shall include the sources of all water supplies.

4.1.2.3.4 Area and Volume

Technical specifications shall include the water surface area and volume of each AQUATIC VENUE and associated water features, if applicable.

4.1.2.3.5 Theoretical Peak Occupancy

The technical specifications for each AQUATIC FACILITY and each AQUATIC VENUE shall include THEORETICAL PEAK OCCUPANCY, respectively.

4.1.2.3.5.1 Used for Designing Systems

The THEORETICAL PEAK OCCUPANCY for an AQUATIC VENUE shall be used for designing systems that serve BATHERS and PATRONS. *(Note: The specified density factors are the lower limits for determining THEORETICAL PEAK OCCUPANCY.)*

4.1.2.3.5.2 Incorporate Non-Water Related Areas

The THEORETICAL PEAK OCCUPANCY for an AQUATIC FACILITY shall be used for designing systems that serve BATHERS and PATRONS and shall incorporate non-water related areas such as DECKS and other adjacent portions of the AQUATIC FACILITY not associated with the AQUATIC VENUE.

4.1.2.3.5.3 Calculating Theoretical Peak Occupancy

The THEORETICAL PEAK OCCUPANCY shall be calculated by dividing the surface area in square feet of the AQUATIC VENUE by the density factor *(D)* that fits the specific AQUATIC VENUE being considered.

THEORETICAL PEAK OCCUPANCY = AQUATIC VENUE surface area / D

The density factors *(D)* are:

Water/bather-related:

1) FLAT WATER density factor = 20 ft^2 *(1.9 m^2)* per BATHER.
2) AGITATED WATER density factor = 15 ft^2 *(1.4 m^2)* per BATHER.
3) HOT WATER density factor = 10 ft^2 *(0.9 m^2)* per BATHER.
4) WATERSLIDE LANDING POOL density factor = manufacturer-established capacity at any given time.
5) INTERACTIVE WATER PLAY water density factor = 10 ft^2 *(0.9 m^2)* per BATHER on surface.

Non-water/PATRON-related

6) DECK density factor = 50 ft^2 *(4.6 m^2)* per BATHER.
7) Stadium seating density factor = 6.6 ft^2 *(0.6 m^2)* per BATHER.

4.1.2.3.5.3.1 Density Factor Modification

The density factors in MAHC Section 4.1.2.3.5.3 may be modified for higher BATHER or PATRON density, but they shall not be modified to be lower than the density factors listed in MAHC Section 4.1.2.3.5.3.

4.1.2.3.5.3.2 Aquatic Facility Theoretical Peak Occupancy

The THEORETICAL PEAK OCCUPANCY for an AQUATIC FACILITY shall be determined by adding the calculations for each AQUATIC VENUE in the AQUATIC FACILITY.

4.1.2.3.6 Equipment Characteristics and Rating

The technical specifications and supplemental engineering data for each AQUATIC FACILITY and each AQUATIC VENUE shall include:

1) Detailed information on the type, size, operating characteristics, and rating of all mechanical and electrical equipment;
2) Hydraulic computations for head loss in all piping and recirculation equipment; and
3) Pump curves that demonstrate that the selected recirculation pump*(s)* are adequate for the calculated required flows.

4.1.2.3.7 Recirculation Rate and Turnover

The technical specifications for each AQUATIC VENUE shall include the recirculation rate and TURNOVER time.

4.1.2.3.8 Filter Media

The technical specifications for each AQUATIC VENUE shall include information on the filter media such as diatomaceous earth, sand, gravel or other approved material.

4.1.2.3.9 Equipment Specifications

The technical specifications for each AQUATIC VENUE shall include information on each piece of equipment associated with that AQUATIC VENUE.

4.1.2.3.10 Safety Equipment Specifications

The technical specifications for each AQUATIC FACILITY shall include information on all aquatic SAFETY equipment.

4.1.2.3.11 Other Specifications

The technical specifications for each AQUATIC FACILITY and each AQUATIC VENUE shall include additional information related to the project requested by the AHJ for the purposes of the construction of the AQUATIC FACILITY and each AQUATIC VENUE and all appurtenances.

4.1.3 Plan Approval

4.1.3.1 New Construction

4.1.3.1.1 Approval Limitations

The AHJ shall clearly state on the plans the limitations of their approval.

4.1.3.1.2 Other Approvals

The approval shall also state that it is independent of all other required approvals such as Building, Zoning, Fire, Electrical, Structural, and any other approvals as required by local or state law or CODE and the applicant must separately obtain all other required approvals and permits.

4.1.3.1.3 Plan Review Coordination

The AHJ shall coordinate their AQUATIC FACILITY plan review and communicate their approval with other agencies involved in the AQUATIC FACILITY construction.

4.1.3.1.4 Plan Review Report

The AHJ shall provide a plan submission compliance review list to the AQUATIC FACILITY owner with the following information:

1) Categorical items marked satisfactory, unsatisfactory, not applicable, or insufficient information;
2) A comment section keyed to the compliance review list shall detail unsatisfactory and insufficient;
3) Indication of the AHJ approval or disapproval of the AQUATIC FACILITY construction plans;
4) In the case of a disapproval, specific reasons for disapproval and procedure for resubmittal; and
5) Reviewer's name, signature, and date of review.

4.1.3.1.5 Plans Maintained

The AQUATIC FACILITY owner shall maintain at least one set of their own approved plans made available to AHJ on-site for as long as the AQUATIC FACILITY is in operation.

4.1.3.2 Non-Substantial Alterations

4.1.3.2.1 Alteration Review

The AQUATIC FACILITY owner planning a non-SUBSTANTIAL ALTERATION shall contact the AHJ to review proposed changes prior to starting the non-SUBSTANTIAL ALTERATION.

4.1.3.2.2 Alteration Scope

The AQUATIC FACILITY operator shall consult with the AHJ to determine if new or modified plans must be submitted for plan review and approval for other non-SUBSTANTIAL ALTERATIONS proposed.

4.1.3.3 Replacements

4.1.3.3.1 Replacement Equipment Equivalency

The replacement of pumps, filters, feeders, controllers, SKIMMERS, flow-meters, valves, or other similar equipment with identical or substantially similar equipment may be done without submission to the AHJ for approval of new or altered AQUATIC FACILITY plans.

4.1.3.3.2 Replacement Approval

The AQUATIC FACILITY owner shall submit to the AHJ the replacement equipment technical specification that verifies the proposed replacement equipment's equivalency to the originally approved and installed equipment prior to equipment's replacement.

4.1.3.3.3 Emergency Replacement

In emergencies, the replacement may be made prior to receiving the AHJ's approval, with the owner accepting responsibility for proper immediate replacement, if the equipment is not deemed equivalent by the AHJ.

4.1.3.3.3.1 Documentation

Where emergency replacements are installed as per MAHC Section 4.1.3.3.3, the owner shall submit documentation for review and approval of the replacement to the AHJ within 45 days.

4.1.3.3.4 Replacement Record Maintenance

The AHJ shall provide the AQUATIC FACILITY owner written approval or disapproval of the proposed replacement equipment's equivalency.

4.1.3.3.5 Documentation

Documentation of proposed, approved, and disapproved replacements shall be maintained in the AHJ's AQUATIC FACILITY files.

4.1.4 Compliance Certificate

4.1.4.1 Construction Compliance Certificate

A certificate of construction compliance shall be submitted to the AHJ for all AQUATIC FACILITY plans for new construction and SUBSTANTIAL ALTERATIONS requiring AHJ approvals.

4.1.4.2 Certificate Preparation

This certificate shall be prepared by a licensed professional and be within the scope of their practice as defined by the state or local laws governing professional practice within the jurisdiction of the permit issuing official.

4.1.4.3 Certificate Statement

The certificate shall also include a statement that the AQUATIC FACILITY, all equipment, and appurtenances have been constructed and/or installed in accordance with approved plans and specifications.

4.1.4.4 Systems Commissioning

If commissioning or testing reports for systems such as AQUATIC FACILITY lighting, air handling, recirculation, filtration, and/or DISINFECTION are conducted, then those reports shall be included in furnished documentation.

4.1.4.5 Maintenance

Documentation of AQUATIC FACILITY new construction or SUBSTANTIAL ALTERATION plan compliance shall be maintained in the AHJ's AQUATIC FACILITY files.

4.1.5 Construction Permits

4.1.5.1 Building Permit for Construction

Construction permits required in this CODE and all other applicable permits shall be obtained before any AQUATIC FACILITY may be constructed.

4.1.5.2 Remodeling Building Permit

A construction permit or other applicable permits may be required from the AHJ before SUBSTANTIAL ALTERATION of an AQUATIC FACILITY.

4.1.5.3 Permit Issuance

The AHJ shall issue a permit to the owner to operate the AQUATIC FACILITY:

1) After receiving a certificate of completion from the design professional verifying information submitted, and
2) When new construction, SUBSTANTIAL ALTERATIONS, or annual renewal requirements of this CODE have been met.

4.1.5.4 Permit Denial

The permit *(license)* to operate may be withheld, revoked or denied by the AHJ for noncompliance of the AQUATIC FACILITY with the requirements of this CODE, and the owner will be provided:

1) Specific reasons for disapproval and procedure for resubmittal;
2) Notice of the rights to appeal this denial and procedures for requesting an appeal; and
3) Reviewer's name, signature and date of review and denial.

4.1.5.5 Documentation

Documentation of AQUATIC FACILITY permit renewal or denial shall be maintained in the AHJ's AQUATIC FACILITY files.

4.2 Materials

4.2.1 Pools

4.2.1.1 Construction Material

AQUATIC VENUES shall be constructed of reinforced concrete or impervious and structurally sound material*(s),* which provide a smooth, easily cleaned, watertight structure capable of withstanding the anticipated stresses/loads for full and empty conditions taking into consideration climatic, hydrostatic, seismic, and the integration of the AQUATIC VENUE with other structural conditions and as required by applicable CODES.

4.2.1.2 Durability

All materials shall be inert, non-toxic, resistant to corrosion, impervious, enduring, and resistant to damages related to environmental conditions of the installation region.

4.2.1.3 Areas Subject to Freezing

Where located in areas subject to freezing, AQUATIC VENUES and appurtenances shall be protected and designed from damage due to freezing.

4.2.1.4 Darker Colors

The AHJ may grant a variance to the color requirements of this CODE for Munsell color values less than 6.5.

4.2.1.5 Competitive Pools

Competitive or lap POOLS may have lane markings and end wall targets installed in accordance with FINA, NCAA, USA Swimming, NFSHSA, or other recognized STANDARD.

4.2.1.6 Design Parameters

Any graphics, color, or finish incorporated into the construction of a POOL or painted on the floor or walls must not prevent the detection of a BATHER in distress, algae, sediment, or other objects in the AQUATIC VENUE.

4.2.1.6.1 Permission in Writing

Permission in writing from the AHJ for the use of graphics that do not comply with the requirements of this CODE shall be obtained before the graphics are used.

4.2.1.7 Watertight

POOLS shall be designed in such a way to maintain their ability to retain the designed amount of water.

4.2.1.8 Smooth Finish

All vertical walls shall have a durable finish suitable for regular scrubbing and cleaning at the waterline.

4.2.1.8.1 Daily Cleaning

The finish shall be able to withstand daily brushing, scrubbing, and cleaning of the surface in accordance with the manufacturer's recommendations.

4.2.1.8.2 Skimmer Pools

SKIMMER POOLS shall have a six inch *(152 mm)* to 12 inch *(305 mm)* high waterline finish that meets the requirements of MAHC Section 4.2.1.8 and 4.2.1.8.1.

4.2.1.8.3 Gutter / Perimeter Overflow Systems

Gutter or perimeter overflow systems shall have a minimum finish height of two inches (*51 mm)* that meets the requirements of MAHC Section 4.2.1.8 and 4.2.1.8.1.

4.2.1.8.4 Dark Colors

If dark colors in excess of what is required in this code are used for the POOL finish, these colors shall not extend more than 12 inches *(305 mm)* below the waterline.

4.2.1.9 Slip Resistant

POOL floors in areas less than three feet *(0.9 m)* deep shall have a slip resistant finish with an acceptable coefficient of friction.

4.2.1.10 Stainless Steel, Vinyl, PVC-P or PVC Pools

Stainless steel, vinyl, PVC-P, or PVC panel and liner POOL finish systems shall be acceptable provided that the system is installed on top of approved materials and design requirements as listed within this section or approved by the AHJ.

4.2.1.10.1 Damaged

If at any time the liner system is damaged or cut in such a way that its integrity is compromised, the POOL shall be shut down until the system is fully repaired.

4.2.1.11 Not Permitted

Wood, sand, or earth shall not be permitted as an interior finish.

4.2.2 Indoor Aquatic Facility

4.2.2.1 Interior Finish

4.2.2.1.1 Relative Humidity

The interior finish of an INDOOR AQUATIC FACILITY shall be designed for an indoor relative humidity as not less than 80%.

4.2.2.2 Condensation Prevention

4.2.2.2.1 Cold Weather

INDOOR AQUATIC FACILITY building envelope construction shall include a vapor-retarder/insulation arrangement to assist in preventing the condensation of water on inside building surfaces under the coldest outdoor conditions based on the ASHRAE climate data for the project locale or nearest reporting city and the highest design indoor relative humidity.

4.2.2.2.2 Paint or Coating

Where a paint or coating serves as the vapor retarder of an INDOOR AQUATIC FACILITY, the paint or coating shall be applied so as to produce a permeability rating of 0.2 U.S. perm *(11.4* $ng \cdot s^{-1} \cdot m^{-2} \cdot Pa^{-1}$*)* or less.

4.2.2.2.2.1 Application

The paint or coating shall be applied according to the manufacturer's recommendations for use as a vapor retarder.

4.2.2.2.3 Perforated Interior-Finish Material

Where a perforated interior-finish material is used in an INDOOR AQUATIC FACILITY, as for acoustic effects, the perforated material shall not be considered to be a vapor retarder unless it has a listed permeability rating less than 0.2 U.S. perm *(11.4* $ng \cdot s^{-1} \cdot m^{-2} \cdot Pa^{-1}$*)*.

4.2.2.3 Mechanical Systems

4.2.2.3.1 Equipment Rooms

For EQUIPMENT ROOMS, see MAHC Section 4.9.1.

4.2.2.3.2 Chemical Storage Spaces

For CHEMICAL STORAGE SPACES, see MAHC Section 4.9.2.

4.2.2.3.3 Indoor Aquatic Facility Air Pressure

AQUATIC FACILITY AIR HANDLING SYSTEM design, construction, and installation shall comply with the 2011 ASHRAE Applications Handbook on Natatorium Design ASHRAE Standard 62.1, *Ventilation for Acceptable Indoor Air Quality*, and/or applicable local CODES with additional requirements as stated in section MAHC 4.6.2.

4.2.2.3.3.1 Chemical Storage Space Air Pressure

AIR HANDLING SYSTEM design for CHEMICAL STORAGE SPACES shall conform to the International Mechanical Code or Uniform Mechanical Code, and either the International Fire Code or the Uniform NFPA 1 Fire Code, and any applicable local CODES.

4.2.2.3.4 Air Ducts

Where air ducts are required, they shall be resistant to corrosion from the airborne chemicals.

4.2.2.3.4.1 Material Options

Coated steel, aluminum, and fabric ducts may be options to consider.

4.2.2.3.4.2 Insulated Exterior

Ducts shall be insulated on the exterior of the duct with a mold-resistant material where the surface temperature of the duct is capable of being less than the airstream temperature within the duct.

4.2.2.3.5 Filters

Filters for outdoor-air intake shall be rated moisture-resistant.

4.2.2.4 Indoor Aquatic Facility Doors

4.2.2.4.1 Corrosion-Resistant

INDOOR AQUATIC FACILITY doors shall either be constructed of corrosion-resistant materials or have a covering or coating to withstand humid and CORROSIVE environments which is acceptable to the AHJ.

4.2.2.4.2 Uncontrolled Condensation

INDOOR AQUATIC FACILITY doors which may be exposed to temperatures below INDOOR AQUATIC FACILITY-air dew point shall have thermal breaks, insulation, and/or glazing as necessary to minimize the risk of uncontrolled condensation.

4.2.2.4.2.1 Heating Systems

Exception: Other doors shall be acceptable, subject to approval by the AHJ, where heating systems are so arranged as to maintain such doors above the maximum design dew point of the INDOOR AQUATIC FACILITY air.

4.2.2.4.3 Biological Contaminants

INDOOR AQUATIC FACILITY doors and door-frame construction shall not contribute to the growth of biological CONTAMINANTS.

4.2.2.4.4 Air Leakage

INDOOR AQUATIC FACILITY doors and/or door frames shall be equipped with seals and/or gaskets to minimize air leakage when the door is closed.

4.2.2.4.5 Automatic Door Closer

All pedestrian doors around the INDOOR AQUATIC FACILITY perimeter shall be equipped with an automatic door closer capable of closing the door completely without human assistance.

4.2.2.4.5.1 Difference in Air Pressure

Door closers must be able to close the door against the specified difference in air pressure between the INDOOR AQUATIC FACILITY and other INTERIOR SPACES.

4.2.2.5 Indoor Aquatic Facility Windows

4.2.2.5.1 Frames

INDOOR AQUATIC FACILITY window frames shall be constructed of suitable materials or shall have a suitable covering or coating to withstand the expected atmosphere.

4.2.2.5.2 Biological Contaminants

INDOOR AQUATIC FACILITY window frames shall be constructed of materials that do not contribute to the growth of biological CONTAMINANTS.

4.2.2.5.3 Thermal Breaks

INDOOR AQUATIC FACILITY window frames shall have thermal breaks or be otherwise constructed to minimize the risk of uncontrolled condensation.

4.2.2.6 Indoor Aquatic Facility Electrical Systems and Components

Refer to MAHC Section 4.6.3

4.3 Equipment Standards

4.3.1 General

4.3.1.1 Accredited Standards

Where applicable, all equipment used or proposed for use in AQUATIC FACILITIES governed under this CODE shall be:

1) Of a proven design and construction, and
2) Listed and labeled to a specific standard for the specified equipment use by an ANSI-accredited certification organization.

4.3.1.2 No Standards

Where STANDARDS do not exist, technical documentation shall be submitted to the AHJ to demonstrate acceptability for use in AQUATIC FACILITIES.

4.3.1.2.1 Proof of Acceptability

The AHJ shall have the authority to require tests, as proof of acceptability.

4.3.2 Recirculation Systems and Equipment

4.3.2.1 ANSI-Accredited Testing

Where applicable, all equipment used or proposed to use in AQUATIC FACILITIES shall be of proven design and construction and shall be listed and labeled by an ANSI-accredited certification organization, or have EPA registration where applicable.

4.3.2.2 Suitable for Intent

RECIRCULATION SYSTEMS and all materials used therein shall be suitable for their intended use and be installed in accordance with this CODE, as listed and labeled to a specific standard by an ANSI-Accredited certification organization, and as specified by the manufacturer.

4.4 Aquatic Venue Operation and Facility Maintenance [N/A]

4.5 Aquatic Venue Structure

4.5.1 Design for Risk Management

Design of AQUATIC FACILITIES and/or AQUATIC VENUE*(s)* shall include the owner and/or an aquatic risk management consultant to incorporate operational considerations such as the layout of zones of PATRON surveillance.

4.5.1.1 Basic Requirements

The AQUATIC VENUE shape shall provide for the SAFETY of swimmers, thorough and complete circulation of the water, the ability to clean and maintain the AQUATIC VENUE, and the supervision of BATHERS and PATRONS using the AQUATIC VENUE.

4.5.1.2 Water Clarity

The water in an AQUATIC VENUE shall be sufficiently clear such that the bottom is visible while the water is static.

4.5.1.2.1 Observing Water Clarity

To make this observation, a four inch x four inch square *(10.2 cm x 10.2 cm)* marker tile in a contrasting color to the POOL floor or main suction outlet shall be located at the deepest part of the POOL.

4.5.1.2.2 Pools Over Ten Feet Deep

For POOLS over 10 feet deep *(3.0 m)* an eight inch by eight inch square *(20.3 cm x 20.3 cm)* marker tile in a contrasting color to the POOL floor or main suction outlet shall be located at the deepest part of the POOL.

4.5.1.2.3 Visible

This reference point shall be visible at all times at any point on the DECK up to 30 feet *(9.1 m)* away in a direct line of sight from the tile or main drain.

4.5.1.2.4 Spas

For SPAS, this test shall be performed when the water is in a non-turbulent state and bubbles have been allowed to dissipate.

4.5.2 Bottom Slope

4.5.2.1 Parameters and Variance

The bottom slope of a POOL shall be governed by the following parameters, but variances may be granted for special uses and situations so long as public SAFETY and health are not compromised.

4.5.2.2 Under Five Feet

In water depths under five feet *(1.5 m)*, the slope of the floor of all POOLS shall not exceed one foot *(30.5 cm)* vertical drop for every 12 feet *(3.7 m)* horizontal.

4.5.2.3 Over Five Feet

In water depths five foot *(1.5 m)* and greater, the slope of the floors of all POOLS shall not exceed one foot *(30.5 cm)* vertical to three feet *(0.9 m)* horizontal, except that POOLS designed and used for competitive diving shall be designed to meet the STANDARDS of the sanctioning organization *(such as NFSHSA, NCAA, USA Diving or FINA)*.

4.5.2.4 Drain

POOLS shall be designed so that they drain without leaving puddles or trapped standing water.

4.5.3 Pool Access / Egress

4.5.3.1 Accessibility

Each POOL shall have a minimum of two means of access and egress with the exception of:

1) WATERSLIDE LANDING POOLS,
2) WATERSLIDE RUNOUTS, and
3) WAVE POOLS.

4.5.3.2 Acceptable Means

Acceptable means of access / egress shall include stairs / handrails, grab rails / RECESSED STEPS, ladders, ramps, swimouts, and zero-depth entries.

4.5.3.3 Large Venues

For POOLS wider than 30 feet *(9.1 m)*, such means of access / egress shall be provided on each side of the POOL, and shall not be more than 75 feet *(22.9 m)* apart.

4.5.4 Stairs

4.5.4.1 Slip Resistant

Where provided, stairs shall be constructed with slip-resistant materials.

4.5.4.2 Outlined Edges

The leading horizontal and vertical edges of stair treads shall be outlined with slip-resistant contrasting tile or other permanent marking of not less than one inch *(25.4 mm)* and not greater than two inches *(50.8 mm)*.

4.5.4.3 Deep Water

Where stairs are provided in POOL water depths greater than five feet *(1.5 m)*, they shall be recessed and not protrude into the swimming area of the POOL. the lowest tread shall be not less than four feet *(1.2 m)* below normal water elevation.

4.5.4.4 Rectangular Stairs

Traditional rectangular stairs shall have a minimum uniform horizontal tread depth of 12 inches *(30.5 cm)*, and a minimum unobstructed tread width of 24 inches *(61.0 cm)*.

4.5.4.5 Dimensions

Dimensions of stair treads for other types of stairs shall conform to requirements of

1) MAHC Table 4.5.4.5,
2) MAHC Figure 4.5.4.5.1,
3) MAHC Figure 4.5.4.5.2, and
4) MAHC Figure 4.5.4.5.3.

Table 4.5.4.5: Required Dimensions for Stair Treads and Risers

Dimensions	T-1 Standard	T-1 Convex, Concave, Triangular	T-2	W-1	H-1
Minimum	12 inches *(30.5 cm)*	21 inches *(53.3 cm)*	12 inches *(30.5 cm)*	24 inches *(61.0 cm)*	6 inches *(15.2 cm)*
Maximum	18 inches *(45.7 cm)*	24 inches *(61.0 cm)*	16 inches *(40.6 cm)*	N/A	12 inches *(30.5 cm)*

Figure 4.5.4.5.1: Stair Treads and Risers

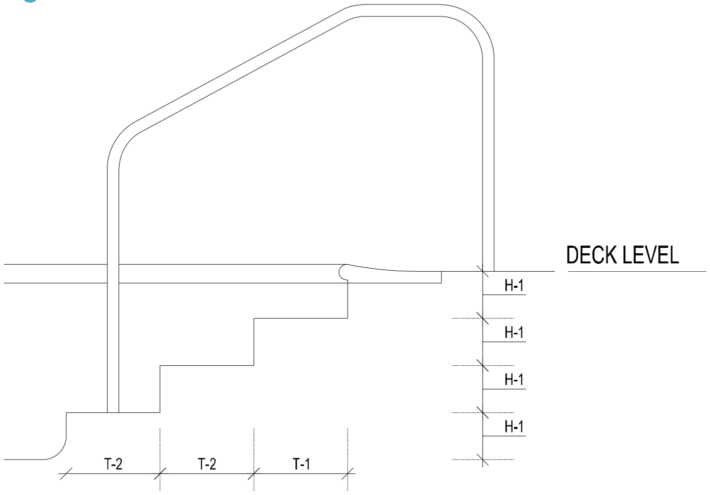

Figure 4.5.4.5.2: Stair Treads

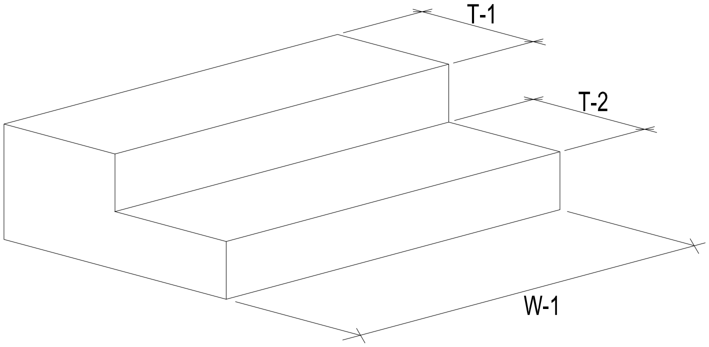

Figure 4.5.4.5.3: Unique Stair Treads

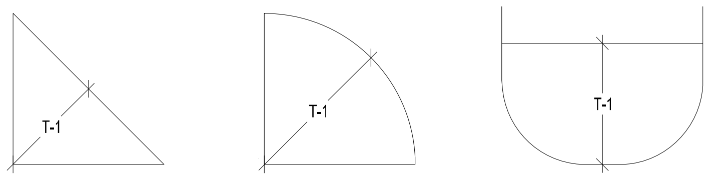

4.5.4.6 Stair Risers

Stair risers shall have a minimum uniform height of six inches *(15.2 cm)* and a maximum height of 12 inches *(30.5 cm)*, with a tolerance of ½ inches *(12.7 mm)* between adjacent risers. Stairs shall not be used underwater to transition between two sections of pool of different depths.

Note: The bottom riser may vary due to potential cross slopes with the POOL floor; however, the bottom step riser may not exceed the maximum allowable height required by this section.

4.5.4.7 Top Surface

The top surface of the uppermost stair tread shall be located not more than 12 inches *(30.5 cm)* below the POOL coping or DECK.

4.5.4.8 Perimeter Gutter Systems

For POOLS with PERIMETER GUTTER SYSTEMS, the gutter may serve as a step, provided that the gutter is provided with a grating or cover and conforms to all construction and dimensional requirements herein specified.

4.5.5 Handrails

4.5.5.1 Provided

Handrail*(s)* shall be provided for each set of stairs.

4.5.5.2 Corrosion-resistant

Handrails shall be constructed of corrosion-resistant materials, and anchored securely.

4.5.5.3 Upper Railing

The upper railing surface of handrails shall extend above the POOL coping or DECK a minimum of 28 inches *(71.1 cm)*.

4.5.5.4 Wider Than Five feet

Stairs wider than five feet *(1.5 m)* shall have at least one additional handrail for every 12 feet *(3.7 m)* of stair width.

4.5.5.5 ADA Accessibility

Handrail outside dimensions intended to serve as a means of ADA accessibility shall conform to requirements of MAHC Section 4.5.5.7.

4.5.5.6 Support

Handrails shall be designed to resist a load of 50 pounds *(22.7 kg)* per linear foot applied in any direction and independently a single concentrated load of 200 pounds (90.7 kg) applied in any direction at any location. Hand rails shall be designed to transfer these loads through the supports to the POOL or DECK structure.

4.5.5.7 Dimensions

Dimensions of handrails shall conform to requirements of MAHC Table 4.5.5.7 and MAHC Figure 4.5.5.7.1.

Table 4.5.5.7: Stair Handrail Dimensions

Dimensions	T-1	H-1
Minimum	3 inches *(7.6 cm)*	28 inches *(71.1 cm)*
Maximum	N/A	36 inches *(91.4 cm)*

Figure 4.5.5.7.1: Stair Handrails

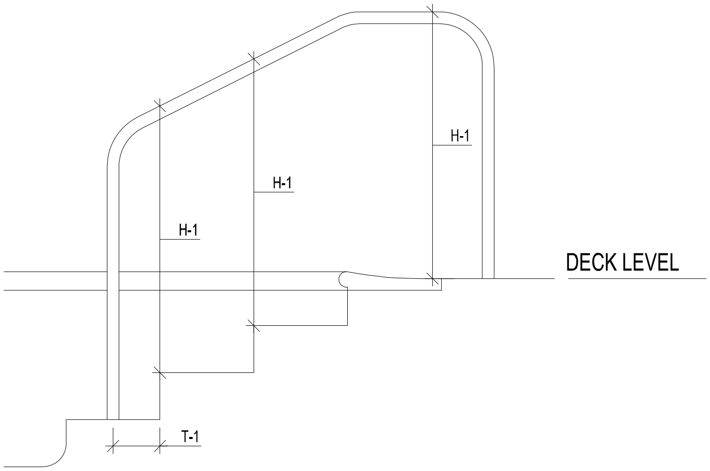

4.5.6 Grab Rails

4.5.6.1 Corrosion- Resistant

Where grab rails are provided, they shall be constructed of corrosion-resistant materials.

4.5.6.2 Anchored

Grab rails shall be anchored securely.

4.5.6.3 Provided

Grab rails shall be provided at both sides of RECESSED STEPS.

4.5.6.4 Clear Space

The horizontal clear space between grab rails shall be not less than 18 inches *(45.7 cm)* and not more than 24 inches *(61.0 cm)*.

4.5.6.5 Upper Railing

The upper railing surface of grab rails shall extend above the POOL coping or DECK a minimum of 28 inches *(71.1 cm)*.

4.5.6.6 Support

Grab rails shall be designed to resist a load of 50 pounds *(22.7 kg)* per linear foot applied in any direction and independently a single concentrated load of 200 pounds *(90.7 kg)* applied in any direction at any location. Grab rails shall be designed to transfer these loads through the supports to the POOL or DECK structure.

4.5.7 Recessed Steps

4.5.7.1 Slip-Resistant

RECESSED STEPS shall be slip-resistant.

4.5.7.2 Easily Cleaned

RECESSED STEPS shall be designed to be easily cleaned.

4.5.7.3 Drain

RECESSED STEPS shall drain into the POOL.

4.5.7.4 Dimensions

Dimensions of RECESSED STEPS shall conform to requirements of:

1) MAHC Table 4.5.7.4,
2) MAHC Figure 4.5.7.4.1, and
3) MAHC Figure 4.5.7.4.2.

Table 4.5.7.4: Recessed Step Dimensions

Dimensions	H-1	H-2	W-1	D-1
Minimum	6 inches *(15.2 cm)*	5 inches *(12.7 cm)*	12 inches *(30.5 cm)*	5 inches *(12.7 cm)*
Maximum	12 inches *(30.5 cm)*	N/A	N/A	N/A

Figure 4.5.7.4.1: Recessed Step Dimensions

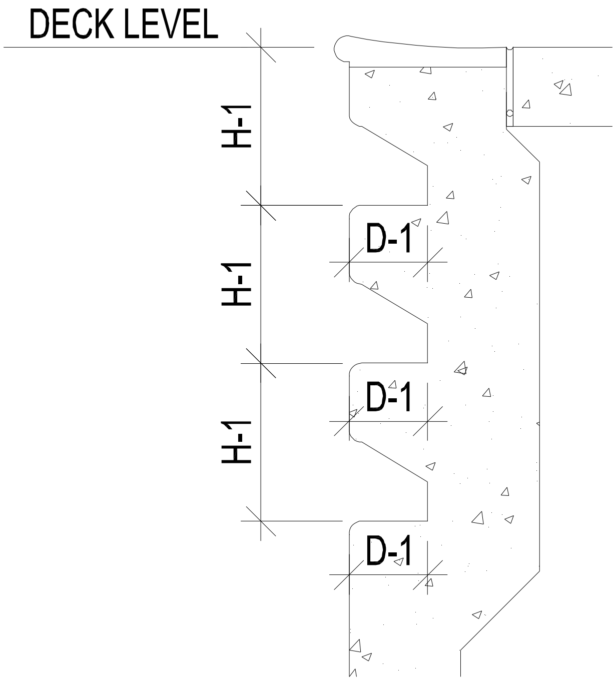

Figure 4.5.7.4.2: Recessed Step Dimensions

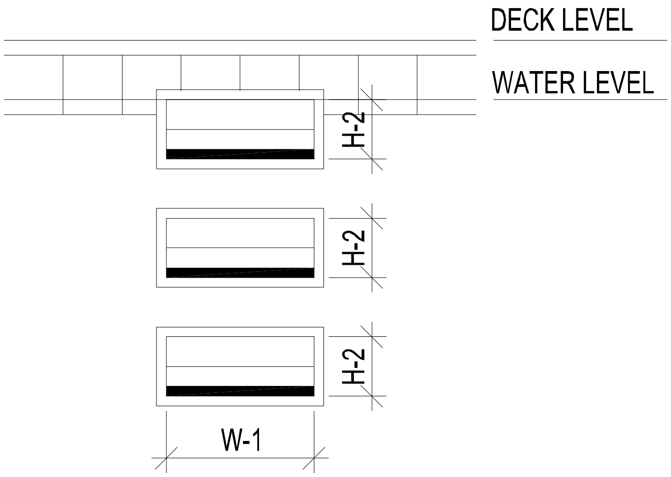

4.5.7.5 Uniformly Spaced

RECESSED STEPS shall be uniformly spaced not less than six inches *(15.2 cm)* and not more than 12 inches *(30.5 cm)* vertically along the POOL wall.

4.5.7.6 Uppermost Step

The top surface of the uppermost RECESSED STEP shall be located not more than 12 inches *(30.5 cm)* below the POOL coping or DECK.

4.5.7.7 Perimeter Gutter Systems

For POOLS with PERIMETER GUTTER SYSTEMS, the gutter may serve as a step, provided that the gutter is provided with a grating or cover and conforms to all construction and dimensional requirements herein specified.

4.5.8 Ladders

4.5.8.1 General Guidelines for Ladders

4.5.8.1.1 Corrosion-Resistant

Where provided, ladders shall be constructed of corrosion-resistant materials.

4.5.8.1.2 Anchored

Ladders shall be anchored securely to the DECK.

4.5.8.2 Ladder Handrails

4.5.8.2.1 Two Handrails Provided

Ladders shall have two handrails.

4.5.8.2.2 Clear Space

The horizontal clear space between handrails shall be not less than 17 inches *(43.2 cm)* and not more than 24 inches *(61.0 cm)*.

4.5.8.2.3 Upper Railing

The upper railing surface of handrails shall extend above the POOL coping or DECK a minimum of 28 inches *(71.7 cm)*.

4.5.8.2.4 Pool Wall

The clear space between handrails and the POOL wall shall be not less than three inches *(7.6 cm)* and not more than six inches *(15.2 cm)*.

4.5.8.2.5 Support

Ladders shall be designed to resist a load of 50 pounds *(22.7 kg)* per linear foot applied in any direction and independently a single concentrated load of 200 pounds *(90.7 kg)* applied in any direction at any location.

4.5.8.2.5.1 Transfer Loads

Ladders shall be designed to transfer these loads through the supports to the POOL or DECK structure.

4.5.8.3 Ladder Treads

4.5.8.3.1 Slip Resistant

Ladder treads shall be slip-resistant.

4.5.8.3.2 Tread Depth

Ladder treads shall have a minimum horizontal tread depth of 1.5 inches *(3.8 cm)* and the distance between the horizontal tread and the POOL wall shall not be greater than four inches *(10.2 cm)*.

4.5.8.3.3 Uniformly Spaced

Ladder treads shall be uniformly spaced not less than seven inches *(17.8 cm)* and not more than 12 inches *(30.5 cm)* vertically at the handrails.

4.5.8.3.4 Upmost Ladder Tread

The top surface of the upmost ladder tread shall be located not more than 12 inches *(30.5 cm)* below the POOL coping, gutter, or DECK.

4.5.9 Zero Depth (Sloped) Entries

4.5.9.1 Slip Resistant

Where ZERO DEPTH ENTRIES are provided, they shall be constructed with slip-resistant materials.

4.5.9.2 Maximum Floor Slope

ZERO DEPTH ENTRIES shall have a maximum floor slope of 1:12, consistent with the requirements of MAHC Section 4.5.2.2.

4.5.9.2.1 Slope Changes

Changes in floor slope shall be permitted.

4.5.9.3 Trench Drains

Trench drains shall be used along ZERO DEPTH ENTRIES at the waterline to facilitate surface skimming.

4.5.9.3.1 Flat or Follow Slope

The trenches may be flat or follow the slope of the ZERO DEPTH ENTRY.

4.5.9.3.2 Handholds

Any handholds that present a trip hazard shall not be continuous along the ZERO DEPTH ENTRY.

4.5.10 Disabled Access

4.5.10.1 Conform to ADA Standards

Access for disabled persons shall conform to ADA Standards as approved by the Department of Justice.

4.5.11 Color and Finish

4.5.11.1 White or Light Pastel

Floors and walls below the water line shall be white or light pastel in color such that from the POOL DECK a BATHER is visible on the POOL floor and the following items can be identified:

1) Algae growth, debris or dirt within the pool, and
2) CRACKS in the surface finish of the POOL, and
3) Marker tiles defined in 4.5.1.2.

4.5.11.1.1 Munsell Color Value

The finish shall be at least 6.5 on the Munsell color value scale.

4.5.11.1.2 Exceptions

An exception shall be made for the following AQUATIC VENUE components:

1) Competitive lane markings,
2) Dedicated competitive diving well floors,
3) Step or bench edge markings,
4) POOLS shallower than 24 inches *(61.0 cm)*,
5) Water line tiles,
6) WAVE POOL and SURF POOL depth change indicator tiles, or
7) Other approved designs.

4.5.11.1.3 Darker Colors

Munsell color values less than 6.5 or designs such as rock formations may be permitted by the AHJ as long as the criteria in MAHC Section 4.5.11.1 are met.

4.5.12 Walls

4.5.12.1 Plumb

POOL walls shall be plumb within a +/- three degree tolerance to a water depth of at least five feet *(1.5 m)*, unless the wall design requires structural support ledges and slopes below to support the upper wall. Refer to MAHC Figure 4.5.12.4.

4.5.12.2 Support Ledges and Slopes

All structural support ledges and slopes of the wall shall fall entirely within a plane slope from the water line at not greater than a +/- three degree tolerance.

4.5.12.2.1 Contrasting Color

A contrasting color shall be provided on the edges of any support ledge to draw attention to the ledge for BATHER SAFETY.

4.5.12.3 Rounded Corners

All corners created by adjoining walls shall be rounded or have a radius in both the vertical and horizontal dimensions to eliminate sharp corners.

4.5.12.4 No Projections

There shall be no projections from a POOL wall with the exception of structures or elements such as stairs, grab rails, ladders, handholds, PENINSULAS, WING WALLS, underwater lights, SAFETY ropes, WATERSLIDES, play features, other approved POOL amenities, UNDERWATER BENCHES, and UNDERWATER LEDGES as described in this section. Refer to MAHC Figure 4.5.12.4.

Figure 4.5.12.4: Pool Walls

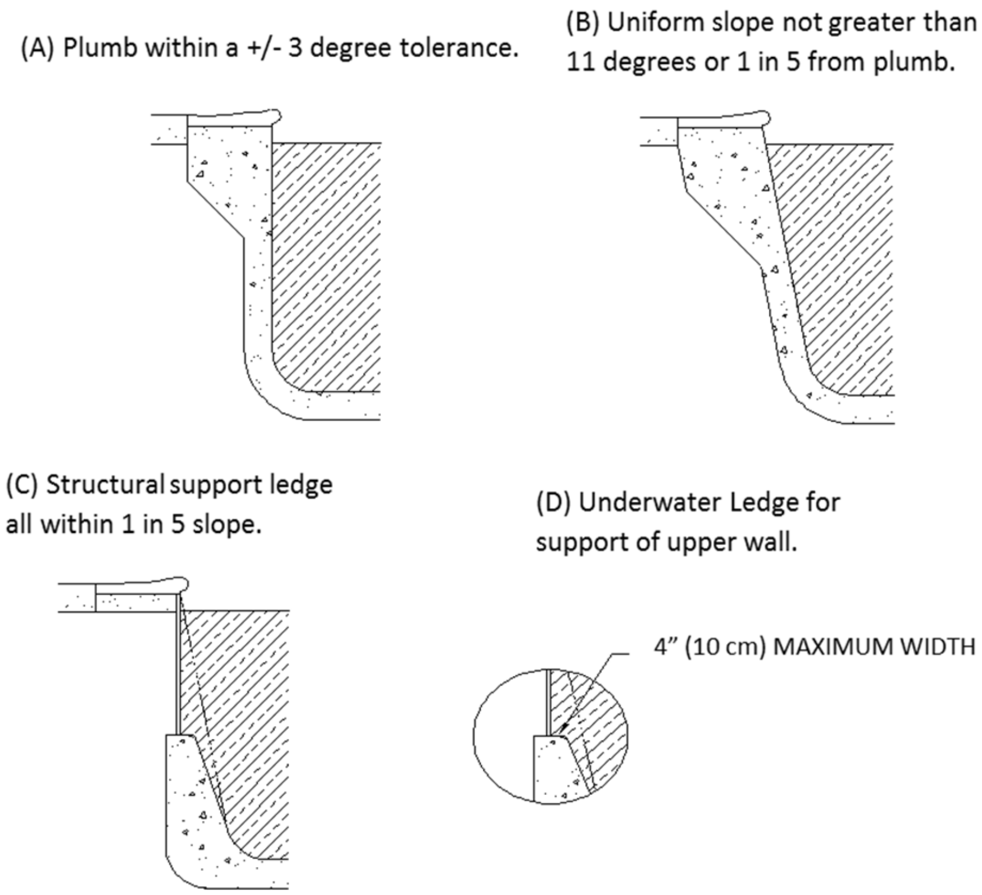

4.5.13 Structural Stability

4.5.13.1 Withstand Loads

POOLS shall be designed to withstand the reasonably anticipated loads imposed by POOL water, BATHERS, and adjacent soils or structures.

4.5.13.2 Hydrostatic Relief Valve

A hydrostatic relief valve and/or suitable under drain system shall be provided where the water table exerts hydrostatic pressure to uplift the pool when empty or drained.

4.5.13.3 Freezing

POOLS and related circulation piping shall be designed with a winterizing strategy when in an area subject to freeze/thaw cycles.

4.5.14 Handholds

4.5.14.1 Handholds Provided

Where not otherwise exempted, every POOL shall be provided with handholds *(perimeter gutter system, coping, horizontal bars, recessed handholds, cantilevered decking)* around the perimeter of the POOL where the water depth at the wall exceeds 24 inches *(61.0 cm)*.

4.5.14.1.1 Installed

These handholds shall be installed not greater than nine inches *(22.9 cm)* above, or three inches *(7.6 cm)* below static water level.

4.5.14.2 Horizontal Recesses

Horizontal recesses may be used for handholds provided they are a minimum of 24 inches *(61.0 cm)* long, a minimum of four inches *(10.2 cm)* high and between two inches *(5.1 cm)* and three inches *(7.6 cm)* deep.

4.5.14.2.1 Drain

Horizontal recesses shall drain into the POOL.

4.5.14.2.2 Consecutive Recesses

Horizontal recesses need not be continuous but consecutive recesses shall be separated by no more than 12 inches *(30.5 cm)* of wall.

4.5.14.3 Decking

Where PERIMETER GUTTER SYSTEMS are not provided, a coping or cantilevered decking of reinforced concrete or material equivalent in strength and durability, with rounded, slip-resistant edges shall be provided.

4.5.14.4 Coping Dimensions

The overhang for coping or cantilevered decking shall not be greater than two inches *(50 mm)* from the vertical plane of the POOL wall, nor less than one inch *(2.5 cm)*.

4.5.14.5 Coping Thickness

The overhang for coping or cantilevered decking shall not exceed 3.5 inches *(8.9 cm)* in thickness for the last two inches *(5.1 cm)* of the overhang.

4.5.15 Infinity Edges

4.5.15.1 Perimeter Restrictions

Not more than fifty percent *(50%)* of the POOL perimeter shall incorporate an INFINITY EDGE detail, unless an adjacent and PATRON accessible DECK space conforming to MAHC Section 4.8.1 is provided.

4.5.15.2 Length

The length of an INFINITY EDGE shall be no more than 30 feet *(9.1 m)* long when in water depths greater than five feet *(1.5 m)*.

4.5.15.2.1 Shallow Water

No maximum distance is enforced for the length of INFINITY EDGES in shallow water five feet *(1.5 m)* and less.

4.5.15.3 Handholds

Handholds conforming to the requirements of MAHC Section 4.5.14 shall be provided for INFINITY EDGES, which may be separate from, or incorporated as part of the INFINITY EDGE detail.

4.5.15.4 Construction Guidelines

Where INFINITY EDGES are provided, they shall be constructed of reinforced concrete or other impervious and structurally rigid material*(s)*, and designed to withstand the loads imposed by POOL water, BATHERS, and adjacent soils or structures.

4.5.15.5 Overflow Basins

Troughs, basins, or capture drains designed to receive the overflow from INFINITY EDGES shall be watertight and free from STRUCTURAL CRACKS.

4.5.15.5.1 Finish

Troughs, basins, or capture drains designed to receive the overflow from INFINITY EDGES shall have a non-toxic, smooth, and slip-resistant finish.

4.5.15.6 Maximum Height

The maximum height of the wall outside of the INFINITY EDGE shall not exceed 30 inches *(76.2 cm)* to the adjacent grade and capture drain.

4.5.16 Underwater Benches

4.5.16.1 Slip Resistant

Where provided, UNDERWATER BENCHES shall be constructed with slip-resistant materials.

4.5.16.2 Outlined Edges

The leading horizontal and vertical edges of UNDERWATER BENCHES shall be outlined with slip-resistant color contrasting tile or other permanent marking of not less than ¾ inch *(1.9 cm)* and not greater than two inches *(5.1 cm)*.

4.5.16.3 Maximum Water Depth

UNDERWATER BENCHES may be installed in areas of varying depths, but the maximum POOL water depth in that area shall not exceed five feet *(1.5 m).*

4.5.16.4 Maximum Seat Depth

The maximum submerged depth of any seat or sitting bench shall be 20 inches *(50.8 cm)* measured from the water line.

4.5.17 Underwater Ledges

4.5.17.1 Slip Resistant

Where UNDERWATER TOE LEDGES are provided to enable swimmers in deep water to rest or to provide structural support for an upper wall, they shall be constructed with slip-resistant materials.

4.5.17.2 Protrude

UNDERWATER TOE LEDGES for resting may be recessed or protrude beyond the vertical plane of the POOL wall, provided they meet the criteria for slip resistance and tread depth outlined in this section.

4.5.17.3 Five Feet or Greater

UNDERWATER TOE LEDGES for resting shall only be provided within areas of a POOL with water depths of five feet *(1.5 m)* or greater.

4.5.17.3.1 Underwater Toe Ledge

UNDERWATER TOE LEDGES must start no earlier than four lineal feet *(1.2 m)* to the deep side of the five foot *(1.5 m)* slope break.

4.5.17.3.2 Below Water Level

UNDERWATER TOE LEDGES must be at least four feet *(1.2 m)* below static water level.

4.5.17.4 Structural Support

UNDERWATER LEDGES for structural support of upper walls are allowed.

4.5.17.5 Outlined

The edges of UNDERWATER TOE LEDGES shall be outlined with slip-resistant color contrasting tile or other permanent marking of not less than one inch *(2.5 cm)* and not greater than two inches *(5.1 cm)*.

4.5.17.5.1 Visible

If they project past the plane of the POOL wall, the edges of UNDERWATER TOE LEDGES shall be clearly visible from the DECK.

4.5.17.6 Tread Depths

UNDERWATER TOE LEDGES shall have a maximum uniform horizontal tread depth of four inches *(10.2 cm)*. See MAHC Figure 4.5.12.4.

4.5.18 Underwater Shelves

4.5.18.1 Immediately Adjacent

UNDERWATER SHELVES may be constructed immediately adjacent to water shallower than five feet *(1.5 m)*.

4.5.18.2 Nosing

UNDERWATER SHELVES shall have a slip-resistant, color contrasting nosing at the leading horizontal and vertical edges on both the top of horizontal edges and leading vertical edges and should be viewable from the DECK or from underwater.

4.5.18.3 Maximum Depth

UNDERWATER SHELVES shall have a maximum depth of 24 inches *(61.0 cm)*.

4.5.19 Depth Markers and Markings

4.5.19.1 Location

4.5.19.1.1 Markings

POOL water depths shall be clearly and permanently marked at the following locations:

1) Minimum depth,
2) Maximum depth,
3) On both sides and at each end of the POOL and,
4) At the break in the floor slope between the shallow and deep portions of the POOL.

4.5.19.1.2 Depth Measurements

Depth markers shall be located on the vertical POOL wall and positioned to be read from within the POOL.

4.5.19.1.3 Below Handhold

Where depth markings cannot be placed on the vertical wall above the water level, other means shall be used so that the markings will be plainly visible to persons in the POOL.

4.5.19.1.4 Coping or Deck

Depth markers shall also be located on the POOL coping or DECK within 18 inches *(45.7 cm)* of the POOL structural wall or perimeter gutter.

4.5.19.1.5 Read on Deck

Depth markers shall be positioned to be read while standing on the DECK facing the POOL.

4.5.19.1.6 Twenty-Five Foot Intervals

Depth markers shall be installed at not more than 25 foot *(7.6 m)* intervals around the POOL perimeter edge and according to the requirements of this section. In addition, for water less than five feet *(1.5 m)* in depth, the depth shall be marked at one foot *(30.5 cm)* depth intervals.

4.5.19.2 Construction / Size

4.5.19.2.1 Durable

Depth markers shall be constructed of a durable material resistant to local weather conditions.

4.5.19.2.2 Slip Resistant

Depth markers shall be slip resistant when they are located on horizontal surfaces.

4.5.19.2.3 Color and Height

Depth markers shall have letters and numbers with a minimum height of four inches *(10. 2 cm)* of a color contrasting with background.

4.5.19.2.4 Feet and Inches

Depth markers shall be marked in units of feet and inches.

4.5.19.2.4.1 Abbreviations

Abbreviations of "FT" and "IN" may be used in lieu of "FEET" and "INCHES."

4.5.19.2.4.1.1 Abbreviations

Symbols for feet *(')* and inches *(")* shall not be permitted on water depth signs.

4.5.19.2.4.2 Metric

Metric units may be provided in addition to—but not in lieu of—units of feet and inches.

4.5.19.3 Tolerance

Depth markers shall be located to indicate water depth to the nearest three inches *(7.6 cm)*, as measured from the POOL floor three feet *(0.9 m)* out from the POOL wall to the gutter lip, mid-point of surface SKIMMER*(s)*, or surge weir*(s)*.

4.5.19.4 No Diving Markers

4.5.19.4.1 Depths

For POOL water depths 5 feet *(1.5 m)* or shallower, all deck depth markers required by MAHC Section 4.5.19 shall be provided with "NO DIVING" warning signs along with the universal international symbol for "NO DIVING"

4.5.19.4.1.1 Spacing

"NO DIVING" warning signs and symbols shall be spaced at no more than 25 foot *(7.6 m)* intervals around the POOL perimeter edge.

4.5.19.4.2 Durable

"NO DIVING" MARKERS shall be constructed of a durable material resistant to local weather conditions.

4.5.19.4.3 Slip Resistant

"NO DIVING" MARKERS shall be slip-resistant when they are located on horizontal surfaces.

4.5.19.4.4 At Least Four Inches

All lettering and symbols shall be at least four inches *(10.2 cm)* in height.

4.5.19.5 Depth Marking At Break in Floor Slope

4.5.19.5.1 Over Five Feet

For POOLS deeper than five feet *(1.5 m)*, a line of contrasting color, not less than two inches *(5.1 cm)* and not more than six inches *(15.2 cm)* in width, shall be clearly and permanently installed on the POOL floor at the shallow side of the break in the floor slope, and extend up the POOL walls to the waterline.

4.5.19.5.2 Durable

Depth marking at break in floor slope shall be constructed of a durable material resistant to local weather conditions and be slip resistant.

4.5.19.5.3 Safety Rope

One foot *(30.5 cm)* to the shallow water side of the break in floor slope and contrasting band, a SAFETY float rope shall extend across the POOL surface with the exception of WAVE POOLS, SURF POOLS, and WATERSLIDE LANDING POOLS.

4.5.19.6 Dual Marking System

Symmetrical AQUATIC VENUE designs with the deep point at the center may be allowed by providing a dual depth marking system which indicates the depth at the wall as measured in MAHC Section 4.5.19.3.1 and at the deep point.

4.5.19.7 Non-Traditional Aquatic Venues

Controlled-access AQUATIC VENUES *(such as activity pool, lazy rivers, and other venues with limited access)* shall only require depth markers on a sign at points of entry.

4.5.19.7.1 Clearly Visible

Depth marker signs shall be clearly visible to PATRONS entering the venue.

4.5.19.7.2 Lettering and Symbols

All lettering and symbols shall be as required for other types of depth markers.

4.5.19.8 Wading Pool Depth Markers

AQUATIC VENUES where the maximum water depth is six inches *(15.2 cm)* of water or less *(such as WADING POOLS and ACTIVITY POOL areas)* shall not be required to have depth markings or “No Diving” signage.

4.5.19.9 Movable Floor Depth Markers

For AQUATIC VENUES with movable floors, a sign indicating movable floor and/or varied water depth shall be provided and clearly visible from the DECK.

4.5.19.9.1 Vertical Measurement

The posted water depth shall be the water level to the floor of the AQUATIC VENUE according to a vertical measurement taken three feet *(0.9 m)* from the AQUATIC VENUE wall.

4.5.19.9.2 Signage

A sign shall be posted to inform the public that the AQUATIC VENUE has a varied depth and refer to the sign showing the current depth.

4.5.19.10 Spas

A minimum of two depth markers shall be provided regardless of the shape or size of the SPA as per MAHC Section 4.12.1.6.

4.5.20 Aquatic Venue Shell Maintenance [N/A]

4.5.21 Special Use Aquatic Venues

4.5.21.1 Adequately Support

The design professional shall provide information to adequately support why the SPECIAL USE AQUATIC VENUE does not meet the definition and use characteristics of other categories of AQUATIC VENUES or POOLS listed in the code.

4.5.21.2 Justification

The design professional shall provide justification for design parameters that do not meet the design standards and construction requirements listed in MAHC Section 4.0.

4.6 Indoor / Outdoor Environment

4.6.1 Lighting

4.6.1.1 General Requirements

4.6.1.1.1 Outdoor Aquatic Venues

Lighting as described in this subsection shall be provided for all outdoor AQUATIC VENUES open for use from 30 minutes before sunset to 30 minutes after sunrise, or during periods of natural illumination below the levels required in MAHC Section 4.6.1.3.1.

4.6.1.1.2 Accessible

No lighting controls shall be accessible to PATRONS or BATHERS.

4.6.1.2 Windows / Natural Light

Where natural lighting methods are used to meet the light level requirements of MAHC Section 4.6.1.3.1 during portions of the day when adequate natural lighting is available, one of the following methods shall be used to ensure that lights are turned on when natural lighting no longer meets these requirements:

1) Automatic lighting controls based on light levels or time of day, or
2) Written operations procedures where manual controls are used.

4.6.1.3 Light Levels

POOL water surface and DECK light levels shall meet the following minimum maintained light levels:

1) Indoor Water Surface: 30 horizontal footcandles *(323 lux)*
2) Outdoor Water Surface: 10 horizontal footcandles *(108 lux)*
3) Deck: 10 horizontal footcandles *(108 lux).*

Note: Higher levels may be advisable for acceptable spectator viewing for competitive swimming and diving events.

4.6.1.4 Overhead Lighting

4.6.1.4.1 Artificial Lighting

Artificial lighting shall be provided at all AQUATIC VENUES which are to be used at night or which do not have adequate natural lighting.

4.6.1.4.2 Aquatic Venue Floor

Lighting shall illuminate all parts of the floor of the AQUATIC VENUE to enable a QUALIFIED LIFEGUARD or other person to determine whether a BATHER is on the floor of the AQUATIC VENUE.

4.6.1.4.3 Aquatic Venue Illumination

Lighting shall illuminate all parts of the AQUATIC VENUE including the water, the depth markers, signs, entrances, restrooms, SAFETY equipment, and the required DECK area and walkways.

4.6.1.5 Underwater Lighting

4.6.1.5.1 Minimum Requirements

Underwater lighting, where provided, shall be not less than eight initial rated lumens per square foot of POOL water surface area.

4.6.1.5.1.1 Location

Such underwater lights, in conjunction with overhead or equivalent DECK lighting, shall be located to provide illumination so that all portions of the AQUATIC VENUE, including the AQUATIC VENUE bottom and drain*(s)*, may be readily seen.

4.6.1.5.1.2 Higher Light Levels

Higher underwater light levels shall be considered for deeper water to achieve this outcome.

4.6.1.5.2 Dimmable Lighting

Dimmable lighting shall not be used for underwater lighting.

4.6.1.6 Night Swimming with No Underwater Lighting

4.6.1.6.1 Minimum Requirements

Where outdoor POOLS are open for use from 30 minutes before sunset to 30 minutes after sunrise, or during periods of low illumination, underwater lighting may be excluded where:

1) Maintained POOL surface lighting levels are a minimum of 15 horizontal footcandles *(161 lux),* and
2) All portions of the POOL, including the bottom and drain*(s)*, are readily visible as required in MAHC Section 5.7.6.1.

4.6.1.7 Emergency Lighting

4.6.1.7.1 Emergency Egress Lighting

POOL areas requiring lighting shall be provided with emergency egress lighting in compliance with the applicable building codes.

4.6.1.7.2 Footcandles

The path of egress shall be illuminated to at least a value of 0.5 footcandles *(5.4 lux)*.

4.6.1.8 Glare

Windows and any other features providing natural light into the POOL space and overhead or equivalent DECK lighting shall be designed or arranged to inhibit or reduce glare on the POOL water surface that would prevent seeing objects on the POOL bottom.

4.6.2 Indoor Aquatic Facility Ventilation

4.6.2.1 Purpose

INDOOR AQUATIC FACILITY AIR HANDLING SYSTEMS shall be designed, constructed, and installed to support the health and SAFETY of the building's PATRONS.

4.6.2.2 Exemptions

INDOOR AQUATIC FACILITY AIR HANDLING SYSTEM design requirements do not apply to AQUATIC FACILITIES that do not meet the definition of a "Building" in the International Building Code 2012.

4.6.2.3 Indoor Aquatic Facility

AIR HANDLING SYSTEM design requirements shall apply to new or SUBSTANTIALLY ALTERED INDOOR AQUATIC FACILITIES including the area of the building's AQUATIC VENUES and the surrounding BATHER and spectator/stadium seating areas.

4.6.2.4 Mechanical Code

INDOOR AQUATIC FACILITY AIR HANDLING SYSTEM design, construction, and installation shall comply with applicable local CODES.

4.6.2.5 ASHRAE 62.1 Compliance

INDOOR AQUATIC FACILITY AIR HANDLING SYSTEM design, construction, and installation shall comply with ASHRAE standard 62.1 2013, *Ventilation for Acceptable Indoor Air Quality*, and/or applicable local CODES with additional requirements as stated in MAHC Section 4.6.2.6.

4.6.2.6 Air Handling System Design

4.6.2.6.1 Mechanical Systems

Ventilation shall be provided through mechanical systems and/or engineered openings for natural ventilation.

4.6.2.6.2 Design Factors and Performance Requirements

The AIR HANDLING SYSTEM design engineer shall provide plan drawings and documentation with the following components showing the design meets the performance requirements per MAHC Section 4.6.2.7:

1) Building layout identifying the location of the INDOOR AQUATIC FACILITY;
2) INDOOR AQUATIC FACILITY size including area in square feet and volume in cubic feet;
3) The area in square feet for DECK and for stadium seating sections;
4) THEORETICAL PEAK OCCUPANCY per AQUATIC VENUE and DECK spaces;
5) Placement of AIR HANDLING SYSTEM and other building outdoor air intakes exterior to the building;
6) Placement of AIR HANDLING SYSTEM and other building exhaust vents exterior to the building;
7) Placement of return air intakes within the INDOOR AQUATIC FACILITY;
8) Placement of supply air locations within the INDOOR AQUATIC FACILITY;
9) Identify system capabilities, if utilized, to automatically or manually modulate the amount of outdoor air for the purposes of reducing the number of cubic feet per minute *(cfm)* of outdoor air when occupancy is lower than THEORETICAL PEAK OCCUPANCY; and
10) Identify system design to maintain negative air pressure in the INDOOR AQUATIC FACILITY relative to the indoor areas external to it.

4.6.2.6.3 Other Air Handling Systems

AIR HANDLING SYSTEM design for CHEMICAL STORAGE SPACES, mechanical, toilet, shower, and dressing rooms are not included in the scope of this section of the CODE, but shall be considered for their effects on the performance requirements of MAHC Section 4.6.2.7 such as maintaining negative pressure, temperature differences, and contribution to the air volume of the INDOOR AQUATIC FACILITY.

4.6.2.6.4 High Volume, Low Speed Fans

AIR HANDLING SYSTEM design may not consider mechanical fans used to push air within the space as part of the outdoor air calculations for the INDOOR AQUATIC FACILITY as defined in MAHC Section 4.6.2.7.

4.6.2.6.4.1 Air Delivery Rate

Mechanical fans used to push air within the space may be used in the calculation for air delivery rate (turnover).

4.6.2.6.5 Occupied and Open All Seasons

AIR HANDLING SYSTEM design may include natural ventilation calculated in accordance with the ASHRAE Handbooks to substitute the corresponding portion of mechanical ventilation only if all the calculated exterior openings will be continuously controlled open during all times the INDOOR AQUATIC FACILITY is occupied, regardless of season.

4.6.2.6.6 Air Distribution Design

The design of the distribution of supply air and distribution of exhaust or return air shall consider obstacles such as support columns, architectural structures, and AQUATIC FEATURES.

4.6.2.7 Performance Requirements for Air Handling Systems

4.6.2.7.1 Minimum Outdoor Air Requirements

The AIR HANDLING SYSTEM shall have a design capability to supply the minimum outdoor air requirements using ASHRAE standard 62.1 2013, *Ventilation for Acceptable Indoor Air Quality*.

4.6.2.7.2 System Alarm

The AIR HANDLING SYSTEM design shall provide system features to notify the operator if the outdoor air flow rate entering the indoor AQUATIC FACILITY is below 0.48 cfm/ft^2.(1.8 *m^3/h).*

4.6.2.7.3 Real-Time Occupancy

Design of the AIR HANDLING SYSTEM shall meet the requirements for the number of cfm/ft^2 based on the THEORETICAL PEAK OCCUPANCY.

4.6.2.7.3.1 Method to Determine

If a method to determine real-time actual occupancy is available, then the system may modulate to reduce outdoor air cubic feet per minute to meet the requirement for the actual occupancy for the associated time frame.

4.6.2.7.4 Air Delivery Rate

The AIR HANDLING SYSTEM shall supply an air delivery rate as defined in ASHRAE Handbook – HVAC Applications 2011, *Places of Assembly, Natatoriums*.

4.6.2.7.5 Consistent Air Flow

INDOOR AQUATIC FACILITY AIR HANDLING SYSTEM shall be designed to provide consistent air flow through all parts of the INDOOR AQUATIC FACILITY to preclude any stagnant areas.

4.6.2.7.6 Relative Humidity

The AIR HANDLING SYSTEM shall maintain the relative humidity in the space as defined in *ASHRAE Handbook: HVAC Applications, 2011, Places of Assembly, Natatoriums*.

4.6.2.7.6.1 Dew Point

The AIR HANDLING SYSTEM shall be designed to maintain the dew point of the interior space less than the dew point of the interior walls at all times so as to prevent damage to structural members and to prevent biological growth on walls.

4.6.2.7.6.2 Condensation & Mold Control

The AIR HANDLING SYSTEM shall be designed to achieve several objectives including maintaining space conditions, delivering the outside air to the breathing area, and to flush the outside walls and windows, which can have the lowest surface temperature and therefore the greatest chance for condensation.

4.6.2.7.7 Negative Air Pressure

AIR HANDLING SYSTEM air flow shall be designed to maintain negative air pressure in the INDOOR AQUATIC FACILITY relative to the areas external to it such as adjacent indoor spaces and outdoor ambient space).

4.6.2.7.8 Disinfection By-Product Removal

Sufficient return air intakes shall be placed near AQUATIC VENUE surfaces such that they remove the highest concentration of airborne DISINFECTION BY-PRODUCT contaminated air.

4.6.2.7.8.1 Airflow Across Water Surface

The AIR HANDLING SYSTEM shall be designed considering airflow across the water surface to promote removal of DISINFECTION BY-PRODUCTS.

4.6.2.7.9 Re-Entrainment of Exhaust

AIR HANDLING SYSTEM outdoor air intakes shall be placed to minimize RE-ENTRAINMENT of exhaust air from building systems back into the facility.

4.6.2.7.9.1 System Exhaust

AIR HANDLING SYSTEM exhaust from CHEMICAL STORAGE SPACES, mechanical, toilet, shower, and dressing rooms shall not be directed into the AQUATIC FACILITY.

4.6.2.7.10 Access Control

The AIR HANDLING SYSTEM shall be designed to provide a means to limit physical or electronic access to system control to the operator and anyone the operator deems to have access.

4.6.2.7.11 Purge

The AIR HANDLING SYSTEM shall have the capability to periodically PURGE air for air quality maintenance or for emergency situations.

4.6.2.7.11.1 Purge Capacity

The AIR HANDLING SYSTEM shall have a PURGE capacity equal or greater than two times the ASHRAE STANDARD 62.1 2013 level.

4.6.2.7.11.1.1 Manual Activation

This PURGE shall be capable of being manually activated.

4.6.2.7.11.2 Outdoor Air

Outdoor air required for PURGE shall not be required to be heated or otherwise treated.

4.6.2.7.12 Air Handling System Filters

The AIR HANDLING SYSTEM design shall include filters for outdoor air and recirculated air with a Minimum Efficiency Reporting Value *(MERV)* rating of eight.

4.6.2.8 Air Handling System Installation

4.6.2.8.1 Air Handling System Procedures

The contractor installing the INDOOR AQUATIC FACILITY AIR HANDLING SYSTEM shall provide the AQUATIC FACILITY owner with an operating manual from the manufacturer which includes:

1) Startup and shutdown procedures;
2) PURGING and other SAFETY procedures;
3) Cleaning procedures;
4) General maintenance requirements with parts listings and frequency of maintenance *(i.e., filter cleaning frequencies, motor bearing maintenance)*;
5) Pressure differential specifications for filter replacement, filter replacement type, and frequency of cleaning or replacement;
6) Troubleshooting processes;
7) Frequency of required calibration of equipment;
8) Descriptions of general operating schemes; and
9) Contact information for the manufacturer.

4.6.2.9 Air Handling System Commissioning

4.6.2.9.1 System Commissioning

A qualified, licensed professional shall commission the AIR HANDLING SYSTEM to verify that the installed system is operating properly in accordance with the system design.

4.6.2.9.2 Written Statement

A written statement of commissioning shall be provided to the AQUATIC FACILITY owner including but not limited to:

1) The number of cubic feet per minute of outdoor air flowing into the INDOOR AQUATIC FACILITY at the time of commissioning;
2) The number of cubic feet per minute of exhaust air flowing through the system at the time of commissioning; and,
3) A statement that the amount of outdoor air meets the performance requirements of MAHC Section 4.6.2.7.

4.6.3 Indoor Aquatic Facility Electrical Systems and Components

4.6.3.1 General Guidelines

4.6.3.1.1 NEC Requirements

Nothing in this CODE shall be construed as providing relief from any applicable requirements of the National Electrical Code *(NEC)* or other applicable CODE.

4.6.3.1.2 Indoor Aquatic Facilities

An INDOOR AQUATIC FACILITY shall be considered a wet and CORROSIVE environment.

4.6.3.2 Electrical Equipment in Interior Chemical Storage Spaces

4.6.3.2.1 Wet and Corrosive

CHEMICAL STORAGE SPACES shall be considered wet and CORROSIVE environments.

4.6.3.2.2 Electrical Conduit

Electrical conduit shall not enter or pass through an interior CHEMICAL STORAGE SPACE, except as required to service devices integral to the function of the room, such as pumps, vessels, controls, lighting and SAFETY devices or, if allowed by the NEC.

4.6.3.2.2.1 Sealed and Inert

Where required, the electrical conduit in an interior CHEMICAL STORAGE SPACE shall be sealed and made of materials that will not interact with any chemicals in the chemical storage space.

4.6.3.2.3 Electrical Devices

Electrical devices or equipment shall not occupy an interior CHEMICAL STORAGE SPACE, except as required to service devices integral to the function of the room, such as pumps, vessels, controls, lighting and SAFETY devices.

4.6.3.2.4 Protected Against Breakage

Lamps, including fluorescent tubes, installed in interior CHEMICAL STORAGE SPACES shall be protected against breakage with a lens or other cover, or be otherwise protected against the accidental release of hot materials.

4.6.4 Pool Water Heating

4.6.4.1 High Temperature

When designing POOL heating equipment, measures shall be taken to prevent BATHER exposure to water temperatures in excess of 104°F *(40°C)*.

4.6.4.2 Pressure Relief Device

Where POOL water heating equipment is installed with valves capable of isolating the heating equipment from the POOL, a listed pressure-relief device shall be installed to limit the pressure on the heating equipment to no more than the maximum value specified by the heating-equipment manufacturer and applicable CODES.

4.6.4.3 Code Compliance

POOL-water heating equipment shall be selected and installed to preserve compliance with the applicable CODES, the terms of listing and labeling of equipment, and with the equipment manufacturer's installation instructions and applicable CODES.

4.6.4.4 Equipment Room Requirements

Where POOL water heaters use COMBUSTION and are located inside a building, the space in which the heater is located shall be considered to be an EQUIPMENT ROOM, and the requirements of MAHC Section 4.9.1 shall apply.

4.6.4.5 Exception

Heaters listed and labeled for the atmosphere shall be acceptable without isolation from chemical fumes and vapors.

4.6.5 First Aid Area

4.6.5.1 Station Design

Design and construction of new AQUATIC FACILITIES shall include an area designated for first aid equipment and/or treatment.

4.6.6 Emergency Exit

4.6.6.1 Labeling

Gates and/or doors which will allow egress without a key shall be clearly and conspicuously labeled in letters at least four inches *(10.2 cm)* high "EMERGENCY EXIT."

4.6.7 Drinking Fountains

4.6.7.1 Provided

A drinking fountain shall be provided inside an AQUATIC FACILITY.

4.6.7.1.1 Alternative

Alternate locations or the use of bottled water shall be evaluated by the AHJ.

4.6.7.1.2 Common Use Area

If the drinking fountain cannot be provided inside the AQUATIC FACILITY, it shall be provided in a common use building or area adjacent to the AQUATIC FACILITY entrance and on the normal path of BATHERS going to the AQUATIC FACILITY entrance.

4.6.7.2 Readily Accessible

The drinking fountain shall be located where it is readily accessible and not a hazard to BATHERS PER MAHC Section 4.10.2.

4.6.7.2.1 Not Located

The drinking fountain shall not be located in a shower area or toilet area.

4.6.7.3 Single Fountain

A single drinking fountain shall be allowed for one or more AQUATIC VENUES within an AQUATIC FACILITY.

4.6.7.4 Angle Jet Type

The drinking fountain shall be an angle jet type installed according to applicable plumbing CODES.

4.6.7.5 Potable Water Supply

The drinking fountain shall be supplied with water from an approved potable water supply.

4.6.7.6 Wastewater

The wastewater discharged from a drinking fountain shall be routed to an approved sanitary sewer system or other approved disposal area according to applicable plumbing CODES.

4.6.8 Garbage Receptacles

4.6.8.1 Sufficient Number

A sufficient number of receptacles shall be provided within an AQUATIC FACILITY to ensure that garbage and refuse can be disposed of properly to maintain safe and sanitary conditions.

4.6.8.2 Number and Location

The number and location of receptacles shall be at the discretion of the AQUATIC FACILITY manager.

4.6.8.3 Closable

Receptacles shall be designed to be closed with a lid or other cover so they remain closed until intentionally opened.

4.6.9 Food and Drink Concessions

4.6.9.1 Meet AHJ Requirements

Concessions for food and drink in an AQUATIC FACILITY shall meet all AHJ requirements.

4.6.10 Spectator Areas

4.6.10.1 Within Aquatic Facility Enclosure

An area designed for use by spectators may be located within an AQUATIC FACILITY ENCLOSURE.

4.6.10.2 Deck

When a spectator area or an access to a spectator area is located within the AQUATIC FACILITY ENCLOSURE, the DECK adjacent to the area or access shall provide egress width for the spectators in addition to the width required by MAHC Section 4.8.1.5.

4.6.10.2.1 Additional Width

The additional width shall be based on the egress requirements in the applicable building CODE based on the THEORETICAL PEAK OCCUPANCY of the AQUATIC FACILITY served with a minimum width of four feet *(1.2 m)* and have either of the following qualities outlined in MAHC Section 4.6.10.2.1.1. or MAHC Section 4.6.10.2.1.2.

4.6.10.2.1.1 Barrier

A BARRIER as defined in MAHC Section 4.8.6.1 located on the DECK to separate the DECK used by spectators from the PERIMETER DECK used by BATHERS.

4.6.10.2.1.1.1 Openings

The BARRIER may have one or more openings directly into the BATHER areas.

4.6.10.2.1.2 Demarcation Line

A demarcation line on the DECK that shows the separation between the DECK used by spectators and the PERIMETER DECK used by BATHERS.

4.6.10.3 Balcony

A spectator or other area located in a balcony within ten feet *(3.0 m)* of or overhanging any portion of an AQUATIC VENUE shall be designed to deter jumping or diving into the AQUATIC VENUE.

4.6.10.4 Bleachers

Bleachers in a spectator area shall be designed according to the International Code Council's most recent version of the 300 Standard or another applicable CODE.

4.7 Recirculation and Water Treatment

4.7.1 Recirculation Systems and Equipment

4.7.1.1 General

4.7.1.1.1 Equipped and Operated

All AQUATIC FACILITIES shall be equipped and operated with a recirculation and filtration system capable of meeting the provisions outlined in MAHC Section 4.7.

4.7.1.1.2 Component Installation

The installation of the recirculation and the filtration system components shall be performed in accordance with the designer's and manufacturer's instructions.

4.7.1.1.3 Recirculation System

A water RECIRCULATION SYSTEM consisting of one or more pumps, pipes, return INLETS, suction outlets, tanks, filters, and other necessary equipment shall be provided.

4.7.1.2 Combined Aquatic Venue Treatment

4.7.1.2.1 Maintain and Measure

When treatment systems of multiple AQUATIC VENUES are combined, the design shall include all appurtenances to maintain and measure the required water characteristics including but not limited to flow rate, pH, and disinfectant concentration in each AQUATIC VENUE or AQUATIC FEATURE.

4.7.1.2.2 Secondary Disinfection

If SECONDARY DISINFECTION is required for an INCREASED RISK AQUATIC VENUE as per MAHC Section 4.7.3.3.1.2, then SECONDARY DISINFECTION shall be required for all treatment systems that are combined with the INCREASED RISK AQUATIC VENUE.

4.7.1.2.3 Isolate

When multiple AQUATIC VENUES are combined in one treatment system, each AQUATIC VENUE shall be capable of being isolated for maintenance purposes.

4.7.1.3 Inlets

4.7.1.3.1 General

4.7.1.3.1.1 Hydraulically Balanced

The RECIRCULATION SYSTEM shall be designed with sufficient flexibility to achieve a HYDRAULIC APPORTIONMENT that will ensure the following:

1) Effective distribution of treated water, and
2) Maintenance of a uniform DISINFECTANT residual and pH throughout the AQUATIC VENUE.

4.7.1.3.1.1.1 Alternative Design Justification
Alternative designs shall be allowed based on adequate engineering justification.

4.7.1.3.1.2 Inlets
Effective distribution of treated water shall be accomplished by either a continuous PERIMETER OVERFLOW system with integral INLETS or by means of directionally adjustable INLETS adequate in design, number, and location.

4.7.1.3.1.3 Adequate Mixing
POOLS shall use wall and/or floor INLETS to provide adequate mixing.

4.7.1.3.1.3.1 Greater Than Fifty Feet Wide
For POOLS greater than 50 feet wide *(15.2 m)*, floor INLETS shall be required.

4.7.1.3.1.4 Other Inlet Types
All other types of INLET systems not covered in this section shall be subject to approval by the AHJ with proper engineering justification.

4.7.1.3.1.5 Hydraulically Sized
INLETS shall be hydraulically sized to provide the design flow rates for each POOL area of multi-zone POOLS based on the required design TURNOVER RATE for each zone.

4.7.1.3.2 Floor Inlets

4.7.1.3.2.1 Uniformly Spaced
Floor INLETS shall be spaced to effectively distribute the treated water throughout the POOL.

4.7.1.3.2.2 Flush with Bottom
Floor INLETS shall be flush with the bottom of the POOL.

4.7.1.3.2.2.1 Distance
Distance between floor INLETS shall be no greater than 20 feet *(6.1 m)*.

4.7.1.3.2.2.2 Row
A row of floor INLETS shall be located within 15 feet *(4.6 m)* of each side wall.

4.7.1.3.2.3 Spaced
Floor INLETS, used in combination with wall INLETS, shall be spaced no greater than 25 feet *(7.6 m)* from nearest side walls.

4.7.1.3.3 Wall Inlets

4.7.1.3.3.1 Effective Mixing
Wall INLET velocity shall mix the water effectively.

4.7.1.3.3.2 Adjustable

INLETS shall be directionally adjustable to provide effective distribution of water.

4.7.1.3.3.3 Inlet Spacing

Wall INLETS shall be spaced no greater than 20 feet *(6.1 m)* apart.

4.7.1.3.3.3.1 Corner

INLETS shall be placed within five feet *(1.5 m)* of each corner of the POOL.

4.7.1.3.3.3.2 Skimmers

INLETS shall be placed at least five feet *(1.5 m)* from a SKIMMER.

4.7.1.3.3.3.3 Isolated

INLETS shall be placed in each recessed or isolated area of the POOL.

4.7.1.3.3.4 Directional Flow

Wall INLETS shall not require design to provide directional flow if part of a manufactured gutter system in which the filtered return water conduit is contained within the gutter structure.

4.7.1.3.3.5 Dye Testing

Dye testing may be required by the AHJ to evaluate the mixing characteristics of the RECIRCULATION SYSTEM.

4.7.1.3.3.5.1 Failed Test

If dye test reveals inadequate mixing in the POOL after 20 minutes, the RECIRCULATION SYSTEM shall be adjusted or modified to assure adequate mixing.

4.7.1.4 Perimeter Overflow Systems/Gutters

4.7.1.4.1 General

4.7.1.4.1.1 Skimming

All POOLS shall be designed to provide SKIMMING for the entire POOL surface area with engineering rationale provided by the design professional.

4.7.1.4.1.1.1 Around Entire Pool

For POOLS that require a perimeter overflow system, the perimeter overflow system shall extend around the entire POOL perimeter except where noted in this CODE.

4.7.1.4.1.2 Zero-Depth Entry

ZERO DEPTH ENTRY POOLS shall have a continuous overflow trench that terminates as close to the side walls as practical including any zero-depth portion of the POOL perimeter.

4.7.1.4.1.2.1 Ends

Where a perimeter overflow system cannot be continuous, the ends of each section shall terminate as close as practical to each other.

4.7.1.4.2 Perimeter Overflow System Size and Shape

4.7.1.4.2.1 Continuous Water Removal

The gutter system shall be designed to allow continuous removal of water from the POOL'S upper surface at a rate of at least 125 percent of the approved total recirculation flow rate chosen by the designer.

4.7.1.4.2.2 Inspection

Gutters shall permit ready inspection, cleaning, and repair.

4.7.1.4.3 Gutter Outlets

Drop boxes, converters, return piping, or FLUMES used to convey water from the gutter shall be designed to:

1) Prevent flooding and BACKFLOW of skimmed water into the POOL, and
2) Handle at least 125 percent of the approved total recirculation flow.

4.7.1.4.4 Surge Tank Capacity

4.7.1.4.4.1 Net Surge Capacity

All perimeter overflow systems shall be designed with an effective net surge capacity of not less than one gallon for each square foot *(40.7 L/m^2)* of POOL surface area.

4.7.1.4.4.1.1 Surge Components

Surge shall be provided within a surge tank, or the gutter or filter above the normal operating level, or elsewhere in the system.

4.7.1.4.4.2 Tank Capacity

The tank capacity specified shall be the net capacity.

4.7.1.4.4.3 Tank Levels

The design professional shall define the minimum, maximum, and normal POOL operating water levels in the surge tank.

4.7.1.4.4.3.1 Marked

The surge tank's minimum, maximum, and normal POOL operating water levels shall be marked on the tank so as to be readily visible for inspection.

4.7.1.4.4.4 Overflow Pipes

Surge tanks, shall have overflow pipes to convey excess water to waste via an air gap or other approved BACKFLOW prevention device.

4.7.1.4.5 Tolerances

Gutters shall be level within a tolerance of plus or minus $^{1}/_{16}$ inch *(1.6 mm)* around the perimeter of the AQUATIC VENUE.

4.7.1.4.6 Makeup Water System

4.7.1.4.6.1 Automatic Makeup

Automatic makeup water supply equipment shall be provided to maintain continuous skimming of POOLS with perimeter overflow systems.

4.7.1.4.6.2 Air Gap

Makeup water shall be supplied through an air gap or other approved BACKFLOW prevention device.

4.7.1.5 Skimmers and Alternative Gutter Technologies Using In-Pool Surge Capacity

4.7.1.5.1 General

4.7.1.5.1.1 Manufactured

The use of manufactured direct suction SKIMMERS shall be in accordance with the manufacturer's recommendations.

4.7.1.5.1.2 Provided

Where SKIMMERS are used, at least one surface SKIMMER shall be provided for each 500 square feet *(46 m^2)* of surface area or fraction thereof.

4.7.1.5.1.2.1 Conditions

Additional SKIMMERS may be required to achieve effective skimming under site-specific conditions *(e.g., heavy winds and/or contaminant loading)* and/or to comply with all applicable building CODES.

4.7.1.5.1.3 Hybrid Systems

Hybrid systems that incorporate surge weirs in the overflow gutters to provide for in-POOL surge shall meet all of the requirements specified for overflow gutters *(with the exception of the surge or balance tank, since the surge capacity requirement will be alternately met by the in-pool surge capacity)*.

4.7.1.5.1.3.1 Surge Weirs

The number of surge weirs shall be based on the individual surge weir capacity and the operational apportionment of the design recirculation flow rate.

4.7.1.5.1.3.1.1 Locations

The location of the required number of surge weirs shall be uniformly spaced in the gutter sections.

4.7.1.5.1.4 Design Capacity

When used, the SKIMMER SYSTEM shall be designed to handle up to 100% of the total recirculation flow rate chosen by the designer.

4.7.1.5.1.5 Pool Width Limitations

POOLS using SKIMMERS shall not exceed 30 feet *(9.1 m)* in width.

4.7.1.5.2 Skimmer Location

4.7.1.5.2.1 Effective

SKIMMERS shall be so located as to provide effective skimming of the entire water surface.

4.7.1.5.2.2 Steps and Recessed Areas

SKIMMERS shall be located so as not to be affected by restricted flow in areas such as near steps and within small recesses.

4.7.1.5.2.3 Wind Direction

Wind direction shall be considered in number and placement of SKIMMERS.

4.7.1.5.3 Skimmer Flow Rate

The flow rate for the SKIMMERS shall comply with manufacturer data plates or NSF/ANSI 50 including Annex K.

4.7.1.5.4 Control

4.7.1.5.4.1 Weir

Each SKIMMER shall have a weir that adjusts automatically to variations in water level over a minimum range of four inches *(10.2 cm)*.

4.7.1.5.4.2 Trimmer Valve

Each SKIMMER shall be equipped with a trimmer valve capable of distributing the total flow between individual SKIMMERS.

4.7.1.5.5 Tolerances

4.7.1.5.5.1 Skimmer Base

The base of each SKIMMER shall be level with all other SKIMMERS in the POOL within a tolerance of plus or minus ¼ inch *(6.4 mm)*.

4.7.1.6 Submerged Suction Outlet

4.7.1.6.1 General

Submerged suction outlets, including sumps and covers, shall listed and labeled to the requirements of ANSI/APSP-16 2011.

4.7.1.6.2 Number and Spacing

4.7.1.6.2.1 Hydraulically Balanced

A minimum of two HYDRAULICALLY BALANCED filtration system outlets are required in the bottom.

4.7.1.6.2.1.1 Located on the Bottom

One of the outlets may be located on the bottom of a side/end wall at the deepest level.

4.7.1.6.2.1.2 Connected

The outlets shall be connected to a single main suction pipe by branch lines piped to provide hydraulic balance between the drains.

4.7.1.6.2.1.3 Valved

The branch lines shall not be valved so as to be capable of operating independently.

4.7.1.6.2.2 Spaced

Outlets shall be equally spaced from the POOL side walls.

4.7.1.6.2.3 Located

Outlets shall be located no less than three feet *(0.9 m)* apart, measuring between the centerlines of the suction outlet covers.

4.7.1.6.3 Tank Connection

Where gravity outlets are used, the main drain outlet shall be connected to a surge tank, collection tank, or balance tank/pipe.

4.7.1.6.4 Flow Distribution and Control

4.7.1.6.4.1 Design Capacity

The main drain system shall be designed at a minimum to handle recirculation flow of 100% of total design recirculation flow rate.

4.7.1.6.4.1.1 Two Main Drain Outlets

Where there are two main drain outlets, the branch pipe from each main drain outlet shall be designed to carry 100% of the recirculation flow rate.

4.7.1.6.4.1.2 Three or More Drains

Where three or more main drain outlets are connected by branch piping in accordance with MAHC Section 4.7.1.6.2.1.1 through MAHC Section 4.7.1.6.2.1.3, the design flow through each branch pipe from each main drain outlet may be as follows:

1) Q_{max} for each drain= Q*(total recirculation rate)* / *(number of drains less one)*, and
2) $Q_{max}=Q_{total}$ / *(N-1)*.

4.7.1.6.4.2 Proportioning Valve

The single main drain suction pipe to the pump shall be equipped with a proportioning valve*(s)* to adjust the flow distribution between the main drain piping and the surface overflow system piping.

4.7.1.6.5 Flow Velocities

4.7.1.6.5.1 Standards

Flow velocities shall meet ANSI/APSP-16 2011 based on 100% design flow through each main drain cover.

4.7.1.7 Piping

4.7.1.7.1 Design

4.7.1.7.1.1 Materials

Piping system components in contact with swimming POOL water shall be of non-toxic material, resistant to corrosion, able to withstand operating pressures, chemicals, and temperatures.

4.7.1.7.1.2 Standards

Piping and piping system component materials shall be suitable for potable water contact and shall be listed and labeled to NSF/ANSI Standard 14, NSF/ANSI Standard 50, and NSF/ANSI Standard 61, as applicable.

4.7.1.7.1.2.1 Certified

Piping and piping system component materials shall be listed and labeled to a specific standard by an ANSI-accredited certification organization.

4.7.1.7.2 Velocity in Pipes

4.7.1.7.2.1 Discharge Piping

RECIRCULATION SYSTEM piping shall be designed so that water velocities do not exceed eight feet *(2.4 m)* per second on the discharge side of the recirculation pump unless alternative values have proper engineering justification.

4.7.1.7.2.2 Suction Piping

Suction piping shall be sized so that the water velocity does not exceed six feet per second *(1.8 m/s)* unless alternative values have proper engineering justification.

4.7.1.7.2.3 Additional Considerations

Gravity piping shall be sized with consideration of available system head or as demonstrated by detailed hydraulic calculations at the design recirculation flow rate.

4.7.1.7.3 Drainage and Installation

4.7.1.7.3.1 Temperature Variations

Provisions shall be made for expansion and contraction of pipes due to temperature variations.

4.7.1.7.3.2 Drainage

Provisions shall be made for complete drainage of all AQUATIC VENUE piping.

4.7.1.7.3.3 Supported

All piping shall be supported continuously or at sufficiently close intervals to prevent sagging and settlement.

4.7.1.7.4 Piping and Component Identification

4.7.1.7.4.1 Clearly Marked

All exposed piping shall be clearly marked to indicate function.

4.7.1.7.4.2 Flow Direction and Source

All piping shall be clearly marked to indicate type or source of water and direction of flow with clear labeling and/or color coding.

4.7.1.7.4.3 Valves

All valves shall be clearly marked to indicate function with clear labeling and/or color coding.

4.7.1.7.4.4 Schematic Displayed

A complete, easily readable schematic of the entire AQUATIC VENUE RECIRCULATION SYSTEM shall be openly displayed in the mechanical room or available to maintenance and inspection personnel.

4.7.1.7.5 Testing

4.7.1.7.5.1 Static Water Pressure Test

Suction and supply POOL piping shall be subjected to a static hydraulic water pressure test for the duration specified by the design engineer and/or AHJ.

4.7.1.7.5.2 Greater

Suction and supply AQUATIC VENUE piping shall be able to maintain the greater of the two following amounts of pressure:

1) 25% greater than the maximum design operating pressure of the system, or
2) 25 psi *(172 KPa)*.

4.7.1.8 Strainers and Pumps

4.7.1.8.1 Strainers

4.7.1.8.1.1 Strainer / Screen

All filter recirculation pumps, except those for vacuum filter installations, shall have a strainer/screen device on the suction side to protect the filtration and pumping equipment.

4.7.1.8.1.2 Materials

All material used in the construction of strainers and screens shall be:

1) Nontoxic, impervious, and enduring,
2) Able to withstand design stresses, and
3) Designed to minimize friction losses.

4.7.1.8.2 Pumping Equipment

4.7.1.8.2.1 Variable Frequency Drives

Variable frequency drives *(VFDs)* may be installed to control all recirculation and feature pumps.

4.7.1.8.2.2 Total Dynamic Head

The recirculation pump*(s)* shall have adequate capacity to meet the recirculation flow design requirements in accordance with the maximum total dynamic head required by the entire RECIRCULATION SYSTEM under the most extreme operating conditions *(e.g., clogged filters in need of backwashing)*.

4.7.1.8.2.3 Required Flow Rate

The pump shall be designed to maintain design recirculation flows under all conditions.

4.7.1.8.2.4 Vacuum Limit Switches

Where vacuum filters are used, a vacuum limit switch shall be provided on the pump suction line.

4.7.1.8.2.5 Maximum

The vacuum limit switch shall be set for a maximum vacuum of 18 inches *(45.7 cm)* of mercury.

4.7.1.8.2.6 Pump Priming

All recirculation pumps shall be self-priming or flooded-suction.

4.7.1.8.2.7 Net Positive Suction Head (NPSH) Requirement

All recirculation pumps shall meet the minimum NPSH requirement for the system.

4.7.1.8.3 Operating Gauges

4.7.1.8.3.1 Vacuum Gauge

A compound vacuum-pressure gauge shall be installed on the pump suction line as close to the pump as possible.

4.7.1.8.3.2 Suction Lift

A vacuum gauge shall be used for pumps with suction lift.

4.7.1.8.3.3 Installed

A pressure gauge shall be installed on the pump discharge line adjacent to the pump.

4.7.1.8.3.4 Easily Read

Gauges shall be installed so they can be easily read.

4.7.1.8.3.5 Valves

All gauges shall be equipped with valves to allow for servicing under operating conditions.

4.7.1.9 Flow Measurement and Control

4.7.1.9.1 Flow Meters

A flow meter accurate to within +/- 5% of the actual design flow shall be provided for each filtration system.

4.7.1.9.1.1 Listed and Labeled

Flow meters shall be listed and labeled to NSF/ANSI Standard 50 by an ANSI-accredited certification organization.

4.7.1.9.2 Valves

All pumps shall be installed with a manual adjustable discharge valve to provide a backup means of flow control as well as for system isolation.

4.7.1.10 Flow Rates / Turnover Times

Table 4.7.1.10: Aquatic Venue Maximum Allowable Turnover Times

Type of Aquatic Venue	Turnover Maximum
Activity Pools	2 hours or less
Diving Pools	8 hours or less
Interactive Play*	0.5 hours or less
Lazy River	2 hours or less
Plunge Pools	1 hour or less
Runout Slide	1 hour or less
Wading Pools*	1 hour or less
Wave Pools	2 hours or less
All Other Pools	6 hours or less
*Shall have secondary disinfection systems	

Spa, Therapy*, & Exercise Pools		
Temperatures	Load	Turnover Maximum
≤ 72°-93°F (22°-34°C)	> 2500 gals/person (9.46 m³)	4 hours or less
≤ 72°-93°F (22°-34°C)	> 450 gals/person (1.7 m³)	2 hours or less
≤ 72°-93°F (22°-34°C)	≤ 450 gals/person (1.7 m³)	1 hour or less
≥ 93-104°F (34°-40°C)	All	0.5 hours or less
*Shall have secondary disinfection systems		

4.7.1.10.1 Maximum Allowable

All AQUATIC VENUES shall comply with the above maximum allowable TURNOVER times shown in MAHC Table 4.7.1.10.

4.7.1.10.2 Calculated

The TURNOVER time shall be calculated based on the total volume of water divided by the flow rate through the filtration process.

4.7.1.10.2.1 Unfiltered Water

Unfiltered water such as water that may be withdrawn from and returned to the AQUATIC VENUE for such AQUATIC FEATURES as slides by a pump separate from the filtration system, shall not factor into TURNOVER time.

4.7.1.10.3 Turnover Variance

The AHJ may grant a TURNOVER time variance for AQUATIC VENUES with extreme volume or operating conditions based on proper engineering justification.

4.7.1.10.4 Turnover Times

TURNOVER times shall be calculated based solely on the flow rate through the filtration system.

4.7.1.10.4.1 Required

The required TURNOVER time shall be the lesser of the following options:

1) The specified time in MAHC Table 4.7.1.10, or
2) The time required for individual components *(e.g., three skimmers with flow rates set by the manufacturer and an additional 20% for the main drains could exceed the minimum value in the table)*.

4.7.1.10.4.2 Total Volume

The total volume of the AQUATIC VENUE system shall include the AQUATIC VENUE and any surge/balance tank.

4.7.1.10.4.3 Supply Water

Where water is drawn from the AQUATIC VENUE to supply water to AQUATIC FEATURES *(e.g., slides, tube rides)*, the water may be reused prior to filtration provided the DISINFECTANT and pH levels of the supply water are maintained at required levels.

4.7.1.10.5 Reuse Ratio

The ratio of INTERACTIVE WATER PLAY AQUATIC VENUE FEATURE water to filtered water shall be no greater than 3:1 in order to maintain the efficiency of the FILTRATION SYSTEM.

4.7.1.10.6 Flow Turndown System

For AQUATIC FACILITIES that intend to reduce the recirculation flow rate below the minimum required design values when the POOL is unoccupied, the flow turndown system shall be designed as follows in MAHC Section 4.7.1.10.6.1 through MAHC Section 4.7.1.10.6.2.

4.7.1.10.6.1 Flowrate

The system flowrate shall not be reduced more than 25% lower than the minimum design requirements and only reduced when the AQUATIC VENUE is unoccupied.

4.7.1.10.6.1.1 Clarity

The system flowrate shall be based on ensuring the minimum water clarity required under MAHC Section 5.7.6 is met before opening to the public.

4.7.1.10.6.1.2 Disinfectant Levels

The turndown system shall be required to maintain required DISINFECTANT and pH levels at all times.

4.7.1.10.6.2 Increase

When the turndown system is also used to intelligently increase the recirculation flow rate above the minimum requirement *(e.g., in times of peak use to maintain water quality goals more effectively)*, the following requirements shall be met at all times:

1) Velocity requirements inside of pipes *(per MAHC Section 4.7.1.7.2)*, and
2) Maximum filtration system flows.

4.7.2 Filtration

4.7.2.1 All Filters

4.7.2.1.1 Required

Filtration shall be required for all AQUATIC VENUES that recirculate water.

4.7.2.2 Granular Media Filters

4.7.2.2.1 General

4.7.2.2.1.1 Valves and Piping

The granular media filter system shall have valves and piping to allow isolation, venting, complete drainage *(for maintenance or inspections)*, and backwashing of individual filters.

4.7.2.2.1.2 Filtration Accessories

Filtration accessories shall include the following items:

1) Influent pressure gauge,
2) Effluent pressure gauge ,
3) Backwash sight glass or other means to view backwash water clarity, and
4) Manual air relief system.

4.7.2.2.1.3 Listed

All filters shall be listed and labeled to NSF/ANSI 50 by an ANSI-accredited certification organization.

4.7.2.2.2 Filter Location and Spacing

4.7.2.2.2.1 Installed

Filters shall be installed with adequate clearance and facilities for ready and safe inspection, maintenance, disassembly, and repair.

4.7.2.2.2.2 Media Removal

A means and access for easy removal of filter media shall be required.

4.7.2.2.3 Filtration and Backwashing Rates

4.7.2.2.3.1 Operate

High-rate granular media filters shall be designed to operate at no more than 15 gallons per minute per square foot (37 m/h) when a minimum bed depth of 15 inches *(38.1 cm)* is provided per manufacturer.

4.7.2.1.3.1.1 Less than Fifteen Inch Bed Depth

When a bed depth is less than 15 inches *(38.1 cm)*, filters shall be designed to operate at no more than 12 gallons per minute per square foot (29 m/h).

4.7.2.2.3.2 Backwash System Design

The granular media filter system shall be designed to backwash each filter at a rate of at least 15 gallons per minute per square foot (37 m/h) of filter bed surface area, unless explicitly prohibited by the filter manufacturer and approved at an alternate rate as specified in their NSF/ANSI 50 listing.

4.7.2.2.4 Minimum Filter Media Depth Requirements

The minimum depth of filter media cannot be less than the depth specified by the manufacturer.

4.7.2.2.5 Differential Pressure Measurement Gauges

Influent and effluent pressure gauges shall have the capability to measure up to a 20 pounds per square inch *(138 KPa)* increase in the differential pressure across the filter bed in increments of one pound per square inch *(6.9 KPa)* or less.

4.7.2.2.6 Coagulant Injection Equipment Installation

If coagulant feed systems are used, they shall be installed with the injection point located before the filters as far ahead as possible, with electrical interlocks in accordance with MAHC Section 4.7.3.2.1.3.

4.7.2.3 Precoat Filters

4.7.2.3.1 General

4.7.2.3.1.1 Listed

All precoat, filters *(i.e., pressure and vacuum)* shall be listed and labeled to NSF/ANSI 50 by an ANSI-accredited certification organization.

4.7.2.3.1.2 Appropriate Media

Filters should be used with the appropriate filter media as recommended by the filter manufacturer for maximum clarity and cycle length for AQUATIC VENUE use.

4.7.2.3.1.2.1 Listed, Labeled, and Sized

Filter media shall be listed and labeled to NSF/ANSI Standard 50 by an ANSI-accredited certification organization and within the size specifications provided by the filter manufacturer and NSF/ANSI 50.

4.7.2.3.1.2.2 Alternate Types

Alternate types of filter media shall be permitted in accordance with the filter manufacturer's recommendation for AQUATIC VENUE use.

4.7.2.3.1.2.3 NSF Standard

Alternate types of filter media shall be listed and labeled to NSF Standard 50 by and ANSI-accredited certification organization.

4.7.2.3.2 Filtration Rates

4.7.2.3.2.1 Vacuum Precoat

The design filtration rate for vacuum precoat filters shall not be greater than either:

1) 2 gallons per minute per square foot *(4.9 m/h)*, or
2) 2.5 gallons per minute per square foot *(6.1 m/h)* when used with a continuous precoat media feed *(commonly referred to as "body-feed")*.

4.7.2.3.2.2 Pressure Precoat

The design filtration rate for pressure precoat filters shall not be greater than 2 gallons per minute per square foot *(4.9 m/h)* of effective filter surface area.

4.7.2.3.2.3 Calculate

The filtration surface area shall be based on the outside surface area of the media with the manufacturer's recommended thickness of precoat media and consistent with their NSF/ANSI 50 listing and labeling.

4.7.2.3.3 Precoat Media Introduction System Process

The precoat process shall follow the manufacturer's recommendations and requirements of NSF/ANSI Standard 50.

4.7.2.3.4 Continuous Filter Media Feed Equipment

4.7.2.3.4.1 Manufacturer Specification

If equipment is provided for the continuous feeding of filter media to the filter influent, the equipment shall be used in accordance with the manufacturer's specifications.

4.7.2.3.4.2 Filter Media Discharge

All discharged filter media shall be handled in accordance with local and state laws, rules, and regulations.

4.7.2.4 Cartridge Filters

4.7.2.4.1 Listed

Cartridge filters shall be installed in accordance with the filter manufacturer's recommendations and listed and labeled to NSF/ANSI 50 by an ANSI-accredited certification organization.

4.7.2.4.2 Filtration Rates

The design filtration rate for surface-type cartridge filter shall not exceed 0.30 gallons per minute per square foot *(0.20 $L/s/m^2$).*

4.7.2.4.3 Supplied and Sized

Filter cartridges shall be supplied and sized in accordance with the filter manufacturer's recommendation for AQUATIC VENUE use.

4.7.2.4.4 Spare Cartridge

One complete set of spare cartridges shall be maintained on site in a clean and dry condition.

4.7.3 Disinfection and pH Control

4.7.3.1 Chemical Addition Methods

4.7.3.1.1 Disinfection and pH

DISINFECTION and pH control chemicals shall be automatically introduced through the RECIRCULATION SYSTEM.

4.7.3.1.1.1 Controller Used

A chemical controller, as specified in MAHC Section 4.7.3.2.8 shall be provided and used for MONITORING and control of DISINFECTANT and pH feed equipment.

4.7.3.1.1.2 Feeder

DISINFECTION and pH control chemicals shall be added using a feeder that meets the requirements outlined in MAHC Section 4.7.3.2.

4.7.3.2 Feed Equipment

4.7.3.2.1 General

4.7.3.2.1.1 Required

Chemical feeders shall be required upon adoption of this CODE.

4.7.3.2.1.2 Feeders & Devices

The AQUATIC FACILITY shall be equipped with chemical feed equipment such as flow-through chemical feeders, electrolytic chemical generators, mechanical chemical feeders, chemical feed pumps, and AUTOMATED CONTROLLERS that are listed and labeled to NSF-ANSI 50 by an ANSI-accredited certification organization.

4.7.3.2.1.3 Interlock Controls and No or Low Flow Deactivation

All chemical feeders shall be provided with an automatic means to be disabled through an electrical interlock with at least two of the following:

1) Recirculation pump power,
2) Flow meter/flow switch in the return line,
3) Chemical control power and paddle wheel or flow cell on the chemical controller ***if*** safety test confirms feed systems are disabled through the controller when the pump is turned off, loses prime, or filters are backwashed.

4.7.3.2.1.4 Installation

The chemical feeders shall be installed according to the manufacturer's instructions.

4.7.3.2.1.4.1 Protective Cover

A physical BARRIER shall be installed between chemical feed pumps supplying acid or liquid hypochlorite solution and other POOL components to shield staff and equipment from chemical sprays from leaking connections.

4.7.3.2.2 Sizing of Disinfection Equipment

4.7.3.2.2.1 Sizing

Feeders shall be capable of supplying DISINFECTANT and pH control chemicals to the AQUATIC VENUE to maintain the minimum required DISINFECTION levels at all times in accordance with the MAHC.

4.7.3.2.2.2 Chlorine Dosing

All CHLORINE dosing and generating equipment including erosion feeders, or in line electrolytic and brine/batch generators, shall be designed with a capacity to provide the following:

1) Outdoor AQUATIC VENUES *(unstabilized)*: 4.0 lbs of FAC/day/10,000 gal *(1.8 kg FAC/day/37,854 L)* of POOL water;
2) Indoor AQUATIC VENUES *(unstabilized)*: 2.5 lbs FAC/day/10,000 gal *(1.1 kg FAC/day/37,854 L)* of POOL water.

4.7.3.2.2.3 Rates

The rates above are suggested minimums and in all cases the engineer shall validate the feed and production equipment specified.

4.7.3.2.3 Introduction of Chemicals

4.7.3.2.3.1 Separation

The injection point of disinfection chemicals shall be located before any pH control chemical injection point with sufficient physical separation of the injection points to reduce the likelihood of mixing of these chemicals in the piping during periods of interruption of recirculation system flow.

4.7.3.2.3.2 Backflow

Means of injection shall not allow BACKFLOW into the chemical system from the POOL system.

4.7.3.2.3.3 Coagulants

Coagulants shall be metered and injected through a pump system prior to the filters per the manufacturer's recommended rate.

4.7.3.2.4 Compressed Chlorine Gas

4.7.3.2.4.1 Prohibited for New Construction

Use of compressed CHLORINE gas shall be prohibited for new construction and after SUBSTANTIAL ALTERATION to existing AQUATIC FACILITIES.

4.7.3.2.4.2 In Existing Aquatic Facilities

Use of compressed chlorine gas in existing AQUATIC FACILITIES is covered in MAHC Section 4.9.2.11.

4.7.3.2.5 Types of Feeders

4.7.3.2.5.1 Liquid Solution Feeders

Liquid solution feeders shall include positive displacement pumps such as peristaltic pumps, diaphragm pumps, and piston pumps.

4.7.3.2.5.1.1 Feed Rates

Feed rates shall be locally adjusted on the pumps and also on/off controlled using an AUTOMATED CONTROLLER.

4.7.3.2.5.2 Erosion

Erosion feeders may be pressure, pressure differential, or spray erosion types.

4.7.3.2.5.2.1 Dry Chemical Feeders

Dry chemicals shall be granules or tablets.

4.7.3.2.5.2.2 Located

Feeders shall have isolation valves on each side of the feeder to be closed before opening the unit.

4.7.3.2.5.2.3 Source Water

Erosion feeders shall use AQUATIC VENUE water post-filtration as the source water unless approved by the feeder manufacturer.

4.7.3.2.5.3 Gas Feed Systems

Carbon dioxide and ozone are the only gas feed systems permitted in AQUATIC FACILITIES.

4.7.3.2.5.4 Ventilation

Proper ventilation shall be required for all gas systems.

4.7.3.2.5.5 Alarms

Where CO_2 cylinders are located indoors, a monitor and alarm shall be provided to alert PATRONS/operator of high CO_2 and/or low O_2 levels.

4.7.3.2.5.6 UV Systems

Where used, ultraviolet light *(UV)* systems shall be installed in the RECIRCULATION SYSTEM after the filters.

4.7.3.2.5.6.1 Bypass

A bypass pipe that is valved on both ends shall be installed to allow maintenance on the UV unit while the POOL is in operation.

4.7.3.2.5.6.2 Interlock

UV system operation shall be interlocked with the recirculation pump so that power to the UV system is interrupted when there is no water flow to the UV unit per MAHC Section 4.7.3.2.1.3.

4.7.3.2.6 Salt Electrolytic Chlorine Generators, Brine Electrolytic Chlorine, or Bromine Generators

4.7.3.2.6.1 Salt Electrolytic Chlorine Generators

In-line generator*(s)* or brine *(batch)* generator*(s)* shall be permitted on AQUATIC VENUES.

4.7.3.2.6.2 In-line Method

In-line generators shall use POOL-grade salt dosed into the AQUATIC VENUE to produce and introduce CHLORINE into the AQUATIC VENUE treatment loop through an electrolytic chamber.

4.7.3.2.6.3 Batch Method

Brine *(Batch)* generators shall produce CHLORINE through an electrolytic cell.

4.7.3.2.6.3.1 Chlorine Production

CHLORINE shall be produced from brines composed of POOL-grade salt.

4.7.3.2.6.4 TDS Readout

Electrolytic generators shall have a TDS or salt *(NaCl)* readout and a low salt indicator.

4.7.3.2.6.5 Feed Rate

The feed rate shall be adjustable from zero *(0)* to full range.

4.7.3.2.6.6 UL Standard

The generator unit shall be listed and labeled to UL 1081 *(for electrical/fire/shock safety)* by an ANSI-accredited certification organization.

4.7.3.2.6.7 Interlock

The generator*(s)* shall be interlocked per MAHC Section 4.7.3.2.1.3.

4.7.3.2.6.8 Installed

The generator units shall be installed according to the manufacturer's instructions.

4.7.3.2.6.8.1 Saline Content

The saline content of the POOL water shall be maintained in the required range specified by the manufacturer.

4.7.3.2.7 Feeders for pH Adjustment

4.7.3.2.7.1 Provided

Feeders for pH adjustment shall be provided on all AQUATIC VENUES upon adoption of this CODE as in MAHC Section 4.7.3.2.1.2.

4.7.3.2.7.2 Approved Substances

Approved substances for pH adjustment shall include but not be limited to muriatic *(hydrochloric)* acid, sodium bisulfate, carbon dioxide, sulfuric acid, sodium bicarbonate, and soda ash.

4.7.3.2.7.3 Adjustable

pH adjustment feeders shall be adjustable from zero *(0)* to full range.

4.7.3.2.7.4 Marked

Reservoirs shall be clearly marked and labeled with contents.

4.7.3.2.8 Automated Controllers

4.7.3.2.8.1 Required

AUTOMATED CONTROLLERS shall be installed for MONITORING and turning on or off chemical feeders used for pH and disinfectants at all AQUATIC VENUES.

4.7.3.2.8.1.1 Installed

AUTOMATED CONTROLLERS shall be required within one year from adoption of this CODE.

4.7.3.2.8.2 NSF Standard

All automated chemical controllers for pH and disinfectant MONITORING/control shall be listed and labeled to NSF/ANSI 50 by an ANSI-accredited certification organization.

4.7.3.2.8.3 Operation Manuals

Operation manuals or other instructions that give clear directions for cleaning and calibrating AUTOMATED CONTROLLER probes and sensors shall be provided in close proximity to the AUTOMATED CONTROLLER.

4.7.3.2.8.4 Set Point

A set point shall be used to target the disinfectant level and the pH level.

4.7.3.3 Secondary Disinfection Systems

4.7.3.3.1 General Requirements

4.7.3.3.1.1 ANSI Listing and Labeling

SECONDARY DISINFECTION SYSTEMS shall be listed and labeled to ANSI/NSF 50 by an ANSI-accredited certification organization approved by the AHJ.

4.7.3.3.1.2 Required Facilities

The new construction or SUBSTANTIAL ALTERATION of the following INCREASED RISK AQUATIC VENUES shall be required to use a SECONDARY DISINFECTION SYSTEM after adoption of this CODE:

1) AQUATIC VENUES designed primarily for children under 5 years old, such as
    a. WADING POOLS,
    b. INTERACTIVE WATER PLAY VENUES with no standing water, and
2) THERAPY POOLS.

4.7.3.3.1.3 Other Aquatic Venues

Optional SECONDARY DISINFECTION SYSTEMS may be installed on other AQUATIC VENUES not specified in MAHC Section 4.7.3.3.1.2.

4.7.3.3.1.4 Labeled

If installed and labeled as SECONDARY DISINFECTION SYSTEMS, then they shall conform to all requirements specified under MAHC Section 4.7.3.3.

4.7.3.3.1.5 Conform

If not labeled as SECONDARY DISINFECTION SYSTEMS, then they shall be labeled as SUPPLEMENTAL TREATMENT SYSTEMS and conform to requirements listed under MAHC Section 4.7.3.4.

4.7.3.3.2 3-log Inactivation and Oocyst Reduction

4.7.3.3.2.1 3-log Inactivation

SECONDARY DISINFECTION SYSTEMS shall be designed to achieve a minimum 3-log *(99.9%)* reduction in the number of infective *Cryptosporidium parvum* OOCYSTS per pass through the SECONDARY DISINFECTION SYSTEM.

4.7.3.3.2.2 *Installation*

The SECONDARY DISINFECTION SYSTEM shall be located in the treatment loop *(post filtration)* and treat a portion *(up to 100%)* of the filtration flow prior to return of the water to the AQUATIC VENUE or AQUATIC FEATURE.

4.7.3.3.2.3 Manufacturer's Instructions

The SECONDARY DISINFECTION SYSTEM shall be installed according to the manufacturer's directions.

4.7.3.3.2.4 Minimum Flow Rate Calculation

The flow rate *(Q)* through the SECONDARY DISINFECTION SYSTEM shall be determined based upon the total volume of the AQUATIC VENUE or AQUATIC FEATURE *(V)* and a prescribed dilution time *(T)* for theoretically reducing the number of assumed infective *Cryptosporidium* OOCYSTS from an initial total number of 100 million *(10^8)* OOCYSTS to a concentration of one OOCYST/100 mL.

4.7.3.3.2.5 Equation

Accounting for a 3 log *(99.9%)* reduction of infective *Cryptosporidium* OOCYSTS through the SECONDARY DISINFECTION SYSTEM with each pass, the SECONDARY DISINFECTION SYSTEM flow rate *(Q)* shall be:

$$Q = V \times \{[14.8 - \ln(V)] / (60 \times T)\}$$, where:

- Q = SECONDARY DISINFECTION SYSTEM flow rate *(gpm)*
- V = Total water volume of the AQUATIC VENUE or AQUATIC FEATURE, including surge tanks, piping, equipment, etc. *(gals)*
- T = Dilution time *(hrs.)*

4.7.3.3.2.6 Time for Dilution Reduction

The dilution time shall be the lesser of nine hours or 75% of the uninterrupted time an AQUATIC VENUE is closed in a 24 hour period.

4.7.3.3.2.7 Flow Rate Measurements

Where a SECONDARY DISINFECTION SYSTEM is installed, a means shall be installed to confirm the required flow rate to maintain a minimum 3 log *(99.9%)* reduction of infective *Cryptosporidium* OOCYSTS at the minimum flow rate.

4.7.3.3.2.7.1 Flow Rate Defined

The minimum required flow rate through the SECONDARY DISINFECTION SYSTEM shall be as defined in MAHC Section 4.7.3.3.2.5.

4.7.3.3.3 Ultraviolet Light Systems

4.7.3.3.3.1 Third Party Validation

UV equipment shall be third party validated in accordance with the practices outlined in the *US EPA Ultraviolet Disinfectant Guidance Manual* dated November, 2006, publication number EPA 815-R-06-007.

4.7.3.3.3.1.1 Validation Standard

The *US EPA Ultraviolet Disinfectant Guidance Manual* shall be considered a recognized national STANDARD in the MAHC.

4.7.3.3.3.2 Suitable for Intended Use

UV systems and all materials used therein shall be suitable for their intended use and be installed:

1) In accordance with the MAHC,
2) As listed and labeled to a specific standard by an ANSI-accredited certification organization, and
3) As specified by the manufacturer.

4.7.3.3.3.3 Installation

The UV equipment shall be installed after the filtration and before addition of primary DISINFECTANT.

4.7.3.3.3.3.1 Labeled

UV equipment shall be labeled with the following design specifications: maximum flow rate, minimum transmissivity, minimum intensity, and minimum dosage.

4.7.3.3.3.3.2 Strainer Installation

An inline strainer shall be installed after the UV unit to capture broken lamp glass or sleeves.

4.7.3.3.3.4 Electronically Interlocked

The equipment shall be electrically interlocked with feature pump*(s)* or automated feature supply valves, such that when the UV equipment fails to produce the required dosage as measured by automated sensor, the water features do not operate.

4.7.3.3.3.4.1 Operation

UV systems shall not operate if the RECIRCULATION SYSTEM is not operating.

4.7.3.3.3.5 Calibrated UV Sensors

The UV equipment shall be complete with calibrated UV sensors, which record the output of all the UV lamps installed in a system.

4.7.3.3.3.5.1 Multiple Lamps

Where multiple lamps are fitted, sufficient sensors shall be provided to measure each lamp.

4.7.3.3.3.5.2 Fewer Sensors

If the design utilizes fewer sensors than lamps, the location of lamps and sensors shall be such that the output of all lamps is adequately measured.

4.7.3.3.3.6 Equipment Audit

In order to ensure that equipment supplied meets all the requirements of the STANDARD the manufacturer shall maintain a quality assurance system audited on a regular basis to a recognized quality STANDARD.

4.7.3.3.3.6.1 Accreditation

An ISO9000:2000 accreditation or listing to NSF Standard 50 are both acceptable methods of meeting this equipment requirement.

4.7.3.3.3.7 Automated Shut Down

The automated shut down of the UV equipment for any reason shall initiate a visual alarm or other indication which will alert staff on-site or remotely.

4.7.3.3.3.7.1 Signage

Signage instructing staff or PATRONS to notify facility management shall be posted adjacent to the visual indication.

4.7.3.3.3.7.2 Not Staffed

If the AQUATIC FACILITY is not staffed, the sign shall include a means to contact management whenever the AQUATIC FACILITY is in use.

4.7.3.3.3.8 Reports and Documentation

The UV equipment shall be supplied with the appropriate validation reports and documentation for that equipment model.

4.7.3.3.3.9 Manufacturer 3-log Inactivation Chart

This documentation will include a graph or chart indicating the dose at which a 3-log inactivation is guaranteed for the system in question.

4.7.3.3.3.9.1 RED Bias

This dose shall be inclusive of validation factors and REDUCTION EQUIVALENT DOSE *(RED)* BIAS.

4.7.3.3.3.9.2 System Performance Curves

System performance curves that do not include such factors are not considered validated systems.

4.7.3.3.3.10 Minimum RED

Validation records shall include the graph indicating the minimum intensity reading required at the operational flow for the minimum RED required to achieve 3-log reduction.

4.7.3.3.3.10.1 Minimum Intensity Shown

Where systems are validated to a specific dose, the graph shall show the minimum intensity reading required at the operational flow for that dose.

4.7.3.3.3.11 Recommended Validation Protocol

Based on the recommended validation protocol presented in the US EPA Disinfection Guidance Manual, UV reactors certified by ÖNORM and DVGW for a *Bacillus subtilis* RED of 40mJ/cm^2 shall be granted 3-log *Cryptosporidium* and 3-log *Giardia* inactivation credit as required in this CODE.

4.7.3.3.4 Ozone Disinfection

4.7.3.3.4.1 3-log Inactivation

SECONDARY DISINFECTION SYSTEMS using ozone shall provide the required inactivation of *Cryptosporidium* in the full flow of the SECONDARY DISINFECTION SYSTEM after any side-stream has remixed into the full flow of the SECONDARY DISINFECTION SYSTEM.

4.7.3.3.4.2 Third Party Validation

Ozone systems shall be validated by an ANSI-accredited third party testing and certification organization to confirm that they provide a minimum 3 log *(99.9%)* inactivation of *Cryptosporidium* in the full SECONDARY DISINFECTION SYSTEM flow after any side-stream has remixed into the full SECONDARY DISINFECTION SYSTEM flow and prior to return of the water to the AQUATIC VENUE or AQUATIC FEATURE recirculation treatment loop.

4.7.3.3.4.3 Suitable for Use

Ozone systems and all materials used therein shall be suitable for their intended use and shall be installed:

1) In accordance with all applicable requirements,
2) As listed and labeled to a specific standard by an ANSI-accredited certification organization, and
3) As specified by the manufacturer.

4.7.3.3.4.4 Ozone System Components

An ozone system shall be a complete system consisting of the following *(either skid-mounted or components)*:

1) Ozone generator,
2) Injector / injector manifold,
3) Reaction tank *(contact tank)* / mixing tank / degas tower,
4) Degas valve *(if applicable, to vent un-dissolved gaseous ozone)*,
5) Ozone destruct *(to destroy un-dissolved gaseous ozone)*,
6) ORP monitor / controller,
7) Ambient ozone monitor / controller,
8) Air flow meter / controller, and
9) Water BACKFLOW prevention device in gas delivery system.

4.7.3.3.4.5 Appropriate Installation

These components *(or skid)* shall be installed as specified by the manufacturer to maintain the required system validation as noted above.

4.7.3.3.4.6 ORP Monitor

The ozone generating equipment shall be designed, sized, and controlled utilizing an ORP *(oxidation REDUCTION POTENTIAL)* monitor / controller *(independent of and in addition to any halogen ORP monitor/controller)*.

4.7.3.3.4.6.1 Placed Downstream

The device shall be placed in the AQUATIC VENUE and AQUATIC FEATURE recirculation water downstream of the ozone side-stream loop and before the halogen feed location.

4.7.3.3.4.6.2 Minimum ORP Reading

The minimum ORP reading shall be no less than 600 mV measured directly after *(one to five feet (30.5 cm to 1.5 m))* the ozone side-stream remixes into the full flow of the RECIRCULATION SYSTEM.

4.7.3.3.4.6.3 Maximum ORP Reading

The maximum ORP reading shall be no greater than 900 mV.

4.7.3.3.4.7 Installation and Injection Point

The ozone system injection point shall be located in the AQUATIC VENUE return line after the filtration and heating equipment, prior to the primary disinfectant injection point.

4.7.3.3.4.7.1 Injection and Mixing

The injection and mixing system shall not prevent the attainment of the recirculation rate required elsewhere in this CODE.

4.7.3.3.4.7.2 Gas Monitor / Controller

An ambient ozone gas monitor/controller located adjacent to the ozone reactor/contact tank shall be utilized to disable the ozone system in the event of an ozone gas leak.

4.7.3.3.4.8 Comply with Fire Code

Ozone system installations shall comply with the NFPA 1 Fire Code or the International Fire Code and any other CODES, STANDARDS, or requirements as mandated by the AHJ.

4.7.3.3.4.9 Air Space Testing

At the time the ozone generating equipment is installed, again after 24 hours of operation, and annually thereafter, the air space within six inches of the AQUATIC VENUE water shall be tested to determine compliance of less than 0.1 PPM *(mg/L)* gaseous ozone.

4.7.3.3.4.9.1 Results

Results of the test shall be maintained on site for review by the AHJ.

4.7.3.3.4.10 Automatic Shut Down

Automatic shutdown shall occur under any condition that would result in the ozone system not operating within the established parameters needed to achieve 3-log inactivation of *Cryptosporidium (i.e. low feed gas supply, loss of vacuum or pressure, high dew point in feed air, water in ozone gas delivery line)*.

4.7.3.3.4.10.1 Electrically Interlocked

The equipment shall be electrically interlocked with AQUATIC VENUE pump*(s)* or automated feature supply valves, such that when the ozone equipment fails to produce the required dosage as measured by ORP, the AQUATIC VENUES do not operate.

4.7.3.3.4.11 ORP Reading Alarm or Visual Indication

If the ORP reading for the ozone system drops below 600 mV *(regardless of the cause)* a visual alarm or other indication shall be initiated that will alert staff on-site or remotely.

4.7.3.3.4.11.1 Signage

Signage to notify facility management shall be present adjacent to the visual alarm.

4.7.3.3.4.12 Regular Audits

In order to ensure that the supplied ozone system meets all the requirements of the STANDARD, the manufacturer shall maintain a quality system audited on a regular basis to a recognized quality STANDARD.

4.7.3.3.4.12.1 Listed

Ozone equipment shall be listed to NSF/ANSI Standard 50.l.

4.7.3.3.4.13 Reports and Documentation

The ozone system shall be supplied with the appropriate validation reports and documentation for that equipment model.

4.7.3.3.4.13.1 3-log Inactivation Chart

Ozone validation reports shall include a graph, chart, or other documentation which clearly indicates the required operating parameters for which a 3-log inactivation is guaranteed for the system in question.

4.7.3.3.4.13.2 Inclusive

This dose shall be inclusive of validation factors.

4.7.3.3.4.13.3 System Performance Curves

System performance curves that do not include such factors are not considered validated systems.

4.7.3.4 Supplemental Treatment Systems

4.7.3.4.1 General Requirements

4.7.3.4.1.1 Optional

AQUATIC VENUES that do not require SECONDARY DISINFECTION SYSTEMS may install SUPPLEMENTAL TREATMENT SYSTEMS for the purpose of enhancing overall system performance and improving water quality.

4.7.3.4.1.2 Not Required

SUPPLEMENTAL TREATMENT SYSTEMS shall not be required on any AQUATIC VENUES.

4.7.3.4.1.3 Clearly Noted

It shall be clearly noted in the AQUATIC FACILITY operating instructions that these SUPPLEMENTAL TREATMENT SYSTEMS do not meet the requirements of a SECONDARY DISINFECTION SYSTEM, and as such, are only considered SUPPLEMENTAL TREATMENT SYSTEMS.

4.7.3.4.1.4 No 3-log Inactivation Required

SUPPLEMENTAL TREATMENT SYSTEMS shall meet all of the requirements of this CODE, except:

1) They do not need to achieve a 3-log *(99.9%)* inactivation of *Cryptosporidium parvum* as required in MAHC Section 4.7.3.3,
2) They do not need to be able to reduce the total number of infective OOCYSTS to one OOCYST per 100 mL as required in MAHC Section 4.7.3.3, and
3) Except as noted in MAHC Sections 4.7.3.4.2 and 4.7.3.4.3 below.

4.7.3.4.1.5 Clearly Labeled

Each system shall be clearly labeled, “Supplemental Water Treatment System—Does Not meet the requirements for Secondary DISINFECTION.”

4.7.3.4.2 Ultraviolet Light

4.7.3.4.2.1 UV as Supplemental Treatment Requirement

When UV is used as a SUPPLEMENTAL TREATMENT SYSTEM, all requirements of MAHC Section 4.7.3.3.3.2 through 4.7.3.3.3.4 shall be met.

4.7.3.4.2.3 Water Features

Water features shall not require shut off if the supplemental UV system does not produce the required dosage.

4.7.3.4.2.4 Exempt

The equipment is exempt from the validation requirements of MAHC Section 4.7.3.3.3.1.

4.7.3.4.3 Ozone

4.7.3.4.3.1 Ozone as Supplemental Treatment Requirement

When ozone is used as a SUPPLEMENTAL TREATMENT SYSTEM, all requirements of MAHC Section 4.7.3.3.4.3 thru 4.7.3.3.4.9 shall be met.

4.7.3.4.3.2 Maximum ORP Reading

The maximum ORP reading shall be no greater than 900 mV.

4.7.3.4.4 Copper / Silver Ion Systems

4.7.3.4.4.1 Only EPA-Registered Disinfectants

Only those systems that are EPA-REGISTERED for use as disinfectants in AQUATIC VENUES or SPAS in the United States shall be permitted.

4.7.3.4.4.2 Suitable

Copper/silver systems, and all materials used therein, shall be suitable for their intended use.

4.7.3.4.4.3 Installed

Copper/silver systems, and all materials used therein, shall be installed in accordance with all applicable requirements and manufacturer's instructions.

4.7.3.4.5 Ultraviolet Light / Hydrogen Peroxide Systems

Ultraviolet light / hydrogen peroxide combination systems shall be prohibited for use in AQUATIC FACILITIES.

4.7.3.5 Water Quality Testing Devices and Kits

4.7.3.5.1 Compliance

WATER QUALITY TESTING DEVICES and kits shall be listed and labeled to NSF/ANSI 50 by an ANSI-accredited certification organization.

4.7.3.5.2 Water Clarity Device

Refer to MAHC Section 5.7.6.

4.7.3.6 Microbiological Testing Equipment

4.7.3.6.1 EPA-Approved

Microbiological testing equipment and methods shall be

1) EPA-Approved, EPA-Accepted, EPA-Equivalent,
2) Conforming to the latest edition of *Standard Methods for the Examination of Water and Wastewater*, or
3) Listed and labeled to NSF/ANSI 50 by an ANSI-accredited certification organization.

4.7.4 Water Replenishment System

4.7.4.1 Discharge and Measure

A means of intentionally discharging and measuring or calculating the volume of both discharged AQUATIC VENUE water and filter backwash wastewater *(or alternate means of achieving the same result)* shall be provided.

4.7.4.1.1 Alternate System

An alternate system capable of removing an equivalent amount of dissolved organic compounds and salts shall also be acceptable in lieu of discharging AQUATIC VENUE water.

4.7.4.1.1.1 Product Water

When an alternate system is used, the return water from the alternate system shall maintain salt and total organic carbon concentrations that are less than or equal to tap water.

4.7.4.1.2 Discharge

This system shall be designed to discharge *(or treat and reuse)* AQUATIC VENUE water at a rate of up to four gallons *(15 L)* per BATHER per day per AQUATIC VENUE.

4.7.5 Spas

4.7.5.1 General

4.7.5.1.1 Requirements

SPAS shall conform to the design, operation, and maintenance requirement of AQUATIC VENUES except as required below.

4.7.5.2 Flow Rates/Turnover Times

4.7.5.2.1 Maximum Allowable

All SPAS as defined in the MAHC shall be designed to have a maximum allowable TURNOVER time of 0.5 hour or less.

4.7.5.2.2 Turnover Time Variance

The AHJ may grant a TURNOVER time variance for AQUATIC VENUES with extreme volume or operating conditions based on proper engineering justification.

4.7.5.2.3 Turnover Times

TURNOVER times shall be calculated based solely on the flow rate through the filtration system.

4.7.5.3 Filtration System Inlets

SPAS shall have a minimum of two adjustable filter system INLETS spaced at least three feet *(0.9 m)* apart and designed to distribute flow evenly.

4.7.5.4 Jet System Inlets

4.7.5.4.1 Air Flow

Air flow shall be permitted through the jet system and/or when injected post-filtration.

4.7.5.4.2 Skimmer

Submerged suction SKIMMERS shall be allowed provided that the manufacturer's recommendations for use are followed.

4.8 Decks and Equipment

4.8.1 Decks

4.8.1.1 General Standards for All Decks

4.8.1.1.1 Constructed

DECKS shall be constructed in conformance with all applicable provisions of this chapter.

4.8.1.1.2 Lifeguard Placement and Safety Considerations

DECKS shall be designed to allow for QUALIFIED LIFEGUARD placement per the zone of BATHER surveillance in MAHC Section 6.3.3.1.1 and SAFETY areas and equipment in MAHC Section 4.8.5.

4.8.1.1.2.1 Deck Clearance

DECKS shall have a minimum of four feet *(1.2 m)* of clearance from AQUATIC VENUE edge to fencing or other obstruction to allow for QUALIFIED LIFEGUARD transit, roaming, or change of positioning to maximize viewing of the zone of BATHER surveillance as well as execution of water extrication.

4.8.1.1.2.2 Access Points

Access points shall be provided to QUALIFIED LIFEGUARDS to transit to QUALIFIED LIFEGUARDS positions.

4.8.1.1.2.3 Bather Zone Surveillance

The designer and owner shall consider impact on BATHER zone surveillance when determining placement of structural, operational, and theming elements.

4.8.1.1.2.3.1 Elements for Deck Placement

These elements shall include but are not limited to chairs, fencing, ADA access equipment, and AQUATIC FEATURES.

4.8.1.1.3 Joints or Gaps

Conditions between adjacent DECK materials, components, and concrete pours shall not have open joints or gaps larger than 3/16 inches wide (4.8 *mm)*, nor a maximum difference in vertical elevation of ¼ inches *(6.4 mm)*.

4.8.1.1.3.1 Vertical Elevation

Any change in vertical elevation shall be considered an edge condition.

4.8.1.1.3.2 Fillers

Open joints or gaps larger than 3/16 inches *(4.8 mm)* wide or with vertical elevations exceeding ¼ inches *(6.4 mm)* shall be rectified using appropriate fillers.

4.8.1.1.3.3 Sealants

The use of fillers such as caulk or sealant in joints or gaps shall be permitted for expansion and contraction and shall not be in violation of MAHC Section 4.8.1.1.3.

4.8.1.1.4 Rounded Edges

All DECK edges shall be beveled, rounded, or otherwise relieved to eliminate sharp corners.

4.8.1.1.5 Minimize Cracks

Joints in decking shall be provided to minimize the potential for CRACKS due to a change in elevation, for movement of the slab and for shrinkage control.

4.8.1.1.6 Concrete Decking

Where concrete is used as a DECK material, it shall be installed in accordance with the latest edition of the American Concrete Institute *(ACI)* Standards and in accordance with local building CODES.

4.8.1.2 Standards for Perimeter Decks

4.8.1.2.1 Impervious

Finish materials for the PERIMETER DECK shall be suitable for the POOL environment, non-toxic, and substantially impervious.

4.8.1.2.2 Watertight Expansion

Continuous watertight EXPANSION JOINT material shall be provided between PERIMETER DECKS and POOL coping.

4.8.1.2.2.1 Expansion Joint

Where applicable, the EXPANSION JOINT shall be designed and constructed so as to protect the coping and its mortar bed from damage as a result of movement of adjoining DECK.

4.8.1.2.3 Watertight Expansion

All conditions between adjacent concrete PERIMETER DECK pours shall be constructed with watertight EXPANSION JOINTS.

4.8.1.2.3.1 Joint Measurements

Joints shall be at least 3/16 inches *(5 mm)* in continuous width.

4.8.1.2.3.2 Vertical Differential

The maximum allowable vertical differential across a joint shall be ¼ inches *(6.5 mm)*.

4.8.1.3 Drains

Also, refer to MAHC Section 4.11.4.

4.8.1.3.1 Slope

DECKS shall be sloped away from the AQUATIC VENUE and in accordance with MAHC Table 4.8.1.3 below.

Table 4.8.1.3 Minimum Slopes for Drainage

Minimum Slopes for Drainage	
**SURFACE**	**MINIMUM SLOPE**
**Smooth finishes**; such as tile, hand-finished concrete & lightly-broomed concrete	$^1/_8$ inch per foot *(3.2 mm/30.5 cm)*
**Moderately textured finishes**; such as exposed aggregate or medium-broomed concrete	$^1/_4$ inch per foot *(6.4 mm/30.5 cm)*
**Heavily textured finishes**; such as brick *(where permitted)*	$^3/_8$ inch per foot *(9.5 mm/30.5 cm)*

4.8.1.3.1.1 Accessible Routes

Where DECK areas or portions thereof serve as ACCESSIBLE ROUTES, slopes in any direction shall not exceed ADA requirements.

4.8.1.3.1.2 All Water

All water that touches areas defined as DECK, including water originating in the AQUATIC VENUE, shall drain effectively to either perimeter areas or to DECK drains.

4.8.1.3.1.3 Remove Wastewater

Drainage shall remove AQUATIC VENUE water that splashes outside of the AQUATIC VENUE and beyond a POOL gutter system, DECK cleaning water, and rain water without leaving standing water.

4.8.1.3.2 Placement

The placement of DECK drains, where provided, shall effectively carry water away from the AQUATIC VENUE and off the DECK without ponding.

4.8.1.3.3 Cross Connection Control

There shall be no direct connection between the DECK drains and the sanitary or storm sewer system, or the AQUATIC VENUE gutter or RECIRCULATION SYSTEM.

4.8.1.3.3.1 Discharge to Sewer or Other Ground Water

If the AHJ requires an outdoor POOL to have DECK drains that discharge to a storm sewer system, ground surface, or holding pond, the pool shall be plumbed through an air-gap, BACKFLOW preventer, or other approved device as allowed by the AHJ.

4.8.1.3.3.2 No Drain

DECK drains shall not drain to the POOL, POOL gutter, or RECIRCULATION SYSTEMS.

4.8.1.3.4 Drain Bodies

Drain receptacles shall consist of non-CORROSIVE or corrosion-resistant materials.

4.8.1.3.5 Drain Covers

Drain covers shall be suitable for bare foot traffic with openings no greater than ½ inch *(1.3 cm)* and easily removable with a simple tool to facilitate regular cleaning.

4.8.1.4 Materials / Slip Resistance

4.8.1.4.1 General

PERIMETER DECK and POOL DECK shall be constructed with a uniform and easily cleaned surface such as concrete, tile, manufactured or acrylic surfaces.

4.8.1.4.2 Slip Resistance

All DECKS shall have slip-resistant, textured finishes, which are not conducive to slipping under contact of bare feet in wet or dry conditions.

4.8.1.4.3 Carpet

Carpet and artificial turf shall be prohibited materials for PERIMETER DECK and POOL DECK.

4.8.1.4.4 Wood

Wood shall be a prohibited material for use as PERIMETER DECK.

4.8.1.4.5 Dry Deck

DRY DECK shall be easily maintained and not create a public health hazard.

4.8.1.4.5.1 Not Required

DRY DECK shall not be required to be hard-paved or impervious.

4.8.1.4.5.2 Wood Decking

Wood decking may be permitted for DRY DECK.

4.8.1.4.6 Landscaping

Loose plant material or bedding shall not be permitted within PERIMETER DECKS.

4.8.1.4.6.1 Stable Materials

Stable materials are permitted.

4.8.1.5 Deck Size / Width

4.8.1.5.1 Perimeter Deck

4.8.1.5.1.1 Width

PERIMETER DECKS shall be four feet *(1.2 m)* minimum in unobstructed width around the POOL perimeter as prescribed in this section.

4.8.1.5.1.1.1 Circulation Path

PERIMETER DECK may serve as part of the CIRCULATION PATH.

4.8.1.5.1.1.2 Flush with Pool Wall

PERIMETER DECK areas shall be flush with POOL walls/copings except where special conditions exist, such as elevated beam or parapet, raised transfer walls, or as permitted by other sections of this CODE.

4.8.1.5.1.2 Perimeter Decking

PERIMETER DECKS shall be provided around 100% of the AQUATIC VENUE perimeter except where special conditions exist as permitted by other sections of this CODE.

4.8.1.5.1.3 Unguarded Aquatic Venues

For unguarded POOLS, PERIMETER DECKS shall be provided in compliance with at least one of two options:

1) Provide PERIMETER DECK around 100% of the of the POOL perimeter; or
2) Provide PERIMETER DECK such that the entire perimeter and depth of the POOL is readily reachable by a pole and hook from the PERIMETER DECK.

4.8.1.5.1.4 Spectator Seating

Refer to MAHC Section 4.6.10.

4.8.1.5.2 Fixed Equipment

4.8.1.5.2.1 Unobstructed Deck

Unobstructed DECK area four feet *(1.2 m)* minimum in width shall be provided for access around:

1) Diving equipment,
2) Special feature stairways *(such as a waterslide)*,
3) Lifeguard stands,
4) Diving boards,
5) Similar DECK equipment,
6) ADA access equipment, and
7) Structural columns.

4.8.1.5.2.2 Circulation Path

This unobstructed area may overlap the CIRCULATION PATH.

4.8.1.5.2.3 Queuing Space

Where reasonably anticipated, queuing space shall be provided at applicable equipment to minimize encroachment into the CIRCULATION PATH.

4.8.1.5.2.4 Free Space

Free area around equipment may consist of PERIMETER DECK and/or POOL DECK, as applicable.

4.8.1.5.3 Circulation Path

4.8.1.5.3.1 Conformance

A continuous and unobstructed CIRCULATION PATH shall be provided in conformance with ADA requirements for an ACCESSIBLE ROUTE.

4.8.1.5.3.2 Equipment and Furniture

DECK furniture locations shall be designed not intrude upon any CIRCULATION PATH.

4.8.1.5.3.3 Connect

CIRCULATION PATHS shall connect all site amenities, entrances and exits as required by ADA.

4.8.1.5.3.4 Deck Types

CIRCULATION PATHS may consist of any combination of permitted DECK types.

4.8.1.6 Wing Walls or Peninsulas

4.8.1.6.1 No Perimeter Deck

WING WALLS or PENINSULAS less than 18 inches *(45.7 cm)* in width shall not be considered a part of the PERIMETER DECK.

4.8.1.6.1.1 Use by Lifeguards

A WING WALL or PENINSULA greater than 18 inches *(45.7 cm)* wide but less than 48 inches *(1.2 m)* wide may be used by QUALIFIED LIFEGUARD personnel but shall not be considered as part of the PERIMETER DECK.

4.8.1.6.1.2 Slip Resistant

Any WING WALL or PENINSULA intended to be accessed by QUALIFIED LIFEGUARDS shall be constructed of slip-resistant materials.

4.8.1.6.2 Perimeter Overflow System

If it is impractical to design a PERIMETER OVERFLOW SYSTEM into the WING WALL or PENINSULA due to width or height, then the overflow system may bypass the WING WALL or PENINSULA.

4.8.1.6.3 Pool Perimeter

WING WALLS and PENINSULAS shall be considered part of the POOL.

4.8.1.6.3.1 Calculating

WING WALLS and PENINSULAS shall not be accounted for in calculating the POOL perimeter.

4.8.1.6.4 Normal Operating Water Level

WING WALLS and PENINSULAS shall be at or above the normal operating water level of the POOL.

4.8.1.6.5 Deck Drainage

DECK drainage shall not be required for WING WALLS or PENINSULAS as they are considered part of the POOL.

4.8.1.6.5.1 Crowned

The tops shall be crowned to prevent standing water and sloped to the POOL or overflow system.

4.8.1.6.6 Vertical Depth Markers

Vertical depth markers shall be provided around WING WALLS and PENINSULAS in accordance with MAHC Section 4.5.19.

4.8.1.7 Islands

4.8.1.7.1 Minimum Width

An ISLAND not more than 18 inches *(45.7 cm)* in width shall be designed to discourage a person from walking on the ISLAND by not providing stairs, ladders, or bridges to the ISLAND.

4.8.1.7.2 Slip Resistant

The surface of ISLANDS intended for foot traffic shall be slip resistant.

4.8.1.7.3 Lifeguards

An ISLAND 18 inches *(45.7 cm)* to 48 inches *(1.2 m)* wide may be allowed for use only by QUALIFIED LIFEGUARDS.

4.8.1.7.4 Vertical Depth Markers

Vertical depth markers shall be provided around ISLANDS in accordance with MAHC Section 4.5.19 and visible from all sides.

4.8.1.7.5 Horizontal Depth Markers

Horizontal depth markings and warning signs shall also be required per MAHC Section 4.5.19 if the ISLAND is designed for BATHER use.

4.8.1.7.5.1 Islands Not Intended for Bather Use

If the island is not designed for bather use, warning signs stating “No Entry” shall be required.

4.8.1.7.6 Bridge or Stairway

An ISLAND designed for BATHER traffic shall be accessible by bridge, ramp, ladder, or stairway from the POOL.

4.8.1.7.7 Minimum Clearance

All bridges spanning a POOL or any other structures not intended for interactive play shall have a minimum clearance of seven feet *(2.1 m)* from the bottom of the POOL to any structure overhead.

4.8.1.7.8 Guard Rails

Any bridge shall have a minimum 42 inch *(1.1 m)* high BARRIER on both sides.

4.8.1.8 Heated Decks

4.8.1.8.1 Freeze Protection

Where heated DECKS are provided for the purpose of freeze protection, the extent of heated area shall minimally include the entire required PERIMETER DECK and required CIRCULATION PATH*(S)*.

4.8.1.8.2 Clearly Delineated

Heated DECK paths must be clearly delineated with respect to un-heated DECKS.

4.8.1.9 Hose Bibbs

4.8.1.9.1 General

Domestic water hose bibbs shall be provided in sufficient quantity, spacing, and type to wash down PERIMETER DECK and POOL DECK areas using a hose of no longer than 100 ft *(30.5 m)*.

4.8.1.9.2 Backflow Prevention

All hose bibbs shall be equipped with BACKFLOW prevention devices.

4.8.2 Diving Boards and Platforms

4.8.2.1 Diving Envelope

4.8.2.1.1 Competitive Diving

Diving boards shall be permitted only when the diving envelope conforms to the STANDARDS of the certifying agency that regulates competitive diving at the AQUATIC FACILITY. Such certifying agencies include:

1) National Collegiate Athletic Association *(NCAA)*,
2) the National Federation of State High School Associations *(NFSHSA)*,
3) the Federation Internationale de Natation Amateur *(FINA)*, or
4) U.S.A. Diving, Inc.

4.8.2.1.2 Non-Competitive Diving

If the AQUATIC VENUE does not have competitive diving, then the diving envelope shall conform to the diving envelope STANDARDS of

1) MAHC Table 4.8.2.2,
2) MAHC Figure 4.8.2.2.1, and
3) MAHC Figure 4.8.2.2.2.

4.8.2.2 Steps and Guardrails

4.8.2.2.1 Higher than Twenty-One Inches

Diving stands higher than 21 inches *(53.3 cm)* measured from the DECK to the top of the butt end of the board or platform shall have steps or a ladder and handrails.

4.8.2.2.2 Self-Draining Treads

Steps or ladder treads shall be self-draining, corrosion resistant, non-slip, and designed to support the maximum expected load.

4.8.2.2.3 Short Platforms

Diving stands or platforms that are one meter *(3.4 ft)* or higher must be protected with guard rails at least 30 inches *(76.2 cm)* above the board, extending at least to the edge of the water along with intermediate rails.

4.8.2.2.4 Tall Platforms

Diving stands or platforms that are two meters *(6.6 ft)* or higher must have guard rails with the top rail at least 36 inches *(0.9 m)* above the board and a second rail approximately half the distance from the platform to the upper rail.

Table 4.8.2.2: Diving Platform Areas

PUBLIC SWIMMING POOLS<br>**Table 1**<br>Diving Areas						
Letters below refer to Figure 1		Board height-meters	0.5 Meter	0.75 Meter	1.0 Meter	3.0 Meters
		Board height (feet)	1'8"	2'6"	3'4"	9'11"
		Board length (feet)	10'0"	12'0"	16'0"	16'0"
		Board width (feet)	1'8"	1'8"	1'8"	1'8"
			Minimum dimensions in feet			
A	Distance from plummet back to pool wall		3'0"	4'6"	6'0"	6'0"
B	Distance from plummet to pool wall at side		10'0"	10'0"	10'0"	11'6"
C	Distance from plummet to adjacent plummet		8'10"	8'10"	8'10"	8'6.5"
D	Distance from plummet to pool wall ahead		26'0"	27'10"	29'7"	33'8"
E	Height,board to ceiling at plummet & distances F and G		16'0"	16'0"	16'0"	16'0"
F	Clear overhead distance behind and each side of plummet		8'0"	8'0"	8'0"	8'0"
G	Clear overhead distance ahead of plummet		16'0"	16'0"	16'0"	16'0"
H	Depth of water at plummet		9'6"	10'9"	12'0"	12'6"
J	Distance ahead of plummet to depth K		12'0"	14'3"	16'6"	19'9"
K	Depth at distance J ahead of plummet		8'9"	10'0"	11'3.375"	12'2"
L	Distance at each side of plummet to depth M		8'0"	8'1.5"	8'3"	9'11"
M	Depth at distance L on each side of plummet		9'1	10'4"	11'7.5"	12'2"
N	Maximum slope to reduce height E		30°	30°	30°	30°
P	Maximum floor slope to reduce depth ahead of K, to the sides of M, or back to pool wall behind H		3:1	3:1	3:1	3:1

Figure 4.8.2.2.1: Diving Platform Longitudinal Section

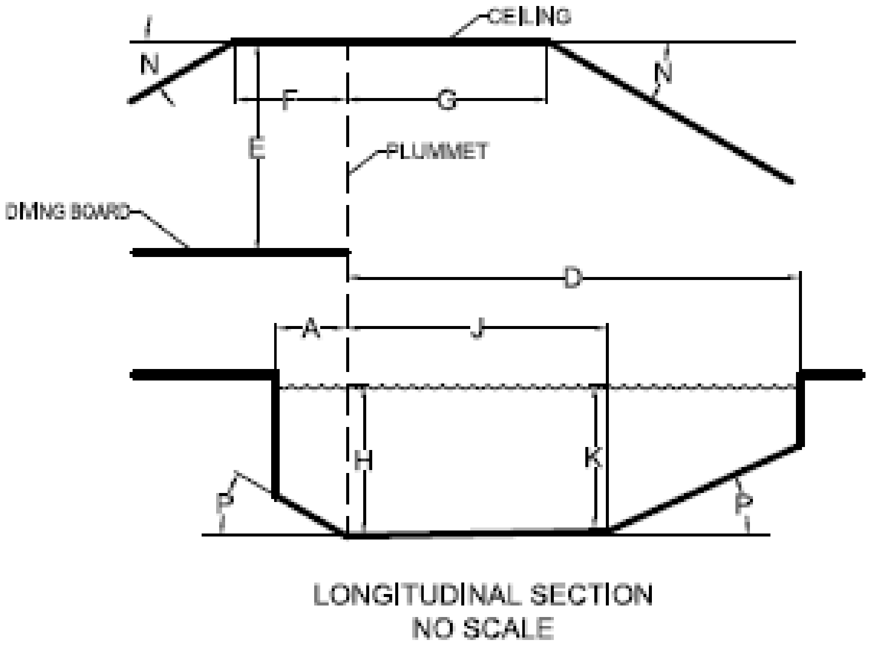

Figure 4.8.2.2.2: Diving Platform Cross Section

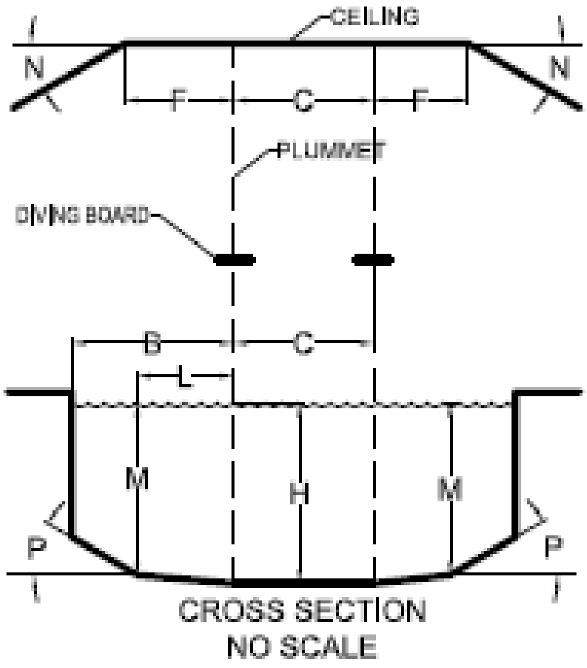

4.8.3 Starting Platforms

4.8.3.1 Conform to Standard Codes

Starting platforms shall be installed and conform to applicable SAFETY STANDARDS established by:

1) Federation Internationale de Natation *(FINA)*,
2) U.S.A. Swimming,
3) National Collegiate Athletic Association *(NCAA)*,
4) National Federation of State High Schools Associations *(NFSHSA)*,
5) YMCA, or
6) Other sanctioning body.

4.8.3.2 Minimum Water Depth

Starting platforms shall be installed in a minimum water depth of four feet *(1.2 m)*.

4.8.3.3 Leading Edge

The leading edge of starting platforms shall have a maximum height of 30 inches *(76.2 cm)* above the water surface.

4.8.3.4 Slip Resistant

Starting platforms shall have slip resistant tread surfaces.

4.8.3.5 Secure and Stable

Starting platforms shall be installed and secured per manufacturer's recommendations at all times when in use.

4.8.4 Pool Slides [N/A]

4.8.5 Lifeguard- and Safety-Related Equipment

4.8.5.1 Equipment Inspection and Maintenance [N/A]

4.8.5.2 Safety Equipment Required at all Aquatic Facilities

4.8.5.2.1 Emergency Communication Equipment

The AQUATIC FACILITY or each AQUATIC VENUE, as necessary, shall have a functional telephone or other communication device that is hard wired and capable of directly dialing 911 or function as the emergency notification system.

4.8.5.2.1.1 Conspicuous and Accessible

The telephone or communication system or device shall be conspicuously provided and accessible to AQUATIC VENUE users such that it can be reached immediately.

4.8.5.2.1.2 Alternate Communication Systems

Alternate systems or devices are allowed with approval of the AHJ in situations when a telephone is not logistically sound, and an alternate means of communication is available, which meet the requirements of MAHC Section 5.8.5.2.1.2.

4.8.5.2.1.3 Internal Communication

The AQUATIC FACILITY design shall include a method for staff to communicate in cases of emergency.

4.8.5.2.1.4 Signage

A sign shall be posted at the telephone providing dialing instructions, address and location of the AQUATIC VENUE location, and the telephone number.

4.8.5.3 Safety Equipment Required at Facilities with Lifeguards

4.8.5.3.1 Lifeguard Chair and Stand Placement

The designer shall coordinate with the owner and/or an aquatic consultant to consider the impact on BATHER surveillance zones for placement of chairs and stands designed to be permanently installed so as to provide an unobstructed view of the BATHER surveillance zones.

4.8.5.3.2 Lifeguard Chair and Stand Design

The chairs/stands must be designed:

1) With no sharp edges or protrusions;
2) With sturdy, durable, and UV resistant materials;
3) To provide enough height to elevate the lifeguard to an eye level above the heads of the BATHERS; and
4) To provide safe access and egress for the lifeguard.

4.8.5.3.3 UV Protection for Chairs and Stands

Where provided, permanently installed chairs/stands, where QUALIFIED LIFEGUARDS can be exposed to ultraviolet radiation, shall include protection from such ultraviolet radiation exposure.

4.8.6 Barriers and Enclosures

4.8.6.1 General Requirements

4.8.6.1.1 Enclosed

All AQUATIC FACILITIES, CHEMICAL STORAGE SPACES, and AQUATIC VENUE mechanical spaces shall be enclosed to prevent unauthorized entry.

4.8.6.1.1.1 Barriers

The ENCLOSURE may consist of any combination of building envelopes, site walls, or fencing as provided for in this section.

4.8.6.1.1.2 Patron Accessibility

BARRIERS shall be provided between CHEMICAL STORAGE SPACES, POOL, mechanical spaces, and areas accessible to the public, in accordance with local building CODES.

4.8.6.2 Construction Requirements

4.8.6.2.1 Discourage Climbing

BARRIERS or ENCLOSURES shall discourage climbing by preventing access to nearby structures to simplify climbing over it, such as: light poles, site furnishings, overhanging tree limbs or other obvious footholds or handholds.

4.8.6.2.1.1 Horizontal Mid-Rails

Horizontal mid-rails shall not be permitted.

4.8.6.2.1.2 Mesh Fencing

Chain-link fencing constructed of a maximum opening of 1¾ inches *(44.4 mm)* mesh shall be permitted.

4.8.6.2.2 Emergency Exit Paths

Enclosures for AQUATIC VENUES shall not block or encumber a required emergency egress path from other structures.

4.8.6.2.2.1 Pathways

Where a required emergency egress path enters an area occupied by an outdoor AQUATIC VENUE, emergency exit pathways from the building*(s)* shall continue on DECK of least equally unencumbered width, and continue to the ENCLOSURE and through gates.

4.8.6.2.2.2 Exit Pathways

Exit pathways shall be separated with a BARRIER from AQUATIC VENUES not in operation.

4.8.6.2.2.3 Seasonal Separation

Seasonal separation may be employed at seasonally operated AQUATIC VENUES, subject to the same physical requirements of permanent barriers for AQUATIC VENUES.

4.8.6.2.3 Windows

Windows on a building that forms part of an ENCLOSURE around an AQUATIC VENUE shall have a maximum opening width not to exceed four inches *(10.2 cm)*.

4.8.6.2.3.1 Opened

If designed to be opened, windows shall also be provided with a non-removable screen.

4.8.6.2.4 Height

For the purposes of this section, height shall be measured from finished grade to the top of the BARRIER on the side outside of the BARRIER surrounding an AQUATIC VENUE.

4.8.6.2.4.1 Change in Grade

Where a change in grade occurs at a BARRIER, height shall be measured from the uppermost grade to the top of the BARRIER.

4.8.6.2.4.2 Fencing Requirements

AQUATIC FACILITY ENCLOSURES shall not be less than six feet *(1.8 m)* in height.

4.8.6.2.4.3 Other Barriers Not Serving as Part of an Enclosure

Except where otherwise noted, all other BARRIERS not serving as part of an AQUATIC FACILITY ENCLOSURE shall not be less than 42 inches *(1.1 m)* in height.

4.8.6.3 Gates and Doors

4.8.6.3.1 Self-Closing and Latching

All primary public access gates or doors serving as part of an AQUATIC FACILITY ENCLOSURE or required AQUATIC VENUE ENCLOSURE shall be self-closing and self-latching from any open position.

4.8.6.3.1.1 Locked

All gates or doors shall be capable of being locked from the exterior.

4.8.6.3.1.2 Emergency Egress

Gates or doors shall be designed in such a way that they do not prevent egress in the event of an emergency.

4.8.6.3.2 Gates

Gates shall be at least equal in height at top and bottom to the BARRIER of which they are a component.

4.8.6.3.3 Turnstiles

Turnstiles shall not form a part of an AQUATIC FACILITY ENCLOSURE.

4.8.6.3.4 Exit Gates

EXIT GATES shall be conspicuously marked on the inside of the AQUATIC VENUE or AQUATIC FACILITY.

4.8.6.3.4.1 Quantity, Location, and Width

Quantity, location, and width*(s)* for EXIT GATES shall be provided consistent with local building and fire CODES and applicable accessibility guidelines.

4.8.6.3.5 Swing Outward

EXIT GATES shall swing away from the AQUATIC VENUE ENCLOSURE except where emergency egress CODES require them to swing into the AQUATIC VENUE ENCLOSURE.

4.8.6.3.6 Absence of Local Building Codes

Where local building CODES do not otherwise govern, at least one EXIT GATE shall be required for each logical AQUATIC VENUE area including individual POOLS or grade levels or both.

4.8.6.3.7 Unguarded Pools

For unguarded AQUATIC VENUES, self-latching mechanisms must be located not less than 3 ½ feet *(1.1 m)* above finished grade.

4.8.6.3.7.1 Operable by Children

For unguarded AQUATIC VENUES, self-latching mechanisms shall not be operable by small children on the outside of the ENCLOSURE around the AQUATIC VENUE.

4.8.6.3.8 Other Aquatic Venues

For all other AQUATIC VENUES, EXIT GATES or doors shall be constructed so as to prevent unauthorized entry from outside of the ENCLOSURE around the AQUATIC VENUE.

4.8.6.4 Indoor Aquatic Venues

4.8.6.4.1 Enclosure

Building walls enclosing an indoor AQUATIC FACILITY may be designated as the AQUATIC FACILITY ENCLOSURE.

4.8.6.4.2 Securable

Indoor AQUATIC VENUES shall be securable from unauthorized entry from other building areas or the exterior.

4.8.6.4.3 Indoor and Outdoor Aquatic Venues

Where separate indoor and outdoor AQUATIC VENUES are located on the same site, an AQUATIC VENUE ENCLOSURE shall be provided between them.

4.8.6.4.3.1 Year-Round Operation

Exception: Where all AQUATIC VENUES are operated continuously 12 months a year on the same schedule.

4.8.6.4.4 Wall Separating

For a passage through a wall separating the indoor portion of an AQUATIC VENUE from an outdoor portion of the same AQUATIC VENUE, the overhead clearance of the passage to the AQUATIC VENUE floor shall be at least six feet eight inches *(2.0 m)* to any solid structure overhead.

4.8.6.5 Multiple Aquatic Venues

4.8.6.5.1 One Enclosure

Except as otherwise required in this CODE, one ENCLOSURE may surround multiple AQUATIC VENUES at one facility.

4.8.6.5.2 Wading Pools

WADING POOLS shall not require separation from other WADING POOLS by a BARRIER. Refer to MAHC Section 4.12.9 for additional guidance about WADING POOLS.

4.8.7 Aquatic Venue Cleaning Systems

4.8.7.1 No Hazard

The cleaning system provided shall not create an entanglement or suction entrapment hazard or interfere with the operation or use of the AQUATIC VENUE.

4.8.7.2 Common Cleaning Equipment

If there are multiple AQUATIC VENUES at one AQUATIC FACILITY, the AQUATIC FACILITY may use common cleaning equipment.

4.8.7.3 Integral Vacuum Systems

Use of integral vacuum systems, meaning a vacuum system that uses the main circulating pump or a dedicated vacuum pump connect to the pool with PVC piping and terminating at the pool with a flush-mounted vacuum port fitting, shall be prohibited.

4.8.7.4 GFCI Power

Where used, PORTABLE VACUUM cleaning equipment shall be powered by circuits having GROUND-FAULT CIRCUIT INTERRUPTERS.

4.8.7.5 Low Voltage

Any ROBOTIC CLEANERS shall utilize low voltage for all components that are immersed in the POOL water.

4.8.7.6 GFCI Connection

Any ROBOTIC CLEANER power supply shall be connected to a circuit equipped with a ground fault interrupter, and should not be operated using an extension cord.

4.9 Filter/Equipment Room

4.9.1 Equipment Room

4.9.1.1 General Requirements

4.9.1.1.1 Nonabsorbent Material

The equipment area or room floor shall be of concrete or other suitable material having a smooth slip resistant finish and shall have positive drainage, including a sump drain pump if necessary.

4.9.1.1.2 Floor Slope

Floors shall have a slope toward the floor drain and/or sump drain pump adequate to prevent standing water at all times.

4.9.1.1.3 Opening

The opening to the EQUIPMENT ROOM or area shall be designed to provide access for all anticipated equipment.

4.9.1.1.4 Hose Bibb

At least one hose bibb with BACKFLOW preventer shall be located in the EQUIPMENT ROOM or shall be accessible within an adequate distance of the EQUIPMENT ROOM so that a hose can service the entire EQUIPMENT ROOM.

4.9.1.2 Construction

4.9.1.2.1 Size

The size of the equipment room or area shall provide working space to perform routine operations and equipment service.

4.9.1.2.1.1 Adequate Storage Space

EQUIPMENT ROOMS also intended for STORAGE shall have adequate space provided for such STORAGE, without reducing the working spaces.

4.9.1.2.2 Lighting

Equipment rooms or areas shall be lighted to provide 30 foot candles *(323 lux)* of illumination at floor level in accordance with IESNA guidelines.

4.9.1.3 Electrical

4.9.1.3.1 Conform to NEC

All electrical wiring shall conform to the edition of NEC adopted by the AHJ.

4.9.1.3.2 Conform to NRTL

Equipment, components, and their application and installation shall conform to the NRTL listing.

4.9.1.4 Ventilation

4.9.1.4.1 Code Conformance

EQUIPMENT ROOM ventilation shall address:

1) COMBUSTION requirements,
2) Heat dissipation from equipment,
3) Humidity from surge or balance tanks,
4) Ventilation to the outside, and
5) Air quality.

4.9.1.5 Markings

4.9.1.5.1 Piping Identified

All piping in the EQUIPMENT ROOM shall be permanently identified by its use and the AQUATIC VENUE and AQUATIC FEATURE it serves.

4.9.1.5.1.1 Components to Identify

Identification shall be provided for:

1) Main drains and SKIMMERS,
2) Filtered water,
3) Make-up water,
4) Backwash water,
5) CHLORINE *(or disinfection)* feeds,
6) Acid *(or pH)* feeds,
7) Compressed air lines,
8) Gutters,
9) Chemical sample piping, and
10) POOL heating lines.

4.9.1.5.2 Piping Marked

All piping shall be marked with directional arrows as necessary to determine flow direction.

4.9.1.5.3 Valves Identified

All valves shall be clearly identified by number with a brass tag, plastic laminate tags, or permanently affixed alternate.

4.9.1.5.3.1 Valves Described

Valves shall be described as to their function and referenced in the operating instruction manual

4.9.1.5.3.2 Piping Diagram

A water-resistant, easily read, wall-mounted piping diagram shall be furnished and installed inside the EQUIPMENT ROOM.

4.9.1.6 Equipment Rooms Containing Combustion Equipment [N/A]

See Annex Language

4.9.1.7 Separation from Chemical Storage Spaces

4.9.1.7.1 Equipment

4.9.1.7.1.1 Contaminated Air

Combustion equipment, air-handling equipment, and electrical equipment shall not be exposed to air contaminated with CORROSIVE chemical fumes or vapors.

4.9.1.7.1.2 Equipment Restrictions

Spaces containing combustion equipment, air handling equipment, and/or electrical equipment and spaces sharing air distribution with spaces containing such equipment shall not be used as CHEMICAL STORAGE SPACES at the same time.

4.9.1.7.1.2.1 Listed and Labeled

Exception: Equipment listed and labeled for use in that atmosphere shall be acceptable, where approved by the AHJ.

4.9.1.7.1.3 Isolated

Spaces containing combustion equipment, air-handling equipment, and/or electrical equipment and spaces sharing air distribution with spaces containing such equipment shall be isolated from CHEMICAL STORAGE SPACE air.

4.9.1.7.2 Doors and Openings

4.9.1.7.2.1 Between Equipment and Chemical Storage

A door or doors shall not be installed in a wall between such EQUIPMENT ROOMS and an interior CHEMICAL STORAGE SPACE.

4.9.1.7.2.2 No Openings

There shall be no ducts, grilles, pass-throughs, or other openings connecting such EQUIPMENT ROOMS to CHEMICAL STORAGE SPACES, except as permitted by the fire code.

4.9.1.7.2.3 Indoor Aquatic Facility Air

Spaces containing combustion equipment, air-handling equipment, and/or electrical equipment and spaces sharing air distribution with spaces containing such equipment shall be isolated from INDOOR AQUATIC FACILITY air.

4.9.1.7.2.3.1 Listed Equipment

Exception: Equipment listed for the atmosphere shall be acceptable.

4.9.1.7.2.4 No Openings

There shall be no ducts, grilles, pass-throughs, or other openings connecting such spaces to an INDOOR AQUATIC FACILITY.

4.9.1.7.2.4.1 Air Handlers

Ducts which connect the INDOOR AQUATIC FACILITY to the duct connections of air handlers shall not be construed as connecting the air-handler space to the INDOOR AQUATIC FACILITY.

4.9.1.7.2.4.2 HVAC Equipment

Exception: HVAC equipment which is rated for INDOOR AQUATIC FACILITY atmosphere and which serves only that INDOOR AQUATIC FACILITY shall be acceptable.

4.9.1.7.2.5 Openings / Gaps

Where building construction leaves any openings or gaps between floors and walls, or between walls and other walls, or between walls and ceilings, such gaps shall be permanently sealed against air leakage.

4.9.1.7.3 Indoor Aquatic Facility Access

4.9.1.7.3.1 Floor Slope

Where a door or doors must be installed in a wall between an EQUIPMENT ROOM and an INDOOR AQUATIC FACILITY, the floor of the EQUIPMENT ROOM shall slope per local building requirements back into the EQUIPMENT ROOM in such a way as to prevent any equipment-room spills from running under the door into the INDOOR AQUATIC FACILITY.

4.9.1.7.3.1.1 Four Inches

Exception: This requirement may be met by a floor all of which is at least 4 inches *(10.2 cm)* below the level of the nearest part of the INDOOR AQUATIC FACILITY floor.

4.9.1.7.3.1.2 Dike

Exception: This requirement may be met by a continuous dike not less than four inches *(10.2 cm)* high located entirely within the EQUIPMENT ROOM, which will prevent spills from reaching the INDOOR AQUATIC FACILITY floor.

4.9.1.7.3.1.3 Floor Drains

Equipment-room floor drains may be required by the AHJ.

4.9.1.7.3.2 Automatic Closer

Such door or doors between an EQUIPMENT ROOM and an INDOOR AQUATIC FACILITY shall be equipped with an automatic closer.

4.9.1.7.3.2.1 Maintained to Close Reliably

The door, frame, and automatic closer shall be installed and maintained so as to ensure that the door closes completely and latches without human assistance.

4.9.1.7.3.3 Automatic Lock

Such door or doors between an EQUIPMENT ROOM and an INDOOR AQUATIC FACILITY shall be equipped with an automatic lock.

4.9.1.7.3.3.1 Restrict Access

Such lock shall require a key or combination to open from the INDOOR AQUATIC FACILITY side.

4.9.1.7.3.3.2 One Hand

Such lock shall be so designed and installed as to be opened by one hand from the inside of the room under all circumstances, without the use of a key or tool.

4.9.1.7.3.4 Warning Sign

Such doors shall be equipped with permanent signage warning against unauthorized entry.

4.9.1.7.3.5 Gasket

All sides of such doors shall be equipped with a gasket.

4.9.1.7.3.1.5 Prevent Air Passage

The gasket shall be so installed as to prevent the passage of air, fumes, or vapors when the door is closed.

4.9.1.7.3.6 Not Relief

This section shall not be construed as granting relief from MAHC Section 4.9.1.7.2.1.

4.9.1.8 Other Equipment Room Guidance

4.9.1.8.1 Access Space

Where ventilation, air filtration, or space dehumidification, heating, or cooling for an INDOOR AQUATIC FACILITY is by mechanical equipment located in an EQUIPMENT ROOM, adequate access space shall be provided to allow for inspection and service.

4.9.1.8.1.1 Size Requirements

The access spaces shall be the greater of:

1) Those required by OSHA, NEC, National Fuel Gas Code, or other official requirements; or
2) The equipment-manufacturers' recommendations.

4.9.1.8.2 Adequate Space

Where ventilation, air filtration, or space heating or cooling for an INDOOR AQUATIC FACILITY is beside mechanical equipment located in an EQUIPMENT ROOM, adequate space for required straight lengths of duct shall be provided as the greater of those described in AMCA 201, SMACNA Duct Manual, ACCA Manual SPS Sec. 13, or the equipment manufacturer's recommendations.

4.9.1.8.3 Minimize Hazards

Where an EQUIPMENT ROOM contains equipment requiring regular service or maintenance, the room shall be so designed and constructed as to minimize the hazards of such maintenance and service.

4.9.1.8.3.1 Ladder Installed

Where a ladder will be required for service or maintenance of equipment, other permanently installed equipment shall not be so located as to interfere with the use of the ladder.

4.9.1.8.3.2 Alternative Access

Where a ladder cannot be safely or practically used to service equipment where any serviceable component is more than 6.5 feet *(2.0 m)* above the floor, a mezzanine floor, platform, or other arrangement for safe access shall be provided.

4.9.1.8.3.2.1 Exception

Exception: Where otherwise specifically allowed by OSHA.

4.9.1.8.3.2.2 Stricter Requirements

Exception: Where OSHA or other applicable CODES or STANDARDS have stricter requirements, those stricter requirements shall prevail.

4.9.1.8.4 Refrigeration Equipment

Where refrigeration equipment such as an air-conditioner or dehumidifier is located indoors in a building intended for occupation, arrangements for refrigerant relief *(if any)* shall be according to the applicable mechanical CODE or other applicable CODE.

4.9.2 Chemical Storage Spaces

Nothing in this section shall be construed as providing relief from applicable requirements of fire CODES, mechanical CODES, electrical CODES, etc.

4.9.2.1 Outdoor / Indoor Storage

4.9.2.1.1 Stored Outdoors

If POOL chemicals, acids, salt, oxidizing cleaning materials, or other CORROSIVE or oxidizing chemicals are STORED outdoors, they shall be stored in a well-ventilated protective area with an installed BARRIER to prevent unauthorized access as per MAHC 4.9.2.3.

4.9.2.1.2 Minimize Vapors

Where such materials must be stored in a building intended for occupancy, the transfer of chemical fumes and vapors from the CHEMICAL STORAGE SPACE to other parts of the building shall be minimized.

4.9.2.1.3 Dedicated Space

At least one space dedicated to CHEMICAL STORAGE SPACE shall be provided to allow safe STORAGE of the chemicals present.

4.9.2.1.4 Eyewash

In all CHEMICAL STORAGE SPACES in which pool chemicals will be STORED, an emergency eyewash station shall be provided.

4.9.2.1.4.1 Outside

Eyewash stations may be provided outside of the CHEMICAL STORAGE SPACE as an alternative.

4.9.2.1.4.2 AHJ Requirements

If more stringent requirements are dictated by the AHJ, then those shall govern and be applicable.

4.9.2.2 Construction

4.9.2.2.1 Foreseeable Hazards

The construction of the CHEMICAL STORAGE SPACE shall take into account the foreseeable hazards.

4.9.2.2.2 Protected

The construction of the CHEMICAL STORAGE SPACE shall, to the extent practical, protect the STORED materials against tampering, wild fires, unintended exposure to water, etc.

4.9.2.2.3 Floor

The floor or DECK of the CHEMICAL STORAGE SPACE shall be protected against substantial chemical damage.

4.9.2.2.4 Minimize Fumes

The construction and operation of a CHEMICAL STORAGE SPACE shall minimize the transfer of chemical fumes into any INTERIOR SPACE of a building intended for occupation.

4.9.2.2.5 Surfaces

Any walls, floors, doors, ceilings, and other building surfaces of an interior CHEMICAL STORAGE SPACE shall join each other tightly.

4.9.2.2.6 No Openings

There shall be no permanent or semi-permanent opening between a CHEMICAL STORAGE SPACE and any other INTERIOR SPACE of a building intended for occupation.

4.9.2.3 Exterior Chemical Storage Spaces

4.9.2.3.1 Outdoor Equipment

Equipment listed for outdoor use may be located in an exterior CHEMICAL STORAGE SPACES as permitted.

4.9.2.3.2 Fencing

Exterior CHEMICAL STORAGE SPACES not joined to a wall of a building shall be completely enclosed by fencing that is at least six feet *(1.8 m)* high and meets the non-climbability requirements of MAHC Section 4.8.6.2.1.

4.9.2.3.3 Gate

Fencing shall be equipped with a self-closing and self-latching gate having a permanent locking device.

4.9.2.4 Chemical Storage Space Doors

4.9.2.4.1 Signage

All doors opening into CHEMICAL STORAGE SPACES shall be equipped with permanent signage:

1) Warning against unauthorized entry, and
2) Specifying the expected hazards, and
3) Specifying the location of the associated SDS forms, and
4) Product chemical hazard NFPA chart.

4.9.2.4.2 Emergency Egress

Where a single door is the only means of egress from a CHEMICAL STORAGE SPACE, the door shall be equipped with an emergency-egress device.

4.9.2.4.3 Interior Door

Where a CHEMICAL STORAGE SPACE door must open to an INTERIOR SPACE, spill containment shall be provided to prevent spilled chemicals from leaving the CHEMICAL STORAGE SPACE.

4.9.2.4.4 Equipment Space

Where a CHEMICAL STORAGE SPACE door must open to an INTERIOR SPACE, the door shall not open to a space containing combustion equipment, air-handling equipment, or electrical equipment.

4.9.2.4.4.1 Corrosive

Such door shall be acceptable where all equipment thus exposed is listed for the CORROSIVE atmosphere.

4.9.2.4.5 Interior Opening

Where a CHEMICAL STORAGE SPACE door must open to an INTERIOR SPACE, such door shall have all of the following requirements outlined from MAHC Sections 4.9.2.4.5.1 to 4.9.2.4.5.7.2.

4.9.2.4.5.1 Corrosion-Resistant

Such doors shall be constructed of corrosion-resistant materials.

4.9.2.4.5.2 Automatic Lock

Such doors shall be equipped with a corrosion-resistant, automatic lock to prevent unauthorized entry.

4.9.2.4.5.2.1 Key or Combination

Such lock shall require a key or combination to open from the outside into the chemical storage space.

4.9.2.4.5.2.2 Opened

Such lock shall be so designed and installed as to be capable of being opened by one hand from the inside of the CHEMICAL STORAGE SPACE without the use of a key or tool.

4.9.2.4.5.3 Supported

Such doors shall be supported on corrosion-resistant hinges, tracks, or other supports.

4.9.2.4.5.4 Air Leakage

Such doors shall be equipped with suitable gaskets or seals on the top and all sides to minimize air leakage between the door and the door frame.

4.9.2.4.5.5 Floor

Such doors shall be equipped with a floor or threshold seal to minimize air leakage between the door and the floor or threshold.

4.9.2.4.5.6 Automatic Closer

Such doors shall be equipped with an automatic door closer that will completely close the door and latch without human assistance.

4.9.2.4.5.6.1 Air Pressure

The door closer shall be able to close the door completely against the specified difference in air pressure.

4.9.2.4.5.7 Limit Switch

Such doors shall be equipped with a limit switch and an alarm that will sound if the door remains open for more than 30 minutes.

4.9.2.4.5.7.1 Alarm

This alarm shall have a minimum output level of 85 dbA at ten feet *(3.0 m)*.

4.9.2.4.5.7.2 Loss of Air Pressure

Where an open door will result in loss of air-pressure difference, this requirement can be met by the audible alarm required under MAHC Section 4.9.2.5.2.4.

4.9.2.5 Interior Chemical Storage Spaces

4.9.2.5.1 No Air Movement

There shall be no transfer grille, pass-through grille, louver, or other device or opening that will allow air movement from the CHEMICAL STORAGE SPACE into any other INTERIOR SPACE of a building intended for occupancy or into another CHEMICAL STORAGE SPACE.

4.9.2.5.2 Electrical Conduit System

Interior CHEMICAL STORAGE SPACES that share any building surface *(wall, floor, ceiling, door, etc.)* with any other INTERIOR SPACE shall be equipped with a ventilation system that operates continuously and insures that all air movement is from all other INTERIOR SPACES and toward the CHEMICAL STORAGE SPACE.

4.9.2.5.2.1 Additional Interior Space

Interior CHEMICAL STORAGE SPACES that share an electrical conduit system with any other INTERIOR SPACE shall be equipped with a ventilation system that operates continuously and insures that all air movement is from all other INTERIOR SPACES and toward the CHEMICAL STORAGE SPACE.

4.9.2.5.2.2 Pressure Difference

This pressure difference shall be maintained by a continuously operated exhaust system used for no other purpose than to remove air from that one CHEMICAL STORAGE SPACE.

4.9.2.5.2.3 Separate Exhaust System

Where more than one CHEMICAL STORAGE SPACE is present, a separate exhaust system shall be provided for each CHEMICAL STORAGE SPACE.

4.9.2.5.2.3.1 Airflow Rate

The exhaust airflow rate shall be the greater of the:

1) OSHA requirements for working in such enclosed spaces, or
2) Amount needed to maintain the concentration of vapors or fumes below the PEL for the expected exposure time *(defined by 29 CFR 1910.1000 (OSHA))* for each stored chemical, or
3) Amount specified by International Mechanical Code, or
4) Amount specified by the Uniform Mechanical Code, or
5) Amount needed to maintain the specified pressure difference.

4.9.2.5.2.4 Alarm

The function of this exhaust system shall be monitored continuously by an audible differential-pressure alarm system which shall sound if the specified differential air pressure is not maintained for a period of thirty minutes.

4.9.2.5.2.4.1 Minimum Output

This alarm shall have a minimum output level of 85 dbA at ten feet *(3.0 m)*.

4.9.2.5.2.4.2 Manual Reset

The specified alarm shall require manual reset to silence it.

4.9.2.6 Air Ducts in Interior Chemical Storage Spaces

4.9.2.6.1 No Air Movement

No duct shall allow air movement from the CHEMICAL STORAGE SPACE into any other INTERIOR SPACE of a building intended for occupation or into any other CHEMICAL STORAGE SPACE.

4.9.2.6.2 Chemical Storage

Air ducts shall not enter or pass through an interior CHEMICAL STORAGE SPACE.

4.9.2.6.2.1 Corrosion-Resistant

Exception: A corrosion-resistant duct used for no other purpose than to exhaust air from the CHEMICAL STORAGE SPACE shall be acceptable.

4.9.2.6.2.1.1 Building Exterior

This corrosion-resistant duct must exhaust to the exterior and must end at a point on the exterior of the building, at least 20 feet *(6.1 m)* from any air intake for breathing air, cooling air, or combustion air.

4.9.2.6.2.2 Makeup Air

Exception: A duct used for no other purpose than to supply makeup air to the CHEMICAL STORAGE SPACE shall be acceptable.

4.9.2.6.2.2.1 Building Exterior

This makeup air supply duct must end at a point on the exterior of the building, at least 20 feet *(6.1 m)* from any air intake for breathing air, cooling air, or combustion air.

4.9.2.6.2.3 Other Ducts

Exception: Any other ducts specifically allowable by applicable building and mechanical CODES where such ducts are corrosion-resistant and joint-free to the extent feasible shall be acceptable.

4.9.2.7 Pipes and Tubes in Interior Chemical Storage Spaces

4.9.2.7.1 Not Enter

Pipes and tubes shall not enter or pass through an interior CHEMICAL STORAGE SPACE.

4.9.2.7.1.1 Service

Exception: As required to service devices integral to the function of the CHEMICAL STORAGE SPACE, such as pumps, vessels, controls, freeze protection, and SAFETY devices.

4.9.2.7.1.2 Automatic Fire Suppression

Exception: As required to allow for automatic fire suppression where required.

4.9.2.7.1.3 Drainage

Exception: As required for drainage.

4.9.2.7.2 Devices

Piping, tubes, drain bodies, grates, and attachment and restraint devices shall be corrosion-resistant and rated for the chemical environment*(s)* present including floor drain bodies and grates.

4.9.2.7.3 Wall Penetrations

All wall penetrations shall be sealed air-tight and shall be commensurate with the rating of the wall assembly.

4.9.2.7.3.1 Sealing Materials

Sealing material*(s)* shall be compatible with the wall assembly and the chemical environment*(s)* present.

4.9.2.8 Combustion Equipment in Interior Chemical Storage Spaces

4.9.2.8.1 Installed

No COMBUSTION DEVICE or appliance shall be installed in a CHEMICAL STORAGE SPACE, or in any other place where it will be exposed to the air from a CHEMICAL STORAGE SPACE.

4.9.2.8.1.1 Exception

Exception: A COMBUSTION DEVICE or appliance which meets all of the following requirements shall be acceptable:

1) The device or appliance is required for one or more processes integral to the function of the room, such as space heat; and
2) The device is listed for such use; and
3) The device as installed is acceptable to the AHJ.

4.9.2.9 Electrical Equipment in Chemical Storage Spaces

Electrical equipment and wiring methods used for or in CHEMICAL STORAGE SPACES shall comply with MAHC Section 4.9.2.

4.9.2.10 Ozone Rooms

4.9.2.10.1 Only Ozone Equipment

An ozone EQUIPMENT ROOM shall not be used for STORAGE of chemicals, solvents, or any combustible materials, other than those required for the operation of the recirculation and ozone generating equipment.

4.9.2.10.2 Emergency Ventilation

Rooms which are designed to include ozone equipment shall be equipped with an emergency ventilation system capable of six air changes per hour.

4.9.2.10.2.1 Exhaust Intake

The exhaust intake shall be located approximately 6 inches *(15.2 cm)* from the floor, on the opposite side of the room from the make-up air intake.

4.9.2.10.2.2 On Command

The emergency ventilation system shall be so arranged as to run on command of an ozone-leak alarm or on command of a manual switch.

4.9.2.10.2.3 Manual Switch

The manual emergency ventilation switch shall be located outside the room and near the door to the ozone room.

4.9.2.10.3 Below Grade

Ozone rooms which are below grade shall be equipped with forced-draft ventilation capable of six air changes per hour.

4.9.2.10.3.1 Exhaust Intake

The exhaust intake shall be located approximately 6 inches *(15.2 cm)* from the floor, on the opposite side of the room from the make-up air intake.

4.9.2.10.3.2 Arranged

Such ventilation system shall be so arranged as to:

1) Run automatically concurrent with the ozone equipment and for at least a time allowing for 15 air changes after the ozone equipment is stopped,
2) Run upon activation of the ozone detection and alarm system, and
3) Run on command of a manual switch.

4.9.2.10.3.3 Manual Ventilation Switch

The manual ventilation switch shall be located outside the room and near the door to the ozone room.

4.9.2.10.4 Signage

In addition to the signs required on all chemical storage areas, a sign shall be posted on the exterior of the entry door, stating “DANGER - GASEOUS OXIDIZER – OZONE” in lettering not less than four inches *(10.2 cm)* high.

4.9.2.10.5 Alarm System

Rooms containing ozone generation equipment shall be equipped with an audible and visible ozone detection and alarm system.

4.9.2.10.5.1 Requirements

The alarm system shall consist of both an audible alarm capable of producing at least 85 decibels at ten feet distance *(3.0 m)*, and a visible alarm consisting of a flashing light mounted in plain view of the entrance to the ozone-EQUIPMENT ROOM.

4.9.2.10.5.2 Sensor

The ozone sensor shall be located at a height of 18-24 inches *(45.7-61.0 cm)* above floor level and shall be capable of measuring ozone in the range of 0-2 ppm.

4.9.2.10.5.3 Ozone Concentration

The alarm system shall alarm when the ozone concentration equals or exceeds 0.1 ppm in the room.

4.9.2.10.5.4 Activation

Activation of the alarm system shall shut off the ozone generating equipment and turn on the emergency ventilation system.

4.9.2.11 Gaseous Chlorination Space

As per MAHC Section 4.7.3.2.4.1, use of compressed CHLORINE gas shall be prohibited for new construction and after SUBSTANTIAL ALTERATION to existing AQUATIC FACILITIES.

4.9.2.11.1 Existing Facilities

MAHC Section 4.9.2.11 shall apply to existing facilities using compressed chlorine gas.

4.9.2.11.2 Adequate Size

A gaseous-chlorination space shall be large enough to house the chlorinator, CHLORINE STORAGE tanks, and associated equipment as required.

4.9.2.11.3 Secure Tanks

A gaseous-chlorination space shall be equipped with facilities for securing tanks.

4.9.2.11.4 Not Below Grade

A gaseous-chlorination space shall not be located in a basement or otherwise be below grade.

4.9.2.11.5 Compressed-Chlorine Gas

Where installed indoors, compressed-CHLORINE gas storage containers and associated chlorinating equipment shall be in a separate room constructed to have a fire rating of not less than 1-hour.

4.9.2.11.6 Entry Door

The entry door to an indoor gaseous-CHLORINE space shall open to the exterior of the building or structure.

4.9.2.11.6.1 Pool or Deck

The entry door to an indoor gaseous-CHLORINE space shall not open directly towards a POOL or DECK.

4.9.2.11.7 Inspection Window

An indoor gaseous-CHLORINE space shall be provided with a shatterproof gas-tight inspection window.

4.9.2.11.8 Ventilation

Indoor gaseous-chlorination spaces shall be provided with a spark-proof ventilation system capable of 60 air changes per hour.

4.9.2.11.8.1 Exhaust-Air Intake

The exhaust-air intake of the ventilation system shall be taken at a point within 6 inches *(15.2 cm)* of the floor, and on the opposite side of the room from the makeup-air intake.

4.9.2.11.8.2 Discharge Point

The exhaust-air discharge point shall be:

1) Outdoors, and
2) Above adjoining grade level, and
3) At least 20 feet *(6.1 m)* from any operable window, and
4) At least 20 feet *(6.1 m)* from any adjacent building.

4.9.2.11.8.3 Make-Up Intake

The make-up air intake shall be within 6 inches *(15.2 cm)* of the ceiling of the space and shall open directly to the outdoors.

4.9.2.11.8.4 PPE Available

Personal protective equipment, consisting of at least a gas mask approved by NIOSH for use with CHLORINE atmospheres, shall be stored directly outside one entrance to an indoor gaseous-chlorination space.

4.9.2.11.8.5 SCBA Systems

A minimum of 2 SCBA systems shall be on hand at all times and two QUALIFIED OPERATORS are to be involved in the changing of the tanks.

4.9.2.11.8.6 Stationed Outside

One of the QUALIFIED OPERATORS should be stationed outside of the chemical room where the QUALIFIED OPERATOR inside can be seen at all times.

4.9.2.11.8.7 Emergency Telephone

An emergency direct line telephone shall be located by the door.

4.9.2.12 Windows in Chemical Storage Spaces

4.9.2.12.1 Not Required

Windows in CHEMICAL STORAGE SPACES shall not be required by this CODE.

4.9.2.12.2 Requirements

Where a window is to be installed in an interior wall, ceiling, or door of a CHEMICAL STORAGE SPACE, such window shall have the following components:

1) Tempered or plasticized glass,
2) A corrosion-resistant frame, and
3) Incapable of being opened or operated.

4.9.2.12.3 Exterior Window

Any CHEMICAL STORAGE SPACE window in an exterior wall or ceiling shall.

1) Be mounted in a corrosion-resistant frame, and
2) Be so protected by a roof, eave, or permanent awning as to minimize the entry of rain or snow in the event of window breakage.

4.9.2.13 Sealing and Blocking Materials

4.9.2.13.1 Minimize Leakage

Materials used for sealing and blocking openings in an interior CHEMICAL STORAGE SPACE shall minimize the leakage of air, vapors, or fumes from the CHEMICAL STORAGE SPACE.

4.9.2.13.2 Compatible

Materials used for sealing and blocking openings in an interior CHEMICAL STORAGE SPACE shall be compatible for use in the environment.

4.9.2.13.3 Fire Rating

Materials used for sealing and blocking openings in an interior CHEMICAL STORAGE SPACE shall be commensurate with the fire rating of the assembly in which they are installed.

4.10 Hygiene Facilities

4.10.1 General

4.10.1.1 Required at Adoption

All design provisions shall be required for new construction or SUBSTANTIAL ALTERATION to an existing AQUATIC FACILITY, except the following MAHC sections which shall be required for all AQUATIC FACILITIES at time of adoption or within one year of adoption as stated:

1) MAHC Section 4.10.4.5: DIAPER-CHANGING STATIONS,
2) MAHC Section 4.10.4.6.5: Soap Dispensers, and
3) MAHC Section 4.10.4.6.9: Trash Can

4.10.1.2 Minimum to Provide

AQUATIC FACILITIES shall provide HYGIENE FACILITIES that include, at a minimum, toilets, urinals, showers, DIAPER-CHANGING STATIONS, and other HYGIENE FIXTURES, as specified herein.

4.10.1.3 Construction

HYGIENE FACILITIES shall be constructed in accordance with applicable state and local CODES or as modified herein.

4.10.1.4 Minimum Toilets, Urinals, and Other Fixtures

The minimum number of toilets, urinals, and other HYGIENE FIXTURES provided, excluding showers, shall be the greater of the following two options:

1) In accordance with applicable state and local CODES, or
2) Based upon maximum THEORETICAL PEAK OCCUPANCY of each AQUATIC VENUE.

4.10.1.5 Theoretical Peak Occupancy

THEORETICAL PEAK OCCUPANCY for all AQUATIC VENUES shall be calculated as defined in MAHC Section 4.1.2.3.5.3.

4.10.2 Location

4.10.2.1 Distance

Except as required in MAHC Section 4.10.2.2, a drinking fountain, toilet, HAND WASH STATION, and DIAPER-CHANGING STATION shall be located no greater than 300 feet *(91 m)* walking distance from each AQUATIC VENUE.

4.10.2.2 Children Less than Five Years of Age

An AQUATIC VENUE designed primarily for use by children less than five years of age shall have a drinking fountain, toilet, HAND WASH STATION, and DIAPER-CHANGING STATION located no greater than 200 feet *(61 m)* walking distance and in clear view from the nearest entry/exit of the AQUATIC VENUE.

4.10.3 Design and Construction

4.10.3.1 Floors

The floors of HYGIENE FACILITIES and dressing areas serving AQUATIC FACILITIES shall have a smooth, easy-to-clean, impervious-to-water, slip-resistant surface.

4.10.3.2 Floor Base

A hard, smooth, impervious-to-water, easy-to-clean base shall provide a sealed, coved juncture between the wall and floor and extend upward on the wall at least six inches *(15.2 cm)*.

4.10.3.3 Floor Drains

Floor drains shall be installed in HYGIENE FACILITIES and dressing areas where PLUMBING FIXTURES are located.

4.10.3.3.1 Opening Grill Covers

Floor drain opening grill covers shall be ½-inch *(1.3 cm)* or less in width or diameter.

4.10.3.3.2 Sloped to Drain

Floors shall be sloped to drain water or other liquids.

4.10.3.3.2.1 Accessible Routes

Where DECK areas serve as ACCESSIBLE ROUTES or portions thereof, slopes in any direction shall not exceed ADA Standards and MAHC Section 4.8.1.3.1.

4.10.3.4 Partitions and Enclosures

Partitions and enclosures adjacent to HYGIENE FACILITIES shall have a smooth, easy-to-clean, impervious surface.

4.10.3.5 Hose Bibb

At least one hose bibb or other potable water source capable of connecting a hose shall be located in each HYGIENE FACILITY to facilitate cleaning.

4.10.4 Plumbing Fixture Requirements

4.10.4.1 General

4.10.4.1.1 Protected

PLUMBING FIXTURES shall be installed and operated in a manner to adequately protect the potable water supply from back siphonage or BACKFLOW in accordance with local, state or federal regulation.

4.10.4.1.2 Easily Cleaned

PLUMBING FIXTURES shall be designed so that they may be readily and frequently cleaned, SANITIZED, and disinfected.

4.10.4.1.3 Toilet Counts

Total toilet or urinal counts shall be in accordance with applicable state and local CODES or as modified herein.

4.10.4.1.4 Hand Wash Sink

Hand wash sink counts shall be in accordance with applicable state and local CODES or as modified herein.

4.10.4.2 Cleansing Showers

4.10.4.2.1 Count

The minimum number of CLEANSING SHOWERS shall be one per sex for AQUATIC FACILITIES less than 4000 square feet *(372 m^2)* in collective AQUATIC VENUE surface area.

4.10.4.2.1.1 Additional Cleansing Showers

An additional CLEANSING SHOWER per sex shall be added for each additional 4000 square feet *(372 m^2)* of AQUATIC VENUE space or portion thereof.

4.10.4.2.2 Distributed

CLEANSING SHOWERS shall be evenly distributed between sexes, as applicable.

4.10.4.2.3 Location

CLEANSING SHOWERS shall be located in a HYGIENE FACILITY near the entrance and within clear view of the AQUATIC VENUE.

4.10.4.2.4 Enclosed

Entryways to private or group CLEANSING SHOWER areas shall be enclosed by a door or curtain.

4.10.4.2.4.1 Doors

Shower doors shall be of a smooth, hard, easy-to-clean material.

4.10.4.2.4.2 Curtains

Shower curtains shall be of a smooth, easy-to-clean material.

4.10.4.2.5 Soap Dispenser

CLEANSING SHOWERS shall be supplied with soap and a soap dispenser adjacent to the shower.

4.10.4.2.6 Exemption

AQUATIC VENUES located in lodging and residential settings shall be exempt from MAHC Section 4.10.4.2.

4.10.4.3 Rinse Showers

4.10.4.3.1 Minimum and Location

A minimum of one RINSE SHOWER shall be provided on the DECK near an entry point to the AQUATIC VENUE.

4.10.4.3.2 Temperature

Water used for RINSE SHOWERS may be at ambient temperature.

4.10.4.3.3 Floor Sloped

Floors of RINSE SHOWERS shall be sloped to drain wastewater away from the AQUATIC VENUE and meet local applicable CODES.

4.10.4.3.4 Large Aquatic Facilities

RINSE SHOWERS in AQUATIC FACILITIES greater than 7500 square feet *(697 m^2)* of water surface area shall be situated adjacent to each AQUATIC VENUE entry point or arranged to encourage BATHERS to use the RINSE SHOWER prior to entering the AQUATIC VENUE.

4.10.4.3.5 Beach Entry

A minimum of four showerheads per 50 feet *(15.2 m)* of beach entry AQUATIC VENUES shall be provided as a RINSE SHOWER.

4.10.4.3.6 Lazy River

A minimum of one RINSE SHOWER shall be provided at each entrance to a LAZY RIVER AQUATIC VENUE.

4.10.4.3.7 Waterslide

A minimum of one RINSE SHOWER shall be provided at each entrance to a waterslide queue line.

4.10.4.4 All Showers

AQUATIC FACILITIES with 7500 square feet *(697 m^2)* of water area or more may be flexible in the number of CLEANSING SHOWERS they provide based on the THEORETICAL PEAK OCCUPANCY IN MAHC Section 4.1.2.3.5:

1) 25% of the required showers shall be CLEANSING SHOWERS,
2) 25% of the required showers shall be RINSE SHOWERS, and
3) the remaining 50% may be either cleansing or RINSE SHOWERS.

4.10.4.5 Diaper-Changing Stations

4.10.4.5.1 Each Facility

All AQUATIC FACILITIES allowing use by DIAPER-AGED BATHERS shall, upon adoption of this CODE, have at least one DIAPER-CHANGING STATION in each male and female HYGIENE FACILITY or make available a unisex DIAPER-CHANGING STATION.

4.10.4.5.1.1 Hand Wash Sink

The adjacent hand wash sink shall be installed and operational within one year from the date of the AHJ's adoption of the MAHC.

4.10.4.5.1.2 Portable

If a hand wash sink is not available adjacent to the DIAPER-CHANGING STATION, a portable HAND-WASH STATION shall be available adjacent to the station at all times.

4.10.4.5.2 Conform

DIAPER-CHANGING UNITS shall conform to either of the following STANDARDS:

1) ASTM standard F2285-04: *Consumer Performance Standards for Commercial Diaper-Changing Stations*, or
2) The STANDARDS for diaper-changing surfaces in the most current version of *Caring for Our Children: National Health and Safety Performance Standards: Guidelines for Out-of-Home Child Care Programs*.

4.10.4.5.3 Unisex

If only a unisex HYGIENE FACILITY is provided, it must have a DIAPER-CHANGING STATION that conforms to MAHC Section 4.10.4.5.

4.10.4.5.4 Trash Can

A covered, hands-free, plastic-lined trash receptacle or diaper pail shall be located directly adjacent to the DIAPER-CHANGING UNIT.

4.10.4.5.5 Disinfecting Surface

An EPA-registered DISINFECTANT shall be provided for maintaining a clean and disinfected DIAPER-CHANGING UNIT surface before and after each use.

4.10.4.6 Non-Plumbing Fixture Requirements

4.10.4.6.1 Easy to Clean

All HYGIENE FIXTURES and appurtenances in the dressing area shall have a smooth, hard, easy-to-clean, impervious-to-water surface and be installed to permit thorough cleaning.

4.10.4.6.2 Glass

Glass, excluding mirrors, shall not be permitted in HYGIENE FACILITIES.

4.10.4.6.3 Mirrors

Mirrors shall be shatter resistant.

4.10.4.6.4 Lockers

If lockers are provided, they shall be installed at least 3.5 inches *(8.9 cm)* above the finished floor or on legs or a base at least 3.5 inches *(8.9 cm)* high and far enough apart to allow for cleaning and drying underneath the locker.

4.10.4.6.5 Soap Dispensers

Soap dispensers shall be securely attached adjacent to hand washing sinks and at each CLEANSING SHOWER.

4.10.4.6.5.1 Dispenser Materials

The dispensers shall be of all metal, plastic, or other shatterproof materials that can be readily and frequently cleaned.

4.10.4.6.6 Dryers / Paper Towels

Hand dryers or paper towel dispensers shall be provided and securely attached adjacent to hand washing sinks.

4.10.4.6.6.1 Materials

Hand dryers and paper towel dispensers shall be of all metal, plastic or other shatterproof materials that can be readily and frequently cleaned.

4.10.4.6.7 Toilet Paper Dispensers

Toilet paper dispensers shall be securely attached to wall or partition adjacent to each toilet.

4.10.4.6.8 Female Facilities

In female HYGIENE FACILITIES, covered receptacles adjacent to each toilet shall be provided for disposal of used feminine hygiene products.

4.10.4.6.9 Trash Can

A minimum of one hands-free trash receptacle shall be provided in areas adjacent to hand washing sinks.

4.10.5 Provision of Suits, Towels, and Shared Equipment

4.10.5.1 Adequate Space

AQUATIC FACILITIES supplying reusable suits, towels, and/or shared equipment shall provide adequate equipment and space for cleaning, sanitizing, drying, and storing of these materials.

4.10.6 Foot Baths

4.10.6.1 Prohibited

FOOT BATHS shall be prohibited.

4.10.7 Sharps

4.10.7.1 Container

If razors or other sharps are supplied by the AQUATIC FACILITY, a sharps container approved by local, state or federal regulations shall be provided within the HYGIENE FACILITY.

4.11 Water Supply/ Wastewater Disposal

4.11.1 Water Supply

4.11.1.1 Public Water System

Water serving an AQUATIC FACILITY shall be supplied from a potable water source.

4.11.1.1.1 Other Sources

Other water sources such as lakes or springs may be approved to serve an AQUATIC FACILITY by the AHJ.

4.11.1.1.2 Condensate / Reclaimed Water

Use of condensate water, collected rain water, or other reclaimed water for water serving an AQUATIC VENUE is prohibited.

4.11.1.1.2.1 Condensate Use

Condensate water may be used for irrigation or other non-potable uses.

4.11.1.1.2.2 Exceptions

Exceptions to MAHC Section 4.11.1.1.2 may be made by the AHJ with evidence that such water has met all EPA potable water quality STANDARDS.

4.11.1.2 Sufficient Capacity

The water supply shall have sufficient capacity to simultaneously serve all PLUMBING FIXTURES.

4.11.1.2.1 Refill Pool

The water supply shall have sufficient capacity and pressure to refill the AQUATIC VENUE to the operating water level after backwashing filters and after any splashing or evaporative losses within one hour if the AQUATIC VENUE is operational at the time of the backwash.

4.11.2 Fill Spout

4.11.2.1 Hazard

If a fill spout is used at an AQUATIC VENUE, the fill spout shall be located so that it is not a SAFETY hazard to BATHERS.

4.11.2.2 Shielded

A fill spout should be located so the possibility of it becoming a trip hazard is minimized.

4.11.2.3 Open End

The open end of fill spouts shall not have sharp edges or protrude more than two inches *(50.8 mm)* beyond the edge of the POOL.

4.11.2.4 Air Gap

The open end shall be separated from the water by an air gap of at least 1.5 pipe diameters measured from the pipe outlet to the POOL.

4.11.3 Cross-Connection Control

4.11.3.1 Protected

The potable water supply serving an AQUATIC VENUE shall be protected against BACKFLOW consisting of either of the following:

1) An acceptable air gap consisting of a vertical distance of not less than two pipe diameters of the water supply pipe or six inches *(15.2 cm)*, whichever is greater, over the lowest free-flowing discharge point of the receiving pipe, tank, or vessel. Splash guards that are open to the atmosphere may be used around the air gap, or
2) Where permitted, an approved reduced pressure zone *(RPZ)* BACKFLOW preventer installed according to the plumbing CODE and the AHJ.

4.11.4 Deck Drains and Rinse Showers

4.11.4.1 Sloped Walkway

The walkway or DECK around an AQUATIC VENUE shall be properly sloped to DECK drains or to the edge of the DECK to prevent the accumulation of standing water.

4.11.4.2 Discharge

If DECK drains are provided, the drains shall discharge to the sanitary or storm sewer or as otherwise allowed by the AHJ and according to applicable plumbing CODES.

4.11.4.3 Area or Linear

DECK drains may be either area drains or linear drains. Refer to MAHC Section 4.8.1.3 for DECK drain area, and other requirements.

4.11.4.4 Rinse Showers

RINSE SHOWER drains shall discharge to the sanitary or storm sewer as allowed by the AHJ and according to applicable plumbing CODES.

4.11.5 Sanitary Wastes

4.11.5.1 Discharged

Wastewater from all PLUMBING FIXTURES in the entire AQUATIC FACILITY shall be discharged to a municipal sanitary sewer system, if available.

4.11.5.2 On-Site Sewer System

If a municipal sanitary sewer system is not available, all wastewater shall be disposed to an on-site sewer system that is properly designed to receive the entire wastewater capacity.

4.11.6 Pool Wastewater

4.11.6.1 Discharged

Wastewater from an AQUATIC VENUE, including filter backwash water, shall be discharged to a sanitary sewer system having sufficient capacity to collect and treat wastewater or to an on-site sewage disposal system designed for this purpose.

4.11.6.1.1 Storm Water Systems and Surface Waters

Wastewater shall not be directed to storm water systems or surface waters without appropriate permits from the AHJ or the U.S. EPA.

4.11.6.1.2 Recovery and Reuse

A water recovery and reuse system may be submitted to the AHJ for review and approval.

4.11.6.2 Ground Surface

If a municipal sanitary sewer system is not available, wastewater from an AQUATIC VENUE may be discharged to the ground surface at a suitable location as approved by the AHJ, as long as the wastewater does not cause erosion, and does not create a threat to public health or SAFETY, a nuisance, or unlawful pollution of public waters.

4.11.6.3 Capacity

The wastewater disposal system shall have sufficient capacity to receive wastewater without flooding when filters are cleaned or when the AQUATIC VENUE is drained.

4.11.6.4 Separation Tank for Precoat Media Filters

A separation tank shall be provided prior to discharge for backwash water from precoat filters using diatomaceous earth *(DE)* as a filter medium.

4.11.6.4.1 Discharged

For precoat filters using perlite or cellulose as a filter medium, the backwash may be discharged to the sanitary sewer, unless directed otherwise by the local AHJ.

4.12 Specific Venues

4.12.1 Spas

4.12.1.1 Additional Provisions

In addition to the general AQUATIC VENUE requirements stated in this CODE, SPAS shall comply with the additional provisions or reliefs of this section.

4.12.1.2 Maximum Water Depth

The maximum water depth in SPAS shall be four feet *(1.2 m)* measured from the designed static water line except for SPAS that are designed for special use and purposes and approved by the AHJ.

4.12.1.2.1 Exercise Spas

The water depth for exercise SPAS shall not exceed six feet six inches *(2.0 m)* measured from the designed static water line.

4.12.1.2.2 Seating

The maximum submerged depth of any seat or sitting bench shall be 28 inches *(71.1 cm)* measured from the water line.

4.12.1.3 Handholds

A SPA shall have one or more suitable, slip-resistant handhold*(s)* around the perimeter and not over 12 inches *(30.5 cm)* above the water line.

4.12.1.3.1 Options

The handhold*(s)* may consist of bull-nosed coping, ledges or DECKS along the immediate top edge of the SPA; ladders, steps, or seat ledges; or railings.

4.12.1.4 Stairs

Interior steps or stairs shall be provided where SPA depths are greater than 24 inches *(61.0 cm)*.

4.12.1.4.1 Handrail

Each set of steps shall be provided with at least one handrail to serve all treads and risers.

4.12.1.4.2 Seating

Seats or benches may be provided as part of these steps.

4.12.1.4.3 Approach Steps

Approach steps on the exterior of a SPA wall extending above the DECK shall also be required unless the raised SPA wall is 19 inches *(48.3 cm)* or less in height above the DECK and it is used as a transfer tier or pivot-seated entry.

4.12.1.5 Perimeter Deck

A four foot *(1.2 m)* wide, continuous, unobstructed PERIMETER DECK shall be provided on two consecutive or adjacent sides or fifty percent or more of the SPA perimeter.

4.12.1.5.1 Lower Ratio

The AHJ could consider a lower ratio upon review of an appropriate SAFETY PLAN that addresses adequate access.

4.12.1.5.2 Coping

The PERIMETER DECK may include the coping.

4.12.1.5.3 Recessed

SPAS may be located adjacent to other AQUATIC VENUES as long as they are recessed in the DECK.

4.12.1.5.4 Elevated Spas

Elevated SPAS may be located adjacent to another AQUATIC VENUE as long as there is an effective BARRIER between the SPA and the adjacent AQUATIC VENUE.

4.12.1.5.5 Minimum Distance

If an effective BARRIER is not provided, a minimum distance of four feet *(1.2 m)* between the AQUATIC VENUE and SPA is required.

4.12.1.6 Depth Markers

A minimum of two depth markers shall be provided regardless of the shape or size of the SPA.

4.12.1.7 Temperature

Water temperatures shall not exceed 104°F *(40°C)*.

4.12.1.8 Drain

A means to drain the SPA shall be provided to allow frequent draining and cleaning.

4.12.1.9 Air Induction System

An air induction system, when provided, shall prevent water back up that could cause electrical shock hazards.

4.12.1.9.1 Intake

Air intake sources shall not permit the introduction of toxic fumes or other CONTAMINANTS.

4.12.1.10 Timers

The agitation system shall be connected to a minute timer that does not exceed 15 minutes that shall be located out of reach of a BATHER in the SPA.

4.12.1.11 Emergency Shutoff

All SPAS shall have a clearly labeled emergency shutoff or control switch for the purpose of stopping the motor*(s)* that provide power to the RECIRCULATION SYSTEM and hydrotherapy or agitation system that shall be installed and be readily accessible to the BATHERS, in accordance with the NEC.

4.12.2 Waterslides and Landing Pools

4.12.2.1 Design and Construction

4.12.2.1.1 Additional Provisions

In addition to the general AQUATIC FACILITY requirements stated in this CODE, WATERSLIDES and LANDING POOLS shall comply with the additional provisions or reliefs of this section.

4.12.2.1.2 Recognized Standards

The following recognized design and construction standards for WATERSLIDES shall be adhered to.

4.12.2.1.2.1 Engineer Compliance

The design engineer shall address compliance with these standards and must provide documentation and/or certification that the WATERSLIDE design is in conformance with these standards:

1) ASTM F2376-13 *Standard Practice for Classification, Design, Manufacture, Construction, and Operation of Water Slide Systems; and*
2) ASTM F2469-09 *Standard Practice for Manufacturer, Construction, Operation, and Maintenance of Aquatic Play Equipment*.

4.12.2.1.2.2 Required Signage

Signs indicating riding instructions, warnings, and requirements in accordance with the manufacturer recommendations shall be posted at the WATERSLIDE entry.

4.12.2.2 Flumes

4.12.2.2.1 Surfaces

FLUME surfaces shall be inert, nontoxic, smooth, and easily cleaned.

4.12.2.2.2 Dips

All FLUME VALLEYS and DIPS shall have proper drainage, SAFETY measures that insure a rider cannot fall from the FLUME, and a means of egress in the event the ride malfunctions or a rider stops on the ride.

4.12.2.3 Flume Exits

4.12.2.3.1 Landing Pool

The exit of any FLUME must be designed to ensure that BATHERS enter the LANDING POOL or slide RUNOUT at a safe speed and angle of entry.

4.12.2.3.2 Intersection

If a WATERSLIDE has two or more FLUMES and there is a point of intersection between the centerlines of any two FLUMES, the distance between that point and the point of exit for each intersecting FLUME must not be less than the slide manufacturer's recommendations and ASTM F2376.

4.12.2.4 Exit into Landing Pools

4.12.2.4.1 Water Level

WATERSLIDES shall be designed to terminate at or below water level, except for DROP SLIDES or unless otherwise permitted by the WATERSLIDE manufacturer and ASTM F2376.

4.12.2.4.2 Perpendicular

WATERSLIDES shall be perpendicular to the wall of the AQUATIC VENUE at the point of exit unless otherwise permitted by the WATERSLIDE manufacturer.

4.12.2.4.3 Exit System

WATERSLIDES shall be designed with an exit system which shall be in accordance with the WATERSLIDE manufacturer's recommendations and ASTM F2376 and provides for safe entry into the LANDING POOL or WATERSLIDE RUNOUT.

4.12.2.4.4 Flume Exits

The FLUME exits shall be in accordance with the WATERSLIDE manufacturer's recommendations and ASTM F2376.

4.12.2.4.5 Point of Exit

The distance between the point of exit and the side of the AQUATIC VENUE opposite the BATHERS as they exit, excluding any steps, shall not be less than the WATERSLIDE manufacturer's recommendations and in accordance with ASTM F2376.

4.12.2.5 Landing Pools

4.12.2.5.1 Steps

If steps are provided instead of exit ladders or RECESSED STEPS with grab rails, they shall be installed at the opposite end of the LANDING POOL from the FLUME exit and a handrail shall be provided.

4.12.2.5.2 Landing Area

If the WATERSLIDE FLUME ends in a swimming POOL, the landing area shall be divided from the rest of the AQUATIC VENUE by a float line, WING WALL, PENINSULA or other similar feature to prevent collisions with other BATHERS.

4.12.2.6 Decks

A PERIMETER DECK shall be provided along the exit side of the LANDING POOL.

4.12.2.7 Means of Access

A walkway, steps, stairway or ramp shall be provided between the LANDING POOL and the top of the FLUME. Refer to MAHC Section 4.8.1.

4.12.2.8 Slide Runouts

4.12.2.8.1 Egress

WATERSLIDE RUNOUTS, if used, shall have a planned means of egress, unless one of the walls of the RUNOUT is not more than 19 inches *(48.3 cm)* in height.

4.12.2.8.2 Designed

WATERSLIDE RUNOUTS shall be designed in accordance with the slide manufacturer's recommendations and ASTM F2376.

4.12.2.9 Drop Slides

4.12.2.9.1 Landing Area

There shall be a slide landing area in accordance with the slide manufacturer's recommendations and ASTM F2376.

4.12.2.9.2 Area Clearance

This area shall not infringe on the landing area for any other slides, diving equipment, or any other minimum AQUATIC VENUE clearance requirements.

4.12.2.9.3 Steps

Steps shall not infringe on this area.

4.12.2.9.4 Water Depth

The minimum required water depth shall be a function of the vertical distance between the terminus of the slide surface and the water surface of the landing pool.

4.12.2.9.5 Manufacturer's Recommendation

The minimum required water depth shall be in accordance with the slide manufacturer's recommendations and ASTM F2376.

4.12.2.10 Pool Slides

4.12.2.10.1 Designed for Safety

All slides installed as an appurtenance to an AQUATIC VENUE shall be designed, constructed, and installed to provide a safe environment for all BATHERS utilizing the AQUATIC VENUE in accordance with applicable ASTM and CPSC STANDARDS.

4.12.2.10.2 Non-Toxic

Components used to construct a POOL SLIDE shall be non-toxic and compatible with the environment contacted under normal use.

4.12.2.10.3 Water Depth

Water depth at the slide terminus shall be determined by the slide manufacturer.

4.12.2.10.4 Pool Edge

Clear space shall be maintained to the POOL edge and other features per manufacturer requirements.

4.12.2.10.4.1 Landing Area

The landing area of the slide shall be protected through the use of a float line, WING WALL, PENINSULA or other similar impediment to prevent collisions with other BATHERS.

4.12.2.10.4.2 Prevent Bather Access

Netting or other barriers shall be provided to prevent BATHER access underneath POOL SLIDES where sufficient clearance is not provided.

4.12.2.10.4.3 Netting or Barrier

Such netting or other barrier shall be designed such that any underwater opening does not allow for the passage of a four inch *(10.2 cm)* ball and no opening can create a finger entrapment.

4.12.2.11 Signage

Warning signs in accordance with manufacturer's recommendations shall be provided.

4.12.3 Wave Pools

4.12.3.1 General

4.12.3.1.1 Additional Provisions

In addition to the general swimming POOL requirements stated in this CODE, WAVE POOLS shall comply with the additional provisions or reliefs of this section.

4.12.3.2 Access

4.12.3.2.1 Access Point

BATHERS must gain access to the WAVE POOL at the shallow or beach end with the exception of an allowable ADA designated entry point.

4.12.3.2.1.1 Sides

The sides of the WAVE POOL shall be protected from unauthorized entry into the WAVE POOL by the use of a fence or other comparable BARRIER.

4.12.3.2.1.2 Handrails

Handrails as required by ADA for accessible entries shall be designed in such a way that they do not present a potential for injury or entrapment with WAVE POOL BATHERS.

4.12.3.2.2 Perimeter Decks

A PERIMETER DECK shall not be required around 100% of the WAVE POOL perimeter.

4.12.3.2.2.1 Wave Pool Access

A PERIMETER DECK shall be provided where BATHERS gain access to the WAVE POOL at the shallow or beach end and in locations where access is required for lifeguards.

4.12.3.2.3 Handholds

WAVE POOLS shall be provided with handholds at the static water level or not more than six inches *(15.2 cm)* above the static water level.

4.12.3.2.3.1 Continuous

These handholds shall be continuous around the WAVE POOL'S perimeter with the exception of at the ZERO DEPTH BEACH ENTRY, water depths less than 24 inches *(61.0 cm),* if this area is roped off not allowed for BATHER access.

4.12.3.2.3.2 Self Draining

These handholds shall be self-draining.

4.12.3.2.3.3 Flush

Handholds shall be installed so that their outer edge is flush with the WAVE POOL wall.

4.12.3.2.3.4 Entangled

The design of the handholds shall ensure that body extremities will not become entangled during wave action.

4.12.3.2.4 Steps and Handrails

RECESSED STEPS shall not be allowed along the walls of the WAVE POOL due to the entrapment potential.

4.12.3.2.5 Ladders

Side wall ladders shall be utilized for egress only and shall be placed so they do not project beyond the plane of the wall surface.

4.12.3.2.6 Float Line

WAVE POOLS shall be fitted with a float line located to restrict access to the caisson wall if required by the WAVE POOL equipment manufacturer.

4.12.3.2.6.1 Exceptions

SAFETY rope and float lines typically required at shallow to deep water transitions shall not apply to WAVE POOLS.

4.12.3.3 Safety

4.12.3.3.1 Life Jackets

Proper STORAGE shall be provided for life jackets and all other equipment used in the WAVE POOL that will allow for thorough drying to prevent mold and other biological growth.

4.12.3.3.2 Shut-Off Switch

A minimum of two emergency shut-off switches to disable the wave action shall be provided, one on each side of the WAVE POOL.

4.12.3.3.2.1 Labeled and Accessible

These switches shall be clearly labeled and readily accessible to QUALIFIED LIFEGUARDS.

4.12.3.3.3 No Diving Sign

SAFETY rope and float lines typically required at shallow to deep water transitions shall not apply to WAVE POOLS.

4.12.3.3.4 Caution Signs

Caisson BARRIERS shall be provided for all WAVE POOLS that prevent the passage of a four-inch *(10.2 cm)* ball.

4.12.4 Therapy Pools

4.12.4.1 Additional Provisions

In addition to the general AQUATIC VENUE requirements stated in this CODE, THERAPY POOLS shall comply with the additional provisions or reliefs of this section and MAHC Section 4.7.3.3 on SECONDARY DISINFECTION.

4.12.4.2 Slope

Floor slope may exceed one foot *(30.5 cm)* in 12 feet *(3.7 m)* for water shallower than five feet *(1.5 m)*.

4.12.4.2.1 Break Points

Break points in floor slope shall be identified with a contrasting band consistent with MAHC Section 4.5.4.2.

4.12.4.3 Hydrotherapy

Hydrotherapy or jet systems shall be independent of the recirculation, filtration, and heating systems.

4.12.4.4 Special Equipment

Special equipment may be allowed by the AHJ with proper justification.

4.12.5 Lazy Rivers

4.12.5.1 General

4.12.5.1.1 Additional Provisions

In addition to the general swimming AQUATIC VENUE requirements stated in this CODE, LAZY RIVERS shall comply with the additional provisions or reliefs of this section.

4.12.5.1.2 Protrusions

Handrails, steps, stairs and propulsion jets for LAZY RIVERS shall not protrude into the river.

4.12.5.2 Access and Egress

4.12.5.2.1 Means

Means of access/egress shall be provided at 150 foot *(45.7 m)* intervals around the LAZY RIVER.

4.12.5.2.2 Handhold

A handhold in compliance with MAHC Section 4.5.5 shall be required on at least one side of the LAZY RIVER.

4.12.5.2.3 Deck

A DECK shall be provided along the entire length of the LAZY RIVER.

4.12.5.2.3.1 Alternate Sides

The DECK shall be allowed to alternate sides of the LAZY RIVER.

4.12.5.2.3.2 Obstructions

Obstructions around the perimeter of the LAZY RIVER, such as bridges or landscaping, shall be allowed provided they do not impact lifeguarding, sight lines, or rescue operations.

4.12.5.2.4 Bridges

All bridges spanning a LAZY RIVER shall have a minimum clearance of both seven feet *(2.1 m)* from the bottom of the LAZY RIVER and four feet *(1.2 m)* above the water surface to any structure overhead.

4.12.6 Moveable Floors

4.12.6.1 General

4.12.6.1.1 Additional Provisions

In addition to the general swimming AQUATIC VENUE requirements stated in this CODE, MOVEABLE FLOORS shall comply with the additional provisions or reliefs of this section.

4.12.6.1.2 Water Treatment

The MOVEABLE FLOOR design shall not impede the effectiveness of the water treatment system.

4.12.6.1.3 Underneath

MOVEABLE FLOORS shall allow inspection, cleaning and maintenance of the area underneath.

4.12.6.2 Slip Resistance

The surface of the MOVEABLE FLOOR shall be slip resistant if it is intended for installation in water depths less than five feet *(1.5 m)*.

4.12.6.3 Safety

4.12.6.3.1 Not Continuous

A strategy for preventing BATHERS from transitioning to deeper water when a MOVEABLE FLOOR is not continuous over the entire surface area of the AQUATIC VENUE shall be provided.

4.12.6.3.2 Underside

The underside of the MOVEABLE FLOOR shall not be accessible to BATHERS.

4.12.6.3.3 Entrapment

The design of a MOVEABLE FLOOR shall protect against BATHER entrapment between the MOVEABLE FLOOR and the POOL walls and floor.

4.12.6.3.4 Hydraulic Fluid

If the MOVEABLE FLOOR is operated using hydraulics, the hydraulic compounds shall be listed as safe for use in POOL water in case there is a hydraulic leak.

4.12.6.4 Movement

4.12.6.4.1 Speed

The speed of a MOVEABLE FLOOR shall be less than or equal to 1.5 feet per minute *(45.7 cm/min)*.

4.12.6.4.2 Use

Use of the MOVEABLE FLOOR portion of the POOL shall not be open to BATHERS when the floor is being raised or lowered.

4.12.6.4.2.1 Exception

Exception: The MOVEABLE FLOOR shall only be used for accessibility purposes under direct supervision.

4.12.6.5 Water Depth and Markings

4.12.6.5.1 Displayed

A floor depth indicator shall be provided that displays the current pool water depth.

4.12.6.5.2 Warning Markings

Warning markings stating "Moveable Floor" shall be provided at 25 foot *(7.6 m)* intervals around the perimeter of the MOVEABLE FLOOR.

4.12.7 Bulkheads

4.12.7.1 Additional Provisions

In addition to the general AQUATIC VENUE requirements stated in this CODE, BULKHEADS shall comply with the additional provisions or reliefs of this section.

4.12.7.2 Entrapment

The bottom of the BULKHEAD shall be designed so that a BATHER cannot be entrapped underneath or inside of the BULKHEAD.

4.12.7.3 Placement

The BULKHEAD placement shall not interfere with the required water circulation in the POOL.

4.12.7.4 Fixed

BULKHEADS shall be fixed to their operational position*(s)* by a tamper-proof system.

4.12.7.5 Gap

The gap between the BULKHEAD and the POOL wall shall be no greater than 1.5 inches *(3.8 cm)*.

4.12.7.6 Handhold

The BULKHEAD shall be designed to afford an acceptable handhold as required in MAHC Section 4.5.14.

4.12.7.7 Entrances and Exits

The proper number of entrances/exits to the POOL as required by MAHC section 4.5.3 shall be provided when the BULKHEAD is in place.

4.12.7.8 Guard Railings

Guard railings at least 34 inches *(86.4 cm)* tall shall be provided on both ends of the BULKHEAD.

4.12.7.9 Width

The width of the walkable area *(total bulkhead width)* of a BULKHEAD shall be greater than or equal to three feet and three inches *(1.0 m)*.

4.12.7.9.1 Starting Platforms

If starting platforms are installed, the width of the walkable area *(total bulkhead width)* of a BULKHEAD shall be greater than or equal to three feet and nine inches *(1.1 m)*.

4.12.7.9.1.1 Side Mount Style

Starting platforms shall be "side mount" style if BULKHEAD is less than four feet six inches *(1.4 m)* wide.

4.12.7.10 Bulkhead Travel

The travel of a BULKHEAD shall be in accordance with one of the following:

1) Limited such that it cannot encroach on any required clearances of other features, such as diving boards; or
2) Designed with modifications incorporated that prevent use of other features when the required clearances have been compromised by the position of the bulkhead.

4.12.8 Interactive Water Play Venues

4.12.8.1 Additional Provisions

In addition to the general AQUATIC VENUE requirements stated in this CODE, INTERACTIVE WATER PLAY VENUES shall comply with the additional provisions or reliefs of this section.

4.12.8.2 Surface

INTERACTIVE WATER PLAY VENUES shall have a slip-resistant and easily cleanable surface.

4.12.8.2.1 Manufactured Surfacing

Any manufactured surfacing shall be deemed suitable by the manufacturer for aquatic and chlorinated environments.

4.12.8.3 Sloped

The INTERACTIVE WATER PLAY VENUE shall be properly sloped so that only water from the AQUATIC FEATURES flows back to the INTERACTIVE WATER PLAY VENUE collection tank.

4.12.8.3.1 Adjacent Areas

Areas adjacent to the INTERACTIVE WATER PLAY VENUE shall be sloped away from the collection drains.

4.12.8.3.2 Water Collection

The slope of the INTERACTIVE WATER PLAY VENUE shall be sufficient to prevent standing water from collecting on the pad.

4.12.8.4 Drains

The size, number and locations of the INTERACTIVE WATER PLAY VENUE drains shall be determined and specified so as to assure water does not accumulate on the INTERACTIVE WATER PLAY VENUES.

4.12.8.4.1 Gravity

Flow through the drains to the INTERACTIVE WATER PLAY VENUE COLLECTION TANK shall be under gravity.

4.12.8.4.2 Direct Suction Outlets

Direct suction outlets from the INTERACTIVE WATER PLAY VENUE shall be prohibited.

4.12.8.5 Grate Openings

Openings in the grates covering the drains shall not exceed ½ inches *(12.7 mm)* wide.

4.12.8.5.1 Tools

Gratings shall not be removable without the use of tools.

4.12.8.6 Treatment Tank

The INTERACTIVE WATER PLAY VENUE COLLECTION TANK shall be designed to provide ready access for cleaning and inspections, and

4.12.8.6.1 Completely Drain

The INTERACTIVE WATER PLAY VENUE COLLECTION TANK shall be capable of complete draining.

4.12.8.6.2 Access Hatch

The access hatch or lid shall be locked or require a tool to open.

4.12.8.7 Deck Area

INTERACTIVE WATER PLAY VENUES shall be kept free of landscape debris by either:

1) Eight feet *(2.4 m)* of DECK area, or
2) Raised curbs, or
3) Raised planters.

4.12.8.7.1 Deck Surface

The DECK shall be of a uniform, easily cleaned, impervious material

4.12.8.7.2 Protected From Runoff

The DECK shall be protected from surface runoff.

4.12.8.8 Barrier

A BARRIER shall be provided to separate an INTERACTIVE WATER PLAY VENUE from another BODY OF WATER within the same facility

Exception: The INTERACTIVE WATER PLAY VENUE is separated by a distance of at least 15 feet *(4.6 m)* from other BODIES OF WATER.

4.12.8.9 Enclosures

If a facility only consists of an INTERACTIVE WATER PLAY VENUE, then the requirements for an ENCLOSURE shall not apply.

4.12.8.10 Hazard

Spray features shall be designed and installed to be seen clearly, so as not to be a hazard to BATHERS due to water velocity from the spray feature discharge, or other SAFETY hazards.

4.12.8.11 Maximum Velocity

Maximum velocity at the orifice of the SPRAY FEATURE nozzle shall not exceed 20 feet *(6.1 m)* per second.

4.12.8.12 Signage

Depth markings and warning signs shall not be required for INTERACTIVE WATER PLAY VENUES.

4.12.8.13 NEC Requirements

NEC swimming POOL requirements shall apply to Interactive water play venues.

4.12.9 Wading Pools

4.12.9.1 Additional Provisions

In addition to the general AQUATIC VENUE requirements stated in this CODE, WADING POOLS shall comply with the additional provisions or reliefs of this section.

4.12.9.2 Barrier

A BARRIER shall be provided to separate a WADING POOL from other POOLS unless the WADING POOL is separated by a distance of 15 feet *(4.6 m)* from other BODIES OF WATER.

4.12.9.2.1 Complete Enclosure

The BARRIER shall not be required to completely surround the WADING POOL if the shortest distance of travel between the WADING POOL around the BARRIER to the other POOL is a minimum of 15 feet *(4.67 m)*.

4.12.9.2.2 Shallow Water

WADING POOLS near other WADING POOLS shall not be required to be separated by a BARRIER.

The Model Aquatic Health Code

The Code

OPERATION AND MAINTENANCE

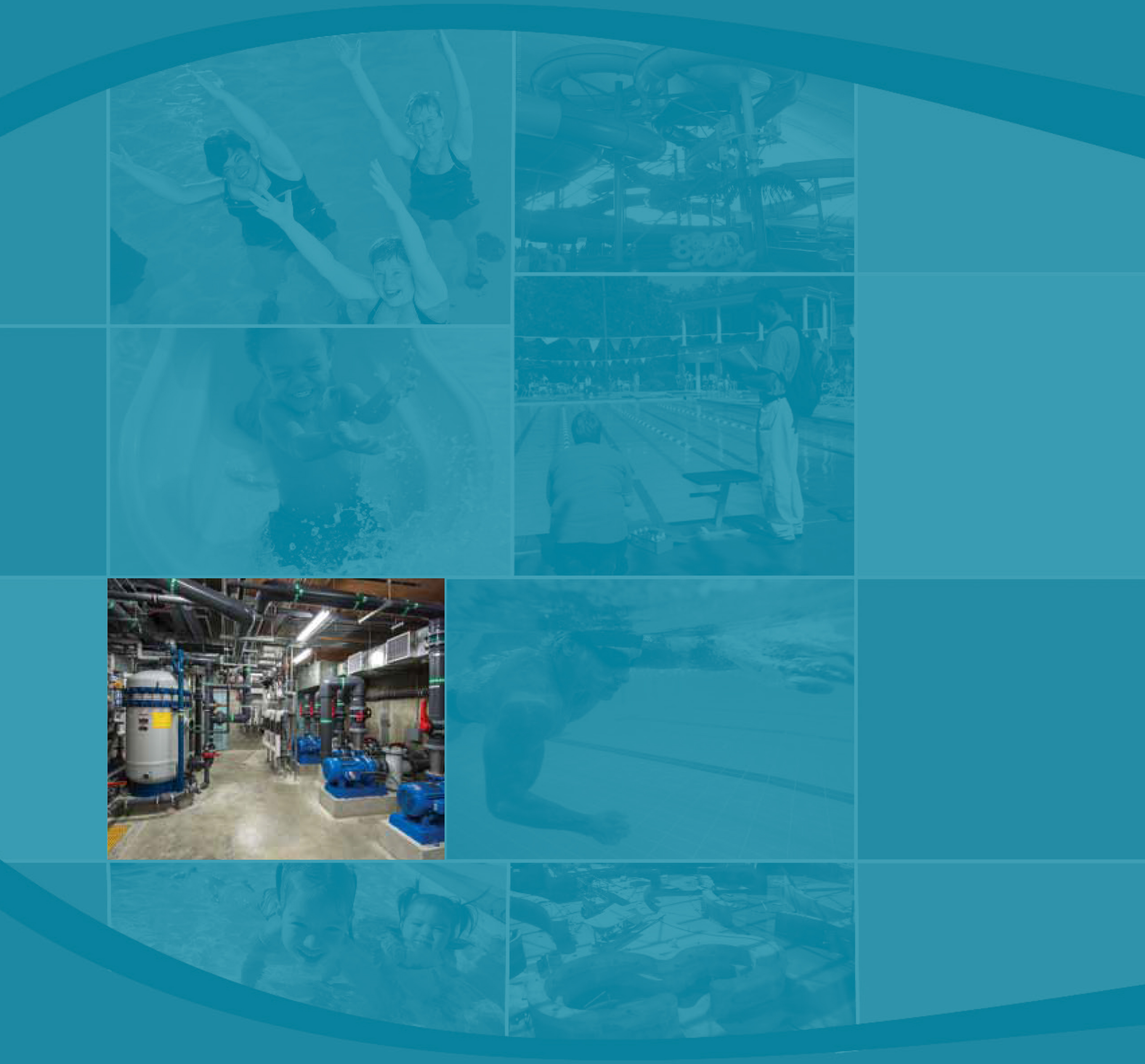

CS249779

5.0 Facility Operation and Maintenance

The provisions of Chapter 5 apply to all AQUATIC FACILITIES covered by this CODE regardless of when constructed, unless otherwise noted.

5.1 Operating Permits

5.1.1 Owner Responsibilities

5.1.1.1 Permit to Operate Required

Prior to opening to the public, the AQUATIC FACILITY owner shall apply to the AHJ for a permit to operate.

5.1.1.2 Separate

A separate permit is required for each newly constructed or SUBSTANTIALLY ALTERED AQUATIC VENUE at an existing AQUATIC FACILITY.

5.1.1.3 Prior to Issuance

Before a permit to operate is issued, the following procedures shall be completed:

1) The AQUATIC FACILITY owner has demonstrated the AQUATIC FACILITY, including all newly constructed or SUBSTANTIALLY ALTERED AQUATIC VENUES, is in compliance with the requirements of this CODE, and
2) The AHJ has approved the AQUATIC FACILITY to be open to the public.

5.1.1.4 Permit Details

The permit to operate shall:

1) Be issued in the name of the owner,
2) List all AQUATIC VENUES included under the permit, and
3) Specify the period of time approved by the AHJ.

5.1.1.5 Permit Expiration

Permits to operate shall terminate according to the AHJ schedule.

5.1.1.6 Permit Renewal

The AQUATIC FACILITY owner shall renew the permit to operate prior to the scheduled expiration of an existing permit to operate an AQUATIC FACILITY.

5.1.1.7 Permit Denial

The permit to operate may be withheld, revoked, or denied by the AHJ for noncompliance of the AQUATIC FACILITY with the requirements of this CODE.

5.1.1.8 Owner Responsibilities

The owner of an AQUATIC FACILITY is responsible for the facility being operated, maintained, and managed in accordance with the requirements of this CODE.

5.1.2 Operating Permits

5.1.2.1 Permit Location

The permit to operate shall be posted at the AQUATIC FACILITY in a location conspicuous to the public.

5.1.2.2 Operating Without a Permit

Operation of an AQUATIC FACILITY or newly constructed or SUBSTANTIALLY ALTERED AQUATIC VENUE without a permit to operate shall be prohibited.

5.1.2.3 Required Closure

The AHJ may order a newly constructed or SUBSTANTIALLY ALTERED AQUATIC VENUE without a permit to operate to close until the AQUATIC FACILITY has obtained a permit to operate.

5.2 Inspections

5.2.1 Preoperational Inspections

5.2.1.1 Terms of Operation

The AQUATIC FACILITY may not be placed in operation until an inspection approved by the AHJ shows compliance with the requirements of this CODE or the AHJ approves opening for operation.

5.2.2 Exemptions

5.2.2.1 Applying for Exemption

An AQUATIC FACILITY seeking an initial exemption or an existing AQUATIC FACILITY claiming to be exempt according to applicable regulations shall contact the AHJ for application details/forms.

5.2.2.2 Change in Exemption Status

An AQUATIC FACILITY that sought and received an exemption from a public regulation shall contact the AHJ if the conditions upon which the exemption was granted change so as to eliminate the exemption status.

5.2.3 Variances

5.2.3.1 Variance Authority

The AHJ may grant a variance to the requirements of this CODE.

5.2.3.2 Applying for a Variance

An AQUATIC FACILITY seeking a variance shall apply in writing with the appropriate forms to the AHJ.

5.2.3.2.1 Application Components

The application shall include, but not be limited to:

1) A citation of the CODE section to which the variance is requested;
2) A statement as to why the applicant is unable to comply with the CODE section to which the variance is requested;
3) The nature and duration of the variance requested;
4) A statement of how the intent of the CODE will be met and the reasons why the public health or SAFETY would not be jeopardized if the variance was granted, and
5) A full description of any policies, procedures, or equipment that the applicant proposes to use to rectify any potential increase in health or SAFETY risks created by granting the variance.

5.2.3.3 Revoked

Each variance shall be revoked when the permit attached to it is revoked.

5.2.3.4 Not Transferable

A variance shall not be transferable unless otherwise provided in writing at the time the variance is granted.

5.3 Equipment Standards [N/A]

5.4 Aquatic Facility and Venue Operation and Maintenance

5.4.1 Closure and Reopening

5.4.1.1 Closure

If an AQUATIC VENUE is not open to the public the following conditions shall be met to protect health and safety:

5.4.1.1.1 Aquatic Venues With a Barrier

Where the AQUATIC VENUE has a barrier enclosing it per MAHC Section 4.8.6:

1) The water shall be recirculated and treated to meet the criteria of this CODE, or
2) The water shall be drained; or
3) An approved SAFETY cover that is listed and labeled to ASTM F1346-91 by an ANSI-accredited certification organization shall be installed.

5.4.1.1.2 Aquatic Venues Without a Barrier but Open to the Public

Where the AQUATIC VENUE does not have a barrier enclosing it per MAHC Section 4.8.6 and other parts of the AQUATIC FACILITY are open to the public:

1) The water shall be recirculated and treated to meet the criteria of this CODE and the AQUATIC VENUE shall be staffed to keep bathers out, or
2) An approved SAFETY cover that is listed and labeled to ASTM F1346-91 by an ANSI-accredited certification organization shall be installed.

5.4.1.1.3 Aquatic Venues Without a Barrier and Closed to the Public

Where the AQUATIC VENUE does not have a BARRIER enclosing it per MAHC 4.8.6, and the AQUATIC FACILITY is closed to the public:

1) The water shall be recirculated and treated to meet the criteria of this code, or
2) The water shall be drained; or
3) An approved **SAFETY** cover listed and labeled to ASTM F1346-91 by an ANSI-accredited certification organization shall be installed

5.4.1.2 Reopening

An owner or operator of a closed AQUATIC VENUE shall verify that the AQUATIC VENUE meets all applicable criteria of this CODE before reopening the AQUATIC VENUE.

5.4.2 Preventive Maintenance Plan

5.4.2.1 Written Plan

5.4.2.1.1 Preventive Maintenance Plan Available

A written comprehensive preventive maintenance plan for each AQUATIC VENUE shall be available at the AQUATIC FACILITY.

5.4.2.1.2 Contents

The AQUATIC FACILITY preventive maintenance plan shall include details and frequency of owner/operator's planned routine facility inspection, maintenance, and replacement of recirculation and water treatment components.

5.4.2.2 Facility Documentation

5.4.2.2.1 Original Plans and Specifications Available

A copy of the approved plans and specifications for each AQUATIC VENUE constructed after the adoption of this CODE shall be available at the AQUATIC FACILITY

5.4.2.2.2 Equipment Inventory

A comprehensive inventory of all mechanical equipment associated with each AQUATIC VENUE shall be available at the AQUATIC FACILITY.

5.4.2.2.3 Inventory Details

This inventory shall include:

1) Equipment name and model number,
2) Manufacturer and contact information,
3) Local vendor/supplier and technical representative, if applicable, and
4) Replacement or service dates and details.

5.4.2.2.4 Equipment Manuals

Operation manuals for all mechanical equipment associated with each AQUATIC VENUE shall be available at the AQUATIC FACILITY.

5.4.2.2.4.1 No Manual

If no manufacturer's operation manual is available, then the AQUATIC FACILITY should create a written document that outlines standard operating procedures for maintaining and operating the piece of equipment.

5.4.3 General Operations [N/A]

5.5 Aquatic Venue Structure

5.5.1 Shape [N/A]

5.5.2 Access Ladders [N/A]

5.5.3 Color and Finish [N/A]

5.5.4 Walls [N/A]

5.5.5 Depth Markings

5.5.5.1 Depth Markers

Depth markers shall be provided in locations in accordance with MAHC Section 4.5.19 and maintained.

5.5.5.2 No Diving Markers

No Diving markers shall be provided in accordance with MAHC Section 4.5.19 and maintained.

5.5.6 Pool Shell Maintenance

5.5.6.1 Cracking

5.5.6.1.1 Repaired

CRACKS shall be repaired when they may increase the potential for:

1) Leakage,
2) Trips or falls,
3) Lacerations, or
4) Impact the ability to properly clean and maintain the AQUATIC VENUE area.

5.5.6.1.2 Document Cracks

Surface CRACKS under 1/8 inch *(3.2 mm)* wide shall be documented and monitored for any movement or change including opening, closing, and/or lengthening.

5.5.6.1.3 Sharp Edges

Any sharp edges shall be removed.

5.6 Indoor / Outdoor Environment

5.6.1 Lighting

5.6.1.1 Lighting Maintained

5.6.1.1.1 Light Levels

Lighting systems, including emergency lighting, shall be maintained in all PATRON areas and maintenance areas, to ensure the required lighting levels are met as specified in MAHC Section 4.6.1.

5.6.1.1.2 Main Drain Visible

The AQUATIC FACILITY shall not be open if light levels are such that the main drain is not visible from poolside.

5.6.1.1.3 Underwater Lighting

Underwater lights, where provided, shall be operational and maintained as designed.

5.6.1.1.4 Cracked Lenses

Cracked lenses that are physically intact on lights shall be replaced before the AQUATIC VENUE reopens to BATHERS.

5.6.1.1.5 Intact Lenses

The AQUATIC VENUE shall be immediately closed if cracked lenses are not intact and the lenses shall be replaced before re-opening.

5.6.1.2 Glare

5.6.1.2.1 Assessments

The AQUATIC FACILITY owner shall ensure that glare conditions are assessed to determine if the AQUATIC VENUE bottom and objects in the POOL are clearly visible throughout operating hours.

5.6.1.2.1.1 Lifeguard Stations

If the AQUATIC VENUE requires lifeguards, the AQUATIC FACILITY owner shall ensure that glare conditions are assessed from each lifeguard station to determine if the AQUATIC VENUE bottom and objects in the POOL are clearly visible throughout operating hours.

5.6.1.2.2 Reduction

Windows and lighting equipment shall be adjusted, if possible, to minimize glare and excessive reflection on the water surface.

5.6.1.3 Night Swimming

Night swimming shall be prohibited unless required light levels in accordance with MAHC Section 4.6.1 are provided.

5.6.1.3.1 Hours

Night swimming shall be considered one half hour before sunset to one half hour after sunrise.

5.6.1.4 Emergency Lighting

Emergency lighting shall be tested and maintained according to manufacturer's recommendations.

5.6.2 Indoor Aquatic Facility Ventilation

5.6.2.1 Purpose

AIR HANDLING SYSTEMS shall be maintained and operated by the owner/operator to protect the health and SAFETY of the facility's PATRONS.

5.6.2.2 Original Characteristics

AIR HANDLING SYSTEMS shall be maintained and operated to comply with all requirements of the original system design, construction, and installation.

5.6.2.3 Indoor Facility Areas

The AIR HANDLING SYSTEM operation and maintenance requirements shall apply to an INDOOR AQUATIC FACILITY including:

1) The AQUATIC VENUES, and
2) The surrounding BATHER and spectator/stadium seating area;

But does not include:

1) Mechanical rooms,
2) Bath and locker rooms, and
3) Any associated rooms which have a direct opening to the AQUATIC FACILITY.

5.6.2.4 Ventilation Procedures

THE INDOOR AQUATIC FACILITY owner/operator shall develop and implement a program of standard AIR HANDLING SYSTEM operation, maintenance, cleaning, testing, and inspection procedures with detailed instructions, necessary equipment and supplies, and oversight for those carrying out these duties, in accordance with the AIR HANDLING SYSTEM design engineer and/or manufacturer's recommendations.

5.6.2.4.1 System Operation

The AIR HANDLING SYSTEM shall operate continuously, including providing the required amount of outdoor air.

5.6.2.4.1.1 Operation Outside of Operating Hours

Exception: During non-use periods, the amount of outdoor air may be reduced by no more than 50% as long as acceptable air quality is maintained.

5.6.2.5 Manuals/Commissioning Reports

The QUALIFIED OPERATOR shall maintain a copy of the AIR HANDLING SYSTEM design engineer and/or manufacturer original operating manuals, commissioning reports, updates, and specifications for any modifications at the facility.

5.6.2.6 Ventilation Monitoring

The QUALIFIED OPERATOR shall monitor, log and maintain AIR HANDLING SYSTEM set-points and other operational parameters as specified by the AIR HANDLING SYSTEM design engineer and/or manufacturer.

5.6.2.7 Air Filter Changing

The QUALIFIED OPERATOR*(s)* shall replace or clean, as appropriate, AIR HANDLING SYSTEM air filters in accordance with the AIR HANDLING SYSTEM design engineer and/or manufacturer's recommendations, whichever is most frequent.

5.6.2.8 Combined Chlorine Reduction

The QUALIFIED OPERATOR shall develop and implement a plan to minimize combined CHLORINE compounds in the INDOOR AQUATIC FACILITY from the operation of AQUATIC VENUES.

5.6.2.9 Building Purge Plan

The QUALIFIED OPERATOR shall develop and implement an air quality action plan with procedures for PURGING the INDOOR AQUATIC FACILITY for chemical emergencies or other indicators of poor air quality.

5.6.2.10 Records

The owner shall ensure documents are maintained at the INDOOR AQUATIC FACILITY to be available for inspection, recording the following:

1) A log recording the set points of operational parameters set during the commissioning of the AIR HANDLING SYSTEM and the actual readings taken at least once daily;
2) Maintenance conducted to the system including the dates of filter changes, cleaning, and repairs;
3) Dates and details of modifications to the AIR HANDLING SYSTEM; and
4) Dates and details of modifications to the operating scheme.

5.6.3 Electrical

5.6.3.1 Electrical Repairs

5.6.3.1.1 Local Codes

Repairs or alterations to electrical equipment and associated equipment shall preserve compliance with the NEC, or with applicable local CODES prevailing at the time of construction, or with subsequent versions of those CODES.

5.6.3.1.2 Immediately Repaired

All defects in the electrical system shall be immediately repaired.

5.6.3.1.3 Wiring

Electrical wiring, whether permanent or temporary, shall comply with the NEC or with applicable local CODE.

5.6.3.2 Electrical Receptacles

5.6.3.2.1 New Receptacles

The installation of new electrical receptacles shall be subject to electrical-construction requirements of this CODE and applicable local CODE.

5.6.3.2.2 Repairs

Repairs or maintenance to existing receptacles shall maintain compliance with the NEC and with CFR 1910.304(b) (3) (ii).

5.6.3.2.3 Replacement

Replacement receptacles shall be of the same type as the previous ones, e.g. grounding-type receptacles shall be replaced only by grounding-type receptacles, with all grounding conductors connected and proper wiring polarity preserved.

5.6.3.2.4 Substitutions

Where the original-type of receptacle is no longer available, a replacement and installation shall be in accordance with applicable local CODE.

5.6.3.3 Ground-Fault Circuit Interrupter

5.6.3.3.1 Manufacturer's Recommendations

Where receptacles are required to be protected by GFCI devices, the GFCI devices shall be tested following the manufacturer's recommendations.

5.6.3.3.2 Permanent Facilities

For permanent AQUATIC FACILITIES, required GFCI devices shall be tested monthly as part of scheduled maintenance.

5.6.3.3.3 Testing

Required GFCI devices shall be tested as part of scheduled maintenance on the first day of operation, and monthly thereafter, until the BODY OF WATER is drained and the equipment is prepared for STORAGE.

5.6.3.4 Grounding

5.6.3.4.1 Maintenance and Repair

Maintenance or repair of electrical circuits or devices shall preserve grounding compliance with the NEC or with applicable local CODES.

5.6.3.4.2 Grounding Conductors

Grounding conductors that have been disconnected shall be re-inspected as required by the local building CODE authority prior to AQUATIC VENUE being used by BATHERS.

5.6.3.4.3 Damaged Conductors

Damaged grounding conductors and grounding electrodes shall be repaired immediately.

5.6.3.4.4 Damaged Conductor Repair

Damaged grounding conductors or grounding electrodes associated with recirculation or DISINFECTION equipment or with underwater lighting systems shall be repaired by a qualified person who has the proper and/or necessary skills, training, or credentials to carry out the this task.

5.6.3.4.5 Public Access

The public shall not have access to the AQUATIC VENUE until such grounding conductors or grounding electrodes are repaired.

5.6.3.4.6 Venue Closure

The AQUATIC VENUE with damaged grounding conductors or grounding electrodes, that are associated with recirculation or DISINFECTION equipment or with underwater lighting systems, shall be closed until repairs are completed and inspected by the AHJ.

5.6.3.5 Bonding

5.6.3.5.1 Local Codes

Maintenance or repair of all metallic equipment, electrical circuits or devices, or reinforced concrete structures shall preserve bonding compliance with the NEC, or with applicable local CODES.

5.6.3.5.2 Bonding Conductors

Bonding conductors shall not be disconnected except where they will be immediately reconnected.

5.6.3.5.3 Disconnected Conductors

The AQUATIC VENUE shall not be used by BATHERS while bonding conductors are disconnected.

5.6.3.5.4 Removable Covers

Removable covers protecting bonding conductors, e.g. at ladders, shall be kept in place except during bonding conductor inspections, repair, or replacement.

5.6.3.5.5 Scheduled Maintenance

Bonding conductors, where accessible, shall be inspected semi-annually as part of scheduled maintenance.

5.6.3.5.6 Corrosion

Bonding conductors and any associated clamps shall not be extensively corroded.

5.6.3.5.7 Continuity

Continuity of the bonding system associated with RECIRCULATION SYSTEM or DISINFECTION equipment or with underwater lighting systems shall be inspected by the AHJ following installation and any major construction around the AQUATIC FACILITY.

5.6.3.6 Extension Cords

5.6.3.6.1 Temporary Cords and Connectors

Temporary extension cords and power connectors shall not be used as a substitute for permanent wiring.

5.6.3.6.2 Minimum Distance from Water

All parts of an extension cord shall be restrained at a minimum of six feet *(1.8 m)* away when measured along the shortest possible path from a BODY OF WATER during times when the AQUATIC FACILITY is open.

5.6.3.6.3 Exception

An extension cord may be used within six feet *(1.8 m)* of the nearest edge of a BODY OF WATER if a permanent wall exists between the BODY OF WATER and the extension cord.

5.6.3.6.4 GFCI Protection

The circuit supplying an extension cord shall be protected by a GFCI device when the extension cord is to be used within six feet *(1.8 m)* of a BODY OF WATER.

5.6.3.6.5 Local Code

An extension cord incorporating a GFCI device may be used if that is acceptable under applicable local CODE.

5.6.3.6.6 Compliance

The use of extension cords shall comply with CFR 1910.304.

5.6.3.7 Portable Electric Devices

Portable line-powered electrical devices, such as radios or drills, shall not be used within six feet *(1.8 m)* horizontally of the nearest inner edge of a BODY OF WATER, unless connected to a GFCI-protected circuit.

5.6.3.8 Communication Devices and Dispatch Systems

The maintenance and repair of Communication Devices and Dispatch Systems shall preserve compliance with the NEC.

5.6.4 Facility Heating

5.6.4.1 Facility Heating

5.6.4.1.1 Maintenance and Repair

Maintenance, repairs, and alterations to facility-heating equipment shall preserve compliance with applicable CODES.

5.6.4.1.2 Defects

Defects in the AQUATIC FACILITY heating equipment shall be immediately repaired.

5.6.4.1.3 Temperature

Air temperature of an INDOOR AQUATIC FACILITY shall be controlled to the original specifications or in the absence of such, maintain the dew point of the INTERIOR SPACE less than the dew point of the interior walls at all times so as to prevent damage to structural members and to prevent biological growth on walls.

5.6.4.1.4 Combustion Device

Items shall not be stored within the COMBUSTION DEVICE manufacturer's specified minimum clearance distance.

5.6.4.2 Water Heating

Maintenance, repairs, and alterations to POOL-water heating equipment shall preserve compliance with applicable CODES.

5.6.5 First Aid Room [N/A]

5.6.6 Emergency Exit

5.6.6.1 Exit Routes

Emergency exit routes shall be established for both INDOOR FACILITIES and OUTDOOR FACILITIES and be maintained so that they are well lit, unobstructed, and accessible at all times.

5.6.7 Plumbing

5.6.7.1 Water Supply

5.6.7.1.1 Water Pressure

All plumbing shall be maintained in good repair with no leaks or discharge.

5.6.7.1.2 Availability

Potable water shall be available at all times to PATRONS.

5.6.7.1.3 Cross-Connection Control

Water introduced into the pool , either directly or to the recirculation system, shall be supplied through an air gap or by another method which will prevent backflow and back-siphonage.

5.6.7.2 Drinking Fountains

5.6.7.2.1 Good Repair

Drinking fountains shall be in good repair.

5.6.7.2.2 Clean

Drinking fountains shall be clean.

5.6.7.2.3 Catch Basin

Drinking fountains shall be adjusted so that water does not go outside the catch basin.

5.6.7.2.4 Contamination

Drinking fountains shall provide an angled jet of water and be adjusted so that the water does not fall back into the drinking water stream.

5.6.7.2.5 Water Pressure

Drinking fountains shall have sufficient water pressure to allow correct adjustment.

5.6.7.3 Waste Water

5.6.7.3.1 Waste Water Disposal

AQUATIC VENUE waste water, including backwash water and cartridge cleaning water, shall be disposed of in accordance with local CODES.

5.6.7.3.2 Drainage

Waste water and backwash water shall not be returned to an AQUATIC VENUE or the AQUATIC FACILITY'S water treatment system.

5.6.7.3.3 Drain Line

Filter backwash lines, DECK drains, and other drain lines connected to the AQUATIC FACILITY or the AQUATIC FACILITY RECIRCULATION SYSTEM shall be discharged through an approved air gap.

5.6.7.3.4 No Standing Water

No standing water shall result from any discharge, nor shall they create a nuisance, offensive odors, stagnant wet areas, or create an environment for the breeding of insects.

5.6.7.4 Water Replenishment

5.6.7.4.1 Volume

Removal of water from the POOL and replacement with make-up water shall be performed as needed to maintain water quality.

5.6.7.4.2 Discharged

A volume of water totaling at least four gallons *(15 L)* per BATHER per day per AQUATIC VENUE shall be either:

1) Discharged from the system, or
2) Treated with an alternate system meeting the requirements of MAHC Section 4.7.4 and reused.

5.6.7.4.2.1 Backwash Water

The required volume of water to be discharged may include backwash water.

5.6.7.4.3 Multi-System Facilities

In multi-RECIRCULATION SYSTEM facilities, WATER REPLENISHMENT shall be proportional to the number of BATHERS in each system.

5.6.8 Solid Waste

5.6.8.1 Storage Receptacles

5.6.8.1.1 Good Repair and Clean

Outside waste and recycling containers shall be maintained in good repair and clean condition.

5.6.8.1.2 Areas

Outside waste and recycling STORAGE areas shall be maintained in good repair and clean condition.

5.6.8.2 Disposal

5.6.8.2.1 Frequency

Solid waste and recycled materials shall be removed at a frequency to prevent attracting vectors or causing odor.

5.6.8.2.2 Local Code Compliance

Solid waste and recycled materials shall be disposed of in compliance with local CODES.

5.6.9 Decks

5.6.9.1 Food Preparation and Consumption

5.6.9.1.1 Preparation

Food preparation and cooking shall only be permitted in designated areas as specified in this CODE.

5.6.9.1.2 Eating and Drinking

BATHERS shall not eat or drink while in or partially in the AQUATIC VENUE water except in designated areas.

5.6.9.1.2.1 Swim-Up Bars

Swim-up bars, when utilized, shall provide facilities for bathers to place food and drinks on a surface which can be SANITIZED.

5.6.9.2 Glass

5.6.9.2.1 Containers

Glass food and beverage containers shall be prohibited in PATRON areas of AQUATIC FACILITIES.

5.6.9.2.2 Furniture

Glass furniture shall not be used in an AQUATIC FACILITY.

5.6.9.3 Deck Maintenance

5.6.9.3.1 Free From Obstructions

The PERIMETER DECK shall be maintained free from obstructions, including PATRON seating, to preserve space required for lifesaving and rescue.

5.6.9.3.2 Diaper Changing

Diaper changing shall only be done at a designated DIAPER-CHANGING STATION.

5.6.9.3.2.1 Prohibited

Diaper changing shall be prohibited on the DECK.

5.6.9.3.3 Vermin

DECK areas shall be cleaned daily and kept free of debris, vermin, and vermin harborage.

5.6.9.3.4 Original Design

DECK surfaces shall be maintained to their original design slope and integrity.

5.6.9.3.4.1 Crack Repair

CRACKS shall be repaired when they may increase the potential for:

1) Trips or falls,
2) Lacerations, or
3) Impact the ability to properly clean and maintain the DECK area.

5.6.9.3.5 Standing Water

DECK areas shall be free from standing water.

5.6.9.3.6 Drains

DECK drains shall be cleaned and maintained to prevent blockage and pooling of water.

5.6.9.3.7 Wet Areas

Wet areas shall not have absorbent materials that cannot be removed for cleaning and DISINFECTION daily.

5.6.9.3.8 Circulation Path

Fixed equipment, loose equipment, and DECK furniture shall not intrude upon the AQUATIC VENUE CIRCULATION PATH.

5.6.10 Aquatic Facility Maintenance

All appurtenances, features, signage, safety and other equipment, and systems required by this CODE shall be provided and maintained.

5.6.10.1 Diving Boards and Platforms

5.6.10.1.1 Slip-Resistant Finish

The finish and profile of surfaces of diving boards and platforms shall be maintained to prevent slips, trips, and falls.

5.6.10.1.2 Loose Bolts and Cracked Boards

Diving boards shall be inspected daily for CRACKS and loose bolts with CRACKED boards removed and loose bolts tightened immediately.

5.6.10.2 Steps and Guardrails

5.6.10.2.1 Immovable

Steps and guardrails shall be secured so as not to move during use.

5.6.10.2.2 Maintenance

The profile and surface of steps shall be maintained to prevent slips and falls.

5.6.10.3 Starting Platforms

The profile and surface of starting platform steps shall be in good repair to prevent slips, trips, falls, and pinch hazards.

5.6.10.4 Waterslides

5.6.10.4.1 Maintenance

WATERSLIDES shall be maintained and operated to manufacturer's/designer's specifications.

5.6.10.4.2 Slime and Biofilm

Slime and biofilm layers shall be removed on all accessible WATERSLIDE surfaces.

5.6.10.4.3 Flow Rates

WATERSLIDE water flow rates shall be checked to be within designer or manufacturer's specifications prior to opening to the public.

5.6.10.4.4 Disinfectant

Where WATERSLIDE plumbing lines are susceptible to holding stagnant water, WATERSLIDE pumps shall be started with sufficient time prior to opening to flush such plumbing lines with treated water.

5.6.10.4.4.1 Water Testing

The water shall be tested to verify the disinfectant in the water is within the parameters specified in MAHC Section 5.7.3.1.1.2.

5.6.10.5 Fencing and Barriers

5.6.10.5.1 Maintenance

Required fencing, BARRIERS, and gates shall be maintained at all times.

5.6.10.5.2 Tested Daily

Gates, locks, and associated alarms, if required, shall be tested daily prior to opening.

5.6.10.6 Aquatic Facility Cleaning

5.6.10.6.1 Cleaning

The AQUATIC VENUE shall be kept clean of debris, organic materials, and slime/biofilm in accessible areas in the water and on surfaces.

5.6.10.6.2 Vacuuming

Vacuuming shall only be done when the AQUATIC VENUE is closed.

5.6.10.6.3 Port Openings

Vacuum port openings shall be covered with an approved device cover when not in use.

5.6.10.6.3.1 Damaged

POOLS with missing or damaged vacuum port openings shall be closed and repairs made before re-opening.

5.7 Recirculation and Water Treatment

5.7.1 Recirculation Systems and Equipment

5.7.1.1 General

5.7.1.1.1 Continuous Operation

All components of the filtration and RECIRCULATION SYSTEMS shall be kept in continuous operation 24 hours per day.

5.7.1.1.1.1 Reduced Flowrates

The system flowrate shall not be reduced more than 25% lower than the minimum design requirements and only reduced when the POOL is unoccupied.

5.7.1.1.1.1.1 System Design

The flow turndown system shall be designed as specified in MAHC Sections 4.7.1.10.6.1 to 4.7.1.10.6.2.

5.7.1.1.1.1.2 Water Clarity

The system flowrate shall be based on ensuring the minimum water clarity required under MAHC Section 5.7.6 is met before opening to the public.

5.7.1.1.1.1.2 Disinfectant Levels

The turndown system shall be required to maintain required DISINFECTANT and pH levels at all times.

5.7.1.1.2 Flow

Flow through the various components of a RECIRCULATION SYSTEM shall be balanced according to the provisions outlined in MAHC Section 5.7.1 to maximize the water clarity and SAFETY of a POOL.

5.7.1.1.3 Gutter / Skimmer Pools

For gutter or SKIMMER POOLS with main drains, the required recirculation flow shall be as follows during normal operation:

1) At least 80% of the flow through the perimeter overflow system, and
2) No greater than 20% through the main drain.

5.7.1.2 Combined Venue Treatment

Each individual AQUATIC VENUE in a combined treatment system shall meet required TURNOVER times specified in MAHC Section 5.7.1.9 and achieve all water quality criteria *(including, but not limited to, pH, disinfectant concentration, and water clarity/turbidity)*.

5.7.1.3 Inlets

Inlets shall be checked at least weekly for rate and direction of flow and adjusted as necessary to produce uniform circulation of water and to facilitate the maintenance of a uniform disinfectant residual throughout the pool.

5.7.1.4 Surface Skimming Devices

5.7.1.4.1 Perimeter Overflow

The PERIMETER OVERFLOW SYSTEMS shall be kept clean and free of debris that may restrict flow.

5.7.1.4.2 Automatic Fill System

The automatic fill system, when installed, shall maintain the water level at an elevation such that the gutters must overflow continuously around the perimeter of the POOL.

5.7.1.4.3 Skimmer Water Levels

The water levels shall be maintained near the middle of the SKIMMER openings.

5.7.1.4.4 Flow

The flow through each SKIMMER shall be adjusted to maintain skimming action that will remove all floating matter from the surface of the water.

5.7.1.4.5 Strainer Baskets

The strainer baskets for SKIMMERS shall be cleaned as necessary to maintain proper skimming.

5.7.1.4.6 Weirs

Weirs shall remain in place and in working condition at all times.

5.7.1.4.6.1 Broken or Missing Weirs

Broken or missing SKIMMER weirs shall be replaced immediately.

5.7.1.4.7 Flotation Test

A flotation test may be required by the AHJ to evaluate the effectiveness of surface skimming.

5.7.1.5 Submerged Drains/Suction Outlet Covers or Gratings

5.7.1.5.1 Replaced

Loose, broken, or missing suction outlet covers and sumps shall be secured or replaced immediately and installed in accordance with the manufacturer's requirements.

5.7.1.5.1.1 Closed

POOLS shall be closed until the required repairs can be completed.

5.7.1.5.1.2 Close/Open Procedures

AQUATIC FACILITIES shall follow procedures for closing and re-opening whenever required as outlined in MAHC Section 5.4.1.

5.7.1.5.2 Documentation

The manufacturer's documentation on all outlet covers and sumps shall be made part of the permanent records of the AQUATIC FACILITY.

5.7.1.6 Piping [N/A]

See Annex discussion.

5.7.1.7 Strainers & Pumps

Strainers shall be in place and cleaned as required to maintain pump performance.

5.7.1.8 Flow Meters

Flow meters in accordance with MAHC Section 4.7.1.9.1 shall be provided and maintained in proper working order.

5.7.1.9 Flow Rates / Turnovers

5.7.1.9.1 New Construction or Substantially Altered Venues

AQUATIC VENUES constructed or substantially altered after the adoption of this code shall be operated at the designed flow rate to provide the required TURNOVER RATE 24-hours per day except as allowed in MAHC Section 4.7.1.10.

5.7.1.9.2 Construction Before Adoption of this Code

AQUATIC VENUES constructed before the adoption of this code shall be operated 24 hours per day at their designed flow rate.

5.7.2 Filtration

Filters and filter media shall be listed and labeled to NSF/ANSI 50 by an ANSI-accredited certification organization. Filters shall be backwashed, cleaned and maintained according to the manufacturer's instructions.

5.7.2.1 Granular Media Filters

5.7.2.1.1 Filtration Rates

High-rate granular media filters shall be operated at no more than 15 gallons per minute per square foot *(36.7 m/h)* when a minimum bed depth of 15 inches *(38.1 cm)* is provided per manufacturer's instructions.

5.7.2.1.1.1 Less than Fifteen Inch Bed Depth

When a bed depth is less than 15 inches *(38.1 cm)*, filters shall operate at no more than 12 gallons per minute per square foot *(29.3 m/h)*.

5.7.2.1.2 Backwashing Rates

The granular media filter system shall be backwashed at a rate of at least 15 gallons per minute per square foot *(36.7 m/h)* of filter bed surface area as per MAHC Section 4.7.2.2.3.2, unless explicitly prohibited by the filter manufacturer and/or approved at an alternate rate as specified in the NSF/ANSI 50 listing.

5.7.2.1.3 Clear Water

Backwashing should be continued until the water leaving the filter is clear.

5.7.2.1.4 Backwashing Frequency

Backwashing of each filter shall be performed at a differential pressure increase over the initial clean filter pressure, as recommended by the filter manufacturer, unless the system can no longer achieve the design flow rate.

5.7.2.1.4.1 Backwash Scheduling

Backwashes shall be scheduled to take place when the AQUATIC VENUE is closed for BATHER use.

5.7.2.1.4.1.1 Backwashing with Bathers Present

A filter may be backwashed while BATHERS are in the AQUATIC VENUE if all of the following criteria are met:

1) Multiple filters are used, and
2) The filter to be backwashed can be isolated from the remaining RECIRCULATION SYSTEM and filters, and
3) The recirculation and filtration system still continues to run as per this CODE, and
4) The chemical feed lines inject at a point where chemicals enter the RECIRCULATION SYSTEM after the isolated filter and where they can mix as needed.

5.7.2.1.5 Filter Media Inspections

Sand or other granular media shall be inspected for proper depth and cleanliness at least one time per year, replacing the media when necessary to restore depth or cleanliness.

5.7.2.1.6 Vacuum Sand Filters

The manual air release valve of the filter shall be opened as necessary to remove any air that collects inside of the filter as well as following each backwash.

5.7.2.1.7 Filtration Enhancing Products

Products used to enhance filter performance shall be used according to manufacturers' recommendations.

5.7.2.2 Precoat Filters

5.7.2.2.1 Appropriate

The appropriate media type and quantity as recommended by the filter manufacturer shall be used.

5.7.2.2.1.1 Approved

The media shall be listed and labeled to NSF/ANSI 50 by an ANSI-accredited certification organization for use in the filter.

5.7.2.2.2 Return to the Pool

Precoating of the filters shall be required in closed loop *(precoat)* mode to minimize the potential for media or debris to be returned to the POOL unless filters are listed and labeled to NSF/ANSI 50 by an ANSI-accredited certification organization to return water to the POOL during the precoat process.

5.7.2.2.3 Operation

Filter operation shall be per manufacturer's instructions.

5.7.2.2.3.1 Uninterrupted Flow

Flow through the filter shall not be interrupted when switching from precoat mode to filtration mode unless the filters are listed and labeled to NSF/ANSI 50 by an ANSI-accredited certification organization to return water to the POOL during the precoat process.

5.7.2.2.3.1.1 Flow Interruption

When a flow interruption occurs on precoat filters not designed to bump, the media must be backwashed out of the filter and a new precoat established according to the manufacturer's recommendations.

5.7.2.2.3.2 Maximum Precoat Media Load

Systems designed to flow to waste while precoating shall use the maximum recommended precoat media load permitted by the filter manufacturer to account for media lost to the waste stream during precoating.

5.7.2.2.4 Cleaning

Backwashing or cleaning of filters shall be performed at a differential pressure increase over the initial clean filter pressure as recommended by the filter manufacturer unless the system can no longer achieve the design flow rate.

5.7.2.2.5 Continuous Feed Equipment

Continuous filter media feed equipment tank agitators shall run continuously.

5.7.2.2.5.1 Batch Application

Filter media feed may also be performed via batch application.

5.7.2.2.6 Bumping

Bumping a precoat filter shall be performed in accordance with the manufacturer's recommendations.

5.7.2.2.7 Filter Media

5.7.2.2.7.1 Diatomaceous Earth

Diatomaceous earth *(DE)*, when used, shall be added to precoat filters in the amount recommended by the filter manufacturer and in accordance with the specifications for the filter listing and labeling to NSF/ANSI 50 by an ANSI-accredited certification organization.

5.7.2.2.7.2 Perlite

Perlite, when used, shall be added to precoat filters in the amount recommended by the filter manufacturer and in accordance with the specifications for the filter listing and labeling to NSF/ANSI 50 by an ANSI-accredited certification organization.

5.7.2.3 Cartridge Filters

5.7.2.3.1 NSF Standards

Cartridge filters shall be operated in accordance with the filter manufacturer's recommendation and be listed and labeled to NSF/ANSI 50 by an ANSI-accredited certification organization.

5.7.2.3.2 Filtration Rates

The maximum operating filtration rate for any surface-type cartridge filter shall not:

1) Exceed the lesser of either the manufacturer's recommended filtration rate or 0.375 gallons per minute per square foot *(0.26 L/s/m^2)* or
2) Drop below the design flow rate required to achieve the turnover rate for the aquatic venue.

5.7.2.3.3 Filter Elements

Active filter cartridges shall be exchanged with clean filter cartridges at a differential pressure increase over the initial clean filter pressure as recommended by the filter manufacturer unless the system can no longer achieve the design flow rate.

5.7.2.3.3.1 Cleaning Procedure

The filter housing and filter cartridge shall be cleaned per manufacturer's recommendation.

5.7.2.3.3.1.1 No Manufacturer Procedure

If there is no established manufacturer cleaning procedure, clean per MAHC Section 5.7.2.3.3.2 and 5.7.2.3.3.3 shall be used.

5.7.2.3.3.2 Filter Housing Cleaning

The following procedures shall be implemented to clean the filter housing when no manufacturer instructions are established:

1) Drain filter housing to waste;
2) Remove the filter cartridges from the housing;
3) Clean the inside of the filter housing with a brush and mild detergent to remove biofilms and algae;
4) Rinse thoroughly; and
5) Mist the filter housing walls with CHLORINE bleach at a 1:10 dilution.

5.7.2.3.3.3 Filter Cartridge Cleaning

The following procedures shall be implemented to clean the filter cartridge when no manufacturer instructions are established.

5.7.2.3.3.3.1 Rinse Thoroughly

The cartridge shall be rinsed thoroughly with a spray nozzle.

5.7.2.3.3.3.2 Pressure Washer

A pressure washer shall not be used to clean cartridge filters.

5.7.2.3.3.3.3 Degrease

Cartridge filters shall be degreased each time they are cleaned per the procedures outlined in this section.

5.7.2.3.3.3.4 Soak

The cartridge shall be soaked overnight in one of the following solutions:

1) A cartridge filter cleaner/degreaser per instructions on product label, or
2) A solution of water with one cup of tri-sodium phosphate *(TSP)* per five gallons of water, or
3) One Cup of automatic dishwashing detergent per five gallons of water.

5.7.2.3.3.3.5 Acid

Muriatic acid or products with acid in them shall never be used prior to degreasing.

5.7.2.3.3.3.6 Rinse

The filter cartridge shall be removed from the degreaser solution and rinsed thoroughly.

5.7.2.3.3.3.7 Sanitize

The filter cartridge shall be SANITIZED by soaking for one hour in a bleach solution made by mixing one quart of household bleach per five gallons *(18.9 L)* of water.

5.7.2.3.3.3.8 Rinse

After soaking for one hour, the sanitized filter cartridge shall be removed and rinsed thoroughly.

5.7.2.3.4 Spare Cartridge

One full set of spare cartridges shall be maintained on site in a clean and dry condition.

5.7.3 Disinfection and pH Control

5.7.3.1 Primary Disinfectants

Only the primary disinfectants outlined in MAHC Section 5.7.3 shall be acceptable for use in AQUATIC VENUES.

5.7.3.1.1 Chlorine (Hypochlorites)

5.7.3.1.1.1 EPA Registered

Only CHLORINE products that are EPA-REGISTERED for use as sanitizers or disinfectants in AQUATIC VENUES or SPAS in the United States are permitted.

5.7.3.1.1.2 Minimum FAC Concentrations

Minimum FAC concentrations shall be maintained at all times in all areas as follows in MAHC Sections 5.7.3.1.1.2.1 to 5.7.3.1.1.2.3.

5.7.3.1.1.2.1 Not Using Cyanuric Acid

AQUATIC VENUES ***not*** using cyanuric acid shall maintain a minimum FAC concentration of 1.0 PPM *(MG/L)*.

5.7.3.1.1.2.2 Using Cyanuric Acid

AQUATIC VENUES using cyanuric acid shall maintain a minimum FAC concentration of 2.0 PPM *(MG/L)*.

5.7.3.1.1.2.3 Spas

SPAS shall maintain a minimum FAC concentration of 3.0 PPM *(MG/L)*.

5.7.3.1.1.3 Stagnant Water Lines

Recirculated AQUATIC FEATURE water lines susceptible to holding stagnant water shall maintain disinfectant throughout the lines as per MAHC Section 5.7.3.1.1.2.

5.7.3.1.1.4 Consistent with Label Instructions

FAC concentrations shall be consistent with label instructions.

5.7.3.1.1.5 Maximum FAC Concentrations

Maximum FAC concentrations shall not exceed 10.0 PPM *(MG/L)* at any time the AQUATIC VENUE is open to bathers.

5.7.3.1.2 Bromine

5.7.3.1.2.1 EPA Registered

Only bromine products that are EPA-REGISTERED for use as sanitizers or DISINFECTANTS in AQUATIC VENUES or SPAS in the United States shall be permitted.

Note: Bromine-based disinfectants may be applied to AQUATIC VENUES and SPAS through the addition of an organic bromine compound (1,3-Dibromo-5,5-dimethylhydantoin (DBDMH) or 1-bromo-3-chloro-5,5-dimethylhydantoin (BCDMH)).

5.7.3.1.2.2 Minimum Bromine Concentrations

Minimum bromine concentrations shall be maintained at all times in all areas as follows:

1) All AQUATIC VENUES: 3.0 PPM *(MG/L), and*
2) SPAS: 4.00 PPM *(MG/L)*.

5.7.3.1.3 Stabilizers

5.7.3.1.3.1 Cyanuric Acid (CYA)

Cyanuric acid or stabilized CHLORINE products shall ***not*** be used at the following for all new construction, SUBSTANTIAL ALTERATION, or DISINFECTION equipment replacements after the effective date of this CODE:

1) SPAS; and
2) THERAPY POOLS.

5.7.3.1.3.1.1 Replacement Times

These AQUATIC VENUES shall no longer use cyanuric acid or stabilized CHLORINE products no later than four years after adoption of this CODE.

5.7.3.1.3.2 Aquatic Venues

The cyanuric acid level at all AQUATIC VENUES shall remain at or below 100 PPM *(MG/L)*.

5.7.3.1.4 Compressed Chlorine Gas

As per MAHC Section 4.7.3.2.4.1, use of compressed CHLORINE gas shall be prohibited for new construction and after SUBSTANTIAL ALTERATION to existing AQUATIC FACILITIES.

5.7.3.1.4.1 Safety Requirements

Facilities using compressed CHLORINE gas shall provide SAFETY precautions per the following MAHC sub-sections.

5.7.3.1.4.1.1 Separate Enclosure

The chlorinators and any cylinders containing CHLORINE gas used therewith shall be housed in an ENCLOSURE separated from other EQUIPMENT ROOMS, including the swimming POOL, corridors, dressing rooms and other space with a door so installed as to prevent gas leakage and equipped with an inspection window.

5.7.3.1.4.1.2 Secured

CHLORINE cylinders shall be secured from falling.

5.7.3.1.4.1.3 Cylinders in Use

Cylinders in use shall be secured on a suitable platform scale.

5.7.3.1.4.1.4 Vent to Exterior

A separate vent opening to the exterior shall be provided.

5.7.3.1.4.1.5 Fan

An electric motor-driven fan shall take suction from near the floor level of the ENCLOSURE and discharge at a suitable point to the exterior above the ground level.

5.7.3.1.4.1.5.1 Fan Switch

The fan switch shall be able to be operated from outside of the ENCLOSURE.

5.7.3.1.4.1.6 Trained Operator

Any person who operates such chlorinating equipment shall be trained in its use.

5.7.3.1.4.1.7 Stop Use

Facilities shall stop the use of CHLORINE gas if specific SAFETY equipment and training requirements, along with local CODE considerations, cannot be met.

5.7.3.1.5 Salt Electrolytic Chlorine Generators, Brine Electrolytic Chlorine or Bromine Generators

5.7.3.1.5.1 Pool Grade Salt

Only POOL grade salt shall be used.

5.7.3.1.5.2 Maintained

The saline content of the POOL water shall be maintained in the required range specified by the manufacturer.

5.7.3.1.5.3 Cleaning

Cleaning of electrolytic plates shall be performed as recommended by the manufacturer.

5.7.3.1.5.4 Corrosion Protection

Corrosion protection systems shall be maintained in the POOL basin.

5.7.3.2 Secondary or Supplemental Treatment Systems

5.7.3.2.1 Ultraviolet Light

5.7.3.2.1.1 Operate with Recirculation System

UV systems shall only operate while the RECIRCULATION SYSTEM is operating.

5.7.3.2.1.2 3-Log Inactivation

Secondary UV systems shall be operated and maintained not to exceed the maximum validated flow rate and meet or exceed the minimum validated output intensity needed to achieve the required dose for a 3-log inactivation.

5.7.3.2.1.3 Free Available Chlorine and Bromine Levels

Use of UV does not modify any other water quality requirements.

5.7.3.2.1.4 Calibrated Sensors

UV sensors shall be calibrated at a frequency in accordance with manufacturer recommendations.

5.7.3.2.1.5 Records

Records of calibration shall be maintained by the facility.

5.7.3.2.2 Ozone

5.7.3.2.2.1 3-Log Inactivation

Ozone systems shall be operated and maintained according to the manufacturer's instructions to maintain the required design performance.

5.7.3.2.2.2 Residual Ozone Concentration

Residual ozone concentration in the AQUATIC VENUE water shall remain below 0.1 PPM (*MG/L*).

5.7.3.2.2.3 Free Available Chlorine and Bromine Levels

Use of ozone does not modify any other water quality requirements.

5.7.3.2.2.4 Standard Operating Manual

A printed STANDARD operating manual shall be provided containing information on the operation and maintenance of the ozone generating equipment, including the responsibilities of workers in an emergency.

5.7.3.2.2.5 Employees Trained

All employees shall be properly trained in the operation and maintenance of the equipment.

5.7.3.2.3 Copper / Silver Ions

5.7.3.2.3.1 EPA Registered

Only those systems that are EPA-REGISTERED for use as sanitizers or disinfectants in AQUATIC VENUES or SPAS in the United States are permitted.

5.7.3.2.3.2 Concentrations

Copper and silver concentrations shall not exceed 1.3 PPM *(MG/L)* for copper and 0.10 PPM *(MG/L)* for silver for use as disinfectants in AQUATIC VENUES and SPAS in the United States.

5.7.3.2.3.3 Free Available Chlorine and Bromine Levels

FREE AVAILABLE CHLORINE or bromine levels shall be maintained in accordance with MAHC Section 5.7.3.1.1 or 5.7.3.1.2, respectively.

5.7.3.3 Other Sanitizers, Disinfectants, or Chemicals

Other sanitizers, disinfectants, or chemicals used must:

1) Be U.S. EPA-REGISTERED under the Federal Insecticide, Fungicide, and Rodenticide Act *(FIFRA)* and,
2) Not create a hazardous condition or compromise disinfectant efficacy when used with required bromine or CHLORINE concentrations, and
3) Not interfere with water quality measures meeting all criteria set forth in this CODE.

5.7.3.3.1 Chlorine Dioxide

CHLORINE dioxide shall only be used for remediation for water quality issues when the AQUATIC VENUE is closed and BATHERS are not present.

5.7.3.3.1.1 Safety Considerations

SAFETY training and SAFETY precautions related to use of CHLORINE dioxide shall be in place.

5.7.3.3.2 Clarifiers, Flocculants, Defoamers

Clarifiers, flocculants, and defoamers shall be used per manufacturer's instructions.

5.7.3.4 pH

5.7.3.4.1 pH levels

The pH of the water shall be maintained between 7.2 and 7.8.

5.7.3.4.2 Approved Substances

Approved substances for pH adjustment shall include but not be limited to muriatic *(hydrochloric)* acid, sodium bisulfate, carbon dioxide, sulfuric acid, sodium bicarbonate, and soda ash.

5.7.3.5 Feed Equipment

5.7.3.5.1 Acceptable Chemical Delivery

Acceptable disinfectant and pH control chemicals shall be delivered through an automatic chemical feed system upon adoption of this code.

5.7.3.5.1.1 Dedicated and Labeled Components

All chemical feed system components must be dedicated to a single chemical and clearly labeled to prevent the introduction of incompatible chemicals.

5.7.3.5.1.2 Installed and Interlocked

Chemical feed system components shall be installed and interlocked so it cannot operate when the RECIRCULATION SYSTEM is in low or no flow circumstances as per MAHC Section 4.7.3.2.1.3.

5.7.3.5.1.3 Fail Proof Safety Features

Chemical feed system components shall incorporate failure-proof features so the chemicals cannot feed directly into the AQUATIC VENUE, the venue piping system not associated with the RECIRCULATION SYSTEM, source water supply system, or area within proximity of the AQUATIC VENUE DECK under any type of failure, low flow, or interruption of operation of the equipment to prevent BATHER exposure to high concentrations of AQUATIC VENUE treatment chemicals.

5.7.3.5.1.4 Maintained

All chemical feed equipment shall be maintained in good working condition.

5.7.3.5.2 Chemical Feeders

Chemical feeders shall be installed such that they are not over chemical STORAGE containers, other feeders, or electrical equipment.

5.7.3.5.3 Dry Chemical Feeders

Chemicals shall be kept dry to avoid clumping and potential feeder plugging for mechanical gate or rotating screw feeders.

5.7.3.5.3.1 Cleaned and Lubricated

The feeder mechanism shall be cleaned and lubricated to maintain a reliable feed system.

5.7.3.5.4 Venturi Inlet

Adequate pressure shall be maintained at the venturi INLET to create the vacuum needed to draw the chemical into the RECIRCULATION SYSTEM.

5.7.3.5.5 Erosion Feeders

Erosion feeders shall only have chemicals added that are approved by the manufacturer.

5.7.3.5.5.1 Opened

A feeder shall only be opened after the internal pressure is relieved by a bleed valve.

5.7.3.5.5.2 Maintained

Erosion feeders shall be maintained according the manufacturer's instructions.

5.7.3.5.6 Liquid Solution Feeders

For liquid solution feeders, spare feeder tubes *(or tubing)* shall be maintained onsite for peristaltic pumps.

5.7.3.5.7 Checked Daily

Tubing and connections shall be checked on a daily basis for leaks.

5.7.3.5.7.1 Routed

All chemical tubing that runs through areas where staff walk shall be routed in PVC piping to support the tubing and to prevent leaks.

5.7.3.5.7.1.1 Size

The double containment PVC pipe shall be of sufficient size to allow for easy replacement of tubing.

5.7.3.5.7.1.2 Turns

Any necessary turns in the piping shall be designed so as to prevent kinking of the tubing.

5.7.3.5.8 Gas Feed Systems

The Chlorine Institute requirements for safe STORAGE and use of CHLORINE gas shall be followed.

5.7.3.5.9 Carbon Dioxide

Carbon dioxide feed shall be permitted to reduce pH.

5.7.3.5.9.1 Controlled

Carbon dioxide feed shall be controlled using a gas regulator.

5.7.3.5.9.2 Alarm Monitor

CO_2/O_2 monitor and alarm shall be maintained in working condition.

5.7.3.5.9.3 Forced Ventilation

Carbon dioxide is heavier than air, so forced ventilation shall be maintained in the STORAGE room.

5.7.3.6 Testing for Water Circulation and Quality

5.7.3.6.1 Available

WATER QUALITY TESTING DEVICES *(WQTDs)* for the measurement of disinfectant residual, pH, alkalinity, CYA *(if used)*, and temperature, at a minimum, shall be available on site.

5.7.3.6.1.1 Expiration Dates

WQTDs utilizing reagents shall be checked for expiration at every use and the date recorded.

5.7.3.6.2 Store

WQTDs shall be stored in accordance with manufacturer's instructions.

5.7.3.6.3 Temperature

Chemical testing reagents shall be maintained at proper manufacturer specified temperatures.

5.7.3.6.4 Calibration

WQTDs that require calibration shall be calibrated in accordance with manufacturer's instructions and the date of calibration recorded.

5.7.3.7 Automated Controllers and Equipment Monitoring

5.7.3.7.1 Use of Controller

A automated controller capable of measuring the disinfectant residual *(FREE AVAILABLE chlorine or bromine)* or surrogate such as ORP shall be used to maintain the disinfectant residual in AQUATIC VENUES as outlined in MAHC Section 4.7.3.2.8.

5.7.3.7.1.1 Installed

An AUTOMATED CONTROLLER shall be required within one year from time of adoption of this CODE.

5.7.3.7.1.2 Interlocked

AUTOMATED CONTROLLERS shall be interlocked per MAHC Section 4.7.3.2.1.3 upon adoption of this code if existing or upon installation if not existing.

5.7.3.7.2 Sampling

The sample line for all probes shall be upstream from all primary, secondary, and supplemental DISINFECTION injection ports or devices.

5.7.3.7.3 Monitor

AUTOMATED CONTROLLERS shall be monitored at the start of the operating day to ensure proper functioning.

5.7.3.7.3.1 In Person

AUTOMATED CONTROLLERS shall be monitored in person by visual observation.

5.7.3.7.4 Activities

MONITORING shall include activities recommended by manufacturers, including but not limited to alerts and leaks.

5.7.3.7.5 Replacement Parts

Only manufacturer-approved OEM replacement parts shall be used.

5.7.3.7.6 Calibration

AUTOMATED CONTROLLERS shall be calibrated per manufacturer directions.

5.7.3.7.7 Ozone System

When an ozone system is utilized as a SECONDARY DISINFECTION SYSTEM, the system shall be monitored and data recorded at a frequency consistent with MAHC Table 5.7.3.7.7.

Table 5.7.3.7.7: Ozone System Monitoring Frequency

Parameter	Monitoring Frequency	Recording Frequency
**ORP**	Continuous	Every 4 hours
**Control System Indicating O_3 Being Created**	Continuous	Every 4 Hours
**Operational Indicators in Range**	Continuous	Every 4 hours
**O_3 Within 6 inches of Aquatic Venue Water Surface**	Annual	Annual

5.7.3.7.7.1 Other Testing

At the time the ozone generating equipment is installed, again after 24 hours of operation, and annually thereafter, the air space within six inches of the AQUATIC VENUE water shall be tested to determine compliance of less than 0.1 PPM *(mg/L)* gaseous ozone.

5.7.3.7.7.1.1 Results

Results of the test shall be maintained on site for review by the AHJ.

5.7.3.7.8 UV Systems

When a UV system is utilized for secondary disinfection, the system shall be monitored and data recorded at a frequency consistent with MAHC Table 5.7.3.7.8.

Table 5.7.3.7.8: UV System Monitoring and Calibration Frequency

Parameter	*Monitoring Frequency*	*Recording Frequency*
**Flow Rate**	Continuous	Every 4 Hours
**Intensity**	Continuous	Every 4 Hours
**Water Temperature** *(MP Medium Pressure)*	Continuous	Daily
**Set Point for Intensity**	Continuous	Daily
**UV Lamp On/Off Cycles**	Continuous	Weekly *(Total Cycles/Week)*
**Iron, Calcium Hardness**	Weekly *(If Fouling is Prevalent)*	Weekly
**UVT** *(UV Transmittance)* **Analyzer Calibration**	Weekly	Weekly
**Calibration of Intensity**	Annual	At Time of Calibration
**Calibration of Flow Meter**	Per Manufacturer's Requirements	At Time of Calibration

5.7.3.7.9 UV Alarm Testing and Maintenance

The automated UV shut-down alarm required in MAHC Section 4.7.3.3.3.7 shall be tested weekly and maintained as needed.

5.7.4 Water Sample Collection and Testing

5.7.4.1 Sample Collection

The QUALIFIED OPERATOR shall ensure a water sample is acquired for testing from the in-line sample port when available as per MAHC Section 5.7.5.

5.7.4.1.1 Same Volume

If an AQUATIC VENUE has more than one RECIRCULATION SYSTEM, the same sample volume shall be collected from each in-line sample port and tested separately.

5.7.4.1.2 No Port

If no in-line sample port is available, the QUALIFIED OPERATOR shall ensure water samples from the AQUATIC VENUE are acquired according to MAHC Section 5.7.4.3.

5.7.4.2 Routine Samples

If routine samples are collected from in-line sample ports, the QUALIFIED OPERATOR shall also ensure water samples are acquired from the bulk water of the AQUATIC VENUE at least once per day.

5.7.4.2.1 Midday Collection

Daily bulk water samples shall be collected in the middle of the AQUATIC VENUE operational day, according to the procedures in MAHC Section 5.7.4.3.

5.7.4.2.2 Compared

Water quality data from these AQUATIC VENUE samples shall be compared to data obtained from in-line port samples to assess potential water quality variability in the AQUATIC VENUE.

5.7.4.3 Bulk Water Sample

The QUALIFIED OPERATOR shall ensure the following procedure is used for acquiring a water sample from bulk water of the POOL.

5.7.4.3.1 Obtain Sample

All samples shall be obtained from a location with the following qualities:

1) At least 18 inches *(45.7 cm)* below the surface of the water, and
2) A water depth of between three to four feet *(91.4 cm to 1.2 m)* when available, and
3) A location between water inlets.

5.7.4.3.2 Rotate

Sampling locations shall rotate around the shallow end of the POOL.

5.7.4.3.3 Deepest Area

The QUALIFIED OPERATOR shall ensure a sample includes a deep end sample from the AQUATIC VENUE in the water sampling rotation once per week.

5.7.4.4 Aquatic Venue Water Chemical Balance

5.7.4.4.1 Total Alkalinity Levels

Total alkalinity shall be maintained in the range of 60 to 180 PPM *(mg/L)*.

5.7.4.4.2 Combined Chlorine (Chloramines)

The owner shall ensure the AQUATIC facility takes action to reduce the level of combined chlorine (chloramines) in the water when the level exceeds 0.4 PPM(mg/L). Such actions may include but are not limited to:

1) Superchlorination;
2) Water exchange; or
3) Patron adherence to appropriate BATHER hygiene practices.

5.7.4.4.3 Calcium Hardness

Calcium hardness shall not exceed 1000 PPM *(MG/L)*.

5.7.4.4.4 Algaecides

Algaecides may be used in an AQUATIC VENUE provided:

1) The product is labeled as an algaecide for AQUATIC VENUE or SPA use;
2) The product is used in strict compliance with label instructions; and,
3) The product is registered with the US EPA and applicable state agency.

5.7.4.5 Source (Fill) Water

The owner of a public AQUATIC VENUE, public SPA, or special use AQUATIC VENUE shall ensure that the water supply for the facility meets one of the following requirements:

1) The water comes from a public water system as defined by the applicable rules of the AHJ in which the facility is located; or
2) The water meets the requirements of the local AHJ for public water systems; or
3) The AHJ has approved an alternative water source for use in the AQUATIC FACILITY.

5.7.4.6 Water Balance for Aquatic Venues

AQUATIC VENUE water shall be chemically balanced.

5.7.4.7 Water Temperature

5.7.4.7.1 Minimize Risk and Protect Safety

Water temperatures shall be considered and planned for based on risk, SAFETY, priority facility usage, and age of participants, while managing water quality concerns.

5.7.4.7.2 Maximum Temperature

The maximum temperature for an AQUATIC VENUE is 104º F *(40°C)*.

5.7.5 Water Quality Chemical Testing Frequency

5.7.5.1 Chemical Levels

FREE AVAILABLE CHLORINE *(FAC)*, combined available CHLORINE *(CAC)*, or total bromine *(TB)*, and pH shall be tested at all AQUATIC VENUES prior to opening each day.

5.7.5.2 Manual Disinfectant Feed System

For all AQUATIC VENUES using a manual DISINFECTANT feed system that delivers DISINFECTANT via a flow through erosion feeder or metering pump without an automated controller, FREE AVAILABLE CHLORINE or bromine and pH shall be tested prior to opening to the public and every two hours while open to the public.

5.7.5.3 Automatic Disinfectant Feed System

For all AQUATIC VENUES using an automated disinfectant feed system, FAC *(or TB)* and pH shall be tested prior to opening and every four hours while open to the public.

5.7.5.4 In-Line ORP Readings

In-line ORP readings, if such systems are installed, shall be recorded at the same time the FAC *(or TB)* and pH tests are performed.

5.7.5.5 Total Alkalinity

Total Alkalinity *(TA)* shall be tested weekly at all AQUATIC VENUES.

5.7.5.6 Calcium Hardness

Calcium hardness shall be tested monthly at all AQUATIC VENUES.

5.7.5.7 Cyanuric Acid

Cyanuric acid shall be tested monthly at all AQUATIC VENUES utilizing cyanuric acid.

5.7.5.8 Saturation Index

The SATURATION INDEX shall be checked monthly.

5.7.5.8.1 Tested

Cyanuric acid shall be tested 24 hours after the addition of cyanuric acid to the AQUATIC VENUE.

5.7.5.8.2 Stabilized Chlorine

If AQUATIC VENUES utilize stabilized CHLORINE as its primary disinfectant, the operator shall test cyanuric acid every two weeks.

5.7.5.9 Total Dissolved Solids

Total dissolved solids *(TDS)* shall be tested quarterly at all AQUATIC VENUES.

5.7.5.10 Water Temperature

For heated AQUATIC VENUES, water temperature shall be recorded at the same time the FAC *(or TB)* and pH tests are performed.

5.7.5.11 Salt

If in-line electrolytic chlorinators are used, salt levels shall be tested at least weekly or per manufacturer's instructions.

5.7.5.12 Copper/Silver Systems

Copper and silver shall be tested daily at all AQUATIC VENUES utilizing copper/silver systems as a supplemental treatment system.

5.7.6 Water Clarity

5.7.6.1 Water Clarity

The water in an AQUATIC VENUE shall be sufficiently clear such that the bottom is visible while the water is static at all times the AQUATIC VENUE is open or available for use .

5.7.6.1.1 Observation

To make this observation, a four inch by four inch square *(10.2 cm X 10.2 cm)* marker tile in a contrasting color to the POOL floor or main suction outlet shall be located at the deepest part of the POOL.

5.7.6.1.2 Pools Over Ten Feet Deep

For POOLS over ten feet *(3.0 m)* deep, an eight inch by eight inch square *(20.3 X 20.3 cm)* marker tile in a contrasting color to the POOL floor or main suction outlet shall be located at the deepest part of the POOL.

5.7.6.1.3 No Marker Tile

In the absence of a marker tile or suction outlet, an alternate means of achieving the goal of observing the bottom of the POOL may be permitted.

5.7.6.2 Visible

This reference point shall be visible at all times at any point on the DECK up to 30 feet *(9.1 m)* away in a direct line of sight from the tile or main drain.

5.7.6.2.1 Spas

For SPAS, this test shall be performed when the water is in a non-turbulent state and bubbles have been allowed to dissipate.

5.8 Decks and Equipment

5.8.1 Spectator Areas

5.8.1.1 Cross-Connection Control

5.8.1.1.1 Deck Drains

Cross connection devices shall be in good working order, and shall be tested as required by the AHJ.

5.8.1.2 Materials / Slip Resistance

5.8.1.2.1 Clean and Good Repair

Surfaces shall be clean and in good repair.

5.8.1.2.2 Risk Management

The finish and profile of DECK surfaces shall be maintained to prevent slips and falls.

5.8.1.2.3 Tripping Hazards

Tripping hazards shall be avoided.

5.8.1.2.3.1 Protect

If tripping hazards are present, they shall be repaired or promptly barricaded to protect PATRONS/employees.

5.8.1.3 Deck Size/Width

The perimeter deck shall be maintained clear of obstructions for at least a four foot *(1.2 m)* width around the entire pool unless otherwise allowed by this code.

5.8.2 Diving Boards and Platforms [N/A]

5.8.3 Starting Blocks

5.8.3.1 Competitive Training and Competition

Starting platforms shall only be used for competitive swimming and training.

5.8.3.1.1 Supervision

Starting platforms shall only be used under the direct supervision of a coach or instructor.

5.8.3.1.2 Removed or Restricted

Starting platforms shall be removed, if possible, or prohibited from use during all recreational or non-competitive swimming activity by covering platforms with a manufacturer-supplied platform cover or with another means or device that is readily visible and clearly prohibits use.

5.8.4 Pool Slides [N/A]

5.8.5 Lifeguard-and Safety Related Equipment

5.8.5.1 Equipment Inspection and Maintenance

AQUATIC FACILITIES shall not be open to users unless the equipment listed under MAHC Section 5.8.5 is present and in a safe and working condition.

5.8.5.2 Safety Equipment Required at All Aquatic Facilities

5.8.5.2.1 Emergency Communication Equipment

5.8.5.2.1.1 Functioning Communication Equipment

The AQUATIC FACILITY shall have equipment for staff to communicate in cases of emergency.

5.8.5.2.1.2 Hard-Wired Telephone for 911 Call

The AQUATIC FACILITY or each AQUATIC VENUE, as necessary, shall have a functional telephone or other communication system or device that is hard wired and capable of directly dialing 911 or function as the emergency notification system.

5.8.5.2.1.3 Conspicuous and Easily Accessible

The telephone or communication system or device shall be conspicuously provided and accessible to AQUATIC VENUE users such that it can be reached immediately.

5.8.5.2.1.4 Alternate Communication Systems

Alternate functional systems, devices, or communication processes are allowed with AHJ approval in situations when a hardwired telephone is not logistically sound, and an alternate means of communication is available.

5.8.5.2.2 First Aid Equipment

5.8.5.2.2.1 Location for First Aid

The AQUATIC FACILITY shall have designated locations for emergency and first aid equipment.

5.8.5.2.2.2 First Aid Supplies

An adequate supply of first aid supplies shall be continuously stocked and include, at a minimum, as follows:

1) A First Aid Guide,
2) Absorbent compress,
3) Adhesive bandages,
4) Adhesive tape,
5) Sterile pads,
6) Disposable gloves,
7) Scissors,
8) Elastic wrap,
9) Emergency blanket,
10) Resuscitation mask with one-way valve, and
11) Blood borne pathogen spill kit.

5.8.5.2.3 Signage

5.8.5.2.3.1 Sign Indicating First Aid Location

Signage shall be provided at the AQUATIC FACILITY or each AQUATIC VENUE, as necessary, which clearly identifies the following:

1) First aid location*(s)*, and
2) Emergency telephone*(s)* or approved communication system or device.

5.8.5.2.3.2 Emergency Dialing Instructions

A permanent sign providing emergency dialing directions and the AQUATIC FACILITY address shall be posted and maintained at the emergency telephone, system or device.

5.8.5.2.3.3 Management Contact Info

A permanent sign shall be conspicuously posted and maintained displaying contact information for emergency personnel and AQUATIC FACILITY management.

5.8.5.2.3.4 Hours of Operation

A sign shall be posted stating the following:

1) The operating hours of the AQUATIC FACILITY, and
2) Unauthorized use of the AQUATIC FACILITY outside of these hours is prohibited.

5.8.5.3 Safety Equipment Required at Facilities with Lifeguards

5.8.5.3.1 UV Protection for Chairs and Stands

When a chair or stand is provided and QUALIFIED LIFEGUARDS can be exposed to ultraviolet radiation, the chair or stand shall be equipped with or in a location with protection from such ultraviolet radiation exposure.

5.8.5.3.2 Spinal Injury Board

At least one spinal injury board constructed of material easily SANITIZED/disinfected shall be provided.

5.8.5.3.2.1 Spinal Injury Board Components

The board shall be equipped with a head immobilizer and sufficient straps to immobilize a person to the spinal injury board.

5.8.5.3.3 Rescue Tube Immediately Available

Each QUALIFIED LIFEGUARD conducting PATRON surveillance with the responsibility of in-water rescue in less than three feet *(0.9 m)* of water shall have a rescue tube immediately available for use.

5.8.5.3.4 Rescue Tube on Person

Each QUALIFIED LIFEGUARD conducting PATRON surveillance in a water depth of three feet *(0.9 m)* or greater shall have a rescue tube on his/her person in a rescue ready position.

5.8.5.3.5 Identifying Uniform

QUALIFIED LIFEGUARDS shall wear attire that readily identifies them as members of the AQUATIC FACILITY'S lifeguard staff.

5.8.5.3.6 Signal Device

A whistle or other signaling device shall be worn by each QUALIFIED LIFEGUARD conducting PATRON surveillance for communicating to users and/or staff.

5.8.5.3.7 Sun Blocking Methods

All AQUATIC FACILITIES where QUALIFIED LIFEGUARDS can be exposed to ultraviolet *(UV)* radiation shall train lifeguards about the use of protective clothing, hats, sun-blocking umbrellas, and sunscreen application and re-application using or exceeding SPF Level 15 to protect exposed skin areas.

5.8.5.3.7.1 Lifeguards Responsible

QUALIFIED LIFEGUARDS are responsible for protecting themselves from UV radiation exposure and wearing appropriate sunglasses and sunscreen.

5.8.5.3.8 Polarized Sunglasses

When glare impacts the ability to see below the water's surface, QUALIFIED LIFEGUARDS shall wear polarized sunglasses while conducting BATHER surveillance.

5.8.5.3.9 Personal Protective Equipment

Personal protective devices including a resuscitation mask with one-way valve and non-latex one-use disposable gloves shall be immediately available to all QUALIFIED LIFEGUARDS.

5.8.5.3.10 Rescue Throwing Device

AQUATIC FACILITIES with one QUALIFIED LIFEGUARD shall provide and maintain a U.S. Coast Guard-approved aquatic rescue throwing device as per the specifications of MAHC Section 5.8.5.4.1.

5.8.5.3.11 Reaching Pole

AQUATIC FACILITIES with one QUALIFIED LIFEGUARD shall provide and maintain a reaching pole as per the specifications of MAHC Section 5.8.5.4.2.

5.8.5.4 Safety Equipment and Signage Required at Facilities without Lifeguards

5.8.5.4.1 Throwing Device

AQUATIC VENUES whose depth exceeds two feet *(61.0 cm)* of standing water shall provide and maintain a U.S. Coast Guard-approved aquatic rescue throwing device, with at least a quarter-inch *(6.3 mm)* thick rope whose length is 50 feet *(15.2 m)* or 1.5 times the width of the POOL, whichever is less.

5.8.5.4.1.1 Throwing Device Location

The rescue throwing device shall be located in the immediate vicinity to the AQUATIC VENUE and be accessible to BATHERS.

5.8.5.4.2 Reaching Pole

AQUATIC VENUES whose depth exceeds two feet *(61 cm)* of standing water shall provide and maintain a reaching pole of 12 foot *(3.7 m)* to 16 foot *(4.9 m)* in length, non-telescopic, light in weight, and with a securely attached Shepherd's Crook with an aperture of at least 18 inches *(45.7 cm)*.

5.8.5.4.2.1 Reaching Pole Location

The reaching pole shall be located in the immediate vicinity to the AQUATIC VENUE and be accessible to BATHERS and PATRONS.

5.8.5.4.2.2 Non-Conductive Material

Reaching poles provided by the AQUATIC FACILITY after the adoption date of this code shall be of non-conductive material.

5.8.5.4.3 CPR Posters

Cardiopulmonary Resuscitation *(CPR)* posters that are up to date with latest CPR programs and protocols shall be posted conspicuously at all times.

5.8.5.4.4 Imminent Hazard Sign

A sign shall be posted outlining the IMMINENT HEALTH HAZARDS, which require AQUATIC VENUE or AQUATIC FACILITY closure as defined in this CODE per MAHC 6.6.3.1 and a telephone number to report problems to the owner/operator.

5.8.5.4.5 *Additional Signage*

For any AQUATIC VENUE with standing water, a sign shall be posted signifying a QUALIFIED LIFEGUARD is not on duty and that the following rules apply:

1) Persons under the age of 14 cannot be in the AQUATIC VENUE without direct adult supervision meaning children shall be in adult view at all times, and
2) Youth and childcare groups, training, lifeguard courses, and swim lessons are not allowed without a QUALIFIED LIFEGUARD providing PATRON surveillance.

5.8.6 Barriers and Enclosures

5.8.6.1 General Requirements

All required BARRIERS and ENCLOSURES shall be maintained to prevent unauthorized entry to the protected space.

5.8.6.2 Construction Requirements (N/A)

5.8.6.3 Gates and Doors

5.8.6.3.1 *Self-Closing and Latching*

All primary public access gates or doors serving as part of an ENCLOSURE shall have functional self-closing and self-latching closures.

5.8.6.3.1.1 Exception

Gates or doors used solely for after-hours maintenance shall remain locked at all times when not in use by staff.

5.8.6.3.1.2 Propping Open

Required self-closing and self-latching gates or doors serving as part of a guarded ENCLOSURE may be maintained in the open position when the AQUATIC VENUE is open and staffed as required.

5.9 Filter/Equipment Room

5.9.1 Chemical Storage

5.9.1.1 Local Codes

Chemical STORAGE shall be in compliance with local building and fire CODES.

5.9.1.2 OSHA and EPA

Chemical handling shall be in compliance with OSHA and EPA regulations.

5.9.1.3 Safety Data Sheets

For each chemical, STORAGE, handling and use of the chemical shall be in compliance with the manufacturer's Safety Data Sheets *(SDS)* and labels.

5.9.1.4 Access Prevention

AQUATIC VENUE chemicals shall be stored to prevent access by unauthorized individuals.

5.9.1.5 Protected

AQUATIC VENUE chemicals shall be stored so that they are protected from getting wet.

5.9.1.6 No Mixing

AQUATIC VENUE chemicals shall be stored so that if the packages were to leak, no mixing of incompatible materials would occur.

5.9.1.6.1 SDS Consulted

Safety Data Sheets *(SDS)* shall be consulted for incompatibilities.

5.9.1.7 Ignition Sources

Possible ignition sources , including but not limited to gasoline, diesel, natural gas, or gas-powered equipment such as lawn mowers, motors, grills, POOL heaters, or portable stoves shall not be stored or installed in the CHEMICAL STORAGE SPACE.

5.9.1.8 Smoking

Smoking shall be prohibited in the CHEMICAL STORAGE SPACE.

5.9.1.9 Lighting

Lighting shall be at minimum 30 footcandles *(323 lux)* to allow operators to read labels on containers throughout the CHEMICAL STORAGE SPACE and pump room.

5.9.1.10 PPE

Personal Protective Equipment *(PPE)* shall be available as indicated on the chemical SDSs.

5.9.1.11 Storage

Chemicals shall be stored away from direct sunlight, temperature extremes, and high humidity.

5.9.1.12 Single Container

A single container of a particular chemical that has been opened and that is currently in use in the pump room may be kept in a staging area of the pump room only if the chemical*(s)* will be protected from exposure to heat and moisture.

5.9.1.13 Separate

The CHEMICAL STORAGE SPACE shall be separate from the EQUIPMENT ROOM.

5.9.1.13.1 Waiver

For AQUATIC FACILITIES that do not currently have a CHEMICAL STORAGE SPACE separate from the EQUIPMENT ROOM, this requirement may be waived at the discretion of the local public health and/or fire officials if the chemicals are protected from exposure to heat and moisture and no imminent health or SAFETY threats are identified.

5.9.1.14 Warning Signs

Warning signs in compliance with NFPA or HMIS ratings shall be posted on CHEMICAL STORAGE SPACE doors.

5.9.2 Chemical Handling

5.9.2.1 Identity

Containers of chemicals shall be labeled, tagged, or marked with the identity of the material and a statement of the hazardous effects of the chemical according to OSHA and/or EPA materials labeling requirements.

5.9.2.1.1 Labeling

All AQUATIC VENUE chemical containers shall be labeled according to OSHA and/or EPA materials labeling requirements.

5.9.2.2 NSF Standard

The chemical equipment used in controlling the quality of water shall be listed and labeled to NSF/ANSI 50 by an ANSI-accredited certification organization and used only in accordance with the manufacturer's instructions.

5.9.2.3 Measuring Devices

Chemicals shall be measured using a dedicated measuring device where applicable.

5.9.2.3.1 Clean and Dry

These measuring devices shall be clean, dry, and constructed of material compatible with the chemical to be measured to prevent the introduction of incompatible chemicals.

5.9.2.4 Chemical Addition Methods

5.9.2.4.1 Automatically Introduced

DISINFECTION and pH control chemicals shall be automatically introduced through the RECIRCULATION SYSTEM.

5.9.2.4.1.1 Manual Addition

SUPERCHLORINATION or shock chemicals and other POOL chemicals other than DISINFECTION and pH control may be added manually to the POOL.

5.9.2.4.1.2 Absence of Bathers

Chemicals added manually directly into the AQUATIC VENUE shall only be introduced in the absence of BATHERS.

5.9.2.4.2 Safety Requirements

Whenever required by the manufacturer, chemicals shall be diluted *(or mixed with water)* prior to application and as per the manufacturer's directions.

5.9.2.4.2.1 Added

Chemicals shall be added to water when diluting as opposed to adding water to a concentrated chemical.

5.9.2.4.2.2 Mixed

Each chemical shall be mixed in a separate, labeled container.

5.9.2.4.2.2.1 Never Mixed Together

Two or more chemicals shall never be mixed in the same dilution water.

5.10 Hygiene Facilities

5.10.1 General [N/A]

5.10.2 Location [N/A]

5.10.3 Bathhouse Design [N/A]

5.10.4 Plumbing Fixture Requirements

5.10.4.1 General Requirements

5.10.4.1.1 Cleaned and Sanitized

HYGIENE FACILITY fixtures, dressing area fixtures, and furniture shall be cleaned and SANITIZED daily and more often if necessary with an EPA-REGISTERED product and more often if necessary to provide a clean and sanitary environment.

5.10.4.1.2 Mold and Mildew

HYGIENE FACILITY floors, walls, and ceilings shall be kept clean and free of visible mold and mildew.

5.10.4.1.3 Hand Wash Station

HAND WASH STATIONS shall include the following items:

1) Hand wash sink,
2) Adjacent soap with dispenser,
3) Hand drying device or paper towels and dispenser, and
4) Trash receptacle.

5.10.4.2 Cleansing Showers

5.10.4.2.1 Cleaned and Sanitized

CLEANSING SHOWERS shall be cleaned and SANITIZED daily and more often if necessary with an EPA-REGISTERED product and more often if necessary to provide a clean and sanitary environment.

5.10.4.3 Rinse Showers

5.10.4.3.1 Cleaned

RINSE SHOWERS shall be cleaned daily and more often if necessary with an EPA-REGISTERED product and more often if necessary to provide a clean and sanitary environment.

5.10.4.3.2 Easy Access

RINSE SHOWERS shall be easily accessible.

5.10.4.3.3 Not Blocked

Equipment and furniture on the DECK shall not block access to RINSE SHOWERS.

5.10.4.3.4 No Soap

Soap dispensers and soap shall be prohibited at RINSE SHOWERS.

5.10.4.4 All Showers [N/A]

5.10.4.5 Diaper-Changing Stations

Diaper-changing stations are required in all AQUATIC FACILITIES upon adoption of this code per MAHC 4.10.4.5.1.

5.10.4.5.1 Hand Wash Sink Installed and Operational

The adjacent hand wash sink shall be installed and operational within ***one*** year from the date of the AHJ's adoption of the MAHC.

5.10.4.5.2 Cleaned

DIAPER-CHANGING STATIONS shall be cleaned and disinfected daily and more often if necessary to provide a clean and sanitary environment.

5.10.4.5.2.1 Maintained

They shall be maintained in good condition and free of visible contamination.

5.10.4.5.3 Disinfectant

EPA-REGISTERED disinfectant shall be provided in the form of either of the following:

1) A solution in a spray dispenser with paper towels and dispenser, or
2) Wipes contained within a dispenser.

5.10.4.5.3.1 Covers

If disposable DIAPER-CHANGING UNIT covers are provided in addition to disinfectant, they shall cover the DIAPER-CHANGING UNIT surface during use and keep the unit in clean condition.

5.10.4.5.4 Portable Hand Wash Station

If a portable HAND WASH STATION is provided for use it shall be operational and maintained in good condition at all times.

5.10.4.6 Non-Plumbing Fixture Requirements

5.10.4.6.1 Paper Towels

If paper towels are used for hand drying, a dispenser and paper towels shall be provided for use at HAND WASH STATIONS.

5.10.4.6.2 Soap

Soap dispensers shall be provided at HAND WASH STATIONS and CLEANSING SHOWERS and shall be kept full of liquid or granular soap.

5.10.4.6.2.1 Bar Soap

Bar soap shall be prohibited.

5.10.4.6.3 Trash

A minimum of one hands-free trash receptacle shall be provided in areas adjacent to hand washing sinks.

5.10.4.6.3.1 Trash Emptying

Trash receptacles shall be emptied daily and more often if necessary to provide a clean and sanitary environment.

5.10.4.6.4 Floor Coverings

Non-permanent floor coverings *(including but not limited to mats and racks)* shall be removable and maintained in accordance with MAHC Section 5.10.4.1.1.

5.10.4.6.4.1 Wood

Wooden racks, duckboards, and wooden mats shall be prohibited on HYGIENE FACILITY and dressing area flooring.

5.10.4.7 Sharps

5.10.4.7.1 Biohazard Action Plan

A biohazard action plan shall also be on file as required by local, state or federal regulations and as part of the AQUATIC FACILITY SAFETY PLAN.

5.10.4.7.2 Disposed

Sharps within approved containers shall be disposed of as needed by the AQUATIC FACILITY in accordance with local, state, or federal regulations.

5.10.5 Provision of Suits, Towels, and Shared Equipment

5.10.5.1 Towels

All towels provided by the AQUATIC FACILITY shall be washed with detergent in warm water, rinsed, and thoroughly dried at the warmest temperature listed on the fabric label after each use.

5.10.5.1.1 Suits

Any attire provided by the AQUATIC FACILITY shall be washed in accordance with the fabric label or manufacturer's instructions.

5.10.5.2 Receptacles

Non-absorbent, easily cleanable receptacles shall be provided for collection of used suits and towels.

5.10.5.3 Shared Equipment Cleaned and Sanitized

Equipment provided by the AQUATIC FACILITY that comes into contact with BATHER's eyes, nose, ears, and mouth *(including but not limited to snorkels, nose clips, and goggles)* shall be cleaned, SANITIZED between uses, and stored in a manner to prevent biological growth.

5.10.5.4 Other Equipment

Other shared equipment provided by the AQUATIC FACILITY, including but not limited to fins, kickboards, tubes, lifejackets, and noodles, shall be kept clean and stored in a manner to prevent mold and other biological growth.

5.10.5.5 Good Repair

Shared equipment shall be maintained in good repair.

5.10.5.6 Used Equipment

Used and un-SANITIZED shared equipment shall be kept separate from cleaned and SANITIZED shared equipment.

5.10.5.6.1 Receptacles

Non-absorbent, easily cleanable receptacles shall be provided for collection of used shared equipment.

5.11 Water Supply / Wastewater Disposal [N/A]

5.12 Special Requirements for Specific Venues

5.12.1 Waterslides

5.12.1.1 Signage

Warning signs shall be posted in accordance with manufacturer's recommendations.

5.12.2 Wave Pools

5.12.2.1 Life Jackets

U.S. Coast Guard-approved life jackets that are properly sized and fitted shall be provided free for use by BATHERS who request them.

5.12.3 Moveable Floors

5.12.3.1 Starting Platforms

The use of starting platforms in the area of a MOVEABLE FLOOR shall be prohibited when the water depth is shallower than the minimum required water depth of four feet *(1.2 m)*. Use may only occur as per MAHC Section 5.6.10.3.

5.12.3.2 Diving Boards

When a MOVEABLE FLOOR is installed into a DIVING POOL, diving shall be prohibited unless the DIVING POOL depth meets criteria set in MAHC Section 4.8.2.1.1.

5.12.4 Bulkheads

5.12.4.1 Open Area

If a BULKHEAD is operated with an open area underneath, no one shall be allowed to swim beneath the BULKHEAD.

5.12.4.2 Bulkhead Travel

The BULKHEAD position shall be maintained such that it cannot encroach on any required clearances of other features such as diving boards.

5.12.5 Interactive Water Play Aquatic Venues

5.12.5.1 Cracks

CRACKS in the INTERACTIVE WATER PLAY AQUATIC VENUE shall be repaired when they may be a potential for leakage, present a tripping hazard, a potential cause of lacerations, or impact the ability to properly clean and maintain the INTERACTIVE WATER PLAY AQUATIC VENUE area.

5.12.5.2 Cleaning

When cleaning the INTERACTIVE WATER PLAY AQUATIC VENUE CONTAMINANTS shall be removed or washed to the sanitary sewer.

5.12.5.2.1 No Sanitary Sewer Drain Available

If no sanitary sewer drain is available then debris shall be washed/rinsed to the nearest DECK drain or removed in a manner that prevents CONTAMINANTS from reentering the INTERACTIVE WATER PLAY AQUATIC VENUE.

5.12.6 Wading Pools

5.12.7 Spas

5.12.7.1 Required Operation Time

SPA filtration systems shall be operated 24 hours per day except for periods of draining, filling, and maintenance.

5.12.7.2 Drainage and Replacement

SPAS shall be drained, cleaned, scrubbed, and water replaced as calculated in MAHC section 5.12.7.2.1.

5.12.7.2.1 Calculated

The water replacement interval *(in days)* shall be calculated by dividing the SPA volume *(in gallons)* by three and then dividing by the average number of users per day.

5.12.7.3 Scrubbed

SPA surfaces, including interior of SKIMMERS, shall be scrubbed or wiped down, and all water drained prior to refill.

The Model Aquatic Health Code

The Code

POLICIES AND MANAGEMENT

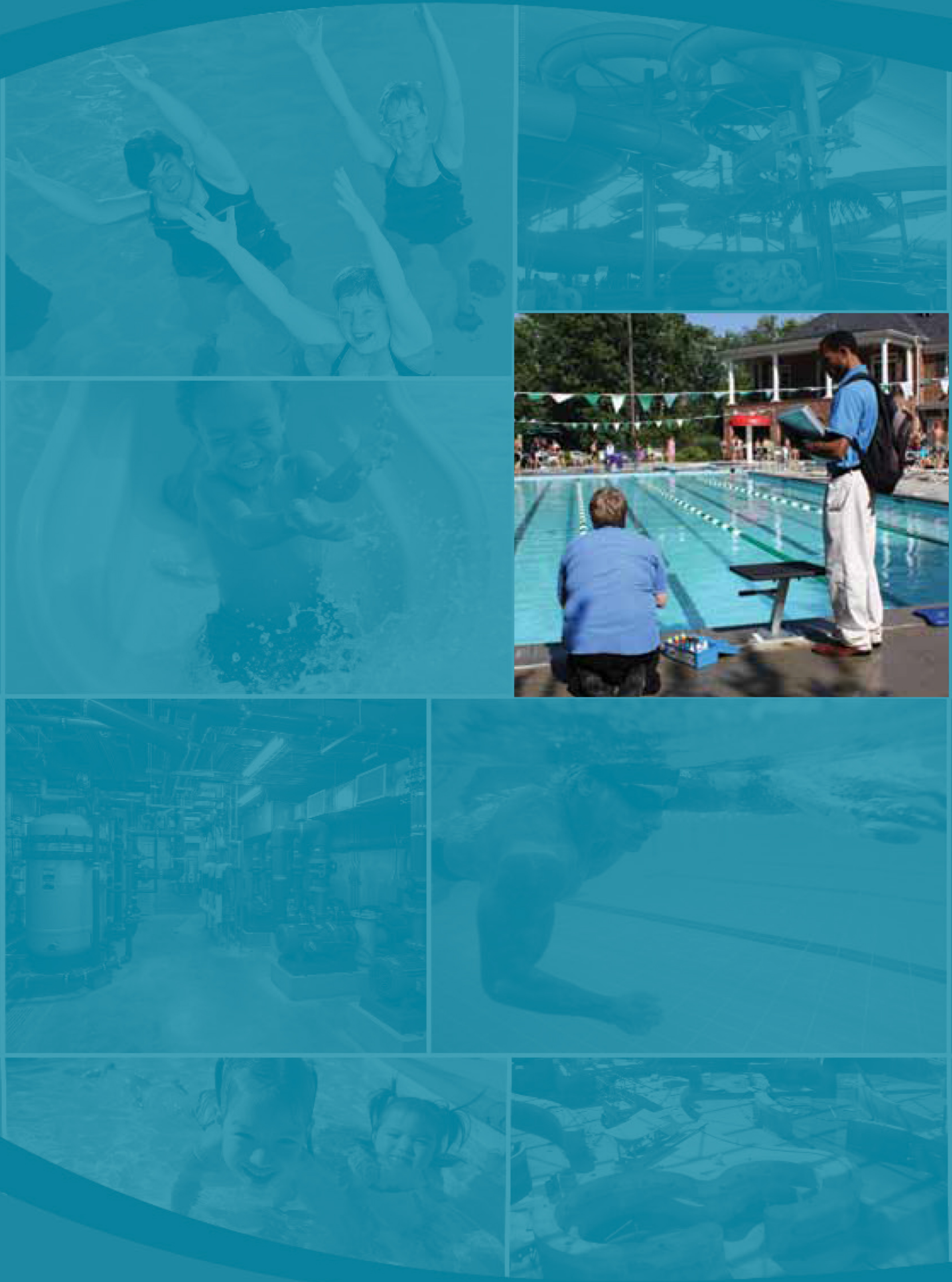

CS249779

6.0 Policies and Management

The provisions of Chapter 6 shall apply to all AQUATIC FACILITIES covered by this CODE regardless of when constructed, unless otherwise noted.

6.0.1 Staff Training

All QUALIFIED OPERATORS, maintenance staff, QUALIFIED LIFEGUARD staff, or any others who are involved in the STORAGE, use, or handling of chemicals shall receive training prior to access of chemicals, and receive at least an annual review of procedures thereafter for the following topics discussed in MAHC Section 6.0.1.1 to 6.0.1.5.

6.0.1.1 Storage and Handling

Procedures for chemical STORAGE and handling outlined in this CODE.

6.0.1.2 PPE Procedures

Standard precautions, PPE, and other measures to minimize exposure to chemicals as required by OSHA. This shall include staff training in PPE and respiratory protective devices when required.

6.0.1.3 Spill Procedures

Spill Procedures and Emergency Response outlined in this CODE.

6.0.1.4 OSHA Requirements

Federal OSHA Requirements: Hazard Communication Standard *(Employee Right-to-Know)* and SDS. Know the location and availability of standard and the written program.

6.0.1.5 Chemical and SDS Lists

Know workplace chemicals list and SDS.

6.0.1.6 Training Plan

Employers shall have a training plan in place and implement training for employees on chemicals used at the AQUATIC FACILITY before their first assignment and whenever a new hazard is introduced into the work area.

6.0.1.6.1 Training Topics

The training shall include at a minimum:

1) How to recognize and avoid chemical hazards;
2) The physical and health hazards of chemicals used at the facility;
3) How to detect the presence or release of a hazardous chemical;
4) Required PPE necessary to avoid the hazards;
5) Use of PPE;
6) Chemical spill response; and
7) How to read and understand the chemical labels or other forms of warning including SDS sheets.

6.0.1.7 Training Records

Records of all training shall be recorded and maintained on file.

6.0.1.8 Body Fluid Exposure

Employees assigned to roles which have the potential for an occupational exposure to bloodborne pathogens, pathogens that cause recreational water illnesses, or other pathogens shall be trained to recognize and respond to body fluid *(blood, feces, vomit)* releases in and around the AQUATIC VENUE area.

6.0.1.9 Exposure Control Program

Employers shall have an Exposure Control Program for bloodborne pathogens as required by OSHA 1910.1030.

6.0.1.10 PPE Provided and Disposed

PPE shall be provided and properly disposed.

6.1 Qualified Operator Training

6.1.1 Qualified Operator Qualifications and Certification

6.1.1.1 Qualifications

A QUALIFIED OPERATOR of an AQUATIC FACILITY shall have completed an operator training course that is recognized by the AHJ.

6.1.1.2 Training Documentation

A QUALIFIED OPERATOR shall have a current certificate or written documentation acceptable to the AHJ showing completion of an operator training course.

6.1.1.2.1 Certificate Available

Originals or copies of such certificate or documentation shall be available on site for inspection by the AHJ for each QUALIFIED OPERATOR employed at or contracted by the site, as specified in this CODE.

6.1.1.2.2 Originals

Originals shall be made available upon request by the AHJ.

6.1.2 Essential Topics in Qualified Operator Training Courses

6.1.2.1 Course Content

All operator training courses recognized by the AHJ shall include, at a minimum, the following teaching elements:

1) Water DISINFECTION,
2) Water chemistry,
3) Mechanical systems, and
4) Health and safety operations.

6.1.2.1.1 Water Disinfection

Water DISINFECTION including:

1) Water DISINFECTION,
2) CT or Concentration X Time values,
3) CHLORINE,
4) Cyanuric acid *(CYA)*,
5) Bromine,
6) BREAKPOINT,
7) SUPERCHLORINATION,
8) HYPERCHLORINATION,
9) Combined CHLORINE,
10) SECONDARY DISINFECTION, and
11) SUPPLEMENTAL DISINFECTION.

6.1.2.1.1.1 Disinfectants

DISINFECTANT types including:

1) Descriptions of different types of DISINFECTANTS,
2) Their unique physical *(e.g., shape or state [solid, liquid, or gas])* and chemical properties *(e.g., how it reacts with acids or bases)*,
3) How they disinfect and impact water chemistry and MONITORING systems,
4) How to calculate dosing,
5) How they are used safely, and
6) The advantages or disadvantages of using each DISINFECTANT.

6.1.2.1.1.2 CT Values

CT or Concentration x Time values including:

1) How to calculate the amount of time needed to inactivate PATHOGENS at a given concentration of a DISINFECTANT, and
2) The importance and reasons for maintaining appropriate water pH and temperature.

6.1.2.1.1.3 Bromine

Bromine including:

1) Definition of bromine as an element,
2) Its use as a residual disinfectant and oxidizer in water,
3) Bromine chemistry,
4) The DISINFECTION role of hypobromous acid,
5) On site generation,
6) pH meter requirements to prevent false readings, and
7) Bromine reuse.

6.1.2.1.1.4 Chlorine

CHLORINE including:

1) Definition of CHLORINE as an element,
2) Its use as a residual disinfectant and oxidizer in water,
3) CHLORINE chemistry and the role of PH,
4) The DISINFECTION role of hypochlorous acid,
5) Unstabilized products *(sodium hypochlorite, calcium hypochlorite, lithium hypochlorite, and chlorine gas)*,
6) Stabilized products *(sodium dichloro-s-triazinetrione and trichloro-s-triazinetrione)*,
7) Safe chemical handling, and
8) On-site CHLORINE generation.

6.1.2.1.1.5 Cyanuric Acid

Cyanuric acid *(CYA)* and stabilized chlorine product use including:

1) Description of CYA and how chlorine is bound to it;
2) Description of CYA use via addition of stabilized chlorine compounds or addition of cyanuric acid alone;
3) Response curves showing the impact of CYA on stabilization of chlorine residuals in the presence of UV;
4) Dose response curves showing the impact of CYA on chlorine kill rates including the impact of CYA concentrations on diarrheal fecal incident remediation procedures;
5) Strategies for controlling the concentration of CYA; and
6) Strategies for reducing the concentration of CYA when it exceeds the maximum allowable level.

6.1.2.1.1.6 Breakpoint/ Super-Chlorination

BREAKPOINT CHLORINATION including how to achieve it through calculation of chemical dosing to reach the desired free CHLORINE level and its relationship to reducing and controlling formation of combined CHLORINE including guidance for how to perform BREAKPOINT CHLORINATION in indoor aquatic settings.

6.1.2.1.1.7 Hyperchlorination

HYPERCHLORINATION including procedures for implementation of fecal/vomit/blood contamination response.

6.1.2.1.1.8 Combined Chlorine

Combined CHLORINE including:

1) How different combined CHLORINE and DISINFECTION BY-PRODUCTS are formed in the water and air;
2) The maximum acceptable level of combined CHLORINE;
3) How methods such as water replacement, BREAKPOINT CHLORINATION, ultraviolet light, ozone, ventilation, and use of other oxidizers can reduce combined CHLORINE level;
4) The advantages and disadvantages of each; and
5) Possible health effects of combined CHLORINE products in the air, particularly in indoor AQUATIC FACILITIES.

6.1.2.1.1.9 Secondary Disinfection

SECONDARY DISINFECTION SYSTEMS including:

1) How ozone and ultraviolet disinfectants are used in conjunction with residual disinfectants to inactivate pathogens, and
2) Sizing guidelines/dosing calculations, safe use, and advantages and disadvantages of each method.

6.1.2.1.1.10 Supplemental Disinfection

SUPPLEMENTAL DISINFECTION including other DISINFECTION chemicals or systems on the market and their effectiveness in water treatment.

6.1.2.1.2 Water Chemistry

Course work for water chemistry shall include:

1) Source water,
2) Water balance,
3) SATURATION INDEX,
4) Water clarity,
5) pH,
6) Total alkalinity,
7) Calcium hardness,
8) Water temperature,
9) Total dissolved solids,
10) Water treatment systems, and
11) Water testing.

6.1.2.1.2.1 Source Water

Source water including requirements for supply and pre-treatment.

6.1.2.1.2.2 Water Balance

Water balance including:

1) Effect of unbalanced water on DISINFECTION, AQUATIC FEATURE surfaces, mechanical equipment, and fixtures; and
2) Details of water balance including pH, total alkalinity, calcium hardness, temperature, and TDS.

6.1.2.1.2.3 Saturation Index

SATURATION INDEX including calculations, ideal values, and effects of values which are too low or too high.

6.1.2.1.2.4 Water Clarity

Water clarity including:

1) Reasons why water quality is so important;
2) Causes of poor water clarity;
3) Maintenance of good water clarity; and
4) Closure requirements when water clarity is poor.

6.1.2.1.2.5 pH

pH including:

1) How pH is a measure of the concentration of hydrogen ions in water;
2) Effects of high and low pH on BATHERS and equipment;
3) Ideal pH range for BATHER and equipment;
4) Factors that affect pH;
5) How pH affects disinfectant efficacy; and
6) How to decrease and increase pH.

6.1.2.1.2.6 Total Alkalinity

Total alkalinity including:

1) How total alkalinity relates to pH;
2) Effects of low and high total alkalinity;
3) Factors that affect total alkalinity;
4) Ideal total alkalinity range, and
5) How to increase or decrease total alkalinity.

6.1.2.1.2.7 Calcium Hardness

Calcium hardness including:

1) Why water naturally contains calcium;
2) How calcium hardness relates to total hardness and temperature;
3) Effects of low and high calcium hardness;
4) Factors that affect calcium hardness;
5) Ideal calcium hardness range; and
6) How to increase or decrease calcium hardness.

6.1.2.1.2.8 Temperature

Water temperature including:

1) How low and high water temperatures increase the likelihood of corrosion and scaling, respectively;
2) Effect on DISINFECTION, its health effects, and other operational considerations;
3) Health effects; and
4) Other operational considerations.

6.1.2.1.2.9 Total Dissolved Solids

Total dissolved solids *(TDS)* including:

1) Why the concentration of TDS increases over time;
2) Association with conductivity and organic CONTAMINANTS; and
3) Key TDS levels as they relate to starting up an AQUATIC FACILITY and galvanic corrosion.

6.1.2.1.2.10 Water Treatment Systems

Water treatment systems including:

1) Descriptions of system use, MONITORING, calibration, and maintenance of automatic controllers;
2) Descriptions of common types of liquid, dry chemical, and gas mechanical feeders;
3) CHLORINE, bromine, and ozone generators;
4) Ultraviolet light systems;
5) Unique features of feeders, generators, and systems;
6) How to generally operate and maintain them;
7) Advantages and disadvantages of different feeders, ultraviolet light systems, and ozonator types; and
8) Alternate treatment methods.

6.1.2.1.2.11 Water Testing

Water testing including:

1) How different methods *(including but not limited to colorimetric, titrimetric, turbidimetric, and electronic)* test water to determine the following levels:
    a. Free available and total CHLORINE,
    b. Total bromine,
    c. pH,
    d. Total alkalinity,
    e. Calcium hardness,
    f. Temperature,
    g. TDS,
    h. CYA,
    i. Metals, and
    j. Any other tests *(including but not limited to salt concentrations, phosphates, nitrates, potassium monopersulfate, copper, iron, and bacterial testing)*;
2) The advantages and disadvantages of each method;
3) How to maintain testing equipment;
4) How to collect water samples;
5) How to perform and interpret tests;
6) How frequently to test;
7) The steps of the dilution method; and
8) How to calculate combined CHLORINE levels.

6.1.2.1.3 Mechanical Systems

Course work for mechanical systems shall include:

1) Calculations,
2) Circulation,
3) Main drains,
4) Gutters and surface SKIMMERS,
5) Mechanical system balance,
6) Circulation pump and motor,
7) Valve,
8) Return INLETS,
9) Filtration, and
10) Filter backwashing/cleaning.

6.1.2.1.3.1 Maintenance Calculations

Calculations including:

1) Explanations of why particular calculations are important;
2) How to convert units of measurement within and between the English and metric systems;
3) How to determine the surface area of regularly and irregularly shape AQUATIC VENUES;
4) How to determine the water volume of regularly and irregularly shaped AQUATIC VENUES; and
5) Why proper sizing of filters, pumps, pipes, and feeders is important.

6.1.2.1.3.2 Circulation

Circulation including:

1) Why circulation is needed;
2) Factors that affect water flow;
3) How direct suction and overflow systems work;
4) How to calculate TURNOVER and flow rates;
5) How the following components of the circulation system relate to each other:
    a. Main drains,
    b. Gutters and surface SKIMMERS,
    c. Circulation pump and motor,
    d. Surge tanks,
    e. Vacuum ports,
    f. Valves, and
    g. Return INLETS;
6) How to read flow meters;
7) How to safely operate pressurized systems after the pump;
8) Information on dye testing;
9) An understanding of total dynamic head *(TDH)*;
10) How it TDH calculated;

11) How TDH is field-determined using vacuum and pressure gauges;
12) TDH effect on pump flow; and
13) Cross connections.

6.1.2.1.3.3 Main Drains

Main drains including:

1) A description of the role of main drains;
2) Why they should not be resized without engineering and public health consultation;
3) The importance of daily inspection of structural integrity; and
4) Discussion on balancing the need to maximize surface water flow while minimizing the likelihood of entrapment.

6.1.2.1.3.4 Gutters & Surface Skimmers

Gutters and surface SKIMMERS including:

1) Why it is important to collect surface water;
2) A description of different gutter types *(at a minimum: scum, surge, and rim-flow)*;
3) How each type generally works;
4) The advantages and disadvantages of each; and
5) Description of the components of SKIMMERS *(e.g., weir, basket, and equalizer assembly)* and their respective roles.

6.1.2.1.3.5 Mechanical System Balance

Mechanical system balance including:

1) An understanding of mechanical system balancing;
2) Methodology for setting proper operational water levels;
3) Basic hydraulics which affect proper functioning of the balance tank and AQUATIC VENUE;
4) Methods of setting and adjusting modulation valves;
5) Balance lines;
6) SKIMMERS;
7) Main drains;
8) The operation of the water make-up system;
9) Collector tanks/gravity drainage systems; and
10) Automatic controllers.

6.1.2.1.3.6 Circulation Pump & Motor

Circulation pump and motor including:

1) Descriptions of the role of the pump and motor;
2) Self-priming and flooded suction pumps;
3) Key components of a pump and how they work together;
4) Cavitation;
5) Possible causes of cavitation; and
6) Troubleshooting problems with the pump and motor.

6.1.2.1.3.7 Valves

Valves including descriptions of different types of valves *(e.g., gate, ball, butterfly/wafer, multi-port, globe, modulating/ automatic, and check)* and their safe operation.

6.1.2.1.3.8 Return Inlets

Return INLETS including a description of the role of return INLETS and the importance of replacing fittings with those that meet original specifications.

6.1.2.1.3.9 Filtration

Filtration including:

1) Why filtration is needed;
2) A description of pressure and vacuum filters and different types of filter media;
3) How to calculate filter surface area;
4) How to read pressure gauges;
5) A general description of sand, cartridge, and diatomaceous earth filters and alternative filter media types to include, at a minimum, perlite, zeolite, and crushed glass;
6) The characteristic flow rates and particle size entrapment of each filter type;
7) How to generally operate and maintain each filter type;
8) Troubleshooting problems with the filter; and
9) The advantages and disadvantages of different filters and filter media.

6.1.2.1.3.10 Filter Backwashing/Cleaning

Filter backwashing/cleaning including:

1) Determining and setting proper backwash flow rates;
2) When backwashing/cleaning should be done and the steps needed for clearing a filter of fine particles and other CONTAMINANTS;
3) Proper disposal of waste water from backwash; and
4) What additional fixtures/equipment may be needed *(i.e., sump, separation tank)*.

6.1.2.1.4 Health and Safety

Course work for health and SAFETY shall include:

1) Recreational water illness *(RWI)*,
2) RWI prevention,
3) Risk management,
4) Record keeping,
5) Chemical SAFETY,
6) Entrapment prevention,
7) Electrical SAFETY,
8) Rescue equipment,
9) Injury prevention,
10) Drowning prevention,
11) BARRIERS,
12) Signage and depth markers,
13) Facility sanitation,
14) Emergency response, and
15) Surveillance and supervision.

6.1.2.1.4.1 Recreational Water Illness

Recreational water illness *(RWI)* including:

1) How water can contain or become contaminated with parasites, bacteria, viruses, fungi, DISINFECTION BY-PRODUCTS, or unsafe levels of chemicals; and
2) The role of the operator in reducing risk.

6.1.2.1.4.2 Causes of RWIs

Common infectious and chemical causes of RWIs, including but not limited to:

1) Diarrheal illness *(Cryptosporidium, Giardia, Shigella, and norovirus)*;
2) Skin rashes *(Pseudomonas aeruginosa, molluscum contagiosum)*;
3) Respiratory illness *(Legionella)*;
4) Neurologic infections *(echovirus, Naegleria)*;
5) Eye/ear illness *(Pseudomonas aeruginosa, adenovirus, Acanthamoeba);*
6) Hypersensitivity reactions *(Mycobacterium avium complex, Pontiac fever, endotoxins)*; and
7) Health effects of chloramines and DISINFECTION BY-PRODUCTS.

6.1.2.1.4.3 RWI Prevention

Recreational water illness *(RWI)* prevention including:

1) Methods of prevention of RWIs, including but not limited to chemical level control;
2) Why public health, operators, and PATRONS need to be educated about RWIs and collaborate on RWI prevention;
3) The role of showering;
4) The efficacy of swim diapers;
5) Formed-stool and diarrheal fecal incident response; and
6) Developing a plan to minimize PATHOGEN and other biological *(e.g., blood, vomit, sweat, urine, and skin and hair care products)* contamination of the water.

6.1.2.1.4.4 Risk Management

Risk management including techniques that identify hazards and risks and that prevent illness and injuries associated with AQUATIC FACILITIES open to the public.

6.1.2.1.4.5 Record Keeping

Record keeping including the need to keep accurate and timely records of the following areas:

1) Operational conditions *(e.g., water chemistry, water temperature, filter pressure differential, flow meter reading, and water clarity)*;
2) Maintenance performed *(e.g., backwashing, change of equipment)*;
3) Incidents and response *(e.g., fecal incidents in the water and injuries)*; and
4) Staff training and attendance.

6.1.2.1.4.6 Chemical Safety

Chemical SAFETY including steps to safely store and handle chemicals including:

1) How to read labels and material safety data sheets;
2) How to prevent individual chemicals and inorganic and organic CHLORINE products from mixing together or with other substances *(including water)* or in chemical feeders; and
3) Use of PPE.

6.1.2.1.4.7 Entrapment Prevention

Entrapment prevention including:

1) Different types of entrapment *(e.g., hair, limb, body, evisceration/disembowelment, and mechanical)*;
2) How to prevent and/or decrease likelihood of entrapment; and
3) Requirements of the Virginia Graeme Baker Pool and Spa Safety Act.

6.1.2.1.4.8 Electrical Safety

Electrical SAFETY including possible causes of electrical shock and steps that can be taken to prevent electrical shock *(e.g., bonding, grounding, ground fault interrupters, and prevention of accidental immersion of electrical devices)*.

6.1.2.1.4.9 Rescue Equipment

Rescue equipment including a description and rationale for the most commonly found rescue equipment including:

1) Rescue tubes,
2) Reaching poles,
3) Ring buoys and throwing lines,
4) Backboards,
5) First aid kits,
6) Emergency alert systems,
7) Emergency phones with current numbers posted, and
8) Resuscitation equipment.

6.1.2.1.4.10 Injury Prevention

Injury prevention including basic steps known to decrease the likelihood of injury, at a minimum:

1) Banning glass containers at AQUATIC FACILITIES,
2) PATRON education, and
3) Daily visual inspection for hazards.

6.1.2.1.4.11 Drowning Prevention

Drowning prevention including causes and prevention of drowning.

6.1.2.1.4.12 Barriers

BARRIERS including descriptions of how fences, gates, doors, and safety covers can be used to prevent access to water; and basics of design that effectively prevent access to water.

6.1.2.1.4.13 Signage & Depth Markers

Signage and depth markers including the importance of maintaining signage and depth markers.

6.1.2.1.4.14 Facility Sanitation

Facility sanitation including:

1) Steps to clean and disinfect all surfaces that PATRONS would commonly come in contact with *(e.g., deck, restrooms, and diaper-changing areas)*, and
2) Procedures for implementation of MAHC Section 6.5: *Fecal-Vomit-Blood Contamination Response*, in relation to responding to a body fluid spill on these surfaces.

6.1.2.1.4.15 Emergency Response Plan

Emergency response plan including:

1) Steps to respond to emergencies *(at a minimum, severe weather events, drowning or injury, contamination of the water, chemical incidents)*; and
2) Communication and coordination with emergency responders and local health department notification as part of an EMERGENCY ACTION PLAN.

6.1.2.1.5 Operations

Course work for operations shall include:

1) Regulations,
2) Local and state health departments,
3) AQUATIC FACILITY types,
4) Daily/routine operations,
5) Preventive maintenance,
6) Weatherizing,
7) AQUATIC FACILITY renovation and design,
8) Heating,
9) Air circulation, and
10) SPA and THERAPY POOL issues.

6.1.2.1.5.1 Regulations

Regulations including the application of local, regional, state, and federal regulations and STANDARDS relating to the operation of AQUATIC FACILITIES.

6.1.2.1.5.1.1 Immediate Closure

Course work shall also highlight reasons why an inspector or operator would immediately close an AQUATIC FACILITY.

6.1.2.1.5.2 Local & State Health Departments

Local and state health departments including stressing the importance of a good working relationship with the local and state health department.

6.1.2.1.5.3 Aquatic Facility Types

AQUATIC FACILITY types including common AQUATIC VENUE types and settings and a discussion of features and play equipment that require specific operation and maintenance steps.

6.1.2.1.5.4 Daily/Routine Operations

Daily/routine operations including listing and describing the daily inspection and maintenance requirements of an AQUATIC FACILITY including, but not limited items listed:

1) Walkways/DECK and exits are clear, clean, free of debris;
2) Drain covers, vacuum fitting covers, SKIMMER equalizer covers, and any other suction outlet covers are in place, secure, and unbroken;
3) SKIMMER baskets, weirs, lids, flow adjusters, and suction outlets are free of any blockage;
4) INLET and return covers and any other fittings are in place, secure, and unbroken;
5) SAFETY warning signs and other signage are in place and in good repair;
6) Entrapment prevention systems are operational;
7) Recirculation, DISINFECTION systems, controller*(s)*, and probes are operating as required;
8) Secondary and/or supplemental DISINFECTION systems are operating as required;
9) Underwater lights and other lighting are intact with no exposed wires or water in lights;
10) Slime and biofilm has been removed from accessible surfaces of AQUATIC VENUE, slides, and other AQUATIC FEATURES;
11) Doors to nonpublic areas *(chemical storage spaces, offices, etc.)* are locked;
12) First aid supplies are stocked;
13) Emergency communication equipment and systems are operational;
14) Fecal/vomit/blood incident contamination response protocols, materials, and equipment are available;
15) AQUATIC FEATURES and amenities are functioning in accordance with the manufacturer's recommendations;
16) Fencing/BARRIERS, gates, and self-latching or other locks are tested and are intact and functioning properly, and BARRIERS do not have nearby furniture to encourage climbing;
17) Drinking fountains are clean and in functional condition;
18) Electrical devices are in good working condition and meet the requirements specified in the NEC and MAHC;
19) Alarms, if required, are tested and functioning properly;
20) Assessing glare conditions throughout operating hours to assess whether the bottom and objects in the POOL are clearly visible;
21) Play structures and diving boards are in good condition;
22) SAFETY equipment is in good condition, properly secured, accessible for intended use, and shall include at a minimum:
    a. Rescue tubes,
    b. Resuscitation masks with one-way valve,
    c. First aid kits,

   d. AED's,
   e. Emergency oxygen,
   f. Backboard, head immobilizer, straps, and
   g. Lifeguard stands;

23) Emergency shut-off systems *(slides, water features, pumps, etc.)* function properly;
24) Depth markings are clearly visible;
25) Lifelines and buoys are in place and in good working order;
26) Ladders are non-slip and rungs secured tightly;
27) WATERSLIDES are in functional, safe condition;
28) Moveable fulcrum is adjusted properly to control spring in the board as necessary;
29) Moveable starting blocks are properly stored;
30) Access to permanent starting blocks is restricted or controlled when not in use by swim teams and prohibited when not in use by competitive swimming or swimming practice that is under direct supervision of an instructor or coach;
31) Railings are secure;
32) SVRS is functioning according to manufacturer's guidelines;
33) SKIMMER baskets and covers are clean and in place;
34) Water quality and clarity is MAHC compliant;
35) Water level is at an appropriate level;
36) Pumps retain the appropriate pressure;
37) Play structures are secure *(consider water velocity and reference manufacturers recommended levels)*; and
38) Verify required documentation and records are in place and signed by the appropriate personnel.

6.1.2.1.5.5 Preventive Maintenance

Preventive maintenance including how to develop:

1) A preventive maintenance plan,
2) Routine maintenance procedures, and
3) Record keeping system needed to track maintenance performed.

6.1.2.1.5.6 Weatherizing

Weatherizing including the importance of weatherizing and the steps to prevent damage to AQUATIC FACILITIES and their mechanical systems due to very low temperatures or extreme weather conditions *(e.g., flooding)*.

6.1.2.1.5.7 Facility Renovation & Design

AQUATIC FACILITY renovation and design including:

1) Definitions of AQUATIC FACILITY renovation, remodeling, and SUBSTANTIAL ALTERATION;
2) When it is necessary to renovate;
3) When it is necessary to notify the AHJ of planned renovations and remodeling; and
4) Current trends in facility renovation and design.

6.1.2.1.5.8 Heating

Heating issues including:

1) Recommended water temperatures and limits,
2) Factors that contribute to the water's heat loss and gain,
3) Heating equipment options,
4) Sizing gas heaters, and
5) How to troubleshoot problems with heaters.

6.1.2.1.5.9 Air Circulation

Air circulation including:

1) AIR HANDLING SYSTEM considerations for an indoor AQUATIC FACILITY,
2) The importance of regulating humidity,
3) The need to maintain negative pressure,
4) How poor indoor air quality can affect PATRONS and staff, and
5) How to balance air change and energy efficiency.

6.1.2.1.5.10 Spa & Therapy Pool Issues

SPA and THERAPY POOL issues including:

1) Operational implications of smaller volumes of water and HOT WATER,
2) How to maintain water chemistry,
3) Typical water temperature ranges highlighting maximum temperatures,
4) Risks of hyperthermia and hypothermia,
5) Need for emergency shut-off switches, and
6) Frequency of cleaning, draining, and DISINFECTION.

6.1.3 General Requirements for Operator Training Courses

6.1.3.1 Course Providers

6.1.3.1.1 Recognized Courses

Providers of recognized operator training courses, if required by the AHJ to verify that the course meets the requirements and intent of this CODE, shall submit course information including:

1) Course development expertise,
2) Course content,
3) Course length,
4) Instructor qualifications,
5) Exam administration,
6) Certificate procedures, and
7) Updates of information as changes are made.

6.1.3.1.2 Providers

Operator training courses shall be developed by individuals or organizations with expertise in AQUATIC FACILITY operation and maintenance and expertise in education or training as evidenced by combined work experience and training.

6.1.3.2 Course Content

Training materials at a minimum, covering all of the essential topics as outlined in MAHC Section 6.1.2.1 shall be provided and used in operator training courses.

6.1.3.3 Course Length

Course agenda or syllabus shall show amount of time planned to cover each of the essential topics.

6.1.3.4 Instructor Requirements

Operator training course providers shall furnish course instructor information including:

1) Expertise in AQUATIC FACILITY operation and maintenance—as evidenced by work experience and/or training;
2) Completion of an operator training course, which at a minimum, covers all of the essential topics as outlined in MAHC Section 6.1.2.1, including passing the final exam;
3) Successful completion of an operator training instructor course; and
4) If the operator training course is online, procedures which make such an instructor available to answer students' questions during normal business hours.

6.1.3.5 Final Exam

Operator training course providers shall furnish course final exam information including:

1) Final exam*(s)*, which at a minimum, covers all of the essential topics as outlined in MAHC Section 6.1.2.1;
2) Final exam passing score criteria; and
3) Final exam security procedures.

6.1.3.5.1 Final Exam Administration

Operator training course providers shall provide final exam administration, proctoring and security procedures including:

1) Checking student's government-issued photo identification, or another established process, to ensure that the individual taking the exam is the same person who is given a certificate documenting course completion and passing of exam,
2) Final exam completion is without assistance or aids that are not allowed by the training agency, and
3) Final exam is passed, prior to issuance of a QUALIFIED OPERATOR certificate.

6.1.3.6 Course Certificates

Operator training course providers shall furnish course certificate information including:

1) Procedures for issuing nontransferable certificates to the individuals who successfully complete the course work and pass the final exam;
2) Procedures for delivery of course certificates to the individuals who successfully complete the course work and pass the final exam;
3) Instructions for the participant to maintain their originally issued certificate, or a copy thereof, for the duration of its validity; and
4) Procedures for the operator training course provider to maintain an individual's training and exam record for a minimum period of five years after the expiration of the individual's certificate.

6.1.3.7 Continuing Education [N/A]

6.1.3.8 Certificate Renewal

Operator training course providers shall furnish course certificate renewal information including:

1) Criteria for re-examination with a renewal exam that meets the specifications for initial exam requirements and certificate issuance specified in this CODE; or
2) Criteria for a refresher course with an exam that meets the specifications for the initial course, exam, and certificate issuance requirements specified in this CODE.

6.1.3.9 Certificate Suspension and Revocation

Course providers shall have procedures in place for the suspension or revocation of certificates.

6.1.3.9.1 Evidence of Health Hazard

Course providers may suspend or revoke a QUALIFIED OPERATOR'S certificate based on evidence that the QUALIFIED OPERATOR'S actions or inactions unduly created SAFETY and health hazards.

6.1.3.9.2 Evidence of Cheating

Course providers may suspend or revoke a QUALIFIED OPERATOR'S certificate based on evidence of cheating or obtaining the certificate under false pretenses.

6.1.3.10 Additional Training or Testing

The AHJ may, at its discretion, require additional operator training or testing.

6.1.3.11 Certificate Recognition

The AHJ may, at its discretion, choose to recognize, not to recognize, or rescind a previously recognized certificate of a QUALIFIED OPERATOR based upon demonstration of inadequate knowledge, poor performance, or due cause.

6.1.3.12 Course Recognition

The AHJ may, at its discretion, recognize, choose not to recognize, or revoke a previously accepted course based upon demonstration of inadequate knowledge or poor performance of its QUALIFIED OPERATORS, or due cause.

6.1.3.13 Length of Certificate Validity

The maximum length of validity for QUALIFIED OPERATOR training certificate shall be five years.

6.2 Lifeguard Training

6.2.1 Lifeguard Qualifications

A QUALIFIED LIFEGUARD shall:

1) Have successfully completed an AHJ-recognized lifeguard training course offered by an AHJ-recognized training agency,
2) Possess a current certificate for such training,
3) Have met all pre-service requirements, and
4) Participate in continuing in-service training requirements of the AQUATIC FACILITY.

6.2.1.1 Course Content

Lifeguard Training Courses shall include but not be limited to:

1) Hazard identification and injury prevention,
2) Emergencies,
3) CPR,
4) AED use,
5) First aid, and
6) Legal issues.

6.2.1.1.1 Hazard Identification and Injury Prevention

Hazard identification and injury prevention shall include:

1) Identification of common hazards or causes of injuries and their prevention;
2) Responsibilities of a QUALIFIED LIFEGUARD in prevention strategies;
3) Victim recognition;
4) Victim recognition scanning strategies;
5) Factors which impede victim recognition;
6) Health and SAFETY issues related to lifeguarding; and
7) Prevention of voluntary hyperventilation and extended breath holding activities.

6.2.1.1.2 Emergency Response Skill Set

Emergency response content shall include:

1) Responsibilities of a QUALIFIED LIFEGUARD in reacting to an emergency;
2) Recognition and identification of a person in distress and/or drowning;
3) Methods to communicate in response to an emergency;
4) Rescue skills for a person who is responsive or unresponsive, in distress, or drowning;
5) Skills required to rescue a person to a position of SAFETY;
6) Skills required to extricate a person from the water with assistance from another lifeguard*(s)* and/or patron*(s)*; and
7) Knowledge of the typical components of an EMERGENCY ACTION PLAN *(EAP)* for AQUATIC VENUES.

6.2.1.1.3 Resuscitation Skills

CPR/AED, AED use, and other resuscitation skills shall be professional level skills that follow treatment protocols consistent with the current ECCU and/or, the ILCOR guidelines for cardiac compressions, foreign body restriction removal, and rescue breathing for infants, children, and adults.

6.2.1.1.4 First Aid

First Aid training shall include:

1) Basic treatment of bleeding, shock, sudden illness, and muscular/skeletal injuries as per the guidelines of the National First Aid Science Advisory Board;
2) Knowing when and how to activate the EMS;
3) Rescue and emergency care skills to minimize movement of the head, neck and spine until EMS arrives for a person who has suffered a suspected spinal injury on land or in the water; and
4) Use and the importance of universal precautions and personal protective equipment in dealing with body fluids, blood, and preventing contamination according to current OSHA guidelines.

6.2.1.1.5 Legal Issues

Course content related to legal issues shall include but not be limited to:

1) Duty to act,
2) Standard of care,
3) Negligence,
4) Consent,
5) Refusal of care,
6) Abandonment,
7) Confidentiality, and
8) Documentation.

6.2.1.2 Lifeguard Training Delivery

6.2.1.2.1 Standardized and Comprehensive

The educational delivery system shall include standardized student and instructor materials to convey all topics including but not limited to those listed per MAHC Section 6.2.1.1.

6.2.1.2.2 Skills Practice

Physical training of lifeguarding skills shall include in-water and out-of-water skill practices led by an individual currently certified as an instructor by the training agency which developed the lifeguard course materials.

6.2.1.2.3 Shallow Water Training

If a training agency offers a certification with a distinction between “shallow water” and “deep water” lifeguards, candidates for shallow water certification shall have training and evaluation in the deepest depth allowed for the certification.

6.2.1.2.4 Deep Water Training

If a training agency offers a certification with a distinction between "shallow water" and "deep water" lifeguards, candidates for deep water certification shall have training and evaluation in at least the minimum depth allowed for the certification.

6.2.1.2.5 Sufficient Time

Course length shall provide sufficient time to cover content, practice, skills, and evaluate competency for the topics listed in MAHC Section 6.2.1.1.

6.2.1.2.6 Certified Instructors

Lifeguard instructor courses shall be taught only by individuals currently certified as instructors by the training agency which developed the lifeguard course materials.

6.2.1.2.6.1 Minimum Prerequisites

Lifeguard training agencies shall develop minimum instructor prerequisites that include, but are not limited to those outlined in MAHC Section 6.2.1.2.6.2.

6.2.1.2.6.2 Completed Training

Prior to instructing lifeguard training, instructors are required to have successfully completed a lifeguard training course which complies with MAHC Section 6.2.1.1 and a lifeguard instructor training course which includes, at a minimum, the following:

1) Mastery and knowledge of lifeguard training course content;
2) Demonstration of the ability to effectively deliver lifeguard training course content;
3) An evaluation and feedback process to improve instructor candidate presentation skills/techniques;
4) Course management and administration procedures; and
5) Testing and evaluation procedures.

6.2.1.2.6.3 Instructor Renewal/Recertification Process

Lifeguard training agencies shall have a lifeguard instructor renewal/recertification process.

6.2.1.2.6.4 Quality Control

Training agencies shall have a quality control system in place for evaluating a lifeguard instructor's ability to conduct courses.

6.2.1.2.7 Training Equipment

All lifeguard training courses shall have, at a minimum, the following pieces of equipment available in appropriate student to equipment ratios during the course:

1) Rescue Tubes,
2) Backboard with head immobilizer and sufficient straps to immobilize the victim to the backboard,
3) CPR manikins *(Adult and infant)*,

4) Resuscitation mask with one-way valve,
5) Bag Valve Mask *(Adult and Pediatric)*,
6) Disposable gloves,
7) AED Trainer with adult and pediatric training pads,
8) First Aid Supplies for first aid training, and
9) Manikin cleaning supplies.

6.2.1.3 Competency and Certification

6.2.1.3.1 Proficiency

Lifeguarding skills per MAHC Section 6.2.1.1 shall be tested, by a certified instructor, to a level of proficiency accepted by the training agency.

6.2.1.3.2 Requirements

Lifeguard training course providers shall have a final exam including but not limited to:

1) Written and practical exams covering topics outlined in MAHC Section 6.2.1.1;
2) Final exam passing score criteria including the level of proficiency needed to pass practical and written exams; and
3) Security procedures for proctoring the final exam to include:
    a. Checking student's government-issued photo identification, or another established process, to ensure that the individual taking the exam is the same person who is given a certificate documenting course completion and passing of exam; and
    b. Final exam is passed, prior to issuance of a certificate.

6.2.1.3.3 Instructor Physically Present

The instructor of record shall be physically present during the practical testing.

6.2.1.3.4 Certifications

Lifeguard and lifeguard instructor certifications shall be issued to recognize successful completion of the course as per the requirements of MAHC Section 6.2.1.1 through 6.2.1.3.8.

6.2.1.3.5 Number of Years

Length of valid certification shall be a maximum of two years for lifeguarding and first aid, and a maximum of one year for Cardiopulmonary Resuscitation *(CPR/AED)*.

6.2.1.3.6 Documentation

Course documentation of training and certificates shall identify the following:

1) Name of trainee,
2) Level of training,
3) Expiration date,
4) Restrictions on depth of water for which the lifeguard is qualified,
5) Identifier of the instructor of record,
6) Any other restrictions that maybe applied by the training agency, and
7) Identifier of the agency providing the certification.

6.2.1.3.7 Expired Certificate

When a certificate has expired for more than 45 days, the QUALIFIED LIFEGUARD shall retake the course or complete a challenge program.

6.2.1.3.7.1 Challenge Program

A QUALIFIED LIFEGUARD challenge program, when utilized, shall be completed in accordance with the training of the original certifying agency, by an instructor certified by the original certifying agency, and include but not be limited to:

1) Pre-requisite screening;
2) A final practical exam demonstrating all skills, in and out of the water required in the original lifeguard course for certification, which complies with MAHC Section 6.2.1.1, and uses the equipment specified in MAHC Section 6.2.1.2.7; and
3) Final written, proctored exam.

6.2.1.3.7.2 Certificate Renewal

Certificate renewal, when used, shall include the following:

1) Completion no later than 45 days after certificate expiration;
2) Conducted in accordance with the training of the original certifying agency;
3) Taught by an instructor certified by the original certifying agency;
4) Conducted with a demonstration of skills, in and out of the water, required in the original course, which complies with MAHC Section 6.2.1.1, and uses the equipment specified in MAHC Section 6.2.1.2.7;
5) A final written, proctored exam; and
6) A final practical exam with a certified instructor(s) of record present; or
7) Completion of a Challenge Program in accordance with MAHC Section 6.2.1.3.7.1, no later than 45 days after certificate expiration.

6.2.1.3.8 Certificate Suspension and Revocation

Lifeguard training agencies shall have procedures in place for the suspension or revocation of certificates.

6.2.2 Aquatic Supervisor Training

6.2.2.1 Lifeguard Supervisor Candidate Prerequisites

LIFEGUARD SUPERVISOR candidate prerequisites shall include but not be limited to:

1) Successful completion of a lifeguard training course in the past;
2) Previous experience as a lifeguard of at least three months; and
3) Ability to effectively communicate verbally in English.

6.2.2.2 Lifeguard Supervisor Training Elements

LIFEGUARD SUPERVISOR training shall include, at a minimum, the following:

1) Activation and execution of EMERGENCY ACTION PLANS *(EAPs)*,
2) CPR/AED and first aid training that complies with MAHC Sections 6.2.1.1.3 and 6.2.1.1.4 or present an unexpired certificate issued by an AHJ-approved agency documenting the required training has been completed;
3) Scanning and vigilance requirements and how to ensure that systems which accomplish these goals are in place and operational;
4) Development and evaluation of zones of BATHER surveillance responsibility diagrams for an AQUATIC VENUE;
5) MONITORING lifeguard performance as it relates to lifeguard and facility-specific training, including pre-service assessments;
6) Strategies to reduce risk and mitigate the health and SAFETY hazards to both the PATRONS and the staff;
7) Knowledge of the legal issues and responsibilities relating to lifeguarding as listed in MAHC Section 6.2.1.1.5; and
8) Knowledge of the proper use and maintenance of the equipment required per MAHC Section 5.8.5.

6.2.2.3 Lifeguard Supervisor Training Delivery

6.2.2.3.1 Standardized and Comprehensive

The educational delivery system shall include standardized student and instructor content and delivery to convey all topics including but not limited to those listed per MAHC Section 6.2.2.2.

6.2.2.3.2 Sufficient Time

Course length shall provide sufficient time to cover content, demonstration, skill practice, and evaluate competency for the topics listed in MAHC Section 6.2.2.2.

6.2.2.3.3 Course Setting

LIFEGUARD SUPERVISOR training courses shall be:

1) Taught in person by a trained lifeguard supervisor instructors; or
2) Blended learning offerings with electronic content deliverables created, and presented by, and in-person portions taught by, trained lifeguard supervisor instructors; or
3) On-line offerings created and presented by trained lifeguard supervisor instructors.

6.2.2.3.4 Lifeguard Supervisor Course Instructor Certification

Lifeguard supervisor course instructors shall be certified through a training agency or by the facility whose training programs meets the requirements specified in MAHC Section 6.2.2.

6.2.2.3.4.1 Lifeguard Supervisor Course Instructor

Lifeguard supervisor course shall be taught by trained LIFEGUARD SUPERVISOR instructors through a training agency or by the facility whose training programs meets the requirements specified in MAHC Section 6.2.2.

6.2.2.3.4.2 Minimum Prerequisites

Course providers shall develop minimum instructor prerequisites that include, but are not limited to:

1) Successful completion of a lifeguard training course in the past;
2) Successful completion of a LIFEGUARD SUPERVISOR training course that complies with MAHC Section 6.2.2.2;
3) Ability to effectively communicate in English;
4) Has completed a LIFEGUARD SUPERVISOR instructor training course which includes, at a minimum, the following:
5) Mastery and knowledge of LIFEGUARD SUPERVISOR training course content,
6) Demonstration of the ability to effectively deliver LIFEGUARD SUPERVISOR training course content,
7) An evaluation and feedback process to improve instructor candidate presentation skills/techniques,
8) Course management and administration procedures, and
9) Testing and evaluation procedures.

6.2.2.3.4.3 Quality Control

Course provider shall have a quality control system in place for evaluating a LIFEGUARD SUPERVISOR instructor's ability to conduct courses.

6.2.2.3.4.4 Lifeguard Supervisor Renewal & Recertification

LIFEGUARD SUPERVISOR training agencies shall have a LIFEGUARD SUPERVISOR instructor renewal/recertification process.

6.2.2.4 Competency and Certification

6.2.2.4.1 Lifeguard Supervisor Proficiency

LIFEGUARD SUPERVISOR training course providers shall have a method to evaluate proficiency of the content in MAHC Section 6.2.2.2.

6.2.2.4.2 Lifeguard Supervisor Certifications

LIFEGUARD SUPERVISOR certifications shall be issued by the course provider to recognize successful completion of the course as per the requirements of MAHC Section 6.2.2.2.

6.3 Facility Staffing

6.3.1 Qualified Operator Requirements and Availability

6.3.1.1 On-Site Qualified Operator Requirements

6.3.1.1.1 At Adoption

The following MAHC sections shall be required for all AQUATIC FACILITIES at time of adoption:

1) MAHC Section 6.3.1.1: On-Site QUALIFIED OPERATORS, and
2) MAHC Section 6.3.1.2: Contracted Off-Site QUALIFIED OPERATORS.

6.3.1.1.2 Size and Use

A QUALIFIED OPERATOR shall be on-site or immediately available within two hours during all hours of operation at an AQUATIC FACILITY that has:

1) More than two AQUATIC VENUES; or
2) An AQUATIC VENUE of over 50,000 gallons of water; or
3) AQUATIC VENUES that include AQUATIC FEATURES with recirculated water; or
4) An AQUATIC VENUE used as a THERAPY POOL; or
5) An AQUATIC VENUE used to provide swimming training.

6.3.1.1.3 Bathers and Management

A QUALIFIED OPERATOR shall be on site or immediately available within two hours during all hours of operation at an AQUATIC FACILITY that is:

1) Permitted BATHER COUNT is greater than 200 BATHERS daily; or
2) Operated by a municipality; or
3) Operated by a school.

6.3.1.1.4 Compliance History

A QUALIFIED OPERATOR shall be available on-site or immediately available within two hours during all hours of operation at an AQUATIC FACILITY that has a history of CODE

violations which in the opinion of the permit issuing official require one or more on-site QUALIFIED OPERATORS.

6.3.1.2 Contracted Off-site Qualified Operators

All other AQUATIC FACILITIES shall have an on-site QUALIFIED OPERATOR immediately available within two hours or a contract with a QUALIFIED OPERATOR for a minimum of weekly visits and assistance whenever needed.

6.3.1.2.1 Visit Documentation

Written documentation of these visits for contracted off-site QUALIFIED OPERATOR visits and assistance consultations shall be available at the AQUATIC FACILITY for review by the AHJ.

6.3.1.2.2 Documentation Details

The written documentation shall indicate the checking, MONITORING, and testing outlined in MAHC 6.4.1.2.2.1 and 6.4.1.2.5 and, when applicable, 6.4.1.2.2.2.

6.3.1.2.3 Visit Corrective Actions

The written documentation shall indicate what corrective actions, if any, were taken by the contracted off-site QUALIFIED OPERATOR during the scheduled visits or assistance requests.

6.3.1.2.4 Onsite Responsible Supervisor

All AQUATIC FACILITIES without a full time on-site QUALIFIED OPERATOR shall have a designated on-site RESPONSIBLE SUPERVISOR.

6.3.1.2.5 Onsite Responsible Supervisor Duties

The designated on-site RESPONSIBLE SUPERVISOR shall:

1) Be capable of testing and recording the water quality parameters required by this CODE;
2) Know how to make adjustments, as needed, to maintain required water quality parameters required by this CODE;
3) Know general maintenance procedures as required by daily operational verifications or adjustments required by this CODE;
4) Know when the AQUATIC FACILITY or individual AQUATIC VENUE should be closed; and
5) Know how and when to contact the contracted off-site QUALIFIED OPERATOR.

6.3.2 Aquatic Facilities Requiring Qualified Lifeguards

AQUATIC VENUES with standing water and with any of the following conditions listed in MAHC Section 6.3.2.1 shall be required to have a lifeguard*(s)* conducting PATRON surveillance at all times the AQUATIC VENUE is open.

6.3.2.1 List of Aquatic Facilities Requiring Qualified Lifeguards

Note: This list includes but shall not be limited to the following:

1) For new construction occurring from the date of acceptance of this CODE, any AQUATIC VENUE deeper than five feet *(1.5 m)* at any point;
2) Any AQUATIC VENUE that allows for unsupervised children under the age of 14 years;
3) Any AQUATIC VENUE while it is being used for the recreation of youth groups, including but not limited to childcare usage or school groups;
4) Any AQUATIC VENUE while it is being used for group training must have a dedicated lifeguard on deck for class surveillance, including but not limited to competitive swimming and/or sports, lifeguard training, exercise programs, and swimming lessons;
5) Any AQUATIC VENUE with a configuration in which any point on the AQUATIC VENUE surface exceeds 30 feet *(9.1 m)* from the nearest DECK;
6) Any AQUATIC VENUE with an induced current or wave action including but not limited to WAVE POOLS and LAZY RIVERS;
7) Waterslide LANDING POOLS; and
8) Any AQUATIC VENUE in which bathers enter the water from any height above the deck including but not limited to diving boards, drop slides, starting platforms, and/or climbing walls. This does not include POOL SLIDES.

6.3.3 Safety Plan

All AQUATIC FACILITIES shall create and implement a SAFETY PLAN to include, but not be limited to the following elements:

1) Staffing Plan,
2) EMERGENCY ACTION PLAN,
3) Biohazard action plan,
4) Pre-Service Training Plan, and
5) In-service Training Plan.

6.3.3.1 Code Compliance Staff Plan

Staffing Plans shall designate person(s) as members of the Safety Team and person(s) for the following responsibilities:

1) Identifying and communicating health and SAFETY hazards;
2) Mitigating health and SAFETY hazards and closing the facility if needed;
3) Interfacing with the AHJ related to the requirements of this CODE;
4) Maintaining water quality and, if required, air quality;

5) Enforcing the AQUATIC FACILITY rules and regulations;
6) Responding to reported emergencies;
7) Supervising the SAFETY TEAM;
8) Conducting pre-service evaluations; and
9) Conducting in-service training.

6.3.3.1.1 Zone of Patron Surveillance

When QUALIFIED LIFEGUARDS are used, the staffing plan shall include diagrammed zones of PATRON surveillance for each AQUATIC VENUE such that:

1) The QUALIFIED LIFEGUARD is capable of viewing the entire area of the assigned zone of PATRON surveillance,
2) The QUALIFIED LIFEGUARD is able to reach the furthest extent of the assigned zone of PATRON surveillance within 20 seconds,
3) Identify whether the QUALIFIED LIFEGUARD is in an elevated stand, walking, in-water and/or other approved position,
4) Identifying any additional responsibilities for each zone, and
5) All areas of each AQUATIC VENUE are assigned a zone of PATRON surveillance.

6.3.3.1.2 Rotation Procedures

When QUALIFIED LIFEGUARDS are used, the staffing plan shall include QUALIFIED LIFEGUARD rotation procedures such that:

1) Identifying all zones of PATRON surveillance responsibility at the AQUATIC FACILITY;
2) Operating in a manner so as to provide an alternation of tasks such that no QUALIFIED LIFEGUARD conducts PATRON surveillance activities for more than 60 continuous minutes; and
3) Have a practice of maintaining coverage of the zone of PATRON surveillance during the change of the QUALIFIED LIFEGUARD.

6.3.3.1.3 Alternation of Tasks

Alternation of tasks may include any one of the following:

1) Change of zone of PATRON surveillance where the QUALIFIED LIFEGUARD must walk or be transported to another zone of PATRON surveillance.
2) Have a period of at least 10 minutes of non-PATRON surveillance activity such as taking a break, conducting maintenance, or conducting ride dispatch.

6.3.3.1.4 Supervision Protocols

When QUALIFIED LIFEGUARDS are used, the STAFFING PLAN shall include lifeguard supervision protocols to achieve the requirements of MAHC Section 6.3.3.

6.3.3.2 Emergency Action Plan

EMERGENCY ACTION PLANS and operating procedures shall include but not be limited to:

1) Outline types of emergencies and IMMINENT HEALTH HAZARDS, as per MAHC Section 6.6.3;
2) Outline the methods of communication between responders, emergency services, and PATRONS;
3) Identify each anticipated responder;
4) Outline the tasks of each responder;
5) Identify required equipment for each task; and
6) Emergency closure requirements.

6.3.3.2.1 Coordination of Response

When one or more QUALIFIED LIFEGUARDS are used, the SAFETY PLAN and the EMERGENCY ACTION PLAN shall identify the best means to provide additional persons to rapidly respond to the emergency to help the initial rescuer.

6.3.3.3 Pre-Service Requirements

The Pre-Service Plan shall include:

1) Policies and procedure training specific to the AQUATIC FACILITY,
2) Demonstration of SAFETY TEAM skills specific to the AQUATIC FACILITY prior to assuming on-duty lifeguard responsibilities, and
3) Documentation of training.

6.3.3.3.1 Safety Team EAP Training

Prior to active duty, all members of the SAFETY TEAM shall be trained on, and receive a copy of, and/or have a copy posted and always available of the specific policies and procedures for the following:

1) Staffing Plan,
2) EMERGENCY ACTION PLAN,
3) Emergency closure, and
4) Fecal, vomit, and blood contamination on surfaces and in the water as outlined in MAHC Section 6.5.

6.3.3.3.2 Safety Team Skills Proficiency

Prior to active duty, all members of the SAFETY TEAM shall demonstrate knowledge and skill competency specific to the AQUATIC FACILITY for the following criteria:

1) Understand their responsibilities and of others on the AQUATIC FACILITY SAFETY TEAM;
2) Ability to execute the EMERGENCY ACTION PLAN;
3) Know what conditions require closure of the facility; and
4) Know what actions to take in response to a fecal, vomit, or blood contamination on a surface and in the water as outlined in MAHC Section 6.5.

6.3.3.3.3 Qualified Lifeguard Emergency Action Plan Training

When QUALIFIED LIFEGUARDS are used, they shall be trained on, and receive a copy of, and/or have a copy of the EAP posted and always available at the AQUATIC FACILITY, the specific policies and procedures for the following:

1) Zone of PATRON Surveillance Plan,
2) Rotation Plan,
3) Minimum Staffing Plan, and
4) Rescue/First Aid Response plan.

6.3.3.3.4 Qualified Lifeguard Skills Proficiency

When QUALIFIED LIFEGUARDS are used, they shall demonstrate knowledge and skill competency specific to the AQUATIC FACILITY for the following criteria:

1) Ability to reach the bottom at the maximum water depth of the venue to be assigned;
2) Ability to identify all zones of BATHER surveillance responsibility to which they could be assigned;
3) Ability to recognize a victim in their assigned zone of BATHER surveillance;
4) Ability to reach the furthest edge of assigned zones of BATHER surveillance within 20 seconds;
5) Water rescue skills outlined in MAHC Section 6.2.1.1.2;
6) CPR/AED and First Aid;
7) Ability to execute EMERGENCY ACTION PLAN;
8) Emergency closure issues; and
9) Fecal, vomit, and blood contamination incident response as outlined in MAHC Section 6.5.

6.3.3.3.5 CPR / AED and First Aid Certificate

The designated person*(s)* with CPR/AED and first aid training shall present unexpired certificate*(s)* as per MAHC Section 6.2.1.1.3 and 6.2.1.1.4 prior to active duty.

6.3.3.3.5.1 Copies Maintained

Originals or copies of certificates shall be maintained at the AQUATIC FACILITY and be available for inspection.

6.3.3.3.6 Documentation of Pre-Service Training

Documentation verifying the pre-service requirements shall be completed by the person conducting the pre-service training, maintained at the facility for three full years, and be available for inspection.

6.3.3.3.6.1 Lifeguard Certificate

When QUALIFIED LIFEGUARDS are used, they shall present an unexpired certificate as per MAHC Section 6.2.1.3.4 prior to assuming on-duty lifeguard responsibilities.

6.3.3.3.6.2 Copies Maintained

Originals or copies of certificates shall be maintained at the facility and be available for inspection.

6.3.3.4 In-Service Training

During the course of their employment, AQUATIC FACILITY staff shall participate in periodic in-service training to maintain their skills.

6.3.3.4.1 Documentation of In-Service Training

Documentation verifying the in-service requirements shall be completed by the person conducting the in-service training, maintained at the AQUATIC FACILITY for three years, and available for inspection.

6.3.3.4.2 In-Service Documentation

Documentation shall include:

1) Names of attendees,
2) Content of training,
3) Date of training, and
4) Name of the trainer*(s)*.

6.3.3.4.3 In-Service Training Plan

The in-service training plan shall include:

1) In-service training frequency,
2) Documentation of in-service training,
3) Maintenance of certifications, and
4) Demonstration of test-ready skills.

6.3.3.4.4 Maintain Certificates

The designated person*(s)* with CPR/AED and first aid training shall maintain certifications to show the following:

1) CPR/AED training is completed annually and certificates are unexpired, and
2) First aid training certificates are unexpired.

6.3.3.4.5 Competency Demonstration

When QUALIFIED LIFEGUARDS are used, they shall be able to demonstrate proficiency in the skills as outlined by MAHC Section 6.2.1 and have the ability to perform the following water rescue skills consecutively so as to demonstrate the ability to respond to victim and complete the rescue:

1) Reach the furthest edge of zones of BATHER surveillance within 20 seconds;
2) Recover a simulated victim, including extrication to a position of SAFETY consistent with MAHC Section 6.2.1.1.2; and
3) Perform resuscitation skills consistent with MAHC Section 6.2.1.1.3.

6.3.3.5 AHJ Authority to Approve Safety Plan

The AHJ shall have the authority, if they so choose, to require:

1) Submittal of the SAFETY PLAN for archiving and reference, or
2) Submittal of the SAFETY PLAN for review and approval prior to opening to the public.

6.3.3.5.1 Safety Plan on File

The SAFETY PLAN shall be kept on file at the AQUATIC FACILITY.

6.3.3.5.2 Safety Plan Implemented

The elements detailed in the SAFETY PLAN must be implemented and in evidence in the AQUATIC FACILITY operation and is subject to review for compliance by the AHJ at any time.

6.3.4 Staff Management

6.3.4.1 Staff Provided Prior to Aquatic Venue Use

Prior to use of any AQUATIC VENUE, the AQUATIC FACILITY shall provide staff required per the provisions of the SAFETY PLAN as stated in MAHC Section 6.3.2.

6.3.4.2 Safety Team Responsibilities

SAFETY TEAM responsibilities shall include but not be limited to:

1) Enforcing the AQUATIC FACILITY rules and regulations by interfacing with PATRONS;
2) Respond to reported emergencies;
3) Identify health and SAFETY hazards and take action to mitigate or avoid the hazard;
4) Know where personal protective equipment is located and use it when required; and
5) Interface with the AHJ related to the requirements of this CODE.

6.3.4.3 Lifeguard Staff

6.3.4.3.1 Minimum Number of Lifeguards

Where QUALIFIED LIFEGUARDS are used, the AQUATIC FACILITY shall provide, prior to opening the AQUATIC FACILITY to the public, the minimum number of QUALIFIED LIFEGUARDS and staff required per the provisions of the SAFETY PLAN such that:

1) All zones of PATRON surveillance are staffed during operation;
    a. Zones of PATRON surveillance for individual AQUATIC VENUES not open for use, must also be staffed unless an effective means is provided to restrict and monitor access to the AQUATIC VENUE;
2) Rotations can be conducted while all zones are staffed;
3) Supervisor is present; and
4) Additional person*(s)* to rapidly respond to an emergency to help the initial rescuer, as required in Section 6.3.3.2.1, are present.

6.3.4.3.2 Lifeguard Responsibilities

QUALIFIED LIFEGUARD responsibilities shall include but not be limited to:

1) Monitor PATRONS within the zone of PATRON surveillance responsibility;
2) Enforce facility rules;
3) Respond to emergencies including water rescue, CPR, AED use, and First Aid;
4) Identify health and SAFETY hazards and take action to mitigate or avoid the hazard;
5) Maintain skills at a test-ready level of proficiency;
6) Wear the identifying uniform;
7) If needed for effective PATRON surveillance, wear corrective eyewear and/or wear polarized sunglasses;
8) If exposed to UV, wear SPF 15 or greater UV protection; and
9) Know where personal protective equipment is located and use it when required.

6.3.4.3.3 Shallow Water Certified Lifeguards

QUALIFIED LIFEGUARDS certified for shallow water depths shall not be assigned to a BODY OF WATER in which any part of the water's depth is greater than the depth for which they are certified.

6.3.4.3.4 Direct Surveillance

QUALIFIED LIFEGUARDS assigned responsibilities for PATRON surveillance shall not be assigned other tasks that intrude on PATRON surveillance while performing those surveillance activities.

6.3.4.3.5 Distractions

While conducting BATHER surveillance, QUALIFIED LIFEGUARDS shall not engage in social conversations or have on their person or lifeguard station cellular telephones, texting devices, music players, or other similar non-emergency electronic devices.

6.3.4.4 Supervisor Staff

6.3.4.4.1 Lifeguard Supervisor Required

AQUATIC FACILITIES that are required to have two or more QUALIFIED LIFEGUARDS per the zone plan of BATHER surveillance responsibility in MAHC Section 6.3.3.1.1 shall have at least one person located at the AQUATIC FACILITY during operation designated as the LIFEGUARD SUPERVISOR who meets the requirement of MAHC Section 6.2.2.

6.3.4.4.2 Designated Supervisor

One of the QUALIFIED LIFEGUARDS as per MAHC Section 6.3.3.1.1 may be designated as the LIFEGUARD SUPERVISOR in addition to fulfilling the duties of QUALIFIED LIFEGUARD.

6.3.4.4.2.1 Lifeguard Supervisor Duties

LIFEGUARD SUPERVISOR duties shall not interfere with the primary duty of PATRON surveillance.

6.3.4.4.3 Lifeguard Supervisor

LIFEGUARD SUPERVISOR responsibilities shall include but not be limited to:

1) Monitor performance of QUALIFIED LIFEGUARDS in their zone of BATHER surveillance responsibility;
2) Make sure the rotation is conducted in accordance with the SAFETY PLAN;
3) Coordinate staff response and BATHER care during an emergency;
4) Identify health and SAFETY hazards and communicate to staff and management to mitigate or otherwise avoid the hazard; and
5) Make sure the required equipment per MAHC Section 5.8.5 is in place and in good condition.

6.3.4.5 Emergency Response and Communications Plans

6.3.4.5.1 Emergency Response and Communication Plan

AQUATIC FACILITIES shall create and maintain an operating procedure manual containing information on the emergency response and communications plan including an EAP, Facility Evacuation Plan, and Inclement Weather Plan.

6.3.4.5.2 Emergency Action Plan

A written EAP shall be developed, maintained, and updated as necessary for the AQUATIC FACILITY.

6.3.4.5.3 Annual Review and Update

The EAP shall be reviewed with the AQUATIC FACILITY staff and management annually or more frequently as required when changes occur with the dates of the review recorded in the EAP.

6.3.4.5.4 Available for Inspection

The written EAP shall be kept at the AQUATIC FACILITY and available for emergency personnel and/or AHJ upon request.

6.3.4.5.5 Training Documentation

Documentation from employees trained in current EAP shall be available upon request.

6.3.4.5.6 Components

The EAP shall include at a minimum:

1) A diagram of the AQUATIC FACILITY;
2) A list of emergency telephone numbers;
3) The location of first aid kit and other rescue equipment *(bag valve mask, AED, if provided, backboard, etc.)*;
4) An emergency response plan for accidental chemical release; and
5) A fecal/vomit/blood CONTAMINATION RESPONSE PLAN as outlined in MAHC 6.5.1.

6.3.4.5.6.1 Accidental Chemical Release Plan

The accidental chemical release plan shall include procedures for:

1) How to determine when professional HAZMAT response is needed,
2) How to obtain it,
3) Response and cleanup,
4) Provision for training staff in these procedures, and
5) A list of equipment and supplies for clean-up.

6.3.4.5.6.2 Remediation Supplies

The availability of equipment and supplies for remediation procedures shall be verified by the operator at least weekly.

6.3.4.5.7 Facility Evacuation Plan

A written Facility Evacuation Plan shall be developed and maintained for the facility.

6.3.4.5.7.1 Evacuation Plan Components

This plan shall include at a minimum:

1) Actions to be taken in cases of drowning, serious illness or injury, chemical handling accidents, weather emergencies, and other serious incidents; and
2) Defined roles and responsibilities for all staff.

6.3.4.5.8 Communication Plan

A communication plan shall exist to facilitate activation of internal emergency response centers and/or community 911/EMS as necessary.

6.3.4.5.8.1 Communication Plan Components

At a minimum, this plan shall include:

1) Provision and use of readily accessible, appropriate communication devices such as telephones, call boxes, and mobile devices;
2) Signage;
3) Procedures to be followed during staffed and unstaffed time periods;
4) Acceptable alternative communication during loss of power; and
5) Training of all personnel.

6.3.4.5.8.2 Notification Procedures

The communication plan shall include a plan for notification to Federal, State, and local agencies in case of a chemical spill that exceeds the EPA reportable quantity.

6.3.4.5.9 Inclement Weather Plan

AQUATIC FACILITIES shall have a contingency/response plan for localized weather events that may affect their operation *(i.e. lightning, hurricanes, tornados, high winds, etc.)*.

6.3.4.5.9.1 Contingency Plan

Contingency plans shall include training for employees, evacuation procedures, and determining when it is acceptable to re-open a facility for operation.

6.3.4.6 Remote Monitoring Systems

6.3.4.6.1 Lifeguard-Based

Lifeguard-based remote SAFETY MONITORING systems shall not replace the need for QUALIFIED LIFEGUARDS.

6.3.4.6.1.1 No Substitute

Remote SAFETY MONITORING systems may be used to aid the operation but not as a substitute for QUALIFIED LIFEGUARDS/slide operators when critical areas such as blind spots in an AQUATIC VENUE or area of a slide cannot be viewed by QUALIFIED LIFEGUARDS/slide operators.

6.3.4.6.2 Operator-Based

QUALIFIED OPERATOR-based remote water quality MONITORING systems shall not be a substitute for manual water quality testing of the AQUATIC VENUE.

6.3.4.6.3 Training

When QUALIFIED LIFEGUARD- or QUALIFIED OPERATOR-based remote MONITORING systems are used, AQUATIC FACILITY staff shall be trained on their use, limitations, and communication and response protocols for communications with the MONITORING group.

6.3.4.7 Employee Illness and Injury Policy

6.3.4.7.1 Illness Policy

Supervisors shall not permit employees who are ill with diarrhea to enter the water or perform in a QUALIFIED LIFEGUARD role.

6.3.4.7.2 Open Wounds

Supervisors shall permit employees with open wounds in the water or in a QUALIFIED LIFEGUARD role only if they have healthcare provider approval or wear a waterproof, occlusive bandage to cover the wound.

6.4 Facility Management

6.4.1 Operations

6.4.1.1 Operations Manual

6.4.1.1.1 Develop

Each AQUATIC FACILITY shall develop an operations manual to keep at the AQUATIC FACILITY in both printed and electronic formats.

6.4.1.1.2 Include

The manual shall at minimum include, but not be limited to the following items:

1) AQUATIC VENUE and AQUATIC FEATURE description*(s)* and locations,
2) Facility communication,
3) List of chemicals and system information,
4) Fecal/vomit and body fluid contamination response protocols,
5) Preventive maintenance plan, and
6) Any other standard operation and maintenance policies and instructions or applicable information for each AQUATIC VENUE and AQUATIC FEATURE at the facility.

6.4.1.2 Operation Records

AQUATIC FACILITIES shall keep records pertaining to the operation, maintenance, and management of the AQUATIC FACILITY on a minimum schedule as prescribed under MAHC Sections 6.4.1.2.

6.4.1.2.1 Record Maintenance

AQUATIC FACILITY records shall be:

1) Kept for a minimum of three years, and
2) Available upon request by the AHJ.

6.4.1.2.2 Additional Documentation

Local CODES may require additional records, documentation, and forms.

6.4.1.3 Safety and Maintenance Inspection and Recordkeeping

The QUALIFIED OPERATOR or RESPONSIBLE SUPERVISOR shall ensure that SAFETY and preventive maintenance inspections are done at the AQUATIC FACILITY during seasons or periods when the AQUATIC FACILITY is open and that the results are recorded in a log or form maintained at the AQUATIC FACILITY.

6.4.1.3.1 Daily Inspection Items

The QUALIFIED OPERATOR or RESPONSIBLE SUPERVISOR shall ensure that a daily AQUATIC FACILITY preventive maintenance inspection is done before opening and that it shall include:

1) Walkways/DECK and exits are clear, clean, free of debris;
2) Drain covers, vacuum fitting covers, SKIMMER equalizer covers, and any other suction outlet covers are in place, secure, and unbroken;
3) SKIMMER baskets, weirs, lids, flow adjusters, and suction outlets are free of any blockage;
4) INLET and return covers and any other fittings are in place, secure, and unbroken;
5) SAFETY warning signs and other signage are in place and in good repair;
6) Safety equipment as required by this CODE are in place and in good repair, including emergency instructions and phone numbers;
7) Entrapment prevention systems are operational;
8) Recirculation, DISINFECTION systems, controller*(s)*, and probes are operating as required;
9) Secondary and/or supplemental DISINFECTION systems are operating as required;
10) Underwater lights and other lighting are intact with no exposed wires or water in lights;
11) Slime and biofilm has been removed from accessible surfaces of AQUATIC VENUES, slides, and other AQUATIC FEATURES;
12) Doors to nonpublic areas *(CHEMICAL storage spaces, offices, etc.)* are locked;

13) First aid supplies are stocked;
14) Emergency communication equipment and systems are operational;
15) Fecal/vomit/blood incident contamination response protocols, materials, and equipment are available;
16) Water features and amenities are functioning in accordance with the manufacturer's recommendations;
17) Fencing/BARRIERS, gates, and self-latching or other locks are tested and are intact and functioning properly, and BARRIERS do not have nearby furniture to encourage climbing;
18) Drinking fountains are clean and in functional condition;
19) Electrical devices are in good working condition and meet the requirements specified in the NEC and MAHC;
20) Alarms, if required, are tested and functioning properly; and
21) Assessing water clarity such that the bottom and objects in the POOL are clearly visible.

6.4.1.3.2 Other Inspection Items

The QUALIFIED OPERATOR or RESPONSIBLE SUPERVISOR shall ensure that the AQUATIC FACILITY preventive maintenance inspections shall also include:

1) Monthly tests of GFCI devices,
2) Inspections every six months of bonding conductors, where accessible.

6.4.1.4 Illness and Injury Incident Reports

6.4.1.4.1 Incidents to Record

The owner/operator shall ensure that a record is made of all injuries and illness incidents which:

1) Results in deaths;
2) Requires resuscitation, CPR, oxygen or AED use;
3) Requires transportation of the PATRON to a medical facility; or
4) Is a PATRON illness or disease outbreak associated with water quality.

6.4.1.4.2 Info to Include

Illness and injury incident report information shall include

1) Date,
2) Time,
3) Location,
4) Incident including type of illness or injury and cause or mechanism,
5) Names and addresses of the individuals involved,
6) Actions taken,
7) Equipment used, and
8) Outcome of the incident.

6.4.1.4.3 Notify the AHJ

In addition to making such records, the owner/operator shall ensure that the AHJ is notified within 24 hours of the occurrence of an incident recorded in MAHC 6.4.1.4.1.

6.4.1.4.4 Lifeguard Rescues

The owner/operator shall also record all lifeguard rescues where the QUALIFIED LIFEGUARD enters the water or uses other equipment to help a BATHER.

6.4.1.4.4.1 Info to Include

These records shall include the date, time, QUALIFIED LIFEGUARD, and PATRON names and reason the rescue was needed.

6.4.1.5 Chemical Inventory Log

A chemical inventory log shall be maintained on site to provide a list of chemicals used in the AQUATIC VENUE water and surrounding deck that could result in water quality issues, chemical interactions, or PATRON exposure.

6.4.1.5.1 Expiration Dates

These records shall include the expiration date for water quality chemical testing reagents.

6.4.1.6 Daily Water Monitoring and Testing Records

Daily, or as often as required, monitoring and testing records shall include, but are not limited to the following:

1) pH level,
2) Disinfectant residuals,
3) Combined CHLORINE concentrations,
4) Operating pressures of water recirculation pumps and filters or the corresponding flow rate from flow meter readings,
5) Cyanuric acid levels, if used,
6) Maintenance and malfunctioning of equipment, including dates and time of all equipment calibration including WQTDs,
7) If heated, AQUATIC VENUE water temperature,
8) The time of filter backwash or cleaning,
9) Calcium hardness,
10) Total alkalinity,
11) SATURATION INDEX,
12) Microbiological testing, if applicable, dates/times samples were taken and results,
13) Any equipment failure, power outage, or error resulting in the interruption of the circulation, filtration, or DISINFECTION systems for more than one hour,
14) The daily attendance at the AQUATIC FACILITY; in POOLS where attendance is not ordinarily recorded, a guest sign in book can be used to track attendance, and

15) SECONDARY DISINFECTION SYSTEMS as outlined in MAHC Sections 5.7.3.7.7 and 5.7.3.7.8.

6.4.1.7 Staff Certifications on File

The originals or copies of all required QUALIFIED LIFEGUARD, LIFEGUARD SUPERVISOR, or QUALIFIED OPERATOR certificates shall be maintained at the AQUATIC FACILITY and made available to AHJ, staff, and PATRONS upon request.

6.4.1.7.1 Multiple Facilities

A copy of the original certificate shall be made available when employees work at multiple AQUATIC FACILITIES.

6.4.1.8 Bodily Fluids Remediation Log

6.4.1.8.1 Contamination Incidents

A Body Fluid Contamination Response Log shall be maintained to document each occurrence of contamination of the water or its immediately adjacent areas by formed or diarrheal fecal material, whole stomach discharge of vomit, and blood.

6.4.1.8.2 Standard Operating Procedures

The AQUATIC FACILITY'S standard operating procedures for responding to these contamination incidents shall be readily available for review by the AHJ.

6.4.1.8.3 Required Information

The log shall include the following information recorded at the time of the incident:

1) Person conducting response;
2) QUALIFIED OPERATOR or on-site RESPONSIBLE SUPERVISOR on duty;
3) Date and time of incident response;
4) Specific area, if not in the water, contaminated by incident;
5) BATHER COUNT in the AQUATIC VENUE at the time of incident *(if applicable)*;
6) Type and form of body fluid observed *(for example, diarrheal or formed stool, vomitus, or blood)*;
7) Date and time when the area was closed;
8) Whether the POOL uses CHLORINE stabilizer and concentration at time of incident;
9) Free residual disinfectant and pH levels at the time of incident;
10) Remediation procedures used after the incident including contact time, if applicable;
11) Free residual disinfectant and pH level at the time of reopening the AQUATIC VENUE to the public;
12) Stabilizer concentration, if used, at the time of reopening; and
13) Date and time of reopening.

6.4.2 Patron-Related Management Aspects

6.4.2.1 Bather Count

6.4.2.1.1 User Guidelines

AQUATIC FACILITIES that typically operate with low BATHER occupancy shall have a plan in place to adjust to potential higher BATHER use.

6.4.2.1.2 Maximum Occupancy

Such plans shall not exceed the maximum designed THEORETICAL PEAK OCCUPANCY for the individual AQUATIC VENUES or the AQUATIC FACILITY.

6.4.2.2 Signage

6.4.2.2.1 Facility Rules

The operator shall post and enforce the AQUATIC FACILITY rules governing health, SAFETY, and sanitation.

6.4.2.2.2 Lettering

The lettering shall be legible and at least one inch *(25.4 mm or 36 point type)* high, with a contrasting background.

6.4.2.2.3 Sign Messages

Signage shall be placed in a conspicuous place at the entrance of the AQUATIC FACILITY communicating expected and prohibited behaviors and other information using text that complies with the intent of the following information:

1) In case of an emergency, dial 911 or other emergency instructions, per MAHC Section 6.3.4.5.8;
2) Hours of operation;
3) THEORETICAL PEAK OCCUPANCY;
4) Pollution of AQUATIC VENUE prohibited;
5) Do not swim if you have open wounds;
6) Do not swim if you are ill with diarrhea or have had diarrhea within the past two weeks;
7) Shower before entering the water;
8) No glass items in the AQUATIC VENUE or on the DECK;
9) Do not swallow or spit water;
10) Diaper changing on the deck is prohibited;
11) No Diving, as applicable per MAHC Section 5.5.5;
12) Intentional hyperventilation or extended breath holding activities are dangerous and prohibited, if applicable;
13) No animals in the AQUATIC VENUE and no animals on the DECK, except service animals.

6.4.2.2.3.1 Aquatic Facilities with On-site Emergency Personnel

MAHC Section 6.4.2.2.3 signage requirement number one may be amended to include on-site emergency staff contact information if emergency trained personnel are on site so that the response would be faster than calling 911.

6.4.2.2.3.2 Diving Well

AQUATIC FACILITIES with diving wells may amend signage requirement number 11 to read that diving is not allowed in all AQUATIC VENUES except for the diving well.

6.4.2.2.3.3 Posters

Recreational Water Illness Prevention posters shall be posted conspicuously in the AQUATIC FACILITY at all times.

6.4.2.2.3.4 Unstaffed Aquatic Facilities without Lifeguards

In addition to signage messages one through 13, unstaffed AQUATIC FACILITIES shall also include signage messages covering:

1) No Lifeguard on Duty: Children under 14 years of age must have adult supervision, and
2) Hours of operation; AQUATIC FACILITY use prohibited at any other time.

6.4.2.2.3.4.1 Posters

In AQUATIC FACILITIES not requiring lifeguards, CPR posters reflecting the latest standards shall be posted conspicuously at all times.

6.4.2.2.3.5 Multiple Aquatic Venues

For AQUATIC FACILITIES with multiple AQUATIC VENUES, MAHC Section 6.4.2.2.3 signage items numbers three through ten and, if applicable, numbers 11 through 13, or text complying with the intent of the information, shall be posted at the entrance to each AQUATIC VENUE.

6.4.2.2.3.6 Movable Bottom Floor Signage

In addition to the MAHC 6.4.2.2.3 requirements, AQUATIC VENUES with moveable bottom floors shall also have the following information or text complying with the intent of the following information:

1) A sign for AQUATIC VENUE water depth in use shall be provided and clearly visible;
2) A "NO DIVING" sign shall be provided; and
3) The floor is movable and AQUATIC VENUE depth varies.

6.4.2.2.3.7 Spa Signs

In addition to the MAHC Section 6.4.2.2.3 requirements, SPAS shall also have the following information or text complying with the intent of the following information:

1) Maximum water temperature is 104° F *(40°F)*;
2) Children under age five and people using alcohol or drugs that cause drowsiness shall not use SPAS;
3) Pregnant women and people with heart disease, high blood pressure or other health problems should not use SPAS without prior consultation with a healthcare provider;
4) Children under 14 years of age shall be supervised by an adult; and
5) Use of the SPA when alone is prohibited *(if no lifeguards on site).*

6.4.2.2.4 Hygiene Facility Signage

Signage shall be posted at the HYGIENE FACILITY exit used to access AQUATIC VENUES stating or containing information, or text complying with the intent of the following information:

1) Do not swim when ill with diarrhea;
2) Do not swim with open wounds and sores;
3) Shower before entering the water;
4) Check your child's swim diapers/rubber pants regularly;
5) Diaper changing on the DECK is prohibited;
6) Do not poop or pee in the water;
7) Do not swallow or spit water; and
8) Wash hands before returning to the pool.

6.4.2.2.5 Diaper-Changing Station Signage

Signage shall be posted at DIAPER-CHANGING STATIONS stating or containing information, or text complying with the intent of the following information:

1) Dispose of used disposable diapers in the diaper bucket or receptacle provided;
2) Dump contents from reusable diapers into toilets and bag diapers to take home;
3) Use the materials provided to clean/SANITIZE the surface of the diaper-changing station before and after each use;
4) Wash your hands and your child's hands after diapering; and
5) Do not swim if ill with diarrhea.

6.4.2.3 Swimmer Empowerment Methods

6.4.2.3.1 Public Information and Health Messaging

The owner/operator shall ensure that a public information and health messaging program to inform INDOOR AQUATIC FACILITY PATRONS of their impact on INDOOR AQUATIC FACILITY air quality is developed and implemented.

6.4.2.3.2 Post Inspection Results

The results of the most recent AHJ inspection of the AQUATIC FACILITY shall be posted at the AQUATIC FACILITY in a location conspicuous to the public.

6.5 Fecal/Vomit/Blood Contamination Response

6.5.1 Contamination Response Plan

6.5.1.1 Contamination Response Plan

All AQUATIC FACILITIES shall have a CONTAMINATION RESPONSE PLAN within the EMERGENCY ACTION PLAN for responding to formed-stool contamination, diarrheal-stool contamination, vomit contamination, and contamination involving blood.

6.5.1.2 Contamination Training

The CONTAMINATION RESPONSE PLAN shall include procedures for response and cleanup, provisions for training staff in these procedures, and a list of equipment and supplies for clean-up.

6.5.1.2.1 Minimum

A minimum of one person on-site while the AQUATIC FACILITY is open for use shall be:

1) Trained in the procedures for response to formed-stool contamination, diarrheal contamination, vomit contamination, and blood contamination; and
2) Trained in Personal Protective Equipment and other OSHA measures including the Bloodborne Pathogens Standard 29 CFR 1910.1030 to minimize exposure to bodily fluids that may be encountered as employees in an aquatic environment.

6.5.1.2.2 Informed

Staff shall be informed of any updates to the response plan.

6.5.1.3 Equipment and Supply Verification

The availability of equipment and supplies for remediation procedures shall be verified by the QUALIFIED OPERATOR at least weekly.

6.5.1.4 Plan Review

The response plan shall be reviewed at least annually and updated as necessary.

6.5.1.5 Plan Availability

The response plan shall be kept on site and available for viewing by the AHJ.

6.5.2 Aquatic Venue Water Contamination Response

6.5.2.1 Closure

In the event of a fecal or vomit contamination in an AQUATIC VENUE, the QUALIFIED OPERATOR shall immediately close the AQUATIC VENUE to swimmers until remediation procedures are complete.

6.5.2.1.1 Closure Includes

This closure shall include the affected AQUATIC VENUE and other AQUATIC VENUES that share the same RECIRCULATION SYSTEM.

6.5.2.2 Physical Removal

Contaminating material shall be removed *(e.g., using a net, scoop, or bucket)* and disposed of in a sanitary manner.

6.5.2.2.1 Clean / Disinfect Net or Scoop

Fecal or vomit contamination of the item used to remove the contamination *(e.g., the net or bucket)* shall be removed by thorough cleaning followed by DISINFECTION *(e.g., after cleaning, leave the net, scoop, or bucket immersed in the pool during the disinfection procedure prescribed for formed-stool, diarrheal-stool, or vomit contamination, as appropriate)*.

6.5.2.2.2 No Vacuum Cleaners

Aquatic vacuum cleaners shall not be used for removal of contamination from the water or adjacent surfaces unless vacuum waste is discharged to a sanitary sewer and the vacuum equipment can be adequately disinfected.

6.5.2.3 Treated

AQUATIC VENUE water that has been contaminated by feces or vomit shall be treated as follows:

1) Check to ensure that the water's pH is 7.5 or lower and adjust if necessary;
2) Verify and maintain water temperature at 77°F (25°C) or higher;
3) Operate the filtration/RECIRCULATION SYSTEM while the POOL reaches and maintains the proper free CHLORINE concentration during the remediation process;
4) Test the CHLORINE residual at multiple sampling points to ensure the proper free CHLORINE concentration is achieved throughout the POOL for the entire DISINFECTION time; and
5) Use only non-stabilized CHLORINE products to raise the free CHLORINE levels during the remediation.

6.5.3 Aquatic Venue Water Contamination Disinfection

6.5.3.1 Formed-Stool Contamination

Formed-stool contaminated water shall have the FREE CHLORINE RESIDUAL checked and the FREE CHLORINE RESIDUAL raised to 2.0 mg/L *(if less than 2.0 mg/L)* and maintained for at least 25 minutes *(or an equivalent time and concentration to reach the CT VALUE)* before reopening the AQUATIC VENUE.

6.5.3.1.1 Pools Containing Chlorine Stabilizers

In AQUATIC VENUE water that contains cyanuric acid or a stabilized CHLORINE product, water shall be treated by doubling the inactivation time required under MAHC Section 6.5.3.1.

6.5.3.1.2 Measurement of Inactivation Time

Measurement of the inactivation time required shall start when the AQUATIC VENUE reaches the intended free CHLORINE level.

6.5.3.2 Diarrheal-Stool Contamination

Diarrheal-stool contaminated water shall:

1) Check the FREE CHLORINE RESIDUAL and then raise the FREE CHLORINE RESIDUAL to 20.0 mg/L and maintain for at least 12.75 hours (*or an equivalent time and concentration to reach the CT VALUE*) before reopening the AQUATIC VENUE, or
2) Circulate the water through a SECONDARY DISINFECTION SYSTEM to theoretically reduce the number of *Cryptosporidium* OOCYSTS in the AQUATIC VENUE below one OOCYST/100 mL as outlined in MAHC Section 4.7.3.3.2.4.

6.5.3.2.1 Pools Containing Chlorine Stabilizers

In AQUATIC VENUE water that contains cyanuric acid or a stabilized CHLORINE product, water shall be treated by:

1) Lowering the pH to 6.5, raising the FREE CHLORINE RESIDUAL to 40 mg/L using a non-stabilized CHLORINE product, and maintaining at 40 mg/L for at least 30 hours or an equivalent time and concentration needed to reach the CT VALUE. *(Measurement of the inactivation time required shall start when the aquatic venue reaches the intended FREE chlorine level.)* or;
2) Circulating the water through a SECONDARY DISINFECTION SYSTEM to theoretically reduce the number of *Cryptosporidium* OOCYSTS in the AQUATIC VENUE below one OOCYST/100 mL as outlined in MAHC Section 4.7.3.3.2.4 or;
3) Draining the AQUATIC VENUE completely.

6.5.3.3 Vomit-Contamination

Vomit-contaminated water shall have the FREE CHLORINE RESIDUAL checked and the FREE CHLORINE RESIDUAL raised to 2.0 mg/L *(if less than 2.0 mg/L)* and maintained for at least 25 minutes (*or an equivalent time and concentration to reach the CT VALUE*) before reopening the AQUATIC VENUE.

6.5.3.3.1 Pools Containing Chlorine Stabilizers

In AQUATIC VENUE water that contains cyanuric acid or a stabilized CHLORINE product, water shall be treated by doubling the inactivation time required under MAHC Section 6.5.3.3.

6.5.3.3.2 Measurement of the Inactivation Time

Measurement of the inactivation time required shall start when the AQUATIC VENUE reaches the intended free CHLORINE level.

6.5.3.4 Blood-Contamination

Blood contamination of a properly maintained AQUATIC VENUE'S water does not pose a public health risk to swimmers.

6.5.3.4.1 Operators Choose Treatment Method

Operators may choose whether or not to close the AQUATIC VENUE and treat as a formed stool contamination as in MAHC Section 6.5.3.1 to satisfy PATRON concerns.

6.5.3.5 Procedures for Brominated Pools

Formed-stool, diarrheal-stool, or vomit-contaminated water in a brominated AQUATIC VENUE shall have CHLORINE added to the AQUATIC VENUE in an amount that will increase the FREE CHLORINE RESIDUAL to the level specified for the specific type of contamination for the specified time.

6.5.3.5.1 Bromine Residual

The bromine residual shall be adjusted if necessary before reopening the AQUATIC VENUE.

6.5.4 Surface Contamination Cleaning and Disinfection

6.5.4.1 Limit Access

If a bodily fluid, such as feces, vomit, or blood, has contaminated a surface in an AQUATIC FACILITY, facility staff shall limit access to the affected area until remediation procedures have been completed.

6.5.4.2 Clean Surface

Before DISINFECTION, all visible CONTAMINANT shall be cleaned and removed with disposable cleaning products effective with regard to type of CONTAMINANT present, type of surface to be cleaned, and the location within the facility.

6.5.4.3 Contaminant Removal and Disposal

CONTAMINANT removed by cleaning shall be disposed of in a sanitary manner or as required by law.

6.5.4.4 Disinfect Surface

Contaminated surfaces shall be disinfected with one of the following DISINFECTION solutions:

1) A 1:10 dilution of fresh household bleach with water; or
2) An equivalent EPA REGISTERED disinfectant that has been approved for body fluids DISINFECTION.

6.5.4.5 Soak

The disinfectant shall be left to soak on the affected area for a minimum of 20 minutes or as otherwise indicated on the disinfectant label directions.

6.5.4.6 Remove

Disinfectant shall be removed by cleaning and shall be disposed of in a sanitary manner or as required by the AHJ.

6.6 AHJ Inspections

6.6.1 Inspection Process

6.6.1.1 Inspection Authority

The AHJ shall have the right to inspect or investigate the operation and management of an AQUATIC FACILITY.

6.6.1.2 Inspection Scope and Right

Upon presenting proper identification, an authorized employee or agent of the AHJ shall have the right to and be permitted to enter any AQUATIC FACILITY or AQUATIC VENUE area, including the recirculation equipment and piping area, at any reasonable time for the purpose of inspecting the AQUATIC VENUE or AQUATIC FEATURES to do any of the following:

1) Inspect, investigate, or evaluate for compliance with this CODE;
2) Verify compliance with previously written violation orders;
3) Collect samples or specimens;
4) Examine, review, and copy relevant documents and records;
5) Obtain photographic or other evidence needed to enforce this CODE; or
6) Question any person.

6.6.1.3 Based on Risk

An AQUATIC FACILITY'S inspection frequency may be amended based on a risk of recreational water injury and illness.

6.6.1.4 Inspection Interference

It is a violation of this CODE for a person to interfere with, deny, or delay an inspection or investigation conducted by the AHJ.

6.6.2 Publication of Inspection Forms

6.6.2.1 Inspection Form Publication

The AHJ may publish or post on the web or other source the reports of AQUATIC FACILITY inspections.

6.6.3 Imminent Health Hazards

6.6.3.1 Violations Requiring Immediate Correction or Closure

Any of the following violations are IMMINENT HEALTH HAZARDS which shall require immediate correction or immediate POOL closure:

1) Failure to provide adequate supervision and staffing of the AQUATIC FACILITY as prescribed in this CODE;
2) Failure to provide the minimum DISINFECTANT residual levels listed in various sections of this CODE;
3) pH level below 6.5;
4) pH level above 8.0;
5) Failure to continuously operate the AQUATIC VENUE filtration and DISINFECTION equipment;
6) Use of an unapproved or contaminated water supply source for potable water use;
7) Unprotected overhead electrical wires within 20 feet horizontally of the AQUATIC VENUE;
8) Non GFCI protected electrical receptacles within 20 feet of the inside wall of the AQUATIC VENUE;
9) Failure to maintain an emergency lighting source;
10) Absence of all required lifesaving equipment on DECK;
11) AQUATIC VENUE bottom not visible;
12) Total absence of or improper depth markings at an AQUATIC VENUE;
13) Plumbing cross-connections between the drinking water supply and AQUATIC VENUE water or between sewage system and the AQUATIC VENUE including filter backwash facilities;
14) Failure to provide and maintain an ENCLOSURE or BARRIER to inhibit unauthorized access to the AQUATIC FACILITY or AQUATIC VENUE when required;
15) Use of unapproved chemicals or the application of chemicals by unapproved methods to the AQUATIC VENUE water;
16) Broken, unsecured, or missing main drain grate or any submerged suction outlet grate in the AQUATIC VENUE;
17) Number of BATHERS/PATRONS exceeds the THEORETICAL PEAK OCCUPANCY;
18) Broken glass or sharp objects in AQUATIC VENUE or on DECK area; or
19) Any other item determined to be a public health hazard by the AHJ.

6.6.3.1.1 Low pH Violations

If pH testing equipment doesn't measure below 6.5, pH level must be at or below the lowest value of the test equipment.

6.6.3.1.2 High pH Violations

If pH testing equipment doesn't measure above 8.0, pH level must be at or above the highest value of the test equipment.

6.6.4 Enforcement

6.6.4.1 Placarding of Pool

Where an imminent public health hazard is found and remains uncorrected, the AQUATIC VENUE shall be placarded to prohibit use until the hazard is corrected in order to protect the public health or SAFETY of BATHERS.

6.6.4.2 Placard Location

When a placard is used, it shall be conspicuously posted at each entrance leading to the AQUATIC VENUE.

6.6.4.2.1 State Authority

When placed by the AHJ, the placard shall state the authority responsible for its placement.

6.6.4.2.2 Tampering with Placard

When placed by the AHJ, the placard shall indicate that concealment, mutilation, alteration, or removal of it by any person without permission of the AHJ shall constitute a violation of this CODE.

6.6.4.3 Operator Follow-up

Within 15 days of the AHJ placarding an AQUATIC FACILITY, the operator of such AQUATIC FACILITY shall be provided with an opportunity to be heard and present proof that continued operation of the facility does not constitute a danger to the public health.

6.6.4.3.1 Correction of Violation

If the IMMINENT HEALTH HAZARD*(s)* have been corrected, the operator may contact the AHJ prior to the hearing and request a follow-up inspection.

6.6.4.3.2 Hearing

The hearing shall be conducted by the AHJ.

6.6.4.4 Follow-up Inspection

The AHJ shall inspect the premises within two working days of notification that the hazard has been eliminated to remove the placards after verifying correction.

6.6.4.4.1 Other Evidence of Correction

The AHJ may accept other evidence of correction of the hazard in lieu of inspecting the premises.

6.6.5 Enforcement Penalties

6.6.5.1 Liability and Jurisdiction

It shall be unlawful for any person to fail to comply with any of the regulations promulgated pursuant to this CODE.

6.6.5.1.1 Failure to Comply

Any person who fails to comply with any such regulation shall be in violation of this CODE.

6.6.5.1.2 Civil Penalty

For each such offense, violators shall be liable for a potential civil penalty.

6.6.5.2 Continued Violation

Each day, or any part thereof, during which a willful violation of this CODE exists or persists shall constitute a separate violation of this CODE.

6.6.5.3 Falsified Documents

Falsifying or presenting to the AHJ falsified documentation and or certificates shall be a civil violation as specified by the AHJ.

6.6.5.4 Enforcement Process

Upon determining that one or more violations of this CODE exists, the AHJ shall cause a written notice of the violation or violations to be delivered to the owner or operator of the AQUATIC FACILITY that is in violation of this CODE.
